# PHF23 promotes NSCLC proliferation, metastasis, and chemoresistance via stabilization of ACTN4 and activation of the ERK pathway

**DOI:** 10.1038/s41419-023-06069-4

**Published:** 2023-08-25

**Authors:** Ming Cheng, Hongyi Cao, Peifeng Yao, Jingqian Guan, Peihong Wu, Hairu Ji, Siyu Jiang, Yinan Yuan, Lin Fu, Qianqian Zheng, Qingchang Li

**Affiliations:** 1 Department of Pathology, College of Basic Medical Sciences, China Medical University, 110000 Shenyang, Liaoning Province People’s Republic of China; 2 Department of Pathology, The First Hospital of China Medical University, No. 155 NanjingBei Street, Heping District, 110000 Shenyang, Liaoning Province People’s Republic of China; 3 Department of Hand Surgery, Central Hospital affiliated to Shenyang Medical College, 110000 Shenyang, Liaoning Province People’s Republic of China; 4 Department of Pathology, Chengde Medical University, 067000 Chengde, Hebei Province People’s Republic of China; 5 Department of Pathophysiology, College of Basic Medical Sciences, China Medical University, 110000 Shenyang, Liaoning Province People’s Republic of China

**Keywords:** Non-small-cell lung cancer, Cancer genomics

## Abstract

At present, non-small cell lung cancer (NSCLC) is still one of the leading causes of cancer-related deaths. Chemotherapy remains the standard treatment for NSCLC. However, the emergence of chemoresistance is one of the major obstacles to lung cancer treatment. Plant homologous structural domain finger protein 23 (PHF23) plays crucial roles in multiple cell fates. However, the clinical significance and biological role of PHF23 in NSCLC remain elusive. The Cancer Genome Atlas data mining, NCBI/GEO data mining, and western blotting analysis were employed to characterize the expression of PHF23 in NSCLC cell lines and tissues. Statistical analysis of immunohistochemistry and the Kaplan–Meier Plotter database were used to investigate the clinical significance of PHF23. A series of in vivo and in vitro assays, including assays for colony formation, cell viability, 5-ethynyl-2’-deoxyuridine (EDU incorporation) and Transwell migration, flow cytometry, RT-PCR, gene set enrichment analysis, co-immunoprecipitation analysis, and a xenograft tumor model, were performed to demonstrate the effects of PHF23 on the chemosensitivity of NSCLC cells and to clarify the underlying molecular mechanisms. PHF23 is overexpressed in NSCLC cell lines and tissues. High PHF23 levels correlate with short survival times and a poor response to chemotherapy in NSCLC patients. PHF23 overexpression facilitates cell proliferation, migration and sensitizes NSCLC cells to Cisplatin and Docetaxel by promoting DNA damage repair. Alpha-actinin-4 (ACTN4), as a downstream regulator, interacts with PHD domain of PHF23. Moreover, PHF23 is involved in ACTN4 stabilization by inhibiting its ubiquitination level. These results show that PHF23 plays an important role in the development and progression of NSCLC and suggest that PHF23 may serve as a therapeutic target in NSCLC patients.

## Background

Worldwide, lung cancer is the second most common cancer and remains a leading cause of cancer incidence and mortality in 2020, accounting for approximately one in ten (11.4%) cancer diagnoses and one in five (18.0%) cancer-related deaths [1]. Lung cancer patients in most countries have a 5-year survival rate of only 10–20% after diagnosis [2]. Non-small cell lung cancer (NSCLC), which accounts for 85% of lung cancers, has a 5-year survival rate of less than 15% [3, 4]. Platinum-based therapy, usually in combination with immunotherapy, is the mainstay of chemotherapy for NSCLC, especially for patients who cannot benefit from targeted therapies [5]. Although chemotherapy has improved the prognosis of NSCLC patients, chemoresistance remains one of the major barriers to lung cancer treatment.

The gene for human plant homologous structural domain finger protein 23 (PHF23) is located on chromosome 17p13.1 and contains five exons. PHF23 protein, consisting of 403 amino acids, is widely expressed in various human tissues. Wang et al. found that PHF23 is a negative regulator of autophagy through the signaling pathway for leucine-rich and sterile alpha motif containing 1 (LRSAM1) [6], an E3 ubiquitin ligase. Furthermore, in osteoarthritis, PHF23 inhibited cellular activation of mitochondrial phagocytosis levels through activation of the AMPK/mTOR/S6K signaling pathway, thus promoting IL-1β-mediated cellular autophagy [7]. However, the expression and mechanisms underlying the oncogenic function of PHF23 in NSCLC remain largely unknown. Few studies have examined the specific role of PHF23 in tumorigenesis. For potential clinical implementation and future drug development, further investigation is needed to determine the exact role and detailed mechanisms of action of PHF23 in NSCLC.

Alpha-actinin-4 (ACTN4) is a member of the spectrin superfamily and an actin-binding protein, with four isoforms: ACTN1, ACTN2, ACTN3, and ACTN4 [8]. While ACTN2 and ACTN3 are expressed in muscle, ACTN1 and ACTN4 are commonly expressed in non-muscle cells [9]. Previous studies have shown that ACTN4 promotes tumor cell proliferation, migration, and invasion through the NF-κB pathway in osteosarcoma [10] and increases the radioresistance and invasiveness of breast cancer cells through the AKT pathway [11]. In NSCLC, high ACTN4 expression is associated with lymph node metastasis [12], and it selectively affects DNA double-strand break repair [13]. Meanwhile, ACTN4 is also involved in the progression of several diseases, including melanoma, hepatocellular carcinoma, nasopharyngeal carcinoma, and ovarian clear-cell adenocarcinomas [14–17].

In this study, we investigated the novel role of PHF23 in NSCLC chemoresistance and DNA repair. Our results demonstrated that PHF23 acts as an oncogenic protein through the regulation of ACTN4 expression. We also found that PHF23 competitively interacts with ACTN4 via the plant homeodomain (PHD) and stabilizes ACTN4 protein, avoiding ubiquitin-dependent proteasomal degradation by RNF38. The PHF23/ACTN4 axis facilitates the activation of the extracellular signal-regulated kinase (ERK)-c-Myc pathway. These findings provide the theoretical and experimental basis for further development of NSCLC therapy.

## Materials and methods

### Patients and specimens

This retrospective study included 104 NSCLC patients who did not harbor driver gene mutations and had not previously received radiotherapy, chemotherapy, and underwent surgery in 2017–2020 at the First Affiliated Hospital of China Medical University. Among 104 samples, eight pairs of NSCLC tumors and paracancerous lung tissues were randomly selected for immunoblot analysis after surgery. The study was approved by the Medical Research Ethics Committee of China Medical University, and informed consent was obtained from all patients.

### Bioinformatics analysis

PHF23 mRNA expression in 33 tumors was examined in The Cancer Genome Atlas (TCGA) database, as well as its expression in 999 lung cancer tissue samples and 103 normal lung tissue samples. The correlation between PHF23 and survival was analyzed using the Kaplan–Meier mapper website (http://kmplot.com/analysis/). Gene set enrichment analysis (GSEA) was performed by TCGA. Genes or molecular pathways that are differentially expressed were analyzed after dividing the samples into high and low groups according to gene expression. Correlations between genes were analyzed using the GEPIA website (http://gepia.cancer-pku.cn/). TCGA data were analyzed using the R package limma, beeswarm in R (version 4.1.0).

### Cell culture

NSCLC cell lines H1299, A549, H460, H1975, SK-MES-1, and HCC827 were obtained from the cell bank of Shanghai Institutes of Biological Sciences (Shanghai, China), and normal bronchial epithelial HBE cells were obtained from American Type Culture Collection (Manassas, VA, USA). H1299, A549, H460, and H1975 cells were cultured in RPMI 1640 medium (Gibco, State of California, USA), while SK-MES-1 cells were cultured in minimum essential medium containing 1.5 g/l sodium bicarbonate and 0.11 g/l sodium pyruvate, and HBE and HCC827 cells were cultured in Dulbecco’s modified Eagle medium (Gibco, State of California, USA) containing 1.5 g/l sodium bicarbonate. All media were supplemented with 10% fetal bovine serum (Clark Biosciences, Richmond, VA, USA). Cells were cultured in a humidified 5% CO_2_ incubator at 37 °C. All human cell lines have been authenticated using STR profiling. H1299 was derived from lymph node tissue from a patient with lymph node metastasis of NSCLC with deletion of the *TP53* gene. A549 was a lung adenocarcinoma cell line with the *KRASG12S* mutation. H1975 was obtained from a patient with lung adenocarcinoma with the presence of *EGFRT790M*, *TP53R273H*, and *PIK3CAG118D* mutations.

### Plasmids and CRISPR-CAS9

The expression plasmid of PHF23 (RC200931) was purchased from Origene Technologies (Rockville, MD, USA). The target sequence of PHF23 sgRNA was 5′-CACCGACCCGCATCTCGCCTTCACTGG-3′. ACTN4 siRNA (stB0005477) were purchased from Ruibo Biosciences (Guangzhou, China). Cells were transfected using Lipofectamine 3000 reagent (Invitrogen, Carlsbad, USA). Transfections were performed according to the manufacturer’s instructions. Stable overexpression cell lines were screened with G418 drug at a concentration of 3.2 µmol/mL in H1299 and 3 µmol/mL in A549. Stable knockoff cell lines were screened with puromycin. The drug concentration was 12.5 µmol/mL in H1299 and 10 µmol/mL in H1975.

### Immunohistochemistry

Tissue sections were incubated with PHF23 rabbit polyclonal antibody (1:300) at 4 °C overnight. Thereafter, they were incubated with the secondary antibody for 2 h at 37 °C. Cell nuclei were stained with hematoxylin for 10 min, 100 μL of 3,3′-diaminobenzidine chromogenic solution (P0202, Bayotime Biotechnology, Shanghai, China) was added to each tissue section, and PHF23 protein expression was observed under a microscope. The intensity of PHF23 staining was scored single-blind as follows: 0 (no staining), 1 (weak staining), 2 (moderate staining), and 3 (strong staining). The percentage of stained cells was scored as 0 (0%), 1 (1–30%), 2 (31–70%), and 3 (71–100%). The two scores for each tumor sample were multiplied to obtain a score of 0–9, with scores ≥3 considered PHF23-positive and scores <3 considered PHF23-negative. A *χ*^2^ test was used to determine the correlation between PHF23 expression and clinicopathological features. *P* < 0.05 was considered to represent a statistically significant difference.

### Nuclear and cytoplasmic protein extraction

Add PMSF-added Cytoplasmic Protein Extraction Reagent A (P0027-1, Beyotime, Biotechnology) to the cell sediment. Shake vigorously and ice bath for 10–15 min. Add Cytoplasmic Protein Extraction Reagent B (P0027-2, Beyotime, Biotechnology). Shake vigorously and centrifuge at 12,000 × *g* for 5 min at 4 °C. Immediately aspirate the supernatant for cytoplasmic proteins. Add Cytosolic Protein Extraction Reagent (P0027-3, Beyotime, Biotechnology) to the precipitate. Shake vigorously and centrifuge at 4 °C 12,000 × *g* for 10 min and aspirate the supernatant for nuclear protein.

### Protein blotting

Cells were lysed with 1% PMSF + NP40 (Beyotime Biotechnology) for 30 min and centrifuged at 12,000 × *g* for 20 min at 4 °C. Protein concentration was determined using a BCA Protein Assay Kit (Beyotime Biotechnology). A total of 40 μg protein was separated on a 10% SDS-PAGE gel and transferred to polyvinylidene difluoride membranes in a wet blotting transfer device. The membranes were blocked with milk in Tris-buffered saline containing 0.05% Tween 20 for 2 h at room temperature. The membranes were incubated with different primary antibodies overnight at 4 °C followed by horseradish peroxidase-labeled goat anti-rabbit or goat anti-mouse incubation at room temperature for 1.5 h. The primary antibody dilution ratios are shown in Table 1. Target proteins were visualized using an enhanced chemiluminescence (ECL) kit (Tanon Science and Technology, Shanghai, China).

**Table 1:** Antibodies for western blot.

Target	Source	Catalog number	Host	Dilution
PHF23	Abcam	ab264292	Rabbit	1:1000
GAPDH	Proteintech	10494-1-AP	Rabbit	1:5000
Cyclin D1	Proteintech	26939-1-AP	Mouse	1:1000
Cyclin A2	Proteintech	18202-1-AP	Rabbit	1:1000
Cyclin E1	Proteintech	11554-1-AP	Rabbit	1:1000
MMP9	Proteintech	10375-2-AP	Rabbit	1:1000
CDK4	Proteintech	11026-1-AP	Rabbit	1:1000
CDK6	Proteintech	14052-1-AP	Rabbit	1:1000
E-cadherin	Proteintech	20874-1-AP	Rabbit	1:5000
N-cadherin	Proteintech	22018-1-AP	Rabbit	1:2000
ERK1/2	Proteintech	11257-1-AP	Rabbit	1:1000
Phospho-JUN (Ser73)	Proteintech	28891-1-AP	Rabbit	1:1000
ZO-1	Proteintech	21773-1-AP	Rabbit	1:1000
Vimentin	Proteintech	10366-1-AP	Rabbit	1:500
MMP2	Proteintech	10373-2-AP	Rabbit	1:1000
PARP1	Proteintech	22999-1-AP	Rabbit	1:1000
BAX	Proteintech	50599-2-Ig	Rabbit	1:1000
Caspase-3/p17/p19	Proteintech	19677-1-AP	Rabbit	1:1000
p38	Abmart	Q16539	Rabbit	1:1000
Phospho-Erk1(T202/Y204)+Erk2(T185/Y187)	Abmart	P27361	Rabbit	1:1000
c-myc	Abmart	P01106	Rabbit	1:1000
Bcl2	Abmart	T40056	Rabbit	1:1000
Cleaved caspase-3(Asp175)	Cell Signaling Technology (CST)	# 9664S	Rabbit	1:1000

### Real-time PCR

Total RNA was extracted from cells using an RNA extraction kit (RC112-01, Vazyme, Nanjing, China) according to the manufacturer’s protocol. A cDNA synthesis kit (Nanjing Vazyme Biotech Co, Nanjing, China) was used to perform reverse transcription with 1 μg RNA. Quantitative RT-PCR experiments were performed using SYBR Green PCR Master Mix in a total volume of 20 μL in the 7900HT Fast Real-Time PCR System (Applied Biosystems, Foster City, CA, USA). The conditions were as follows: Hold stage: 95 °C for 30 s; PCR stage step1: 95 °C for 10 s; step2: 60 °C for 30 s for 40 cycles; melt stage: 95 °C for 15 s and 60 °C for 60 s. Gene expression relative to glyceraldehyde-3-phosphate dehydrogenase was calculated using the 2^-ΔΔCt^ method. The list of primers is shown in Table 2.

**Table 2:** Primers for real-time PCR.

Genes	Primers (5′–3′)
PHF23-forward	GACAGTGCTACCTTGCTTGAG
PHF23-reverse	TCGGTTCTTTCGGTCCTTCTT
ACTN4-forward	ACATAGCCGATTCTCTGCCC
ACTN4-reverse	AAACCATCAACCACCAGGCA
GAPDH-forward	GGAGCGAGATCCCTCCAAAAT
GAPDH-reverse	GGCTGTTGTCATAACTTCTCATGG

### Cell viability assay

Cells were digested with trypsin, counted, and inoculated into 96-well plates (H1299, A549: 2 × 10^3^ cells; H1975: 3 × 10^3^ cells) in 100 μL medium containing 10% fetal bovine serum. Cell Counting Kit-8 (CCK8) reagent (10 μL; MedChemExpress, Monmouth Junction, NJ, USA) was added to each well. The cells were then incubated in an incubator containing 5% CO_2_ at 37 °C for 1.5 h. The absorbance was measured at 450 nm using an enzyme marker (Bio-Rad Laboratories, Hercules, CA, USA) on days 1–5. In cytotoxicity assays, 5 × 10^3^ A549 or H1299 cells were incubated for 24 h. Cisplatin (0, 6, 12, 24, 36, 48, 60, or 72 μM) or docetaxel (0, 6, 12, 24, 36, 48, 60, or 72 μM) was applied for 24 h.

### Colony formation assay

Six-well plates were inoculated with 500 cells per well and incubated in a cell incubator for 10–14 days. The plates were washed with phosphate-buffered saline (PBS), fixed in pre-cooled methanol for 10 min, and then stained with crystal violet for 10 min. Colonies were counted and photographed.

### Transwell assay analysis

PHF23 protein-expressing lung cancer cells (4 × 10^5^ cells per 200 μL) were inoculated into Transwells (8-μm pore size, Corning, Inc., Corning, NY, USA) for 24 h to observe their migratory capacity. Cells that crossed the Transwell membrane were stained with crystal violet, counted manually, and photographed.

### Flow cytometry for cell cycle analysis

A total of 1 × 10^6^ cells were collected, fixed in 70% alcohol, washed with PBS, and stained with 500 ml propidium iodide/RNase staining solution (Roche, Indianapolis, IN, USA) at 37 °C for 30 min. Data were collected using a BD Bioscience (San Jose, CA, USA) system. A one-parameter histogram was plotted based on the nuclear DNA content of each cell detected by flow cytometry. Cells at each stage of the cell cycle were identified based on their DNA ploidy profiles.

### EDU staining

Cells were inoculated into 24-well culture plates for 24 h. A concentration of 50 μM 5-ethynyl-2’-deoxyuridine (EDU; C0075S-1, Beyotime, Biotechnology) was added and incubated for 1.5 h. Cells were then washed with PBS, fixed with 4% paraformaldehyde for 15 min, and permeabilized with 0.5% Triton X-100 for 10 min. Subsequently, cells were treated with 200 μL Click Additive Solution (C0075S-5, Beyotime, Biotechnology) at room temperature and protected from light for 30 min. Cells were then stained with 200 μL of 1× Hoechst 33342 (C0075S-6, Beyotime Biotechnology) at room temperature for 30 min under a laser scanning confocal focusing microscope (FV-1000; Olympus, Tokyo, Japan). Five areas of positive cells were randomly counted.

### Immunofluorescence

Appropriate numbers of cells were inoculated into 2-cm culture dishes (801002, NEST, Hong Kong, China), fixed in 4% paraformaldehyde for 15 min, and treated with 0.1% Triton X-100 for 10 min. Subsequently, they were blocked with 3% bovine serum albumin for 1 h. Cells were left overnight at 4 °C with PHF23 primary antibody. Next, cells were washed three times with PBS and incubated with a secondary antibody at 37 °C for 2 h. Cell nuclei were stained with DAPI for 10 min. Finally, images were taken using the confocal microscope (FV-1000; Olympus).

### Co-immunoprecipitation analysis

Cells were grown in 10-cm cell culture dishes, and when fully grown, they were lysed with lysis solution and centrifuged at 13,400 × *g* for 20 min at 4 °C. Next, 40 μL of protein A/G agarose (P2012; Beyotime Biotechnology) were added to the supernatant and mixed for 4 h. The supernatant was then centrifuged at 4 °C for 5 min at 1000 rpm, and the supernatant was divided into two parts. One part was added to the target antibody (6 µg), and the other part was added to anti-rabbit IgG (1:5000; Beyotime Biotechnology) with shaking at 4 °C overnight. The next day, 25 μL of agarose A/G magnetic beads were added to each tube and incubated at 4 °C for 6 h. The magnetic beads were then washed three times with lysate buffer and heated continuously in boiling water for 10 min before protein immunoblotting.

### Ubiquitination assays and immunoprecipitation

The stably transfected cell lines were transfected with the Ub-HA plasmid, and the 26 S proteasome inhibitor MG132 (HY-13259, MedChemExpress, Monmouth Junction, NJ, USA) was added at a final concentration of 20 μM. The experiments were performed according to the steps of co-immunoprecipitation analysis. The immune complexes were collected by centrifugation, washed in cell lysis buffer, and subjected to immunoblot analysis.

### Cell-derived xenograft model

The nude mice used in this study were handled following the ethical guidelines for laboratory animals issued by China Medical University. Female BALB/c nude mice (4–5 weeks old) were purchased from Beijing Weitonglihua Laboratory Animal Technology Co., Ltd. and randomly divided into six groups (five mice in each group). Then, 150 µL of RPMI 1640 medium containing 3 × 10^6^ H1299-NC, H1299-PHF23 or H1299-PHF23^∆PHD^ cells were injected into the nude mice subcutaneously. Tumor volumes were measured every 3 days using vernier calipers and calculated as 0.5×length×width^2^. When tumor volumes reached ~200 mm^3^ (12 days after cell inoculation), mice were injected intraperitoneally with saline (control group) or cisplatin (5 mg/kg, experimental group) every 3 days. At 3 weeks later, xenograft tumors were executed, excised, weighed, and fixed in 4% formalin for further studies.

### Statistical analysis

Statistical analysis was performed using the SPSS 19.0 statistical package (IBM, Armonk, NY, USA) or GraphPad Prism 6.0 software (Graphpad Software, Inc., San Diego, CA, USA). The cardinality test was used to analyze the relationship between PHF23 expression and clinicopathological characteristics. Two-tailed, unpaired *t* tests or one-way ANOVA tests were used for comparisons between groups to obtain their statistical significance. All experiments were repeated at least three times independently under the same conditions. *P* values < 0.05 were considered statistically significant.

## Results

### PHF23 is upregulated in NSCLC, and high PHF23 expression predicts poor survival of NSCLC patients

**Fig. 1:**
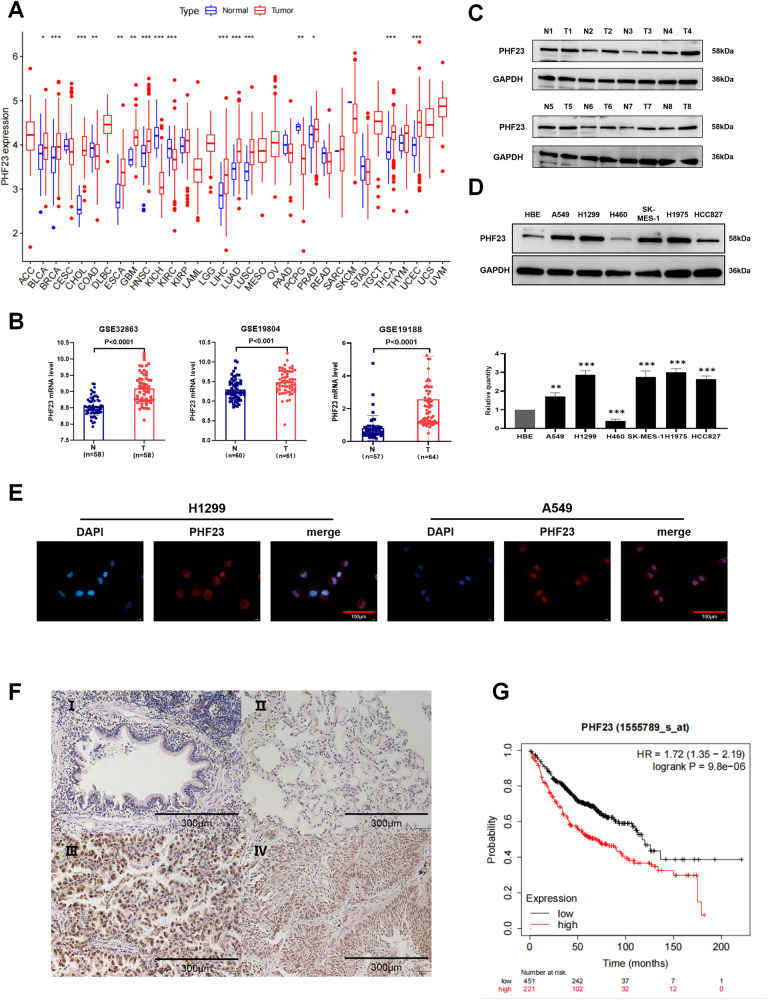
PHF23 is expressed in lung cancer and associated with poor prognosis. **A** PHF23 mRNA expression was investigated in the TCGA database and compared among 33 kinds of tumors. **B** Analysis of the expression of PHF23 mRNA in NSCLC datasets from the GEO database. **C** Western blotting analyses of PHF23 levels in eight lung cancer tissues and matched normal tissues. N normal tissue, T tumor tissue. **D** Western blotting analyses of PHF23 expression in lung epithelial cell line, HBE, and six NSCLC cell lines. Error bars represent the mean ± SD from three independent experiments. *P* values are calculated by a two-tailed, unpaired *t* test. *P < 0.05; *P < 0.01; ***P < 0.001. **E** Immunofluorescence was performed to detect PHF23 localization in H1299 and A549 cell lines. **F** PHF23 levels in normal bronchial epithelial cells (I) and alveolar (II), adenocarcinoma (III), and squamous cell carcinoma (IV) using immunohistochemistry. Magnification: ×200. **G** Kaplan–Meier analysis of overall survival in all NSCLC patients.

We explored the expression of PHF23 in tumors using TCGA and found that PHF23 mRNA was highly expressed in various tumor tissues (Fig. 1A), including lung adenocarcinoma and lung squamous carcinoma tissues (Supplementary Fig. 1A). The same results were confirmed in the GSE32863, GSE19804, and GSE19188 databases (Fig. 1B).

Western blot assays of clinical lung cancer tissues and paraneoplastic tissues showed that PHF23 protein was significantly higher in tumors than in paraneoplastic tissues (Fig. 1C). To further investigate the expression of PHF23 in NSCLC cells, we examined multiple lung cancer cell lines and found that PHF23 was highly expressed in multiple lung cancer cell lines except for the NCI-H460 cell line, compared with normal bronchial epithelial HBE cells (Fig. 1D). Immunofluorescence experiments further verified that PHF23 was widely present in NSCLC cells and was mainly localized to the nucleus and cytoplasm (Fig. 1E).

Immunohistochemical experiments revealed that PHF23 protein was highly expressed in lung cancer tissues and weakly expressed in normal bronchial epithelial and alveolar tissues (Fig. 1F). Correlation analysis with clinicopathological factors revealed that PHF23 expression was positively correlated with tumor differentiation (*P* = 0.011), tumor size (*P* = 0.002), lymph node metastasis (*P* = 0.005), and TMN stage (*P* = 0.009; Table 3). The correlation between PHF23 and survival was analyzed using the Kaplan–Meier mapping website, and the results indicated that higher PHF23 expression was associated with shorter overall survival (OS), first progression survival (FPS) and post-progression survival (PPS; Fig. 1G and Supplementary Fig. 1B, C). Prediction of samples from TCGA revealed that patients’ survival at 1, 2, and 3 years decreased significantly with elevated expression of PHF23 (Supplementary Fig. 1D). Overall, our results suggest that PHF23 may serve as a potential therapeutic target for NSCLC.

**Table 3:** Association of PHF23 expression with clinical and pathological characteristics of patients.

Factors	Features	Number of samples	PHF23 expression		*P* value
			Negative	Positive	
Gender	Male	65	21	44	0.055
	Famale	39	20	19	
Age (years)	≤60	57	25	32	0.308
	>60	47	16	31	
Histological type	Squamous carcinoma	66	30	36	0.097
	Adenocarcinoma	38	11	27	
Differentiation	Poor-moderate	41	10	31	**0.011**
	Well	63	31	32	
Tumor size (cm)	≤3	65	18	47	**0.002**
	>3	39	23	16	
Lymph node status	Negative	61	31	30	**0.005**
	Positive	43	10	33	
TNM stage	I	52	27	25	**0.009**
	II–III	52	14	38	

TNM Eighth Edition of tumor-node-metastasis classification.

The bold and underlined values were considered statistically significant.

### PHF23 overexpression promotes malignant progression of NSCLC in vitro

**Fig. 2:**
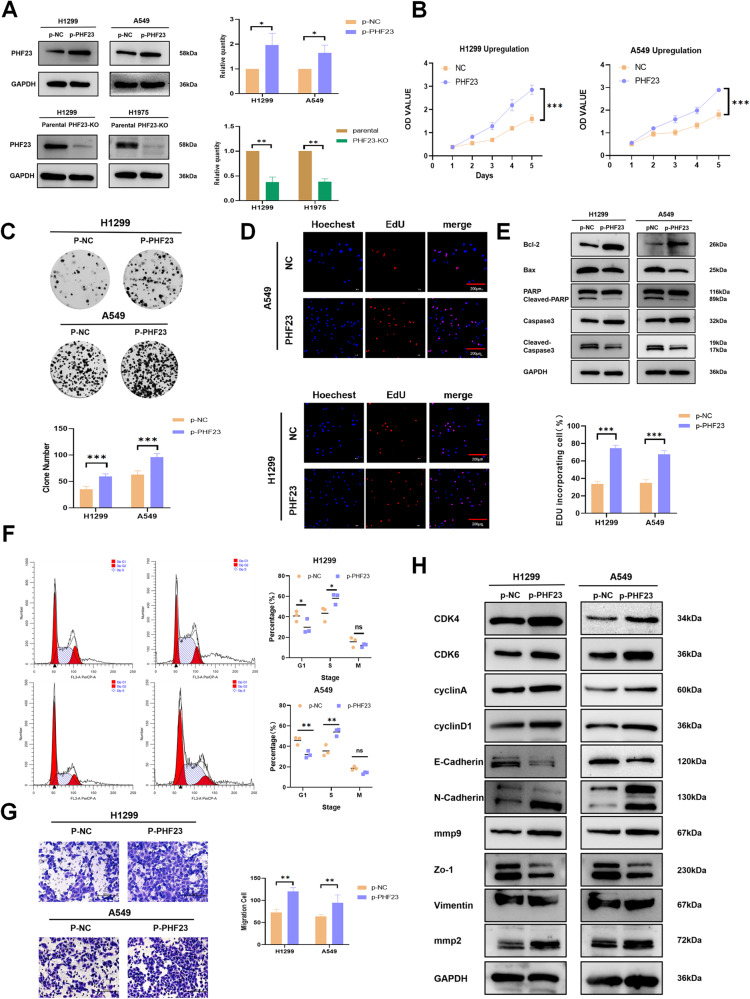
PHF23 promotes the proliferation and migration of NSCLC cells. **A** Western blotting analyzing the expression of PHF23 in the indicated cells. *P < 0.05; *P < 0.01. **B** Cell viability was analyzed by CCK8. Mean ± SD, *n* = 3. ***P < 0.001. **C** Cell growth was determined by colony formation. Mean ± SD, *n* = 3. *P < 0.05; **P < 0.01; ***P < 0.001. **D** DNA replication of H1299 and A549 cells was determined by EDU staining. Scale bar: 200 μm. Mean ± SD, *n* = 3. *P < 0.05; *P < 0.01; ***P < 0.001. **E** Expression of apoptosis proteins in A549 and H1299 cells. **F** Cell cycle of H1299 and A549 cells was analyzed by flow cytometry. Mean ± SD, *n* = 3. *P < 0.05; **P < 0.01, ***P < 0.001. **G** Cell migration was evaluated by the Transwell migration assay; cells that migrated to the lower chamber were stained with hematoxylin and counted. **P < 0.01. **H** Expression of cell proliferation- and migration-related proteins in A549 and H1299.

Given the correlation between PHF23 and tumor size and lymph node metastasis, we hypothesized that PHF23 might also be associated with cell proliferation and migration in lung cancer cells. We selected three NSCLC cell lines, H1299, A549, and H1975, in which PHF23 was differentially expressed for further studies. PHF23 expression was stably upregulated in H1299 and A549 cells and stably knocked down in H1299 and H1975 cells (Fig. 2A).

In vitro growth of lung cancer cells was assessed by CCK8, colony formation, and EDU incorporation assays, which revealed that strong expression of PHF23 promoted high proliferation of lung cancer cells (Fig. 2B–E and Supplementary Fig. 2A–C). The effect of PHF23 on the cell cycle was further examined by flow cytometry. Overexpression of PHF23 increased the S phase fraction, but decreased the G1 phase fraction (Fig. 2F and Supplementary Fig. 2D), indicating that PHF23 overexpression promoted the G1/S transition. Consistent with this finding, PHF23 overexpression increased the expression of G1/S transition-related factors such as CDK4, CDK6, Cyclin D1, and Cyclin A2 (Fig. 2H and Supplementary Fig. 2F). Moreover, PHF23 expression was significantly associated with the increased migration (Fig. 2G and Supplementary Fig. 2E). Overexpression of PHF23 also upregulated MMP2, MMP9, and N-Cadherin gene expression and downregulated E-Cadherin expression. Conversely, knockdown of PHF23 inhibited the proliferation, G1/S transition, and migration of lung cancer cells (Fig. 2H and Supplementary Fig. 2F).

### PHF23 enhances chemotherapy resistance and DNA damage repair in NSCLC

**Fig. 3:**
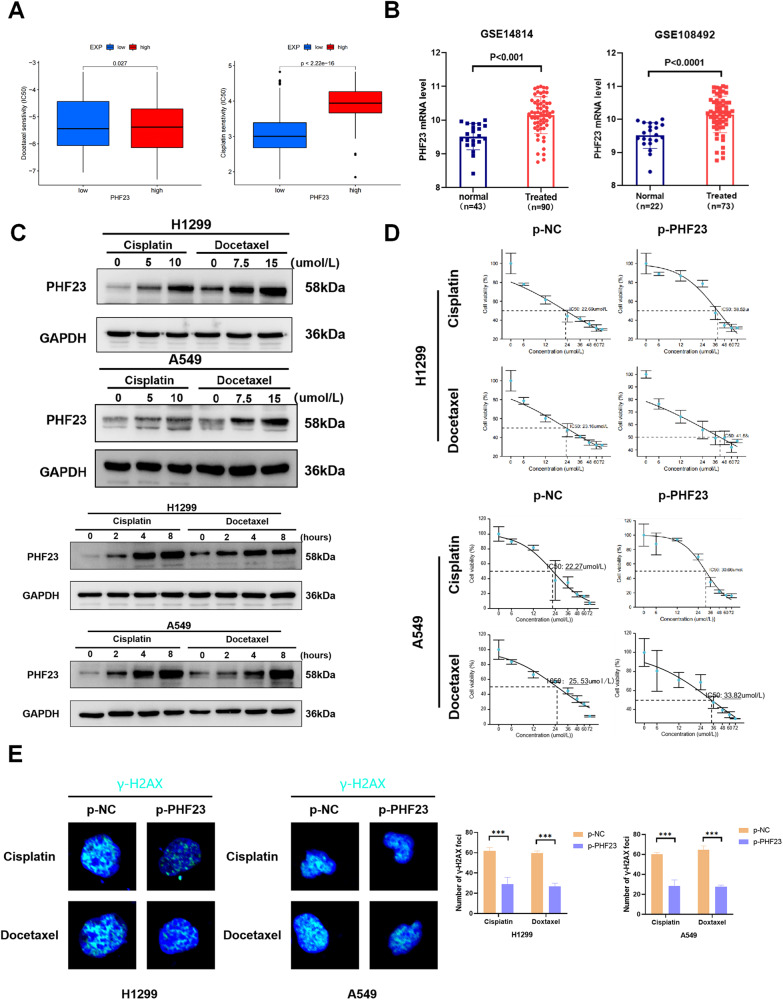
PHF23 enhances chemotherapy resistance and DNA damage repair in lung cancer cells. **A** IC50 analysis of NSCLC patients with high and low PHF23 expression based on TCGA data. **B** Analysis of the expression of PHF23 mRNA in NSCLC datasets from the GEO database. **C** Western blotting analyzing the expression of PHF23 in the H1299 and A549 with increasing concentration and duration of action of cisplatin and docetaxel. **D** Viability of H1299 and A54 cells was analyzed by CCK8 24 h after treatment with different concentrations of cisplatin or docetaxel. **E** γ-H2AX foci formation in H1299 and A549 cells was detected by Immunofluorescence 24 h after treatment with cisplatin (15 μmol/L) or docetaxel (25 μmol/L). Mean ± SD, *n* = 3. ***P < 0.001.

Chemotherapy is a key treatment for NSCLC and is known to improve patient prognosis. A comprehensive analysis of lung cancer samples from TCGA revealed that upregulation of PHF23 increased the 50% inhibitory concentration (IC50) of cisplatin and docetaxel, two commonly used drugs for chemotherapy in NSCLC (Fig. 3A). This suggests that PHF23 may be involved in the development of chemoresistance in lung cancer. Correlation analysis of some common drug resistance proteins revealed that PHF23 was positively correlated with ABCC1, ABCC2, ABCC3, ABCC4, and PGP (Supplementary Fig. 3A). Analysis of the GSE14814 and GSE108492 datasets revealed high expression of PHF23 after treatment with chemotherapeutic agents (Fig. 3B).

Moreover, PHF23 was upregulated in the cisplatin-resistant cell line A549-DDP (Supplementary Fig. 3B). PHF23 protein levels increased with increasing chemotherapeutic drug concentration and duration of action in H1299 and A549 cell lines (Fig. 3C). Colony formation assays showed that PHF23 overexpressing cells were more likely to survive when treated with cisplatin compared to vector cells (Supplementary Fig. 3B). CCK8 assays of cell viability showed that the IC50 of PHF23 overexpressing cells in response to cisplatin and docetaxel were much higher than those of vector control cells (Fig. 3D). Since chemotherapeutic drugs cause cellular DNA damage and DNA replication is associated with DNA damage repair (DDR), the role of PHF23 in DDR was further explored. After adding the effect of chemotherapeutic drugs, staining for γ-H2AX, as a marker of DNA double-stranded breaks, was less in H1299 and A549 cells compared with control cells, indicating that PHF23 overexpression promoted DDR (Fig. 3E) [18]. These data suggest that upregulation of PHF23 induces chemoresistance in lung cancer.

### PHF23 facilitates cell proliferation and metastasis via the ERK signaling pathway in NSCLC

**Fig. 4:**
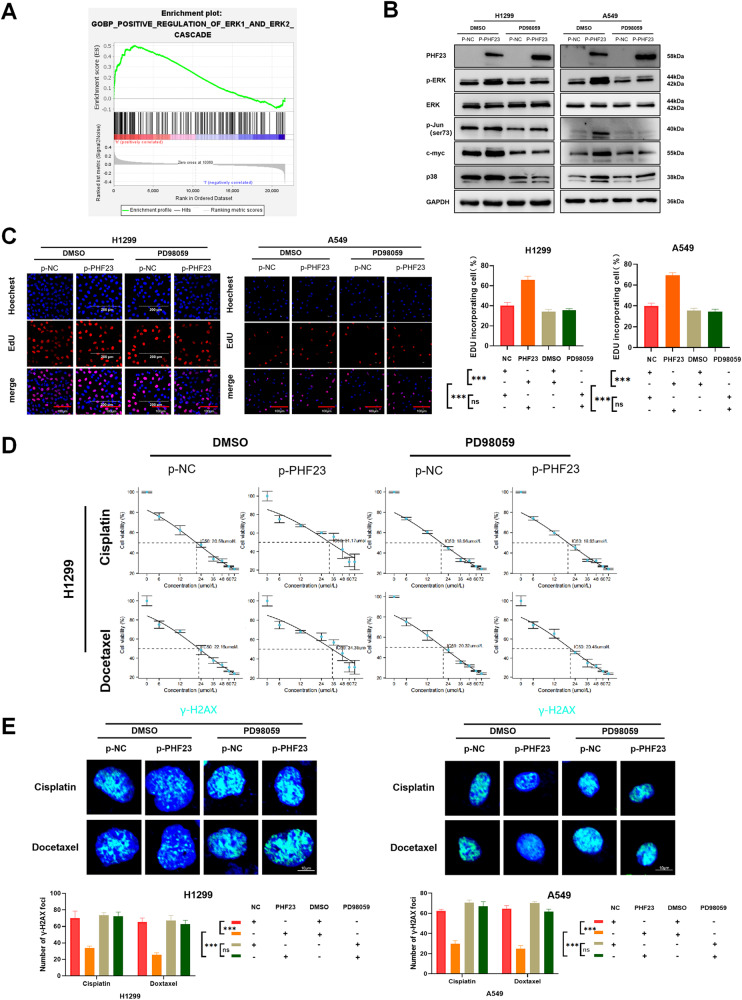
PHF23 promotes lung cancer progression through the activation of ERK signaling pathway. **A** GSEA in the TCGA database was performed. PHF23-related enrichment plots were shown. **B** Expression of proteins involved in the ERK signaling in A549 and H1299 cells treated with DMSO or the ERK inhibitor PD98059. **C** DNA replication of H1299 and A549 cells was determined by EDU staining with DMSO or the ERK inhibitor PD98059. Scale bar: 200 μm. Mean ± SD, *n* = 3. *P < 0.05; **P < 0.01; ***P < 0.001. **D** Viability of H1299 and A549 cells was analyzed by CCK8 24 h after treatment with the ERK inhibitor PD98059 and different concentrations of cisplatin or docetaxel. **E** γ-H2AX foci formation in H1299 cells with the ERK inhibitor PD98059 was detected by Immunofluorescence 24 h after treatment with cisplatin (15 μmol/L) or docetaxel (25 μmol/L). Mean ± SD, *n* = 3. ***P < 0.001.

Given the GSEA prediction that PHF23 is associated with the ERK signaling pathway (Fig. 4A), we investigated whether PHF23 is involved in the activation of the ERK signaling pathway in NSCLC cells. The ERK signaling pathway plays a crucial role in various biological processes, including cell metabolism, cell proliferation, and cell growth [19–21]. Using western blot assays, we found that p-ERK-1/2 (T202/Y204) levels were upregulated after PHF23 overexpression, whereas some downstream molecules of the ERK signaling pathway, such as c-myc, p38 and p-Jun, were downregulated (Supplementary Fig. 4A). The opposite result was observed after PHF23 knockdown (Supplementary Fig. 4A), further confirming the GSEA results. To verify whether PHF23 regulates the biological functions of NSCLC cells through the ERK signaling pathway, we used PD98059, an inhibitor of the ERK1/2 signaling pathway. The activation of the ERK signaling pathway by PHF23 overexpression was inhibited by the addition of PD98059 (Fig. 4B). This conclusion was further confirmed by CCK8, colony formation, EdU, and Transwell migration assays. The promotion of cell proliferation and migratory capacity by PHF23 was also inhibited by the addition of PD98059 (Fig. 4C and Supplementary Fig. 4B–F), as was DDR (Fig. 4E). The above results suggest that PHF23 promotes proliferation, migration, DDR, and chemoresistance of lung cancer cells through activation of the ERK signaling pathway.

### ACTN4 is a new interaction protein of PHF23

**Fig. 5:**
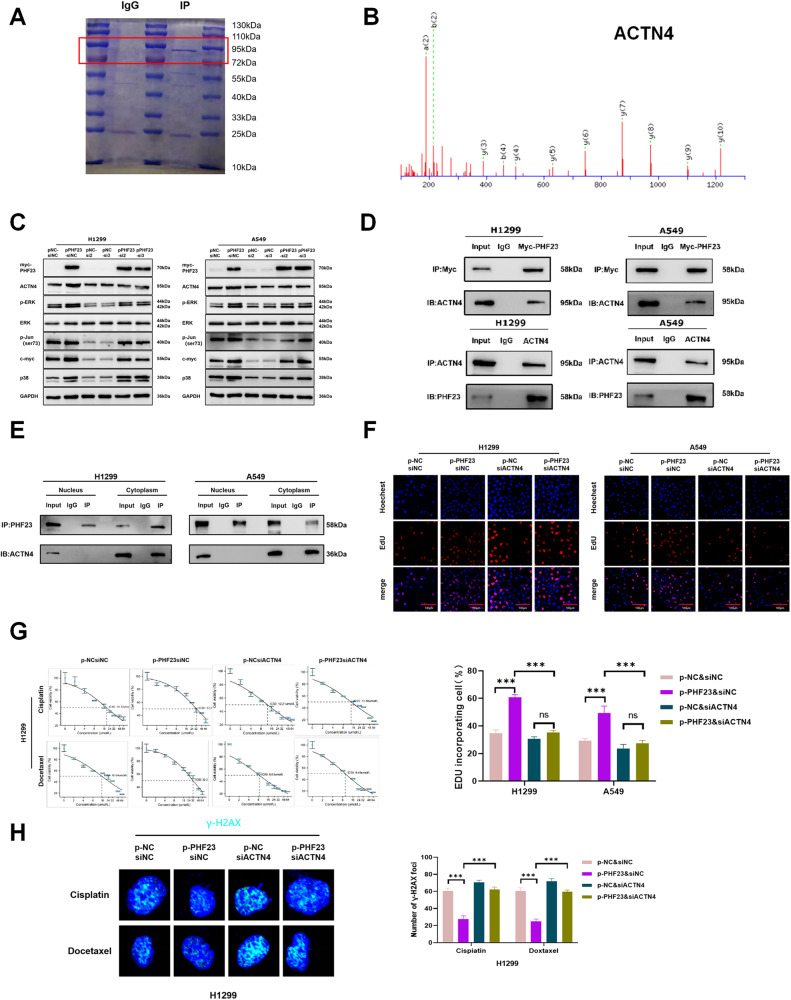
ACTN4 is the target protein of PHF23. **A** The coomassie brilliant blue staining of 10% SDS-PAGE gel. **B** Mass spectrometry detection showing the spectrogram of ACTN4 in H1299 cells pulled down by the PHF23 antibody. **C** Expression of proteins involved in the ERK signaling in A549 and H1299 cells transfected with PHF23 cDNA or siACTN4. **D** Interactions between PHF23 and ACTN4 in A549 and H1299 cells measured by co-immunoprecipitation. **E** Co-immunoprecipitation showing the interactions between PHF23 and ACTN4 in A549 and H1299 cells with the nucleoplasm separation experiment. **F** DNA replication of H1299 and A549 cells was determined by EDU staining with PHF23 cDNA or the siACTN4. Scale bar: 200 μm. Mean ± SD, *n* = 3. *P < 0.05; *P < 0.01; ***P < 0.001. **G** Viability of H1299 cells was analyzed by CCK8 24 h after transfection with PHF23 cDNA or siACTN4 and treatment of different concentrations of cisplatin or docetaxel. **H** γ-H2AX foci formation in H1299 cells transfected with PHF23 cDNA or siACTN4 was detected by Immunofluorescence 24 h after treatment with cisplatin (15 μmol/L) or docetaxel (25 μmol/L) . Mean ± SD, *n* = 3. ***P < 0.001.

To identify the potential binding partners of PHF23 involved in ERK signaling regulation, we conducted protein mass spectrometry experiments and identified ACTN4 as a candidate protein interacting with PHF23 (Fig. 5A, B). Previous studies have shown that ACTN4 is involved in the ERK signaling pathway in cancer cells [15]. We confirmed that the downregulation of p-ERK was mediated by siACTN4 in two lung cancer cell lines, further supporting the role of ACTN4 in ERK signaling regulation (Supplementary Fig. 5A). We found that ACTN4 mRNA levels were unchanged, while protein levels were upregulated after overexpression of PHF23. There was no effect on PHF23 expression after the knockdown of ACTN4 (Fig. 5C and Supplementary Fig. 5B). Furthermore, protein IP assays demonstrated that PHF23 interacts with ACTN4 in the cell plasma, suggesting that PHF23 in the cytoplasm interacts with ACTN4 to activate the ERK signaling pathway and promote the malignant phenotype of NSCLC cells (Fig. 5D, E). In addition, knockdown of ACTN4 in H1299 and A549 cells abrogated PHF23 overexpression-induced cell proliferation, migration, chemoresistance, and DDR (Fig. 5F–H and Supplementary Fig. 5C–G). In summary, our results suggest that ACTN4 is an interaction protein of PHF23 involved in the activation of the ERK signaling pathway and promote the malignant phenotype of NSCLC cells.

### PHF23 interacts with ACTN4 through the PHD

**Fig. 6:**
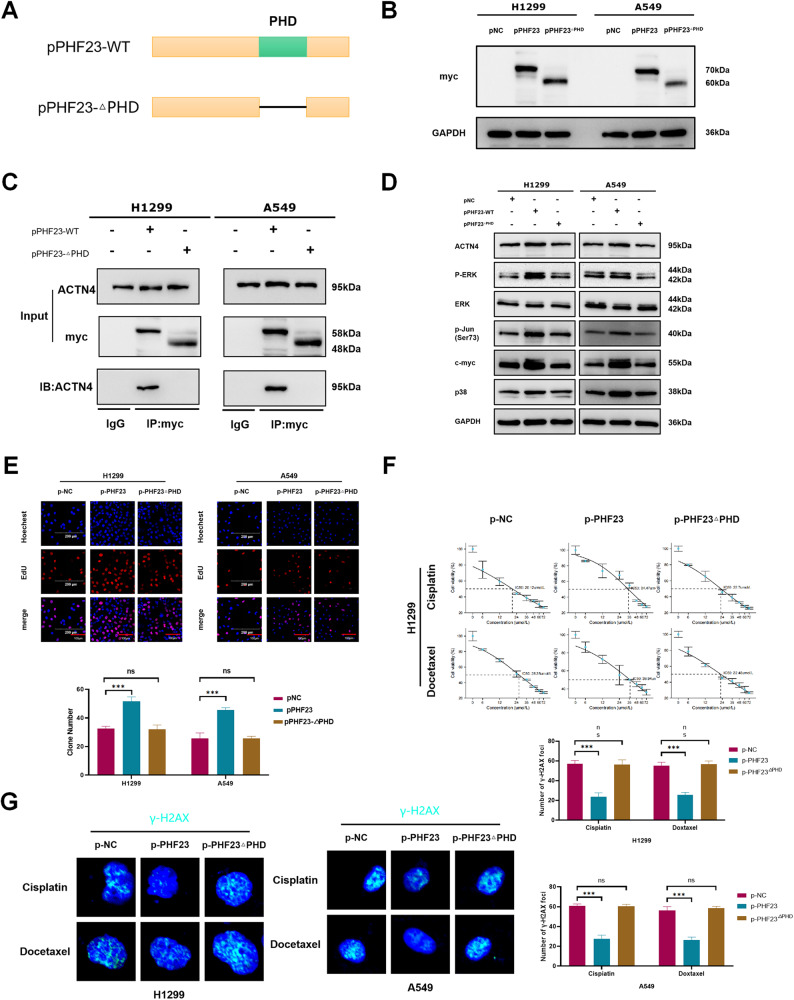
PHF23 binds to ACTN4 through the PHD structural domain. **A** Schematic diagram of PHF23 splicing mutants. **B** The expression of myc-tag after transfected with PHF23 or PHF23^△PHD^ cDNA. **C** Interactions between PHF23 and ACTN4 or PHF23^△PHD^ and ACTN4 in A549 and H1299 cells measured by co-immunoprecipitation. **D** Expression of proteins involved in the ERK signaling in A549 and H1299 cells transfected with PHF23 cDNA or PHF23^△PHD^ cDNA. **E** DNA replication of H1299 and A549 cells was determined by EDU staining with PHF23 cDNA or the PHF23^△PHD^ cDNA. Scale bar: 200 μm. Mean ± SD, *n* = 3. *P < 0.05; **P < 0.01; ***P < 0.001. **F** The viability of H1299 cells was analyzed by CCK8 24 h after transfection with PHF23 cDNA or PHF23^△PHD^ cDNA and treatment of different concentrations of cisplatin or docetaxel. **G** γ-H2AX foci formation in H1299 and A549 cells transfection with PHF23 cDNA or PHF23^△PHD^ cDNA was detected by immunofluorescence 24 h after treatment with cisplatin (15 μmol/L) or docetaxel (25 μmol/L). Mean ± SD, *n* = 3. ***P < 0.001.

Bioinformatics analysis revealed that PHF23 contains a PHD-like zinc finger structural domain at the C-terminus, which is required for the function of PHD-like zinc finger proteins. Previous studies have shown that PHF23 interacts with other proteins through the PHD and is involved in tumorigenesis [6]. We hypothesized that PHF23 in lung cancer cells functions through the PHD in cell biological processes. To test this hypothesis, we constructed the PHF23-ΔPHD plasmid (Fig. 6A, B). Deletion of the PHD from PHF23 abolished the interaction between PHF23 and ACTN4 as revealed by IP experiments in H1299 and A549 cells (Fig. 6C). In addition, knockdown of the PHD significantly inhibited activation of the ERK signaling pathway and the promotion of biological behaviors of lung cancer cells, including proliferation, migration, chemoresistance and DDR (Fig. 6E–G and Supplementary Fig. 6). These results suggest that PHF23 binds to ACTN4 in a manner dependent on the PHD.

### PHF23 is involved in ACTN4 stabilization by inhibiting its ubiquitination level

**Fig. 7:**
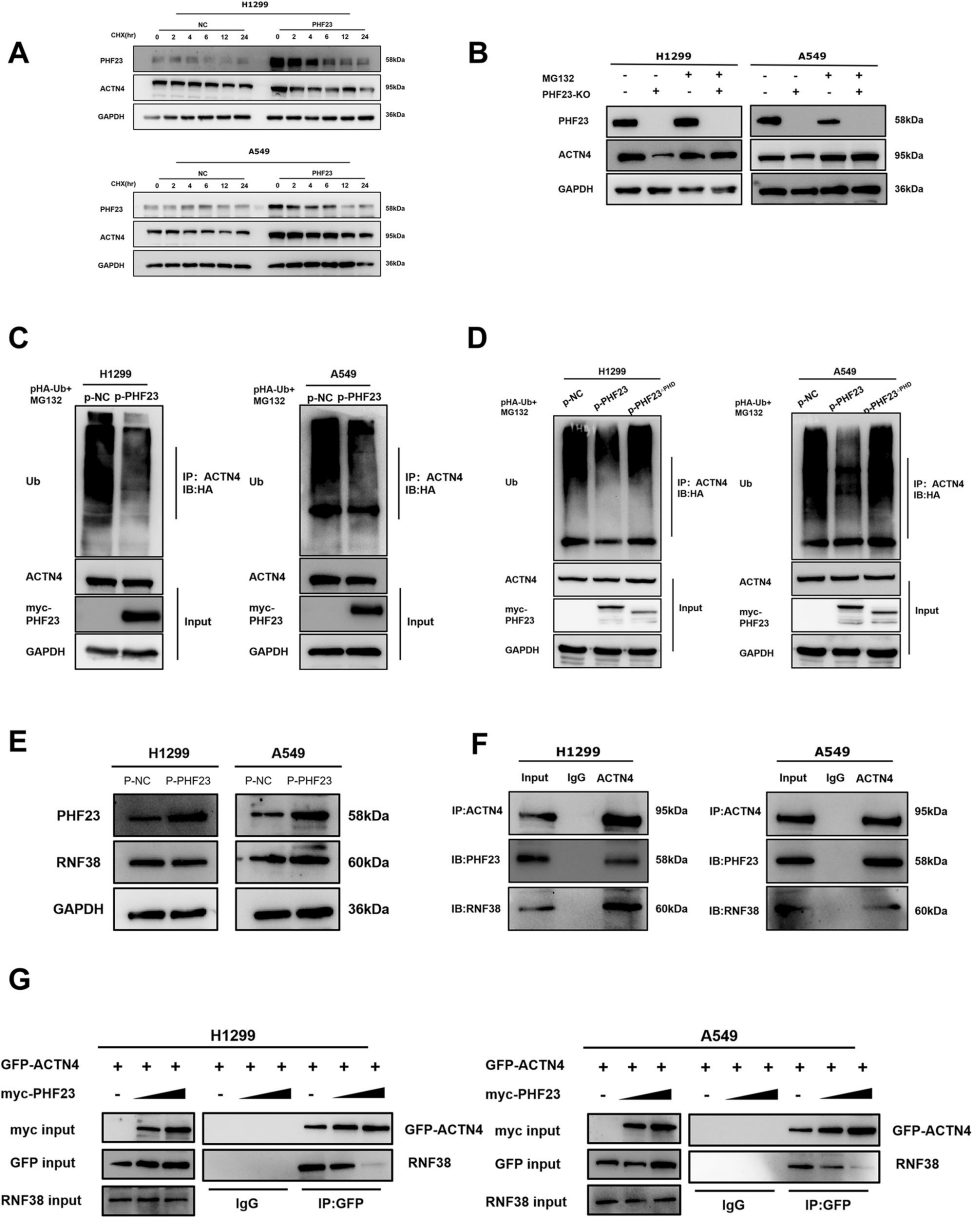
PHF23 promotes the stability of ACTN4 by inhibiting its ubiquitination level. **A** The level of PHF23 and ACTN4 proteins in H1299 and A549 cells treated with increasing concentration of cycloheximide (CHX) by western blot. **B** The level of ACTN4 protein in H1299 and A549 treated with MG132 by western blot. **C** Levels of ACTN4 ubiquitination were evaluated by immunoprecipitation using an anti-ACTN4 antibody, followed by anti-HA immunoblotting. **D** PHF23^△PHD^ reversed PHF23 downregulation of ACTN4 ubiquitination. **E** The level of RNF38 protein in PHF23 overexpressed H1299 and A549 by western blot. **F** The level of RNF38 mRNA in PHF23 overexpressed H1299 by RT-PCR. **G** Interactions between ACTN4 and RNF38 and PHF23 in A549 and H1299 cells measured by co-immunoprecipitation.

Based on the results of the previous experiments, we hypothesized that PHF23 plays a role in regulating the post-translational modifications of ACNT4, and we decided to investigate the specific mechanism by which PHF23 regulates ACTN4. We blocked protein synthesis using cycloheximide and investigated the effect of PHF23 overexpression on ACTN4 stability. We found that degradation of ACTN4 was inhibited by the addition of MG132, a potent reversible proteasome inhibitor, in H1299 and A549 cells overexpressing PHF23 (Fig. 7A). Furthermore, the addition of MG132 reversed the inhibition of ACTN4 caused by knockdown of PHF23 (Fig. 7B). We then performed IP experiments using anti-ACTN4 antibodies and assessed the level of ACTN4 ubiquitination using anti-hemagglutinin immunoblotting. The results showed that PHF23 overexpression inhibited ACTN4 ubiquitination and degradation (Fig. 7C). However, deletion of the PHD in PHF23 eliminated the inhibitory effect of PHF23 on ACTN4 ubiquitination (Fig. 7D). The results suggest that PHF23 enhances the stability of ACTN4 by inhibiting its ubiquitination through the interaction on PHD.

### PHF23 promotes ACTN4 stabilization by competitively inhibiting the interaction between RNF38 and ACTN4

Through previous literature, it is known that RING finger protein 38 (RNF38) ubiquitinates ACTN4 in nasopharyngeal carcinoma, leading to the degradation of ACTN4 and inhibiting tumor proliferation and metastasis [15]. RNF38, a member of the RNF protein family, is considered to be important for cancer progression in various tumors including cervical cancer [22], hepatocellular carcinoma [23], and NSCLC [24]. In this study, we aimed to investigate whether PHF23 competes with RNF38 for its ability to ubiquitinate ACTN4 in NSCLC. First, we overexpressed PHF23 in H1299 and A549 cells and found that RNF38 was unaltered at both mRNA and protein levels (Fig. 7E). In addition, we confirmed the interaction between endogenous ACTN4 and RNF38 in NSCLC cells using Co-IP (Fig. 7F). Then, quantitative IP experiments showed that the binding of ACTN4 and RNF38 was progressively reduced in a dose-dependent manner upon PHF23 overexpression (Fig. 7G). These results demonstrated that PHF23 competitively inhibits the interaction between RNF38 and ACTN4, thereby promoting the stabilization of ACTN4 protein and preventing its ubiquitination by RNF38.

### PHF23 promotes the malignant phenotype of lung cancer in vivo

**Fig. 8:**
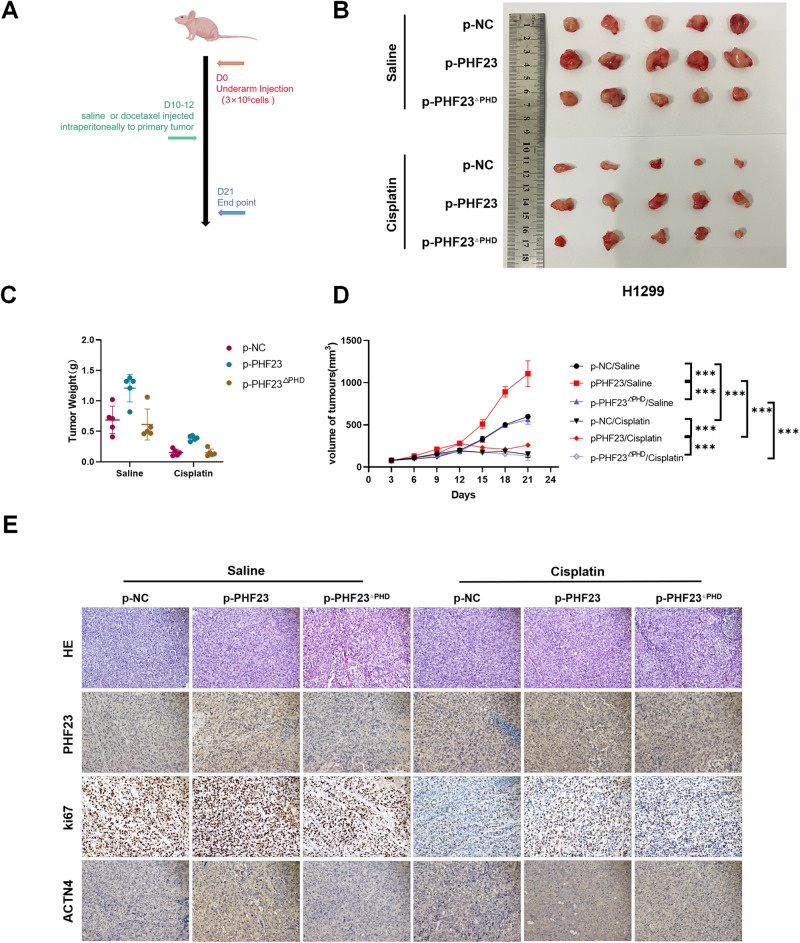
PHF23 promotes malignant phenotype of lung cancer in vivo. **A** Representative explanted tumor growth curve of mice treated as indicated (*n* = 5 per group). **B** Xenograft tumors from H1299 cells. **C** Quantification of xenograft tumor weights. **D** Tumor growth curves were shown. Error bars represent the mean ± SD. *P < 0.05, two-tailed, unpaired *t* test. Mean ± SD, *n* = 5. ***P < 0.001. **E** Representative pictures of H&E staining and IHC staining of PHF23, Ki-67, and ACTN4 in the indicated xenograft tumors. Scale bar, 50 μm.

We further investigated the biological role of PHF23 in vivo, using the H1299 cell-derived xenograft tumor model for validation (Fig. 8A). Overexpression of PHF23 resulted in a significant increase in both the volume and weight of xenograft tumors in vivo, whereas the PHF23-mediated promotional effect on tumor growth was abolished in the absence of the PHD. Moreover, PHF23 significantly counteracted the tumor suppressive effect of cisplatin (Fig. 8B–D). Immunohistochemical staining revealed that overexpression of PHF23 significantly promoted the expression of Ki-67 and ACTN4 (Fig. 8E and Supplementary Fig. 7A–C). Western blot assay of proteins extracted from xenograft tumors revealed that overexpression of PHF23 upregulated the expression of ACTN4 and activated the ERK signaling pathway and its downstream molecules in vivo (Supplementary Fig. 7D). Therefore, PHF23 can promote tumor cell proliferation and chemoresistance through activation of the ERK signaling pathway in vivo.

## Discussion

NSCLC is the most common histopathological type of lung cancer, responsible for a high number of cancer-related death worldwide [4]. Resistance to cisplatin-based chemotherapy is a major challenge in the treatment of NSCLC, leading to the failure of combination therapies [2, 25]. Therefore, there is a critical need to understand the mechanisms of chemoresistance in NSCLC and to develop effective therapeutic strategies to overcome drug resistance.

In this study, we revealed that PHF23 is mainly localized in the nucleus, and its expression level correlated with various clinicopathological parameters of NSCLC, including tumor size, lymph node metastasis, and TNM stages. Bioinformatic analysis and PHF23 staining in NSCLC patient specimens revealed that PHF23 is associated with a poor prognosis for NSCLC patients. We found that PHF23 significantly promotes aggressive proliferation, migration, DDR, and chemoresistance of NSCLC via the ERK signaling pathway in vitro and in vivo. We identified ACTN4 as a new interacting protein with PHF23, and demonstrated that PHF23 stabilizes ACTN4 by repressing its ubiquitination level by competing with RNF38. The PHF23/ACTN4 complex activates the ERK signaling pathway and promotes the malignant progression of NSCLC. Moreover, we verified that PHF23 interacts with ACTN4 through the PHD, which is critical for its stability.

Previous studies have reported that NUP98 fused with PHF23 to form NP23, which plays an important role in acute myeloid leukemia and is closely associated with disease onset and evolution [26, 27]. In addition, PHF23 has been frequently studied in tumors. It was found to inhibit autophagy in osteosarcoma U2OS cells and HeLa cells [6], while been highly expressed in cartilage and synovium in human osteoarthritis, inhibiting cellular autophagy [7]. PHF23 deficiency also impairs B-cell differentiation and promotes immature B-lymphocyte malignancies [28]. Mechanistically, PHF23 binds directly to the SIN3-HDAC complex through the N-terminus and inhibits its deacetylation activity on H3K27AC [29]. However, the role of PHF23 in NSCLC remains unclear. In this study, we demonstrate that PHF23 acts as a pro-oncogene, promoting proliferation, migration and drug resistance in lung cancer.

The ERK signaling pathway is one of the most common oncogenic signaling mechanisms in cancer cells and has been confirmed to be involved in multiple biological processes of tumor cells, including proliferation, migration, invasion and drug resistance [30]. In our experiments, we used bioinformatics analysis to predict that PHF23 was positively correlated with the ERK signaling pathway. Then, we experimentally verified that PHF23 activates the ERK signaling pathway by stabilizing ACTN4, thereby promoting proliferation, migration, and chemoresistance of lung cancer cells in vitro and in vivo. Interestingly, persistent activation of the ERK signaling pathway is a molecular mechanism of EGFR-TKI resistance in NSCLC, and many receptors have been found to induce acquired drug resistance through persistent activation of ERK signaling [31–34]. Moreover, the combination of chemotherapeutic drugs with EGFR-TKI is commonly used in clinical practice. Therefore, it is worth exploring whether PHF23 can be used as a target to alleviate chemoresistance in clinical practice and whether PHF23 affects the efficacy and survival of patients treated with the combination of chemotherapeutic drugs and EGFR-TKI in further studies.

In our study, we identified ACTN4 as a leading candidate for the protein interacting with PHF23 through mass spectrometry analysis. Using Co-IP experiments, we verified for the first time, to the best of our knowledge, that PHF23 interacts with ACTN4. Therefore, we further investigated ACTN4 as a target protein of PHF23. ACTN4 is an actin-binding protein that is normally expressed in non-muscle cells and is involved in regulation of cytoskeletal integrity and cell motility as well as the development of various tumors. It plays a role in promoting tumor proliferation, migration, invasion, and chemoresistance in a variety of tumors including osteosarcoma, breast cancer, and melanoma [9–11, 35]. In studies related to lung adenocarcinoma, high expression of ACTN4 has been identified as a marker of platinum-based treatment outcomes in patients [12]. Kriger et al. noted that in NSCLC cells, ACTN4 selectively affects the repair of DNA double-strand breaks [13]. Consistently, our experiments suggested that the knockdown of ACTN4 partially abrogates the role of PHF23 in promoting chemoresistance in lung cancer. However, further exploration is needed to determine whether ACTN4 is a downstream target protein of PHF23 in other diseases and tumors.

Next, we investigated the specific mechanism underlying the interaction between PHF23 and ACTN4. First, through RT-PCR and western blot experiments, we found that PHF23 regulates ACTN4 at the translational level rather than at the transcriptional level. Moreover, in PHF23 knockdown cells, the addition of MG132 inhibited ACTN4 degradation. Intriguingly, we demonstrated that PHF23 inhibits the ubiquitinated degradation of ACTN4 for the first time, thereby enhancing the protein stability of ACTN4. However, little research has been reported on the mechanism by which PHF23 participates in the post-translational modifications of proteins. In U2OS and HeLa cells, it has been reported that PHF23 binds to LRSAM1 through the PHD and promotes its ubiquitinated degradation, thereby promoting cellular autophagy [6]. This report thus raises a question: Does the binding of PHF23 to ACTN4 dependent on the PHD?

The PHD is a structural pattern common to all eukaryotic genomes [36, 37]. Previous studies have shown that the PHD as a bridge between protein and histone binding, regulating gene expression and modifications [38, 39]. In addition, the PHD may act as a widely distributed subfamily of zinc fingers involved in the regulation of gene transcription [40]. Therefore, we investigated whether, in NSCLC, PHF23 functions depends on the PHD. We constructed a mutant plasmid with knockout of the PHD in PHF23 and found that PHF23 binds to ACTN4 via the PHD and inhibits the ubiquitination level of ACTN4, thereby promoting malignant biological behaviors of lung cancer cells. These results fill a gap in our understanding of the involvement of PHF23 in the protein regulatory functions of lung cancer.

Platinum-based therapy is the major chemotherapy approach for NSCLC and is frequently combined with other agents, such as docetaxel, in clinical practice [41, 42]. Although chemotherapy has significantly enhanced the prognosis of NSCLC patients, chemoresistance remains a major impediment to effective treatment [43]. In our study, we identified PHF23 as a potential therapeutic target for lung cancer chemotherapy. Targeted agents also play a crucial role in NSCLC treatment. EGFR-TKIs are currently a widely used targeted therapy in the clinical treatment of lung cancer, which can effectively improve the prognosis of NSCLC patients with EGFR mutations. Nevertheless, multiple-driver gene mutations are the primary cause of drug resistance in patients and present a significant challenge for NSCLC management [44–46]. Fu et al. reported in bacterial meningitis that EGFR competitively recruits ACTN4 from F-actin, facilitating the invasion of E. coli meningitidis into brain microvascular endothelial cells [47]. Hence, exploring whether the effect of PHF23 on ACTN4 protein stability is implicated in the resistance process to EGFR-TKIs could provide a new target for combating drug resistance in lung cancer.

**Fig. 9:**
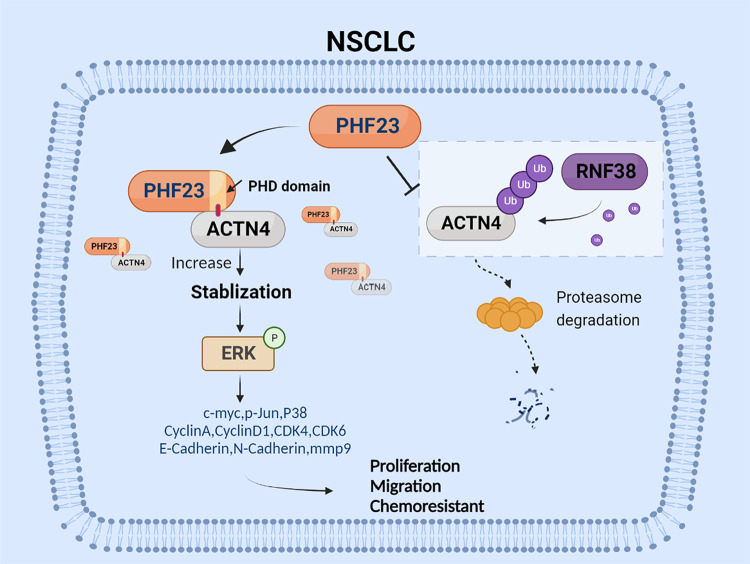
A schematic figure depicting the proposed mechanism of PHF23 in NSCLC. PHF23 promotes proliferation, migration, and chemoresistance by regulating ERK signaling pathway. Modulation of ERK phosphorylation activity by PHF23 is dependent on preventing ACTN4 from being ubiquitinated by RNF38.

In summary, identifying the PHF23/ACTN4/ERK axis provides a complementary and comprehensive understanding of NSCLC and presents an opportunity to translate fundamental research into potential treatments in clinical practice (Fig. 9).

## Conclusions

We demonstrate novel biological functions of PHF23 in promoting NSCLC carcinogenesis. Our study identifies the mechanism by which the PHF23/ACTN4/ERK axis regulates malignant biological behavior and chemoresistance of NSCLC cells. These findings highlight the potential of targeting PHF23 and its associated pathways as a strategy to improve the sensitivity of chemotherapy in NSCLC.

## Supplementary information

Collated Supplementary Information File

## Data Availability

The datasets used and analyzed during the current study are available from the corresponding author on reasonable request.
