# Supplementary material for: PHF23 promotes NSCLC proliferation, metastasis, and chemoresistance via stabilization of ACTN4 and activation of the ERK pathway
Source: Cell Death Dis. 2023 Aug 25;14(8):558. doi: 10.1038/s41419-023-06069-4 (PMC10457402; doi:10.1038/s41419-023-06069-4)

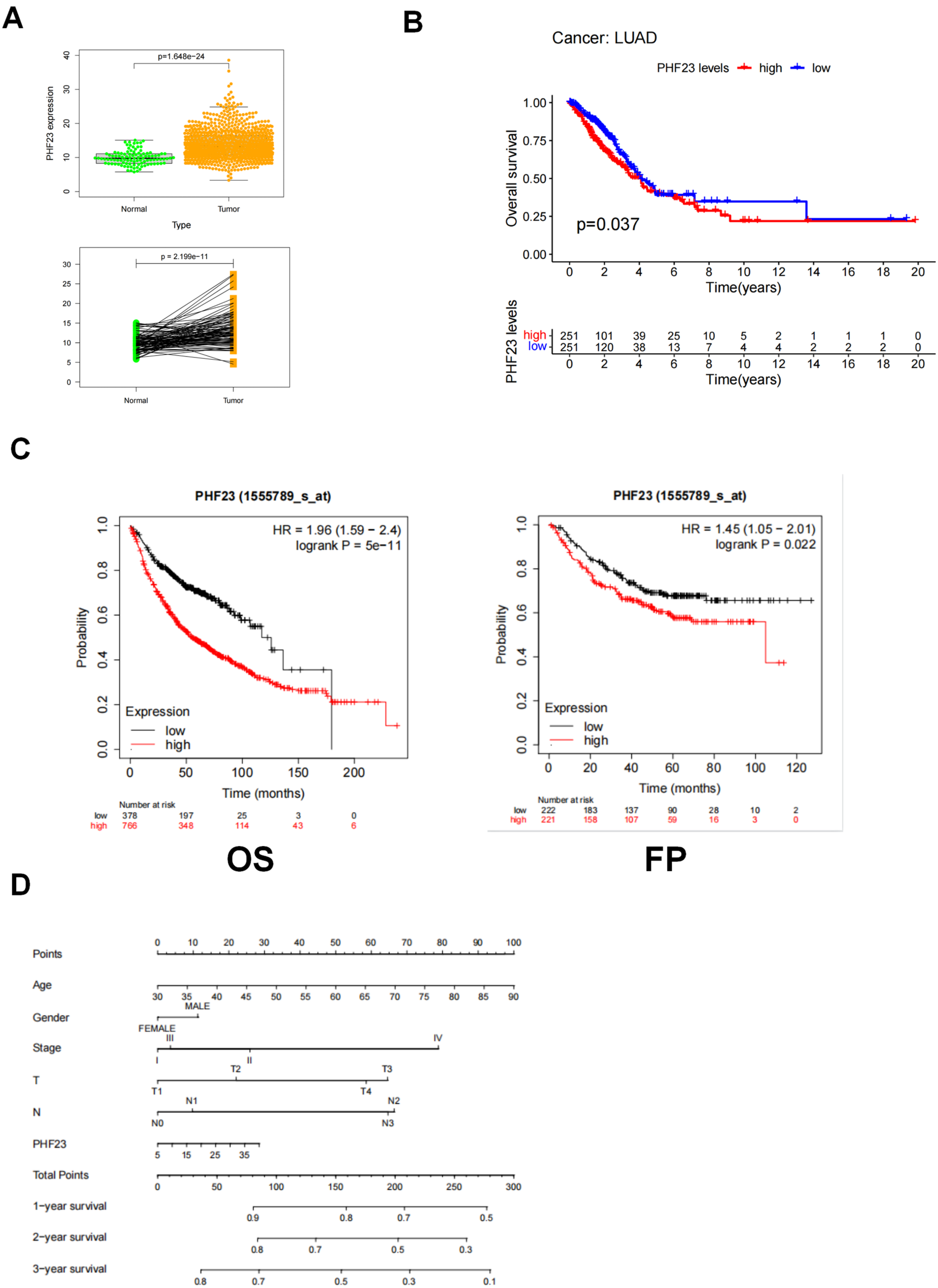

A
p=1.648e−24
PHF23 expression
Normal
Tumor
Type
p = 2.199e−11
Normal
Tumor
B
Cancer: LUAD
PHF23 levels high low
Overall survival
p=0.037
Time(years)
PHF23 levels
high 251 101 39 25 10 5 2 1 1 1 0
low 251 120 38 13 7 4 4 2 2 2 0
Time(years)
C
PHF23 (1555789_s_at)
HR = 1.96 (1.59 − 2.4)
logrank P = 5e−11
Probability
Expression
low
high
Time (months)
Number at risk
low 378 197 25 3 0
high 766 348 114 43 6
PHF23 (1555789_s_at)
HR = 1.45 (1.05 − 2.01)
logrank P = 0.022
Probability
Expression
low
high
Time (months)
Number at risk
low 222 183 137 90 28 10 2
high 221 158 107 59 16 3 0
OS
FP
D
Points
Age
Gender
MALE
FEMALE
Stage
T
N
PHF23
Total Points
1−year survival
2−year survival
3−year survival

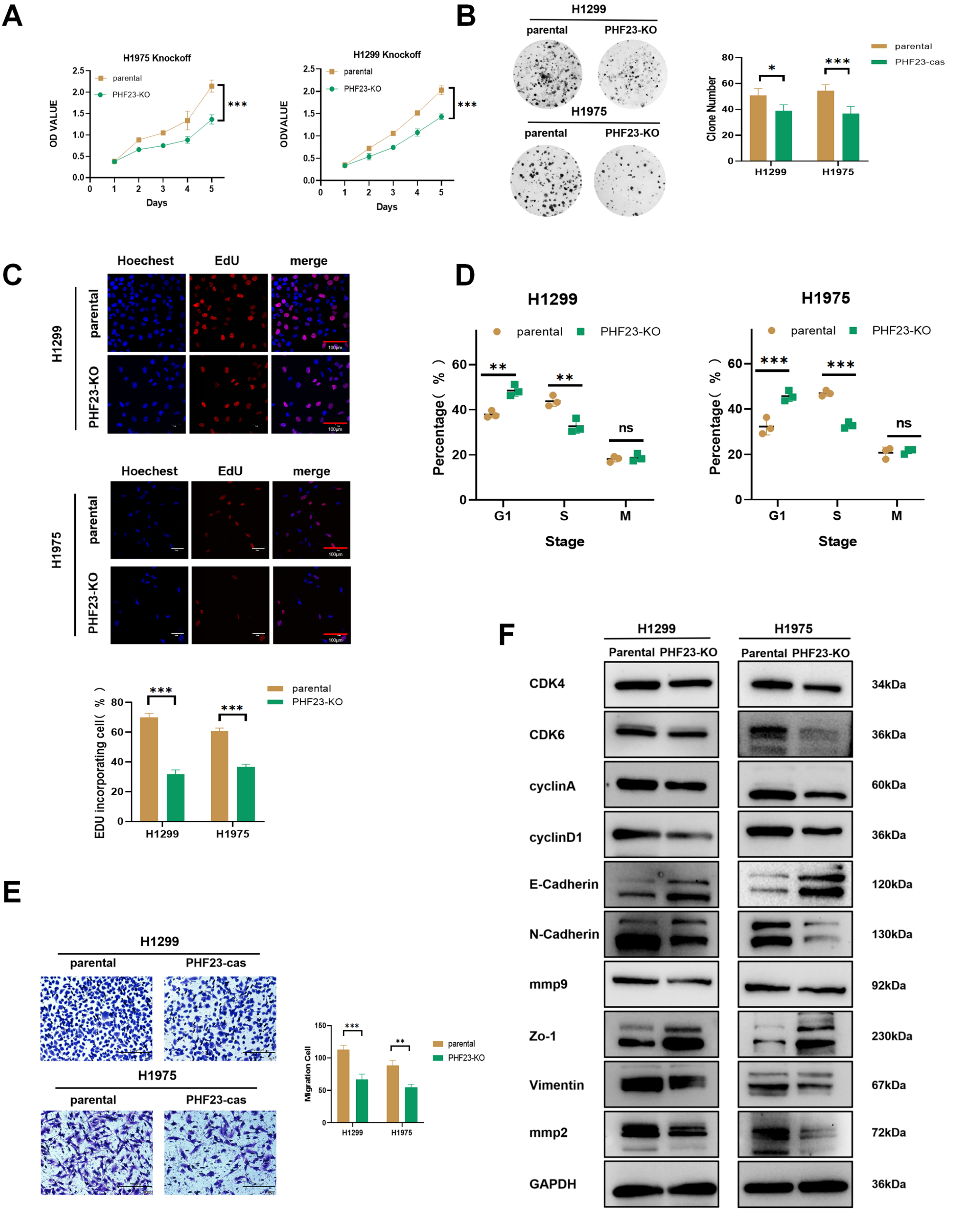
A
H1975 Knockoff
parental
PHF23-KO
OD VALUE
Days
***
H1299 Knockoff
parental
PHF23-KO
ODVALUE
Days
***
B
H1299
parental
PHF23-KO
H1975
parental
PHF23-KO
Clone Number
parental
PHF23-cas
H1299
H1975
*
***
C
Hoechest
EdU
merge
H1299
parental
PHF23-KO
Hoechest
EdU
merge
H1975
parental
PHF23-KO
EDU incorporating cell（ % ）
parental
PHF23-KO
H1299
H1975
***
***
D
H1299
parental
PHF23-KO
Percentage（ % ）
G1
S
M
Stage
**
**
ns
H1975
parental
PHF23-KO
Percentage（ % ）
G1
S
M
Stage
***
***
ns
E
H1299
parental
PHF23-cas
H1975
parental
PHF23-cas
Migration Cell
parental
PHF23-KO
H1299
H1975
***
**
F
H1299
Parental
PHF23-KO
H1975
Parental
PHF23-KO
CDK4
34kDa
CDK6
36kDa
cyclinA
60kDa
cyclinD1
36kDa
E-Cadherin
120kDa
N-Cadherin
130kDa
mmp9
92kDa
Zo-1
230kDa
Vimentin
67kDa
mmp2
72kDa
GAPDH
36kDa

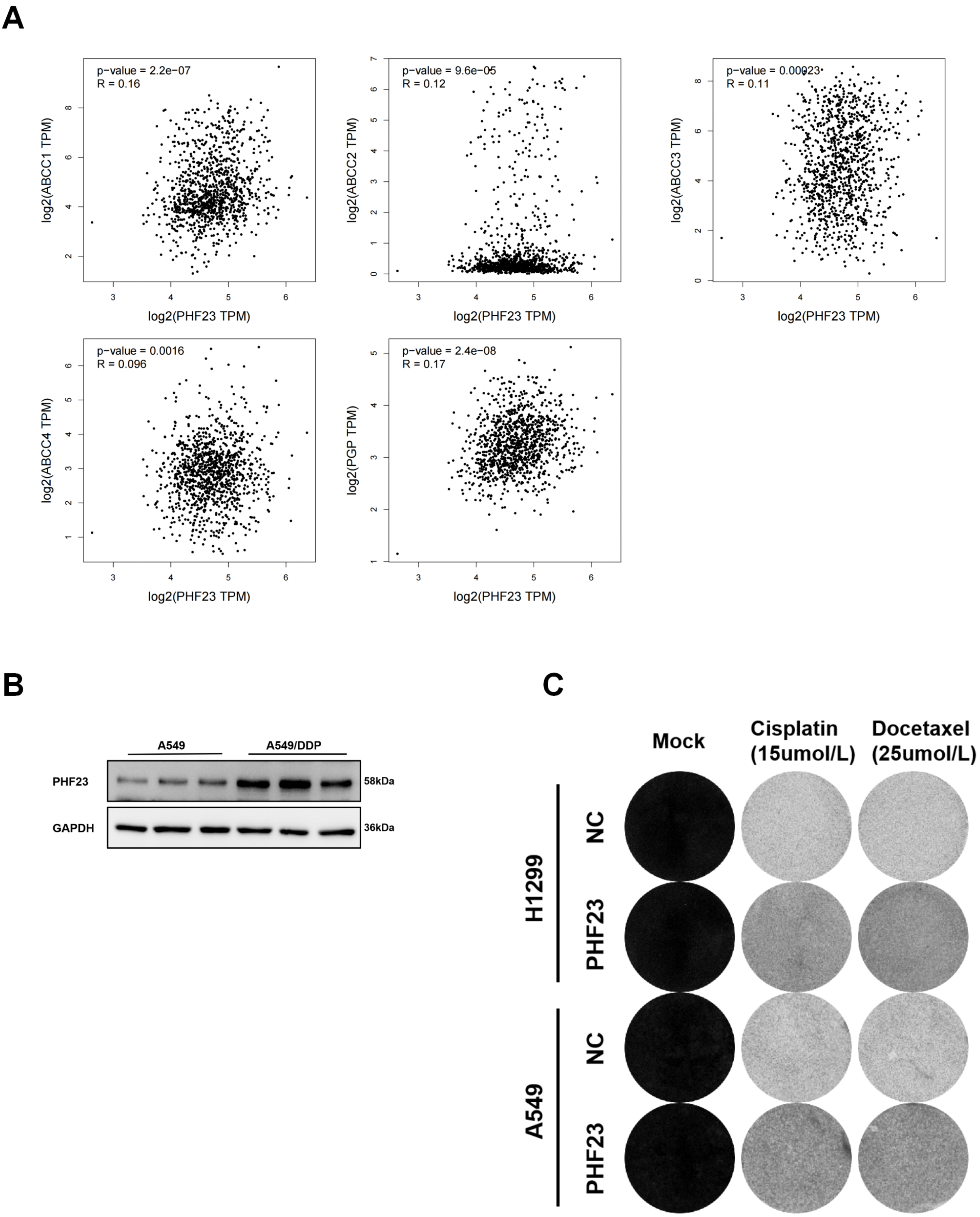
A
p−value = 2.2e−07
R = 0.16
log2(ABCC1 TPM)
log2(PHF23 TPM)
p−value = 9.6e−05
R = 0.12
log2(ABCC2 TPM)
log2(PHF23 TPM)
p−value = 0.00023
R = 0.11
log2(ABCC3 TPM)
log2(PHF23 TPM)
p−value = 0.0016
R = 0.096
log2(ABCC4 TPM)
log2(PHF23 TPM)
p−value = 2.4e−08
R = 0.17
log2(PGP TPM)
log2(PHF23 TPM)
B
A549
A549/DDP
PHF23
58kDa
GAPDH
36kDa
C
Mock
Cisplatin (15umol/L)
Docetaxel (25umol/L)
H1299
NC
PHF23
A549
NC
PHF23

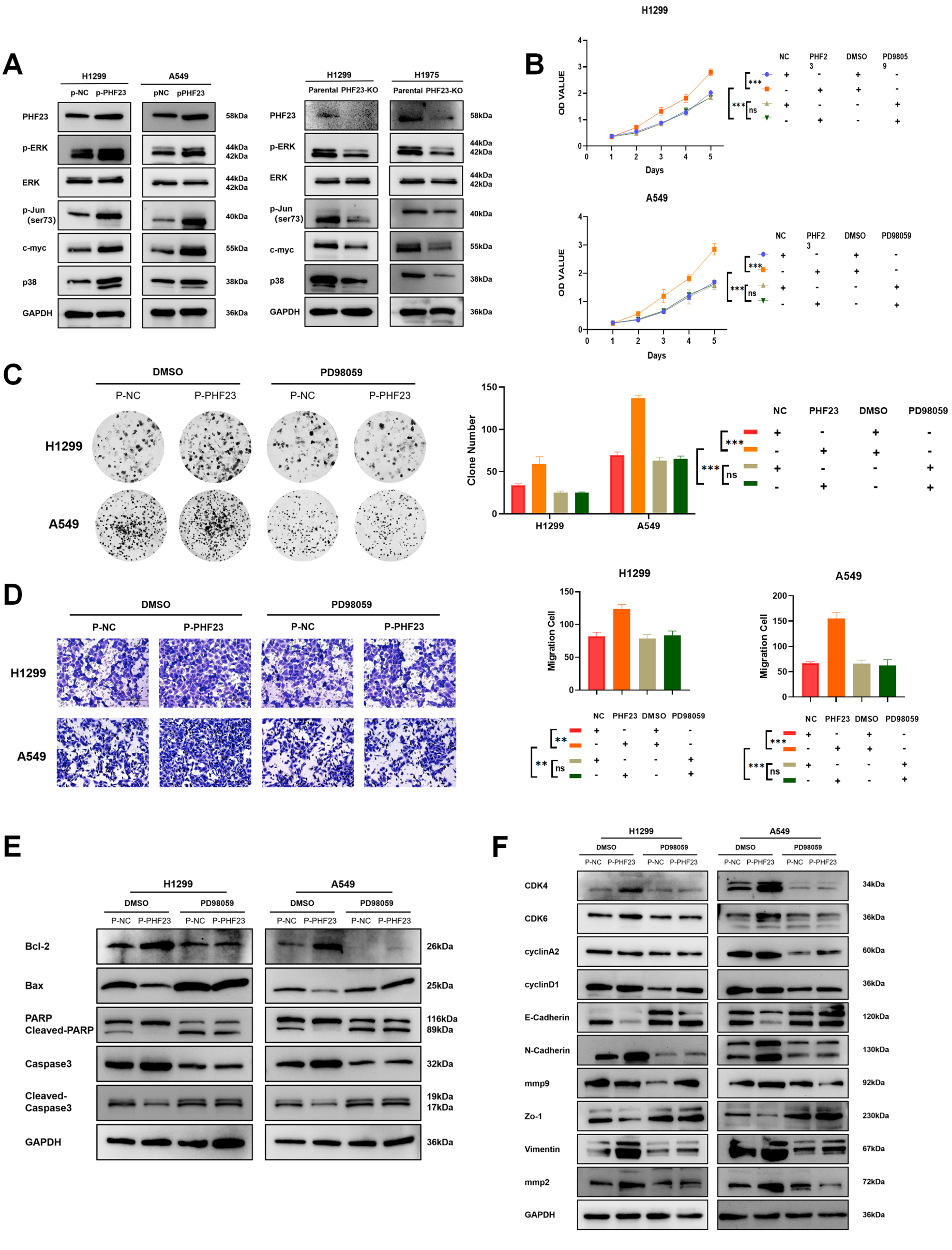

A
H1299
A549
p-NC p-PHF23
pNC pPHF23
PHF23
p-ERK
ERK
p-Jun (ser73)
c-myc
p38
GAPDH
58kDa
44kDa 42kDa
40kDa
55kDa
38kDa
36kDa
H1299
H1975
Parental PHF23-KO
B
H1299
A549
OD VALUE
Days
NC PHF23 DMSO PD98059
***
ns
C
DMSO
PD98059
P-NC P-PHF23
H1299
A549
Clone Number
D
Migration Cell
**
E
H1299
A549
DMSO PD98059
P-NC P-PHF23
Bcl-2
Bax
PARP
Cleaved-PARP
Caspase3
Cleaved-Caspase3
GAPDH
26kDa
25kDa
116kDa 89kDa
32kDa
19kDa 17kDa
36kDa
F
CDK4
CDK6
cyclinA2
cyclinD1
E-Cadherin
N-Cadherin
mmp9
Zo-1
Vimentin
mmp2
GAPDH
34kDa
36kDa
60kDa
36kDa
120kDa
130kDa
92kDa
230kDa
67kDa
72kDa
36kDa

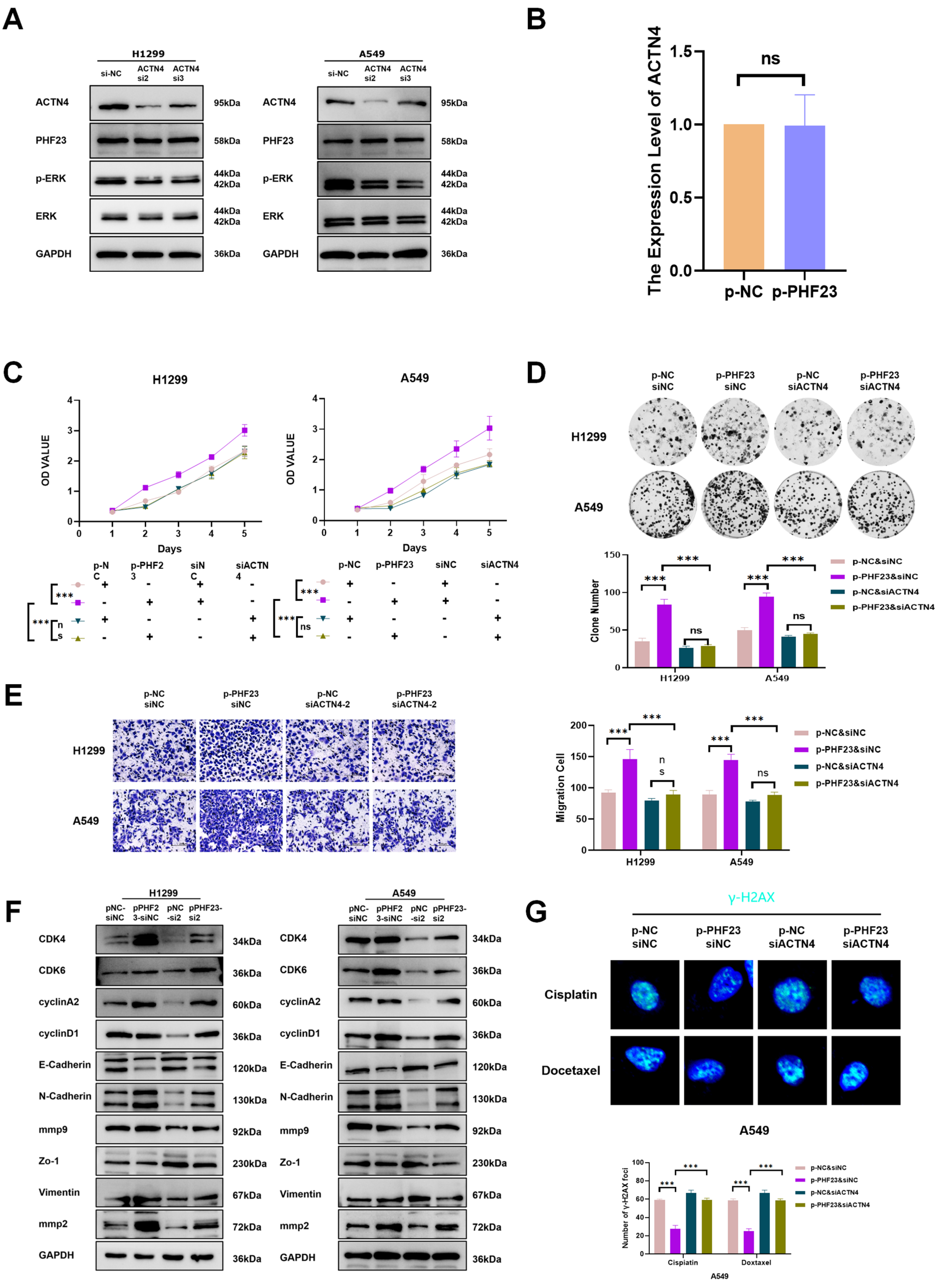

A
H1299
si-NC
ACTN4 si2
ACTN4 si3
ACTN4
PHF23
p-ERK
ERK
GAPDH
95kDa
58kDa
44kDa 42kDa
36kDa
A549
B
ns
The Expression Level of ACTN4
p-NC
p-PHF23
C
H1299
A549
OD VALUE
Days
p-NC
p-PHF23
siNC
siACTN4
***
ns
D
p-NC siNC
p-PHF23 siNC
p-NC siACTN4
p-PHF23 siACTN4
Clone Number
p-NC&siNC
p-PHF23&siNC
p-NC&siACTN4
p-PHF23&siACTN4
E
p-NC siACTN4-2
p-PHF23 siACTN4-2
Migration Cell
F
pNC-siNC
pPHF23-siNC
pNC-si2
pPHF23-si2
CDK4
CDK6
cyclinA2
cyclinD1
E-Cadherin
N-Cadherin
mmp9
Zo-1
Vimentin
mmp2
GAPDH
34kDa
36kDa
60kDa
120kDa
130kDa
92kDa
230kDa
67kDa
72kDa
G
γ-H2AX
Cisplatin
Docetaxel
Number of γ-H2AX foci
Cispiatin
Doxtaxel

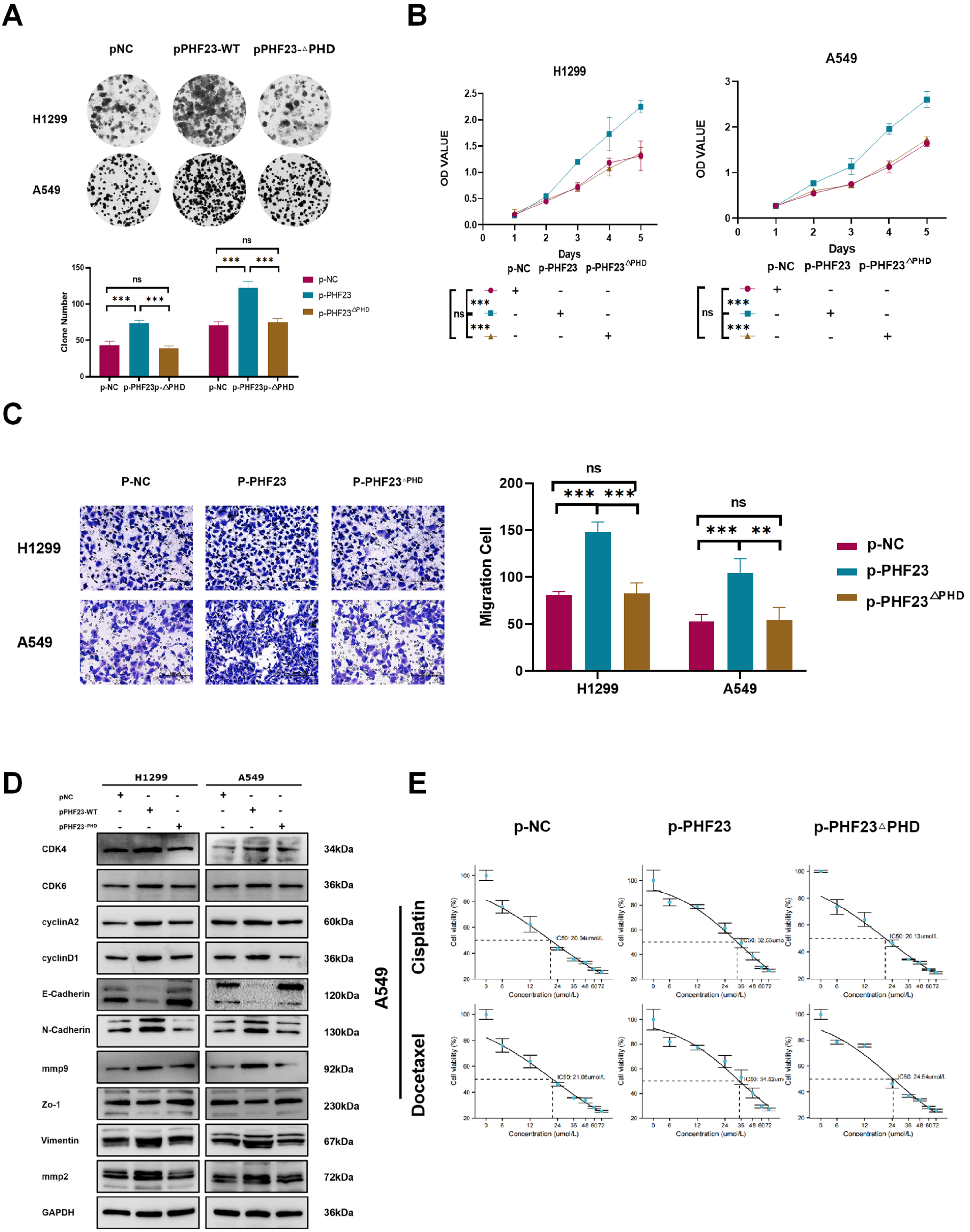
A
pNC
pPHF23-WT
pPHF23-△PHD
H1299
A549
Clone Number
ns
***
p-NC
p-PHF23
p-PHF23ΔPHD
B
H1299
A549
OD VALUE
Days
C
P-NC
P-PHF23
P-PHF23△PHD
Migration Cell
D
pNC
pPHF23-WT
CDK4
CDK6
cyclinA2
cyclinD1
E-Cadherin
N-Cadherin
mmp9
Zo-1
Vimentin
mmp2
GAPDH
34kDa
36kDa
60kDa
36kDa
120kDa
130kDa
92kDa
230kDa
67kDa
72kDa
36kDa
E
p-NC
p-PHF23
p-PHF23△PHD
A549
Cisplatin
Docetaxel
Cell viability (%)
Concentration (umol/L)
IC50: 20.04umol/L
IC50: 32.55umol
IC50: 20.13umol/L
IC50: 21.06umol/L
IC50: 34.52um
IC50: 24.64umol/L

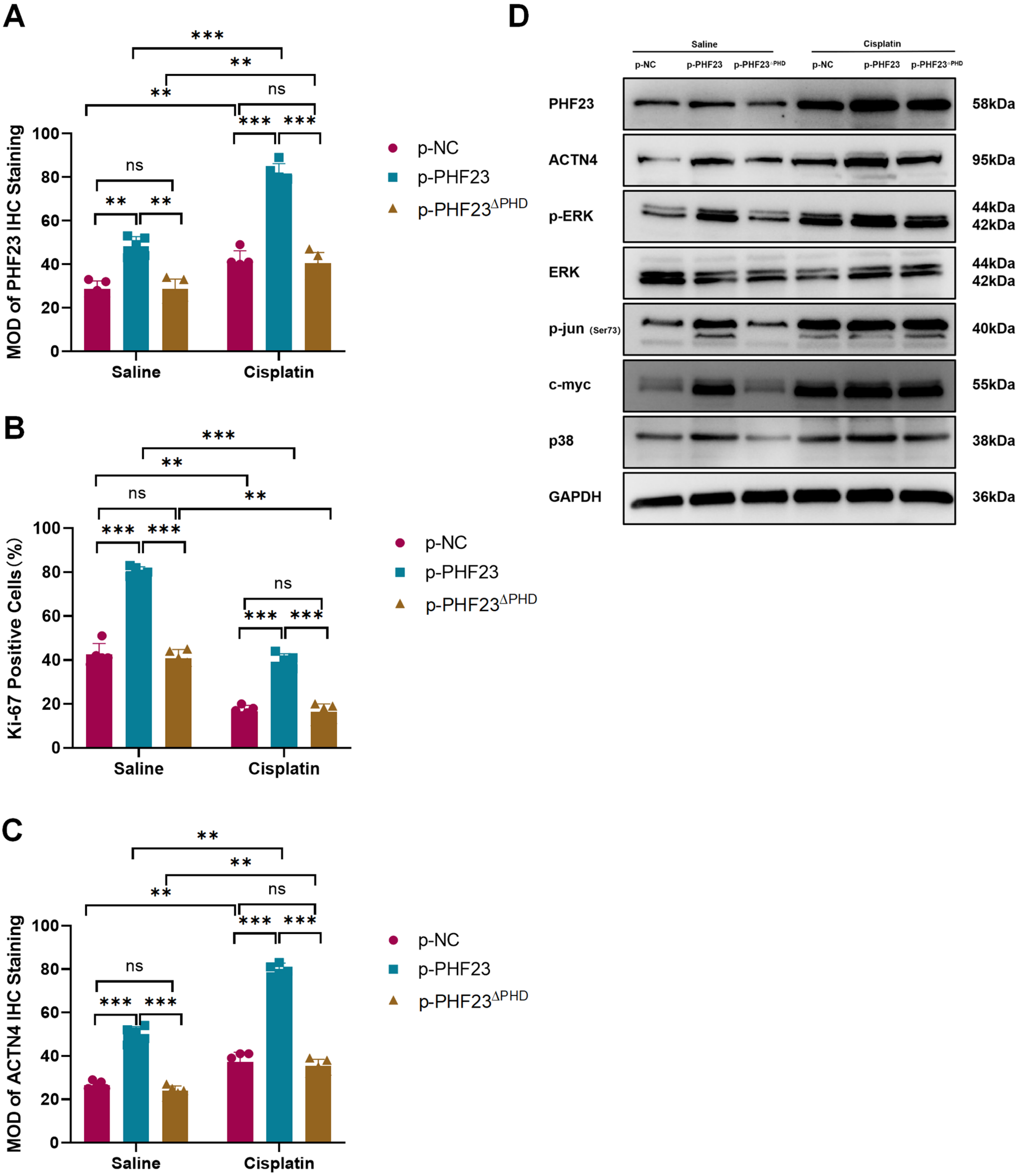

A
MOD of PHF23 IHC Staining
100
80
60
40
20
0
Saline
Cisplatin
***
**
**
ns
*** ***
ns
** **
p-NC
p-PHF23
p-PHF23ΔPHD
B
Ki-67 Positive Cells (%)
100
80
60
40
20
0
Saline
Cisplatin
***
**
ns
**
*** ***
ns
*** ***
p-NC
p-PHF23
p-PHF23ΔPHD
C
MOD of ACTN4 IHC Staining
100
80
60
40
20
0
Saline
Cisplatin
**
**
**
ns
*** ***
ns
*** ***
p-NC
p-PHF23
p-PHF23ΔPHD
D
Saline
Cisplatin
p-NC
p-PHF23
p-PHF23ΔPHD
p-NC
p-PHF23
p-PHF23ΔPHD
PHF23
58kDa
ACTN4
95kDa
p-ERK
44kDa
42kDa
ERK
44kDa
42kDa
p-jun (Ser73)
40kDa
c-myc
55kDa
p38
38kDa
GAPDH
36kDa

**Additional files**

Supplementary Figure 1.

**A.** Analysis of the expression of PHF23 mRNA in NSCLC datasets from the TCGA database.

**B.** Survival analysis of NSCLC patients with high and low PHF23 expression in lung adenocarcinoma based on TCGA data.

**C.** The association of PHF23 with OS and FP was analyzed in Kaplan Meier plotter website.

**D.** The nomogram established by identified risk factors for predicting 1- ， 2- and 3-years survival.

Supplementary Figure 2.

**A.** Cell viability was analyzed by CCK8. Mean±SD, n = 3. ***, P < 0.001.

**B.** Cell growth was determined by Colony formation. Mean±SD, n = 3. *, P < 0.05; **, P < 0.01;*** P < 0.001.

**C.** DNA replication of H1299 and A549 cells was determined by EDU staining. Scale bar: 200 μm. Mean±SD,n = 3. *, P < 0.05; *, P < 0.01;***, P < 0.001.

**D.** Cell cycle of H1299 and H1975 cells was analyzed by Flowcytometry. Mean±SD,n = 3. *, P < 0.05;**P < 0.01, ***P < 0.001.

**E.** Cell migration evaluated by the transwell migration assay; cells that migrated to the lower chamber were stained with hematoxylin and counted. **P < 0.01.

**F.** Expression of cell proliferation- and migration- related proteins in H1299 and H1975.

Supplementary Figure 3.

**A.** GEPIA database was interrogated for PHF23 and ABCC1,ABCC2,ABCC3,ABCC4 and PGP expression. Correlations were analyzed by Pearson statistics.

**B.** Western blotting analyzing the expression of PHF23 in the A549 and A549-DDP cells.

**C.** Colony formation of the indicated cells after treatment with cisplatin or docetaxel.

Supplementary Figure 4.

**A.** Expression of proteins involved in the ERK signaling in A549 and H1299 cells transfected with PHF23 cDNA.

**B.** Cell viability was analyzed by CCK8. Mean±SD, n = 3. ***P < 0.001.

**C.** Cell growth was determined by Colony formation. Mean±SD, n = 3.P < 0.05; **, P < 0.01;*** P < 0.001.

**D.** Cell migration evaluated by the transwell migration assay. Mean±SD, n = 3.**P < 0.01,*** P < 0.001.

**E.** Expression of proteins involved in cell apoptosis in A549 and H1299 cells transfected with PHF23 cDNA upon treatment with DMSO or the ERK inhibitor PD98059.

**F.** Expression of proteins involved in cell proliferation and migration in A549 and H1299 cells transfected with PHF23 cDNA upon treatment with DMSO or the ERK inhibitor PD98059.

Supplementary Figure 5.

**A.** Expression of proteins involved in the ERK signaling in H1299 and A549 cells transfected with siACTN4-2 or siACTN4-3.

**B.** The mRNA level of ACTN4 after transfection of PHF23 cDNA by RT-PCR.Mean±SD, n = 3.

**C.** Cell viability was analyzed by CCK8. Mean±SD, n = 3. P < 0.01;***P < 0.001.

**D.** Cell growth was determined by Colony formation. Mean±SD, n = 3. *, P < 0.05; **, P < 0.01;*** P < 0.001.

**E.** Cell migration evaluated by the transwell migration assay. Mean±SD, n = 3. *** P < 0.001.

**F.** Expression of proteins involved in cell proliferation and migration in A549 and H1299 cells transfected with PHF23 cDNA or siACTN4.

**G.** γ-H2AX foci formation in A549 cells transfected with PHF23 cDNA or siACTN4 was detected by Immunofluorescence 24 h after treatment with cisplatin(15μmol/L) or docetaxel(25μmol/L) . Mean±SD, n = 3. ***P < 0.001.

Supplementary Figure 6.

**A.** Cell growth was determined by Colony formation. Mean±SD, n = 3.*** P < 0.001.

**B.** Cell viability was analyzed by CCK8. Meanation. Mean±SD, n = 3. *** P < 0.001.

**C.** Cell migration evaluated by the transwell migration assay. Mean±SD, n = 3.**P < 0.01, *** P < 0.001.

**D.** Expression of proteins involved in cell proliferation and migration in A549 and H1299 cells transfection with PHF23 cDNA or $PHF23^{\triangle PHD}$ cDNA.

**E.** Viability of A549 cells was analyzed by CCK8 24 h after transfection with PHF23 cDNA or $PHF23^{\triangle PHD}$ cDNA and treatment of different concentrations of cisplatin or docetaxel.

Supplementary Figure 7.

**A.** Comparison of IHC staining for PHF23 of tumors in the nude mouse model. The grayscale PHF23 signal is the mean optical density (MOD) of staining. Mean±SD, $n = 3$. **, $P < 0.01$;*** $P < 0.001$.

**B.** Percentage of Ki-67-positive cells. Mean±SD, $n = 3$. **, $P < 0.01$;*** $P < 0.001$.

**C.** Comparison of IHC staining for ACTN4 of tumors in the nude mouse model. The grayscale PHF23 signal is the mean optical density (MOD) of staining. Mean±SD, $n = 3$. **, $P < 0.01$;*** $P < 0.001$.

**D.** Expression of PHF23, ACTN4 and proteins involved in the ERK signaling in xenograft tumors was detected by Western Blot.

Figure1B

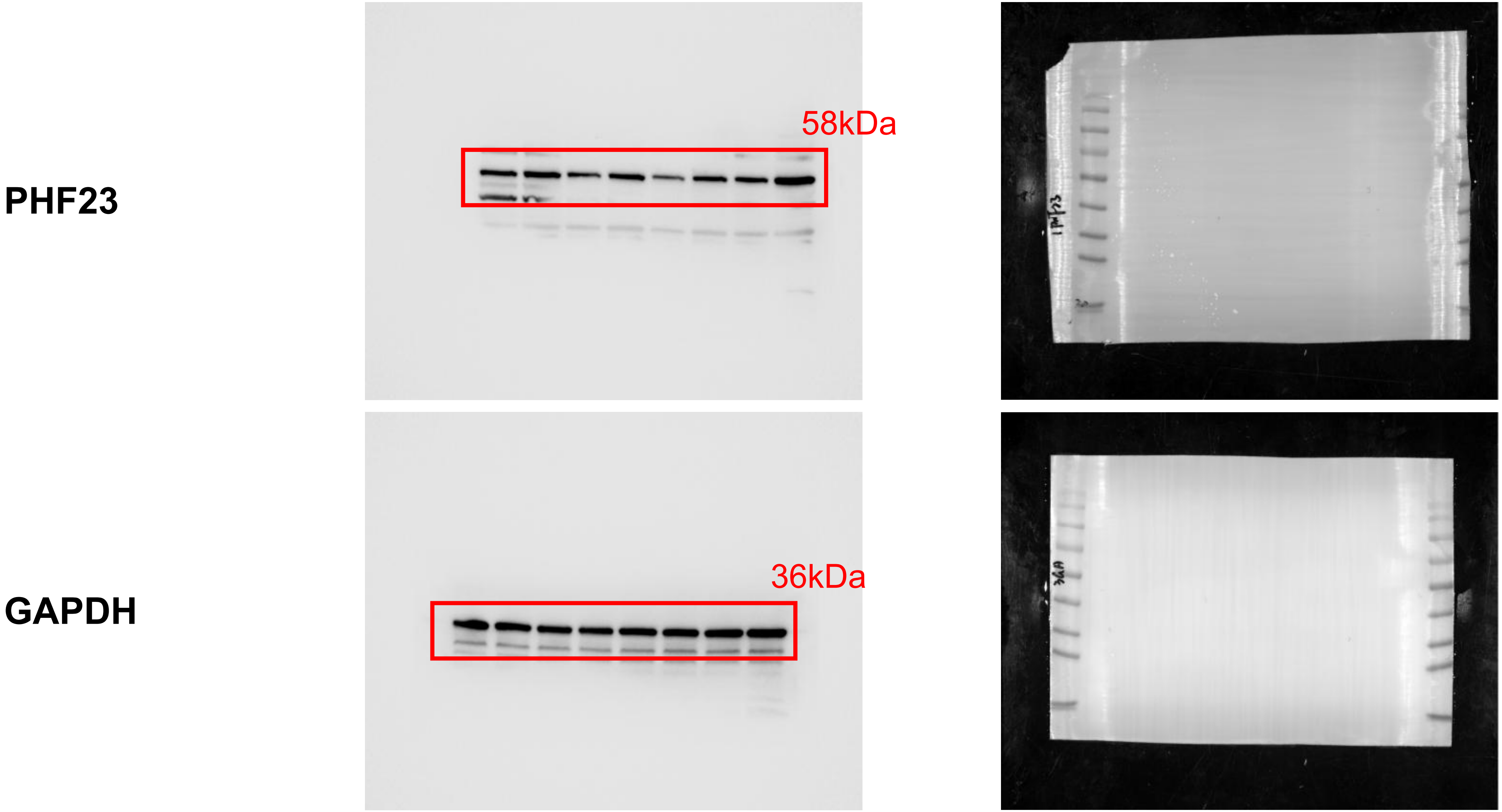

Figure1B

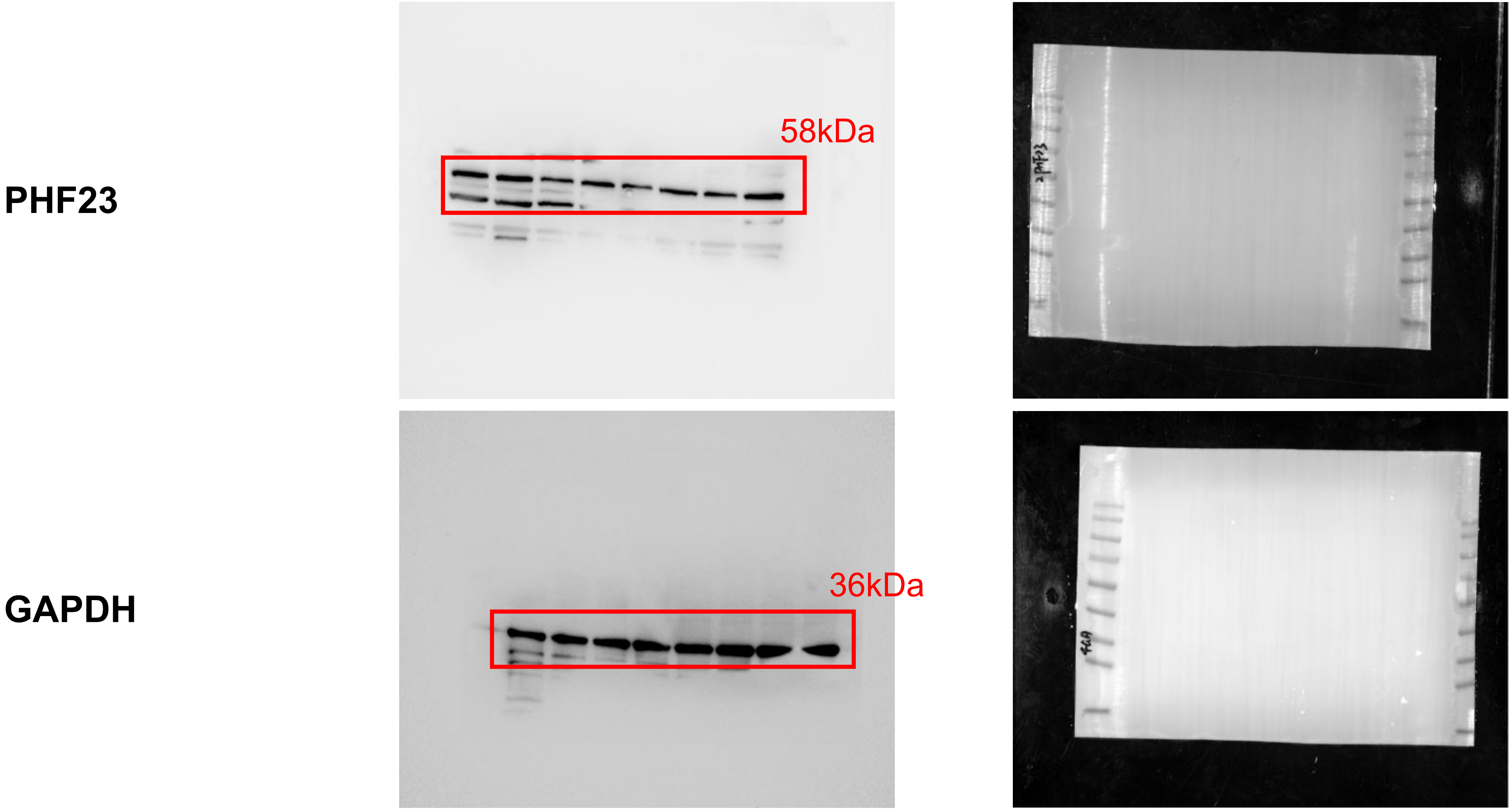

# Figure2D

PHF23

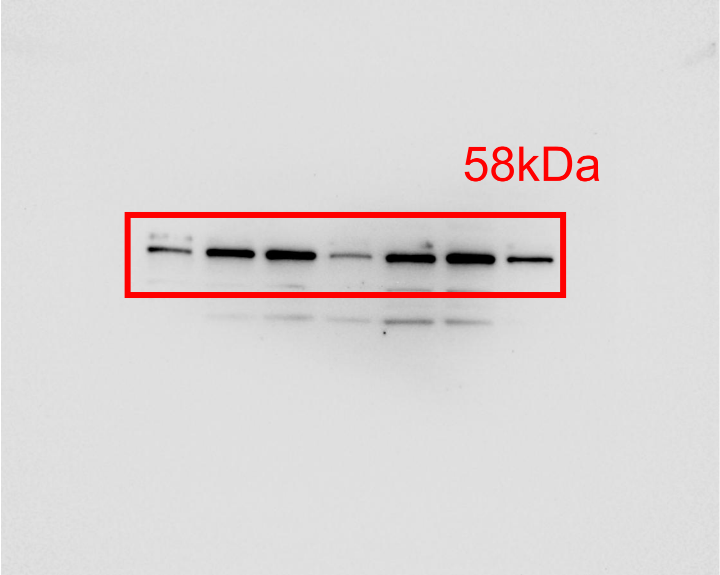


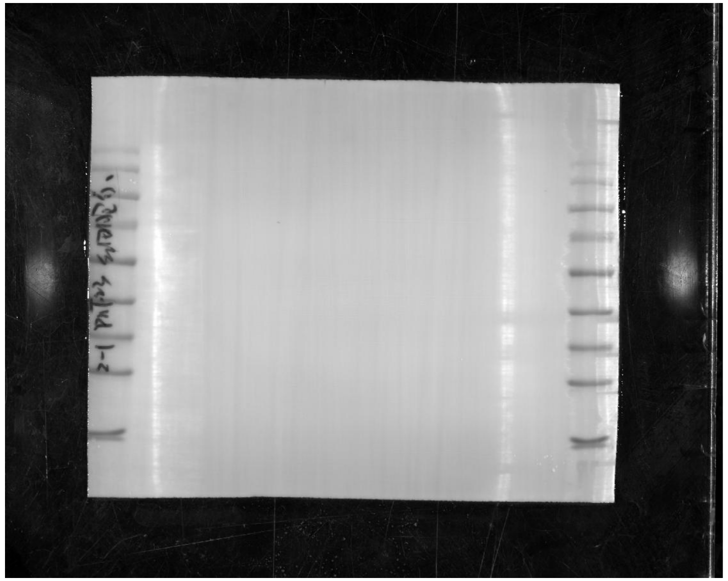

GAPDH

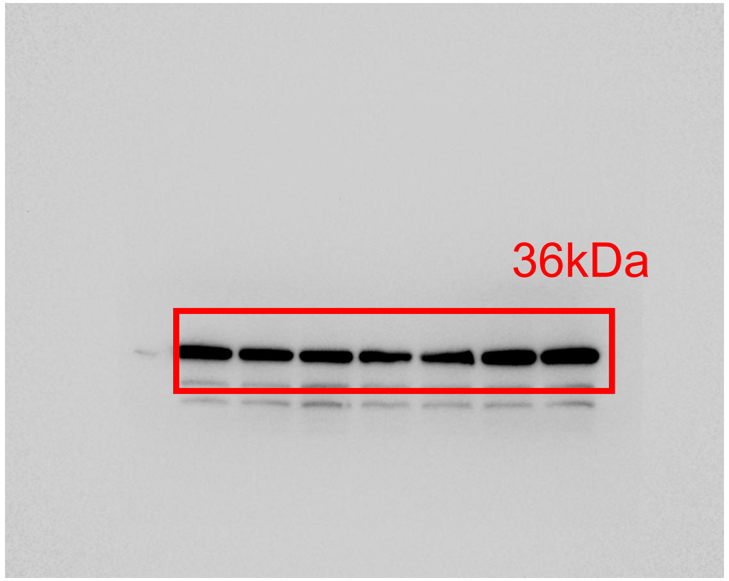


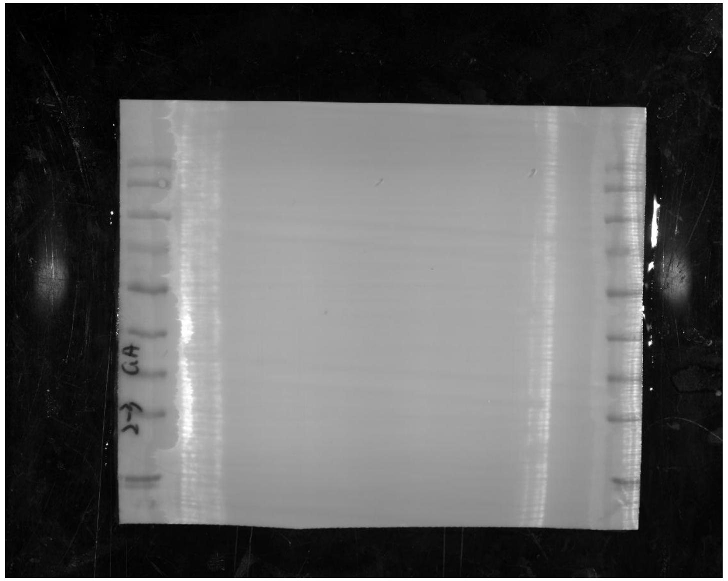

# Figure2A

H1299 PHF23

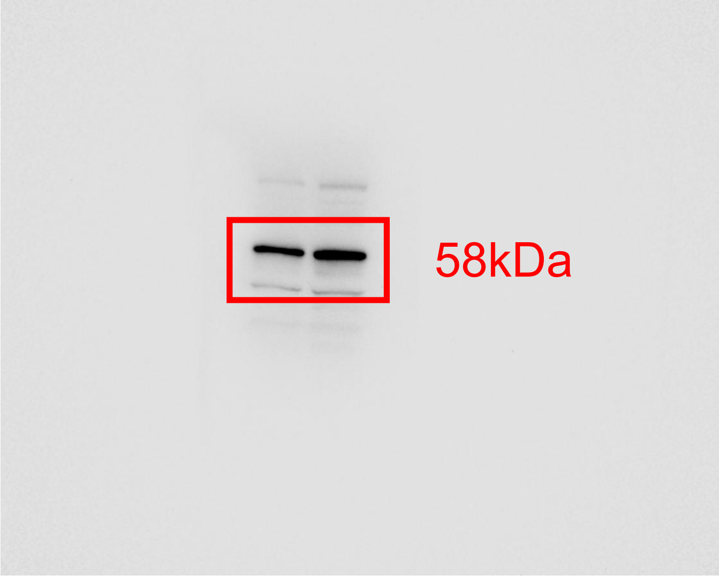

58kDa

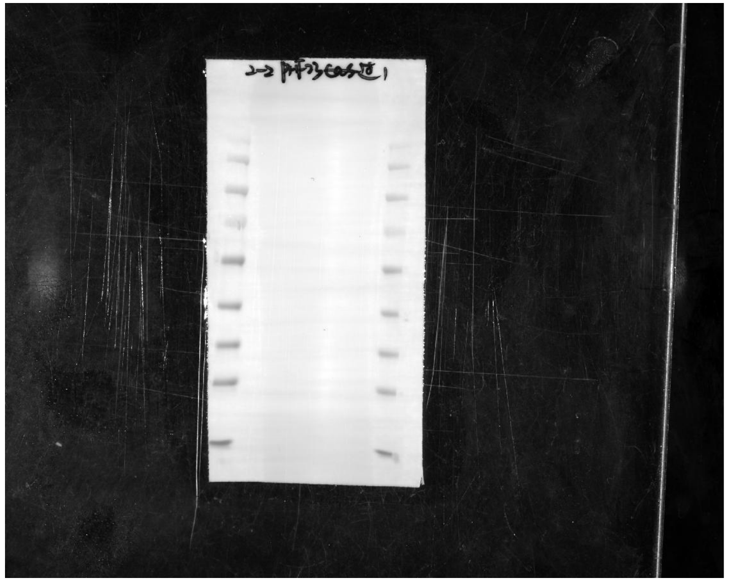

A549 PHF23

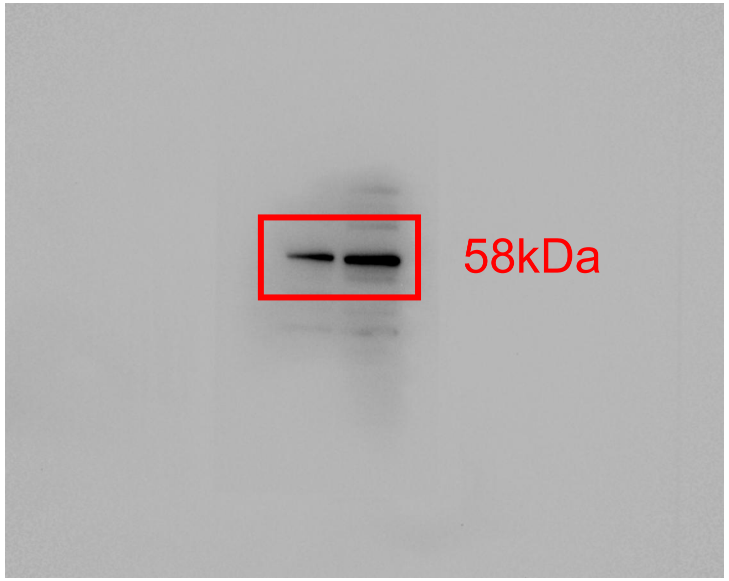

58kDa

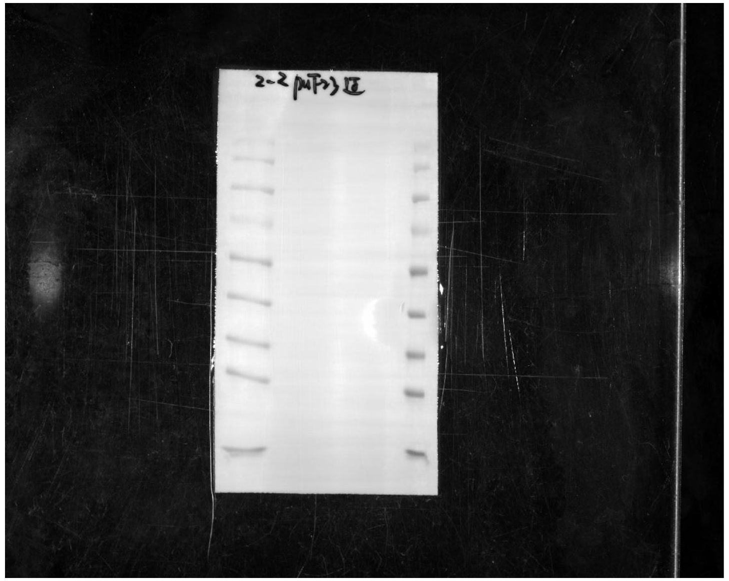

H1299 GA

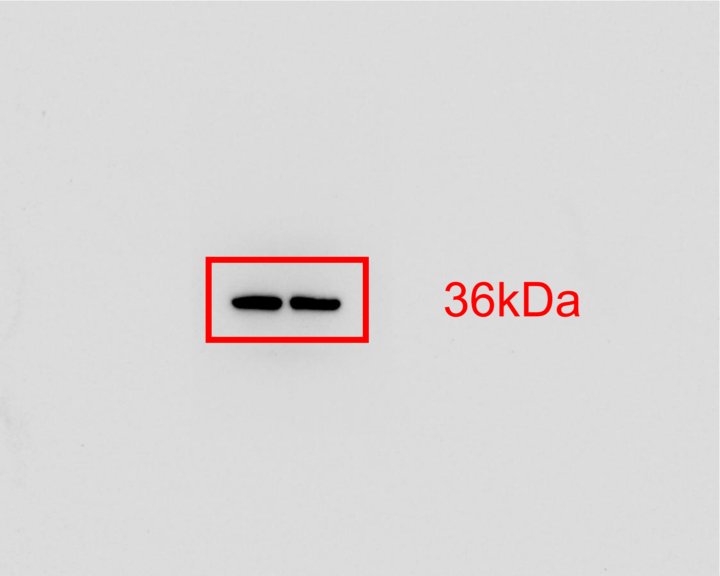


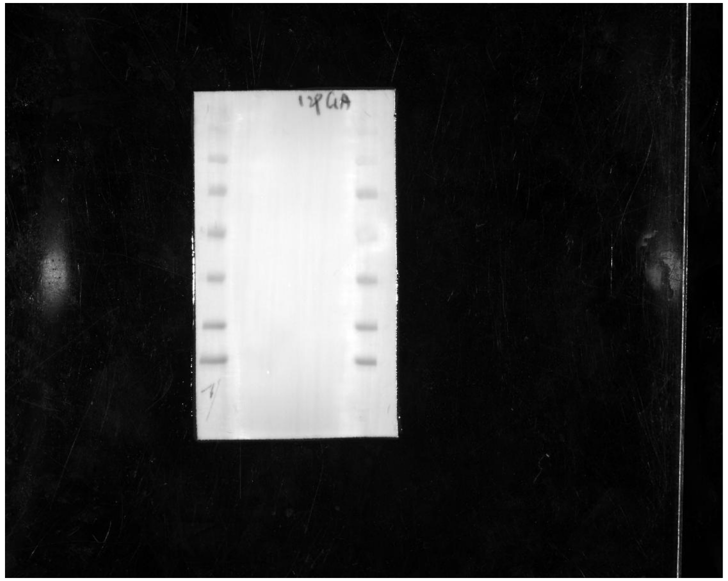

A549 GA

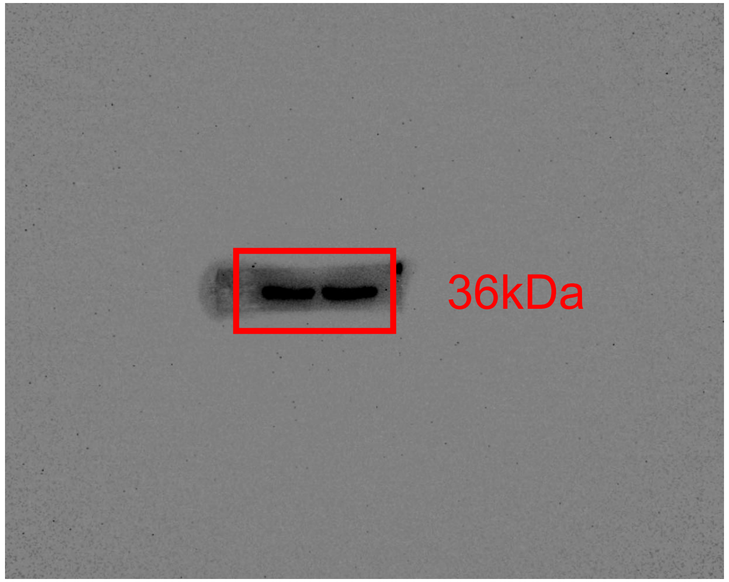


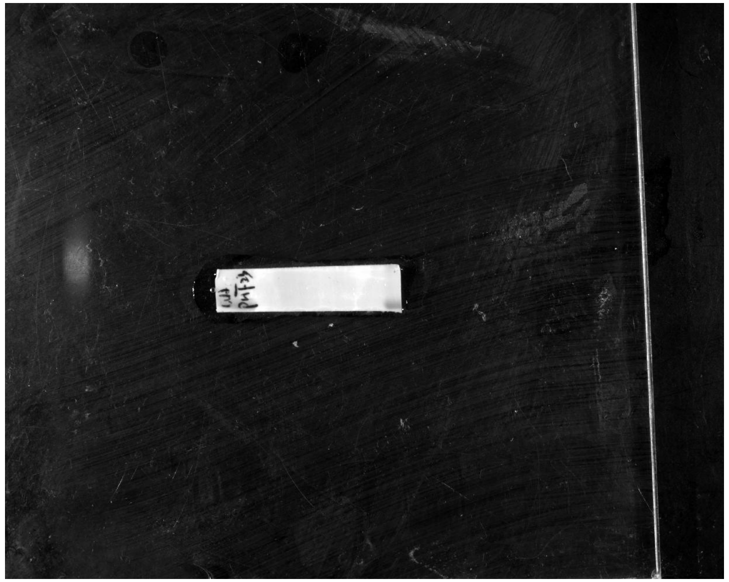

# cas9

H1299 P H F 2 3

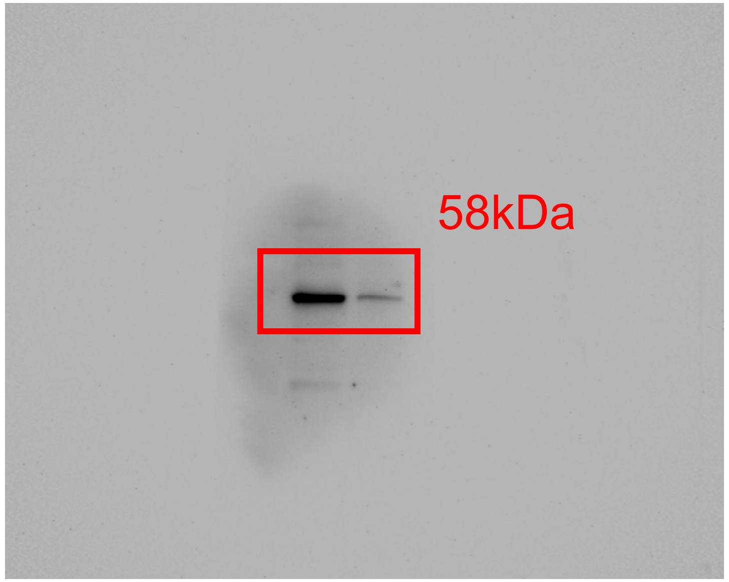


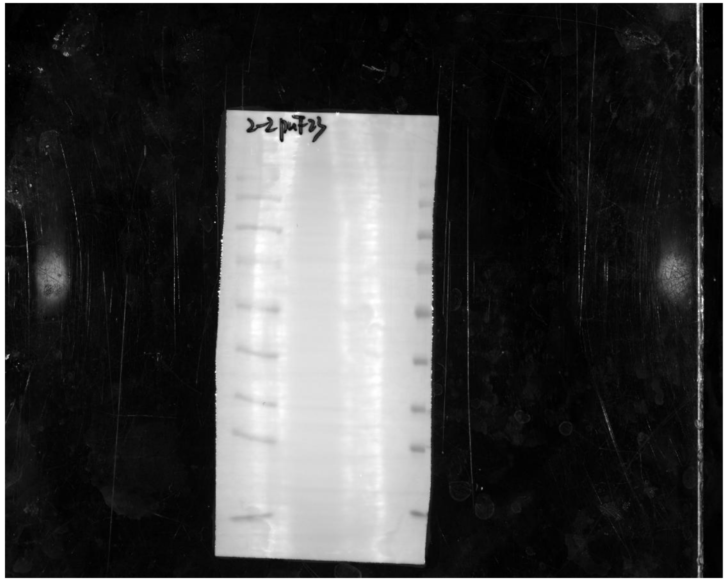

H1975 P H F 2 3

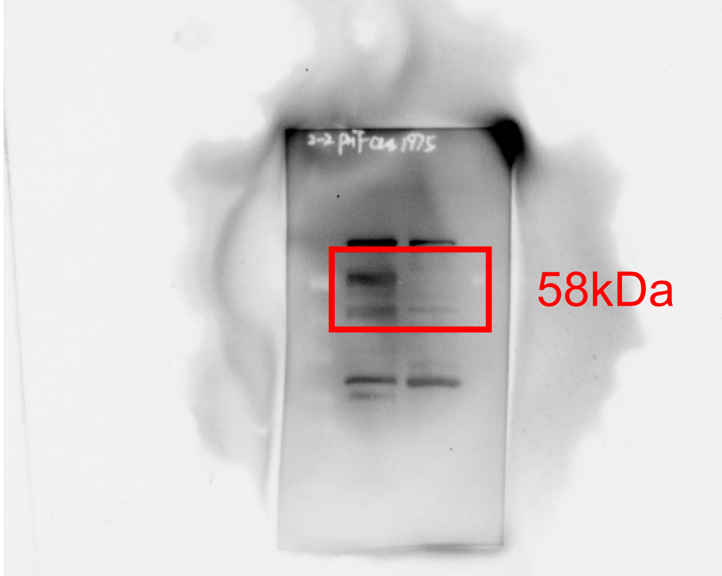


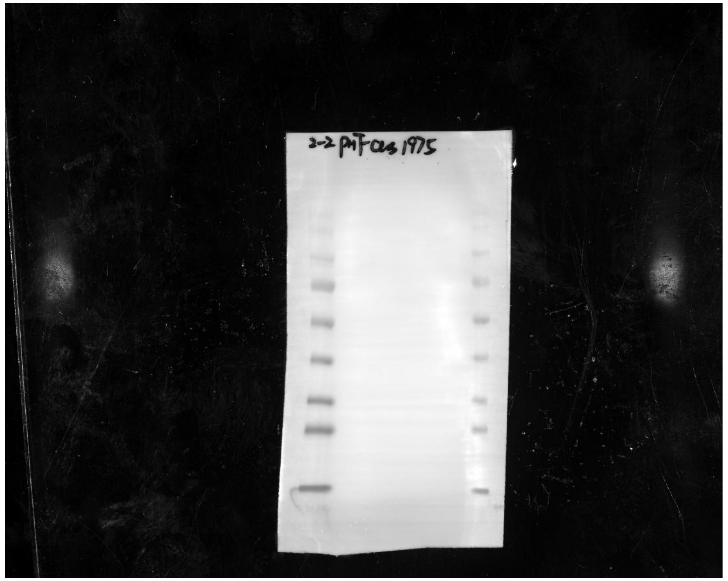

# cas9

H1299 ga

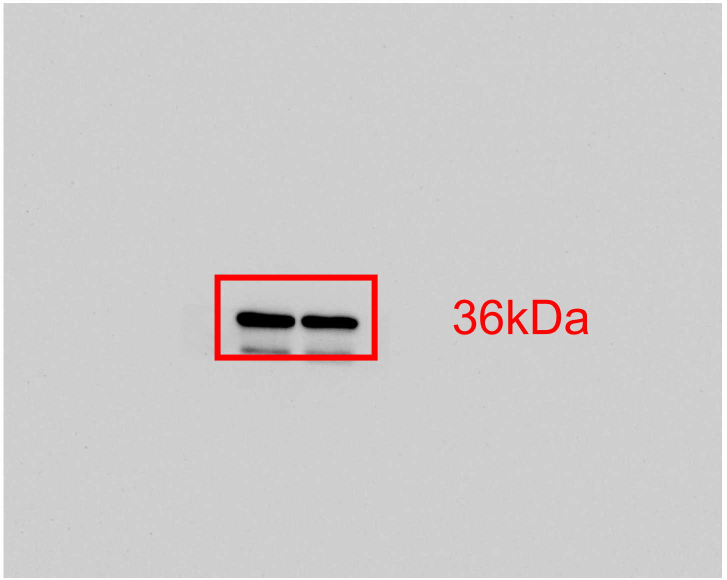


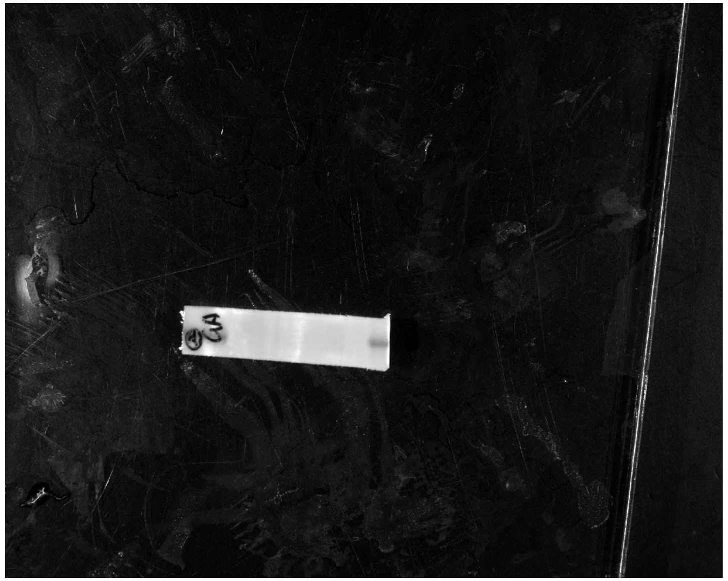

H1975 ga

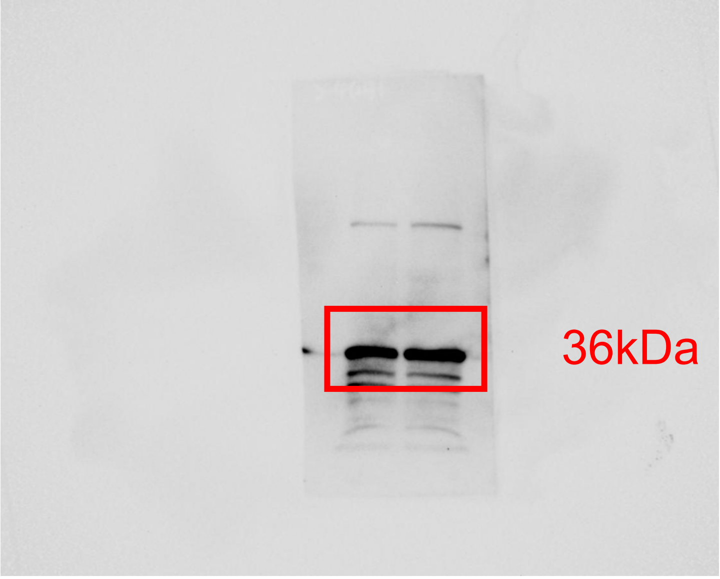


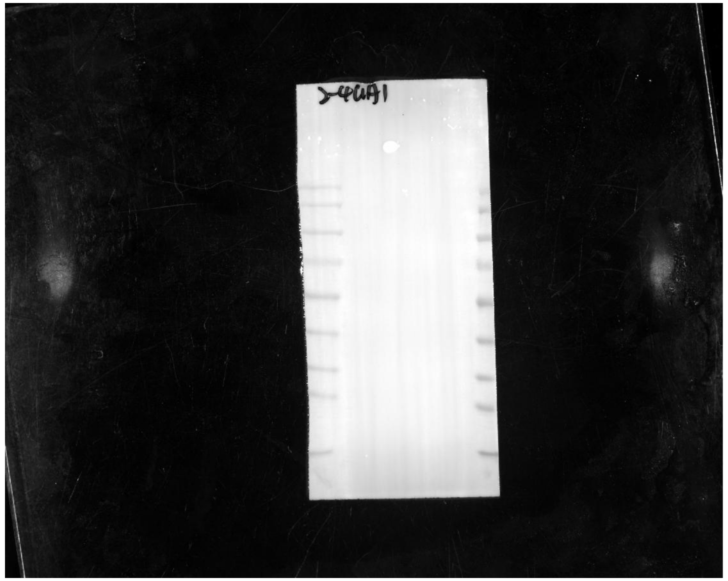

# 2E

H1299 Bax

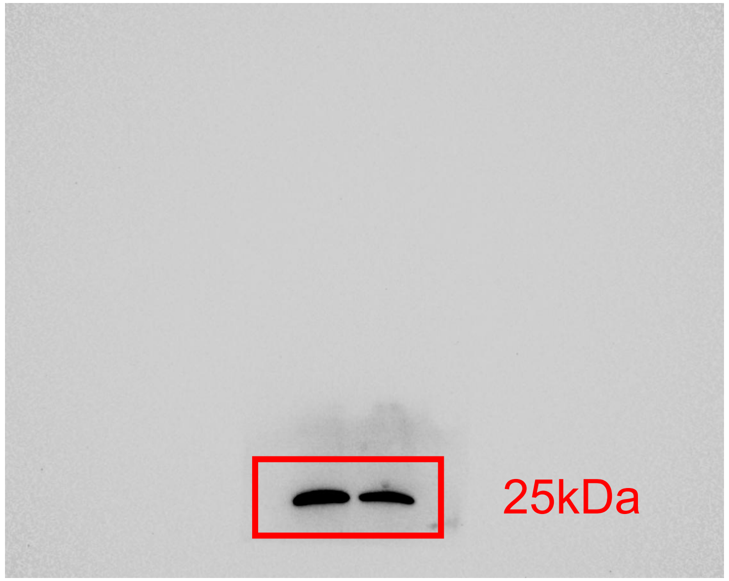


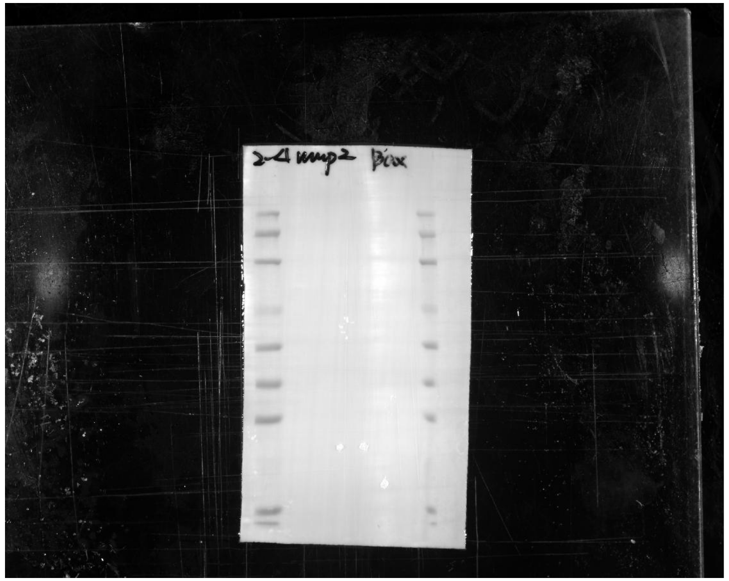

A549 Bax

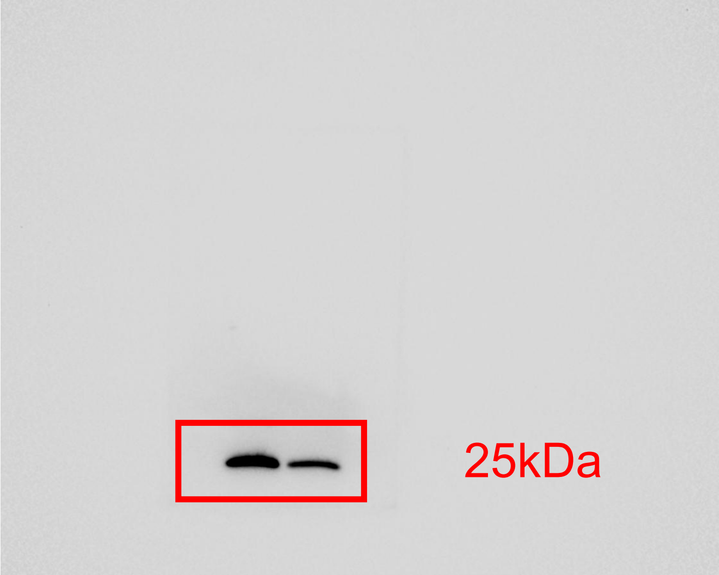


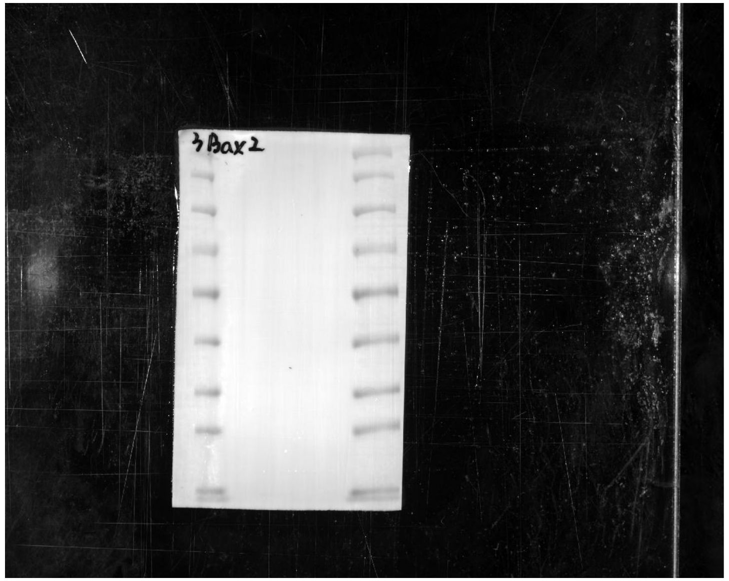

H1299 Bcl-2

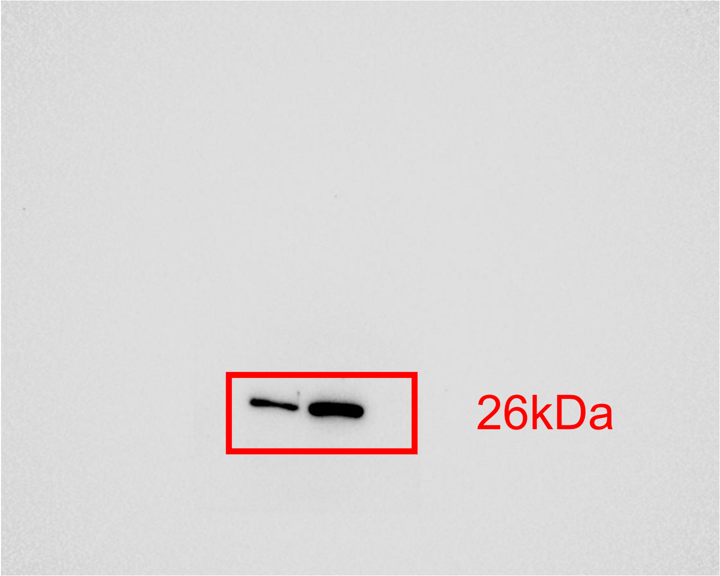


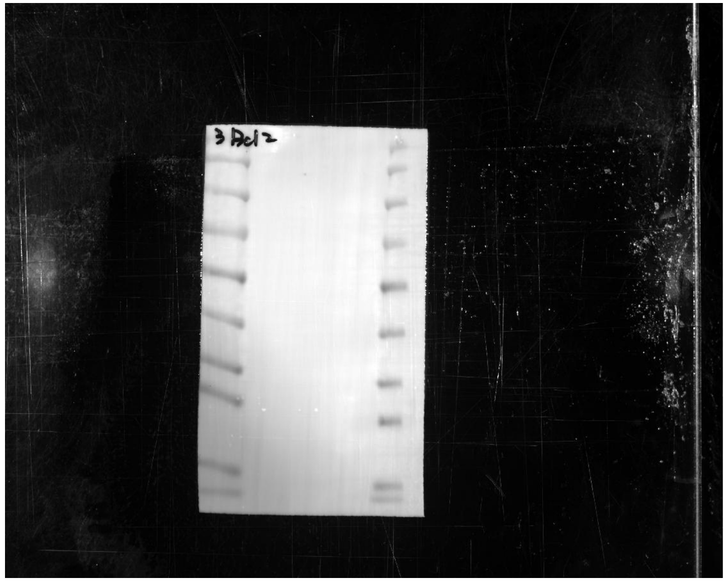

A549 Bcl-2

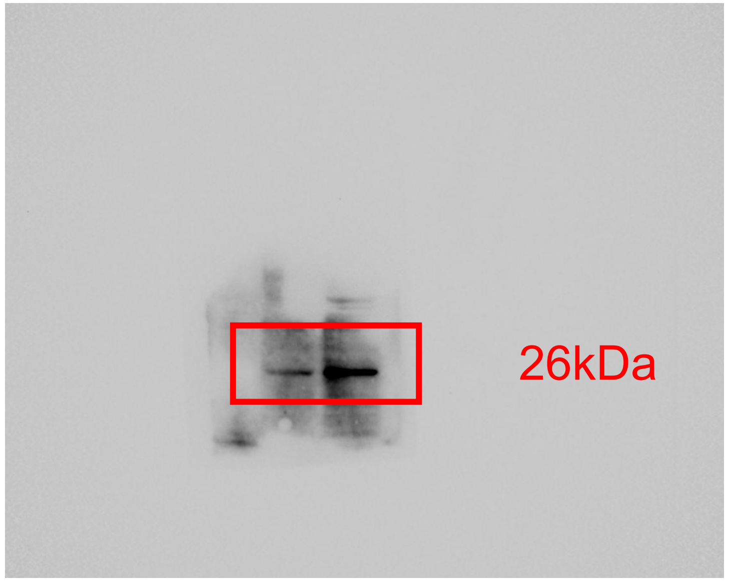


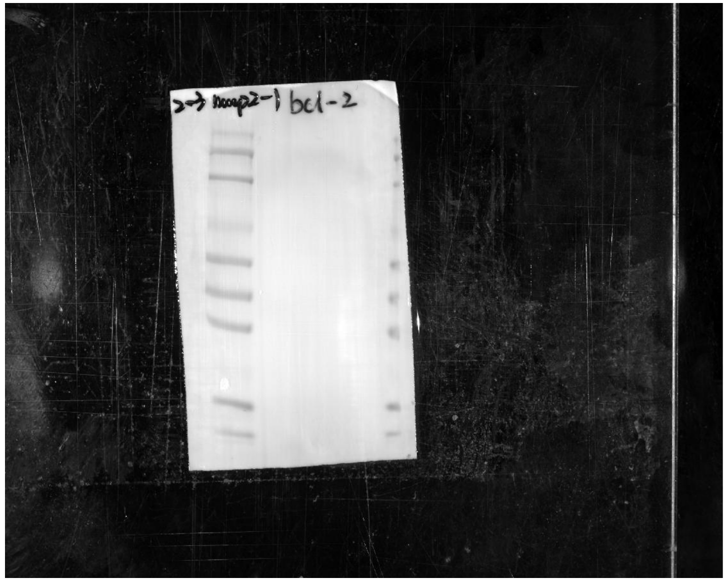

H1299
Caspase3

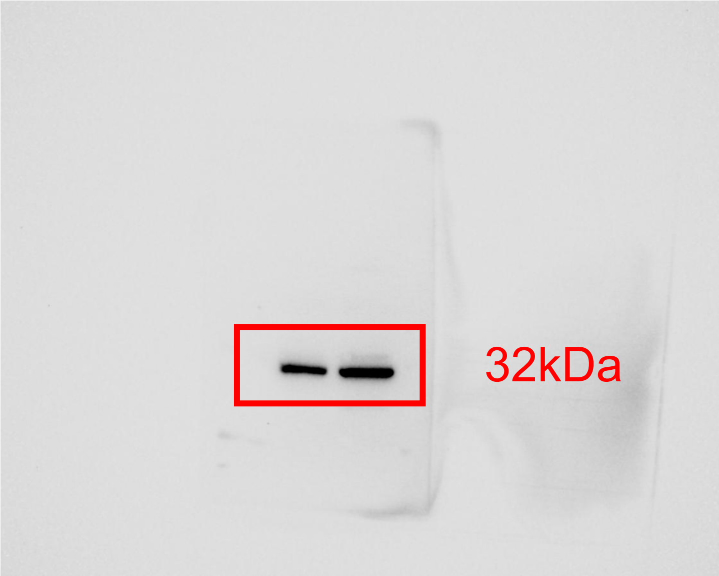


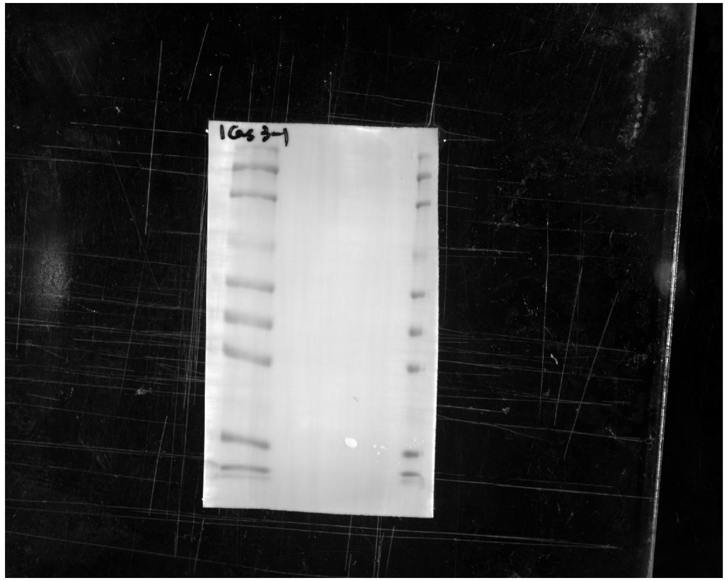

A549
Caspase3

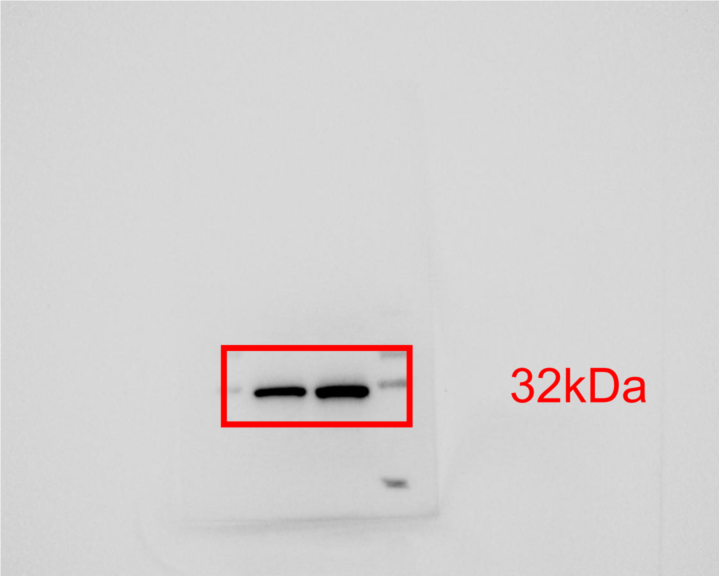


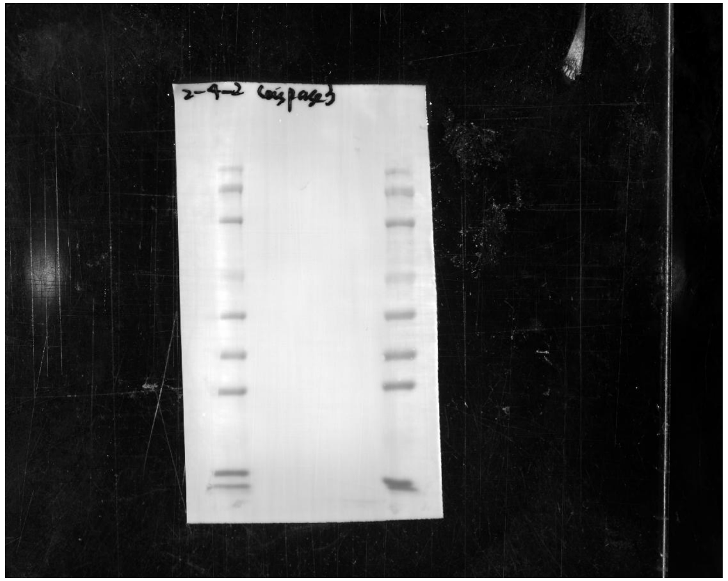

H1299
Cleaved-
Caspase3

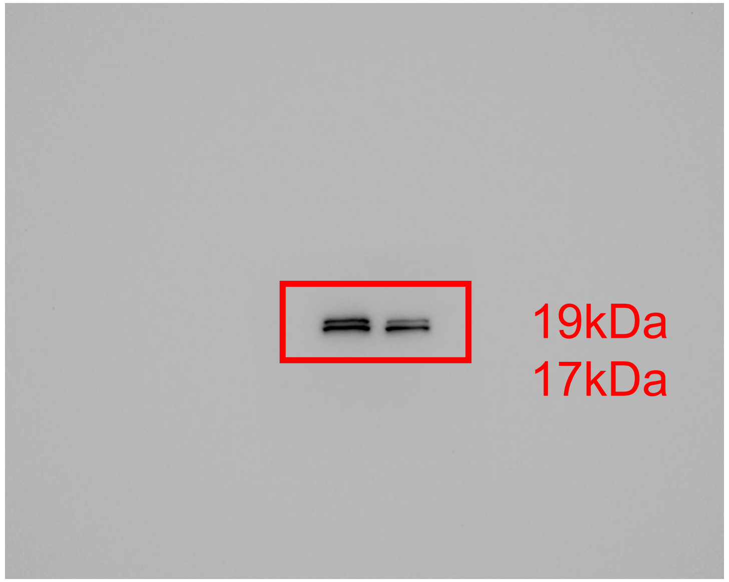


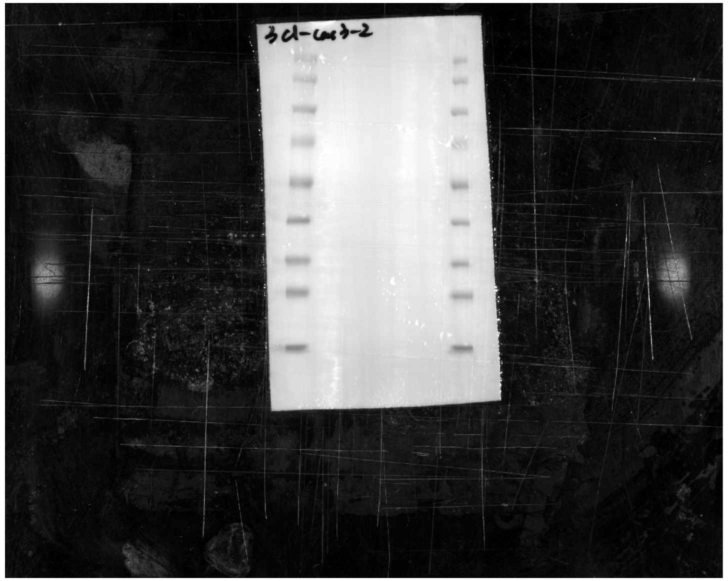

A549 Cleaved-
Caspase3

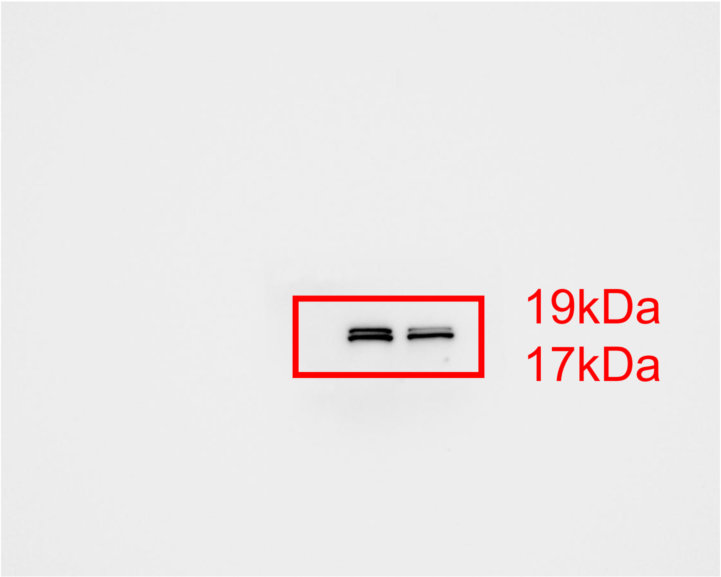


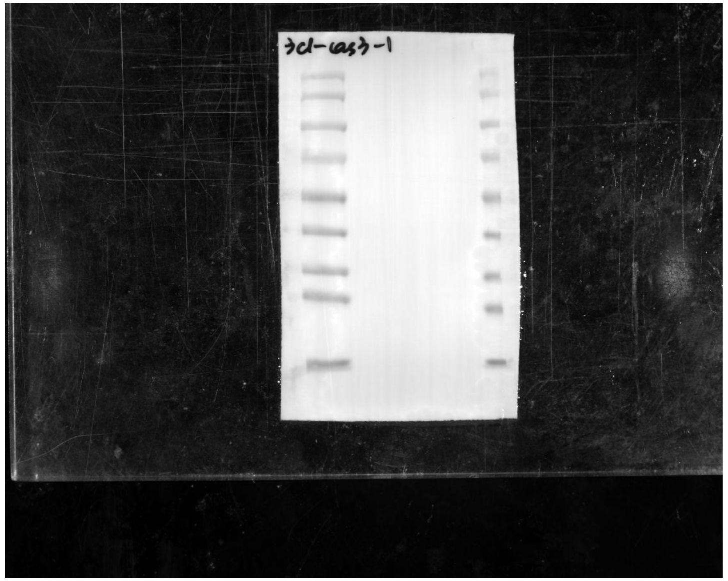

H1299
PARP
Cleaved-PARP

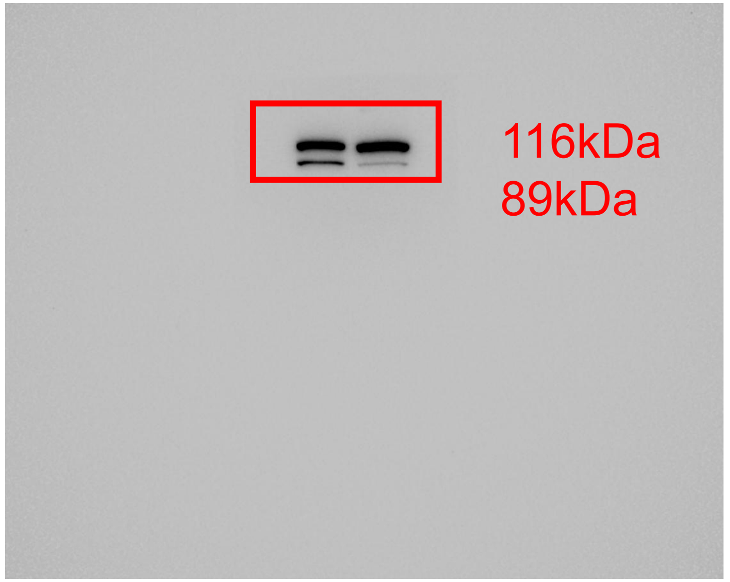


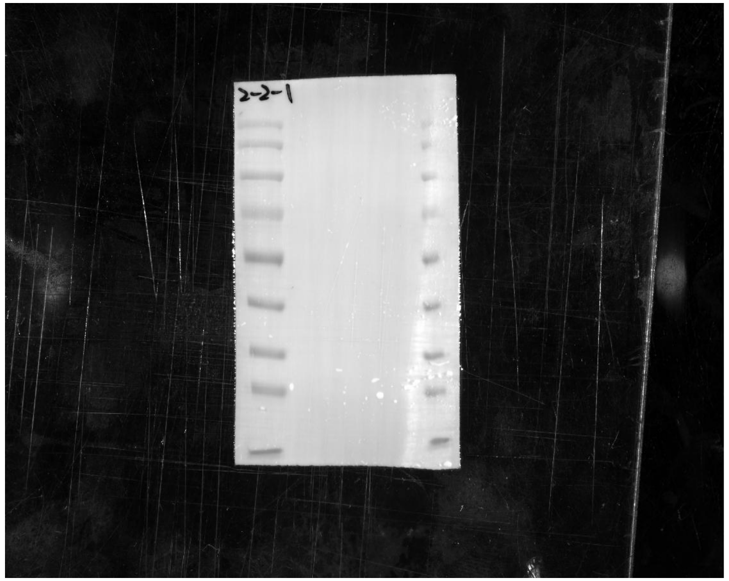

A549
PARP
Cleaved-PARP

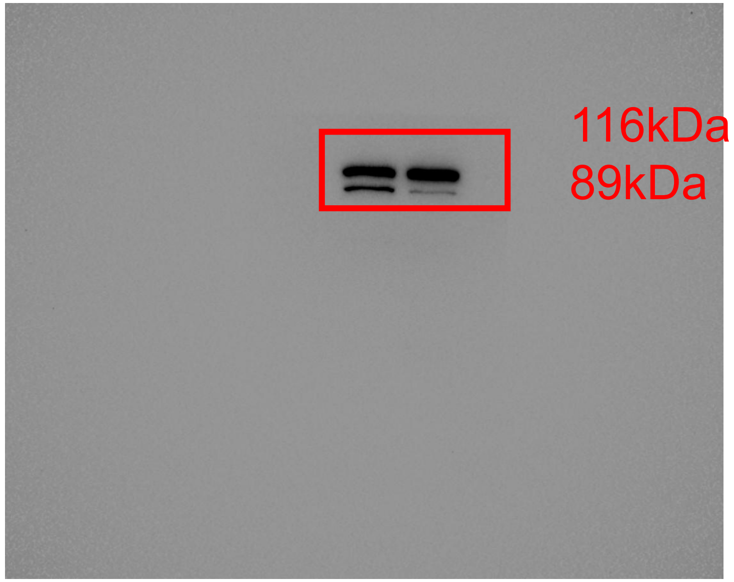


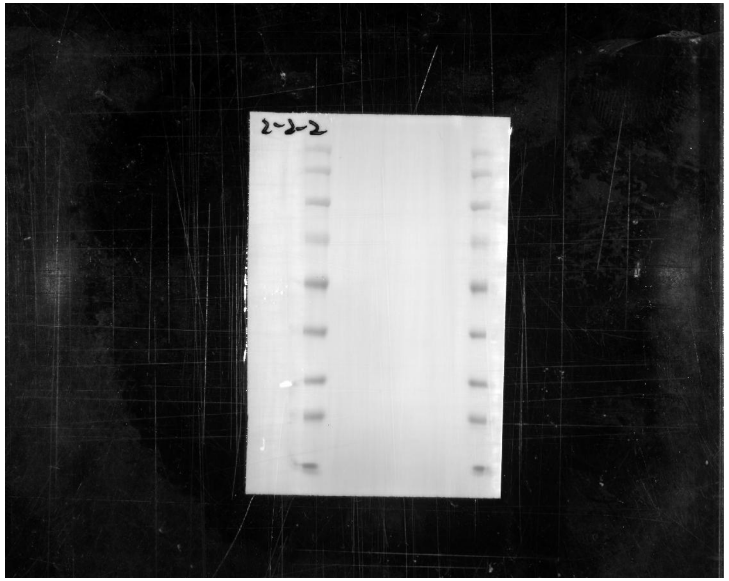

H1299 GAPDH

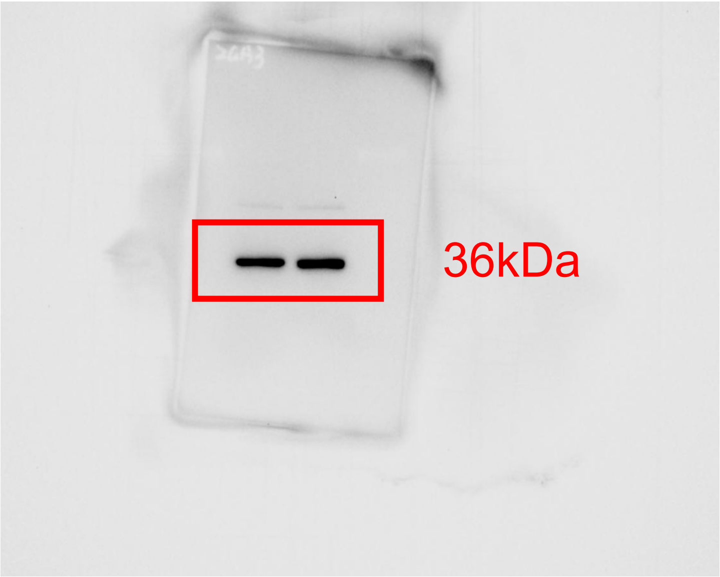


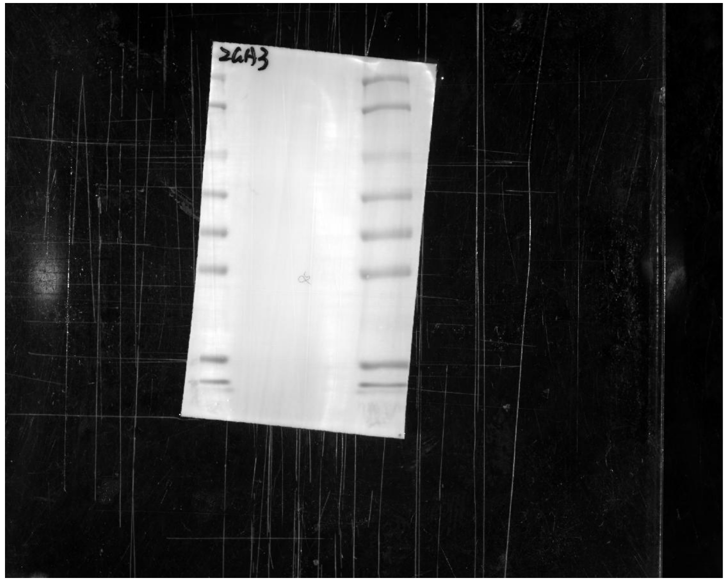

A549 GAPDH

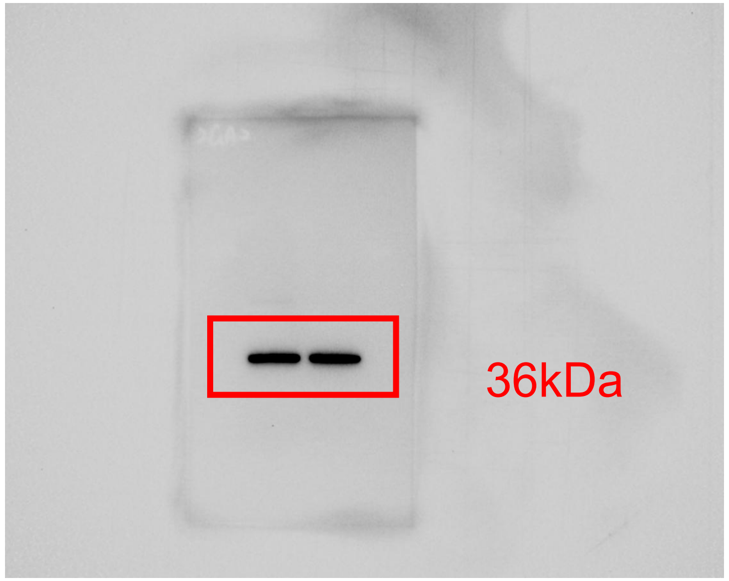


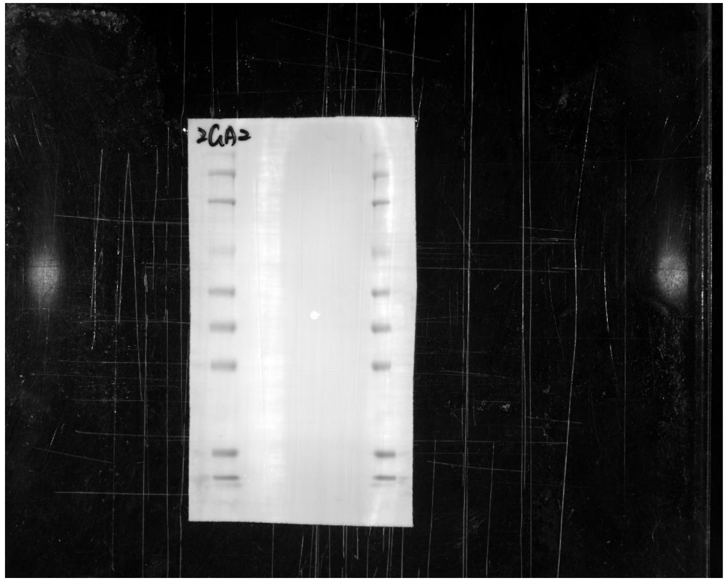

# Figure2H

H1299 CDK4

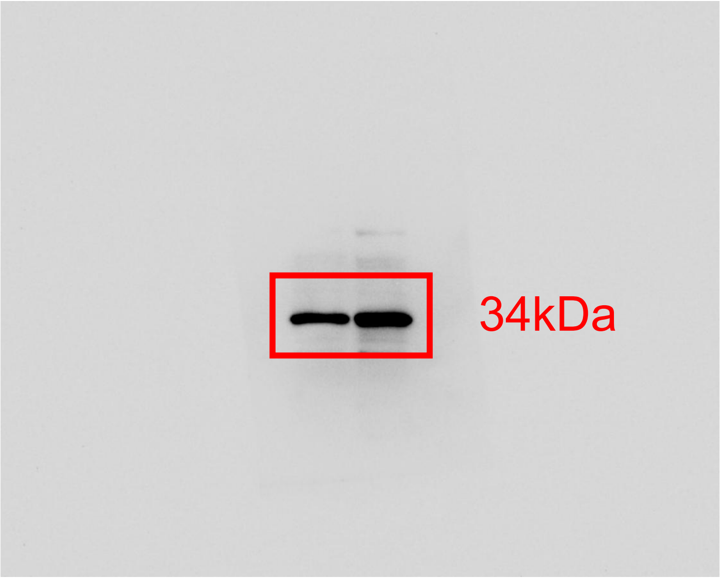


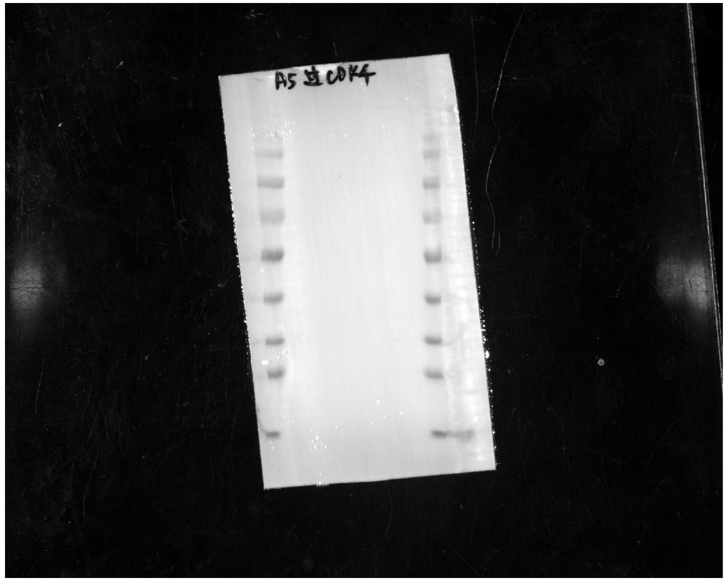

A549 CDK4

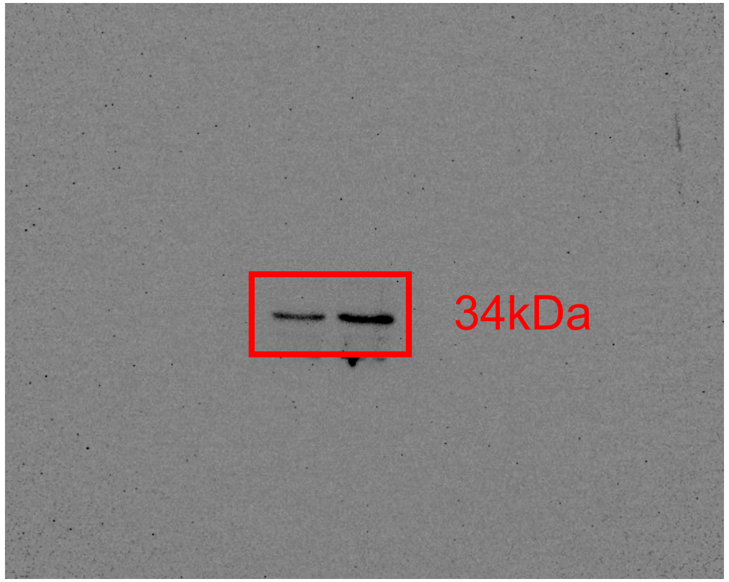


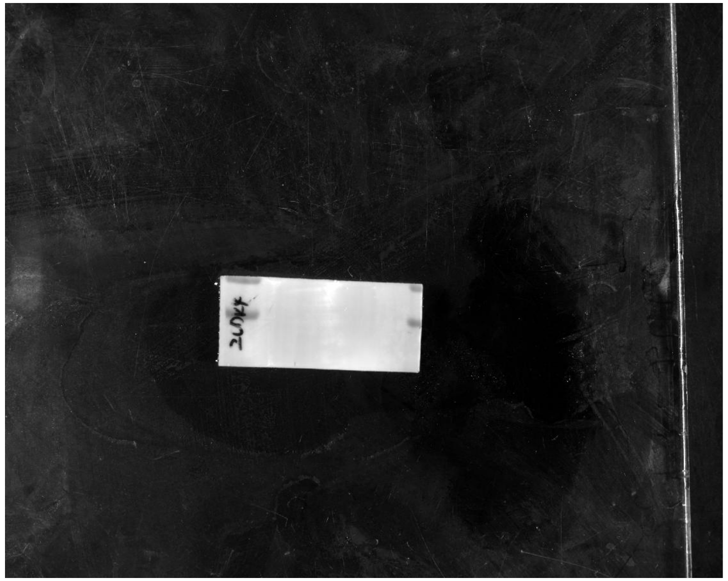

H1299 CDK6

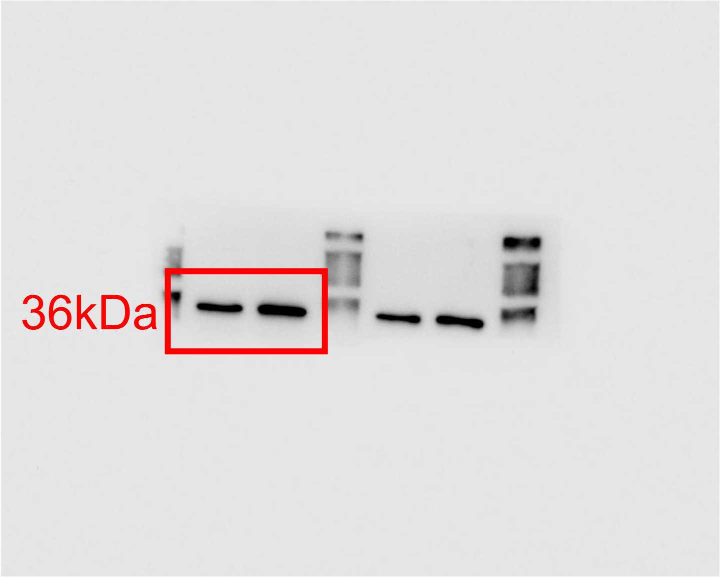


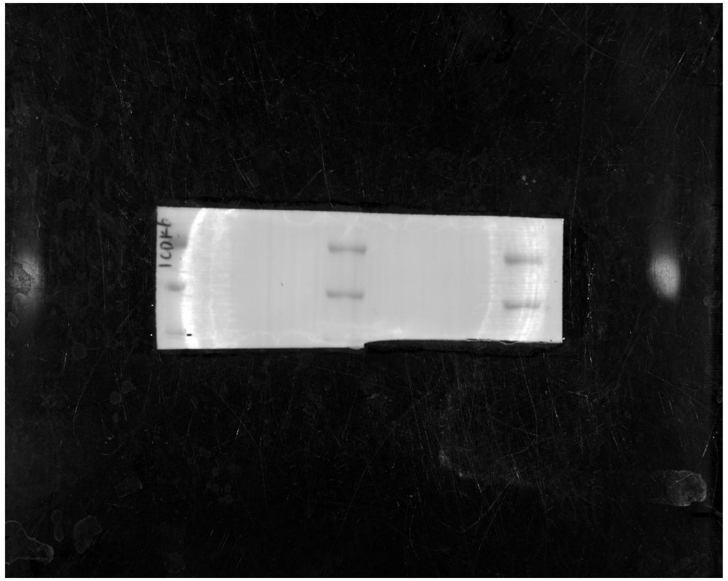

A549 CDK6

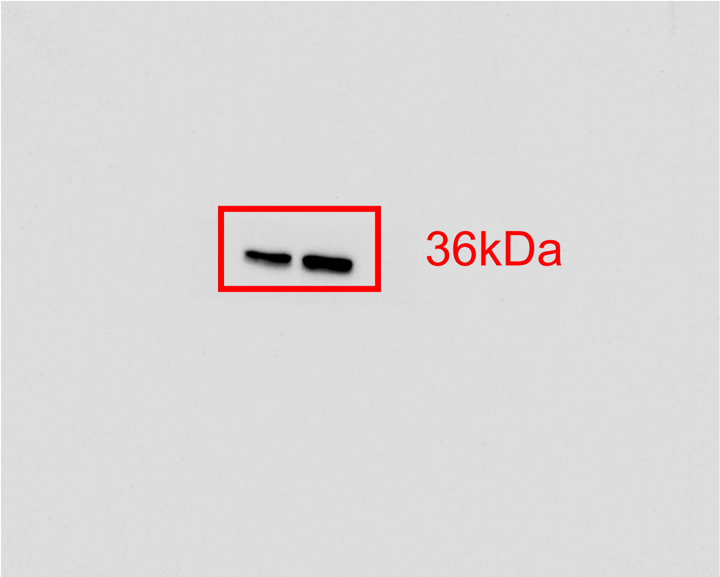


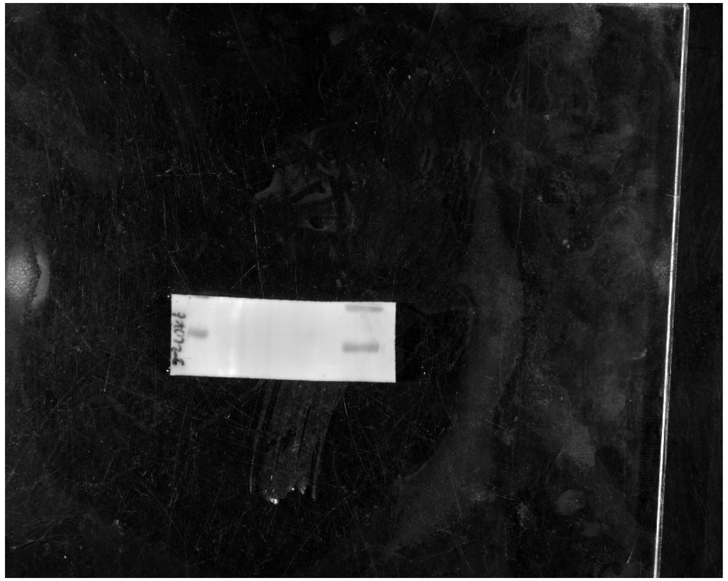

H1299 A2

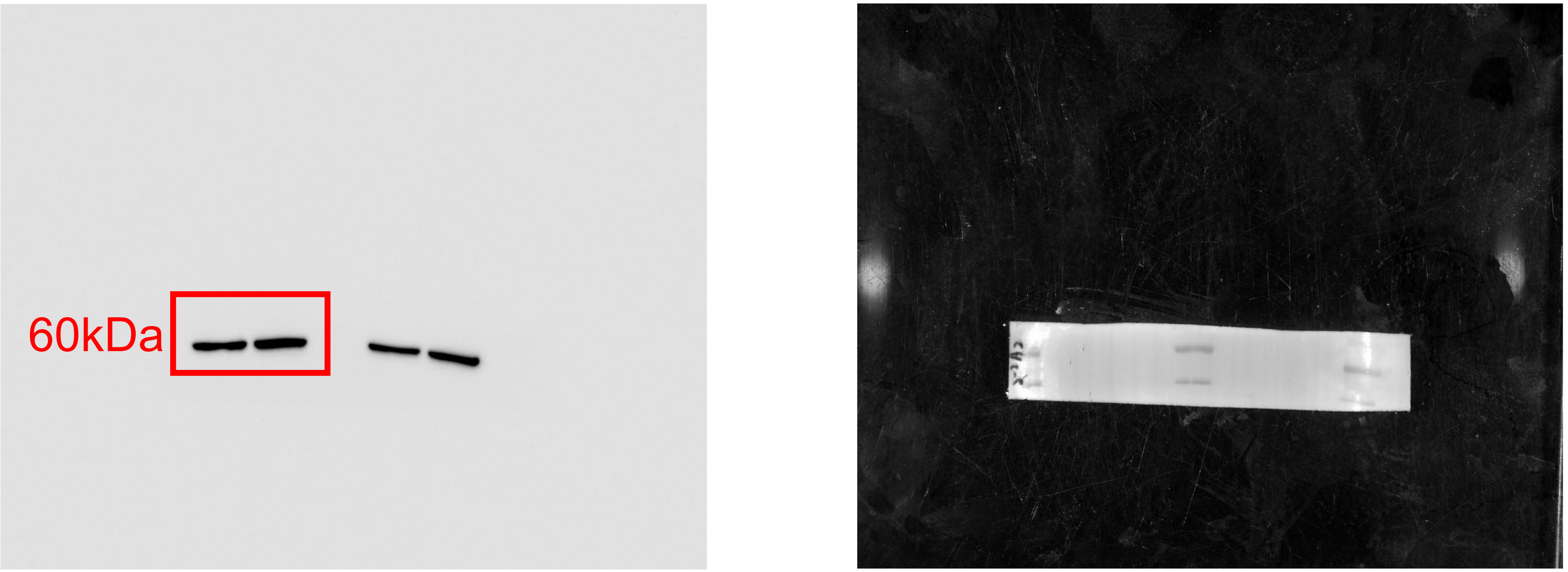


A549 A2

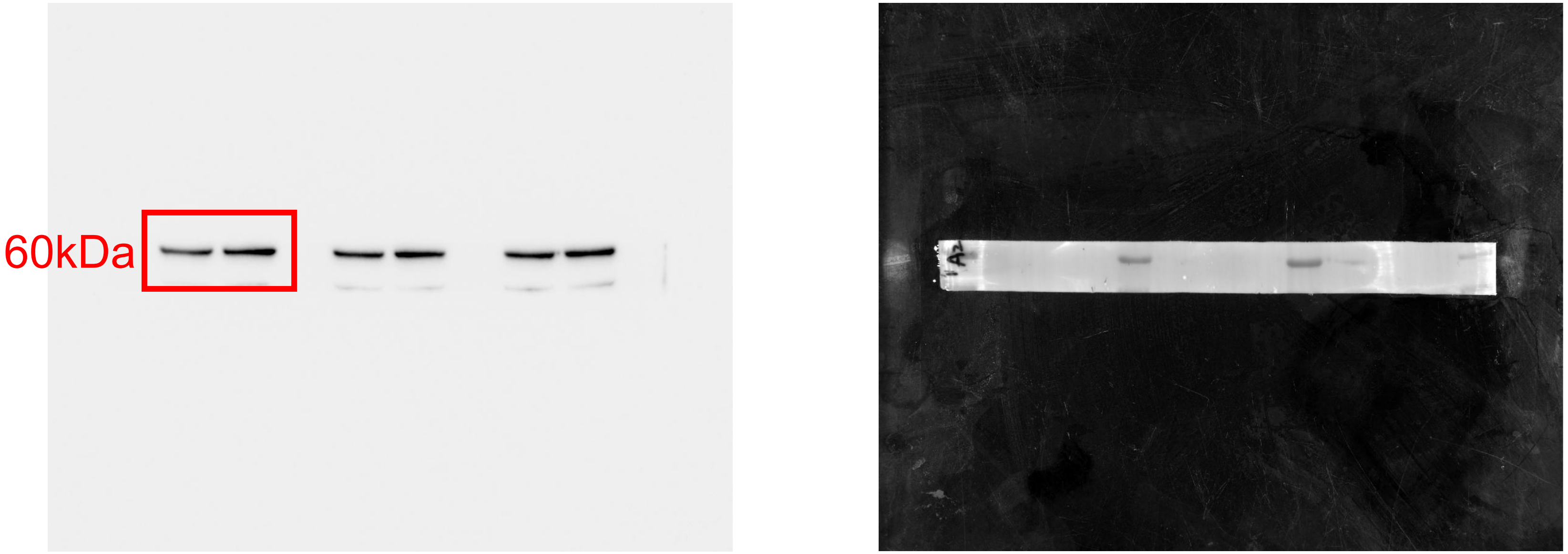

H1299 D1

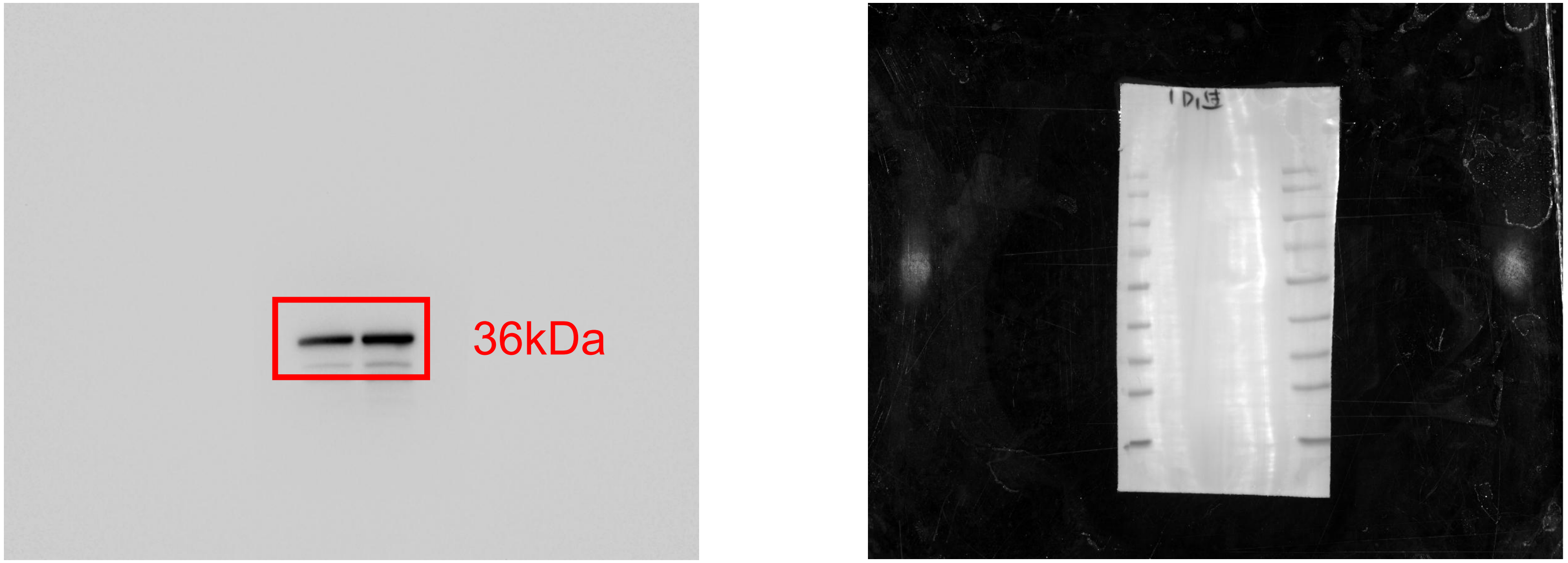


A549 D1

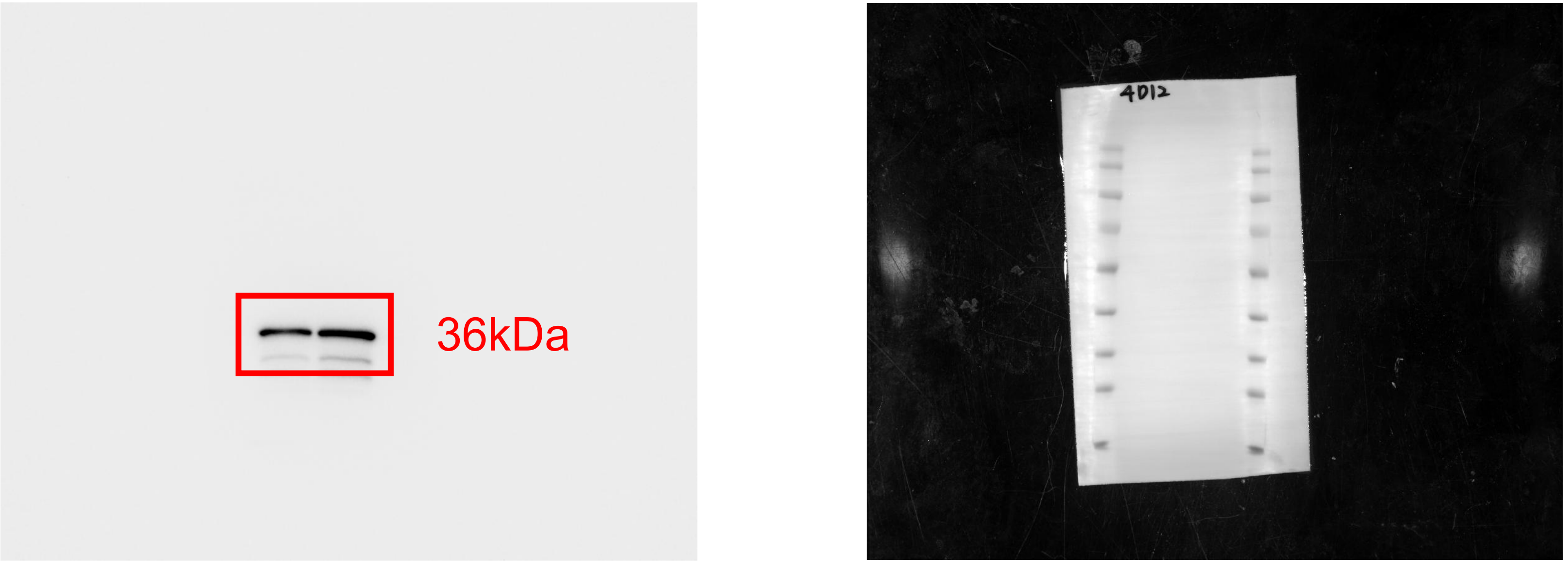

H1299 E-ca

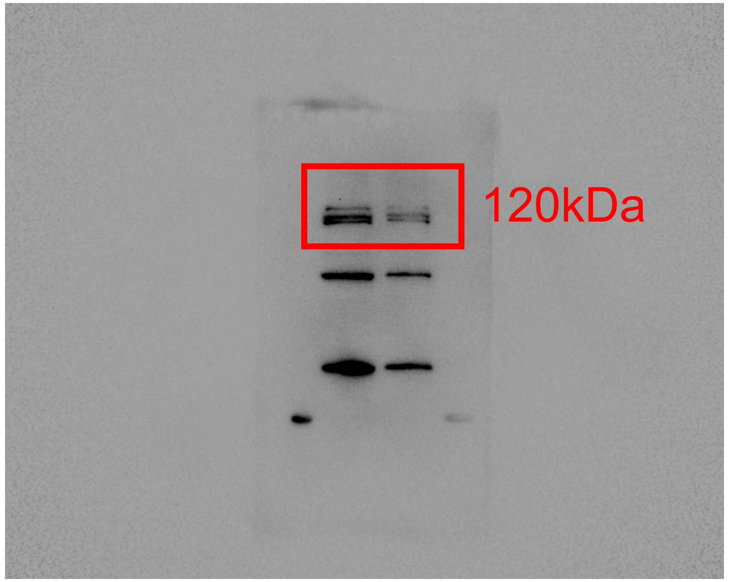


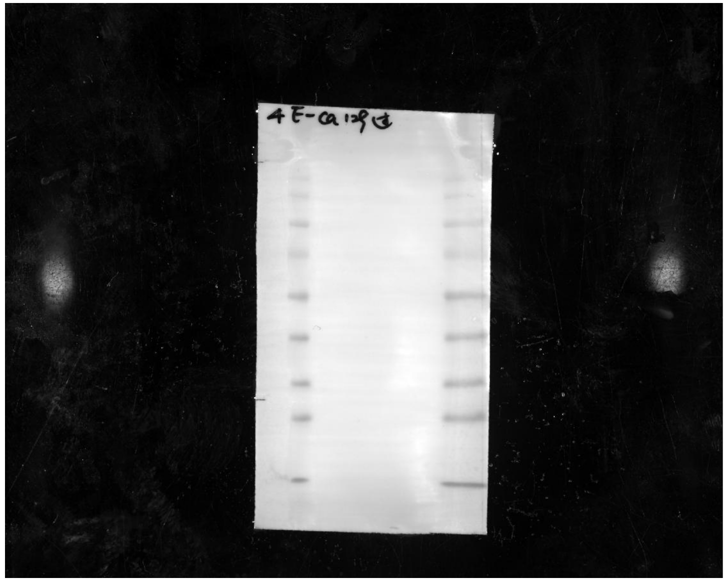

A549 E-ca

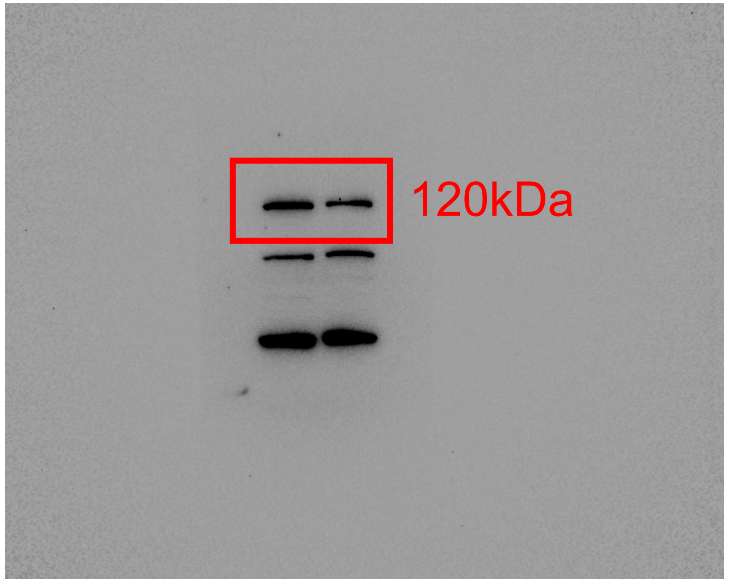


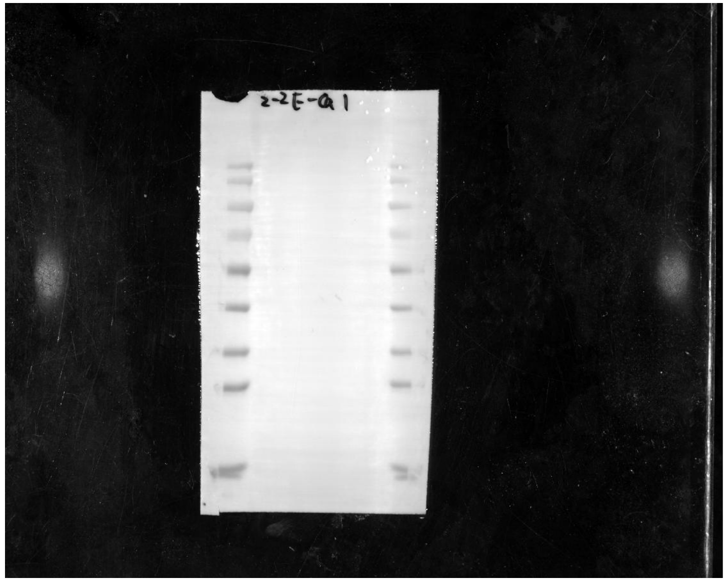

H1299 N-ca

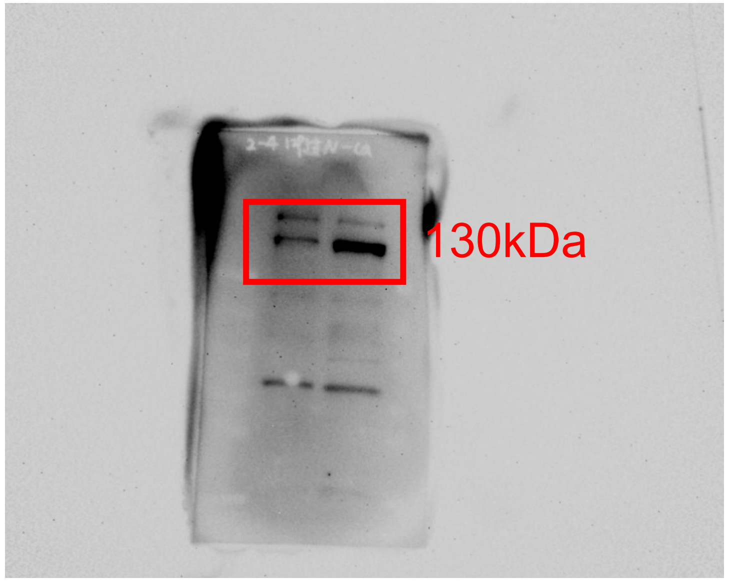


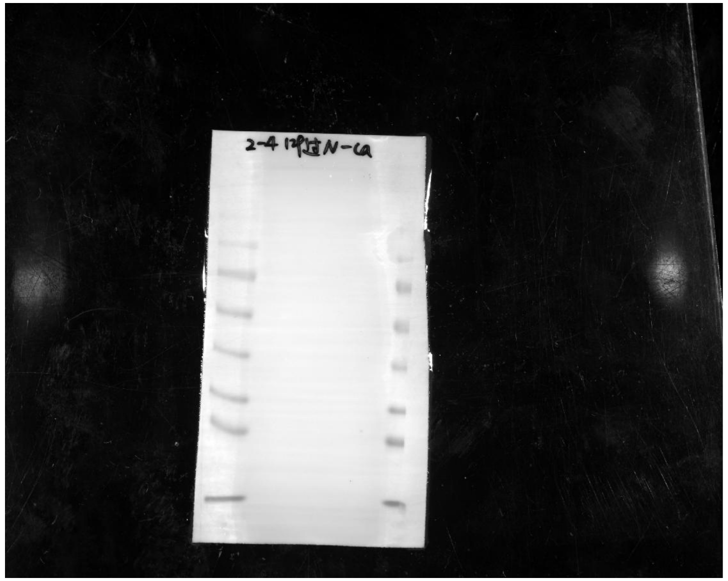

A549 N-ca

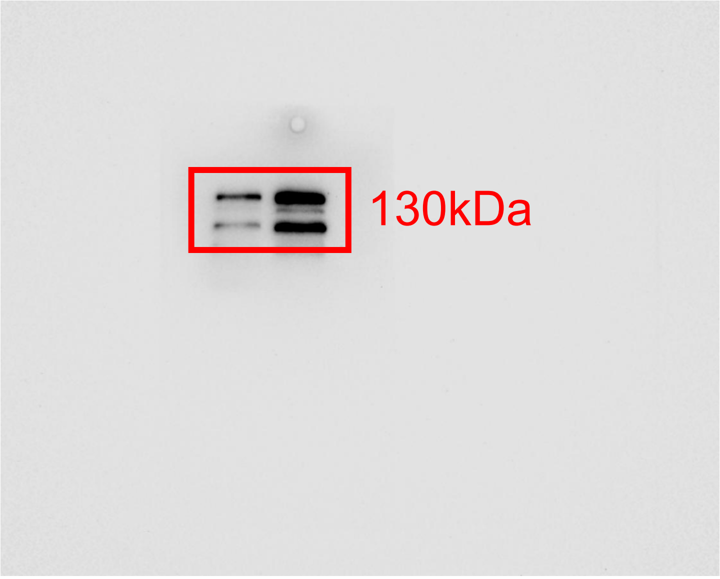


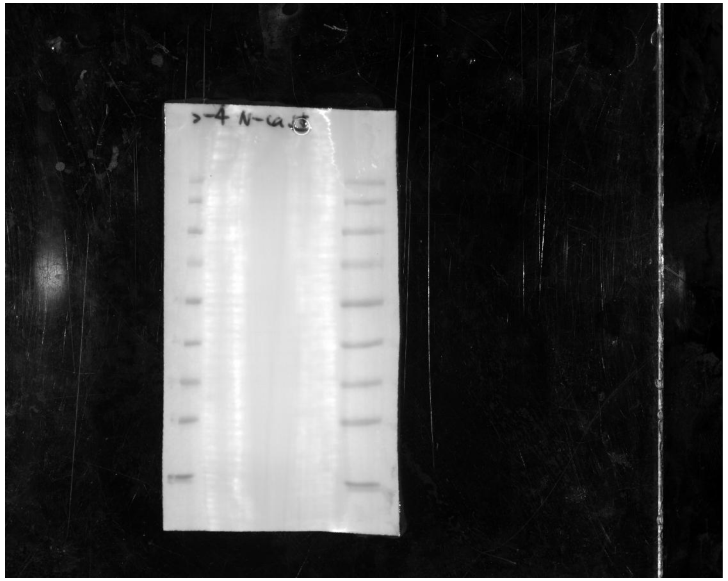

H1299 MMP9

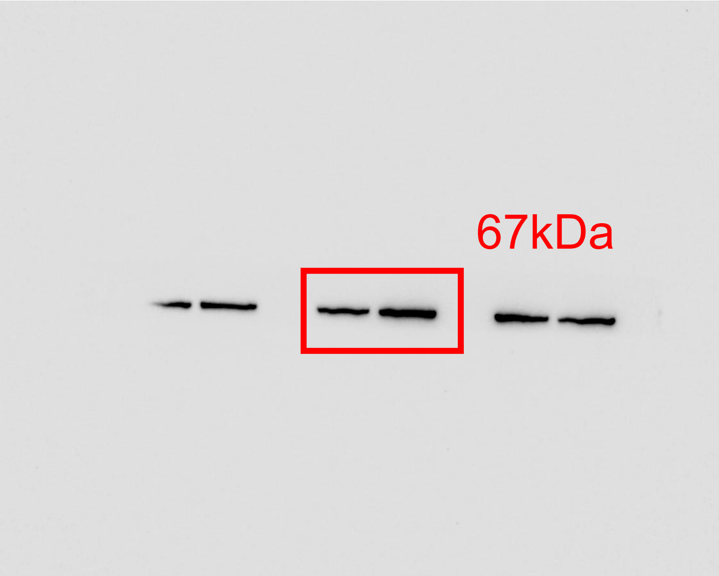


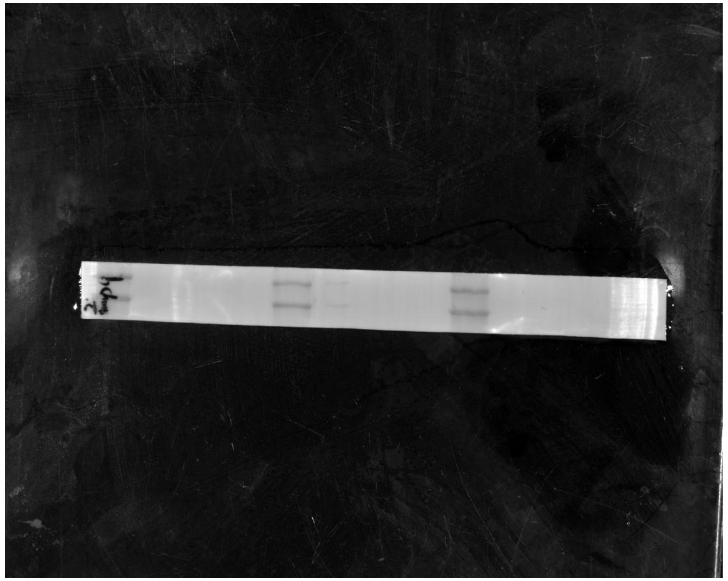

A549 MMP9

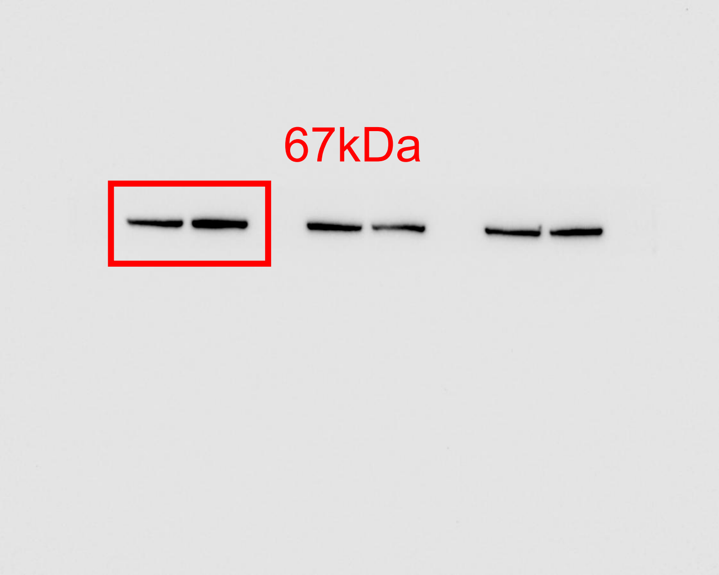


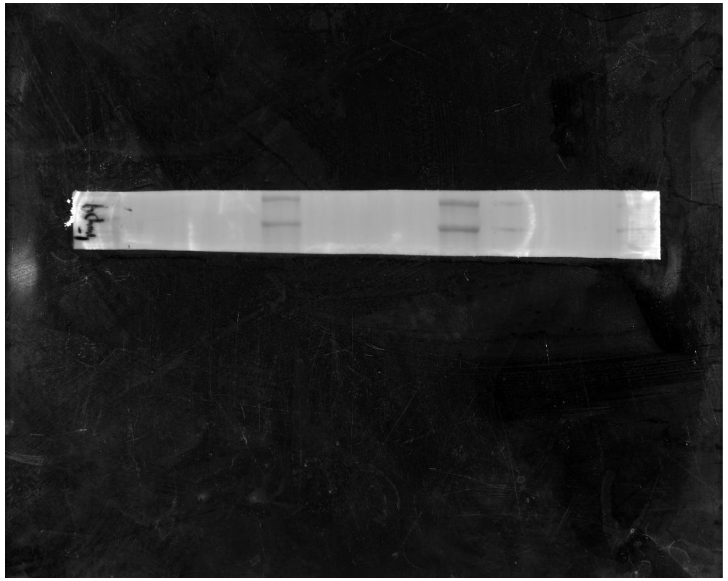

GAPDH

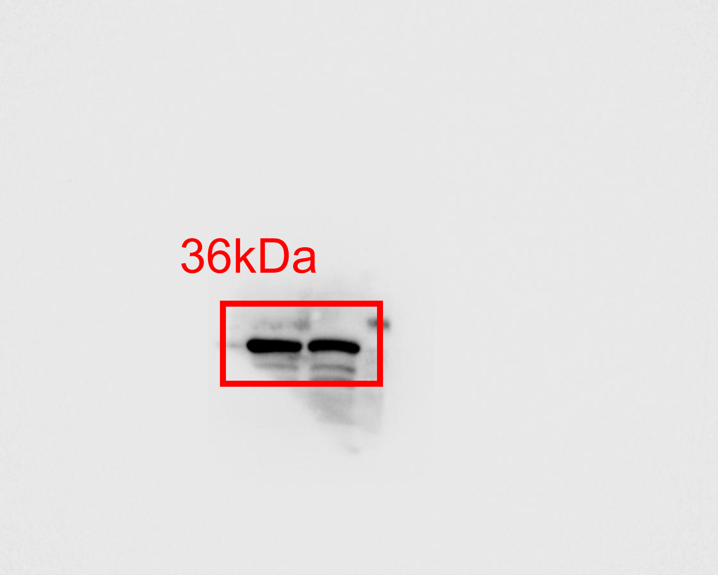


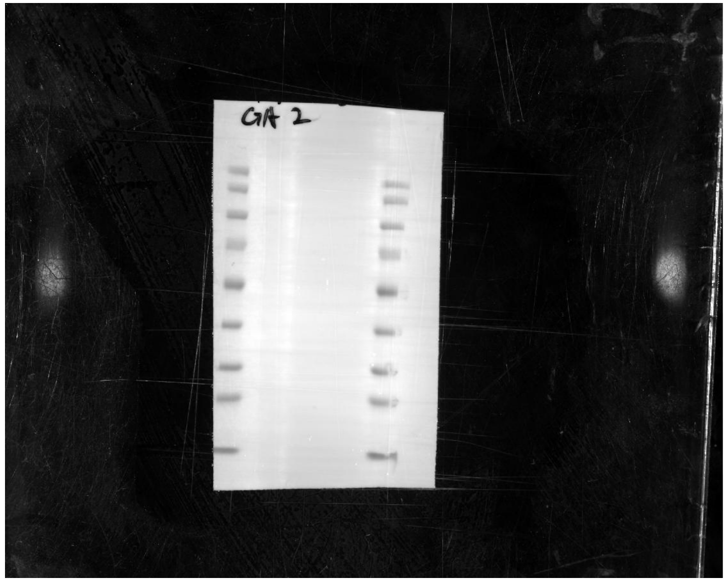

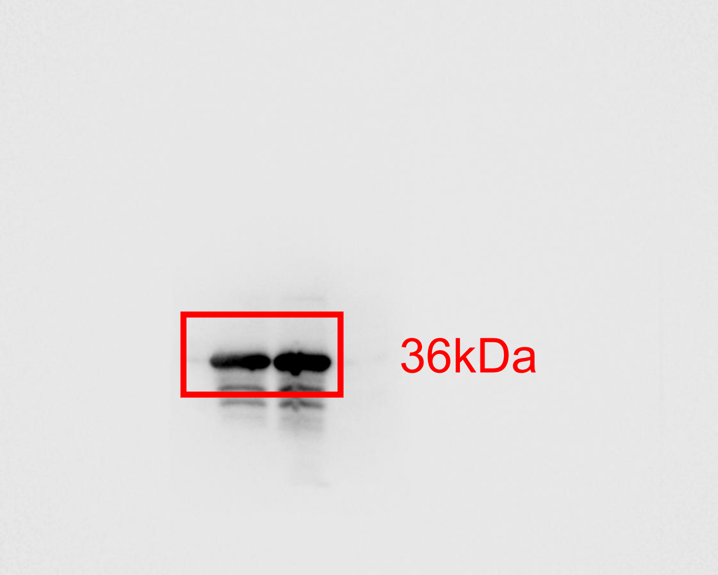


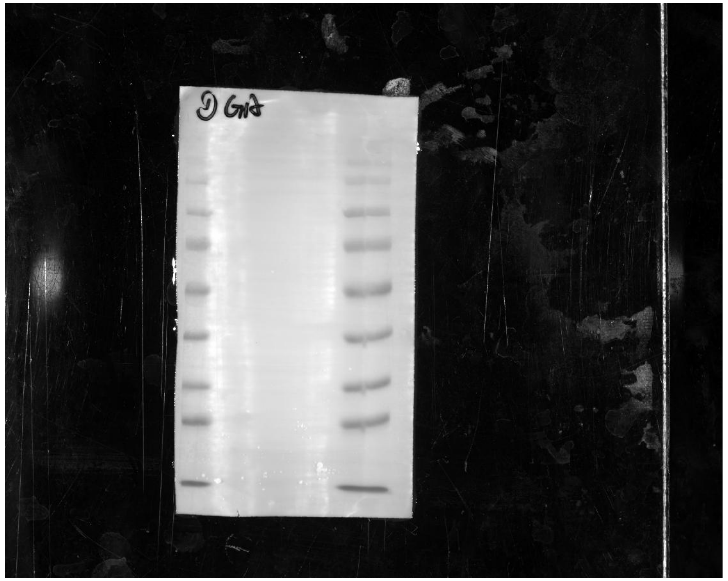

# 2H

H1299 Zo-1

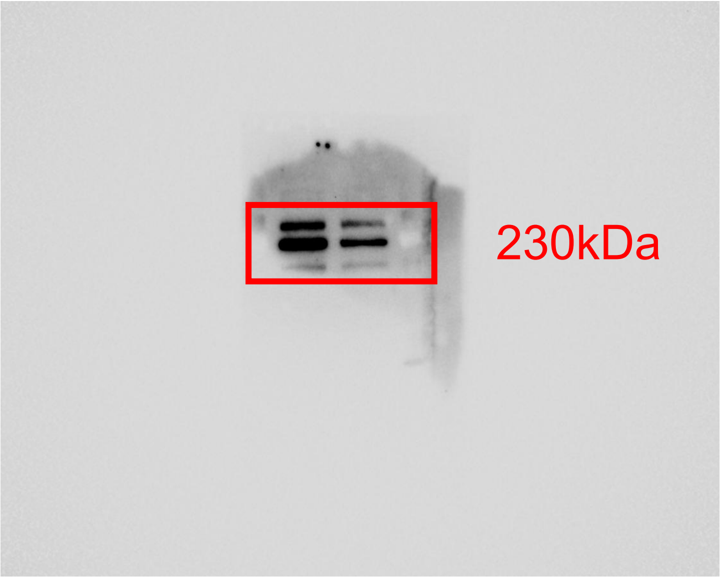


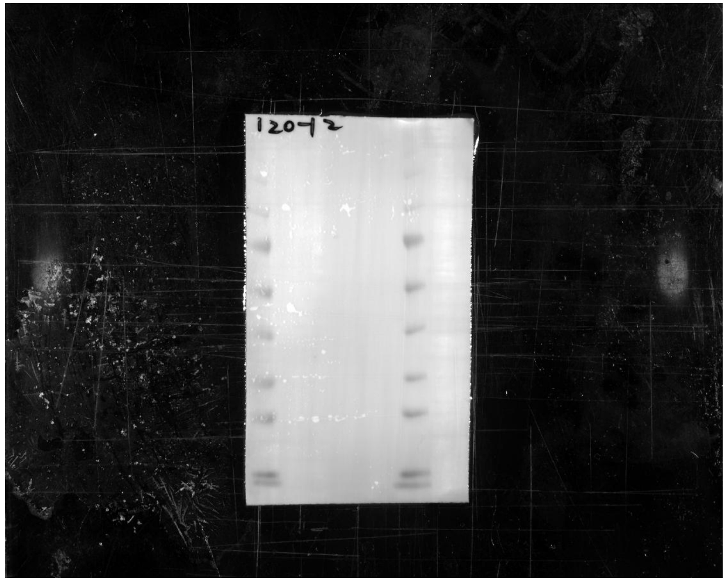

A549 Zo-1

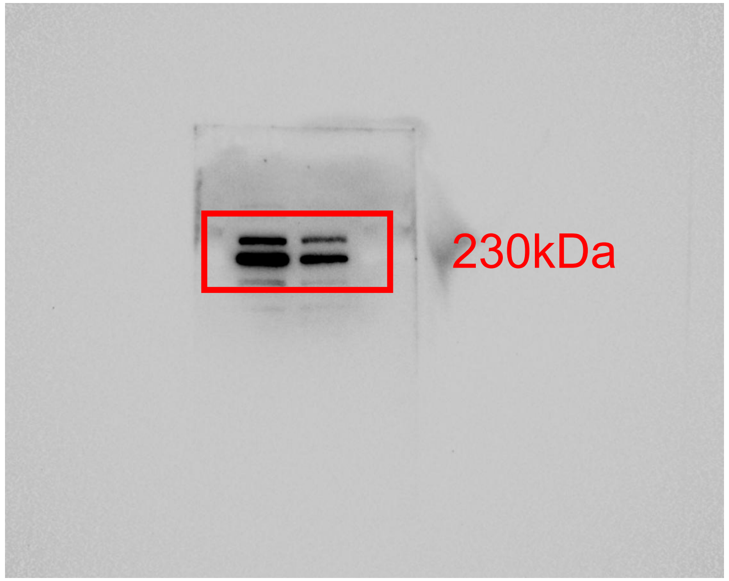


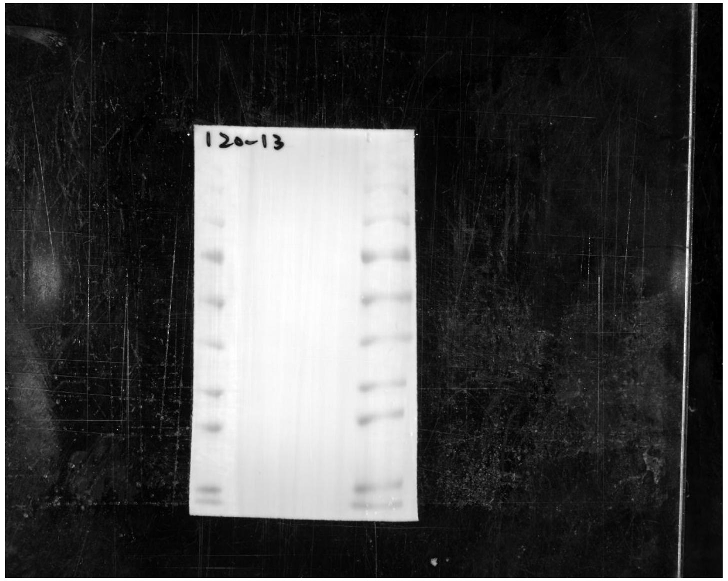

# 2H

H1299
vimentin

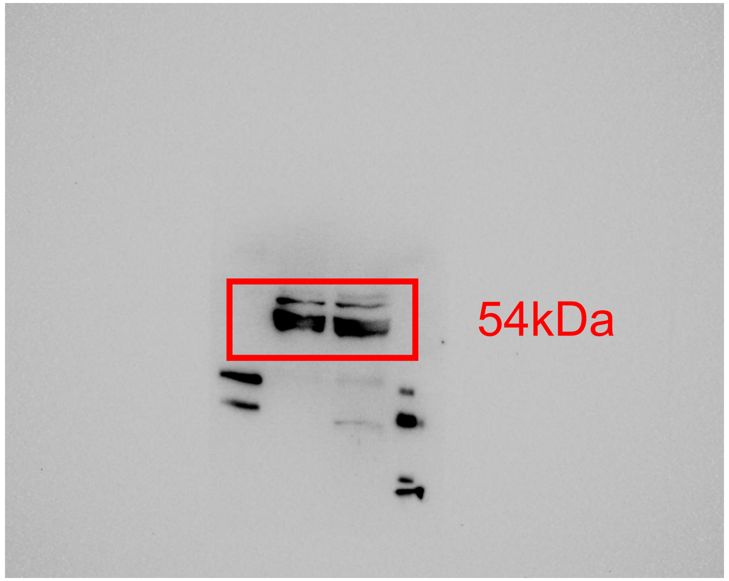


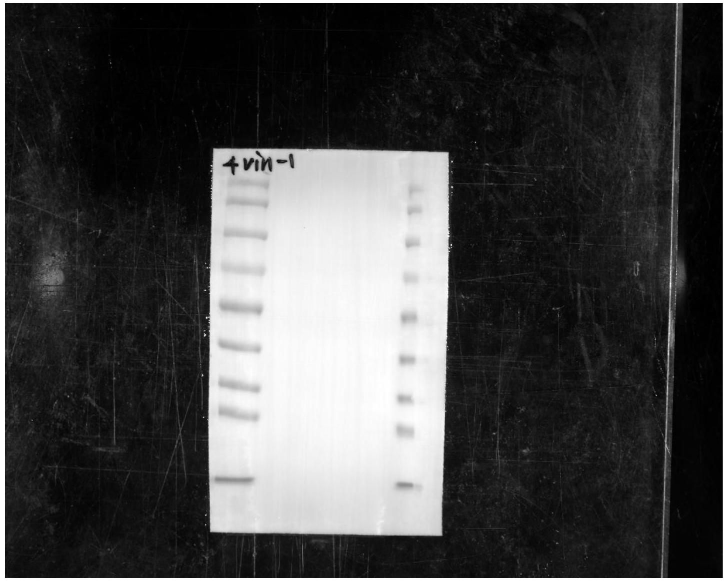


A549 vimentin

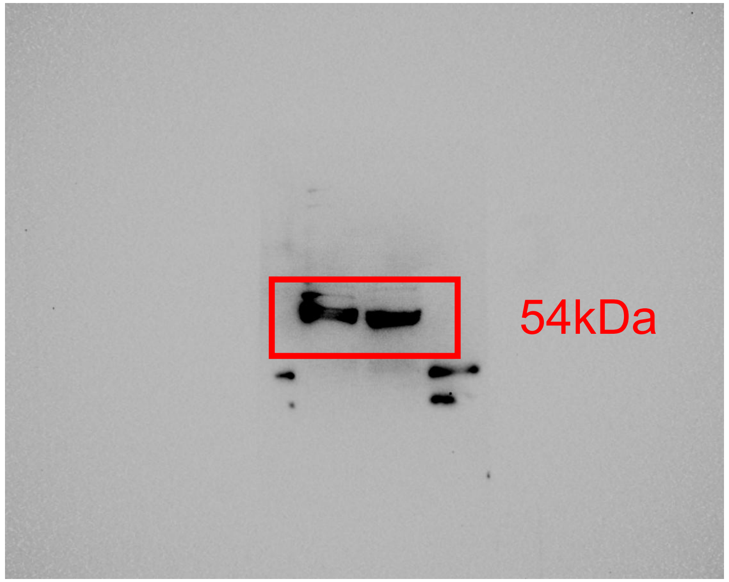


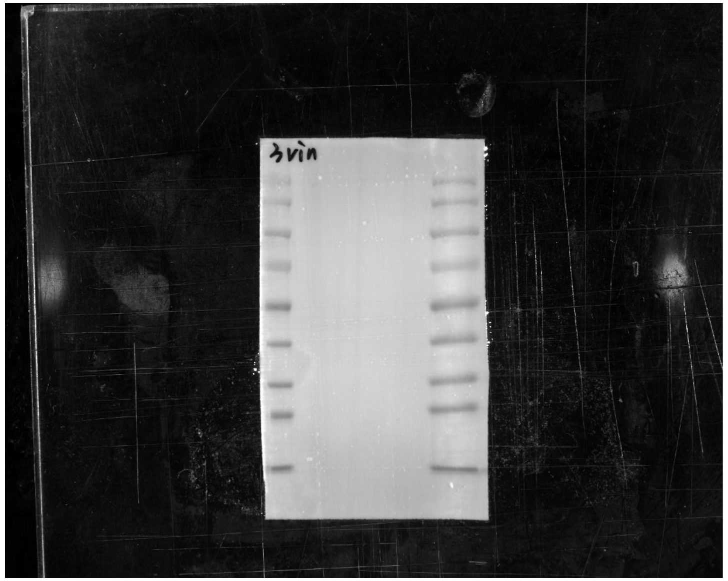

# 2H

H1299 mmp2

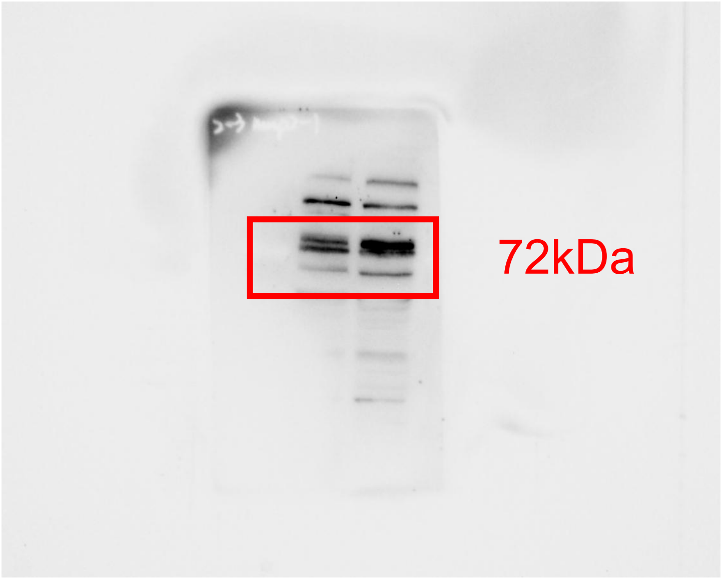


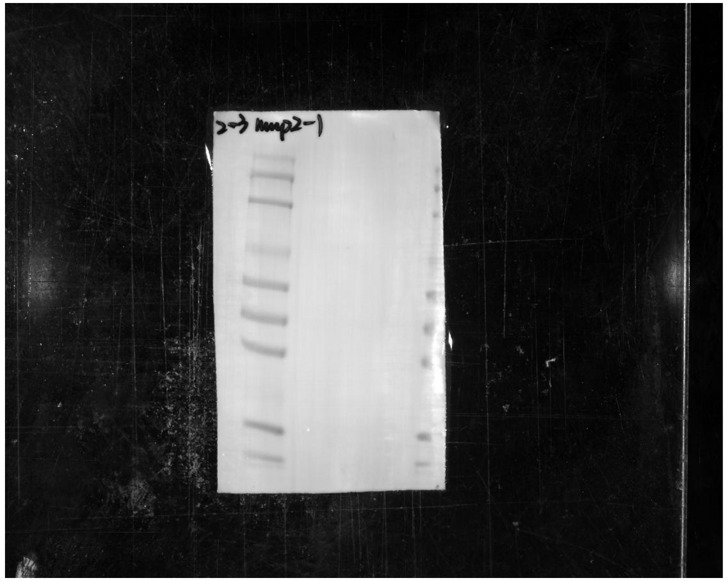

A549 mmp2

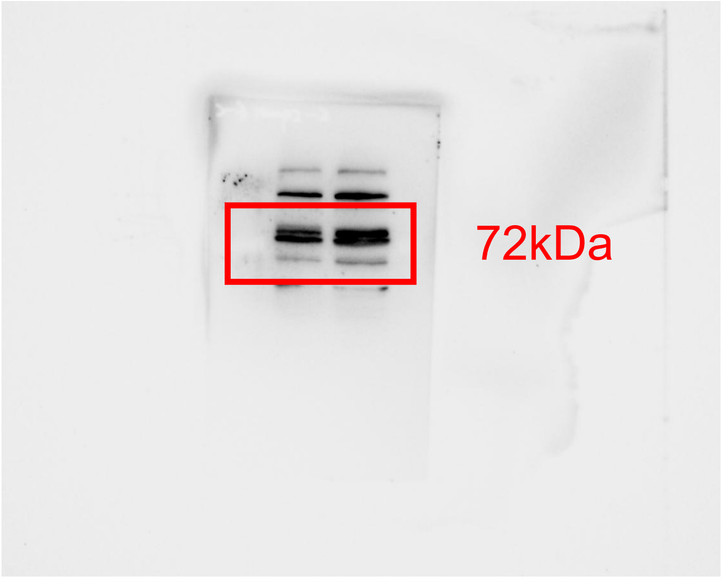


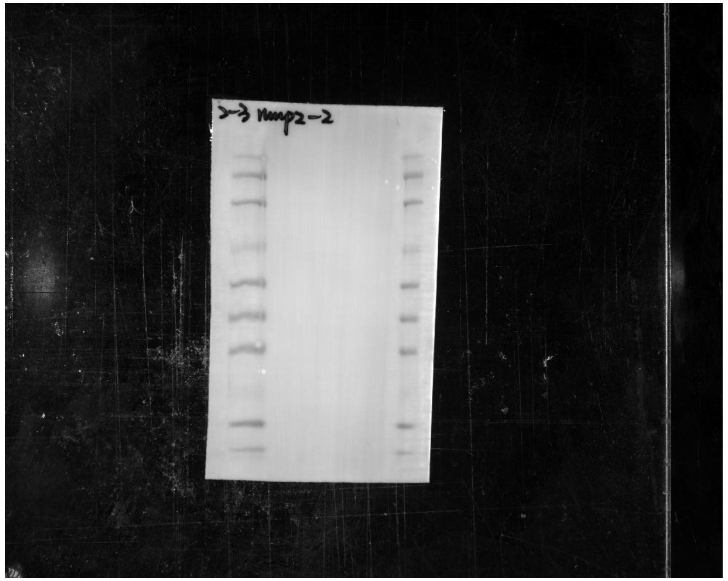

# S-Figure2F

cas9

H1299 CDK4

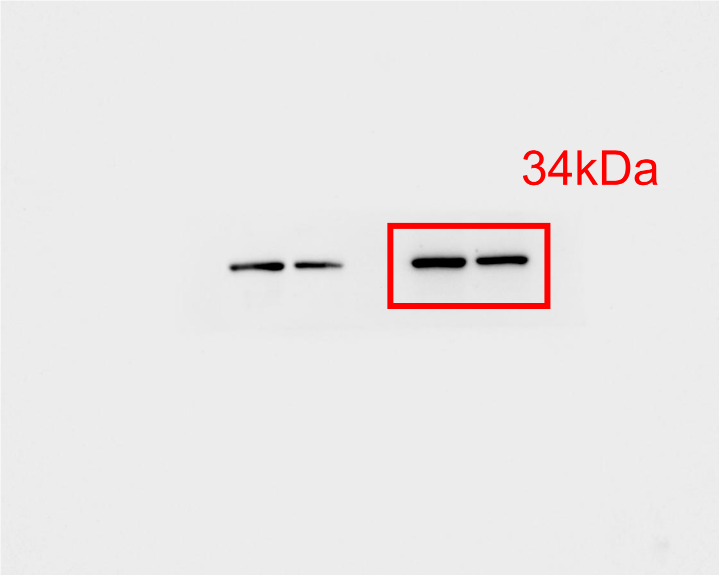


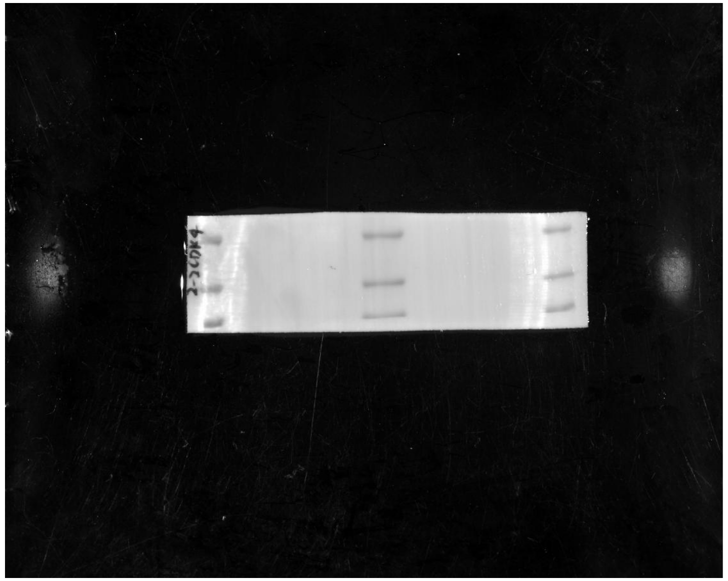

H1975 CDK4

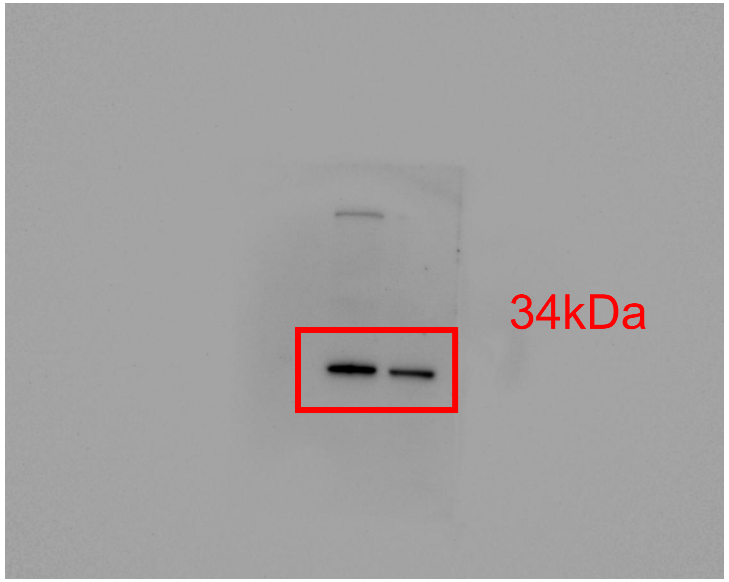


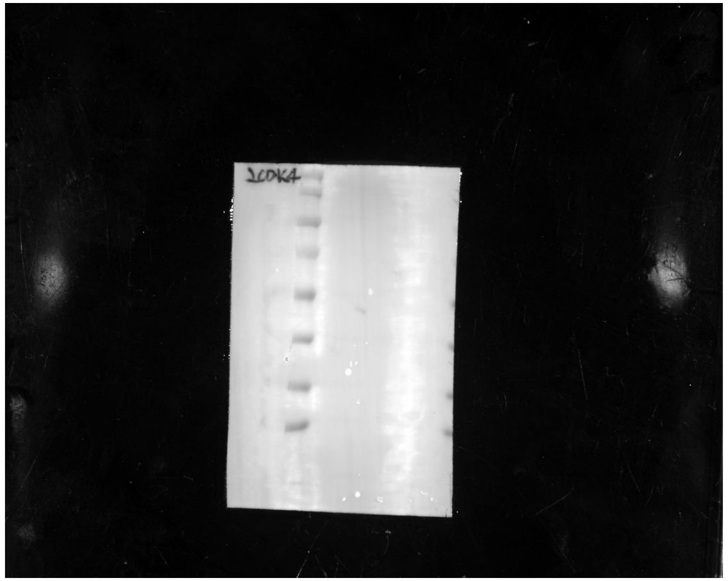

cas9

H1299 CDK6

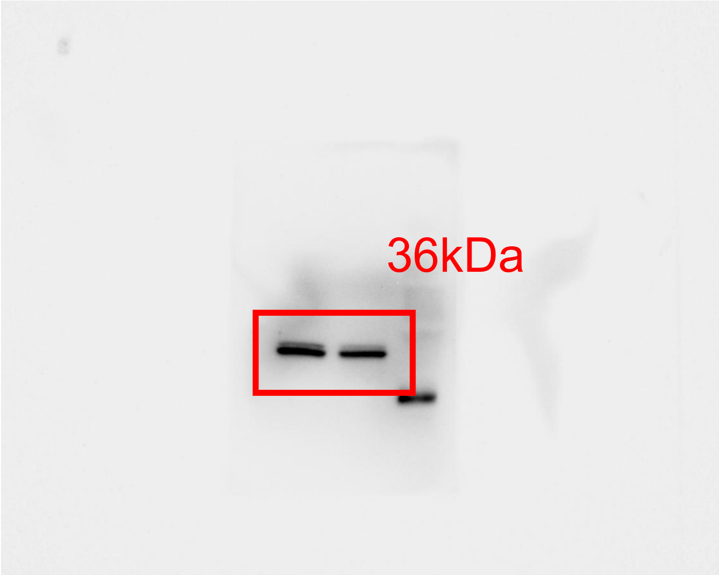


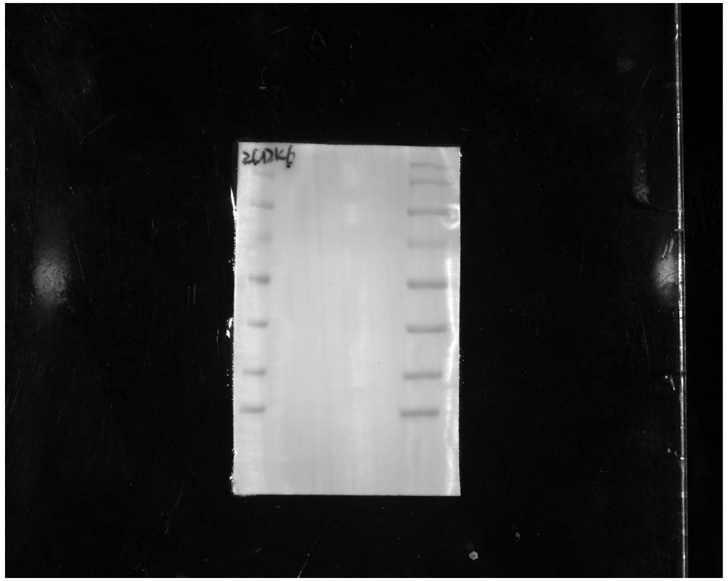

H1975 CDK6

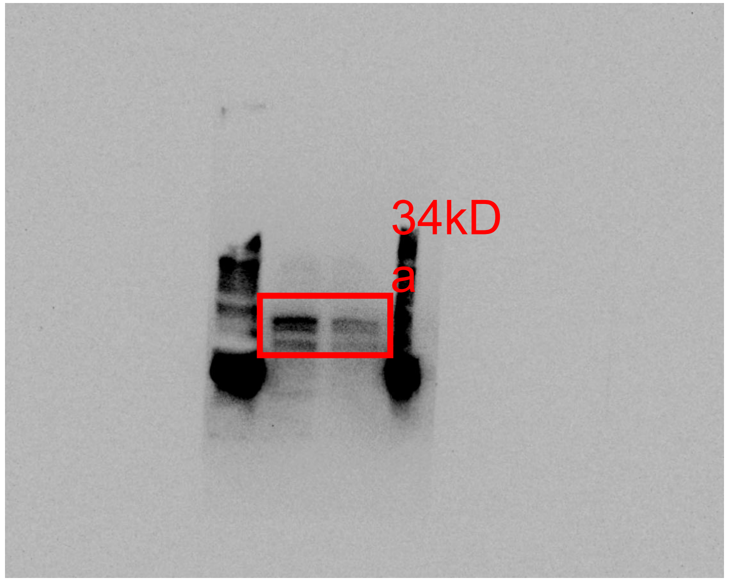


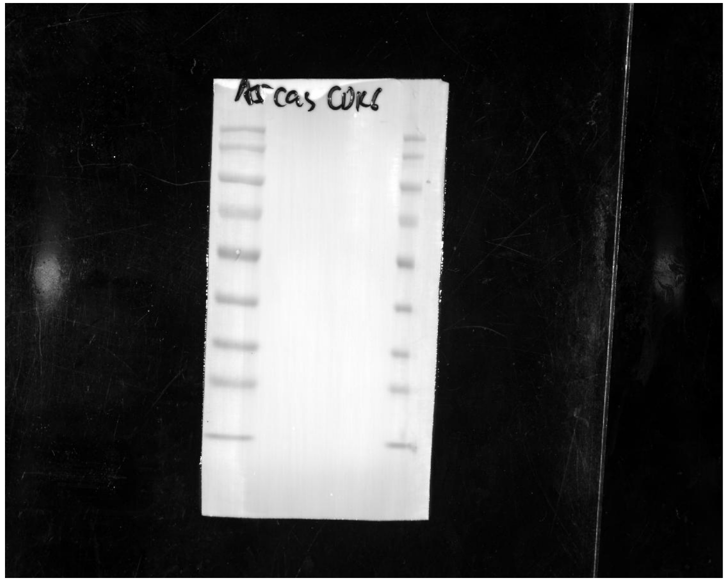

# cas9

H1299 A2

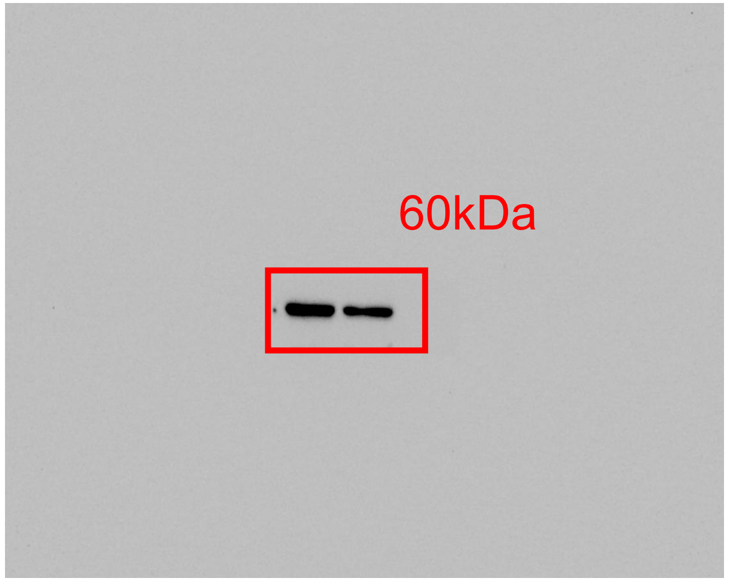


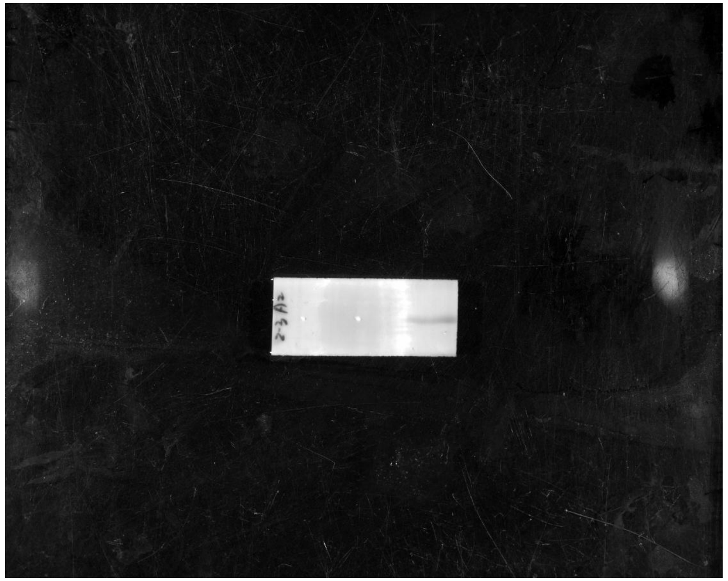

H1975 A2

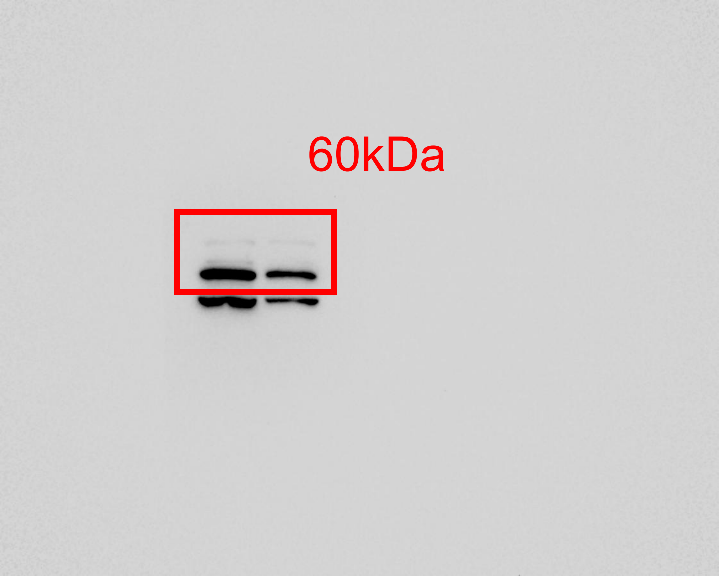


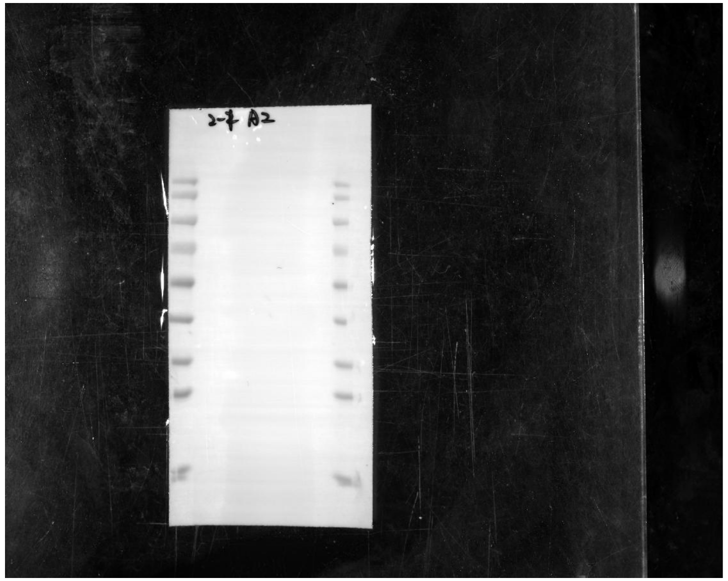

# cas9

H1299 D1

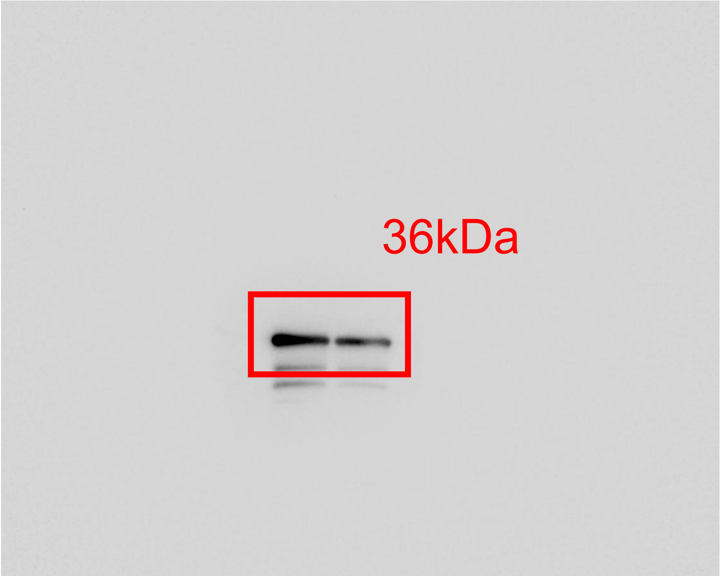


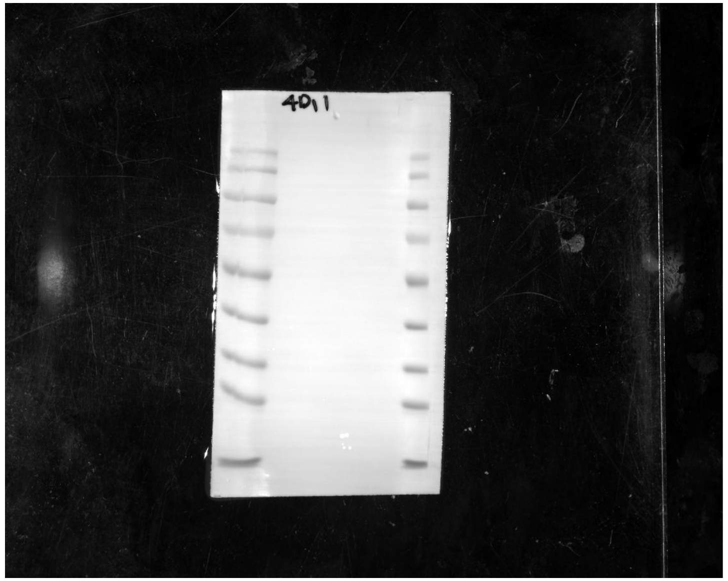

H1975 D1

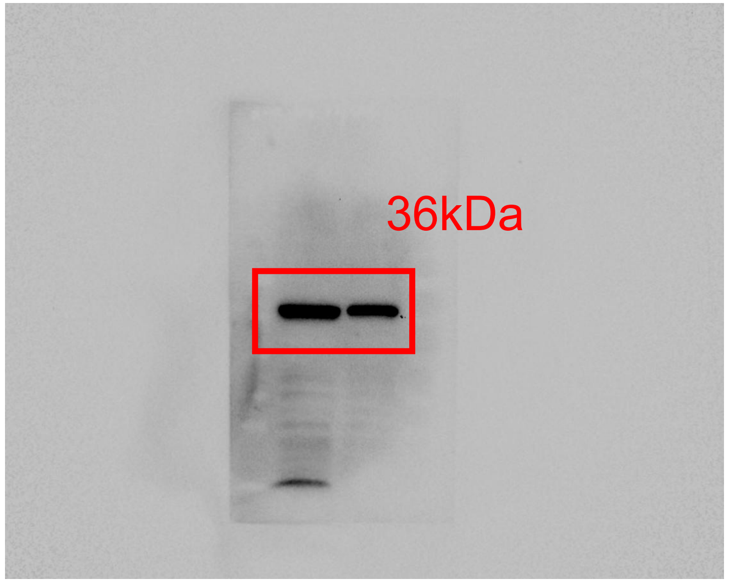


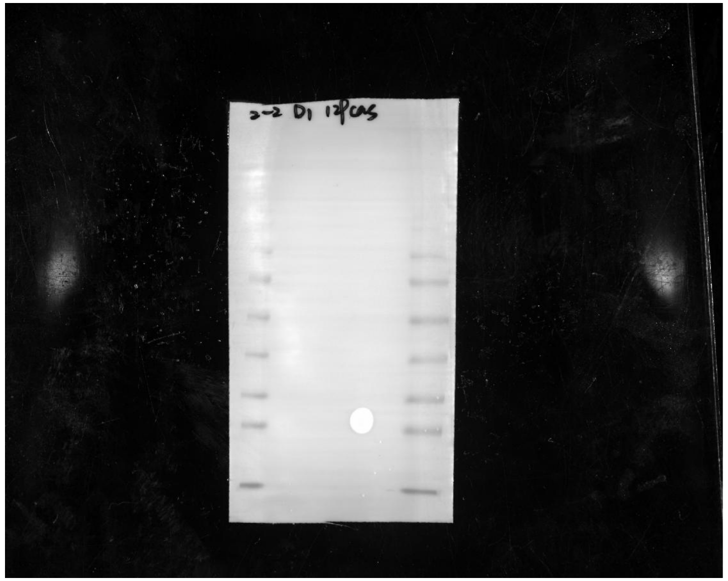

cas9

H1299 E-ca

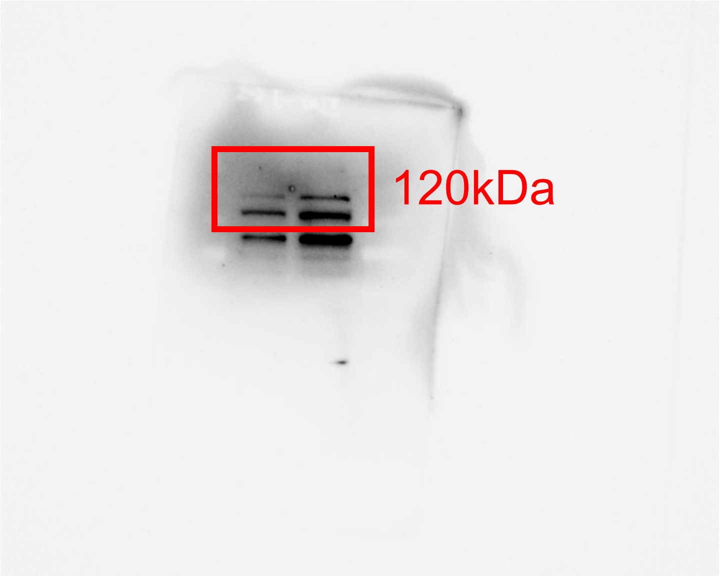

120kDa

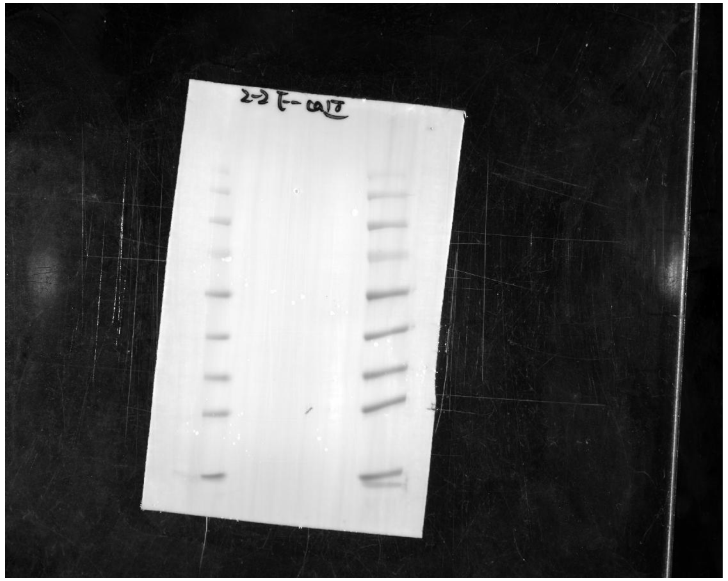

H1975 E-ca

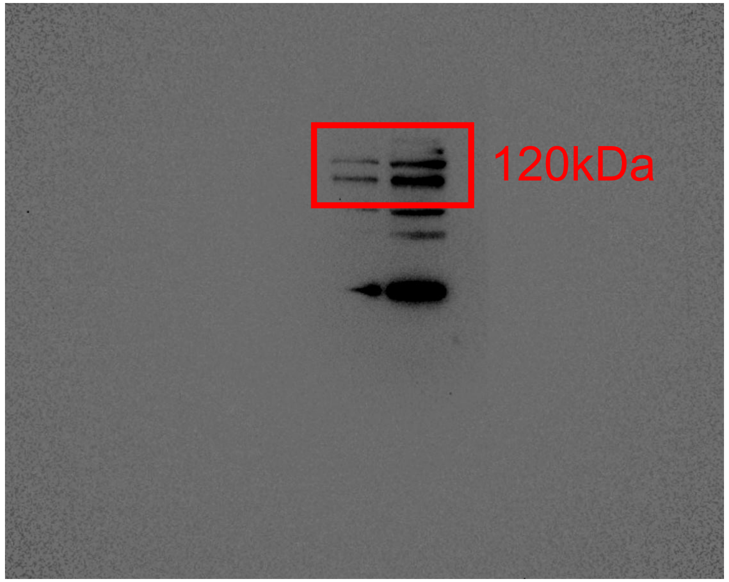

120kDa

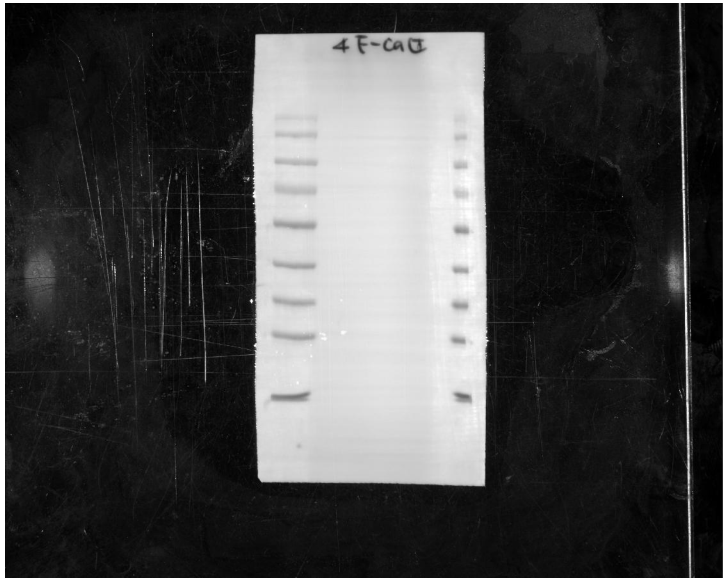

# cas9

H1299 N-ca

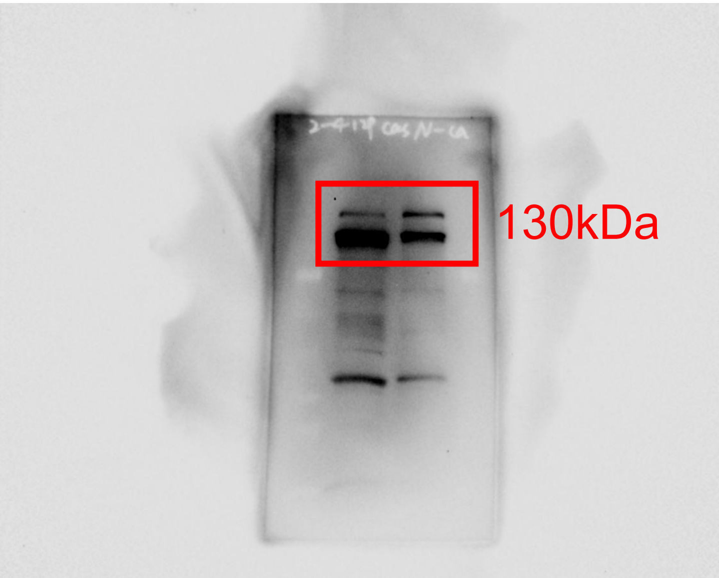


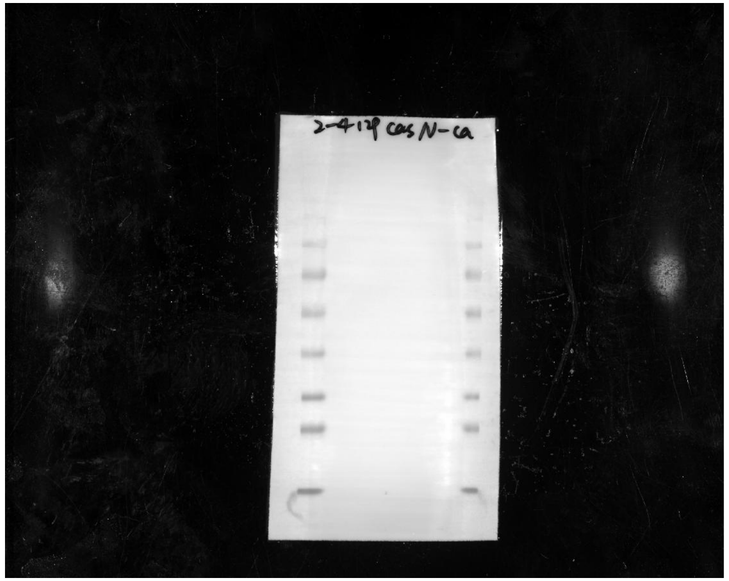

H1975 N-ca

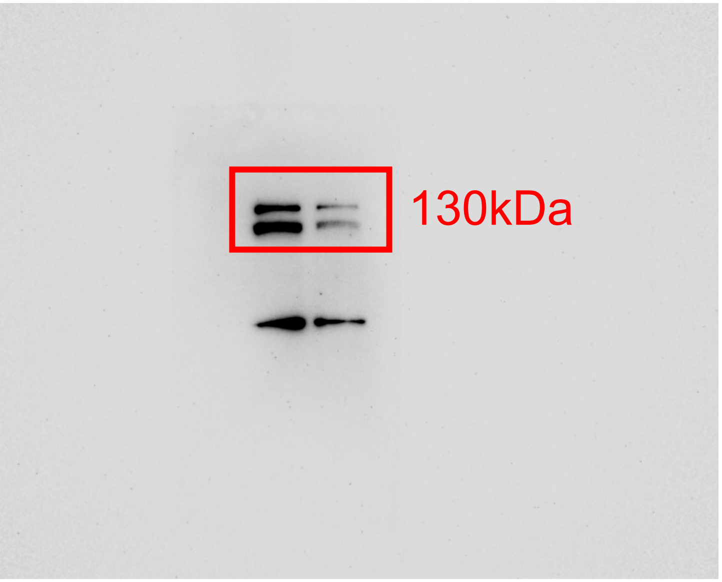


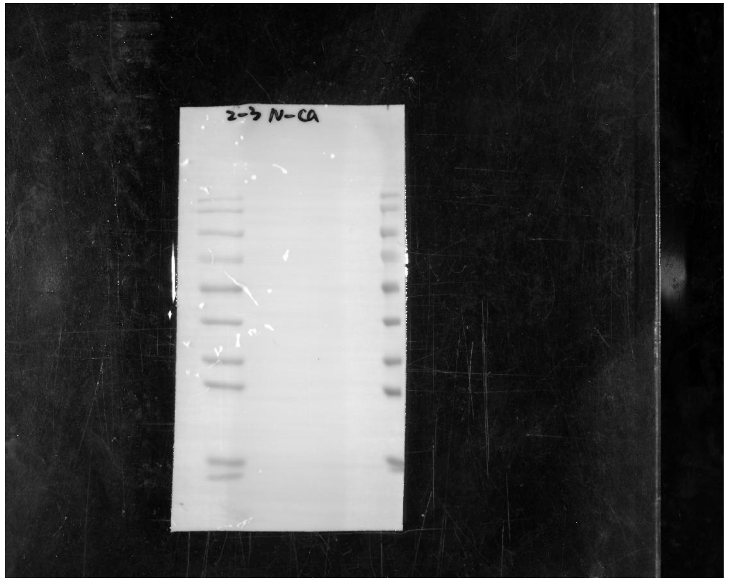

# cas9

H1299 mmp9

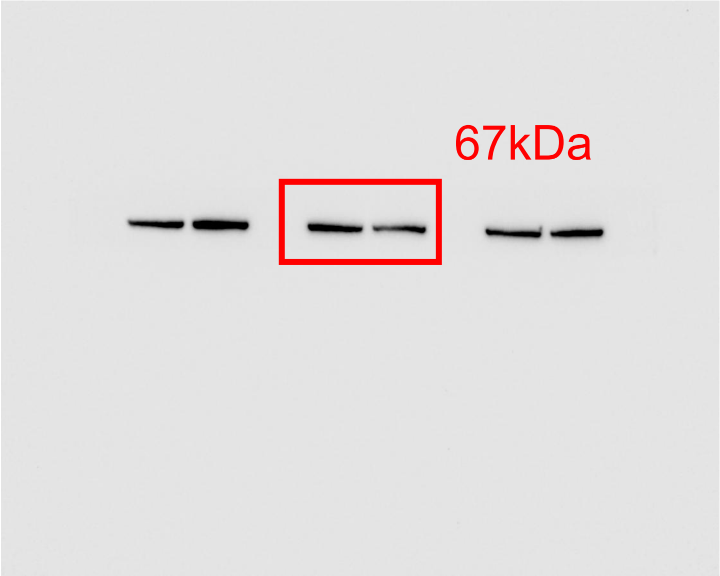


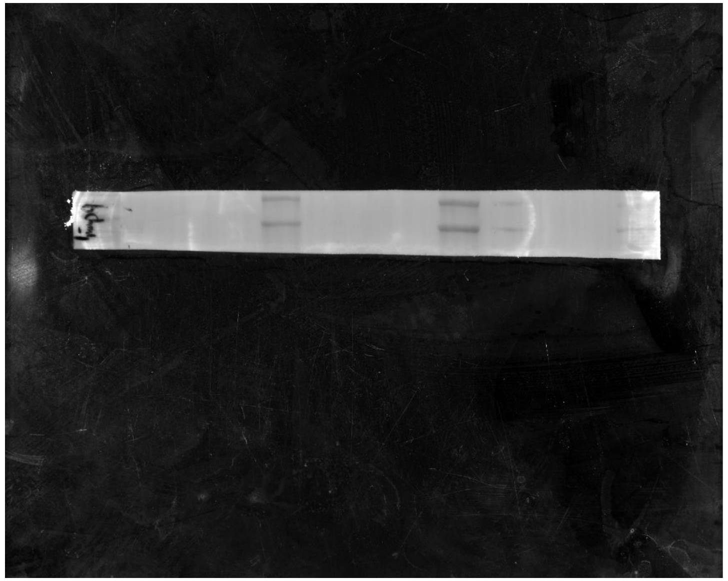

H1975 mmp9

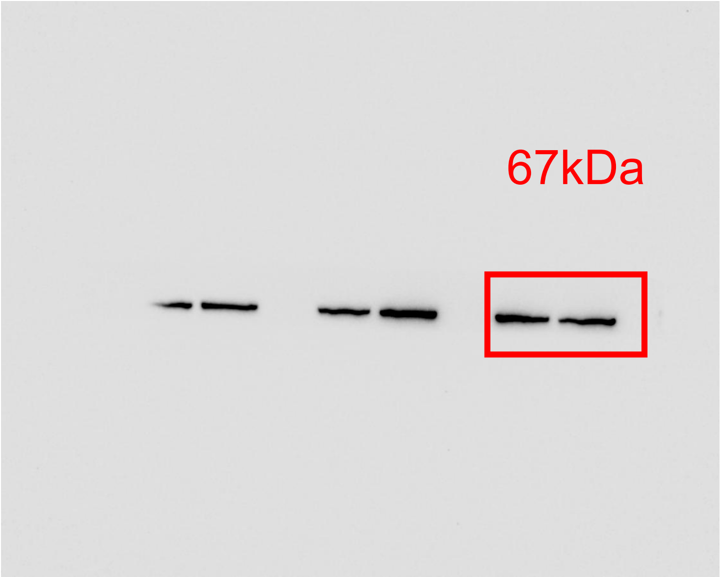


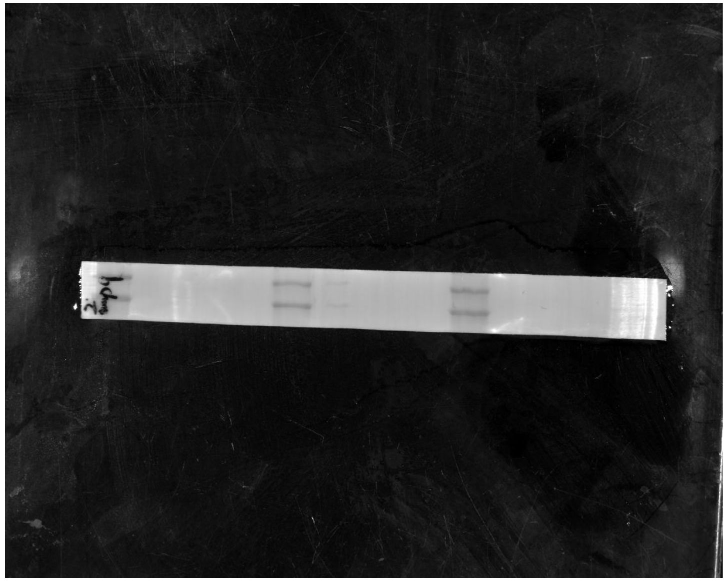

# cas9

H1299cas9GAPDH

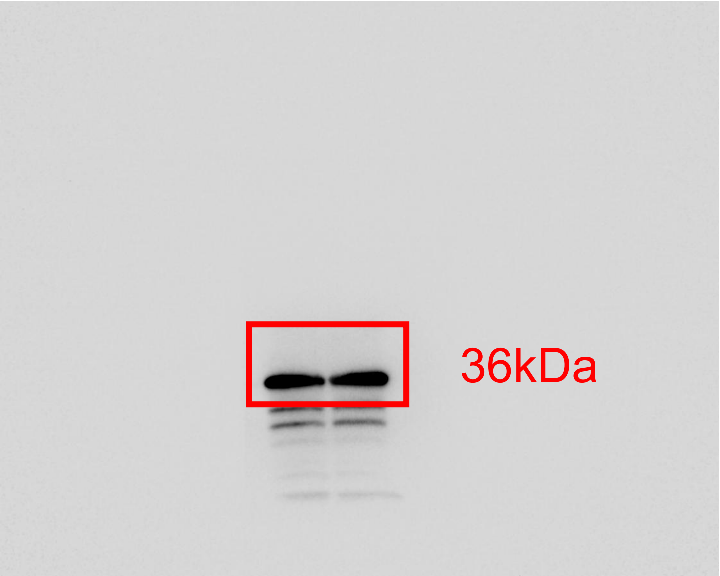


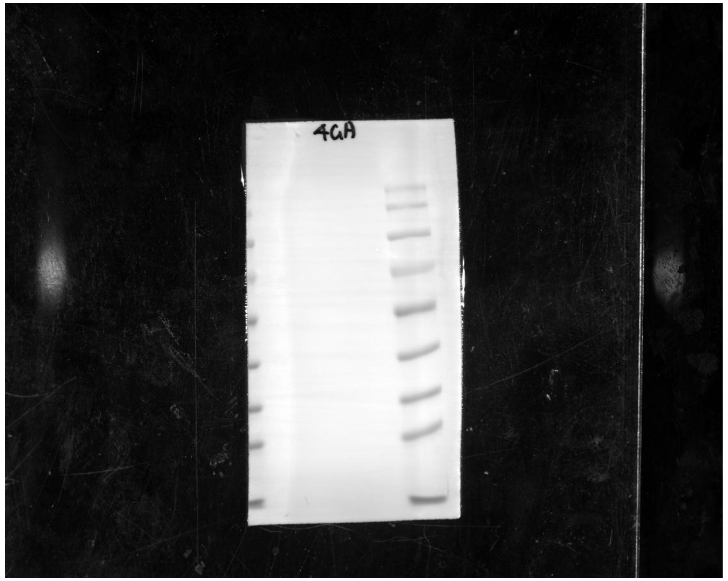

H1975cas9GAPDH

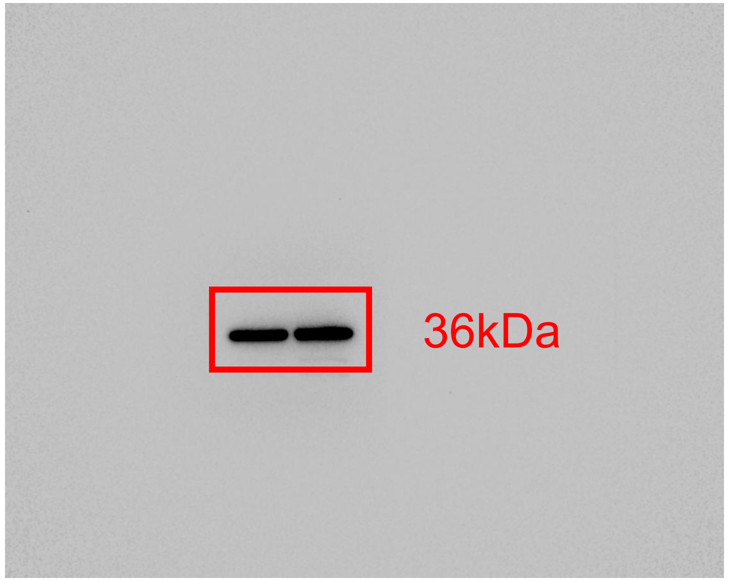


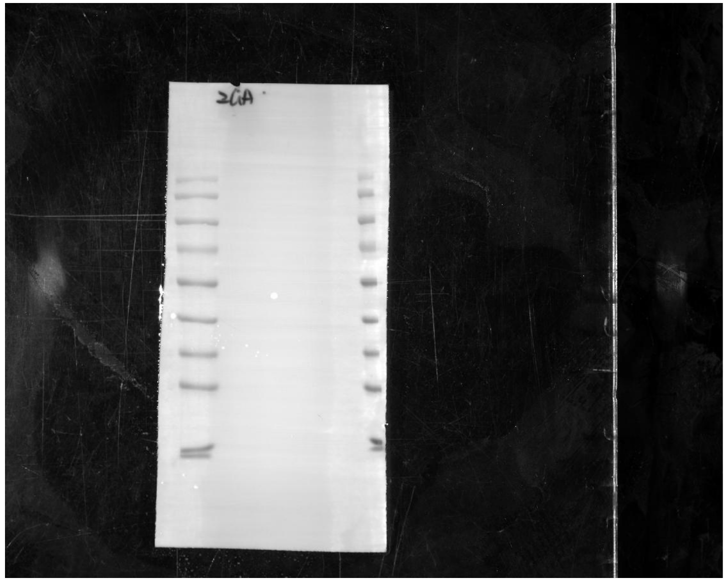

# S-2F

H1299 Zo-1

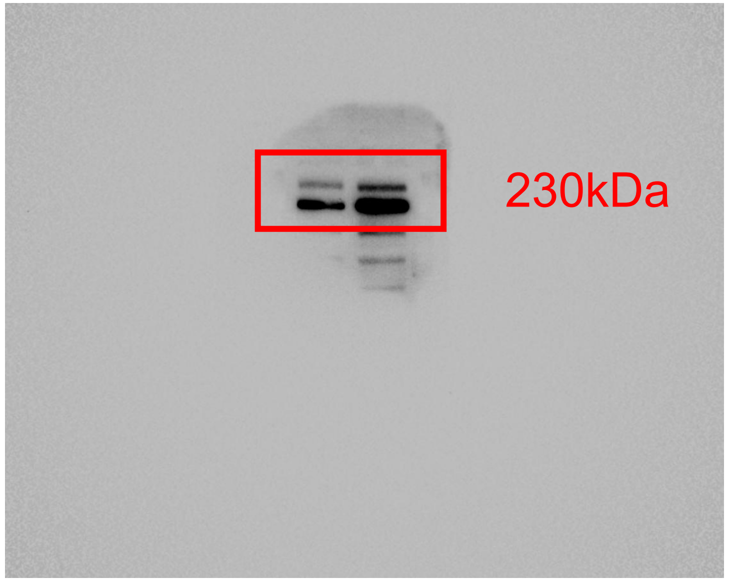


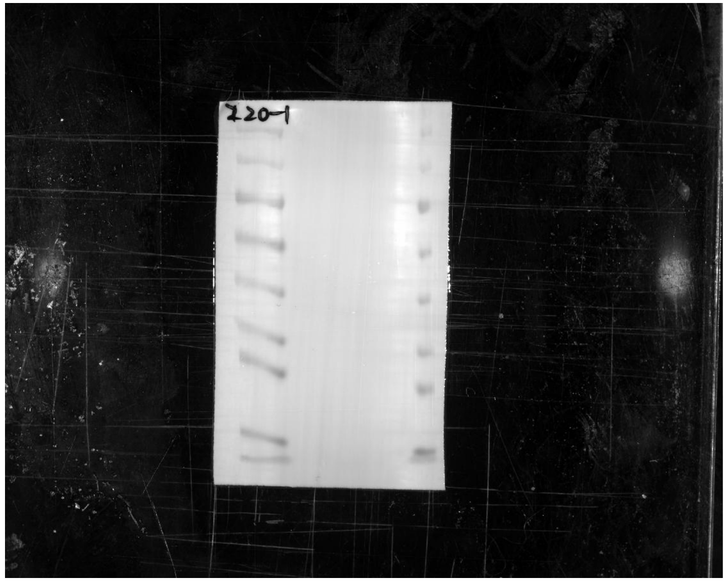

H1975 Zo-1

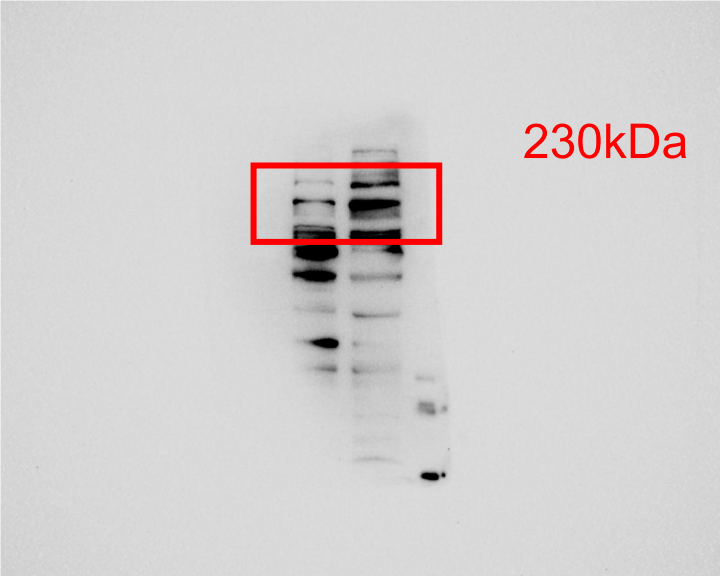


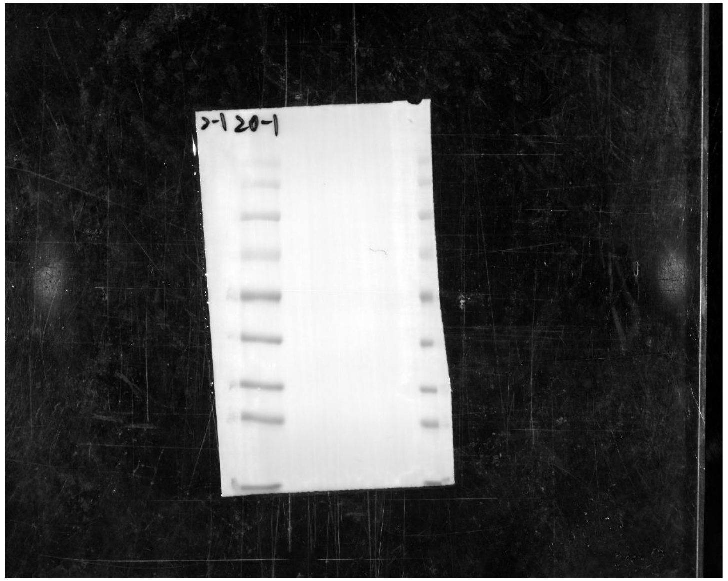

H1299
vimentin

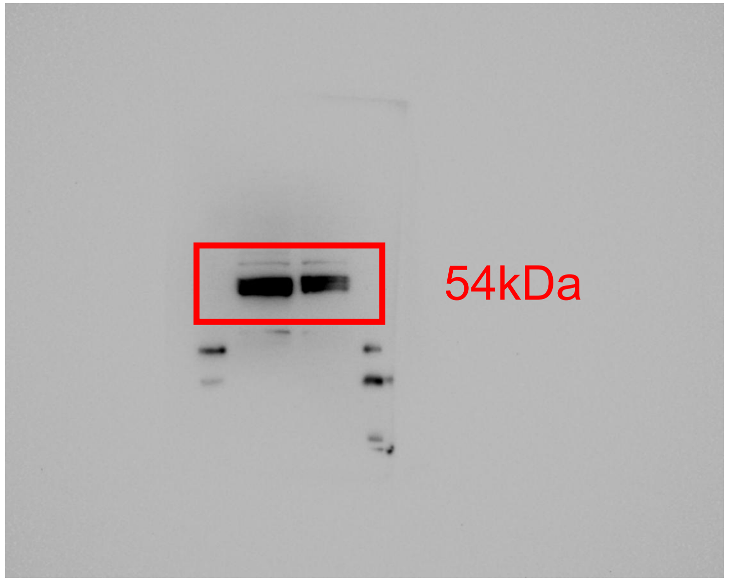


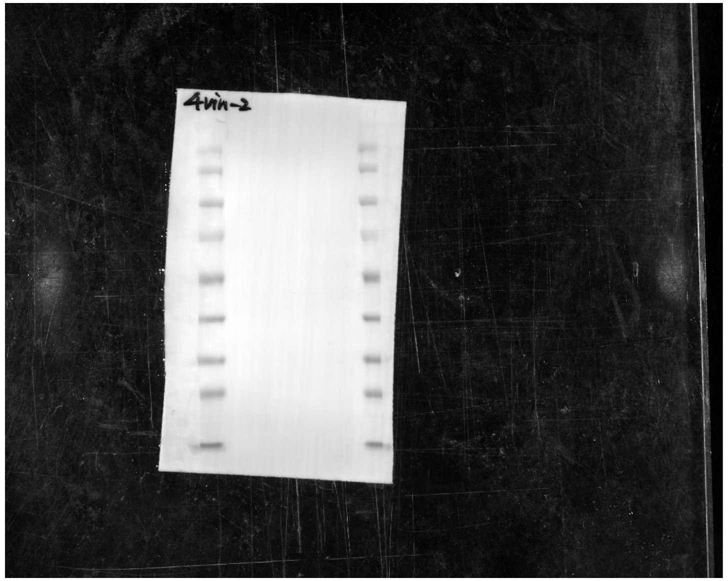

H1975
vimentin

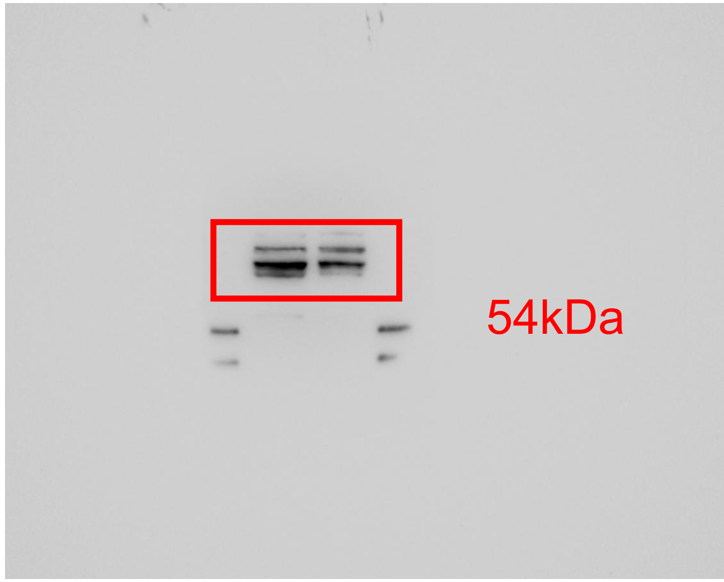


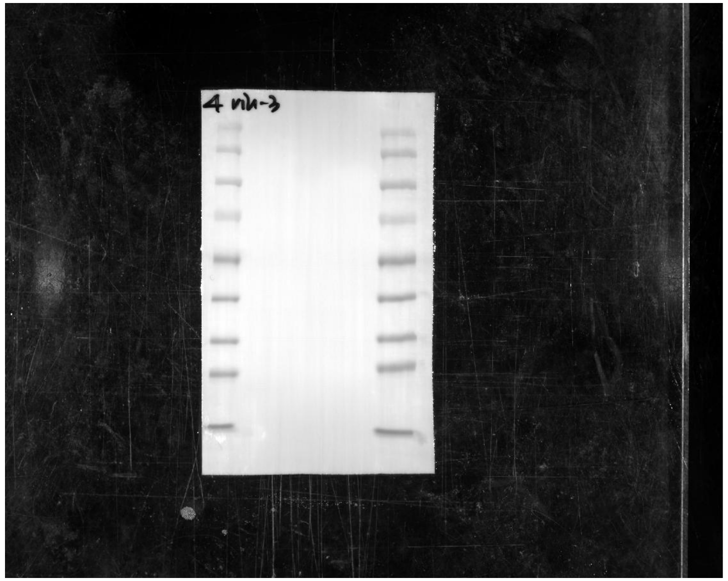

H1299 mmp2

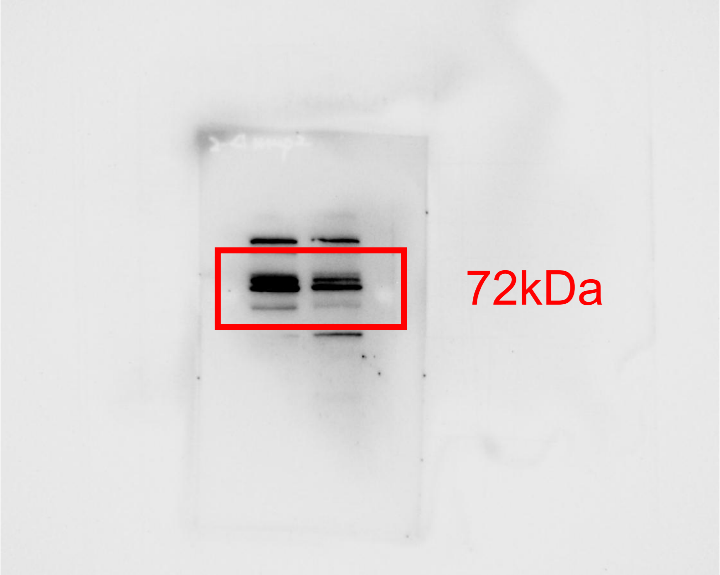


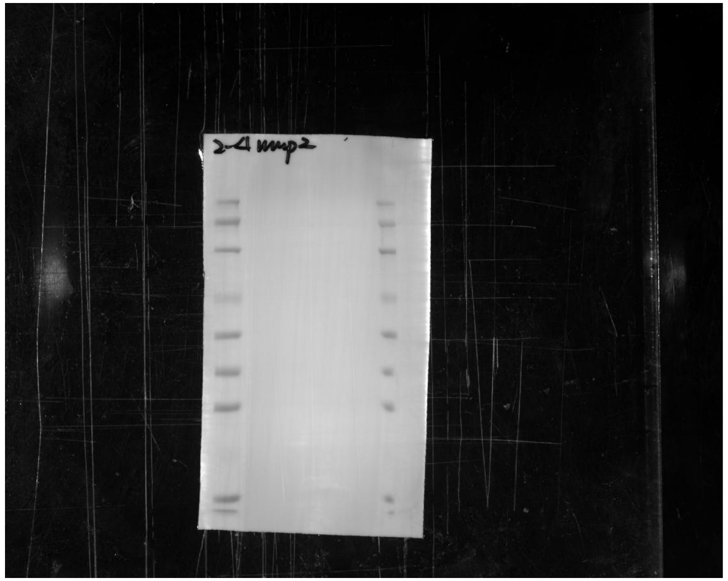

H1975 mmp2

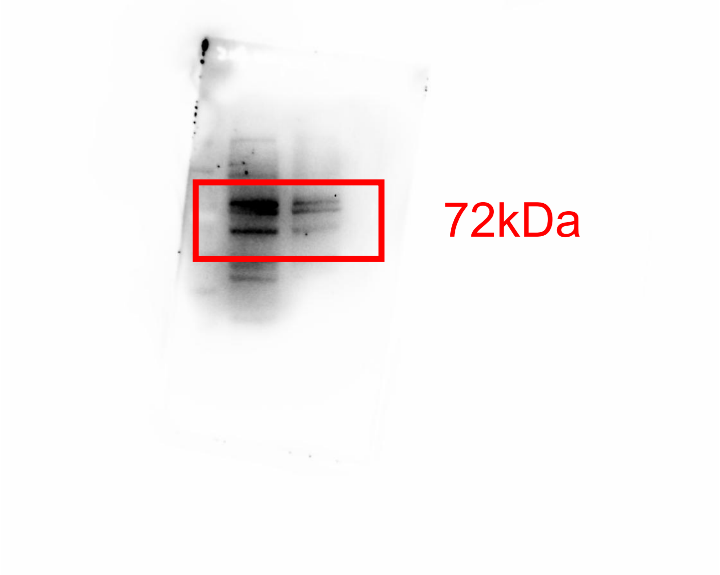


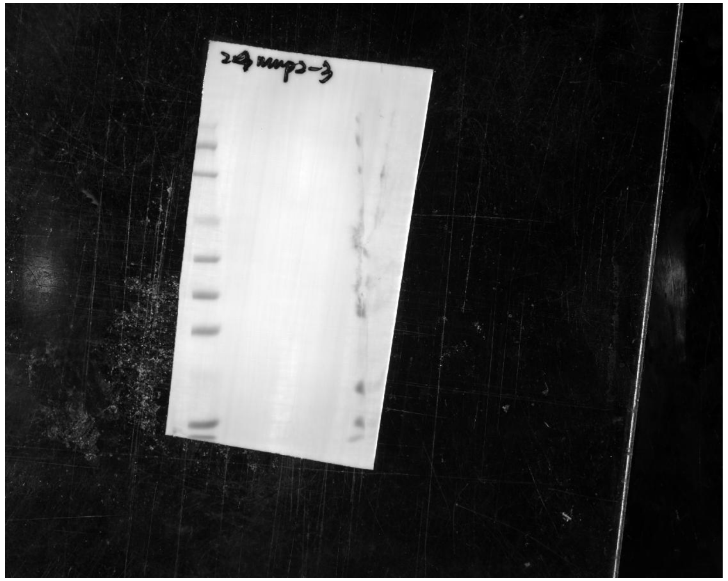

# Figure3D

H1299PHF23

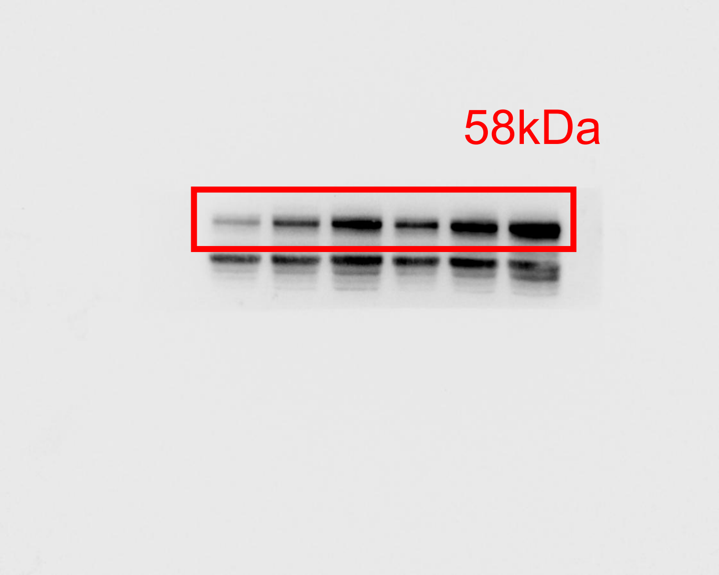


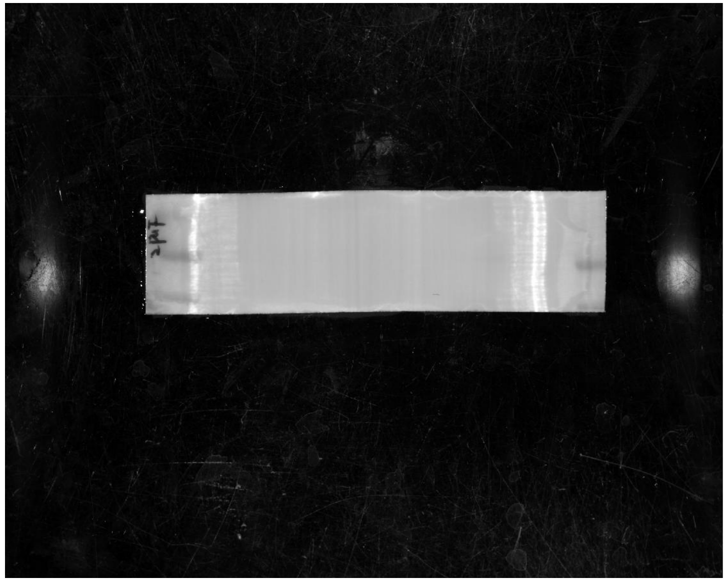

H1299GAPDH

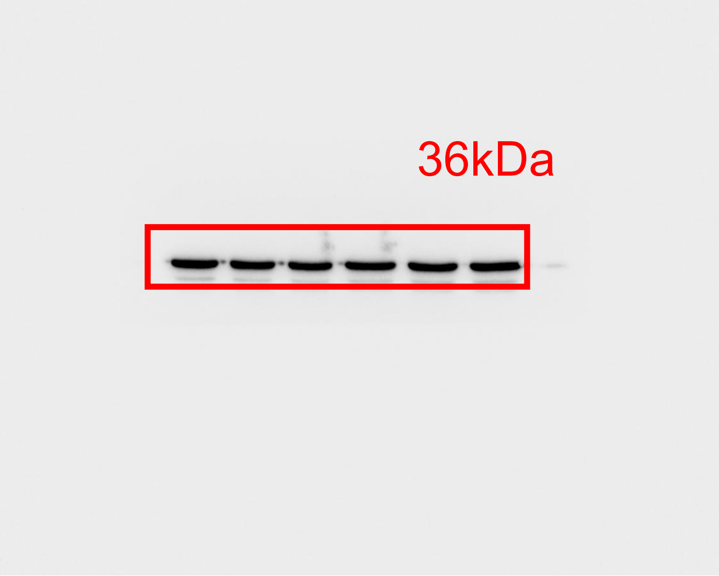


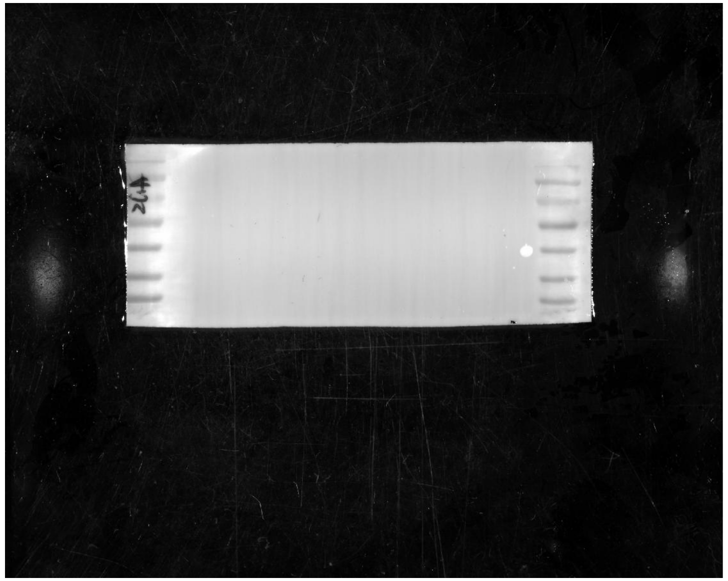

A549PHF23

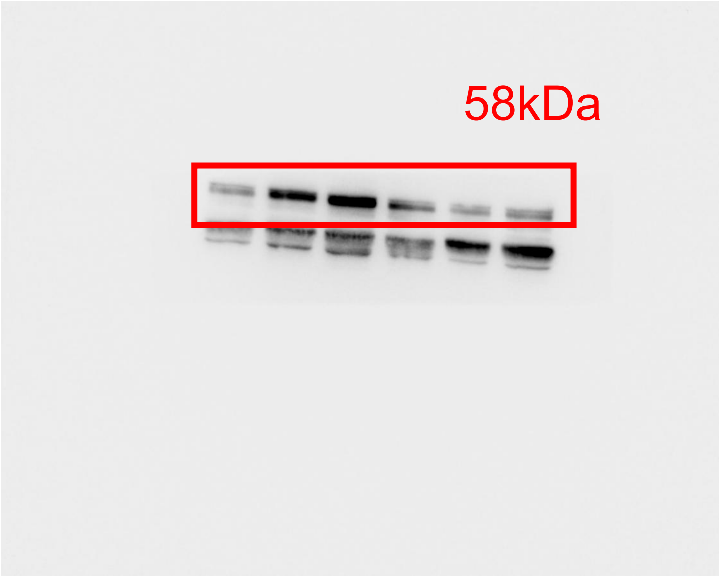


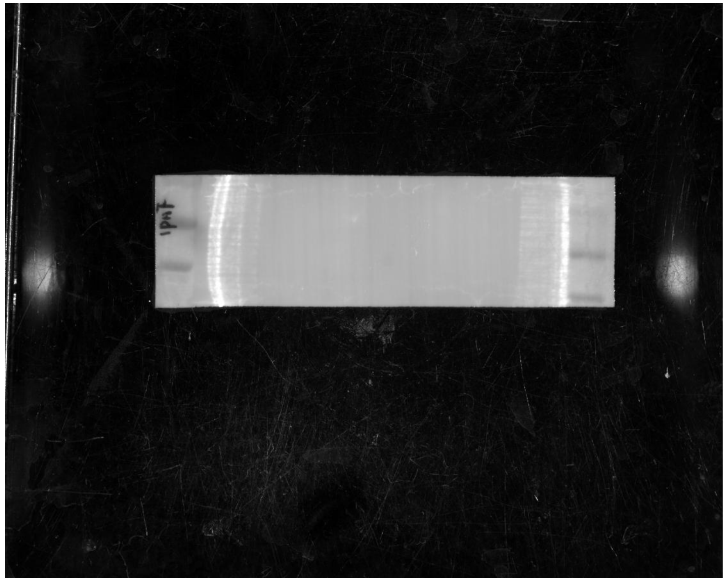

A549GAPDH

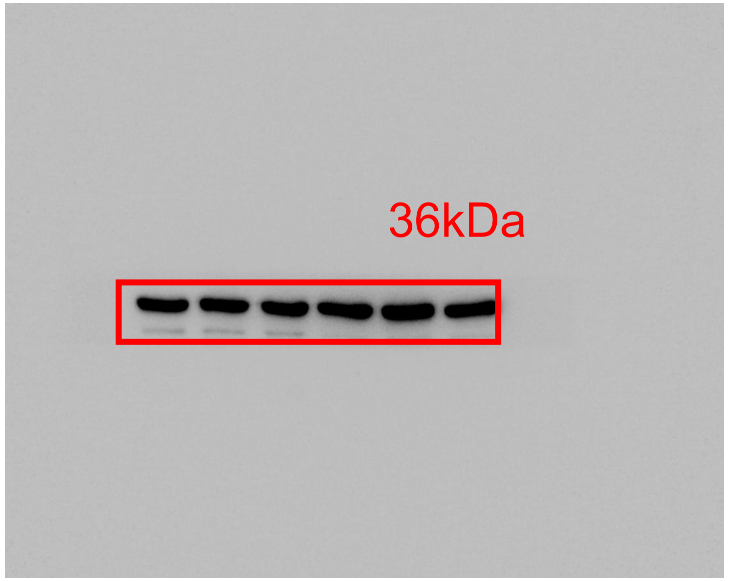


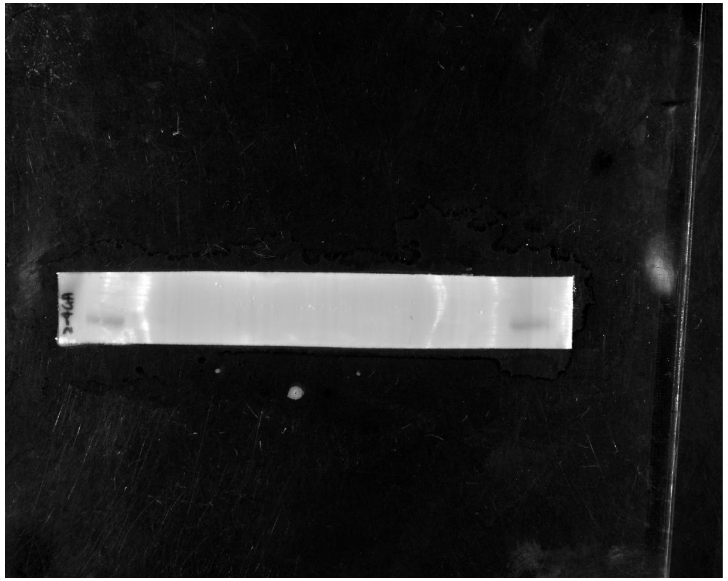

# Figure3E

H1299PHF23

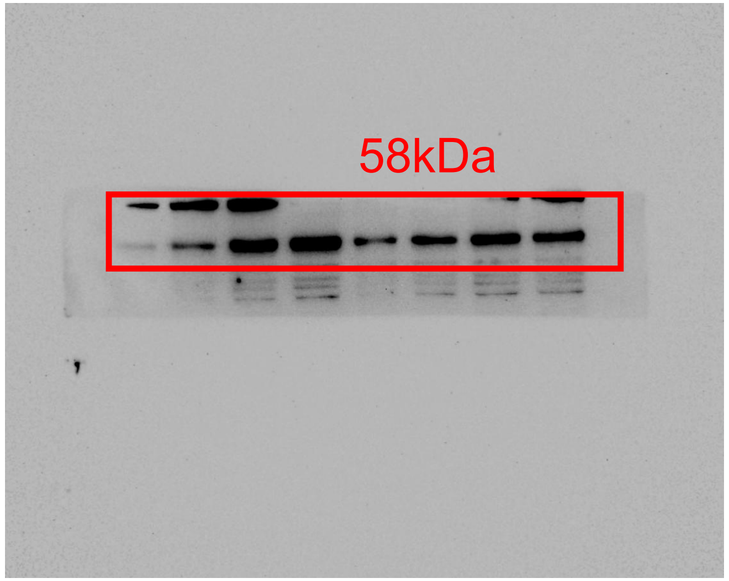


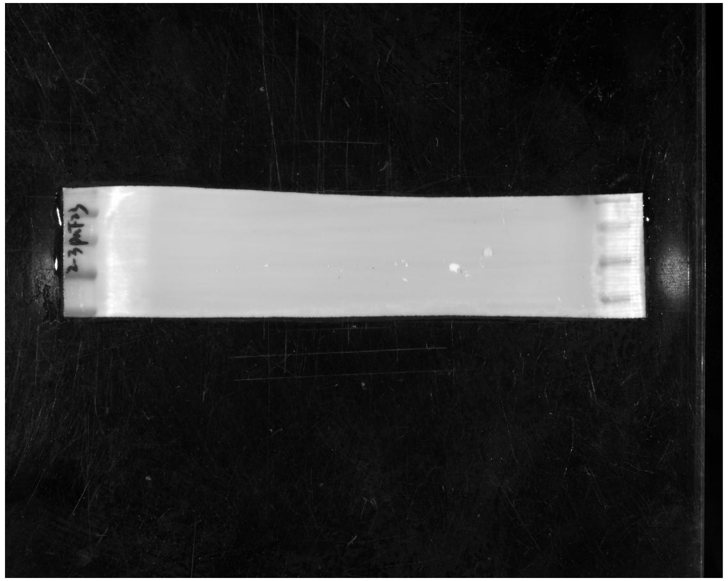

H1299GAPDH

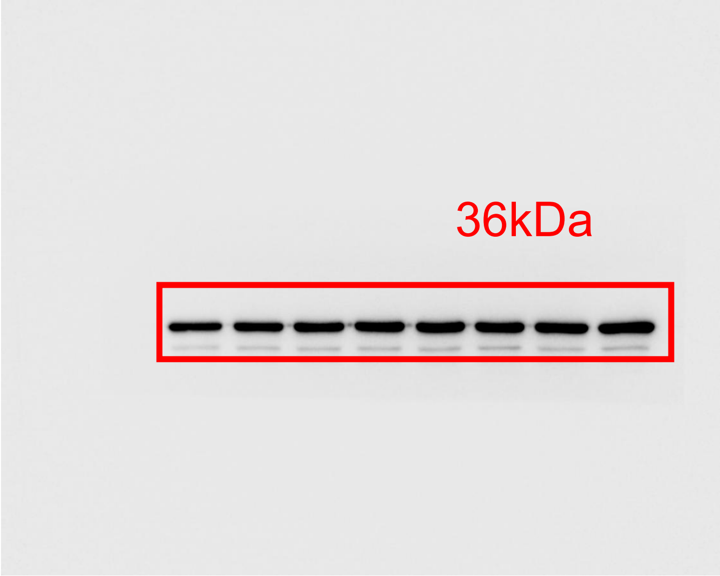


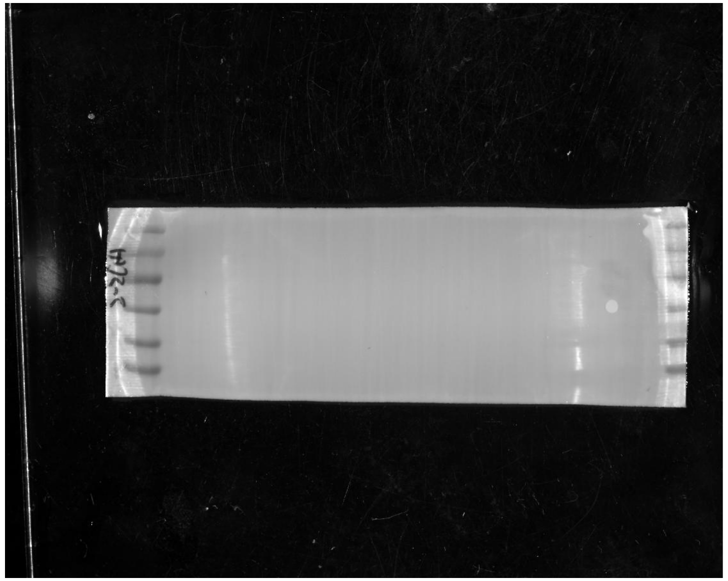

A549PHF23

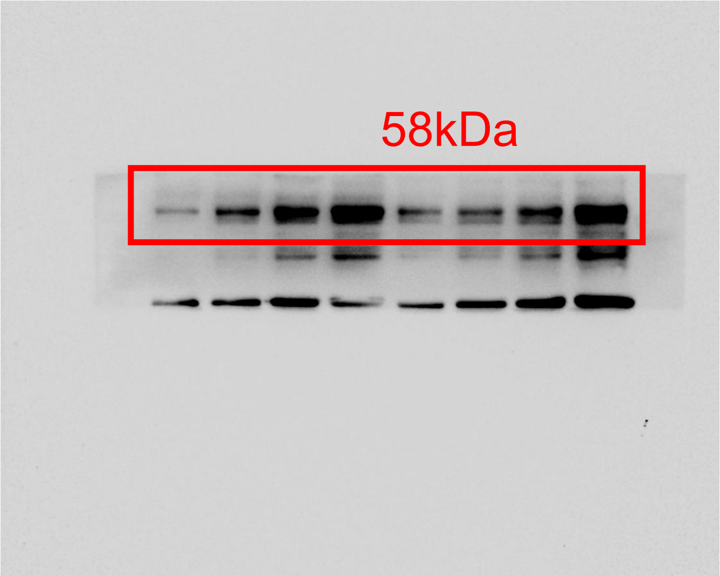


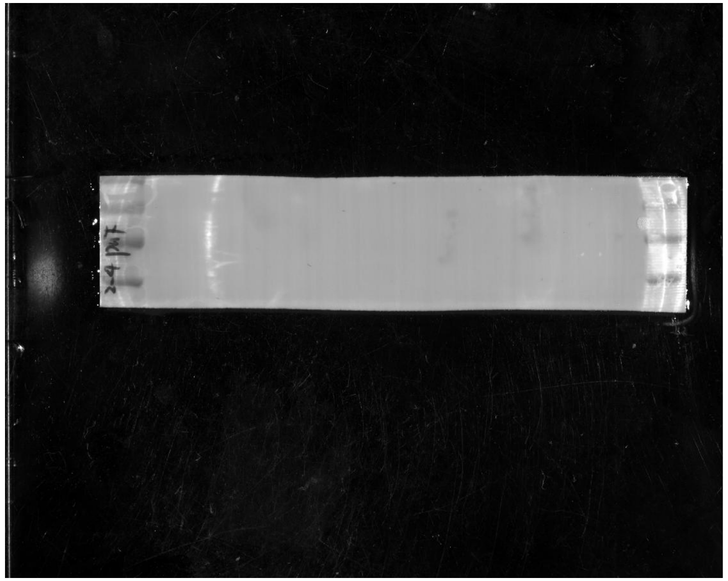

A549GAPDH

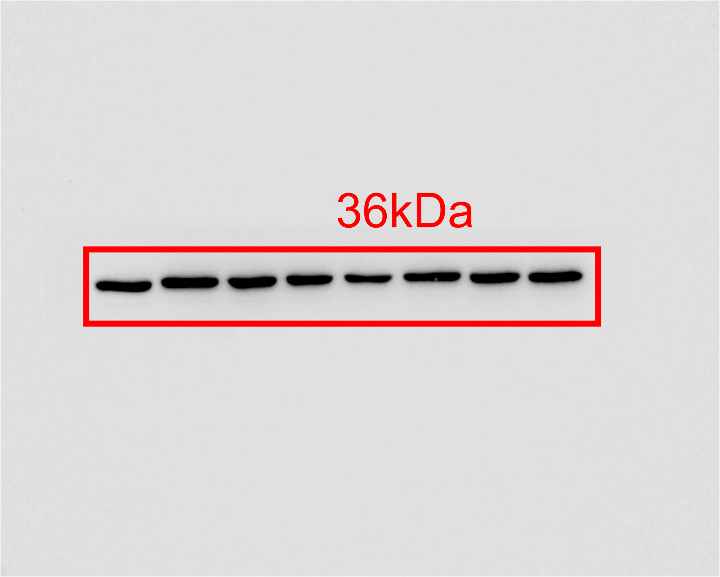


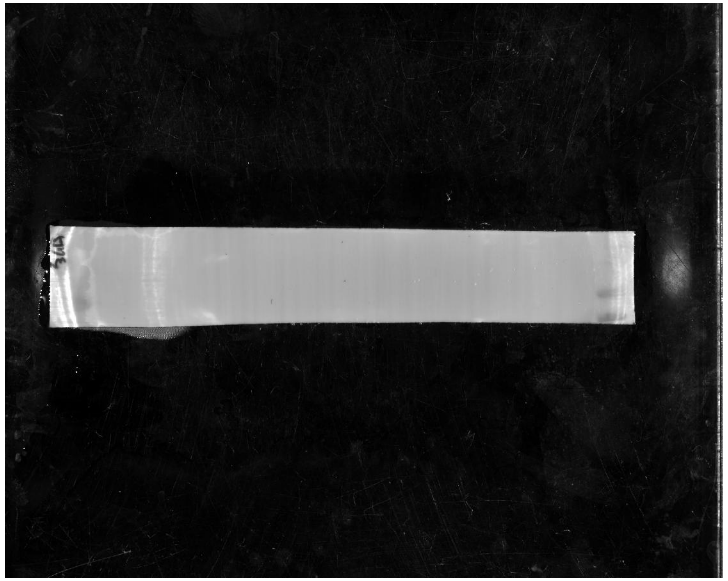

# S-Figure3A

PHF23

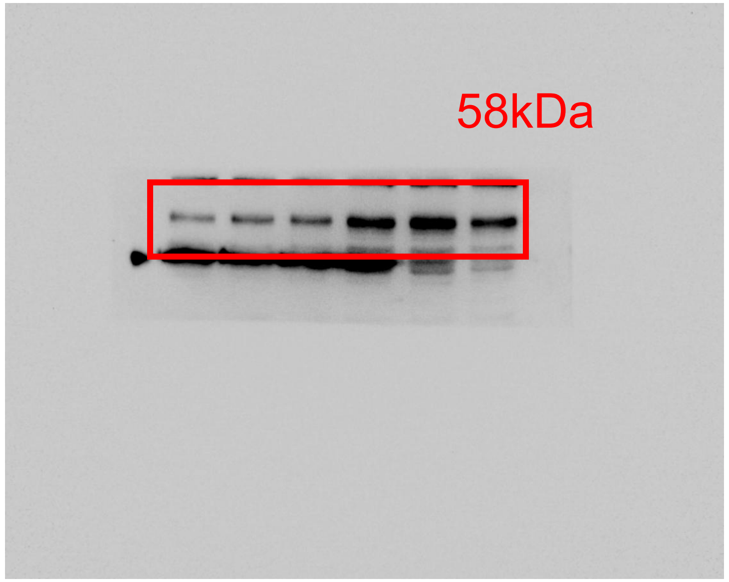


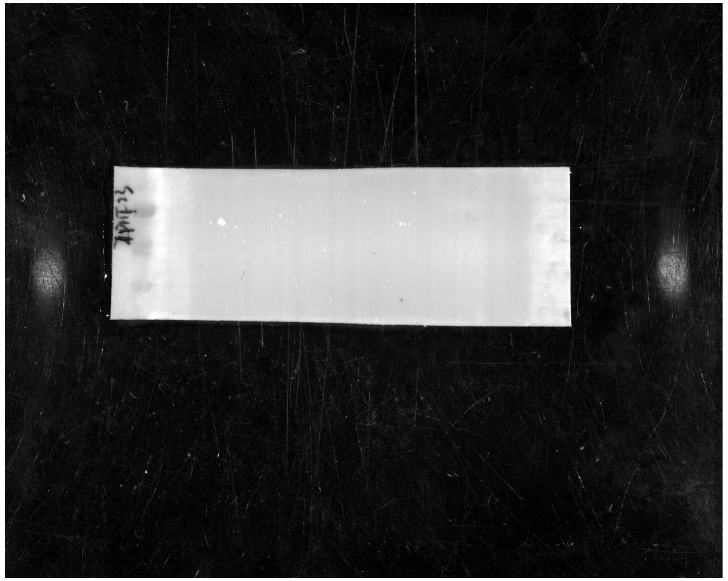

# Figure4B

H1299PHF23

A549PHF23

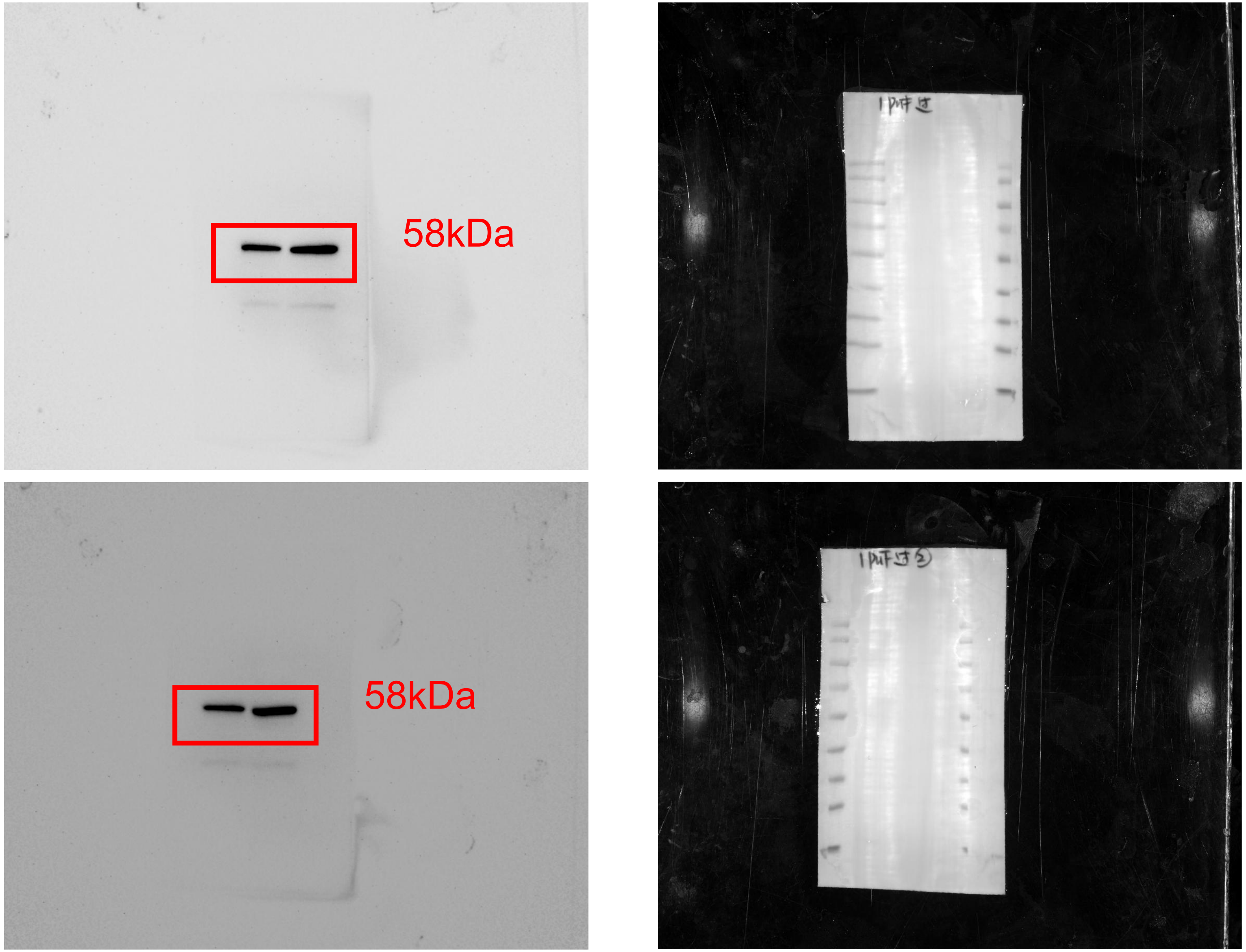

H1299p-erk

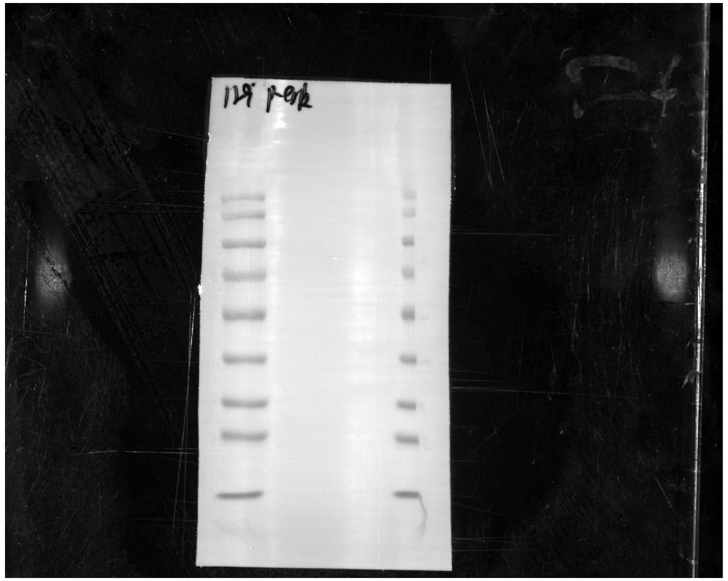

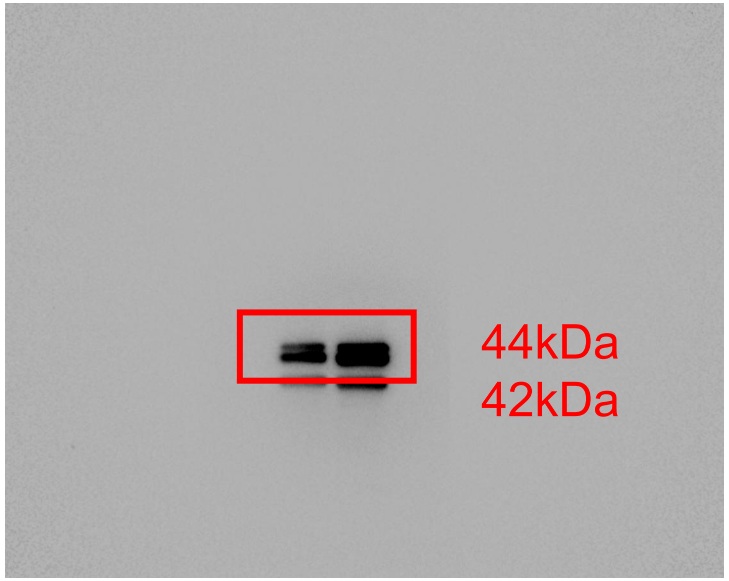


A549 p-erk

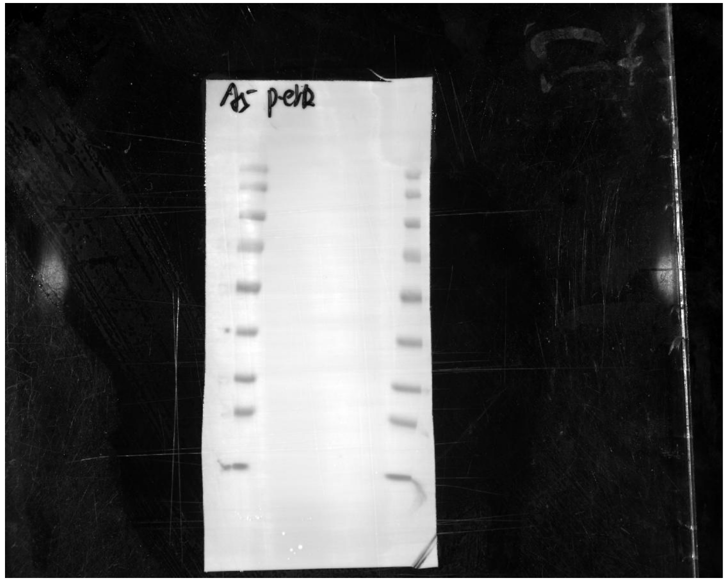

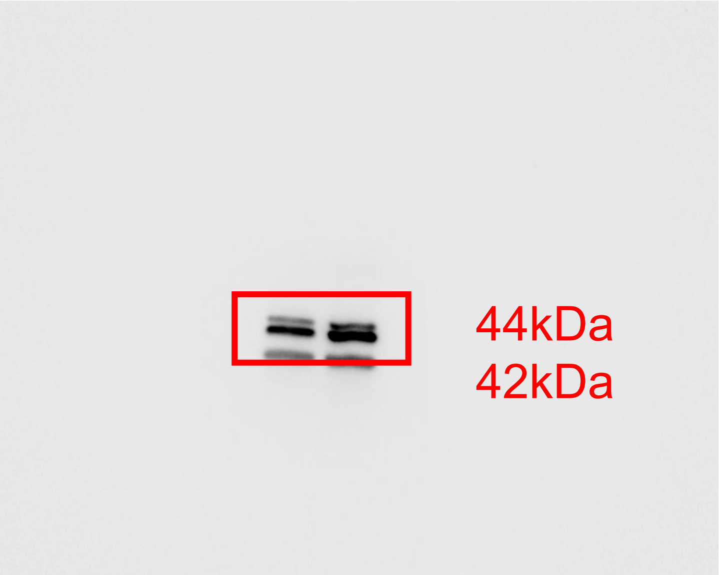

H1299erk

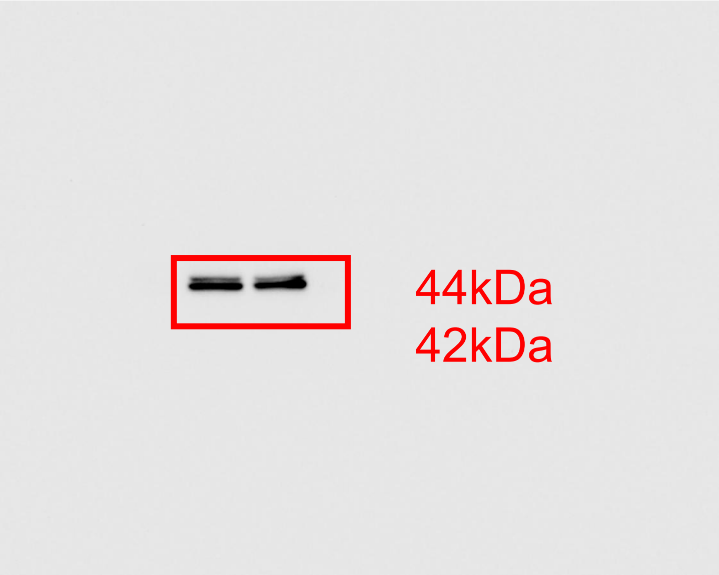


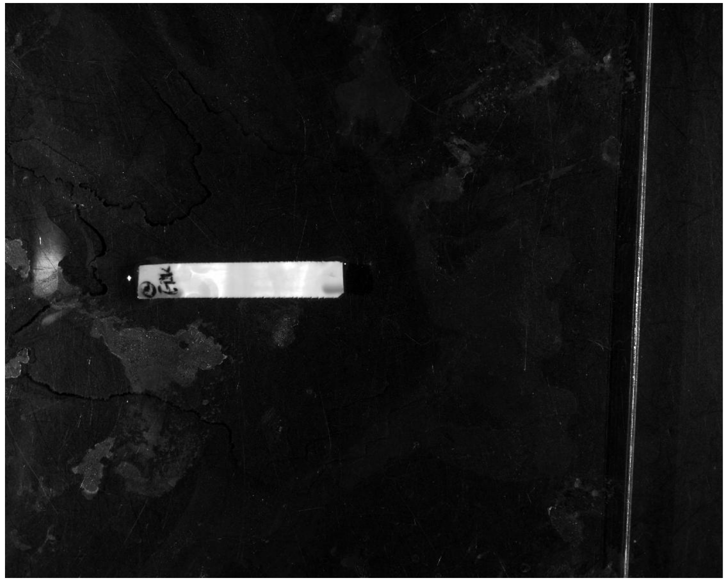

A549erk

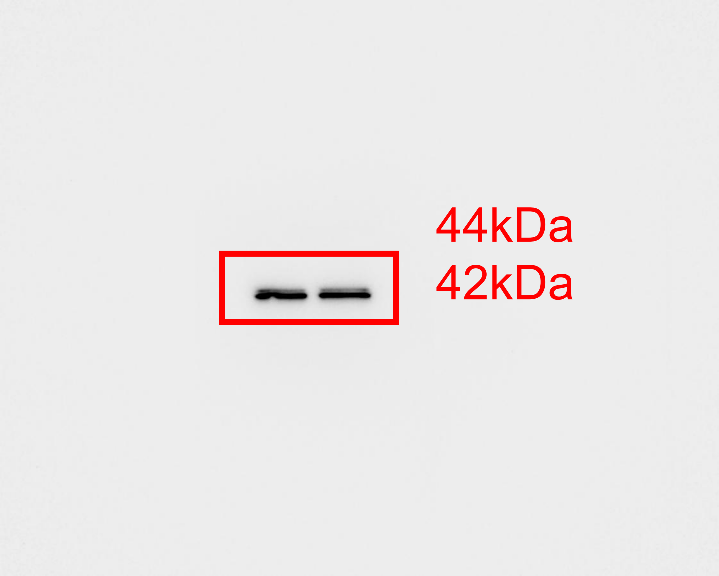


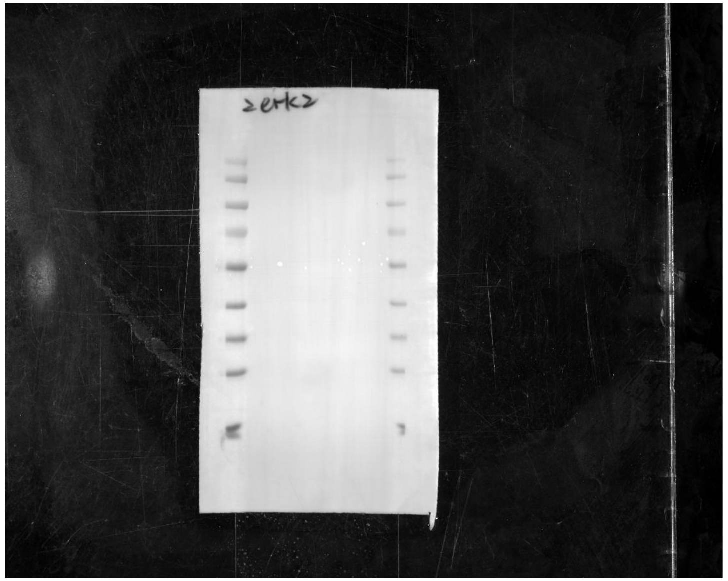

H1299c-myc

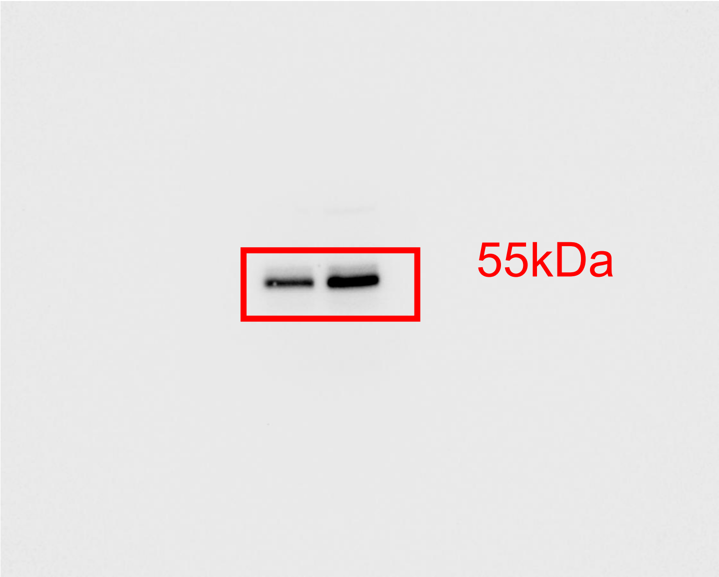


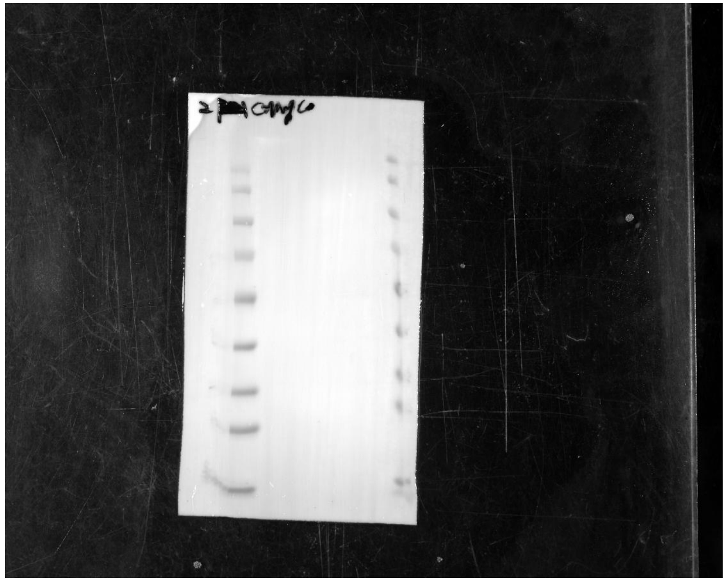

A549c-myc

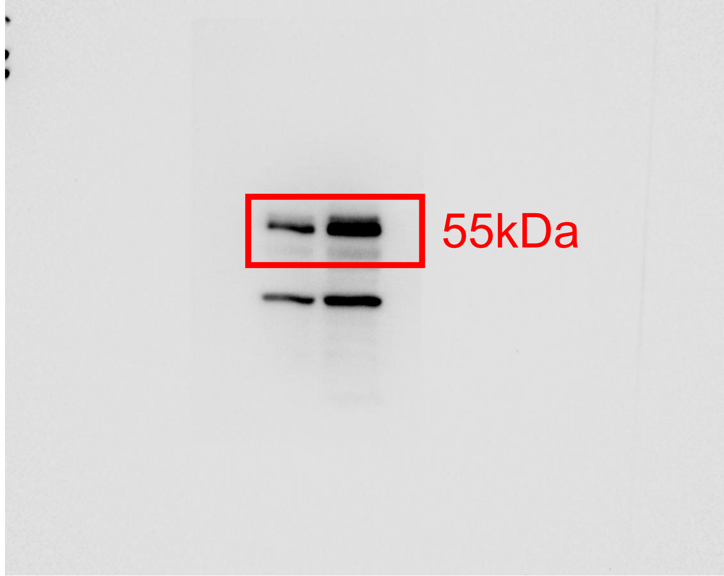


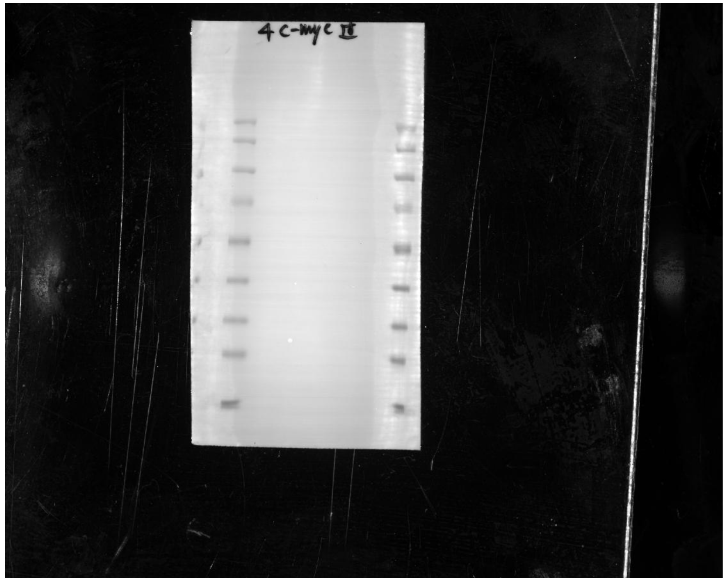

H1299p38

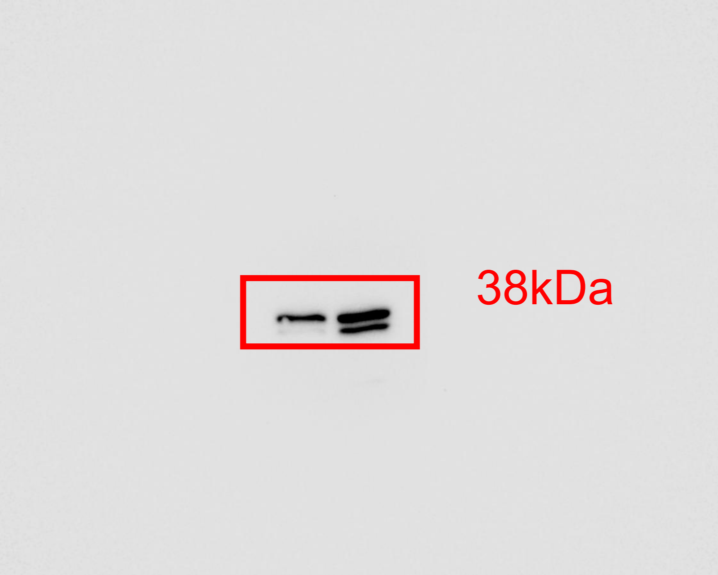


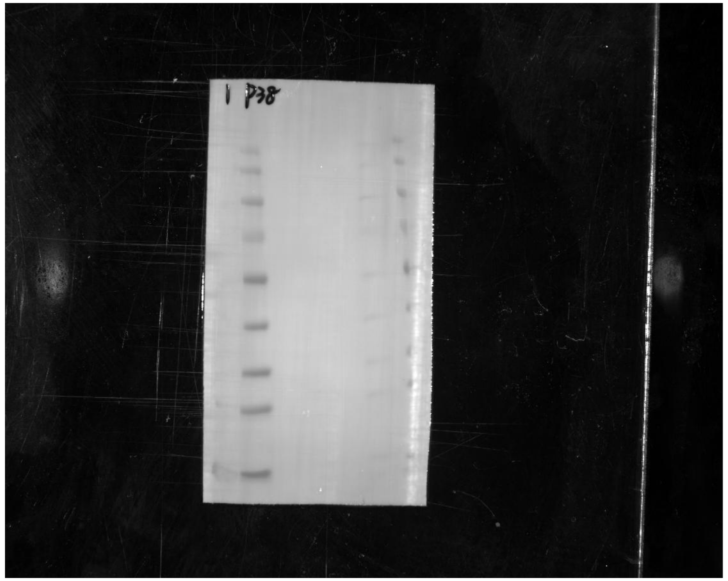

A549p38

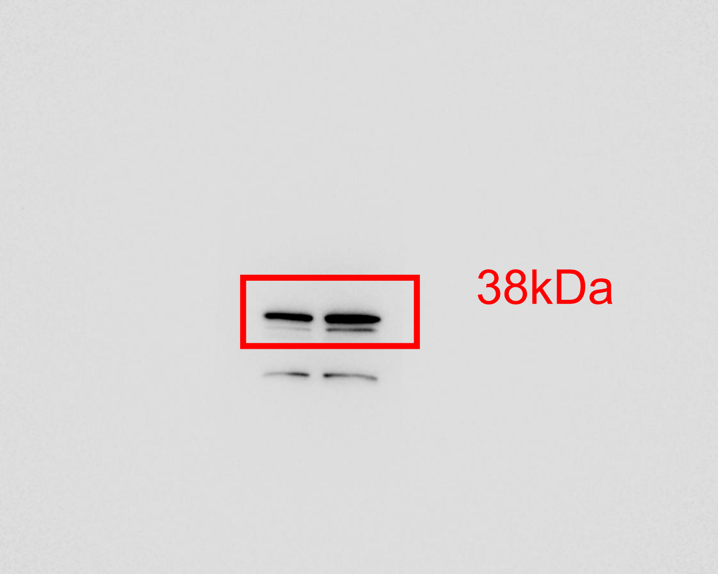


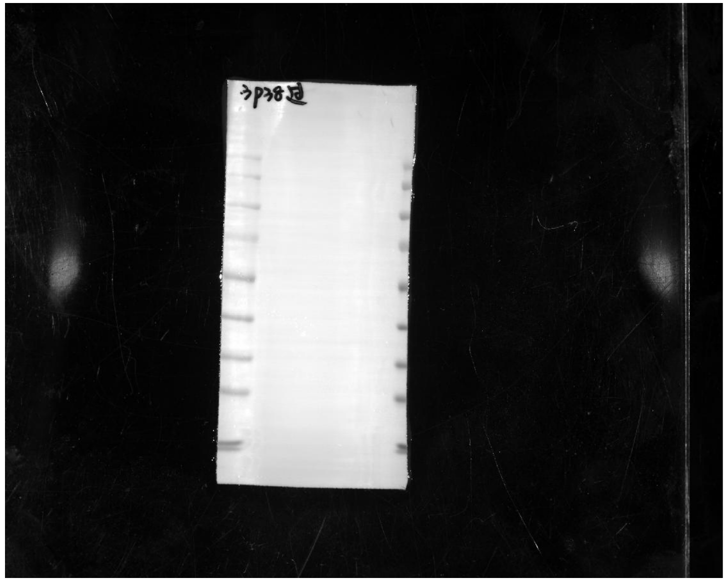

H1299p-jun

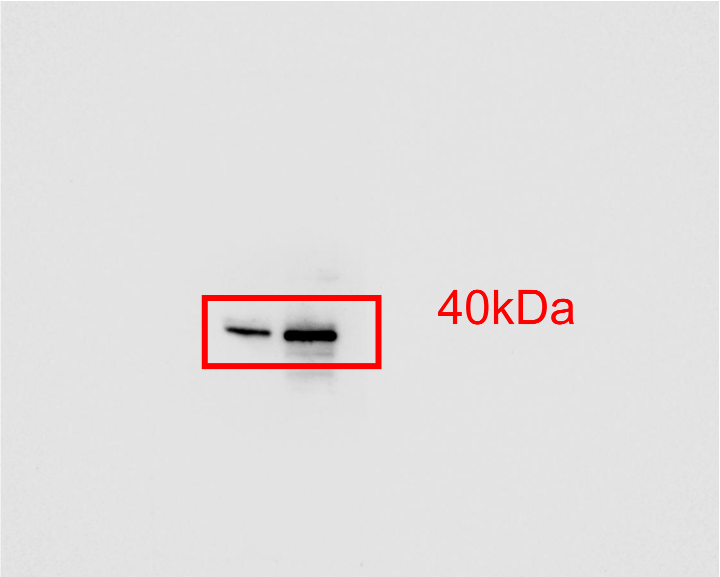


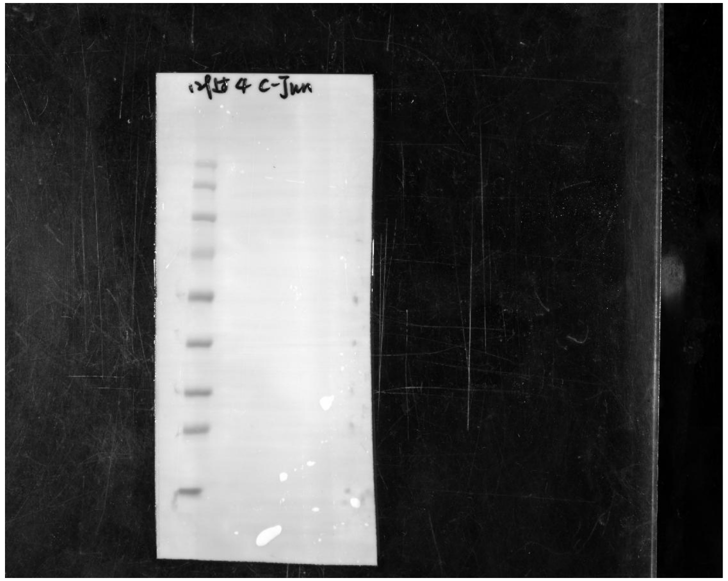

A549p-jun

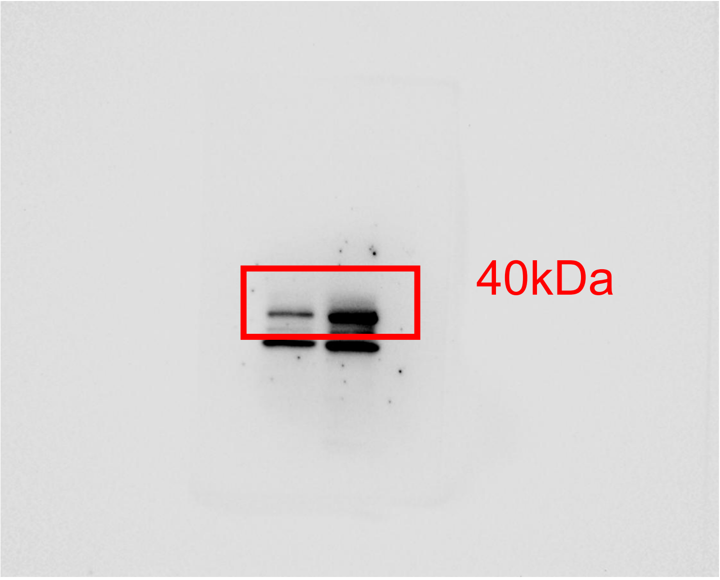


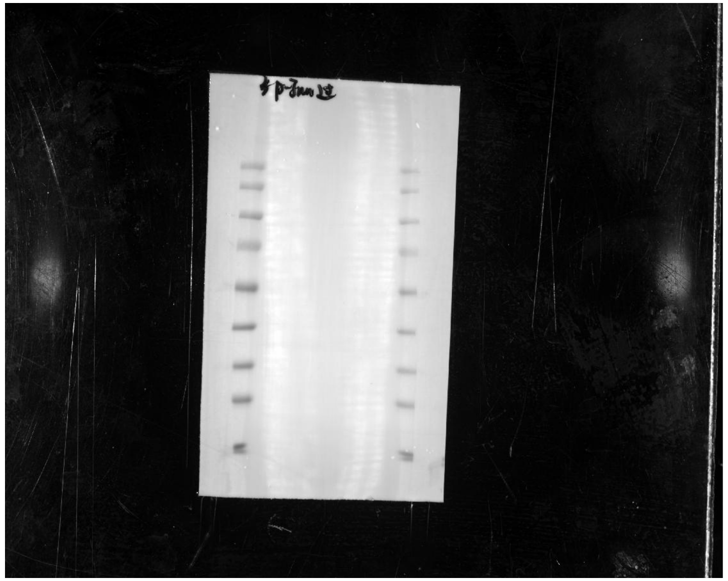

H1299
GA

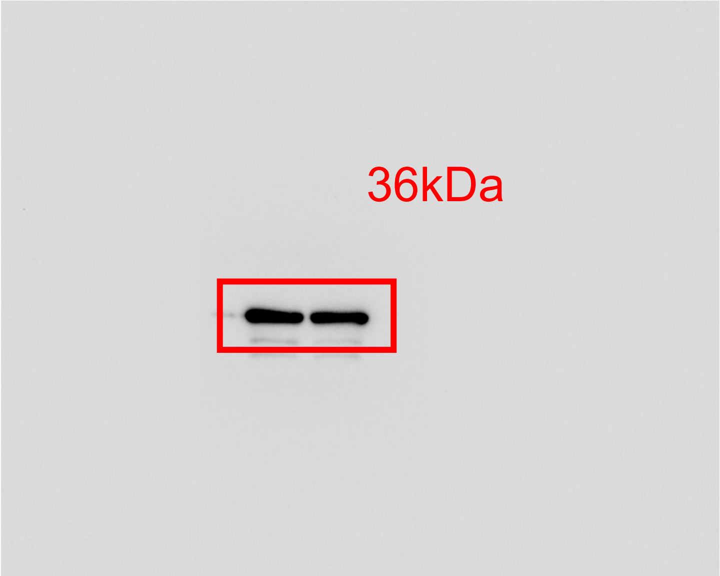


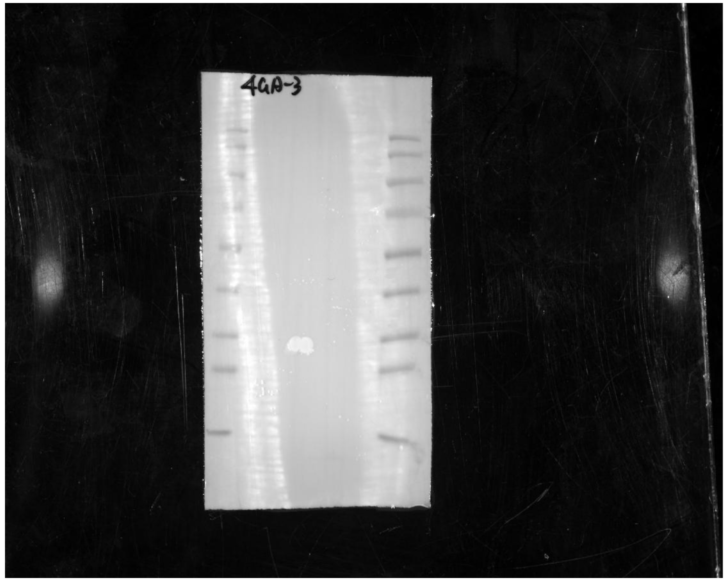


A549
GA

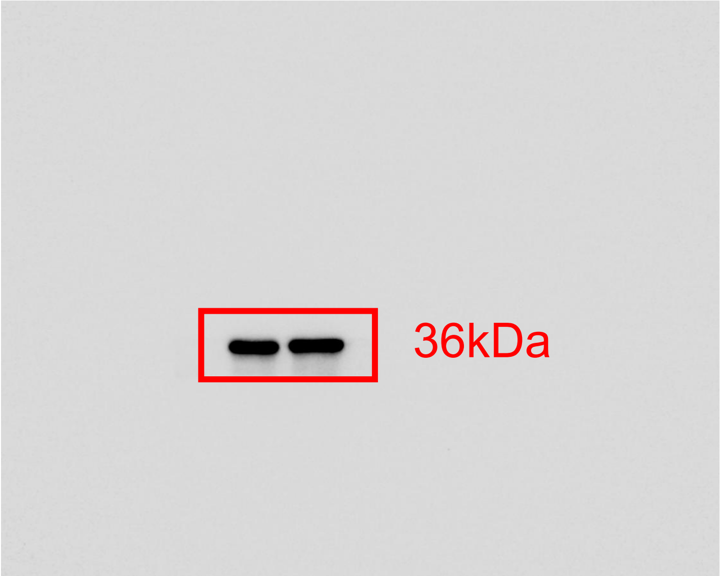


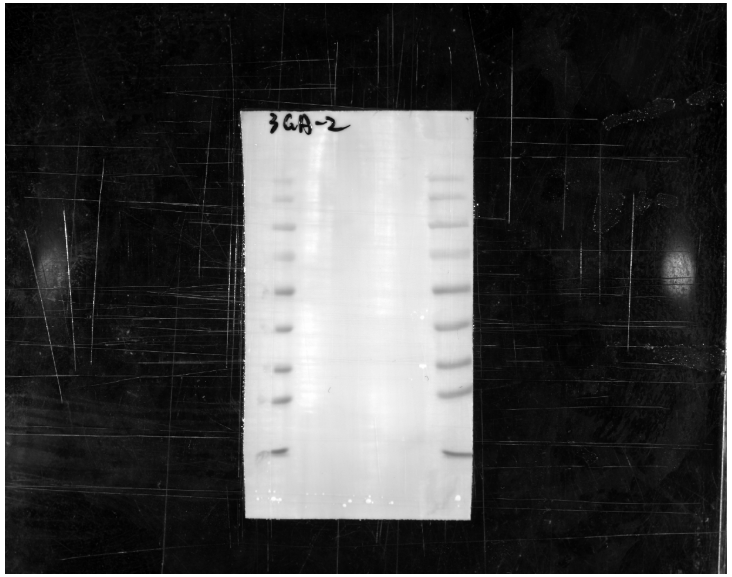

# S-Figure4B

H1299cas9PHF23

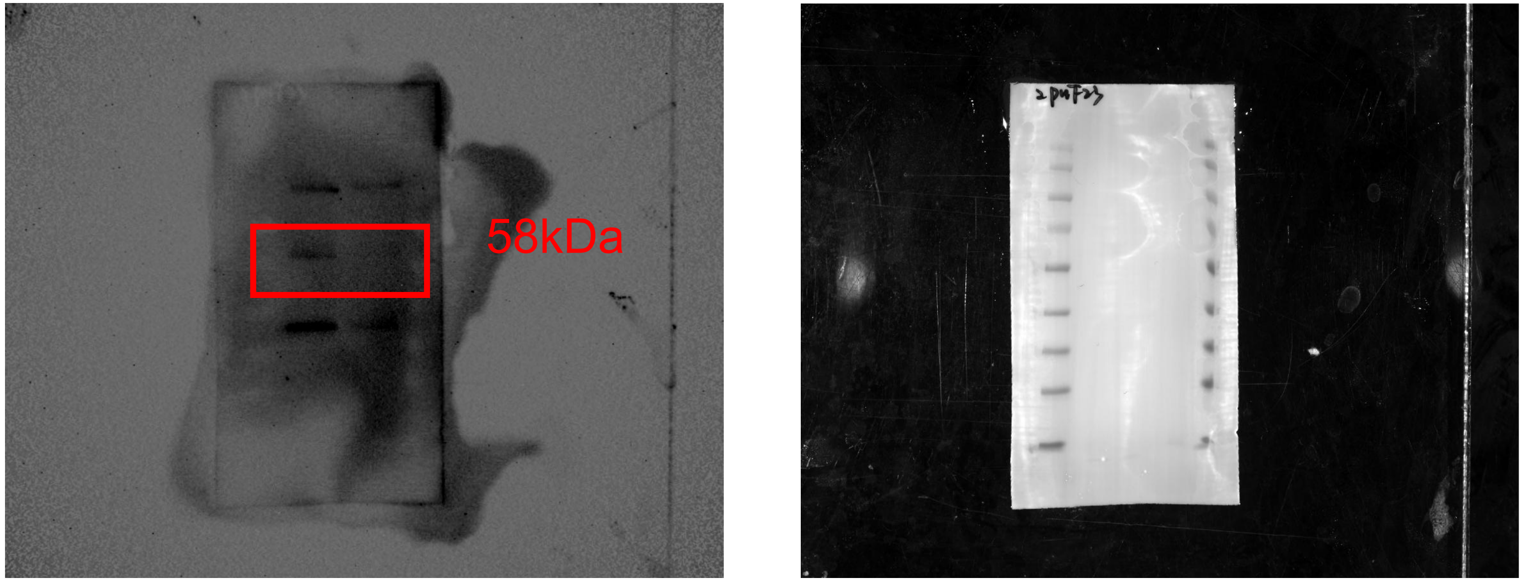


A549cas9PHF23

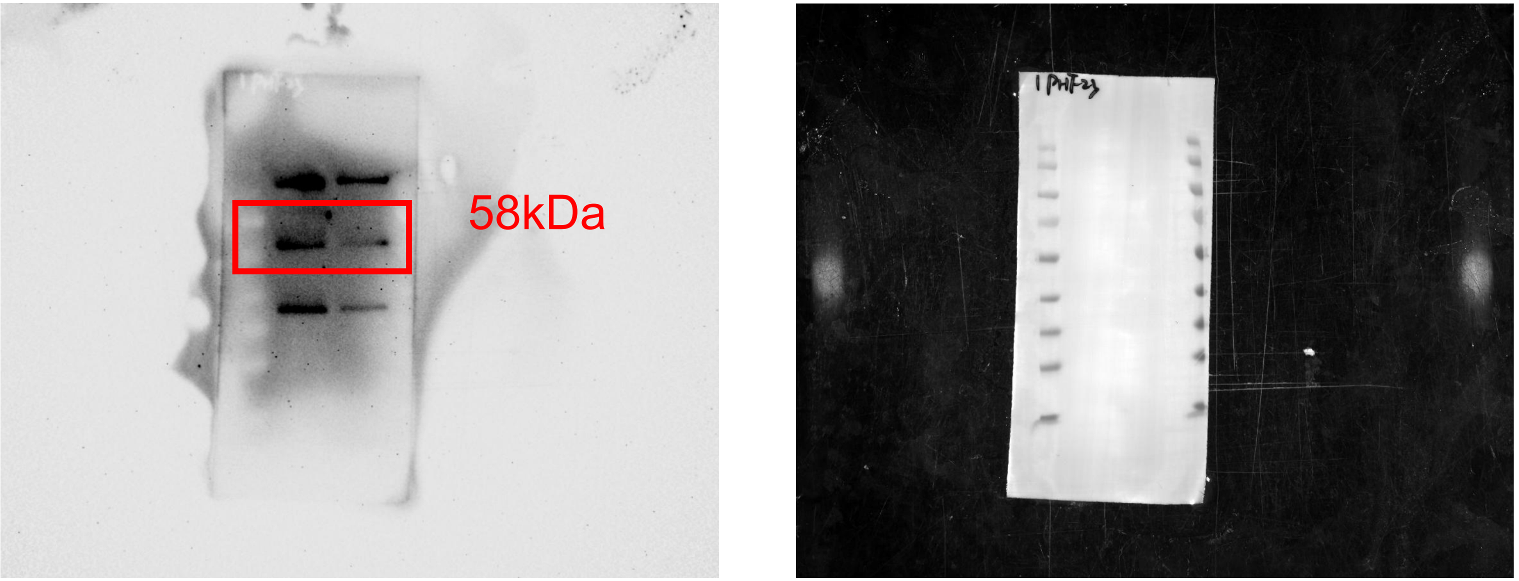

H1299p-erk

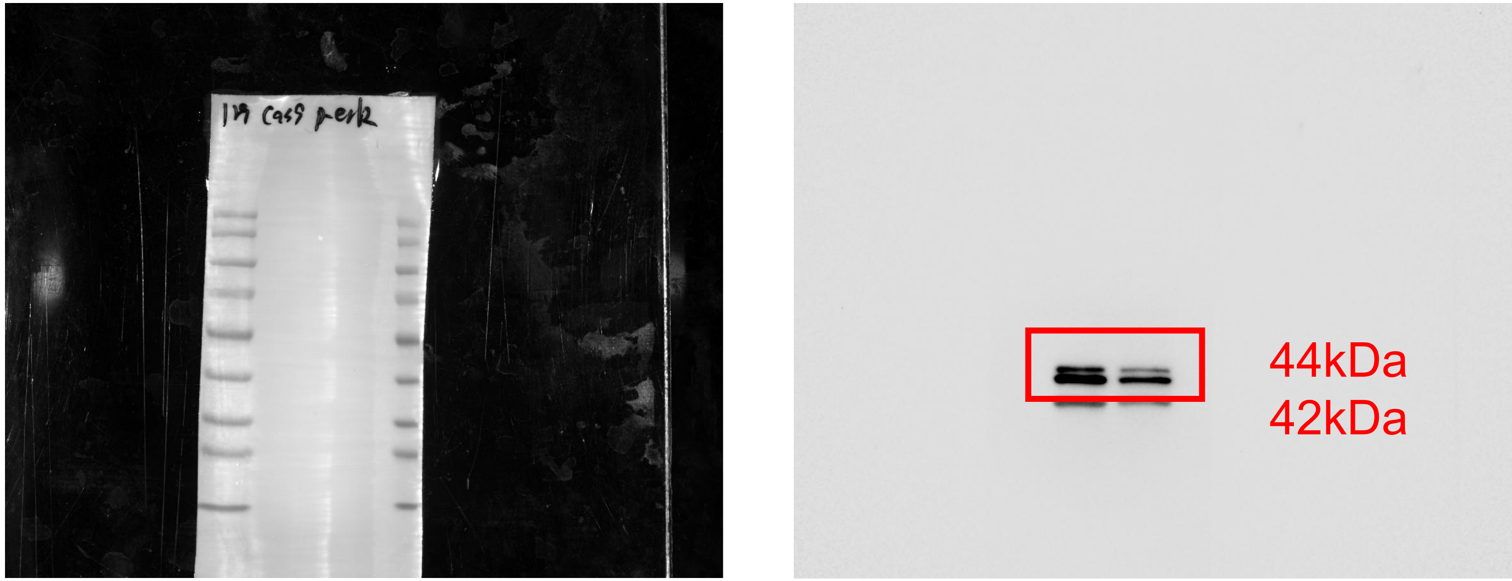


H1975p-erk

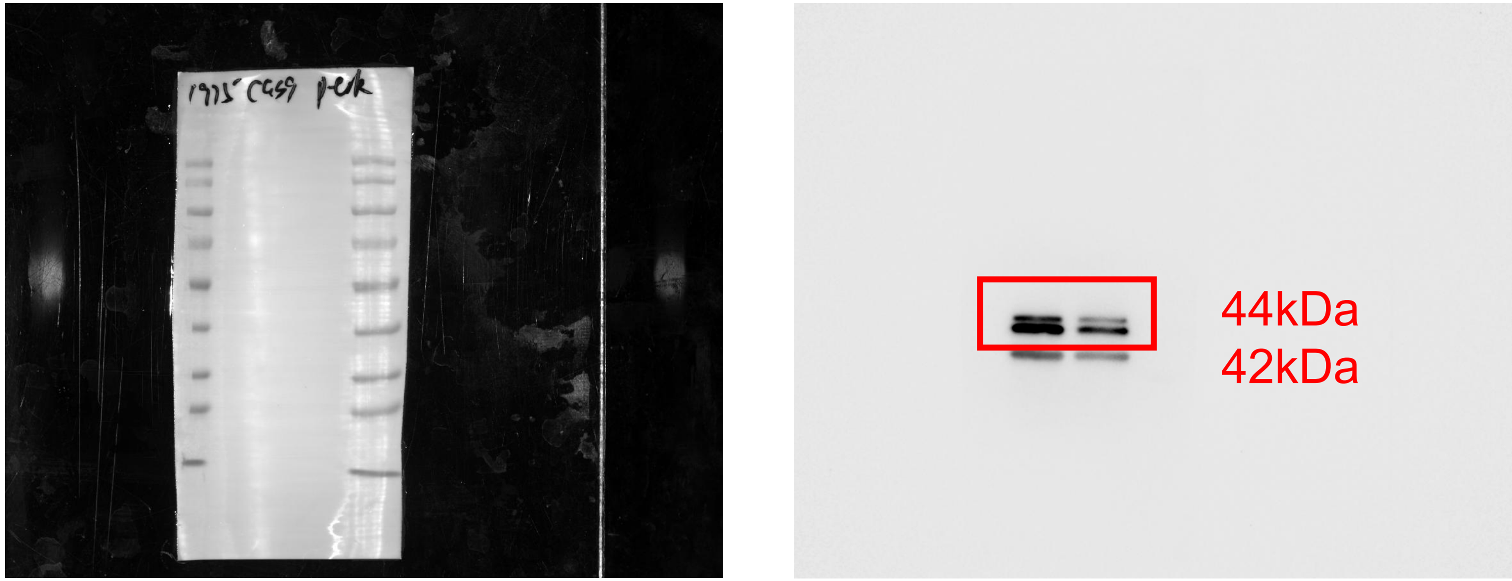

H1299erk

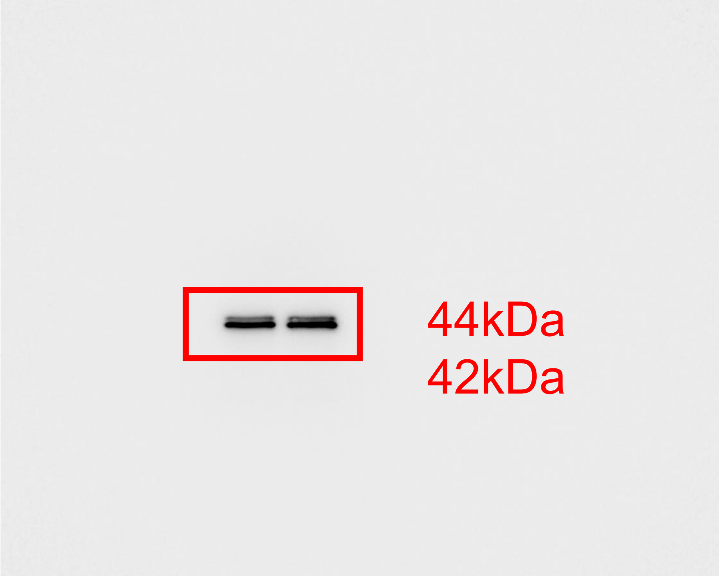


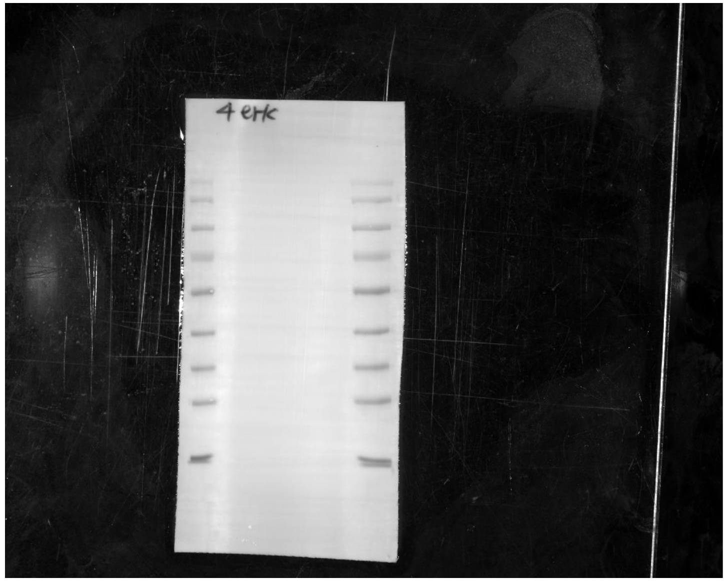


H1975erk

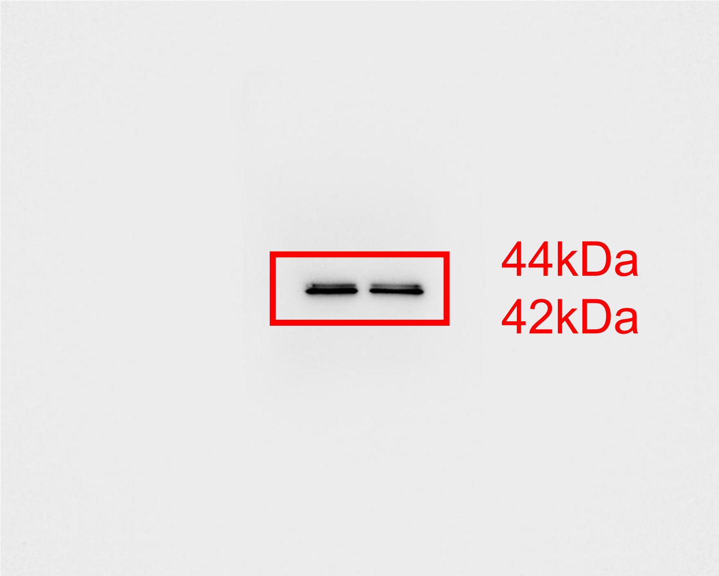


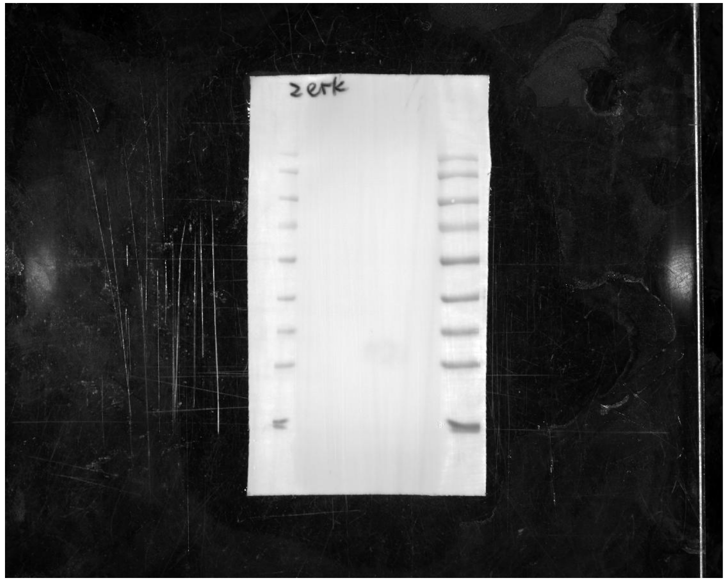

H1299cas9c-myc

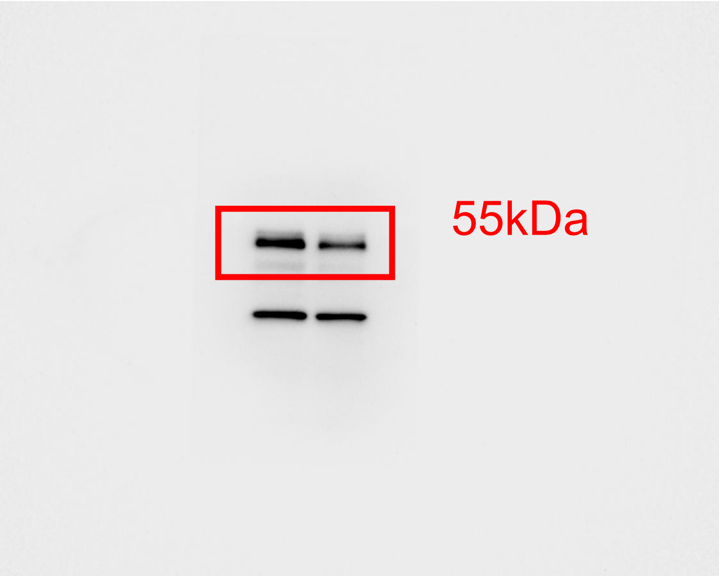


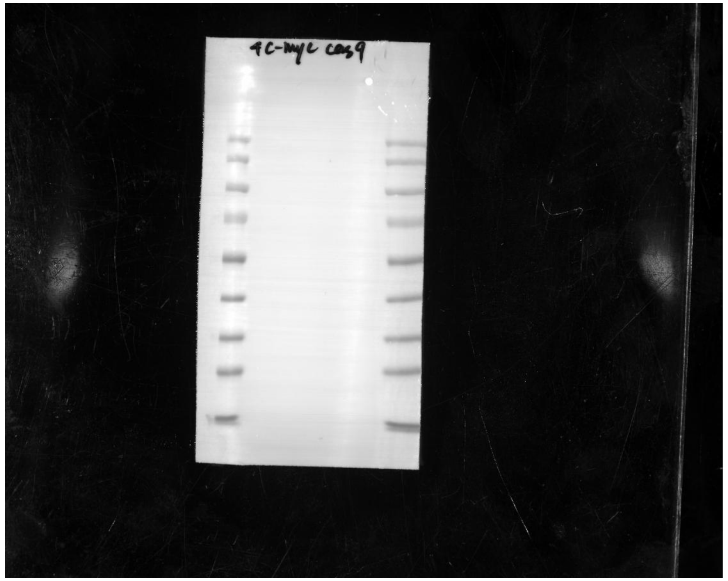

H1975cas9 c-myc

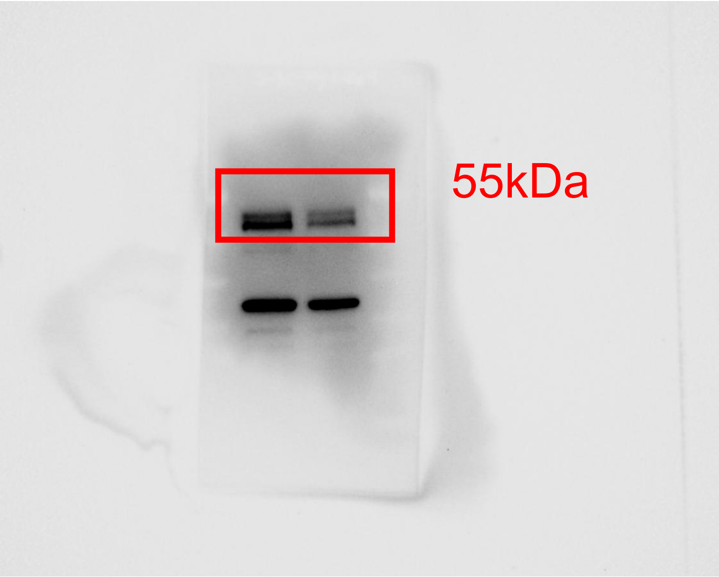


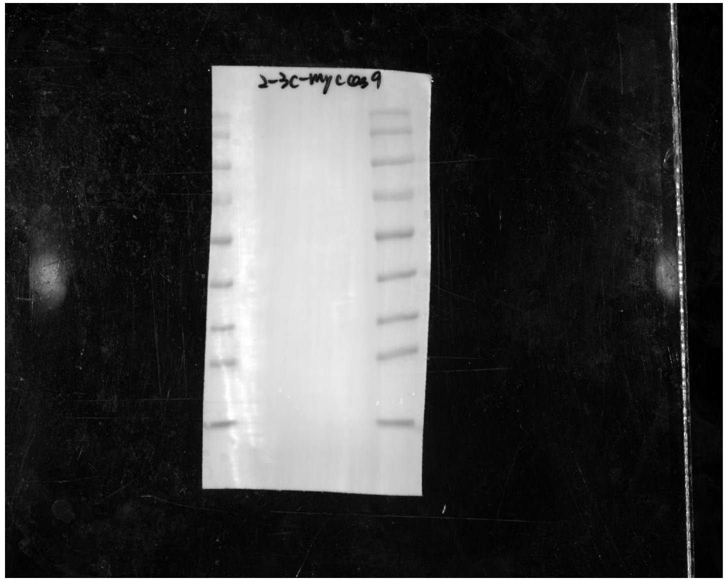

H1299cas9p38

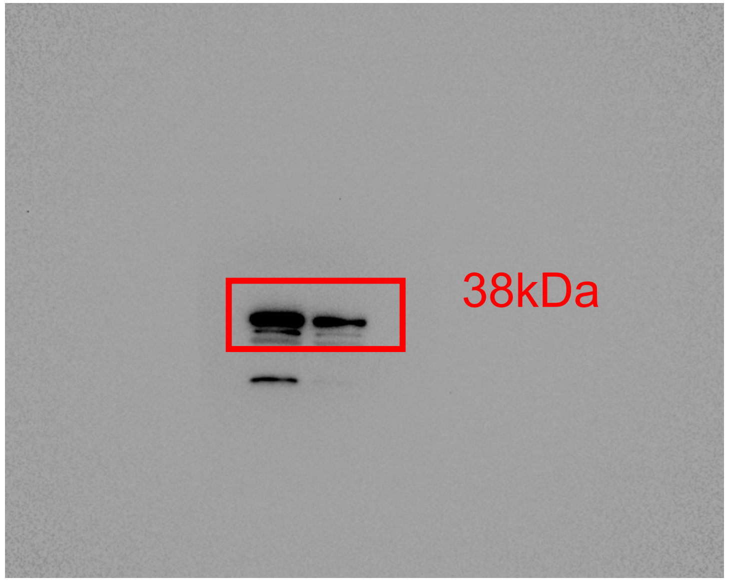


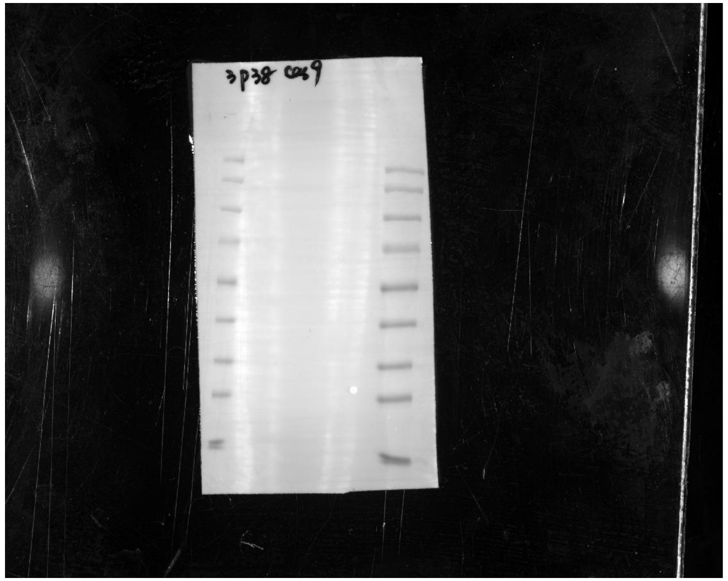

H1975cas9p38

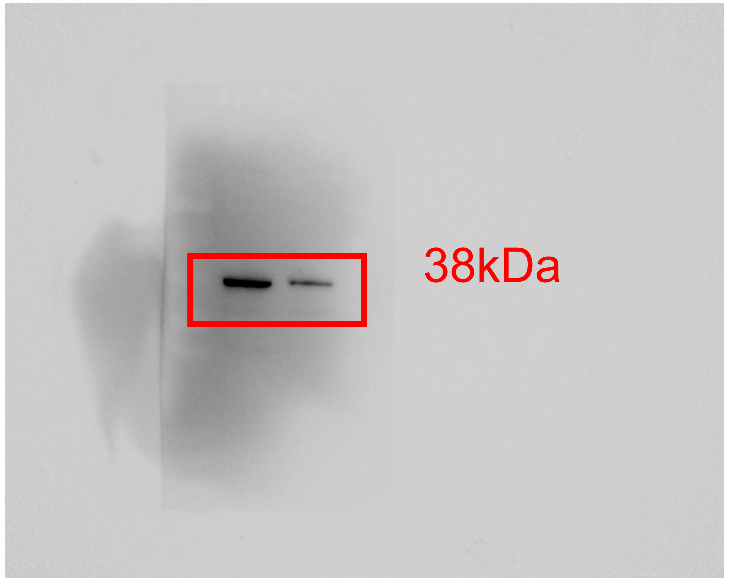


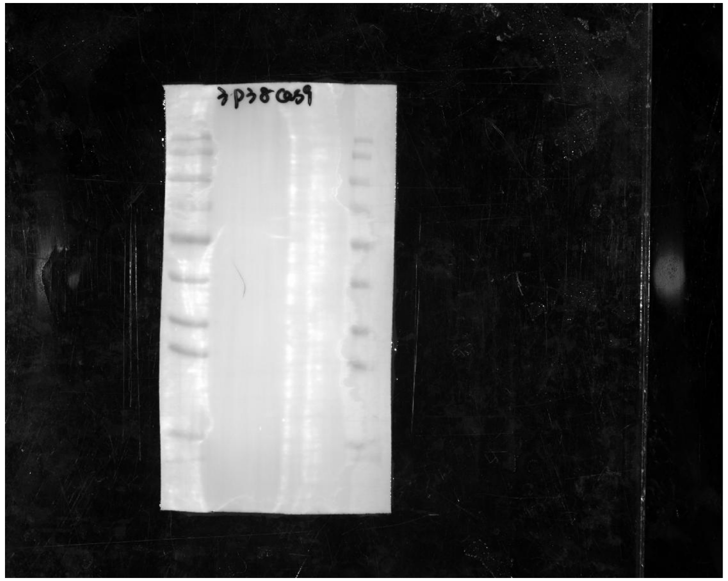

H1299cas9p-jun

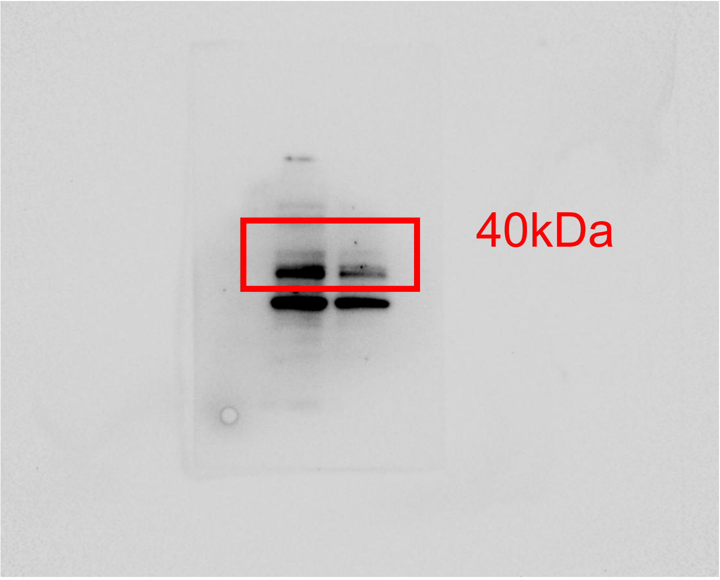


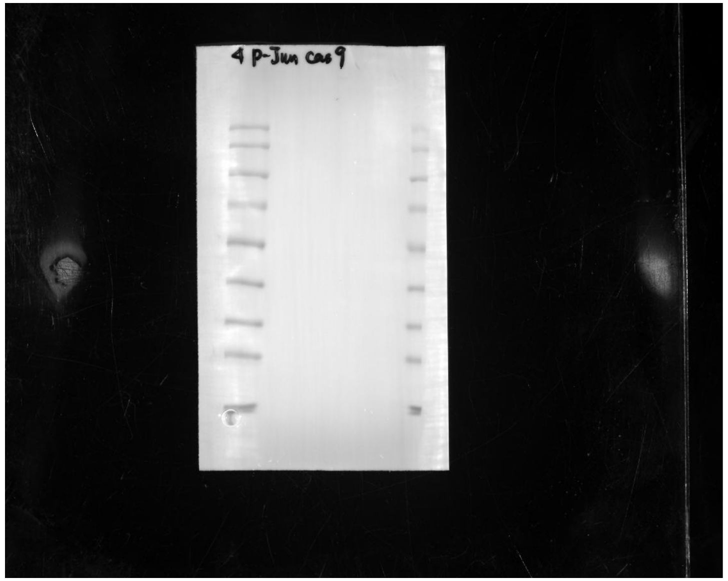


H1975cas9p-jun

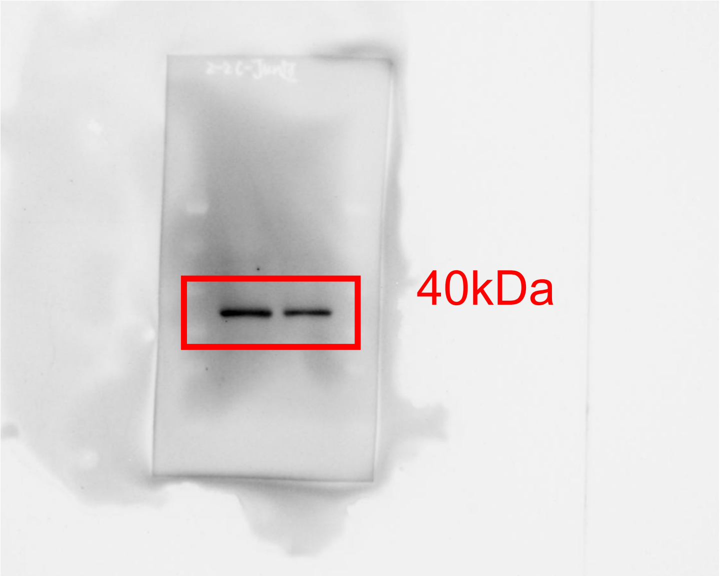


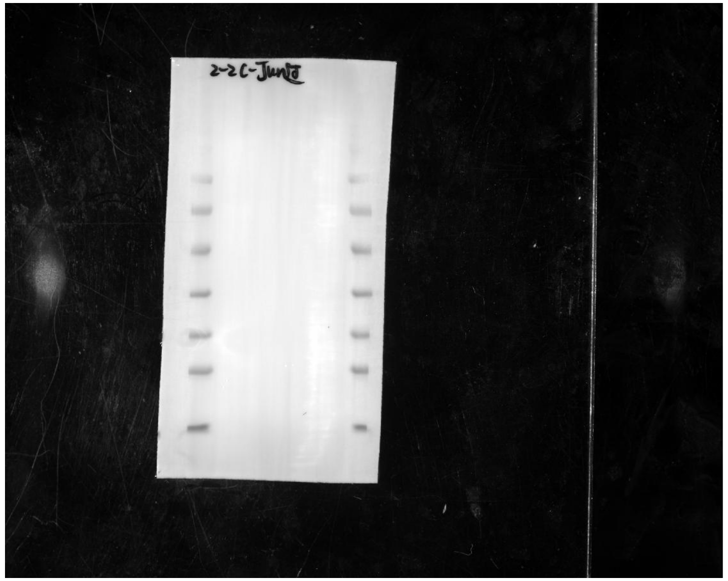

H1299
GAPDH

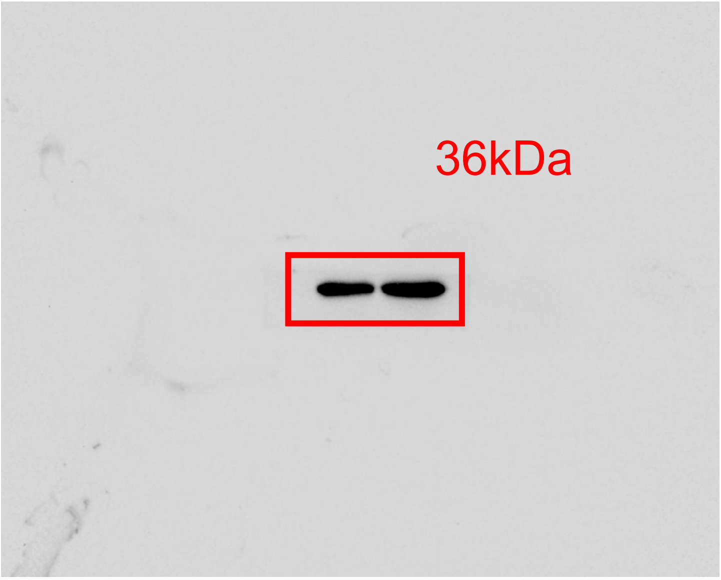


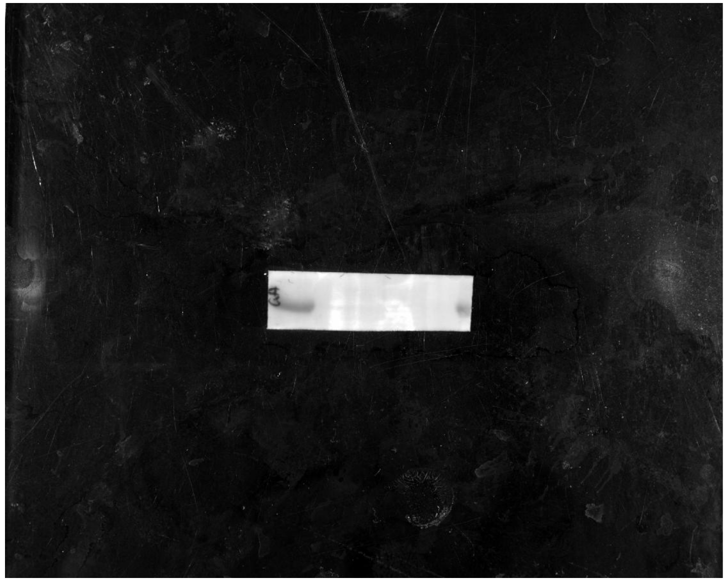

H1975
GAPDH

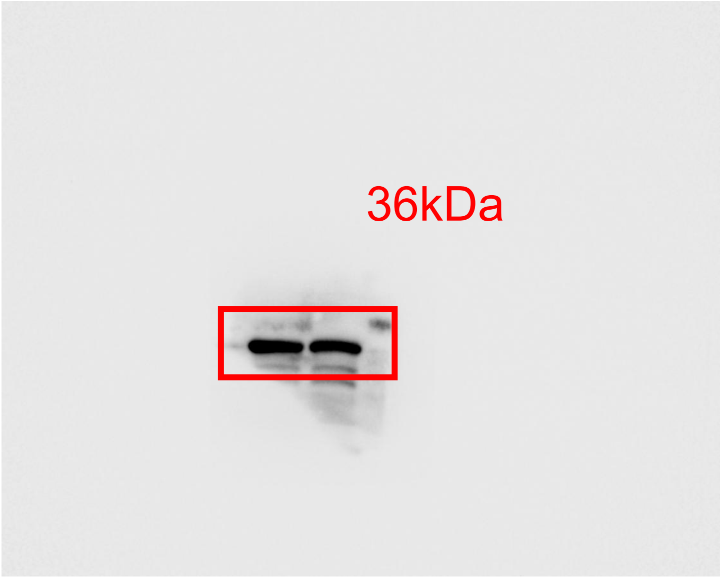


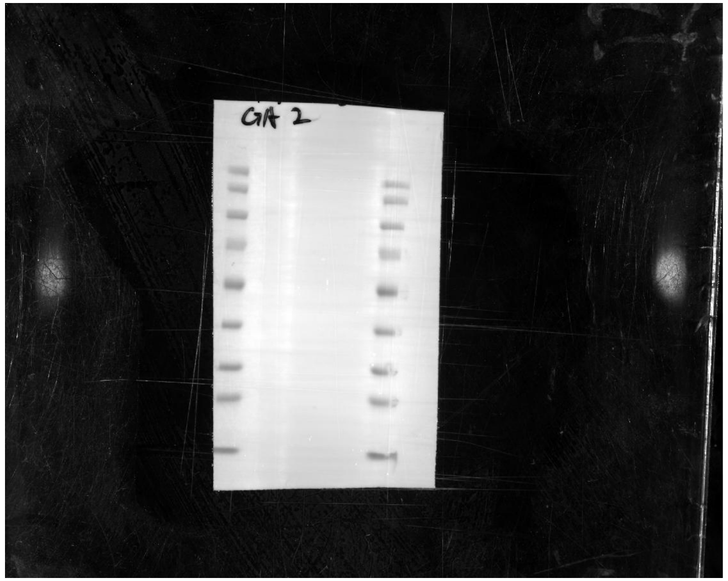

# Figure4C

H1299PHF23-myc

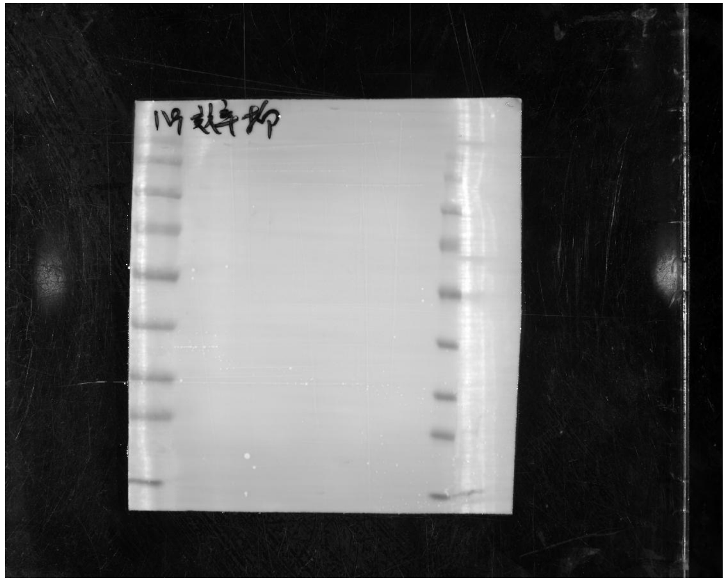

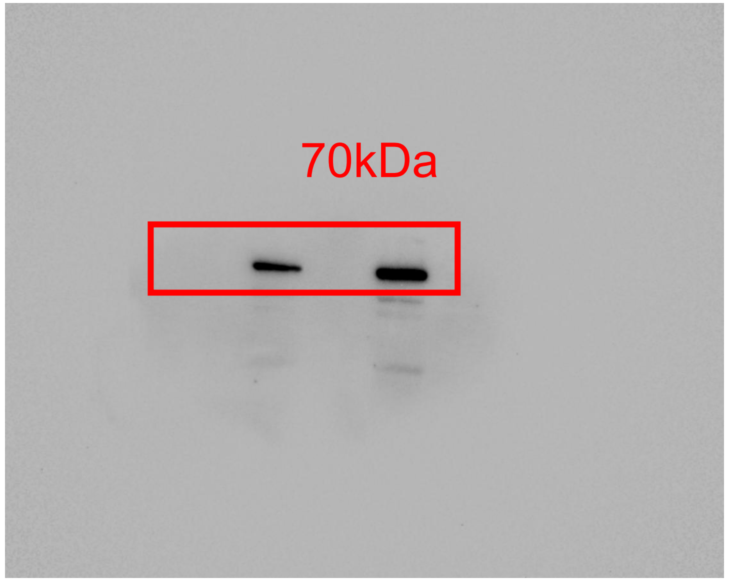


A549PHF23-myc

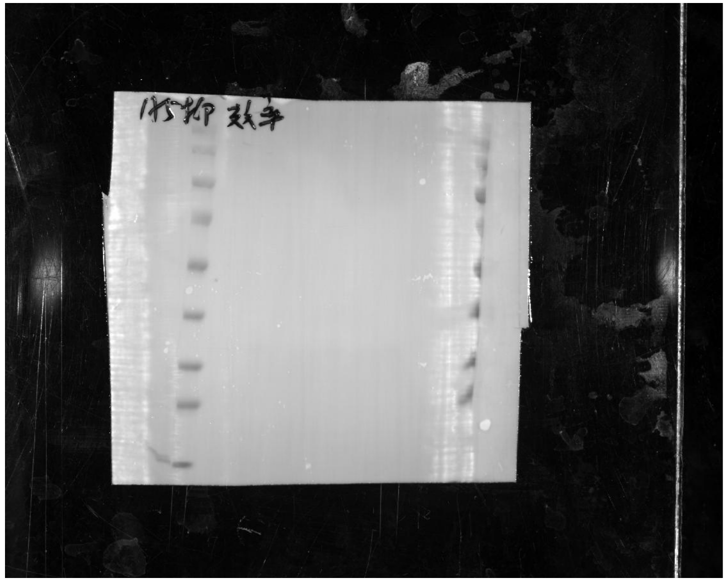

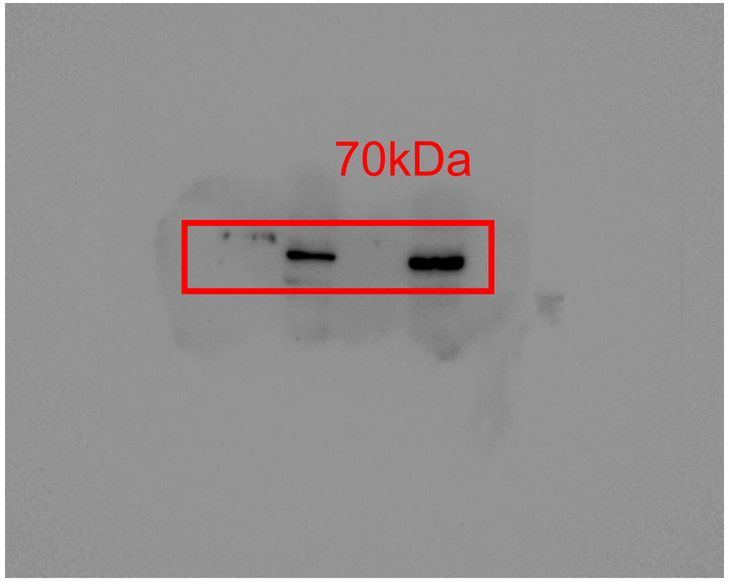

H1299p-erk

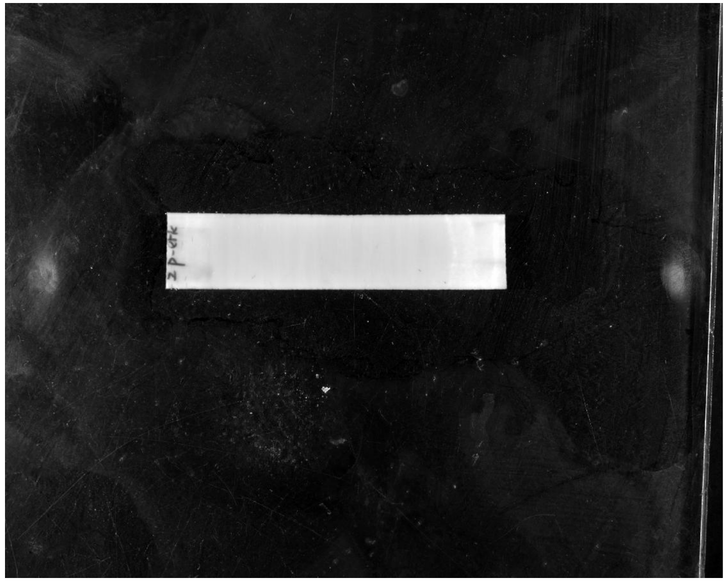

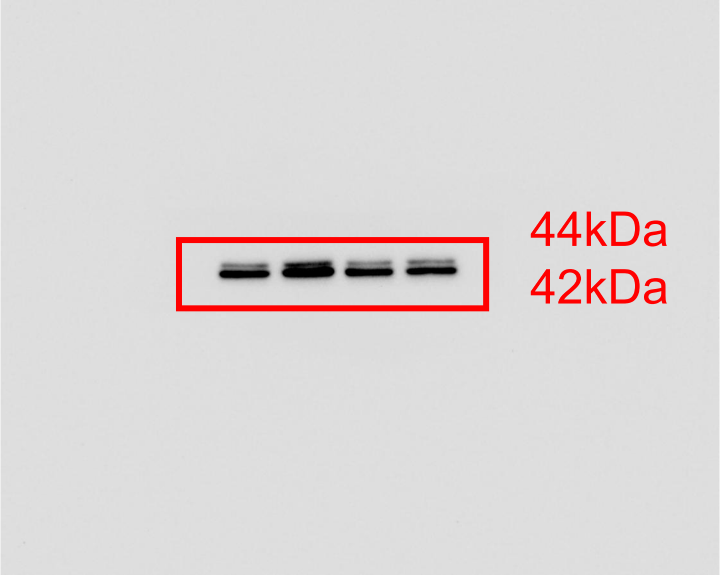


A549p-erk

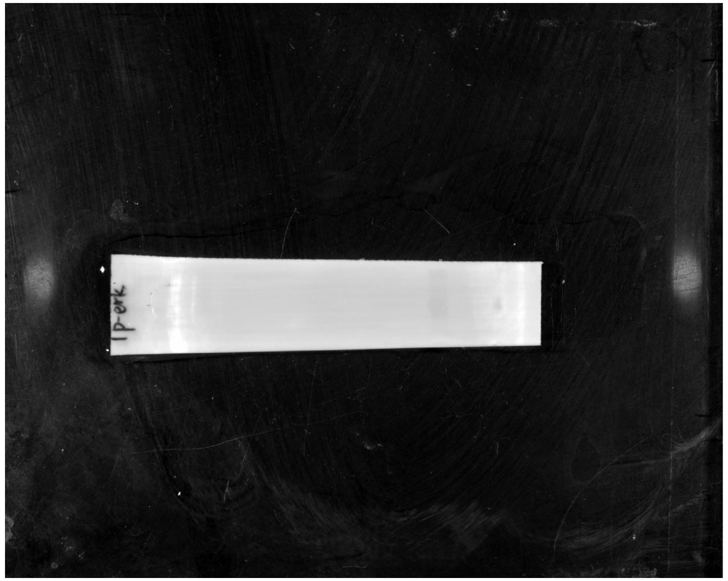

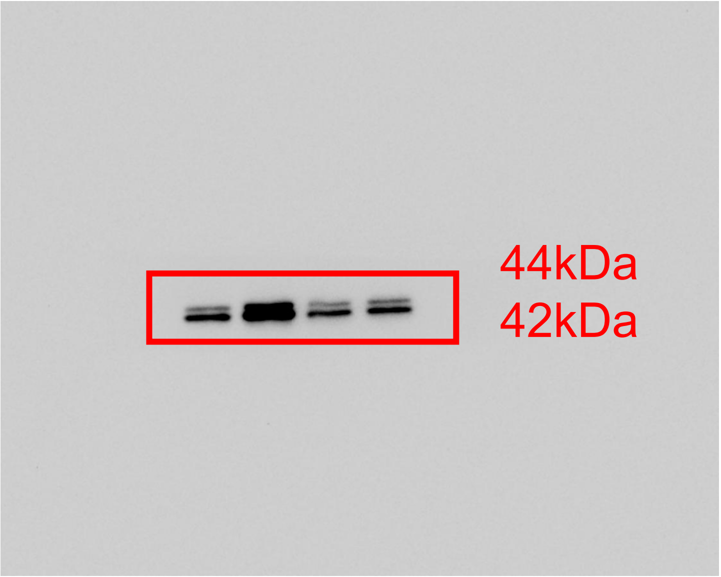

H1299erk

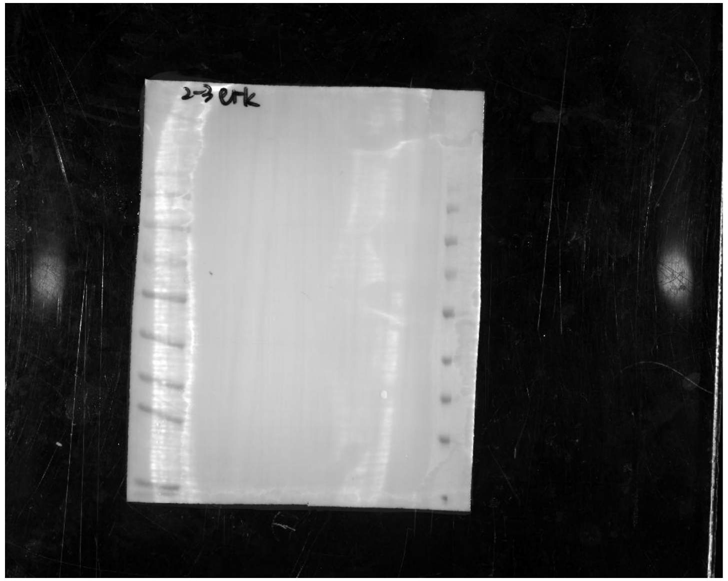

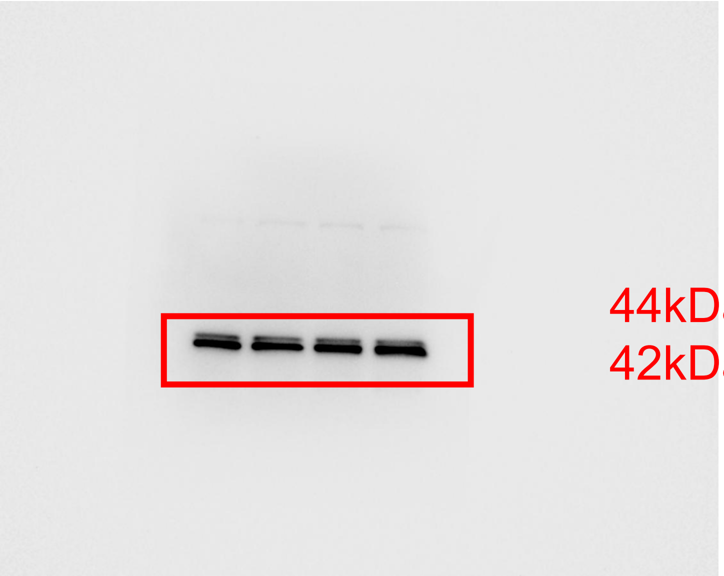

44kDa
42kDa

A549erk

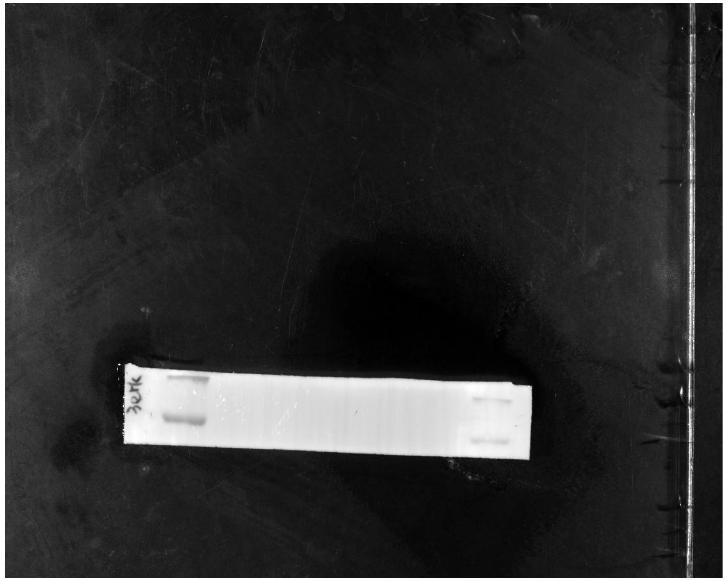

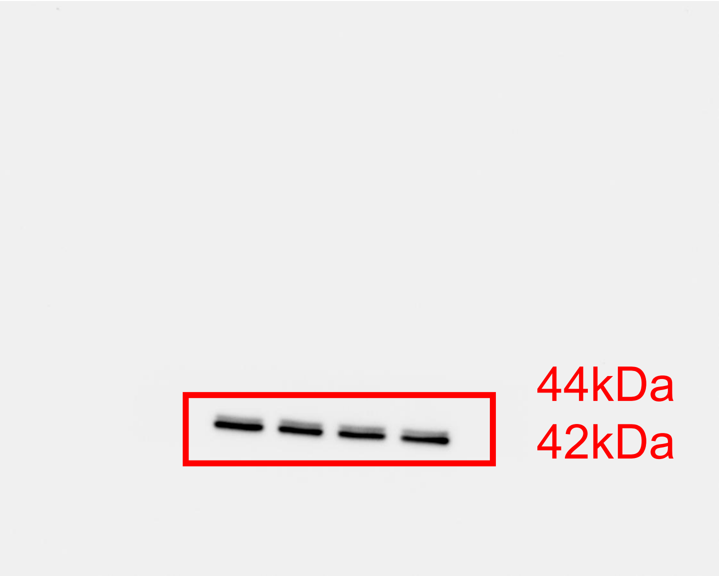

44kDa
42kDa

H1299p38

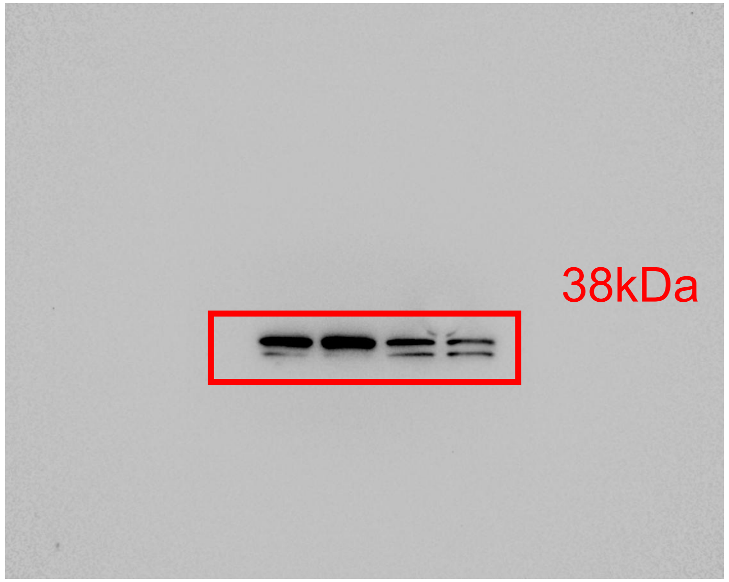


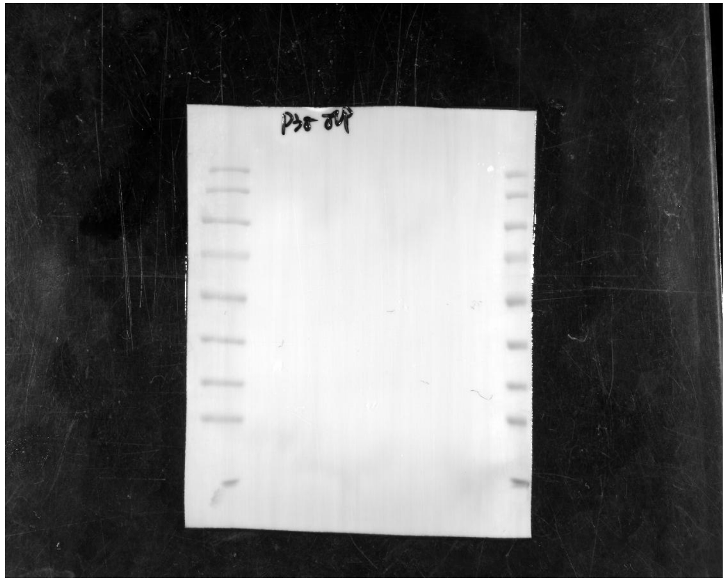

A549p38

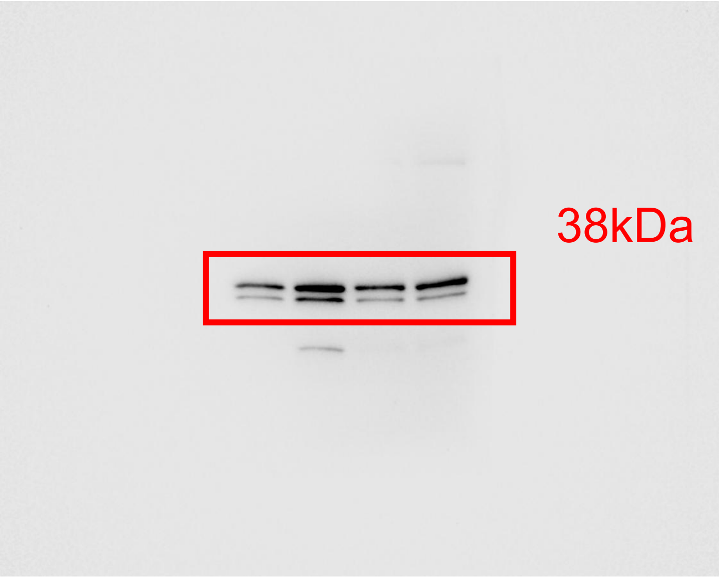


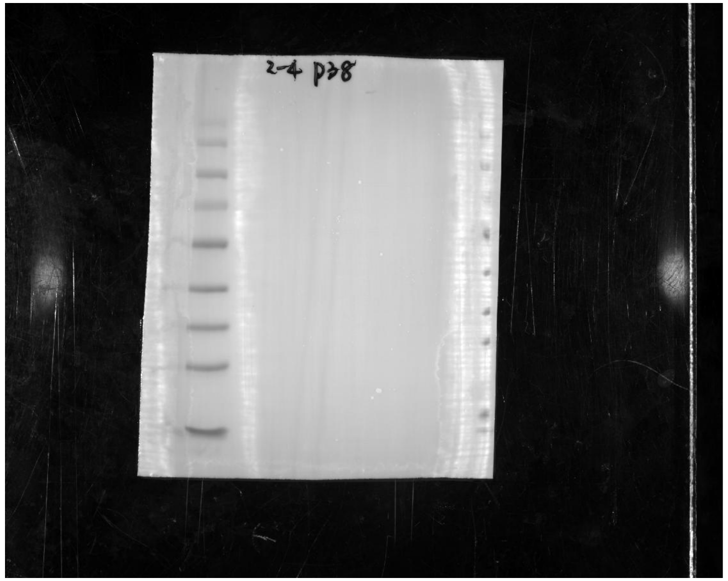

H1299c-myc

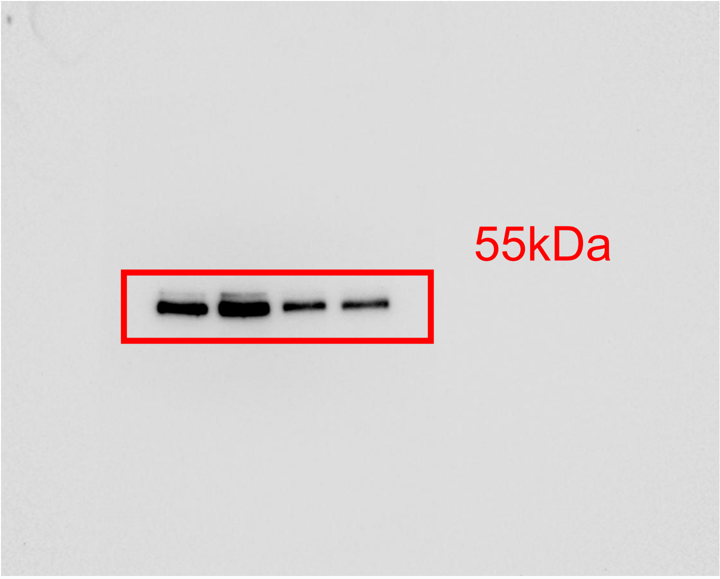


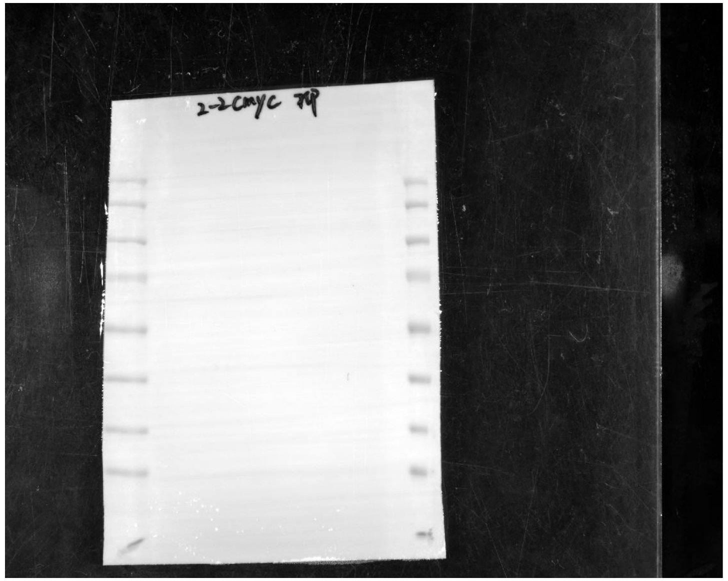

A549c-myc

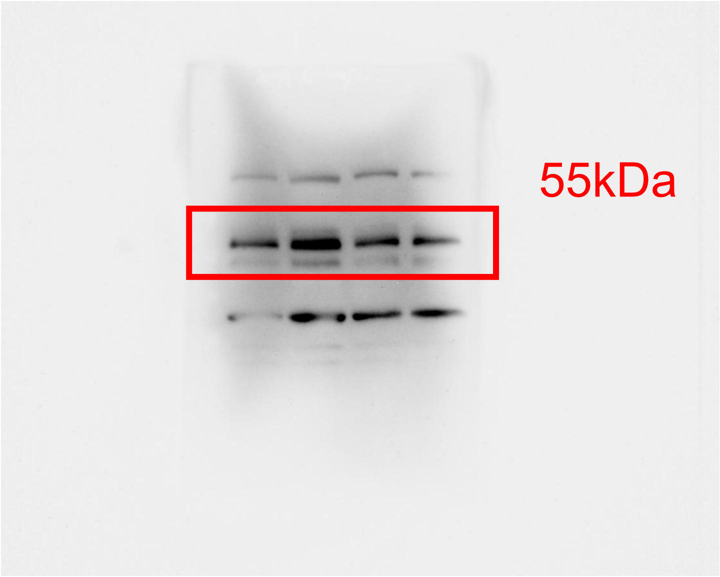


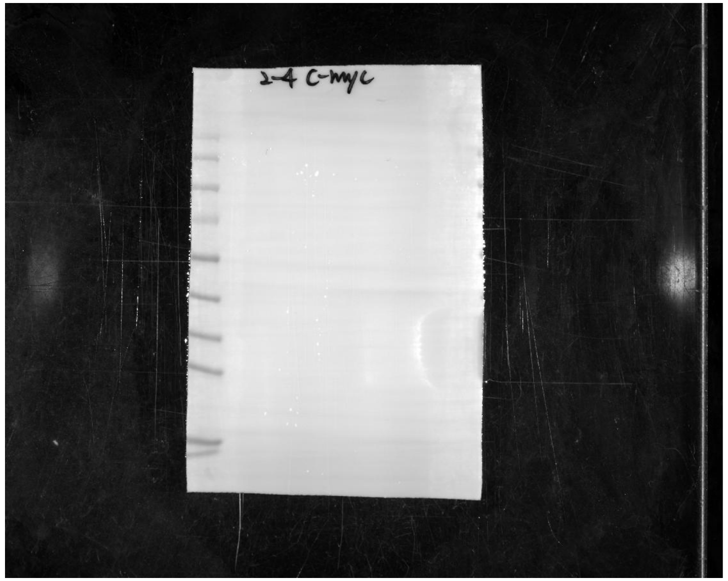

H1299p-jun(Ser73)

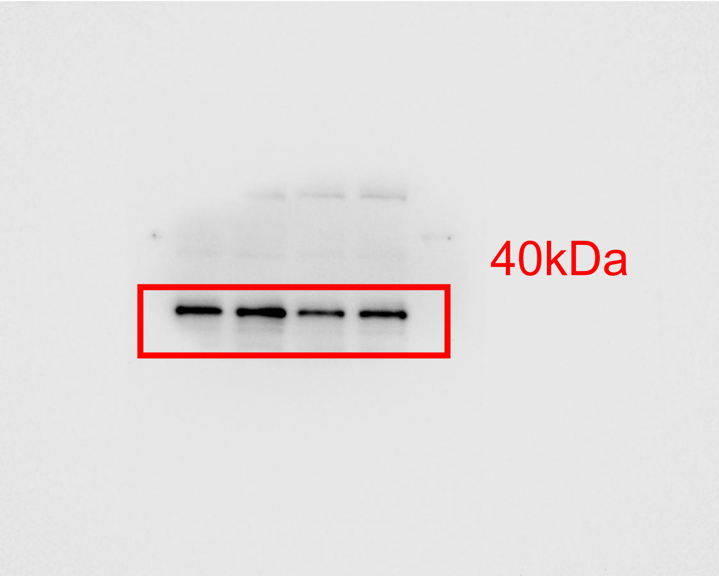

40kDa

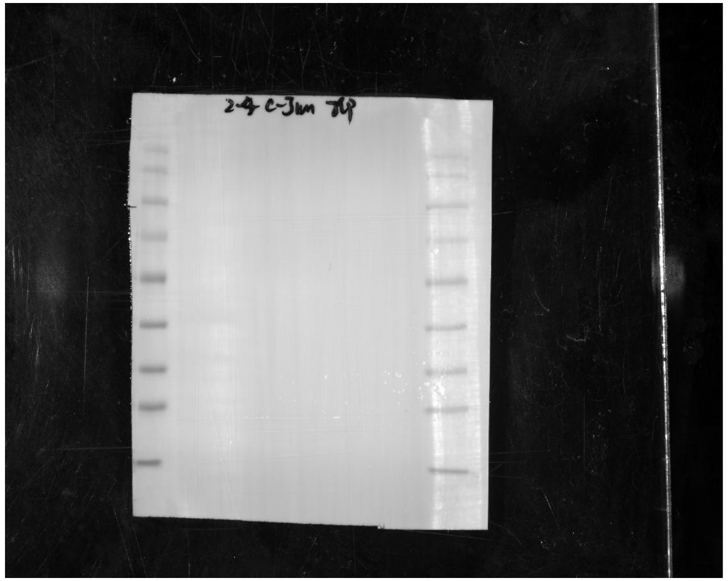

A549p-jun(Ser73)

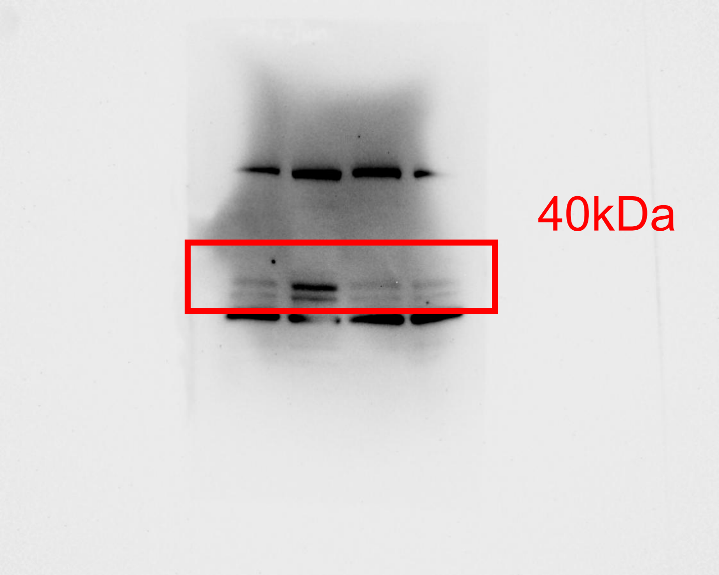

40kDa

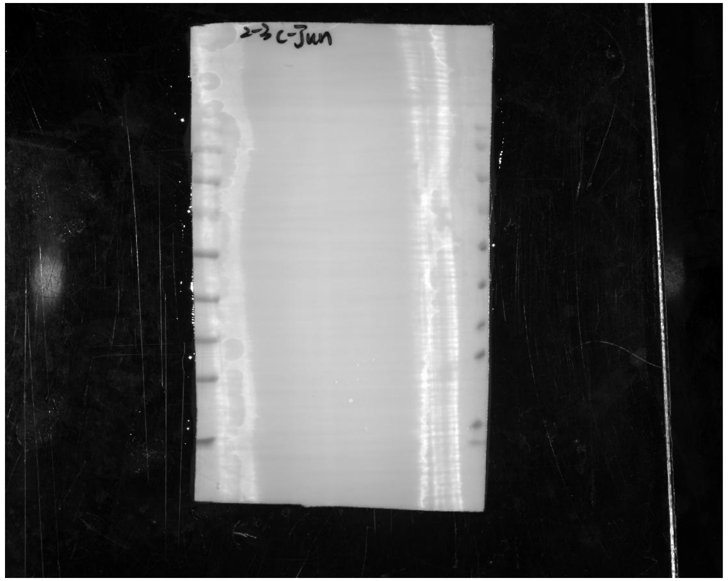

H1299GAPDH

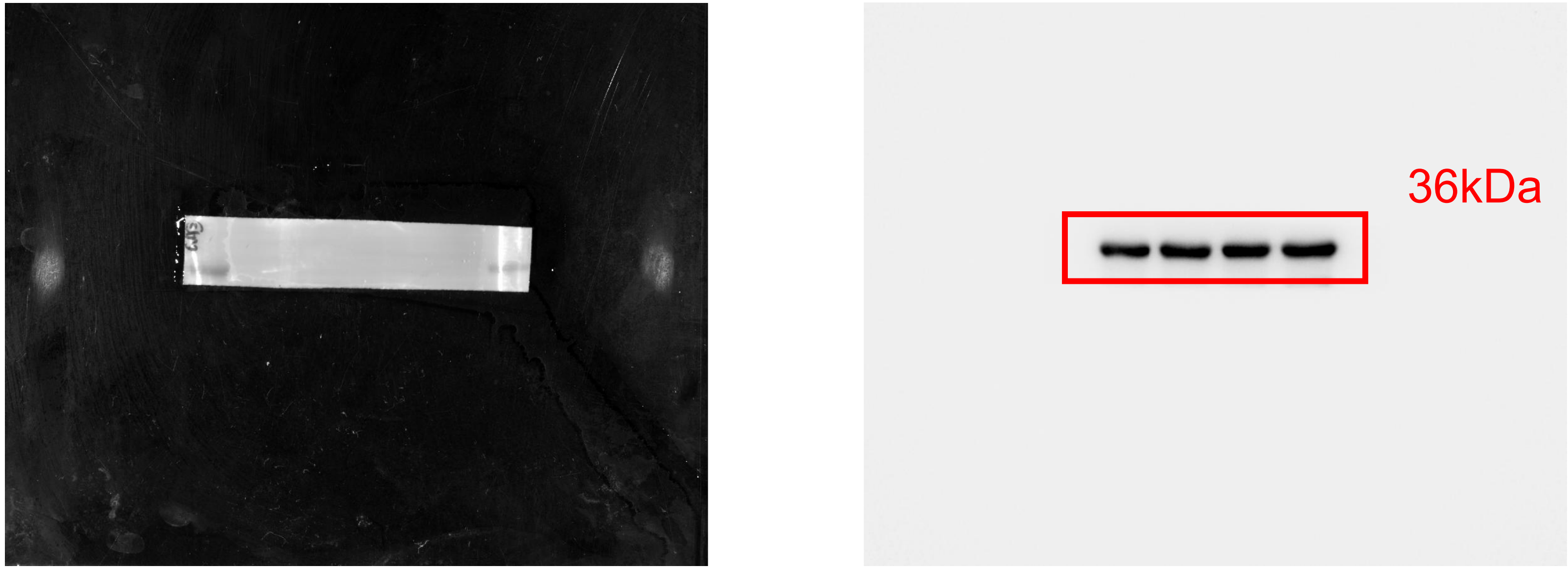


A549GAPDH

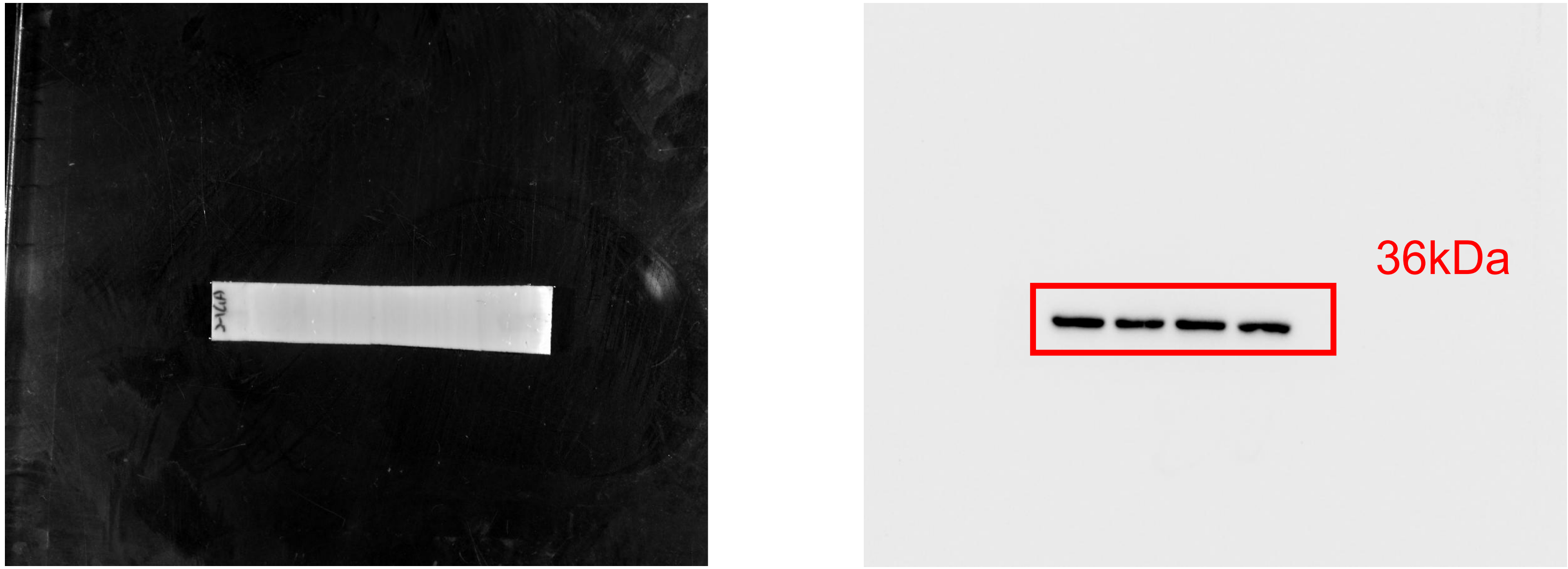

# S-4E

H1299 Bax

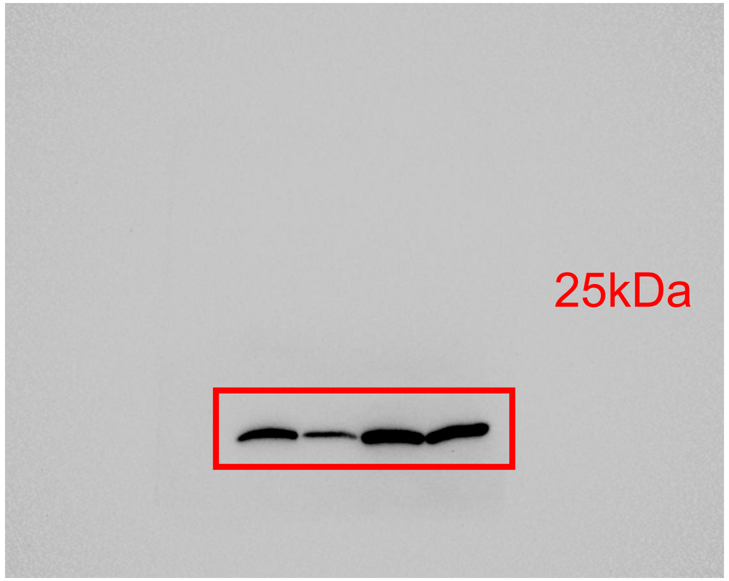


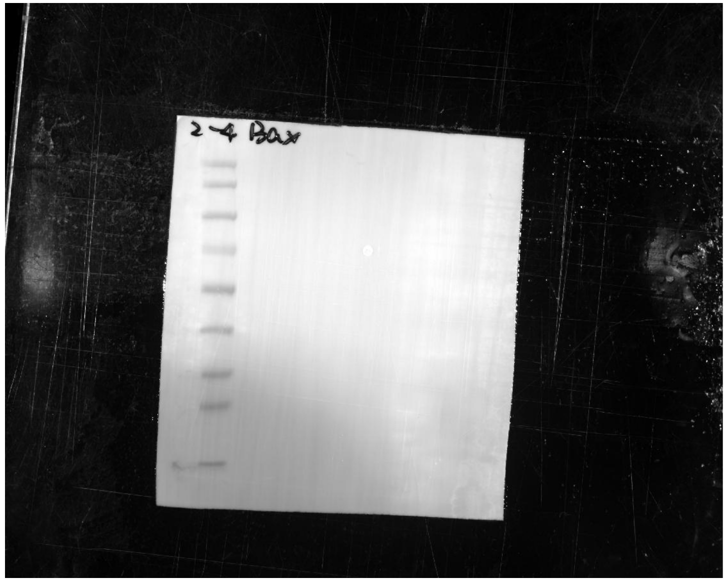

A549 Bax

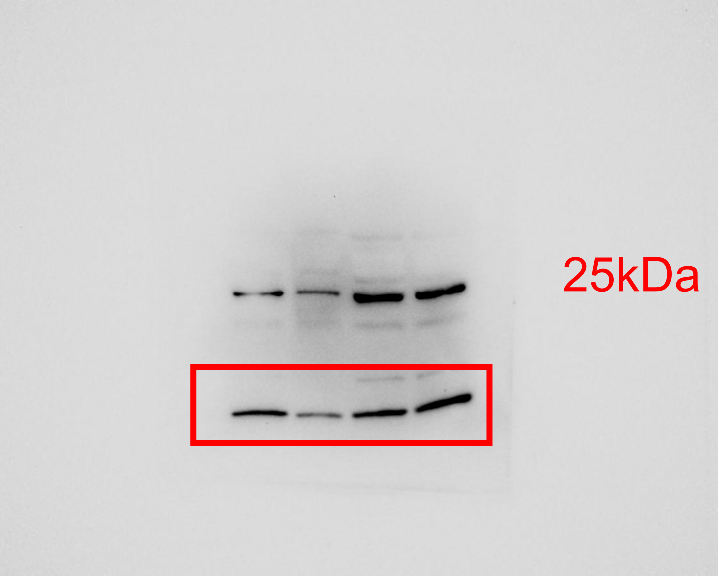


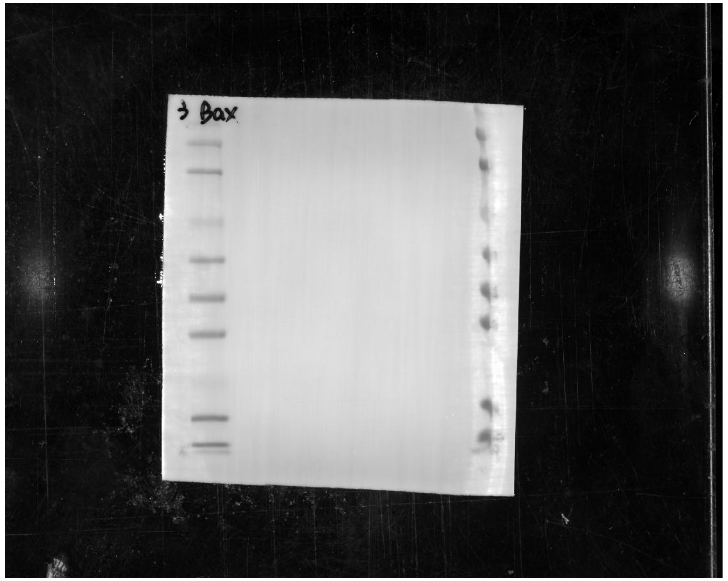

H1299 Bcl-2

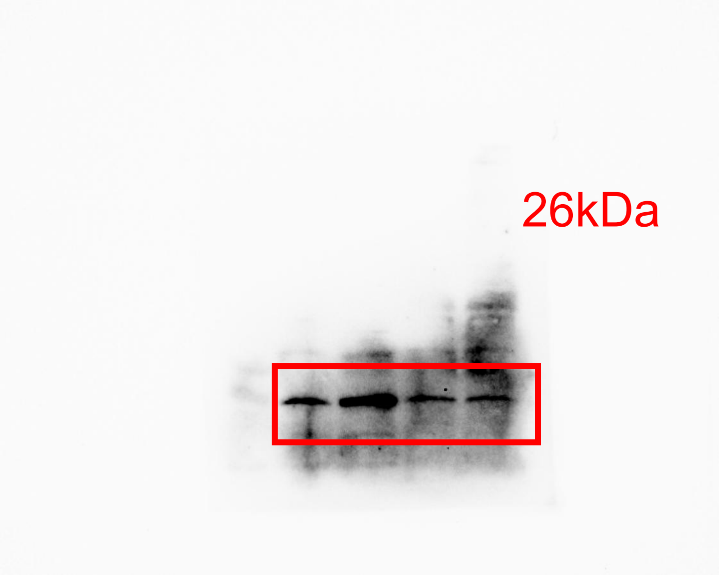


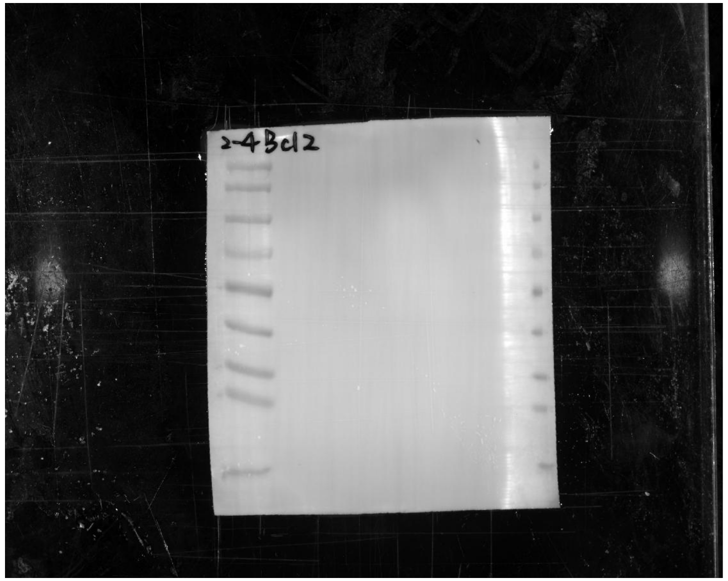

A549 Bcl-2

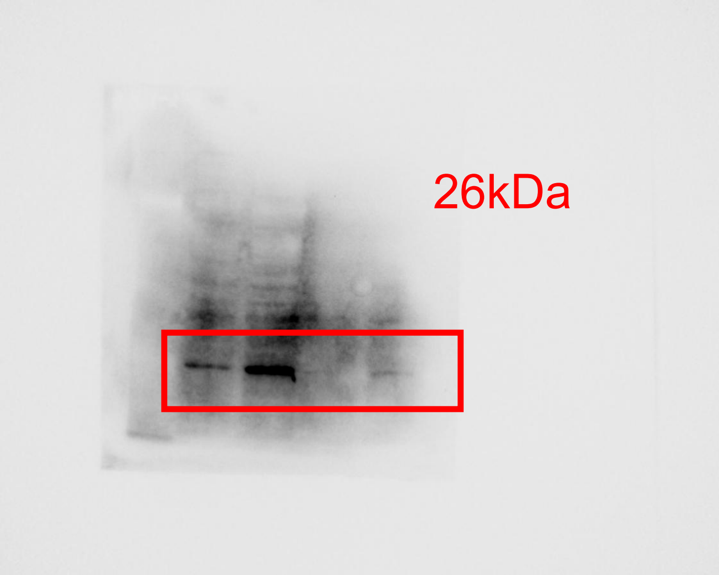


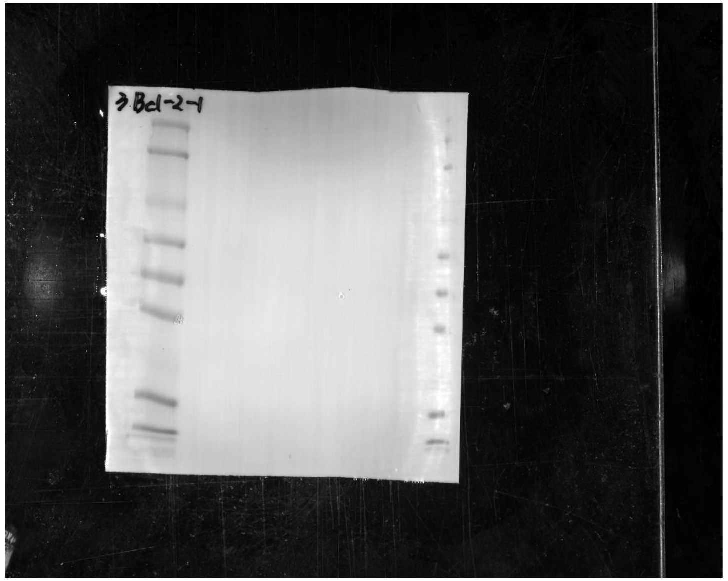

H1299
Caspase3

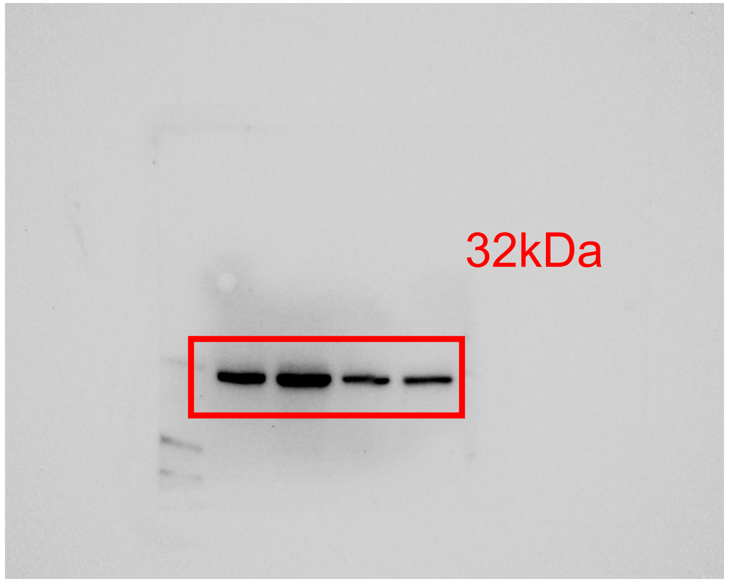


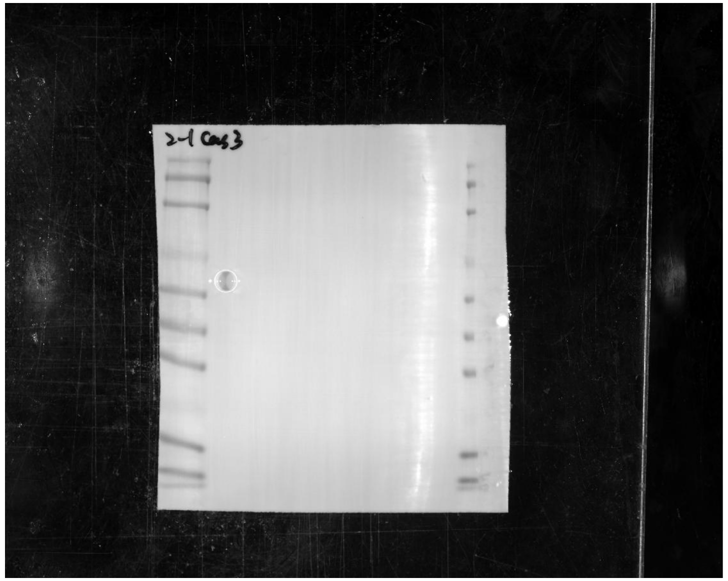

A549
Caspase3

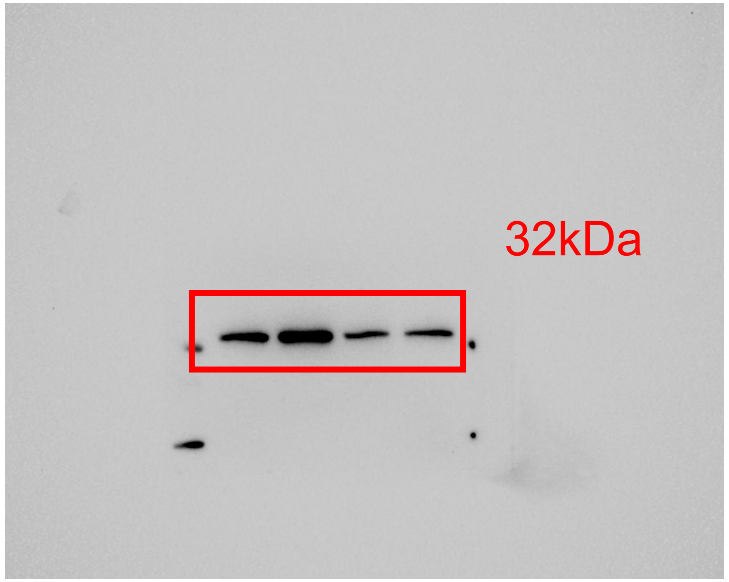


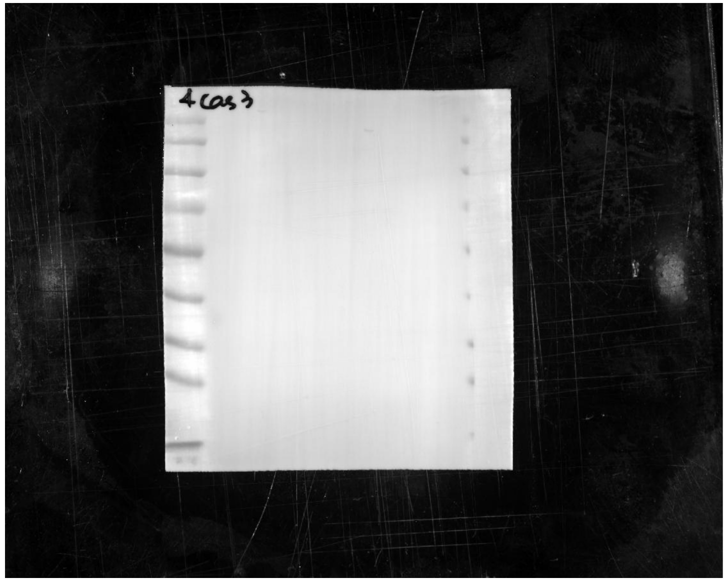

H1299
Cleaved-
Caspase3

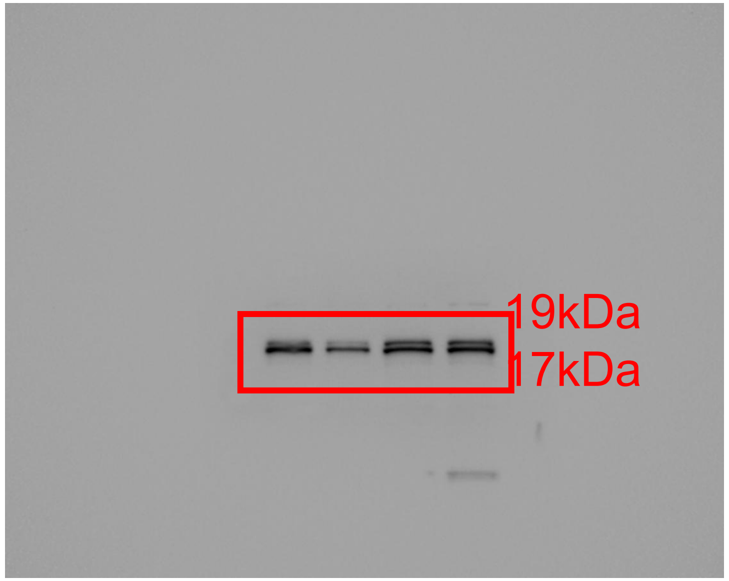


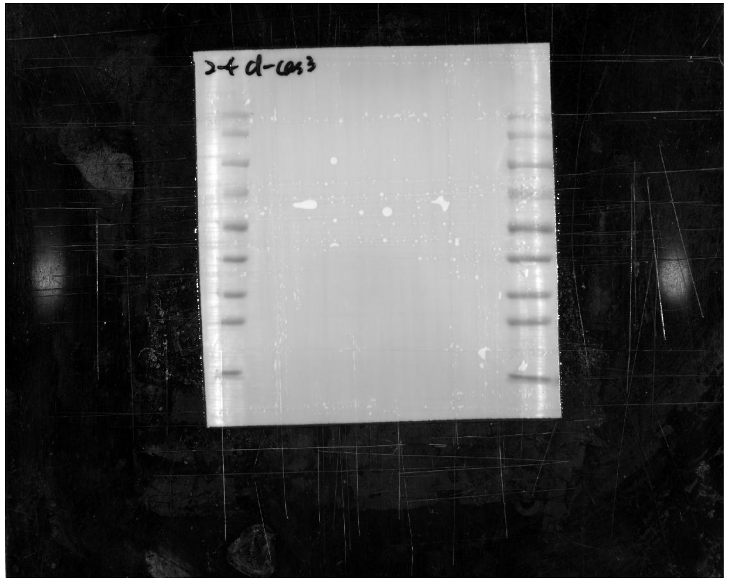

A549 Cleaved-
Caspase3

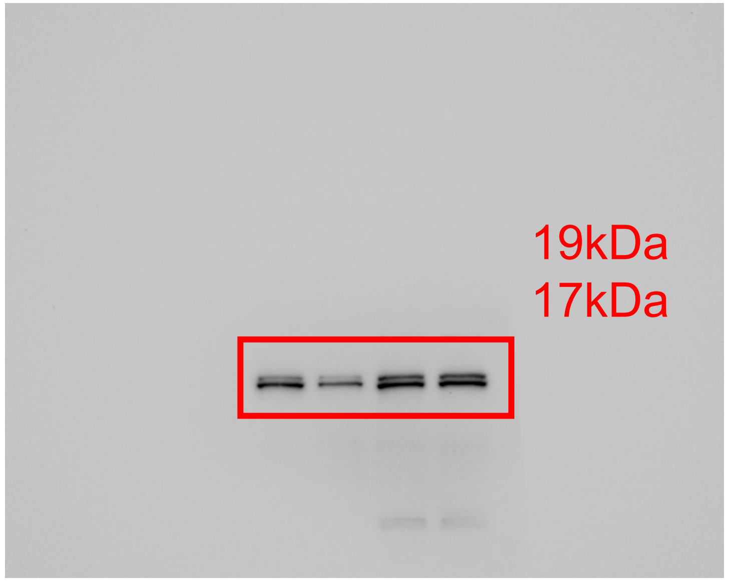


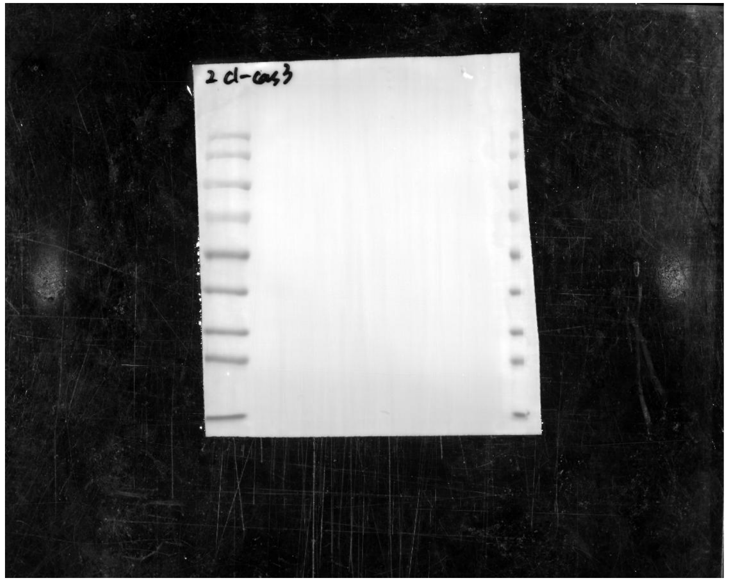

H1299
PARP
Cleaved-PARP

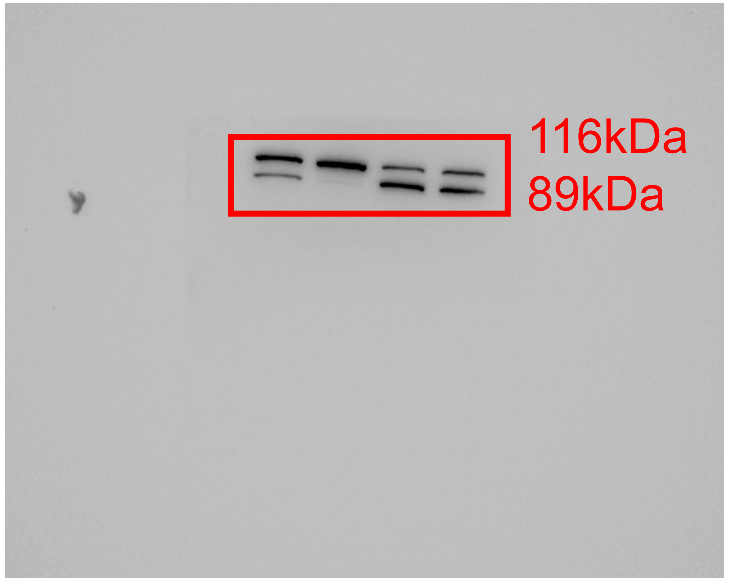


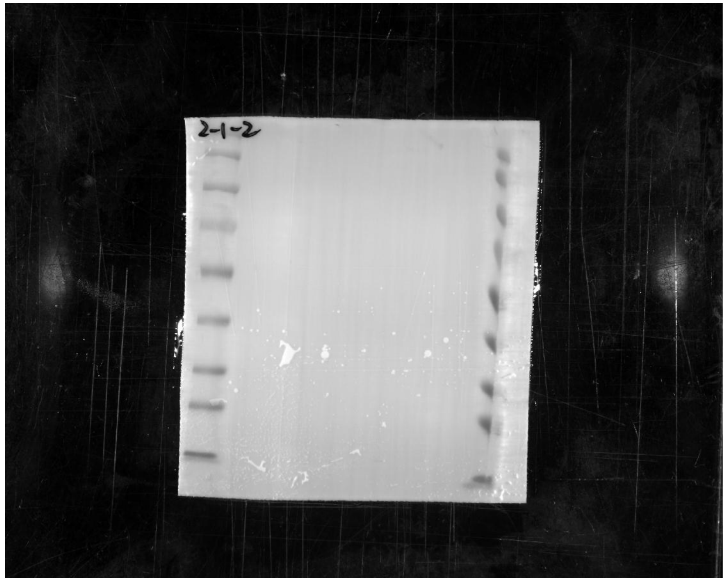

A549
PARP
Cleaved-PARP

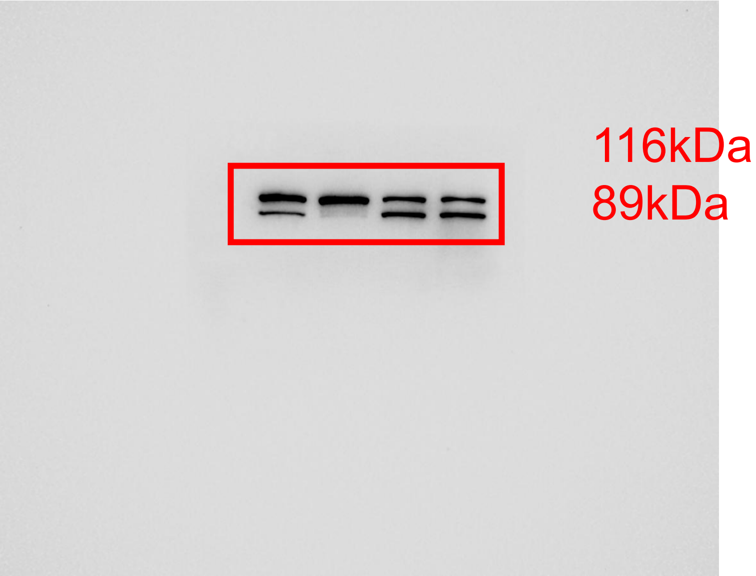


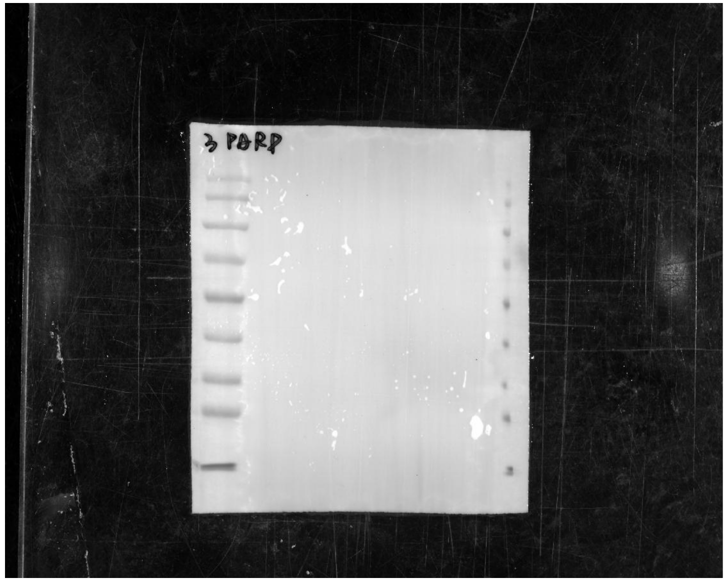

H1299 GAPDH

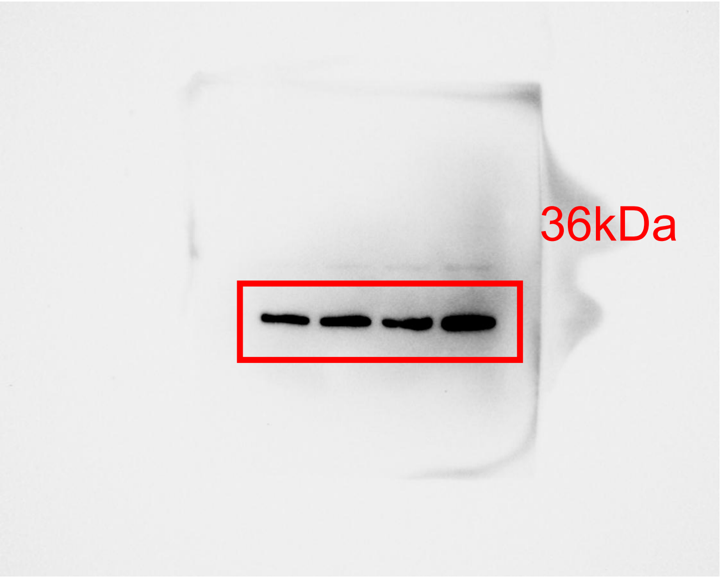


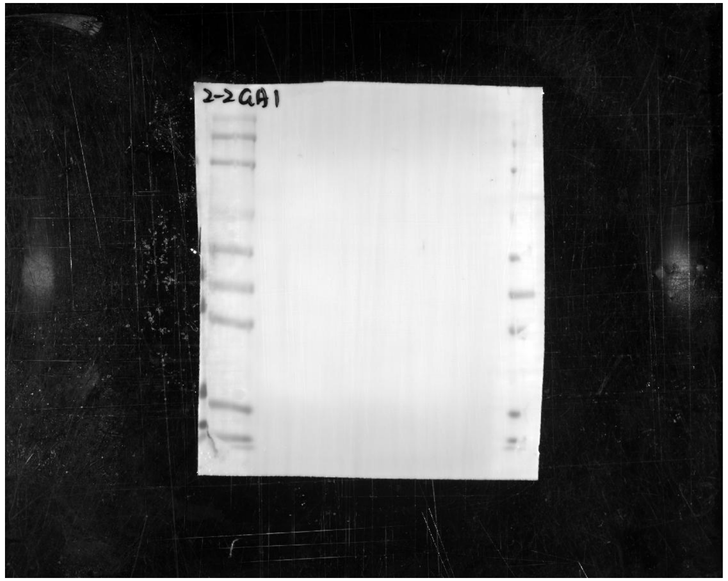

A549 GAPDH

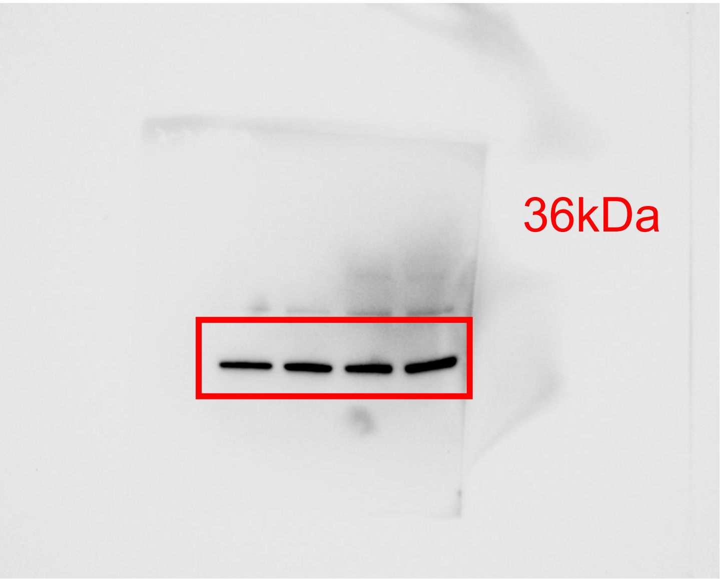


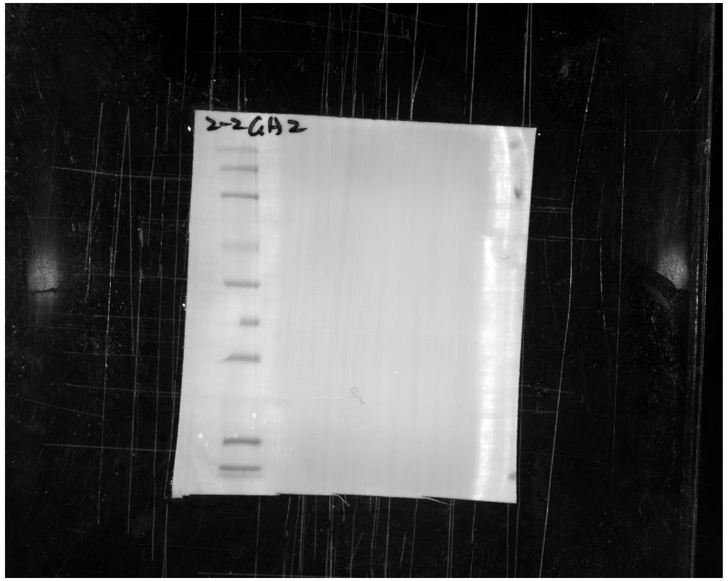

# S-Figure4F

H1299CDK4

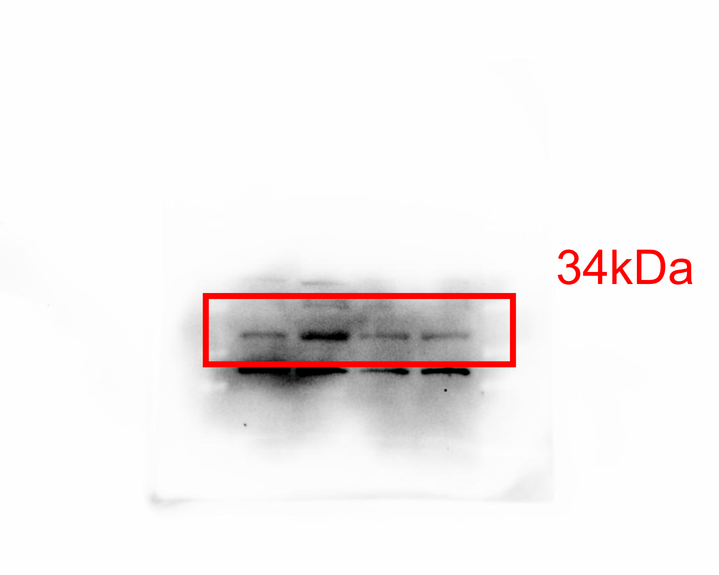

34kDa

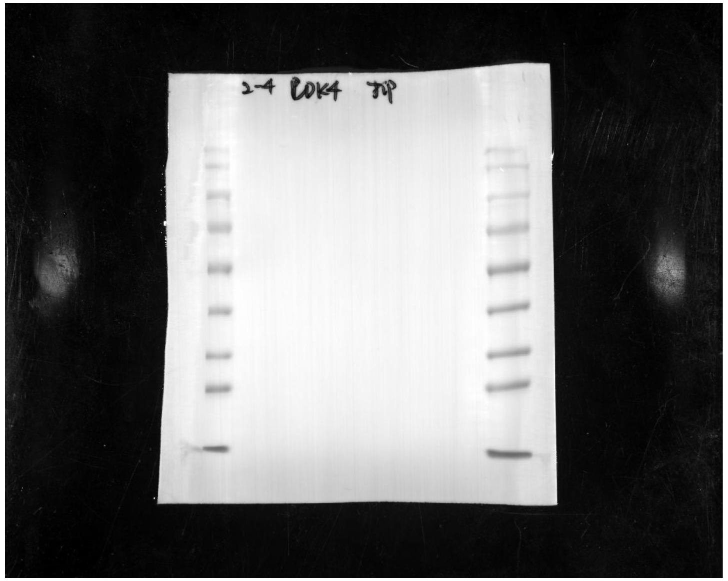

A549CDK4

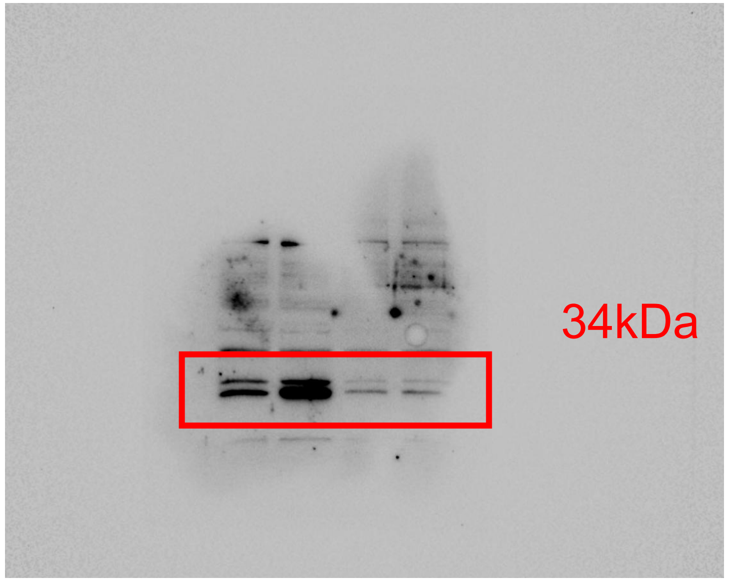

34kDa

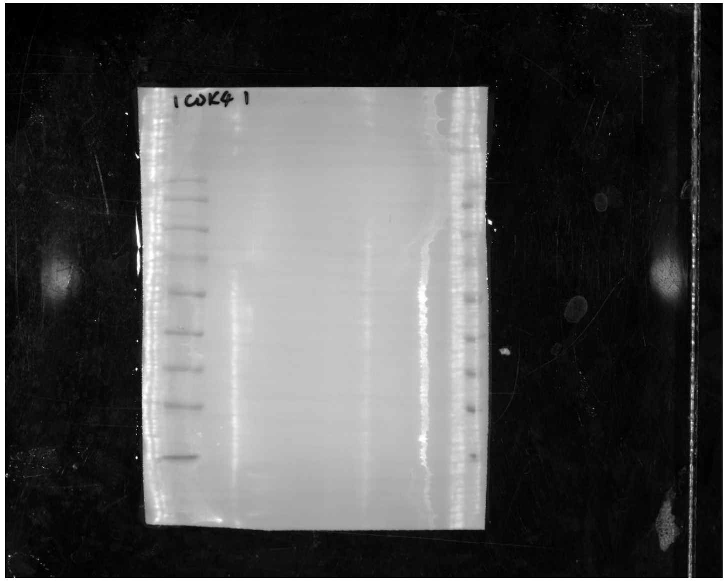

H1299CDK6

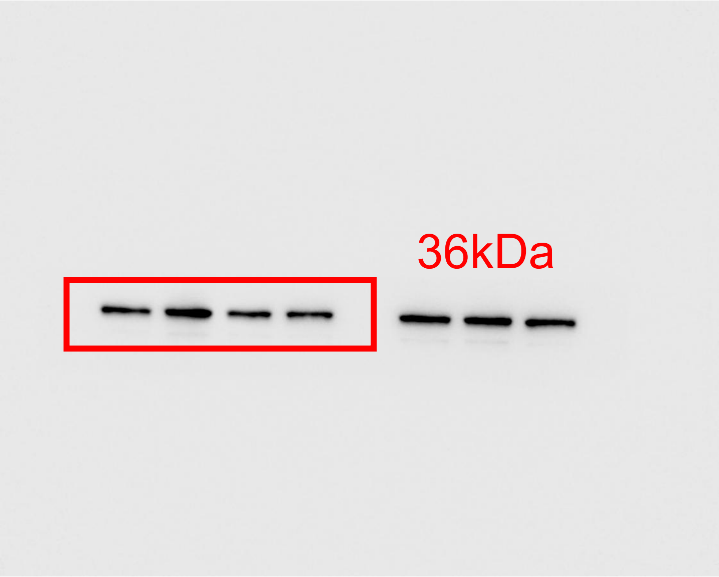


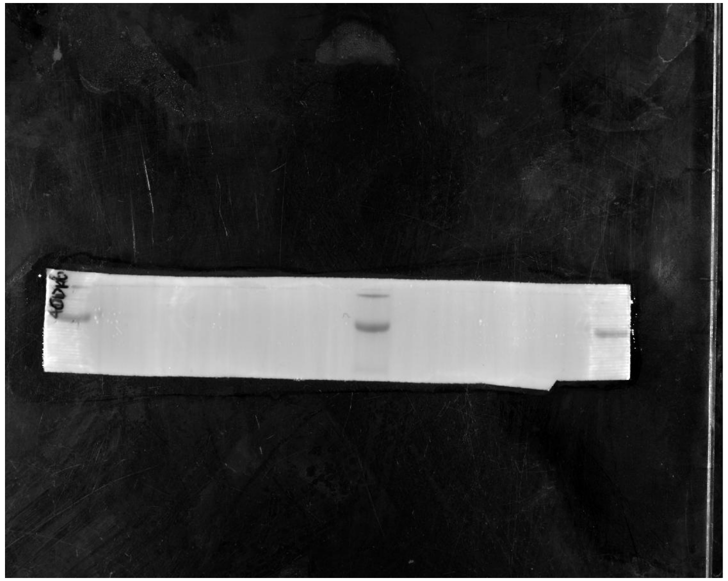

A549CDK6

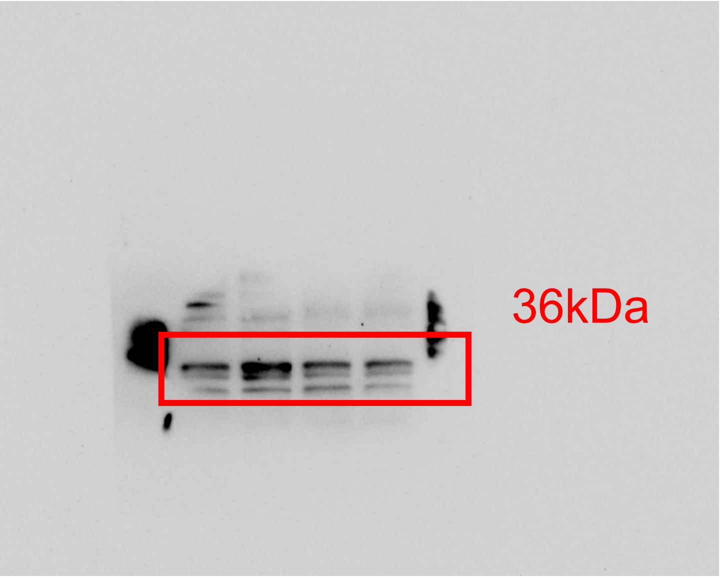


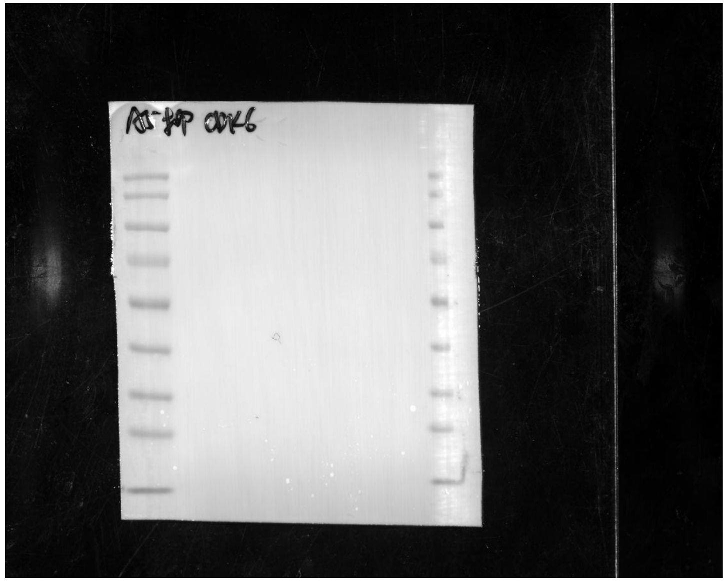

H1299A2

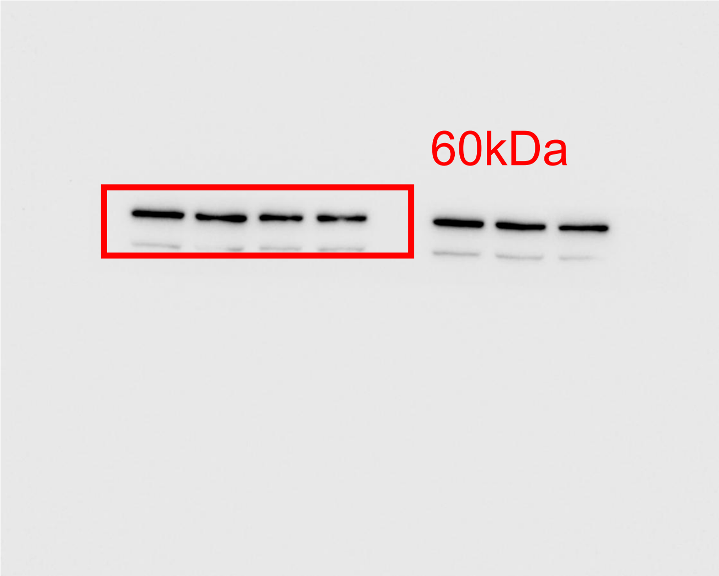


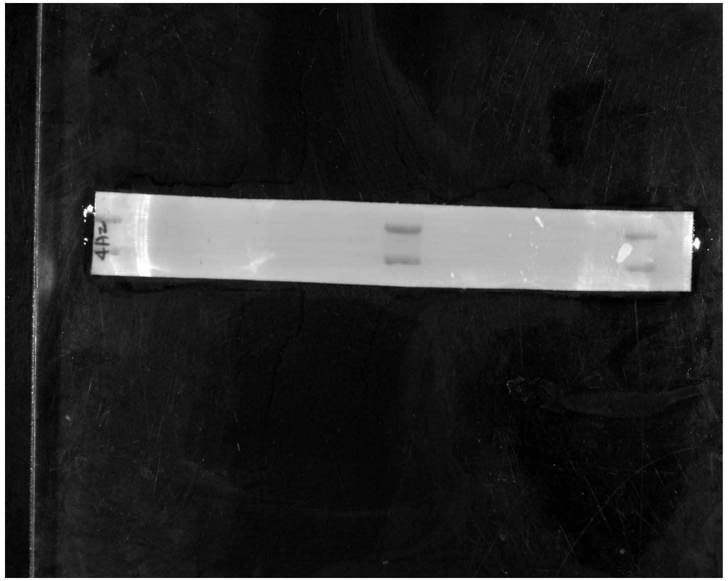

A549A2

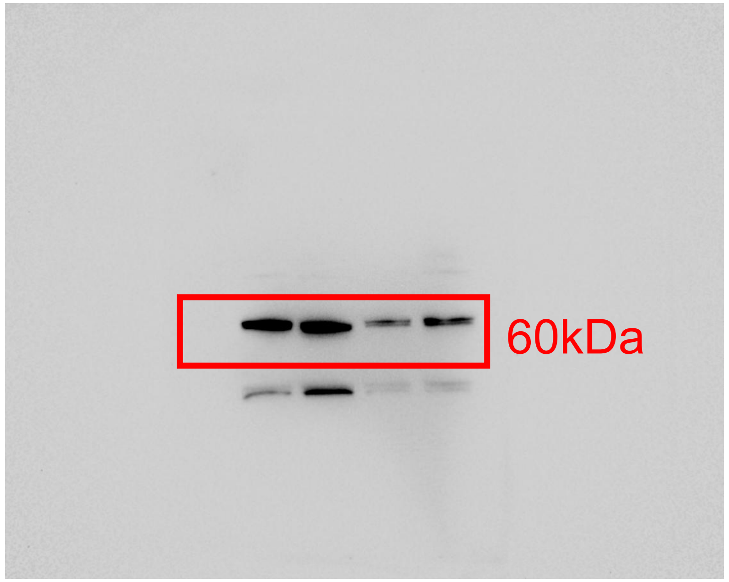


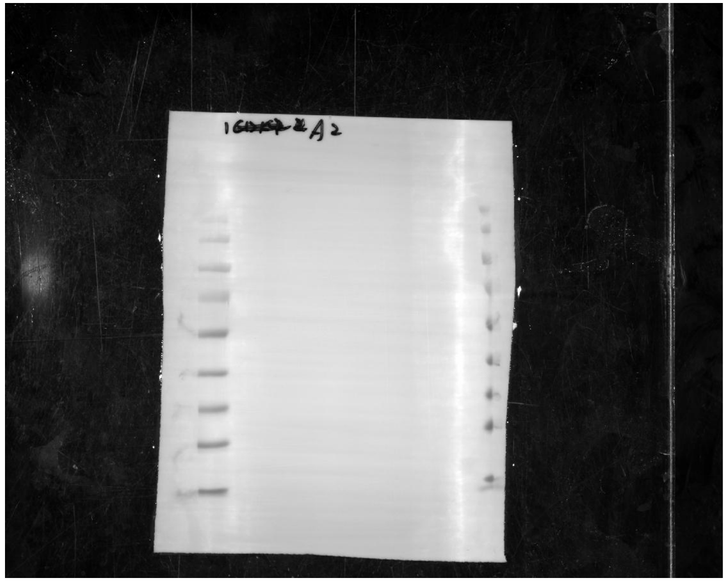

H1299D1

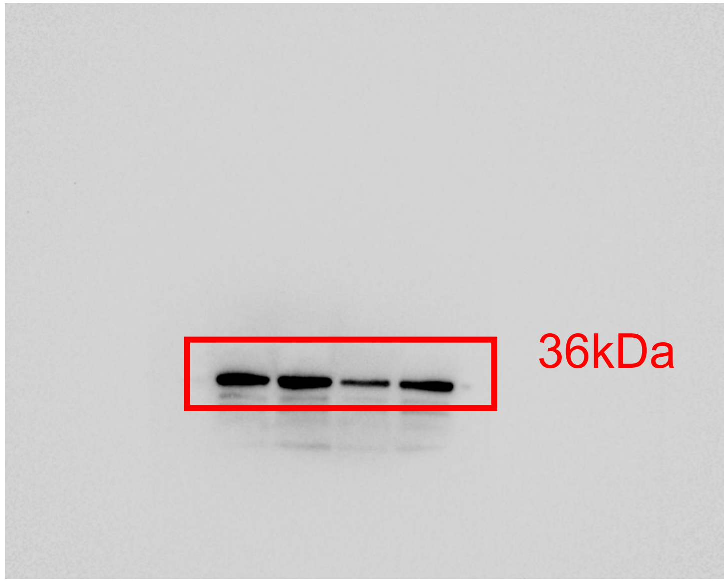


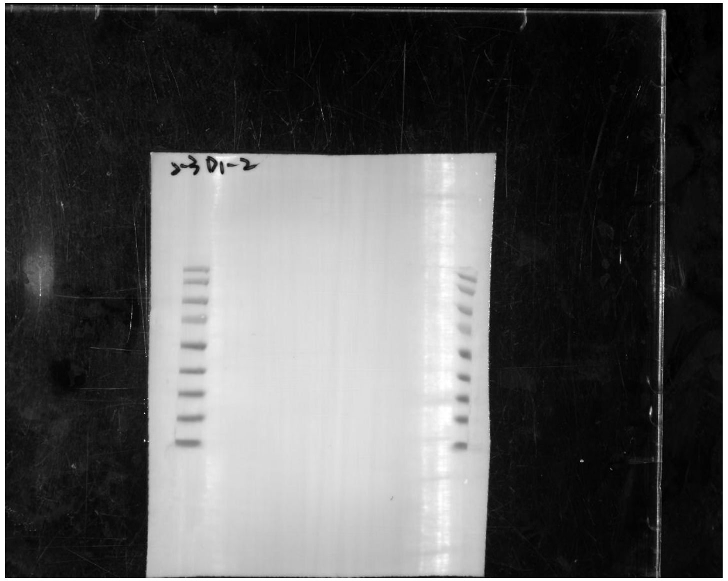

A549D1

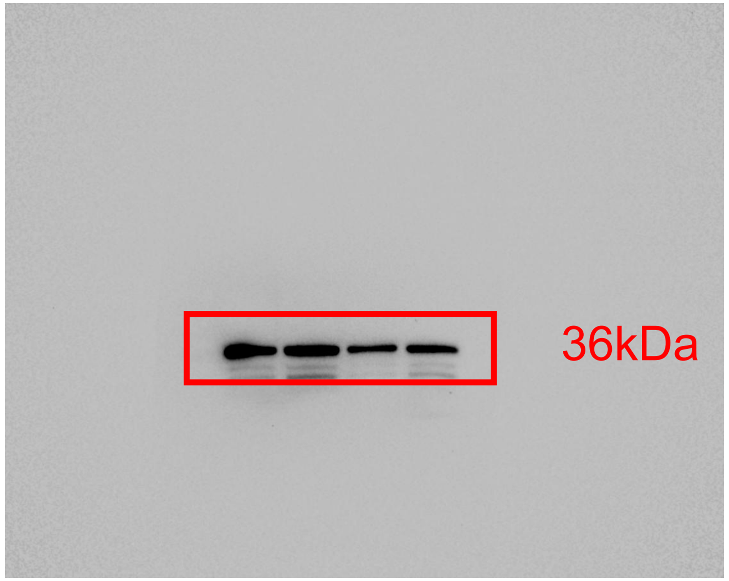


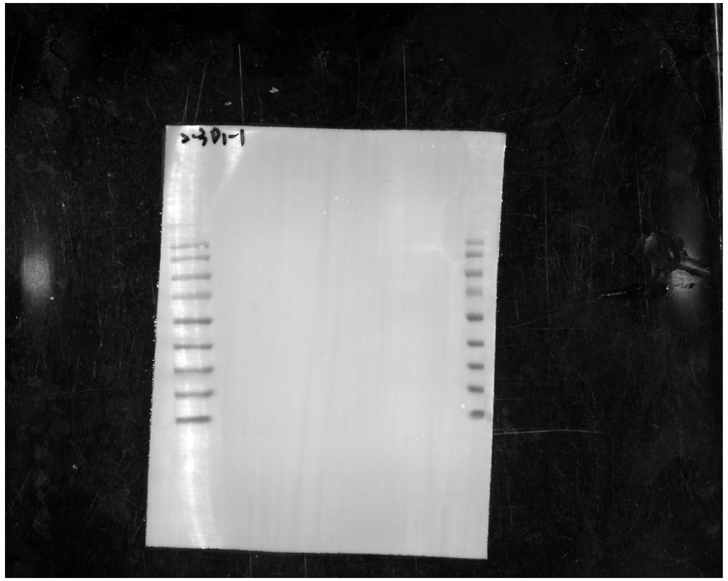

H1299N-ca

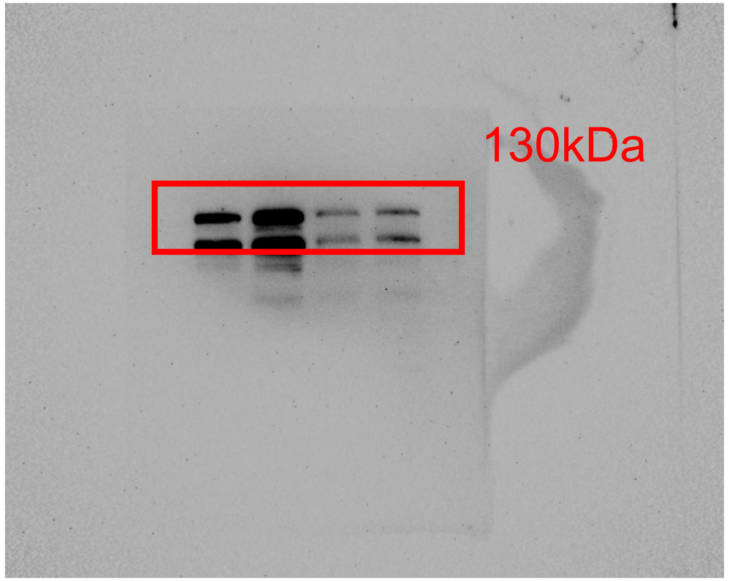


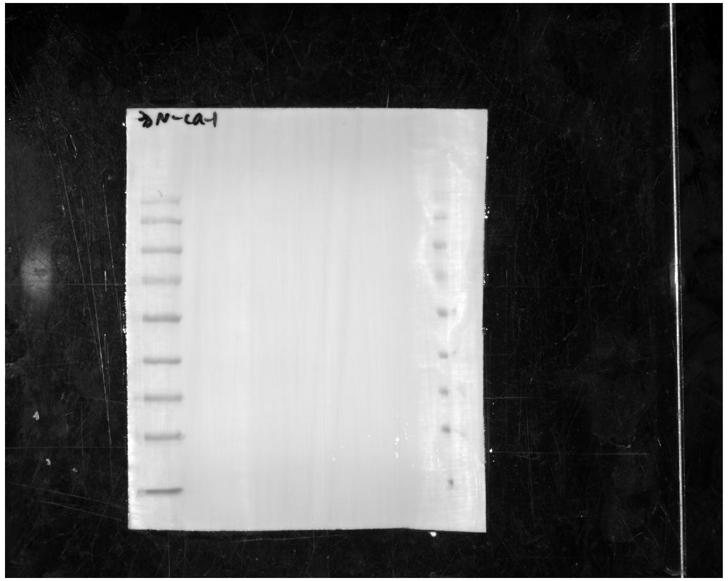

A549N-ca

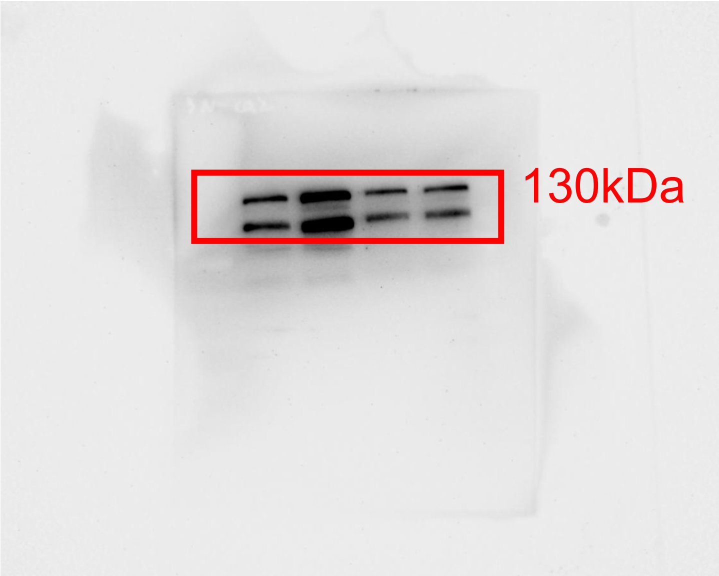


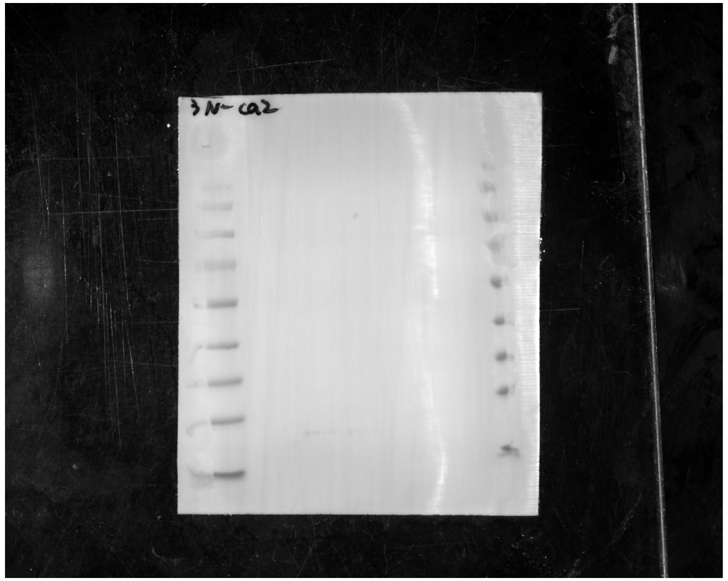

H1299E-ca

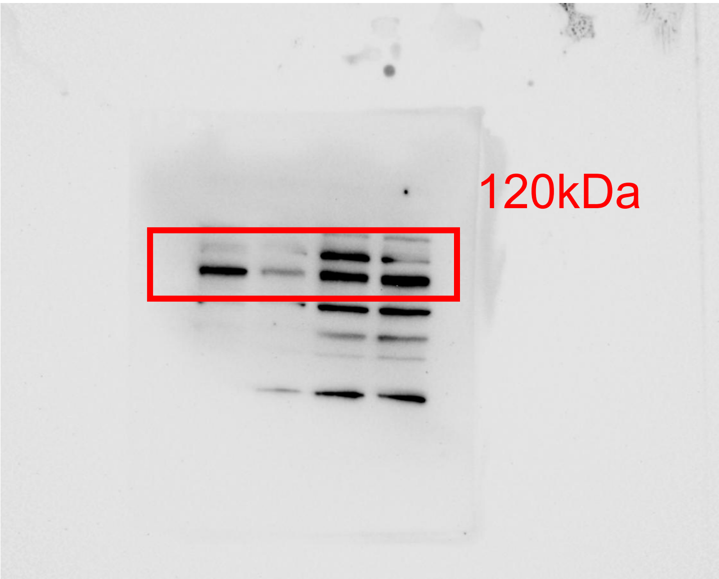


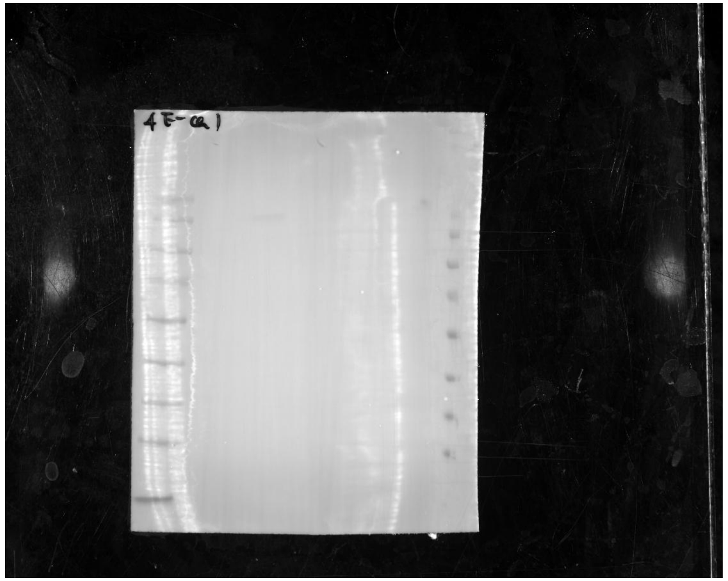

A549E-ca

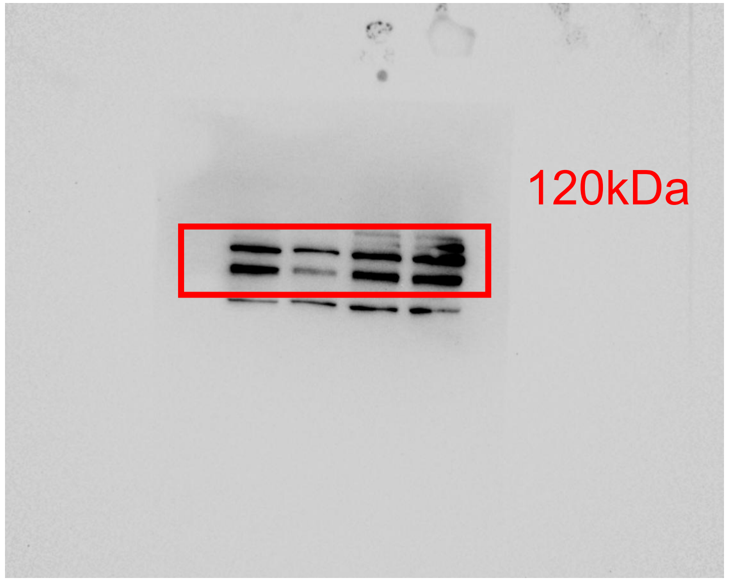


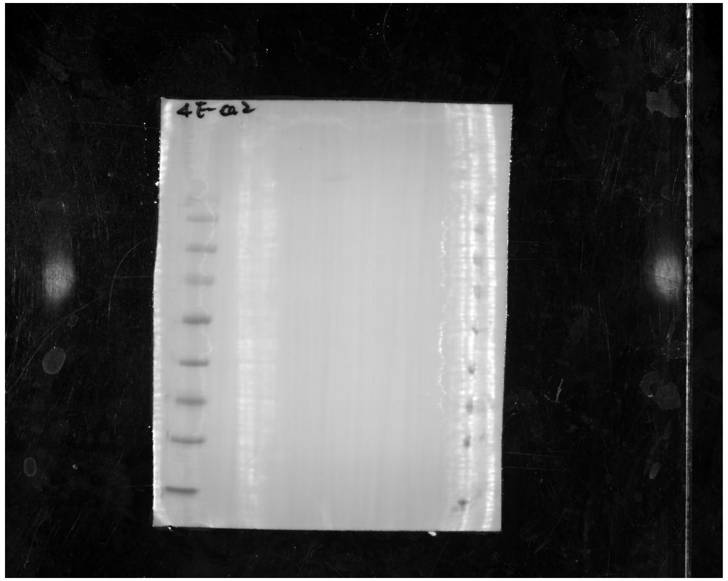

H1299mmp9

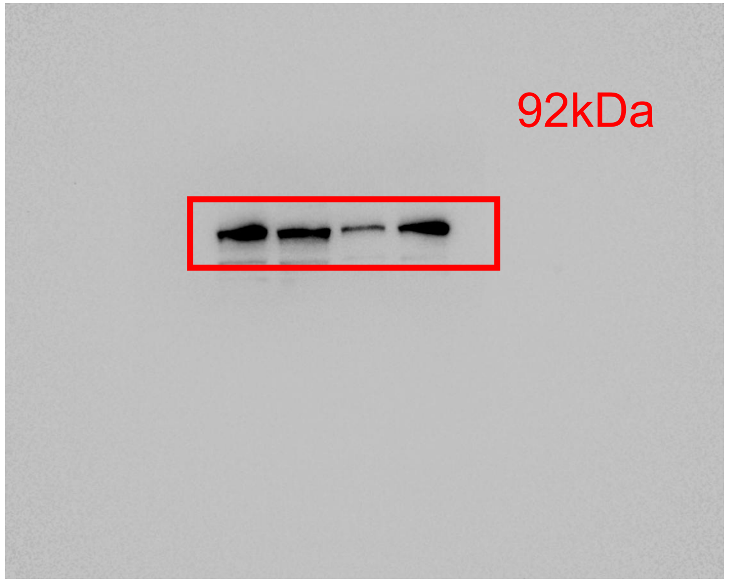


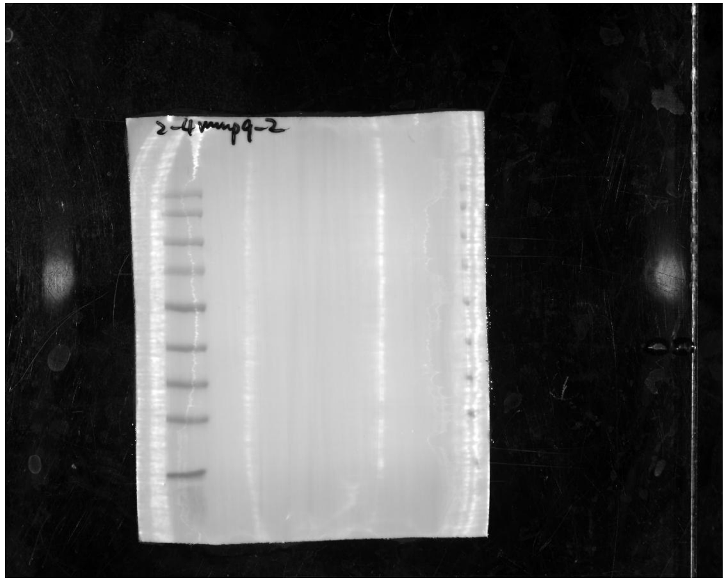

A549mmp9

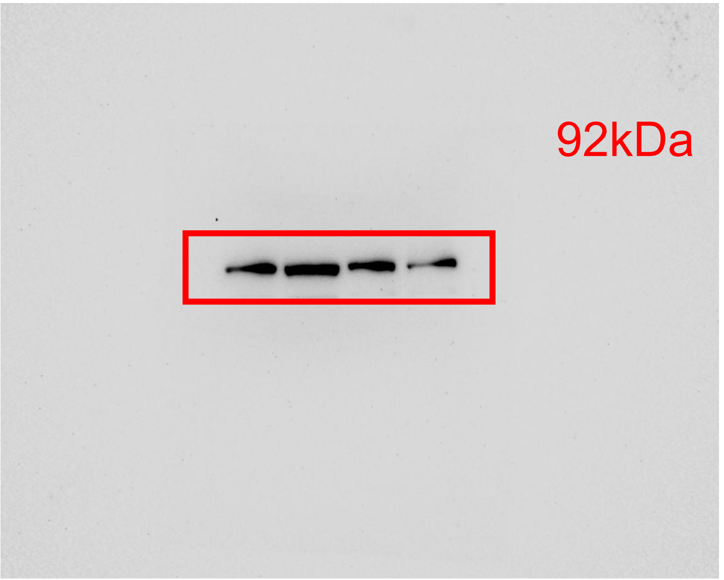


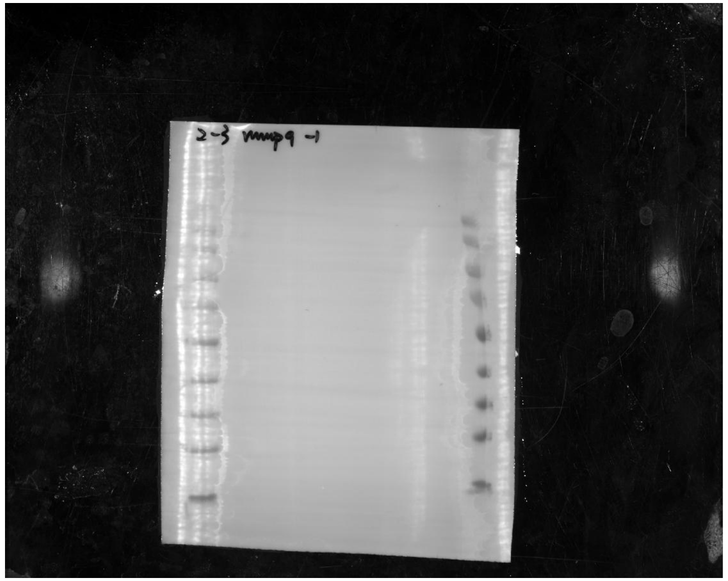

H1299GAPDH

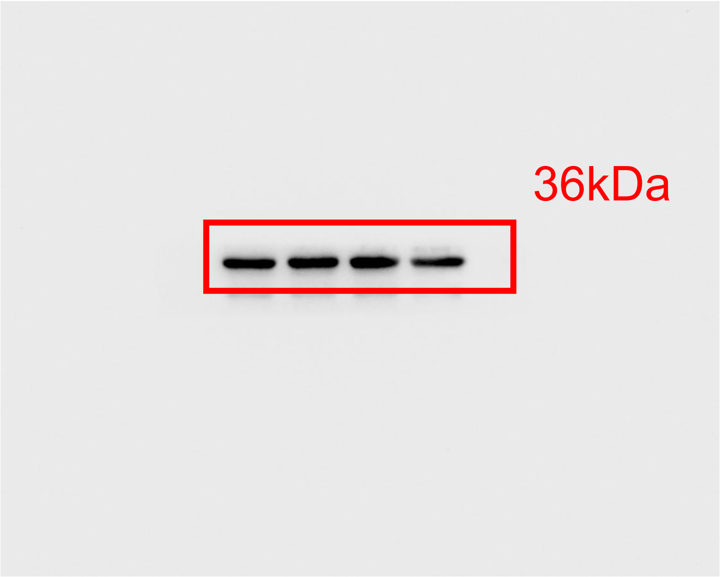


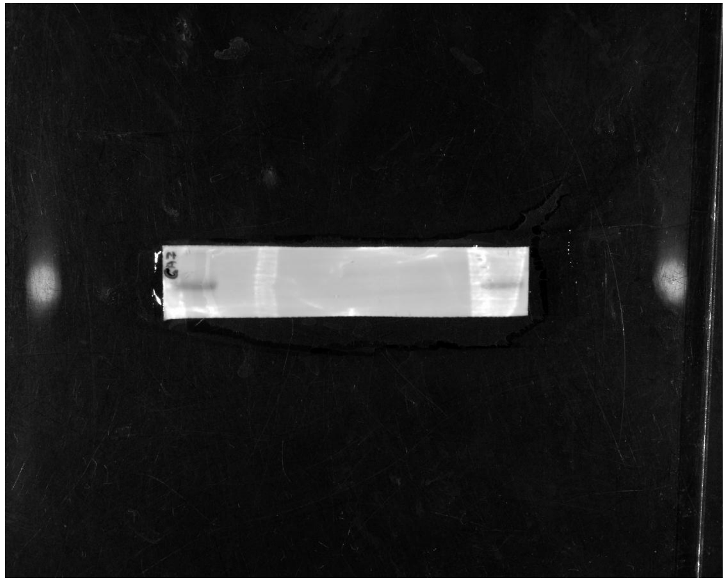

A549GAPDH

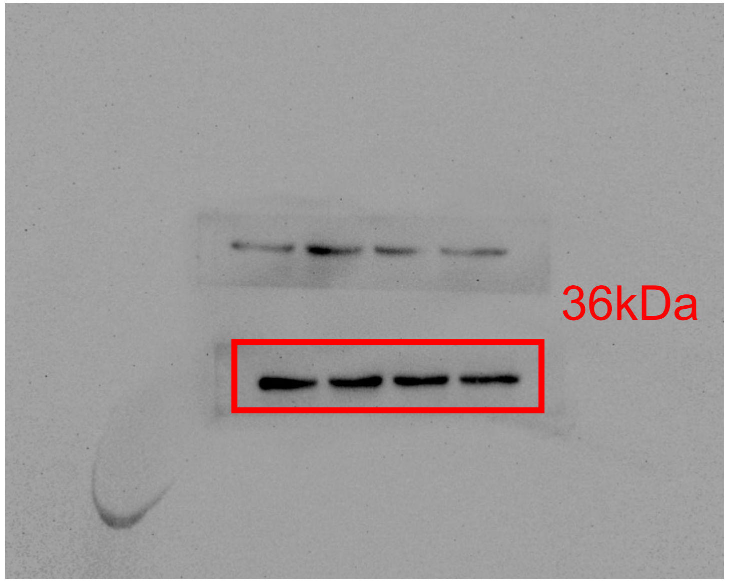


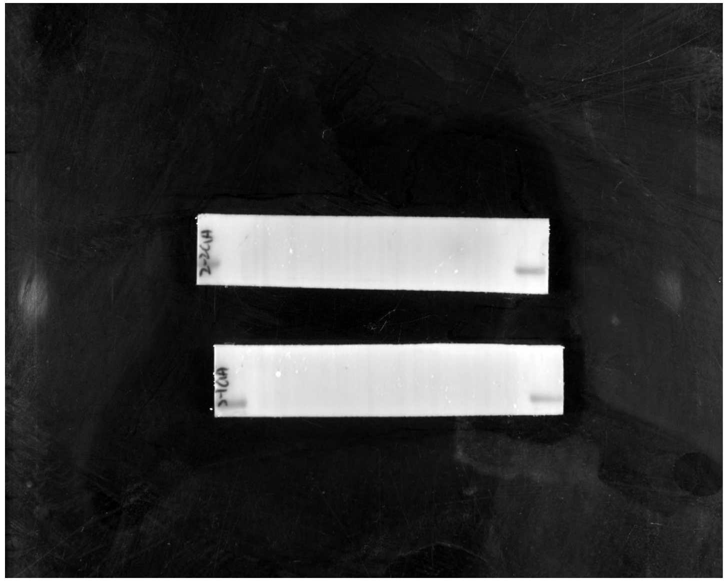

# S-4F

H1299 Zo-1

A549 Zo-1

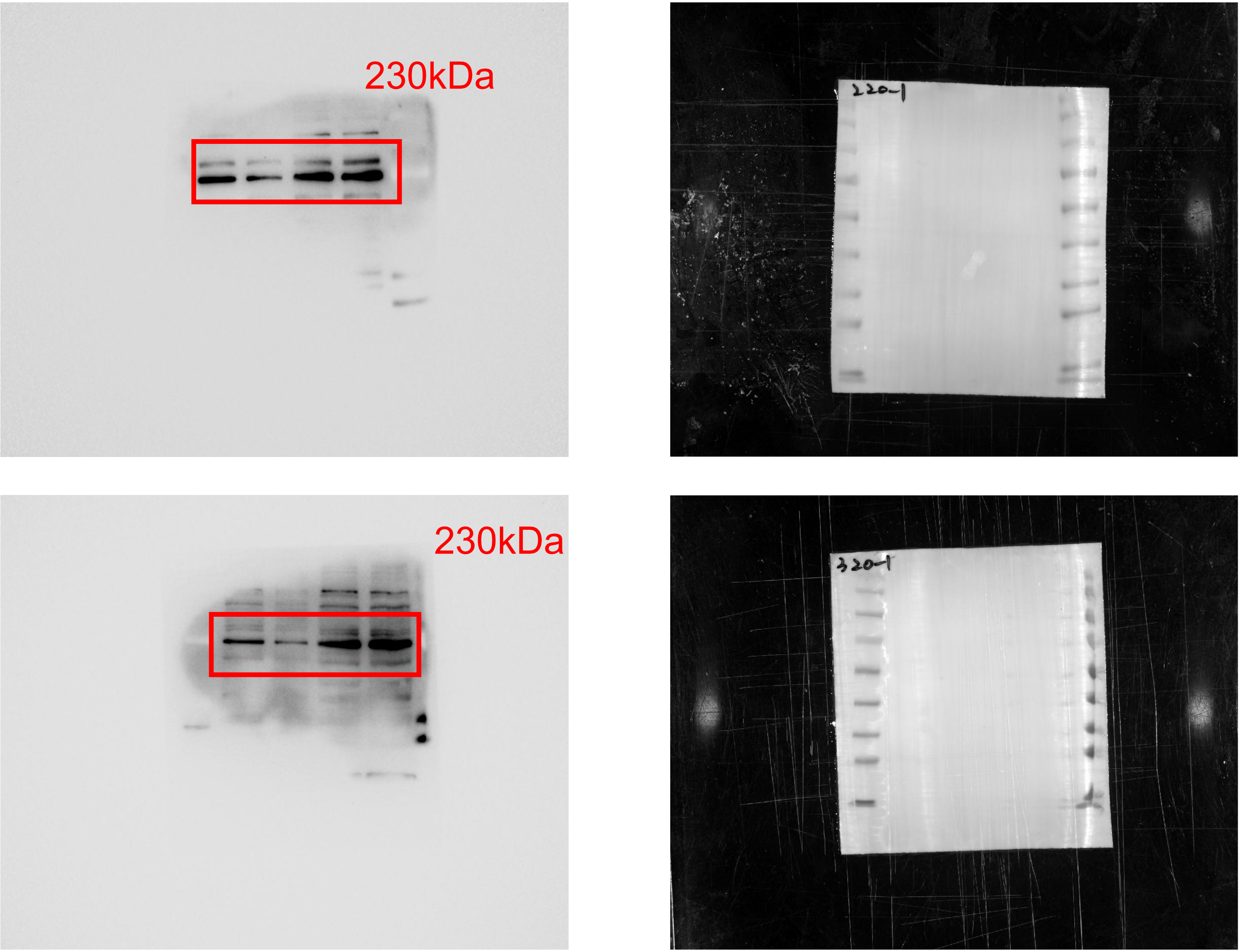

H1299
vimentin

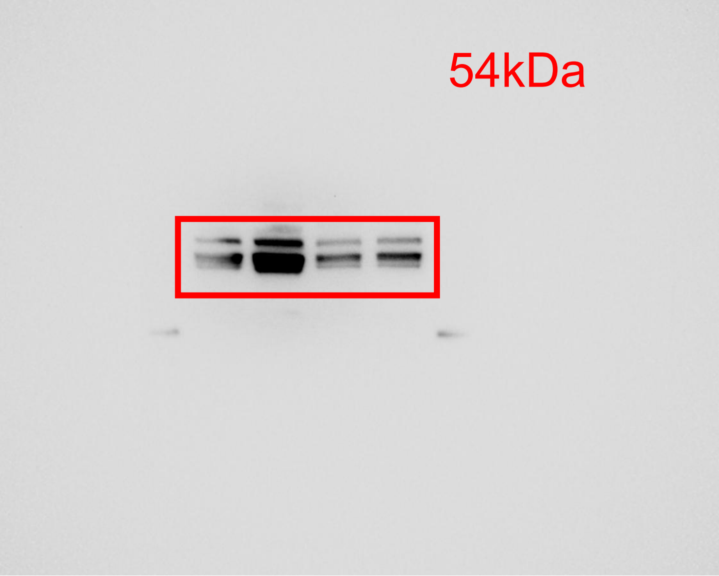


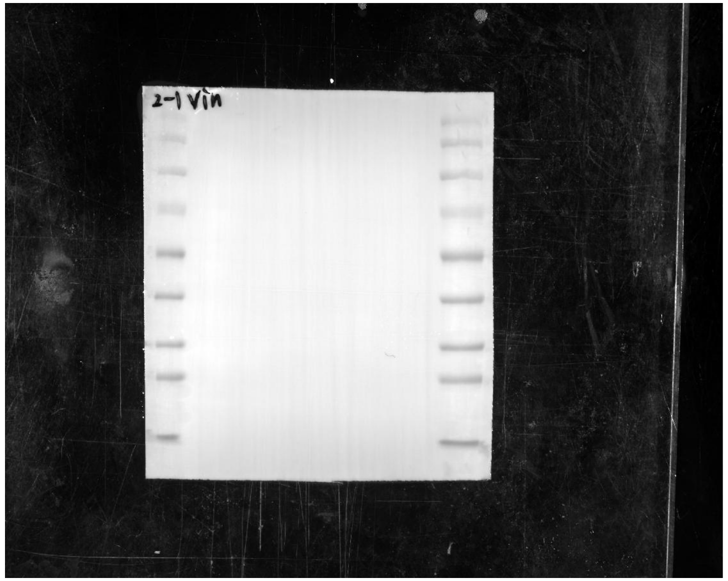


A549 vimentin

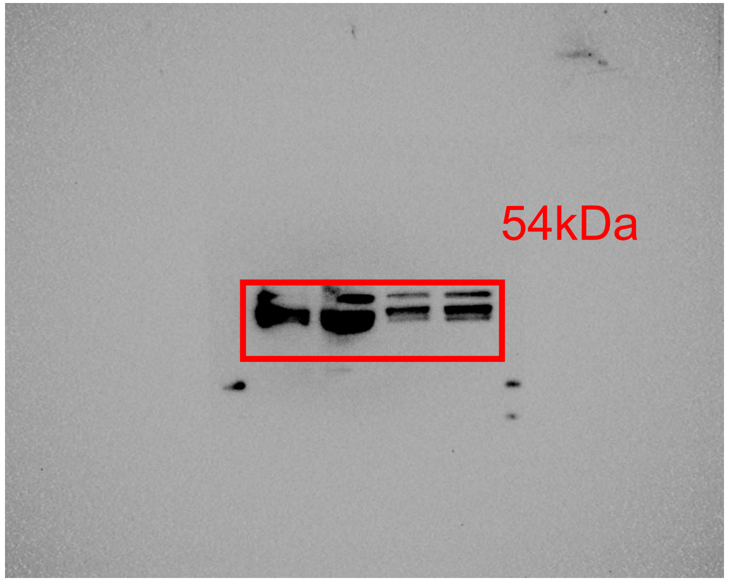


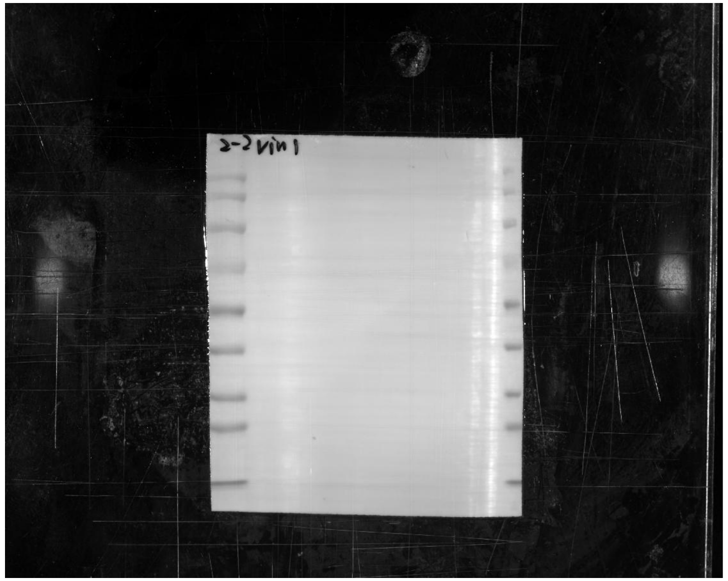

H1299 mmp2

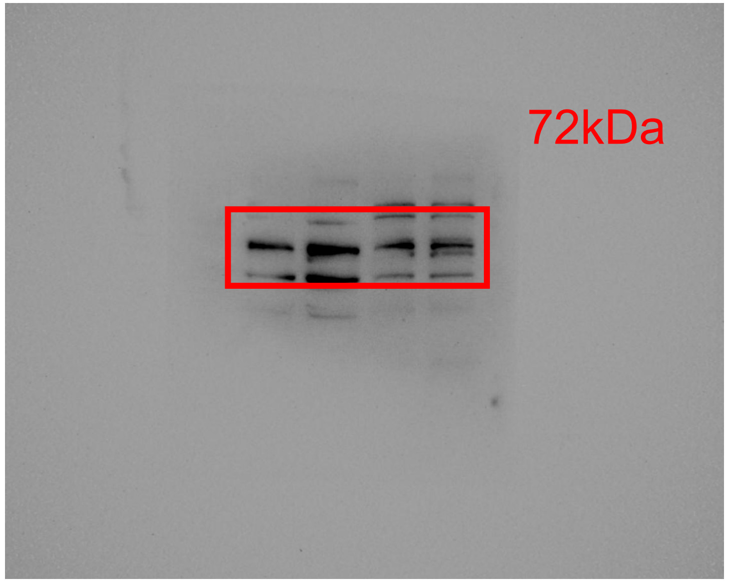


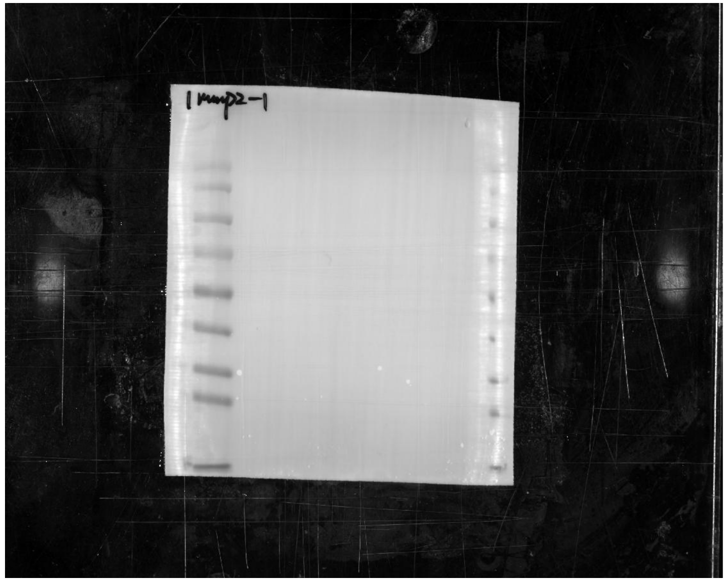

A549 mmp2

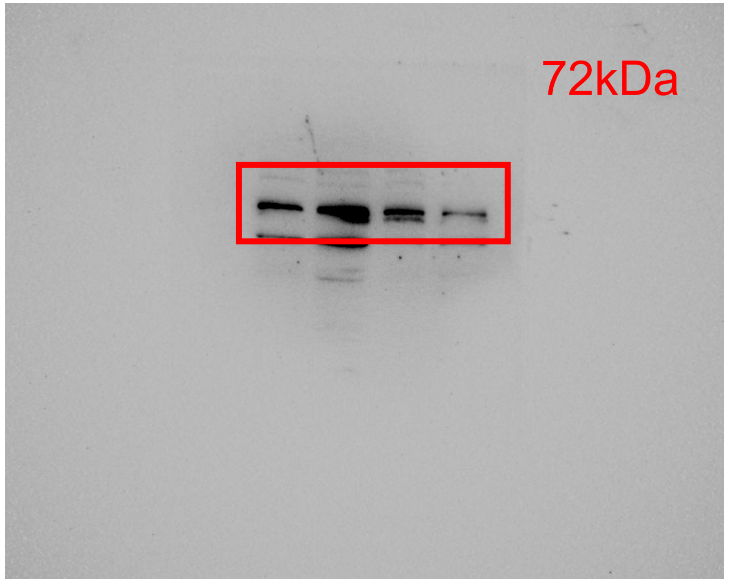


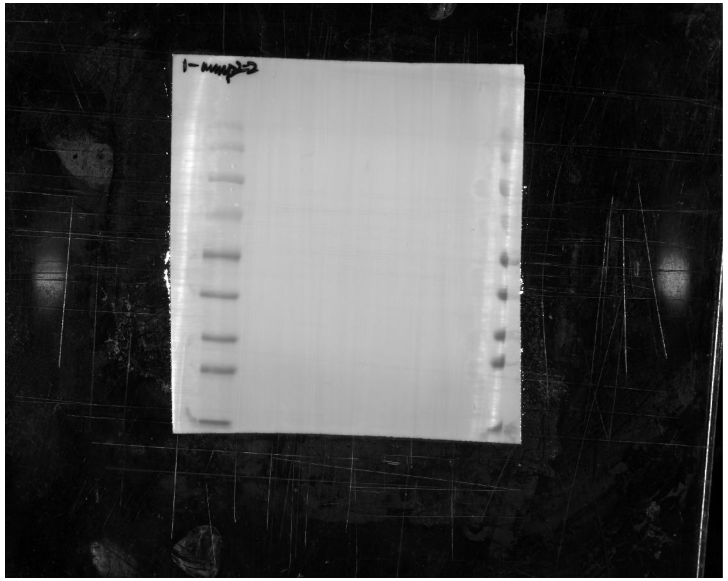

# Figure5B

IP-myc

H1299IB-ACTN4

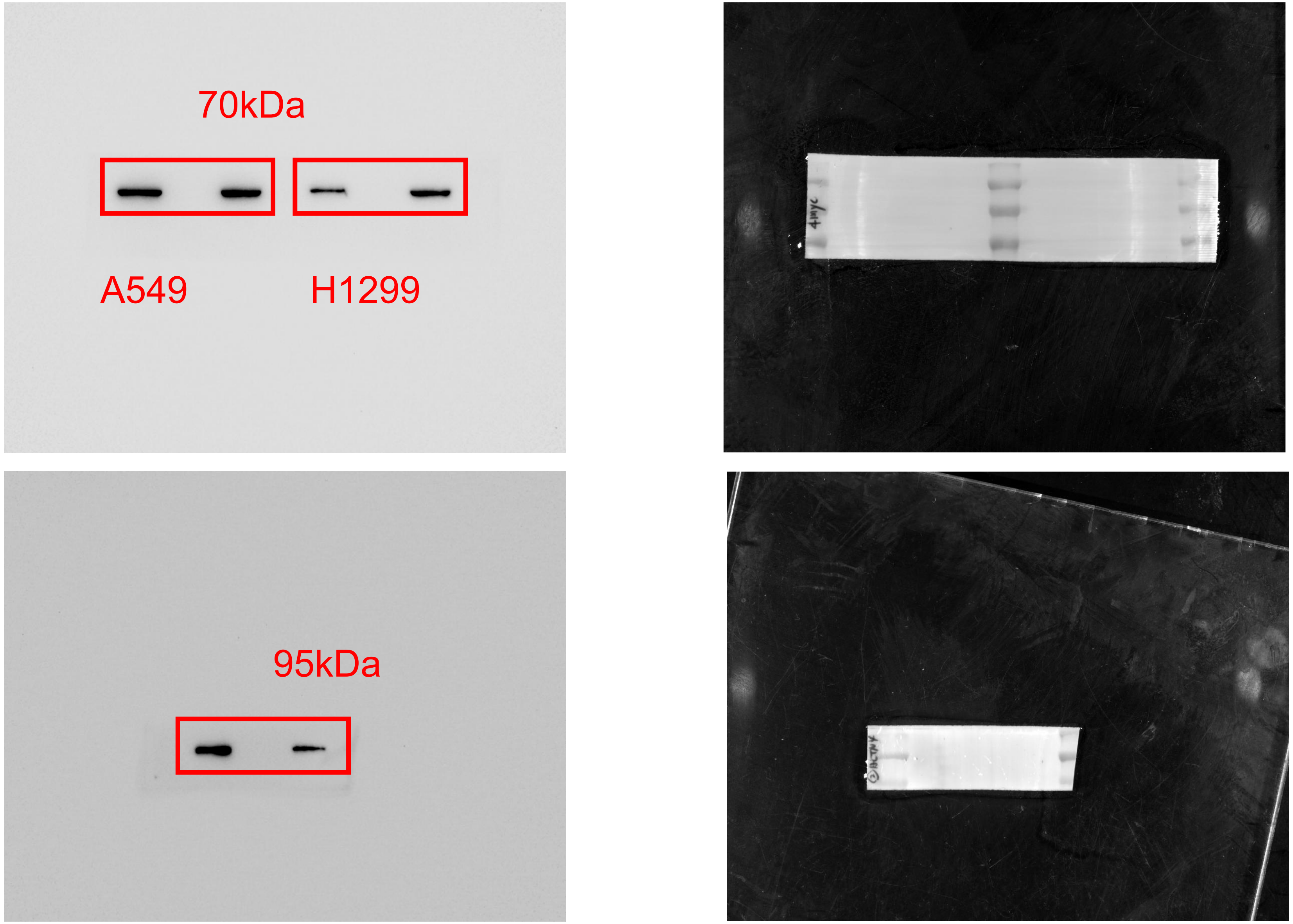

A549IB-ACTN4

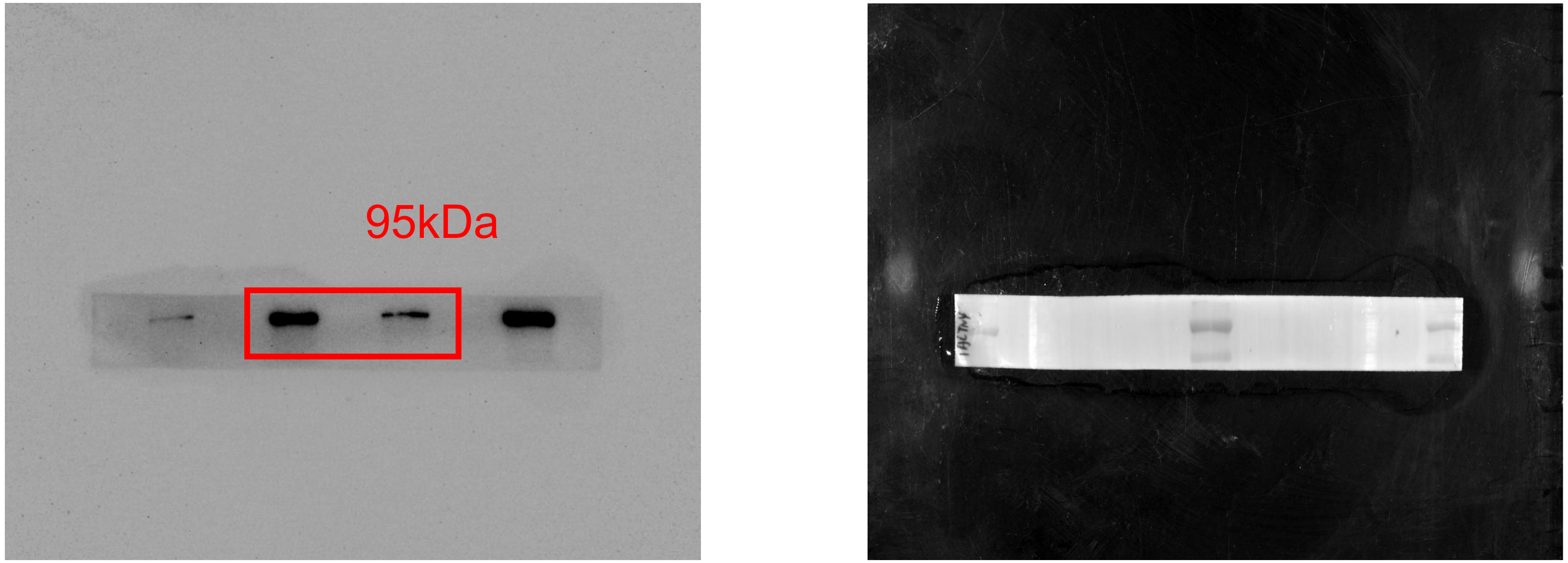


IP-ACTN4

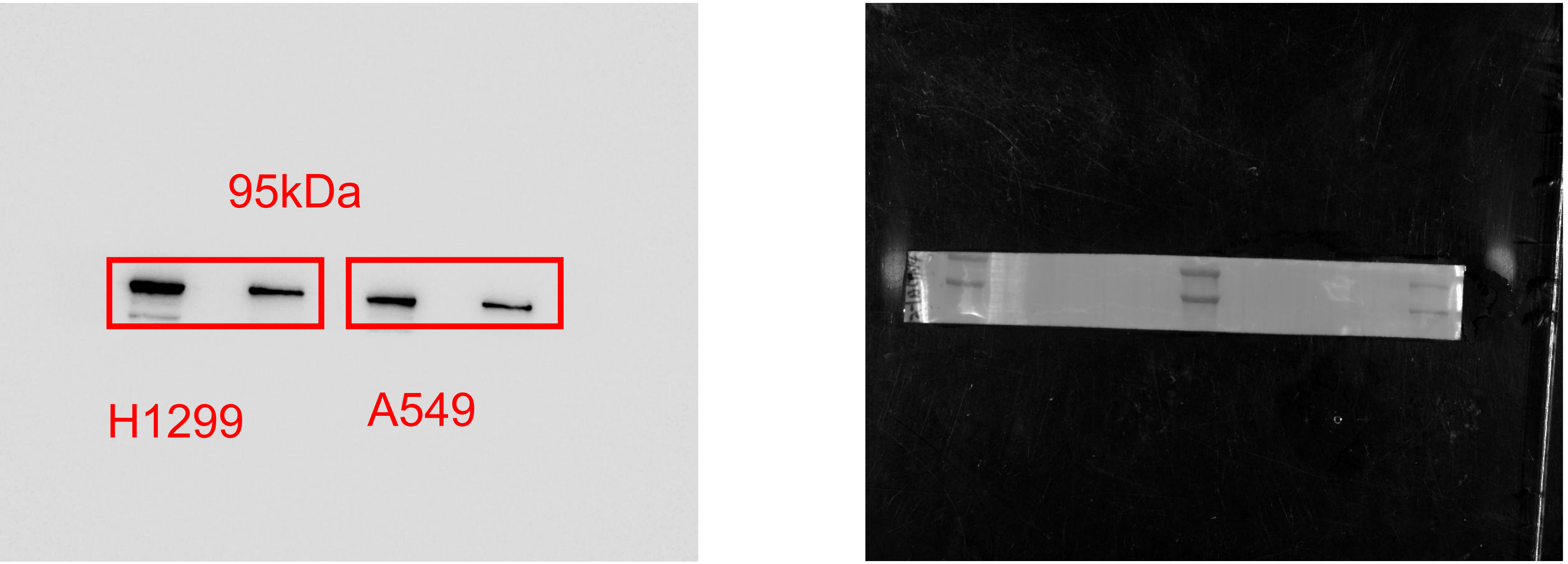

IB-PHF23

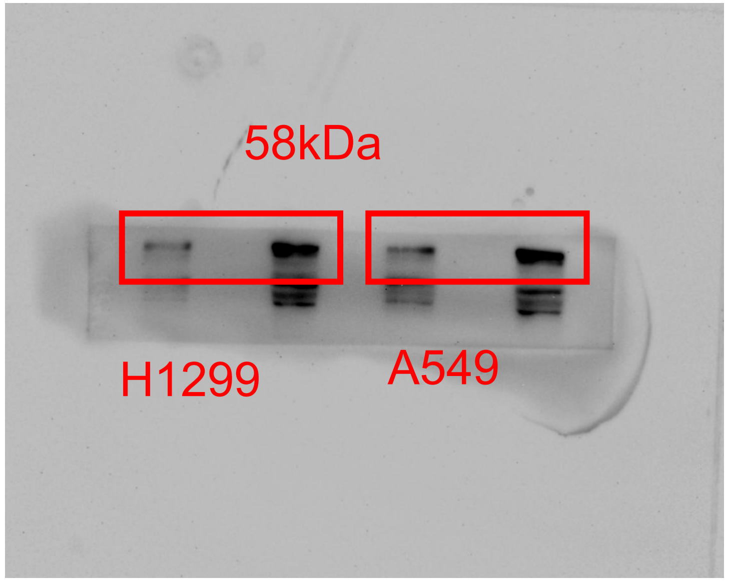


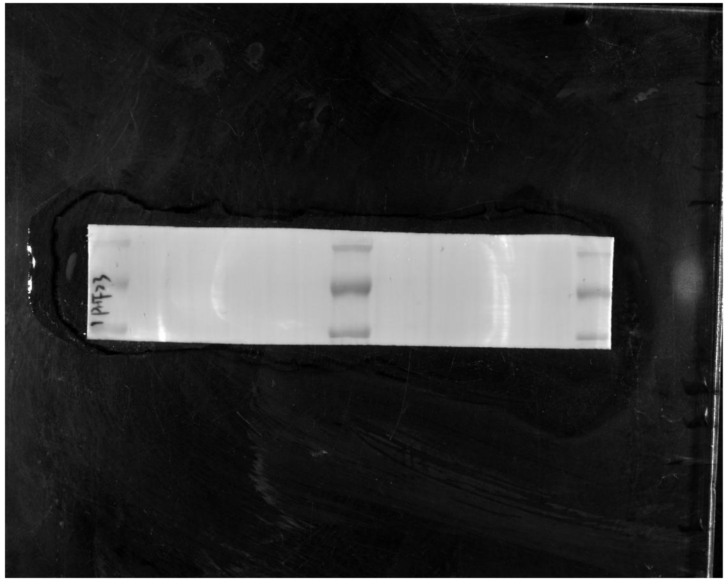

# Figure5C

H1299-IP-PHF23

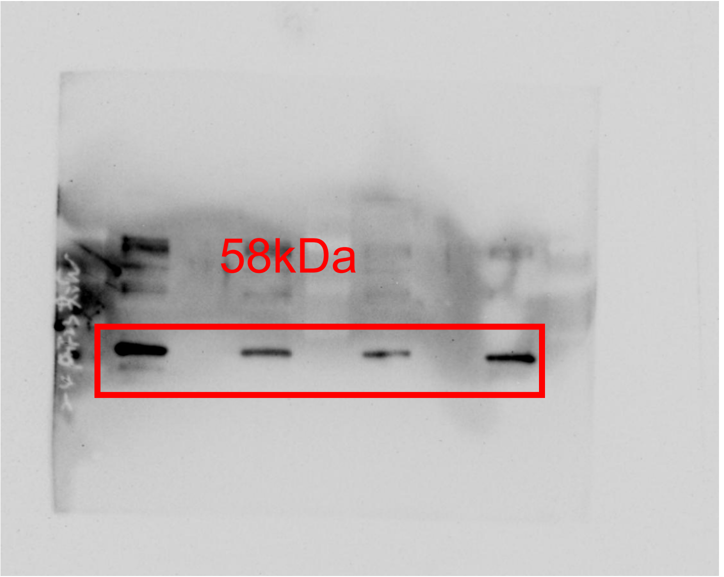


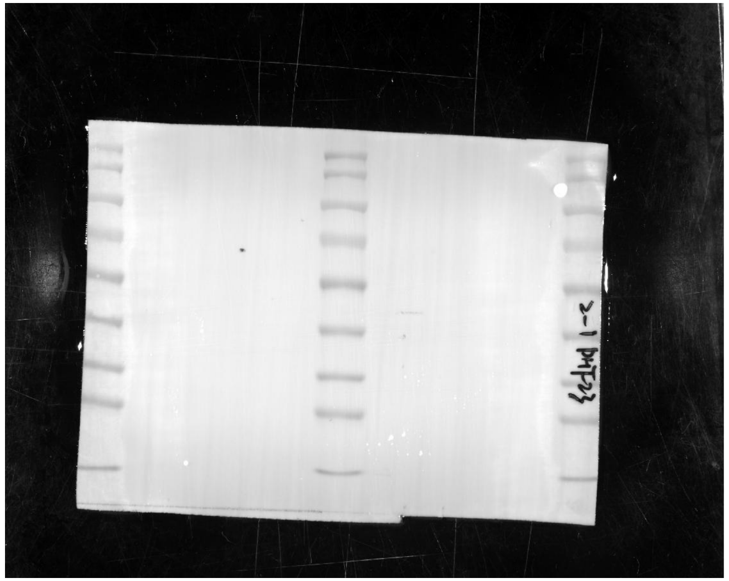

H1299-IB-ACTN4

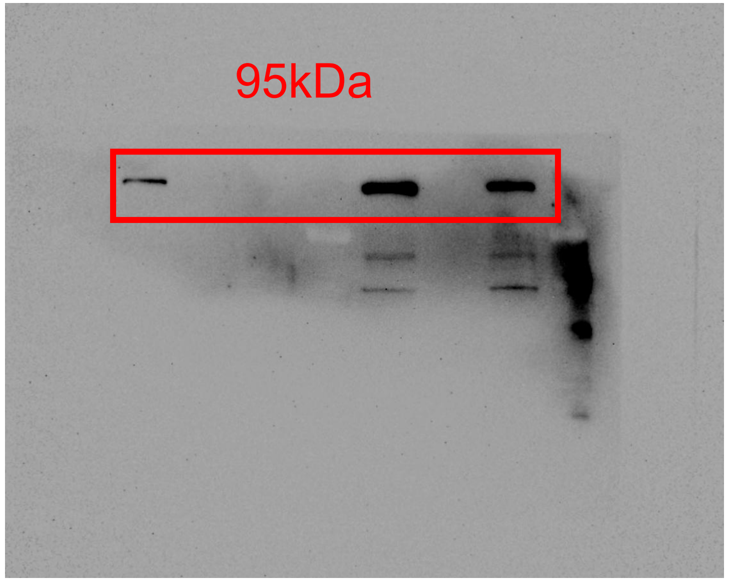


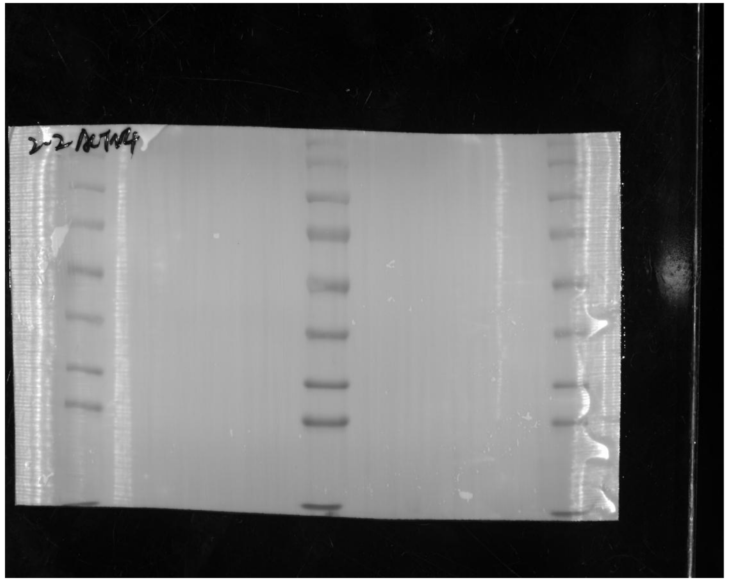

A549-IP-PHF23

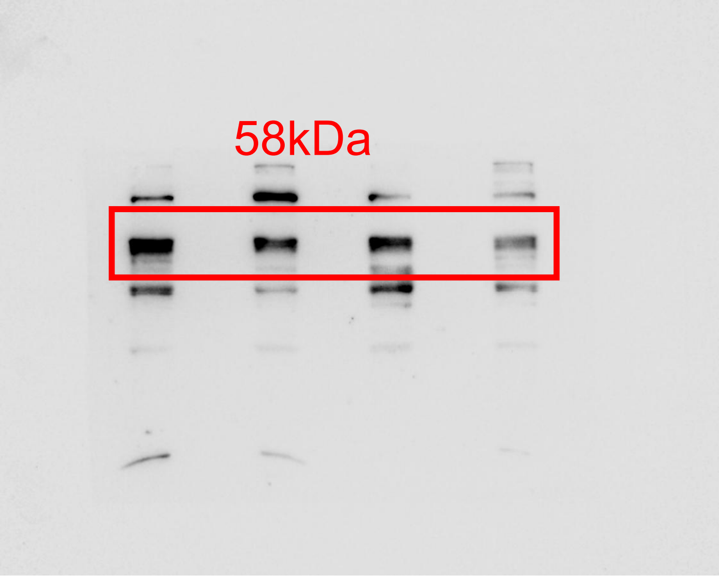


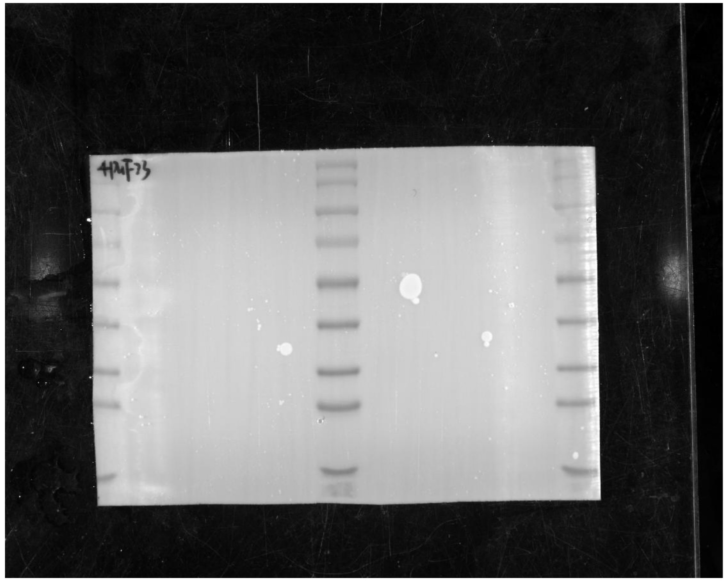

A549-IB-ACTN4

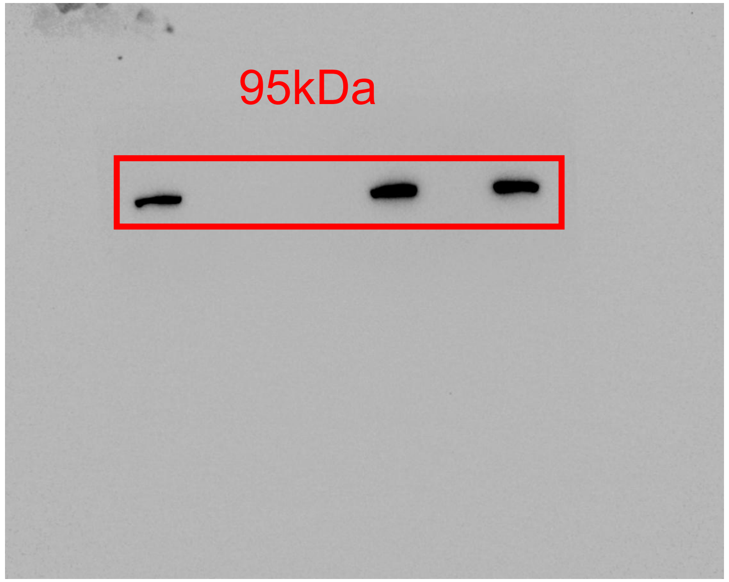


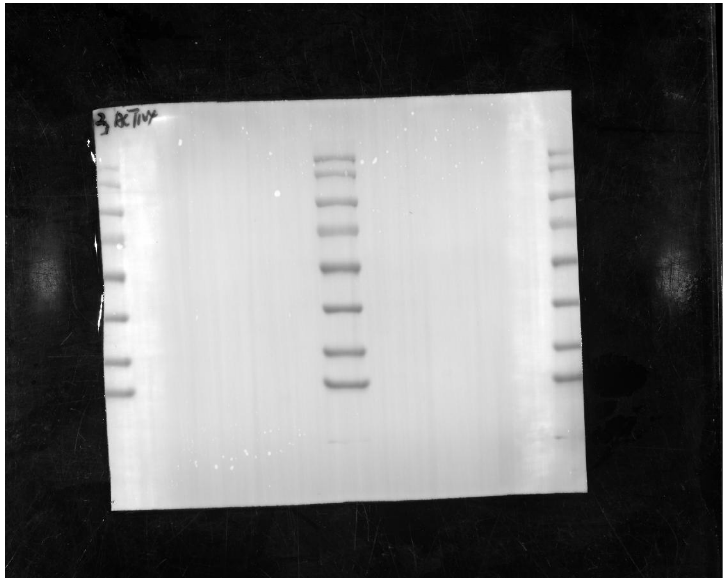

# Figure5D

H1299ACTN4

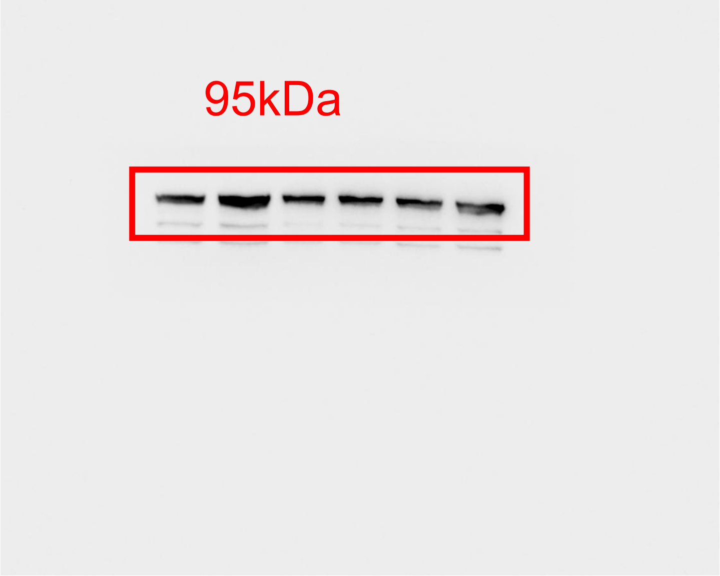


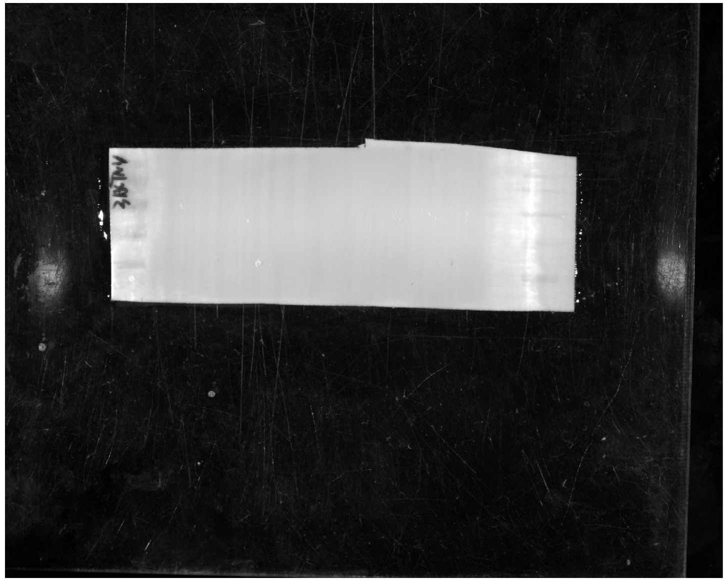

A549ACTN4

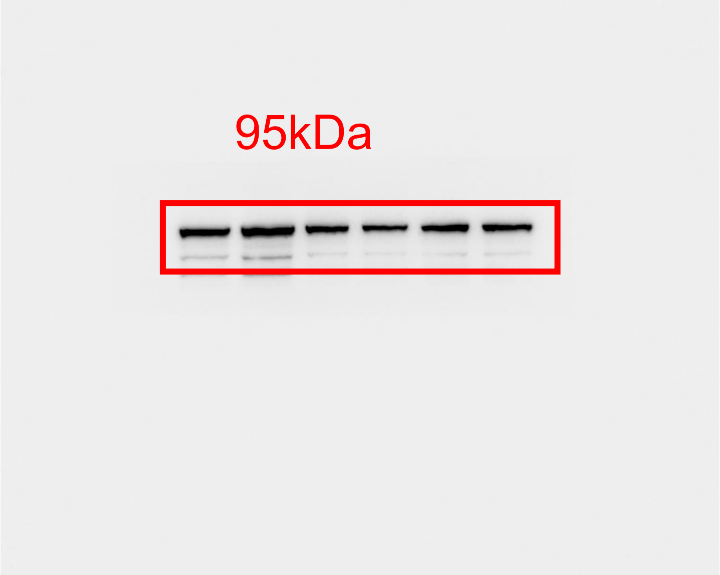


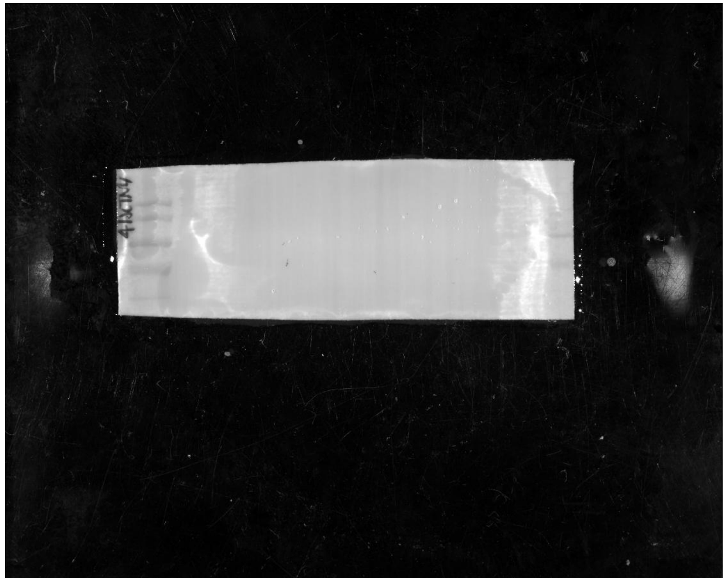

H1299MYC-PHF23

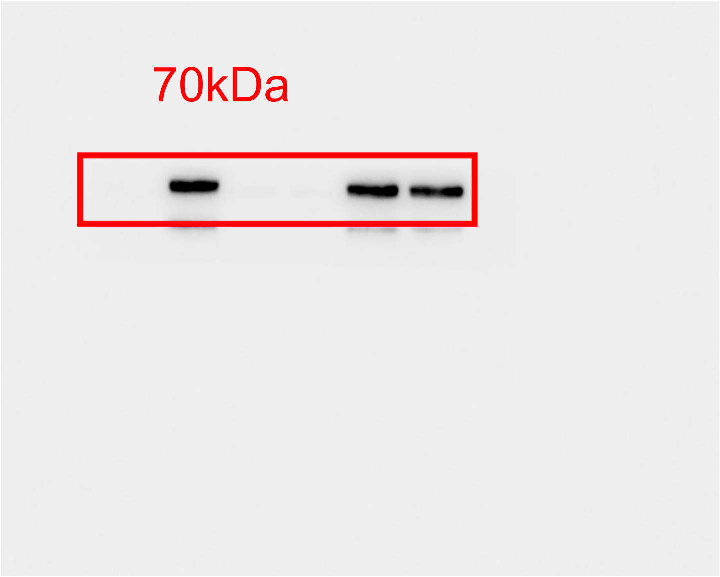


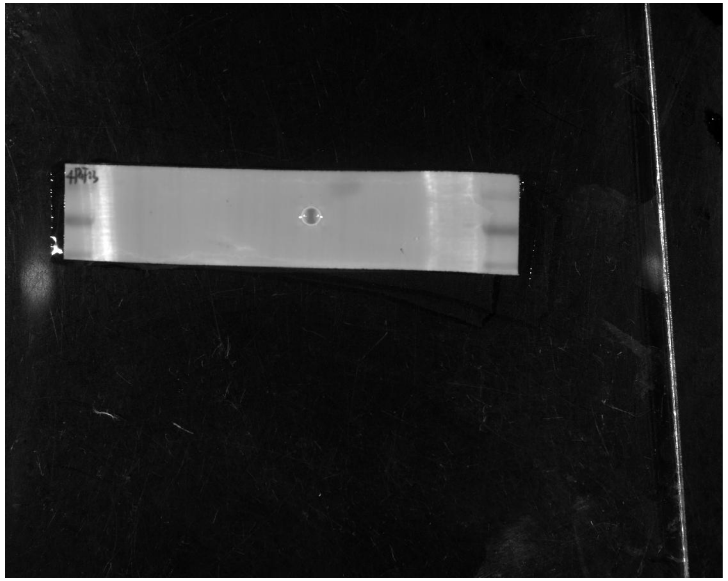

A549MYC-PHF23

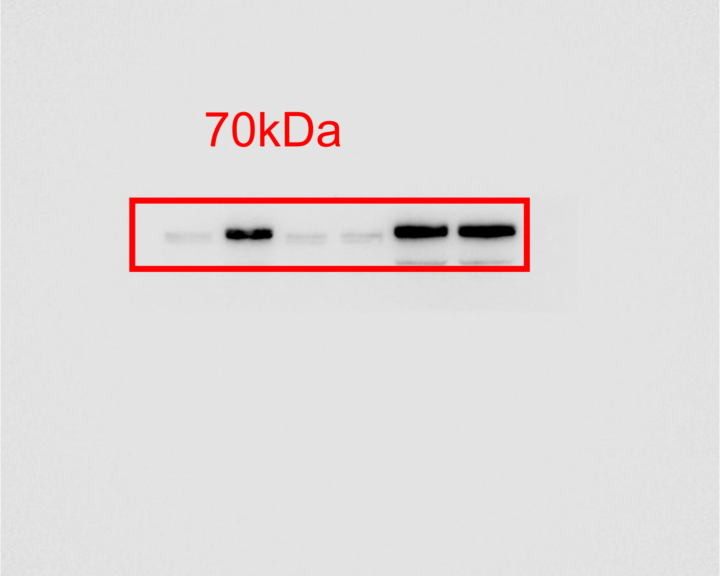


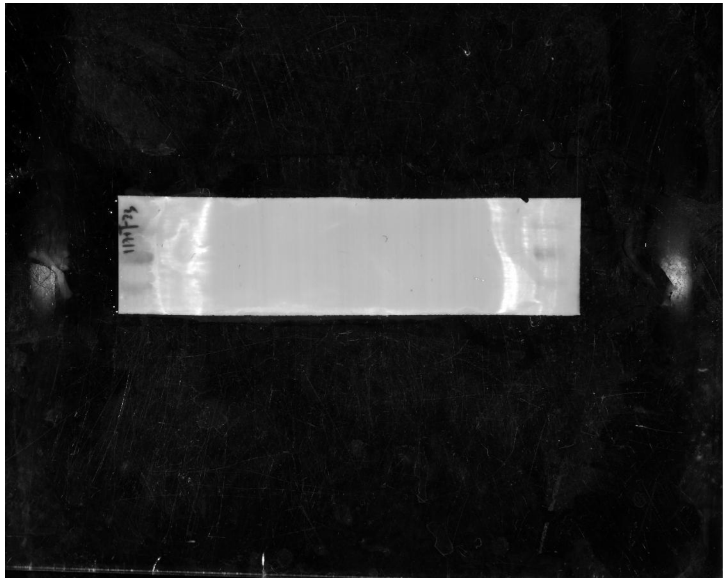

H1299p-ERK

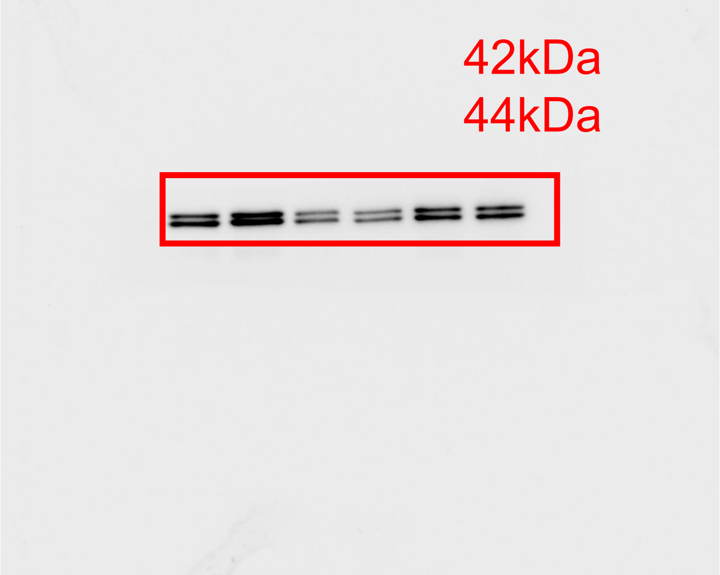


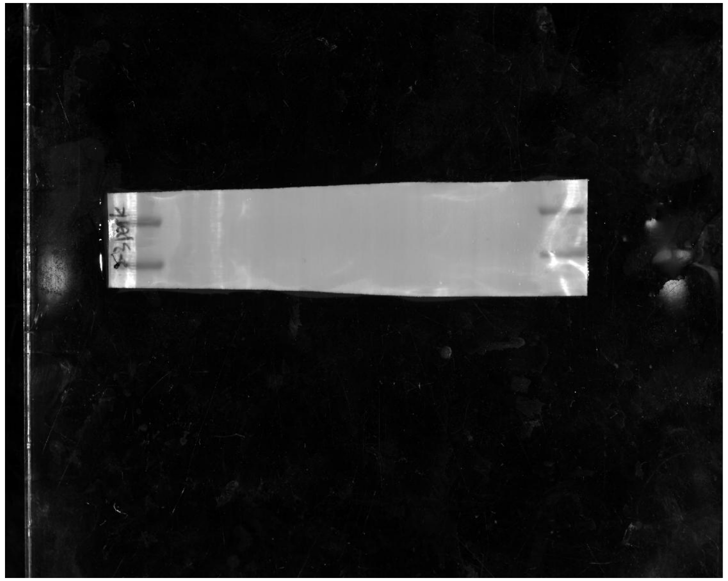

A549p-ERK

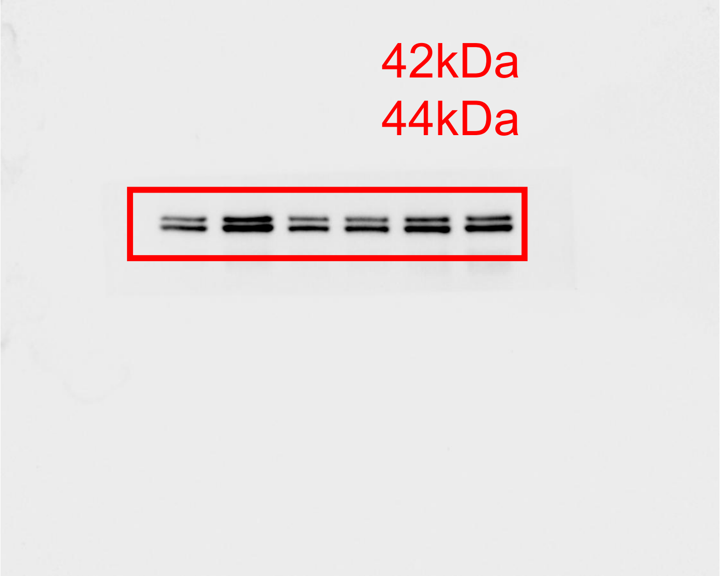


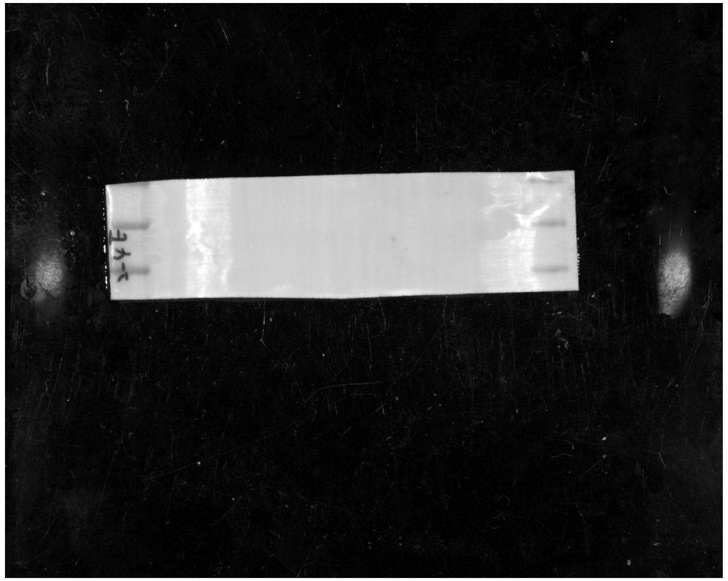

H1299ERK

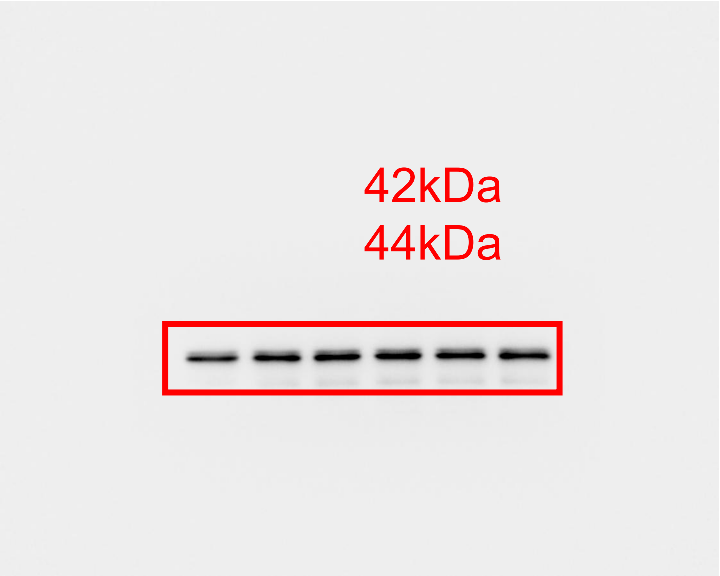


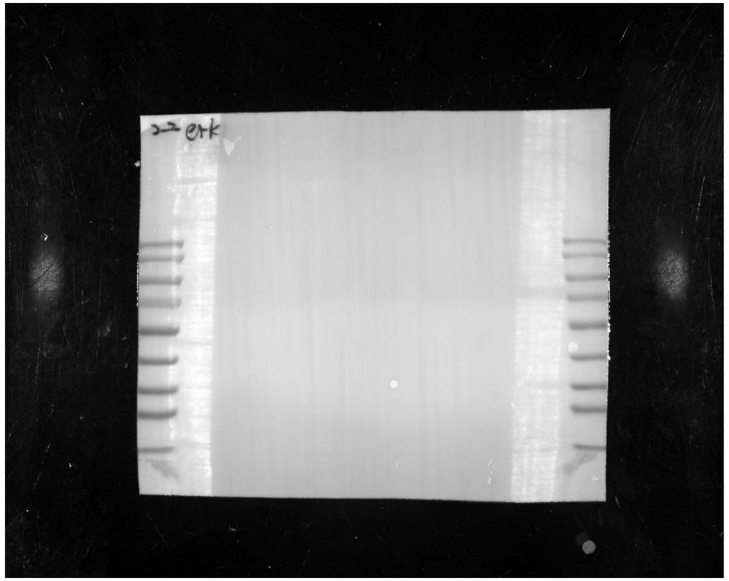

A549ERK

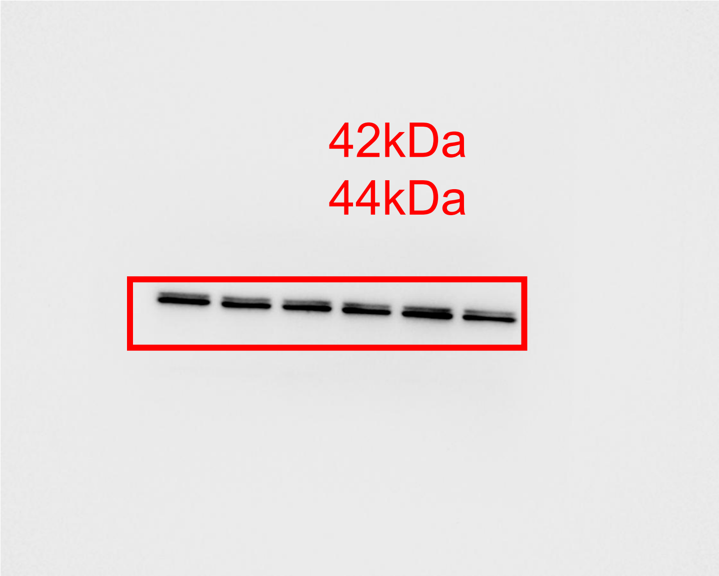


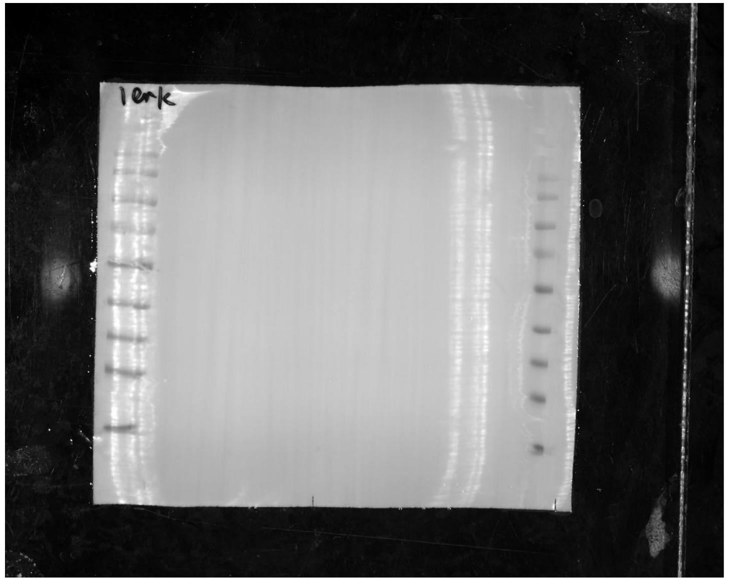

H1299p-Jun (Ser73)

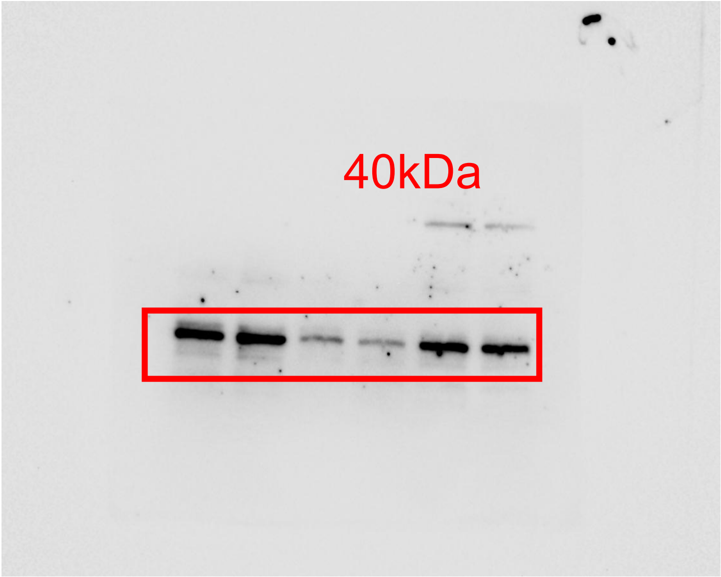


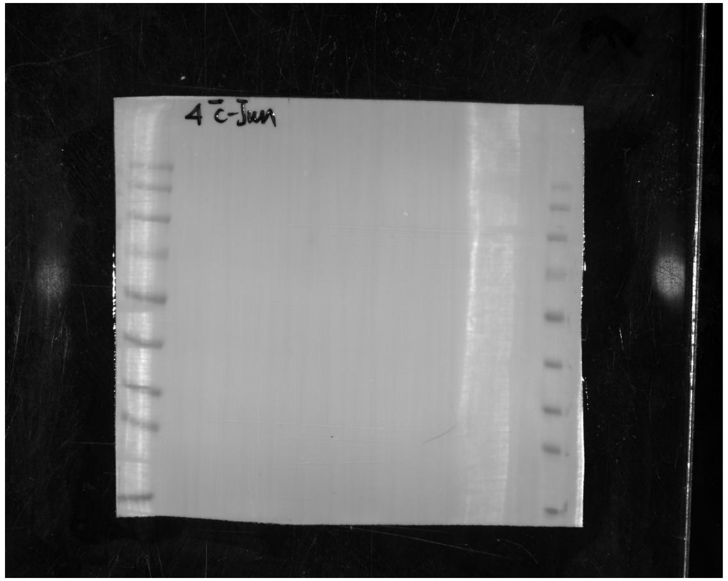


A549p-Jun (Ser73)

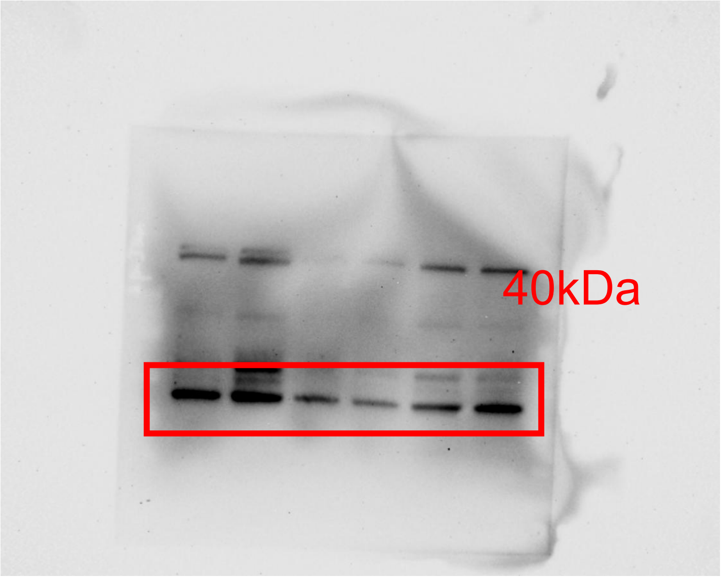


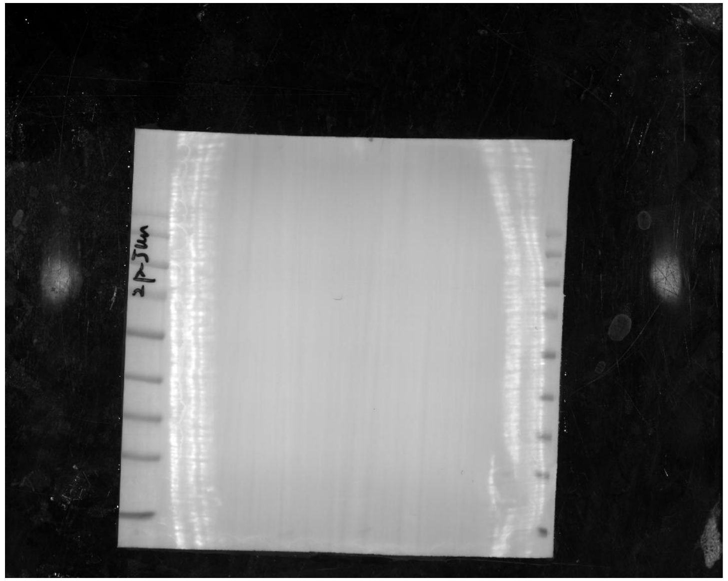

H1299p38

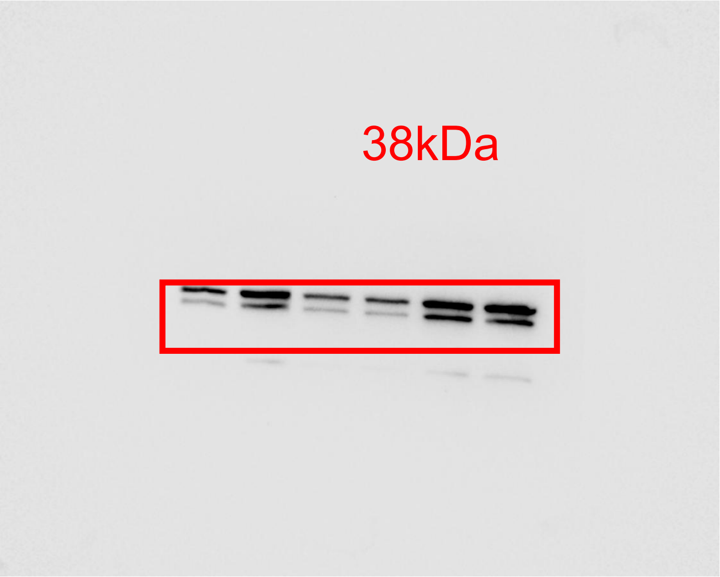


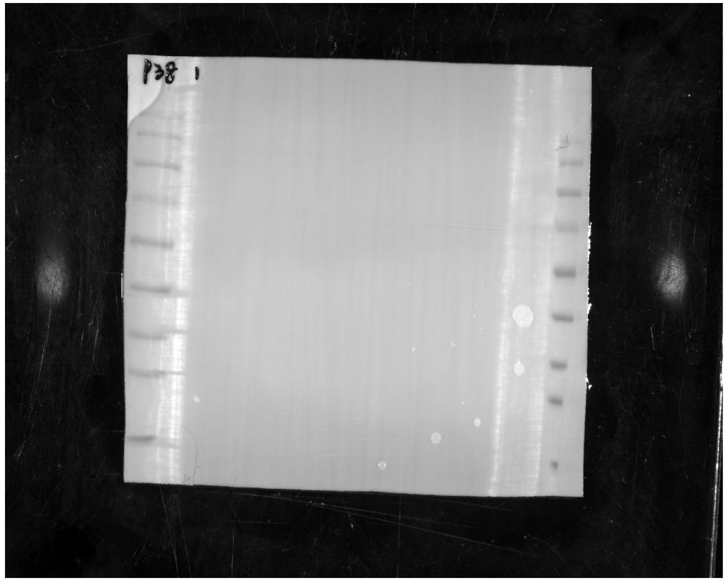

A549p38

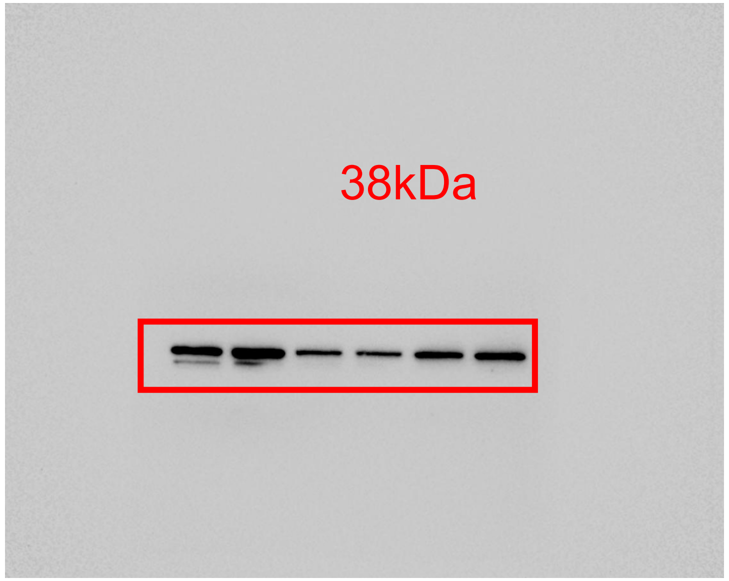


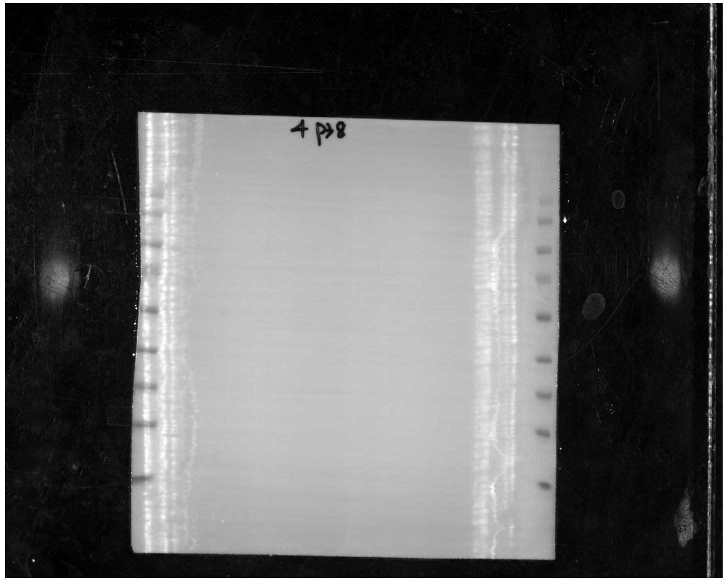

H1299c-myc

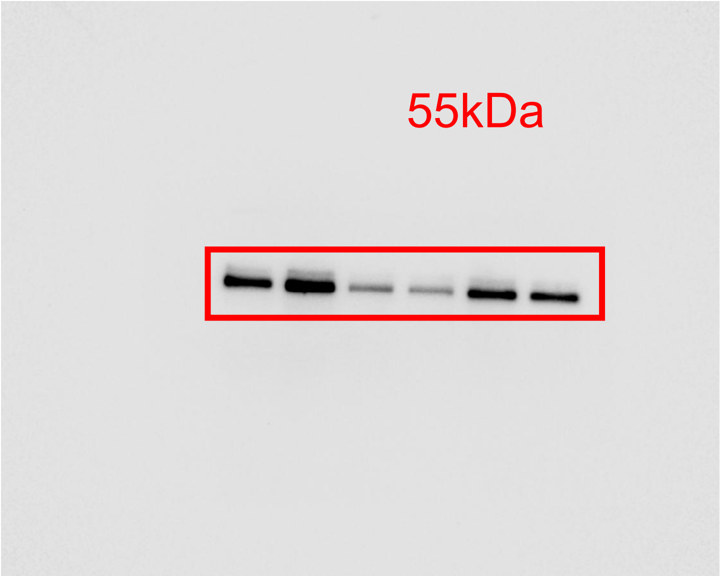


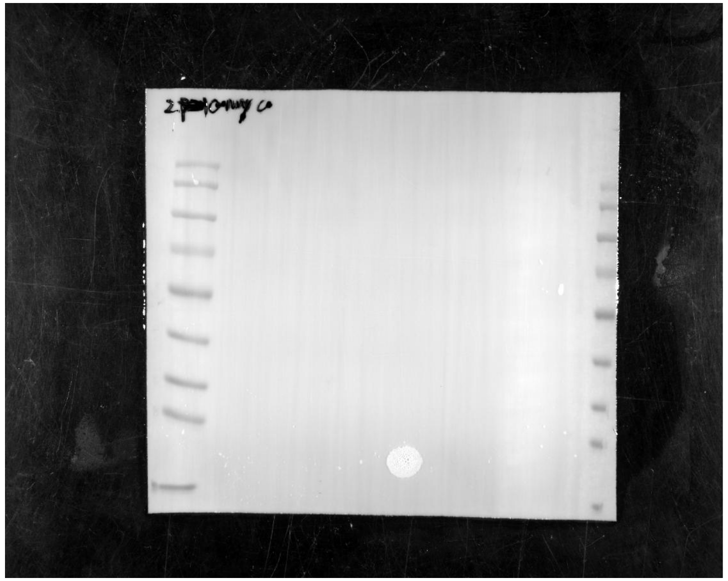

A549c-myc

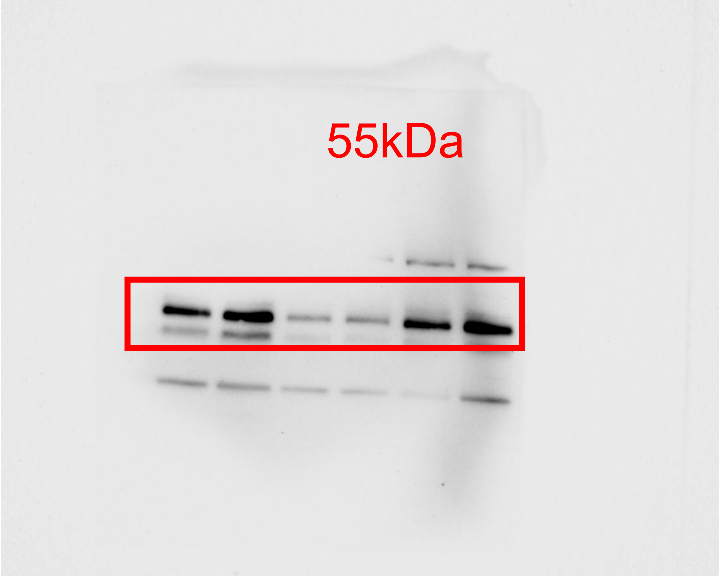


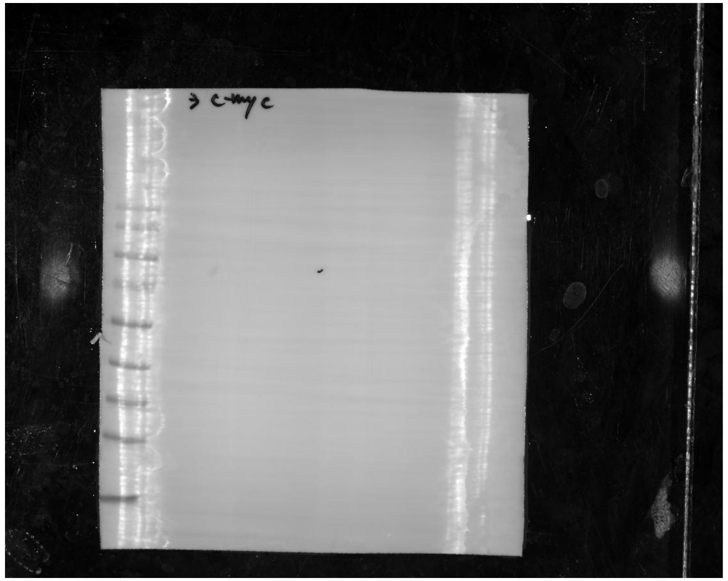

H1299GAPDH

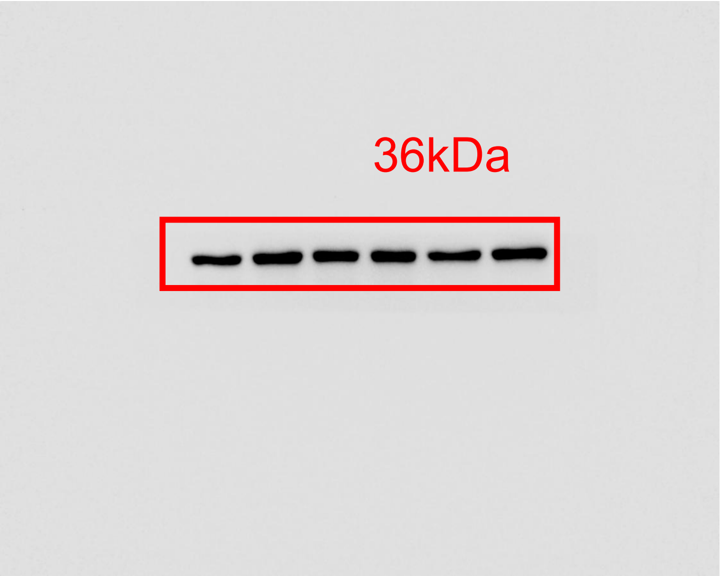


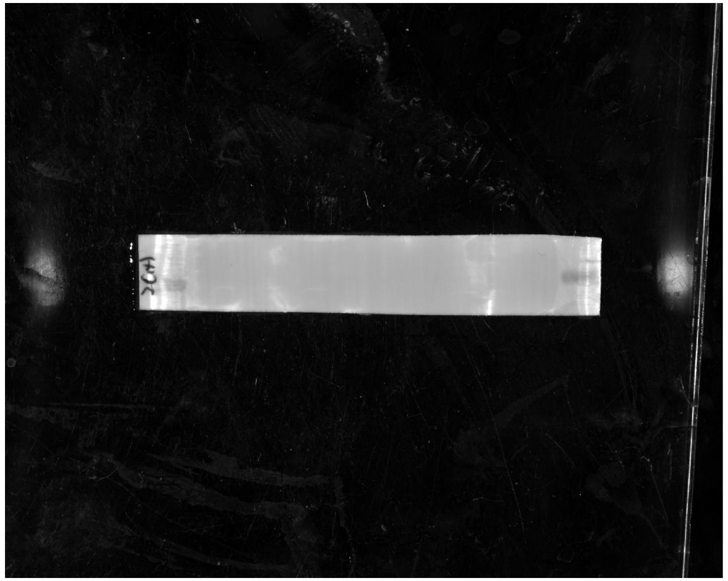

A549GAPDH

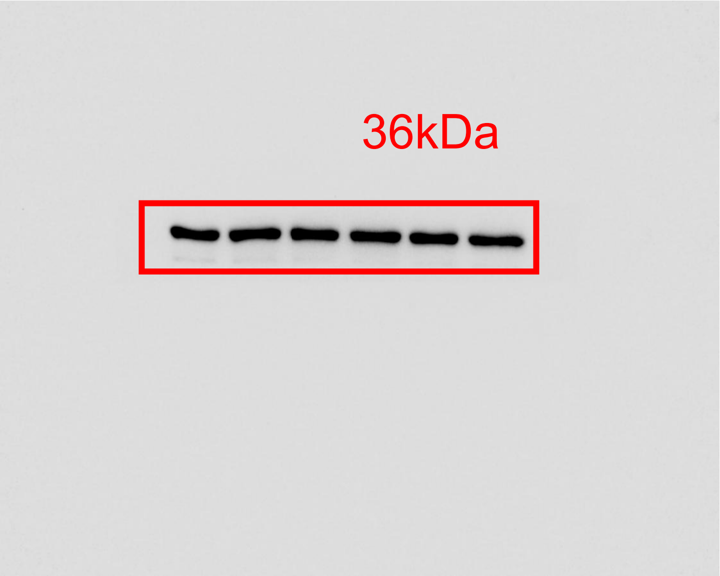


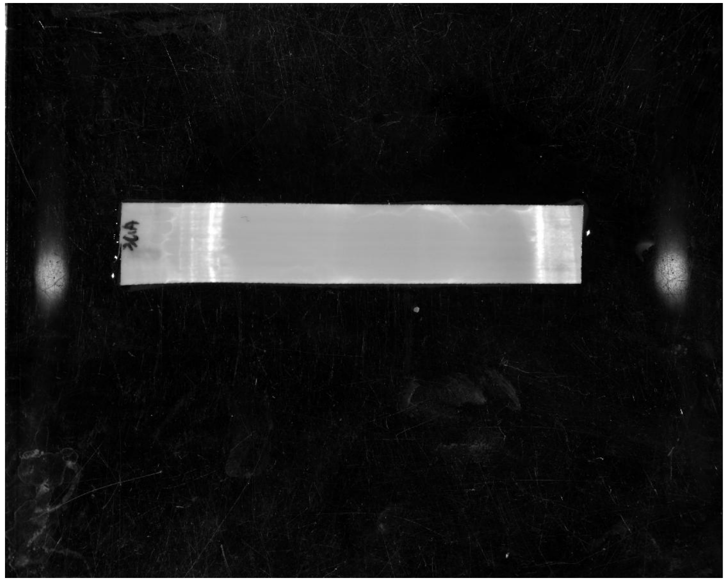

# S-Figure5C

H1299ACTN4

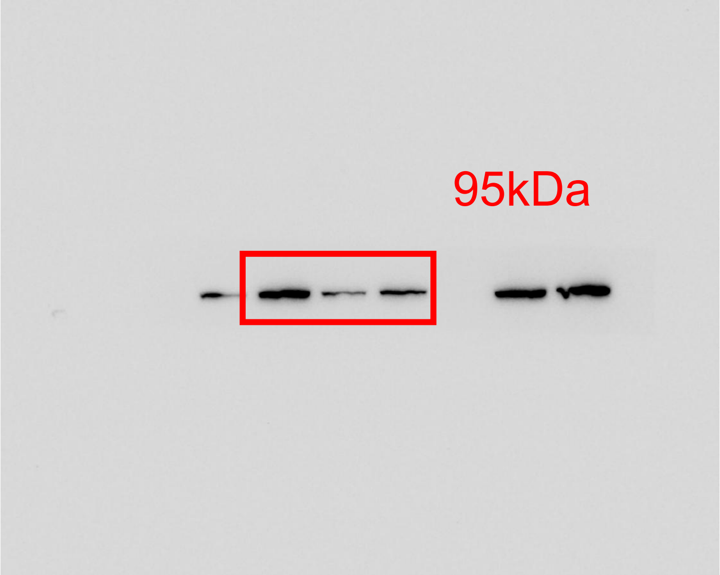


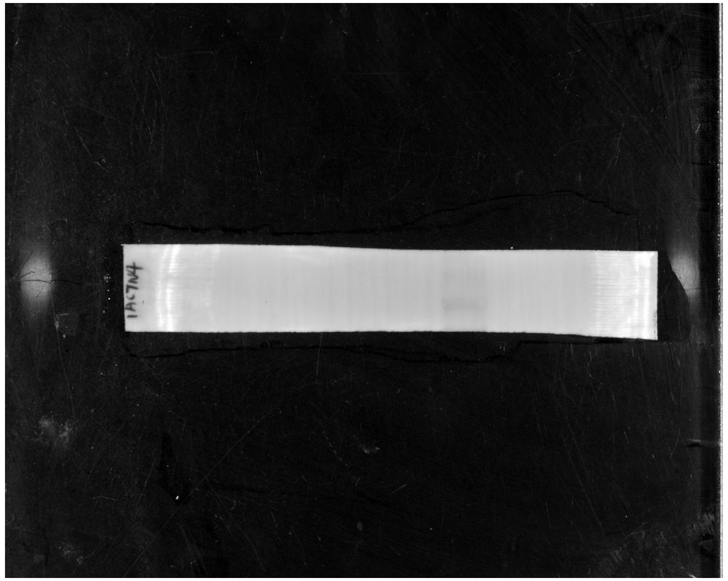

H1299p-ERK

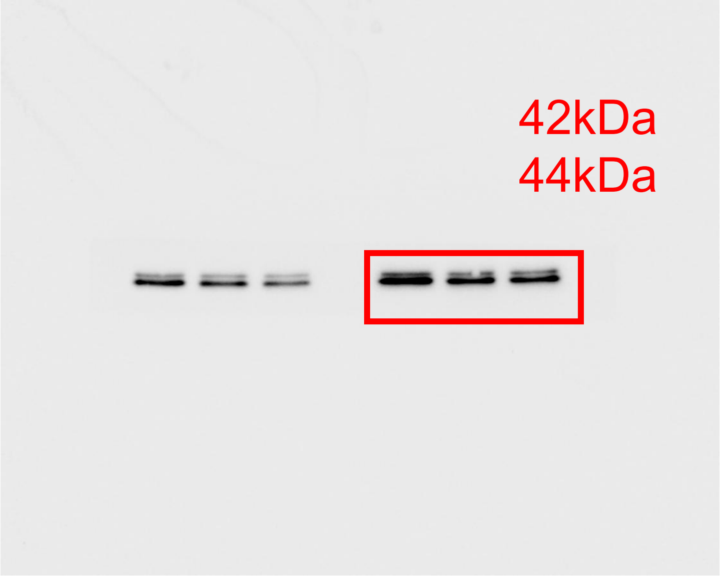


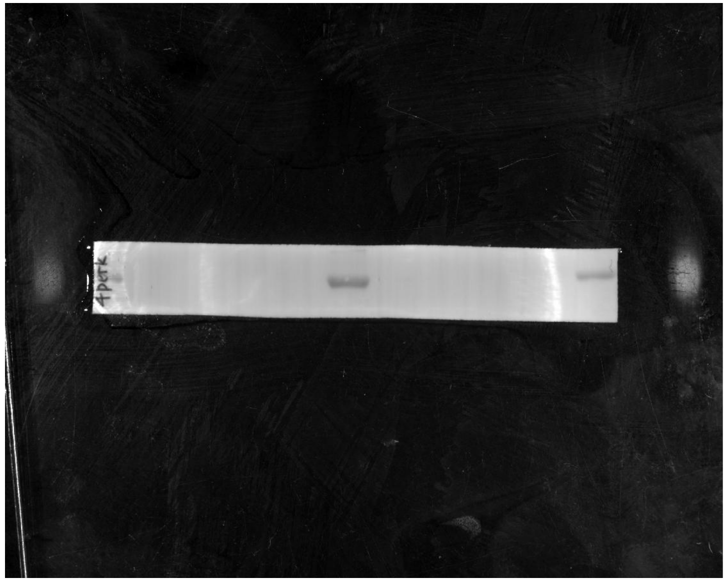

H1299PHF23

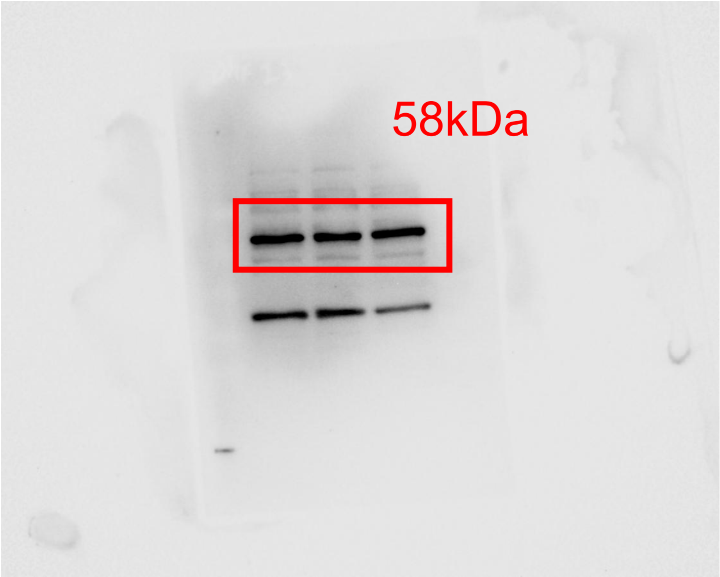


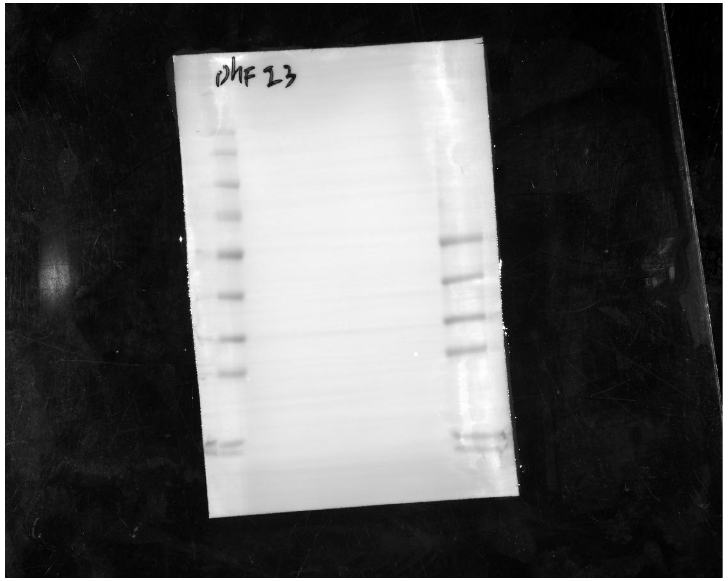

H1299
ERK

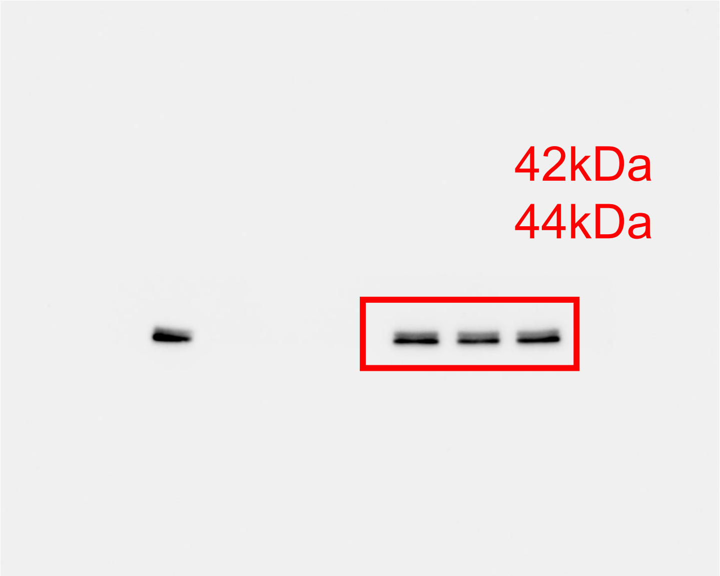


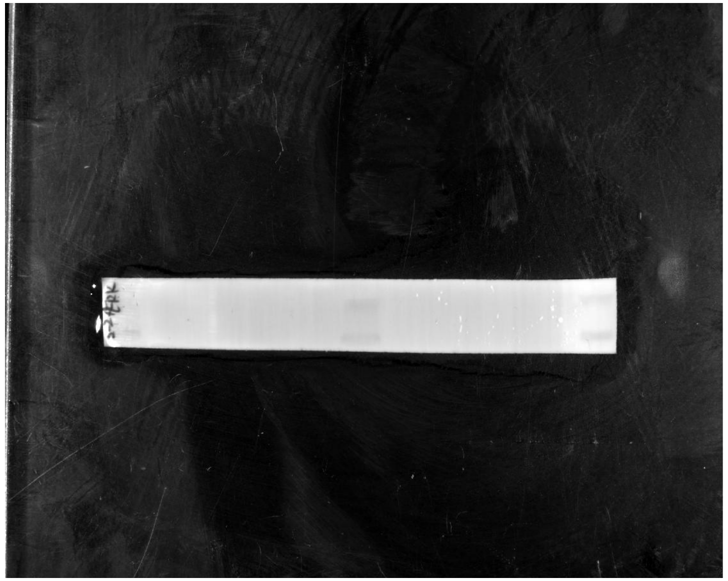

GAPDH

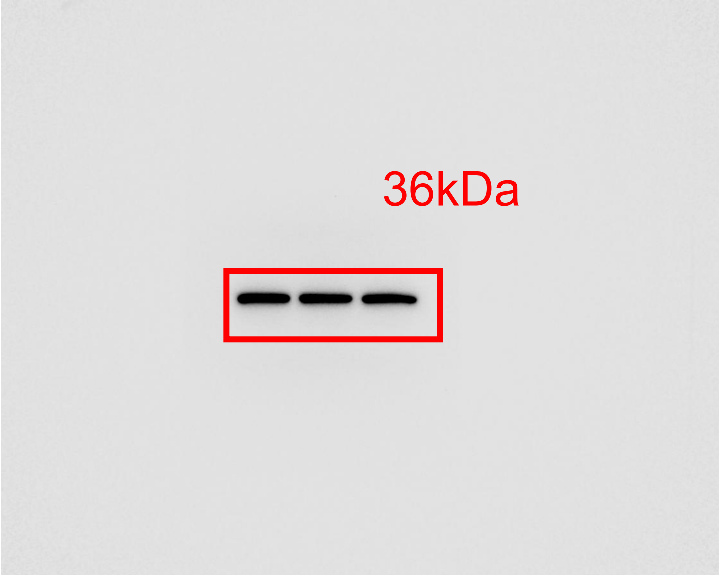


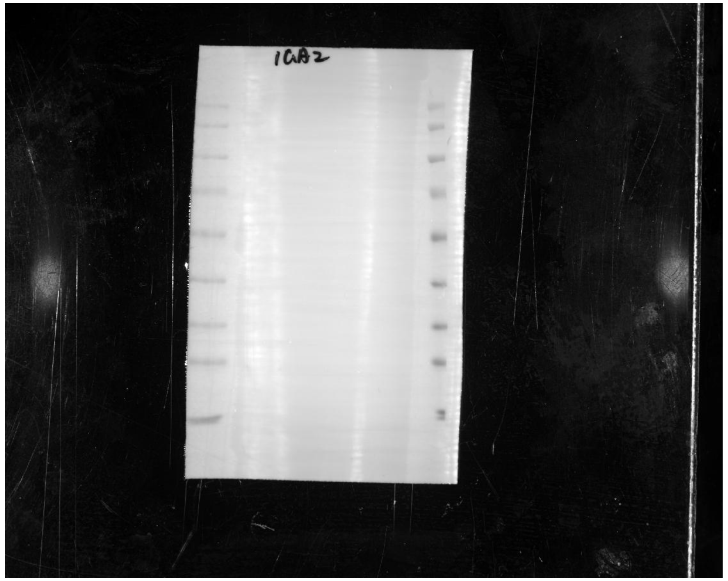

A549ACTN4

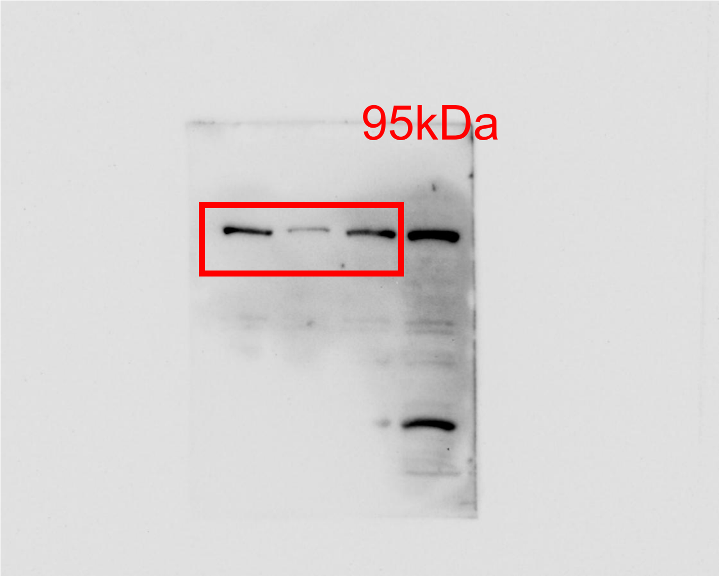


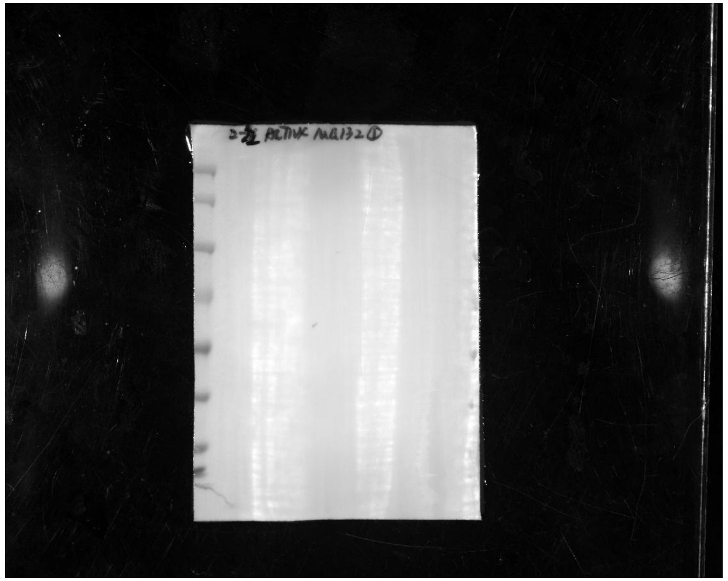

A549p-ERK

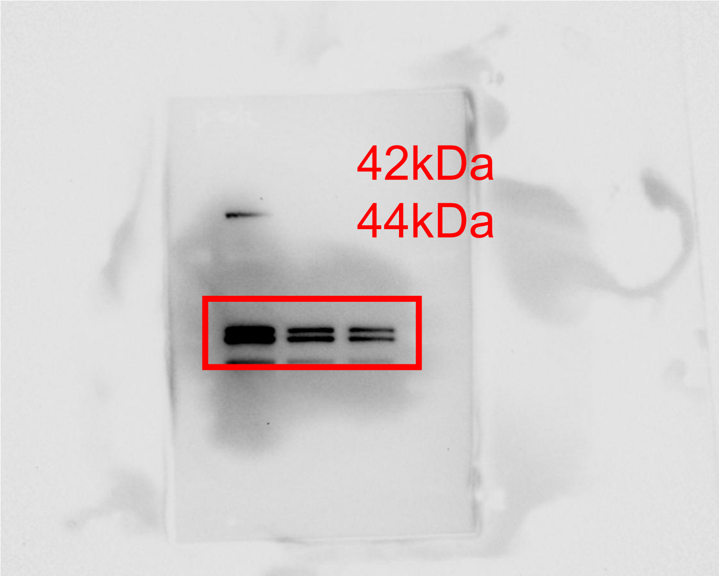


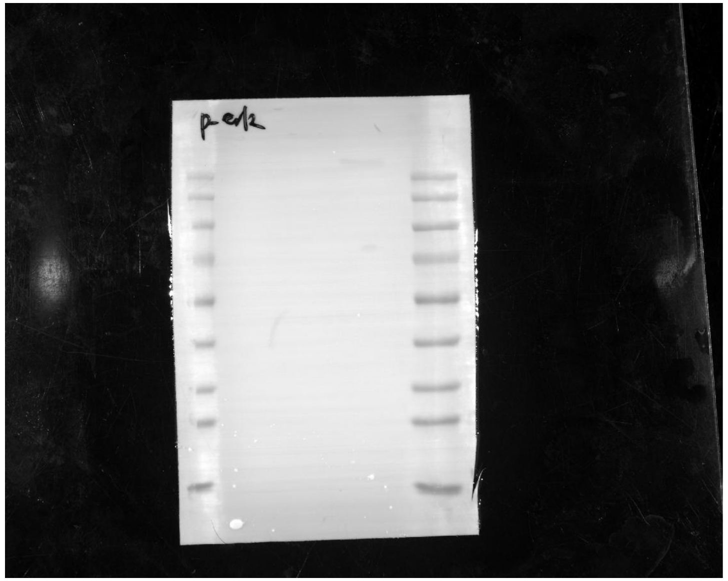

A549PHF23

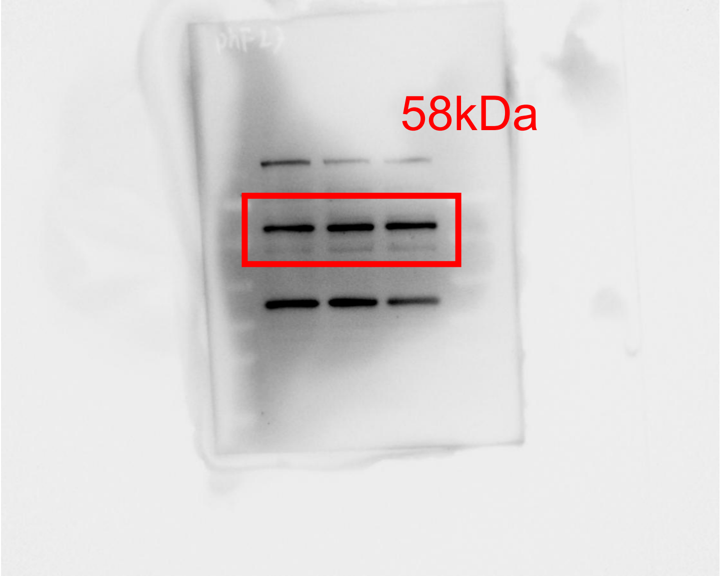


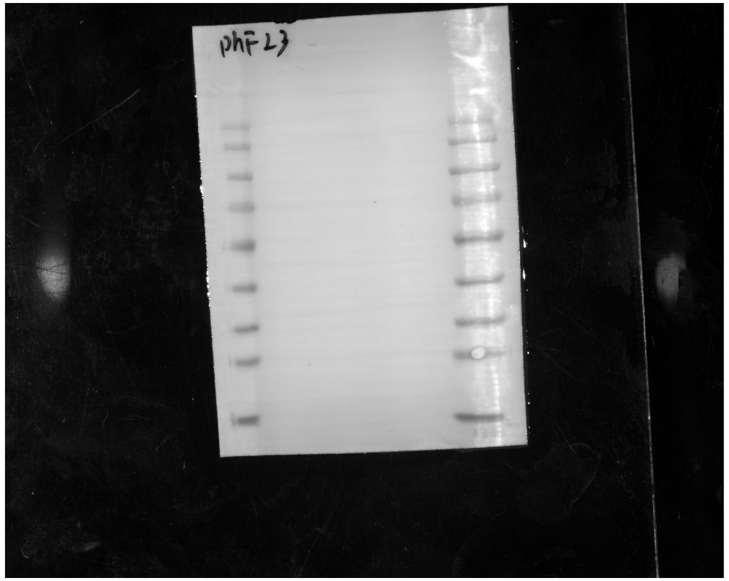


A549ERK

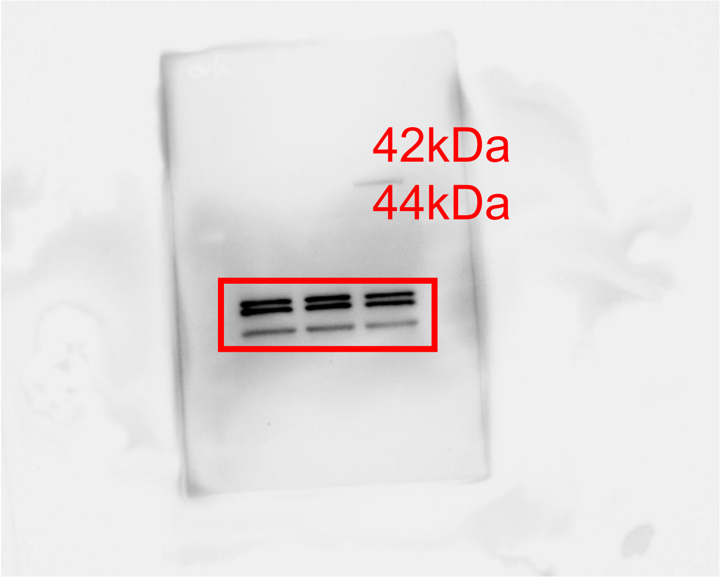


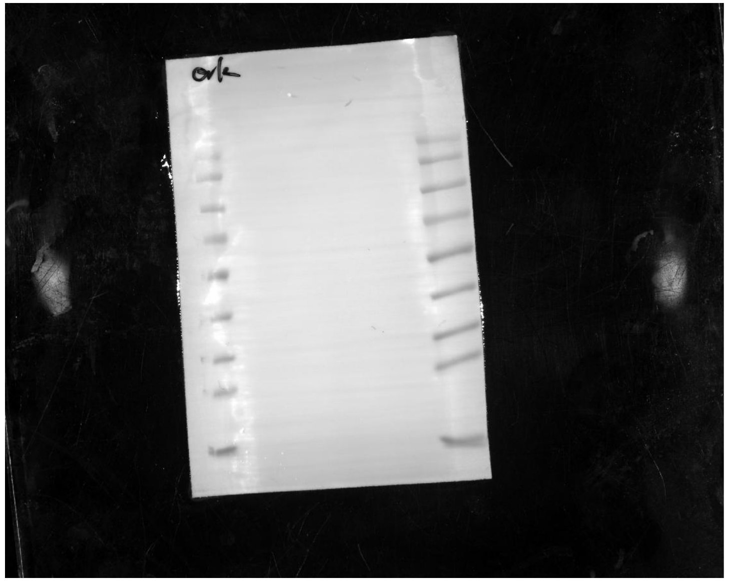

A549GAPDH

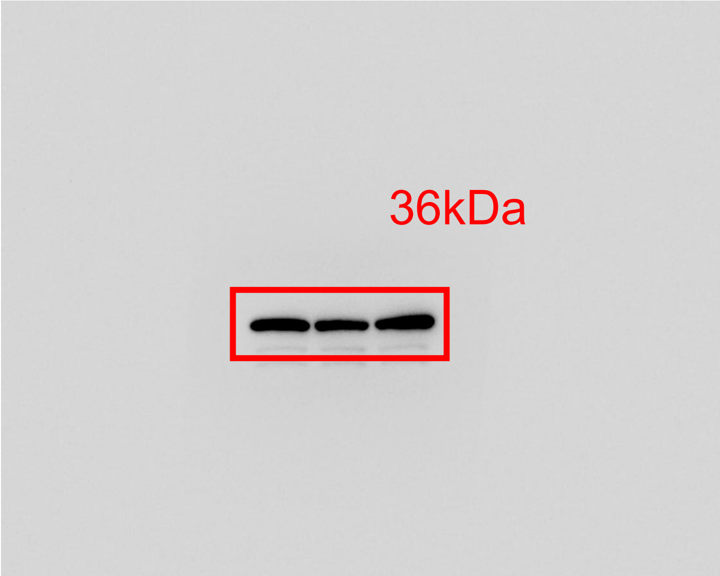


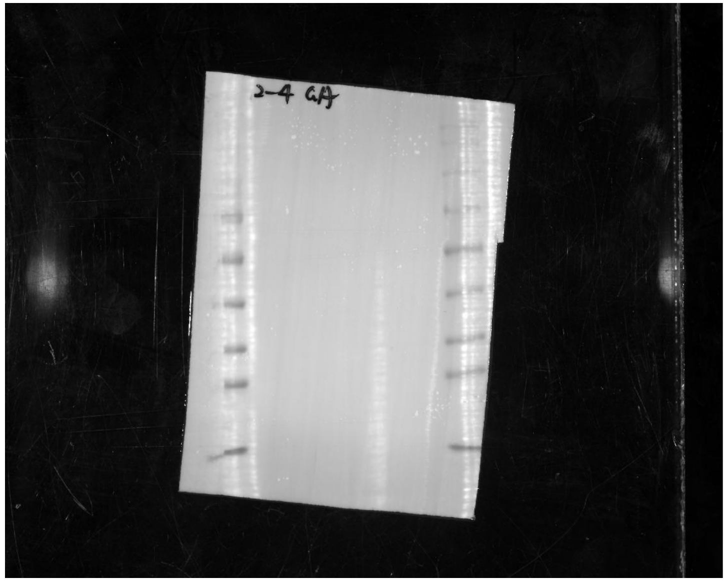

# S-Figure5D

H1299CDK4

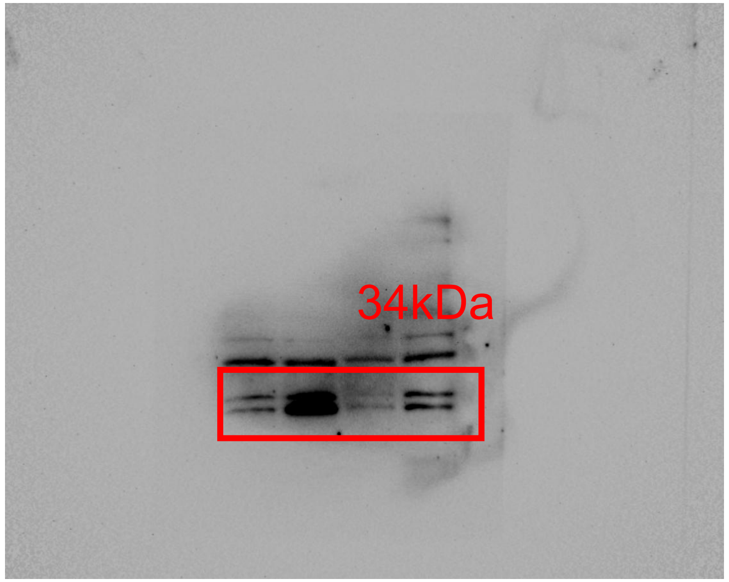


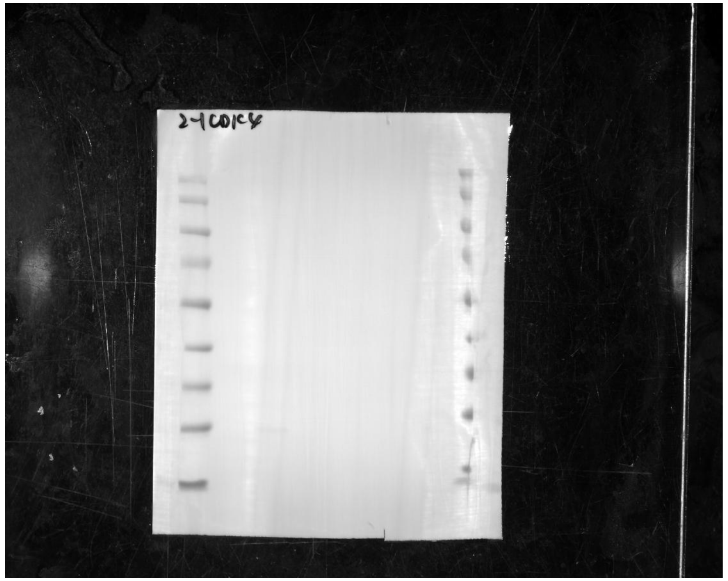

A549CDK4

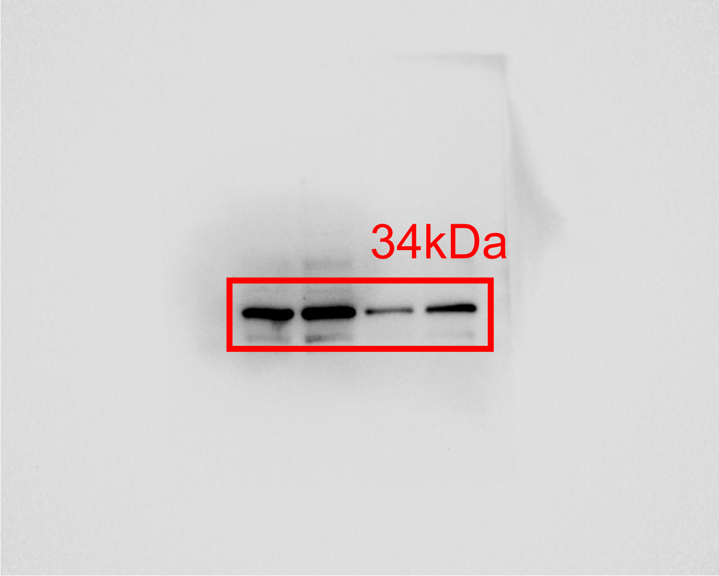


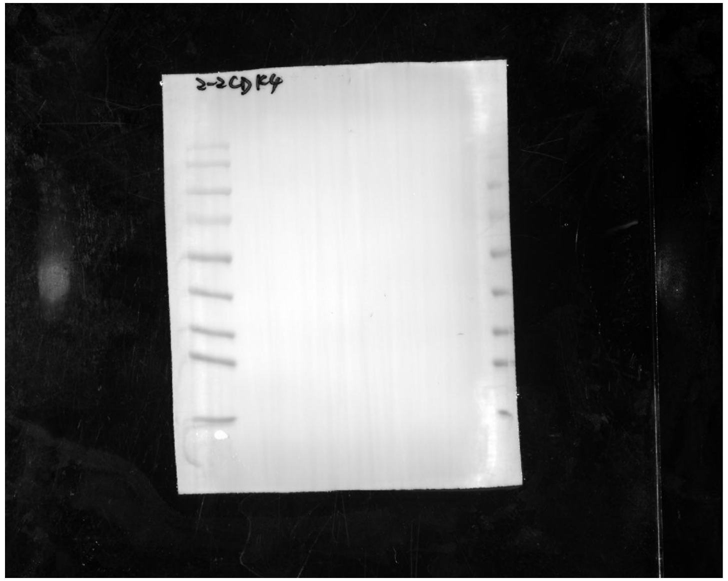

**H1299CDK6**

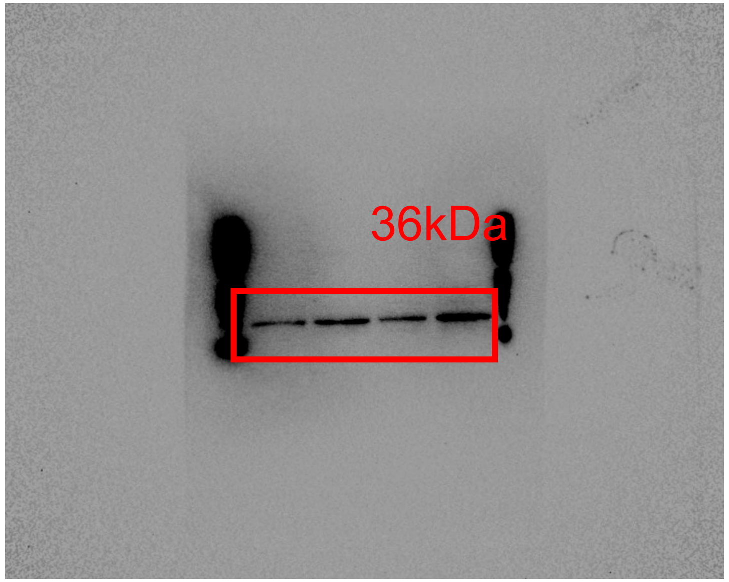


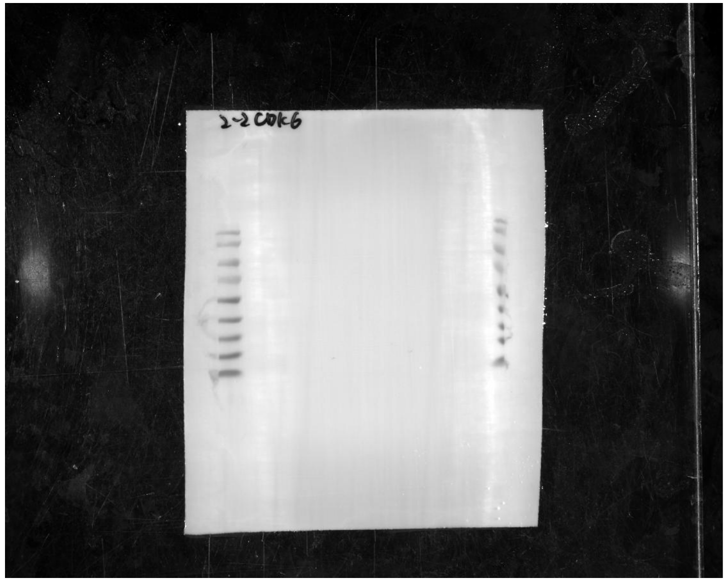

**A549CDK6**

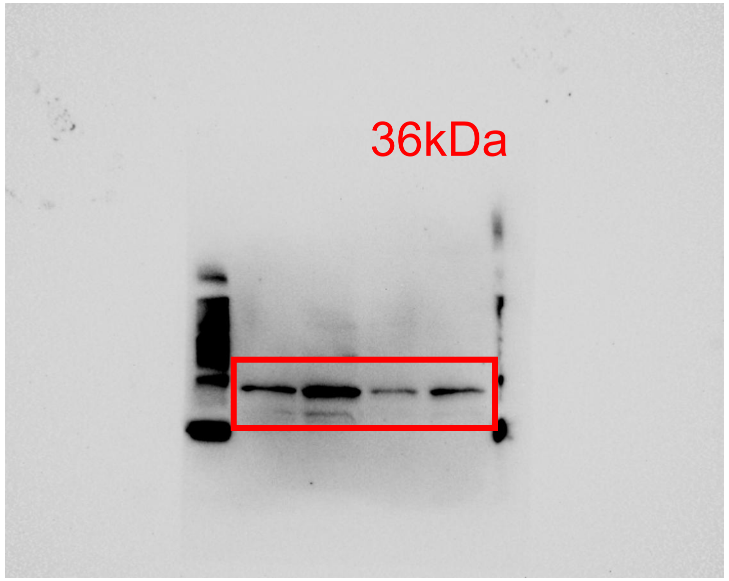


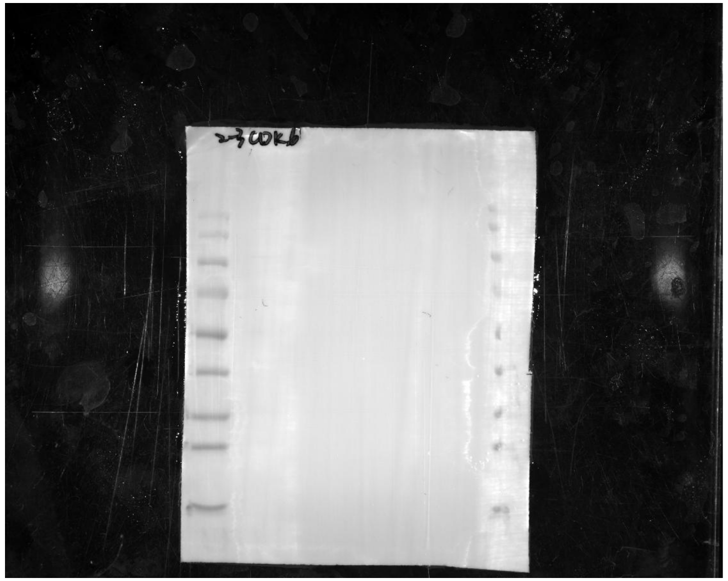

**H1299cyclinA2**

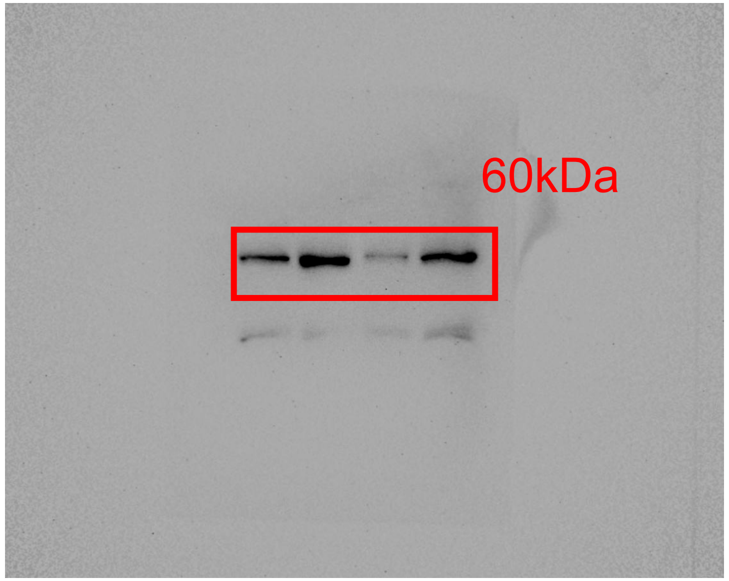


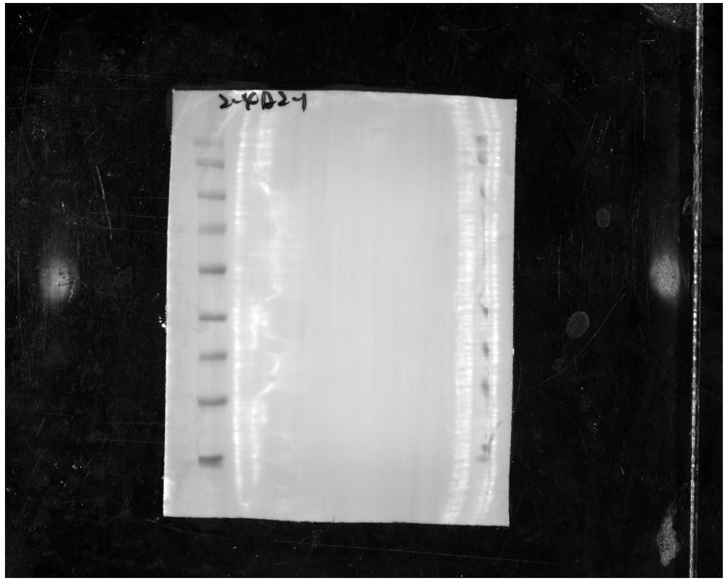

**A549cyclinA2**

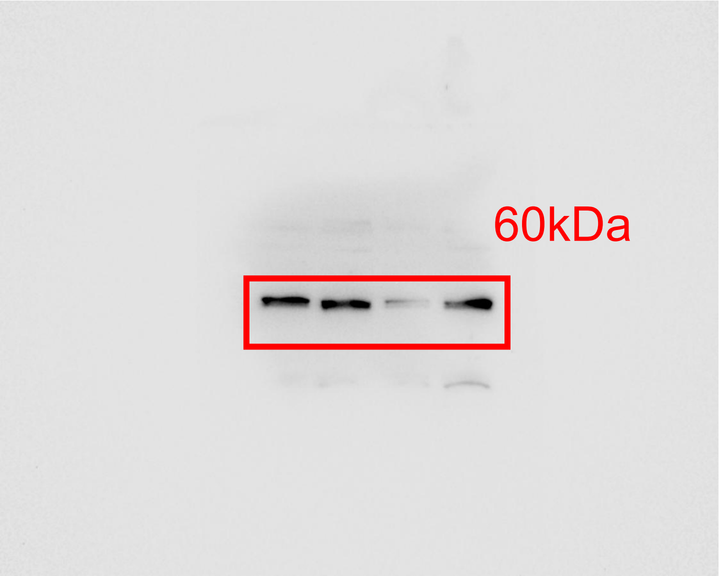


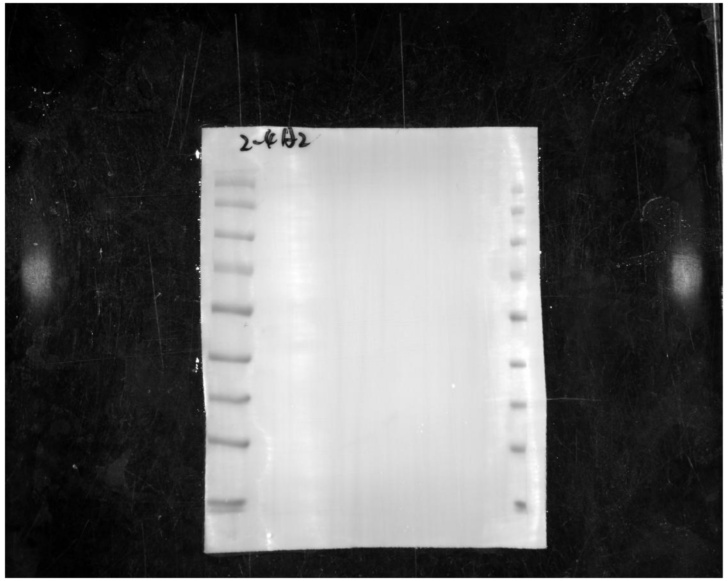

H1299cyclinD1

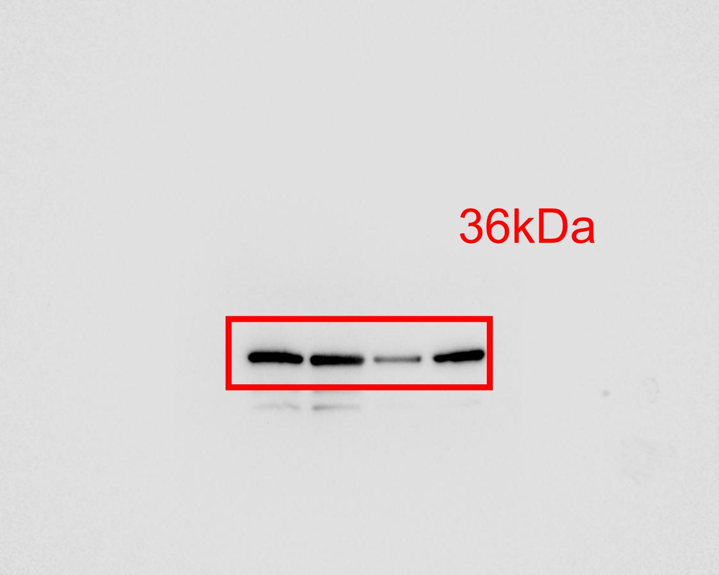


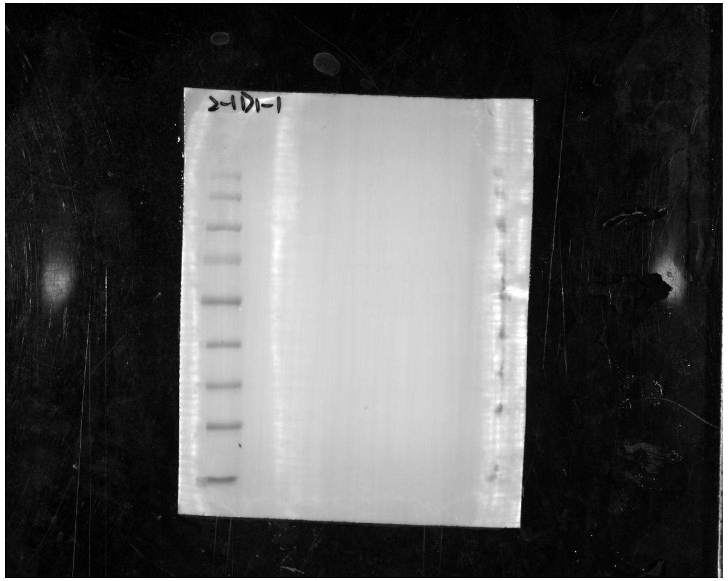

A549cyclinD1

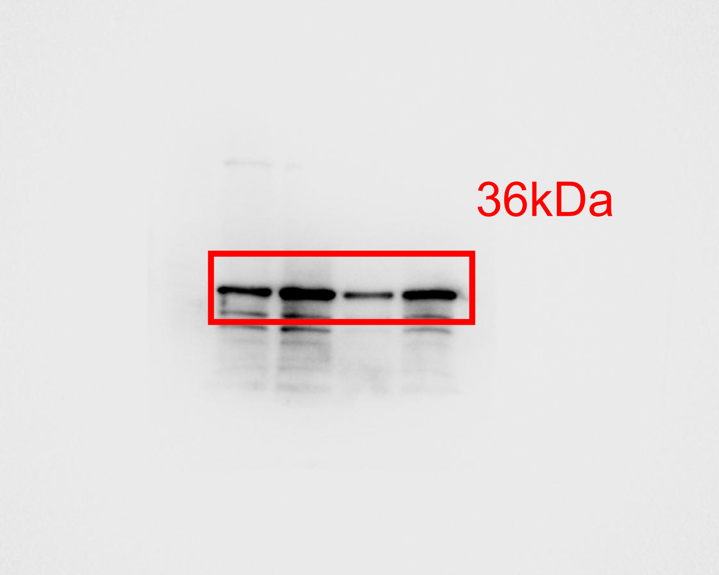


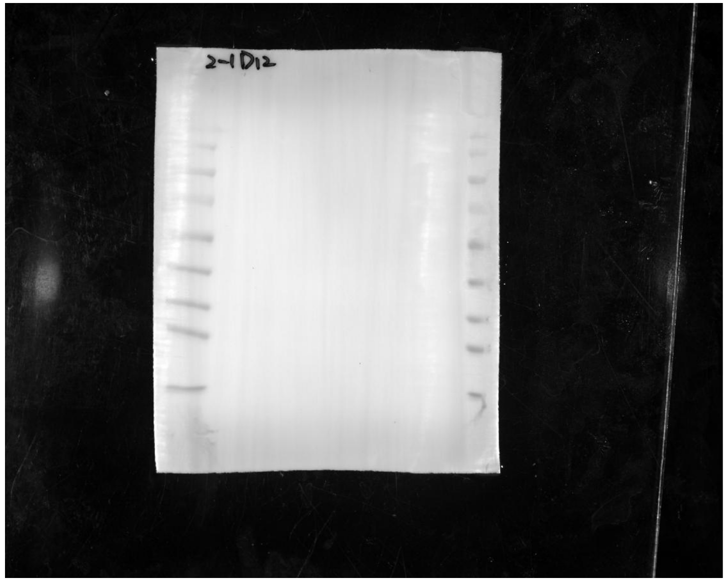

**H1299E-Cadherin**

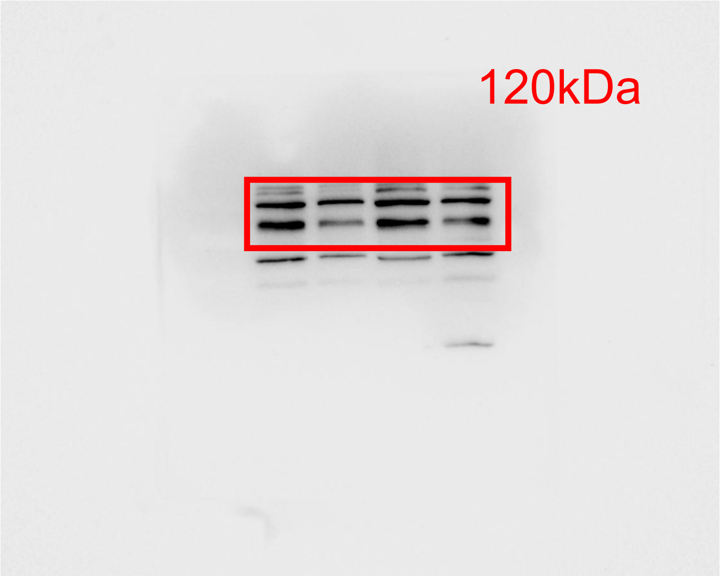


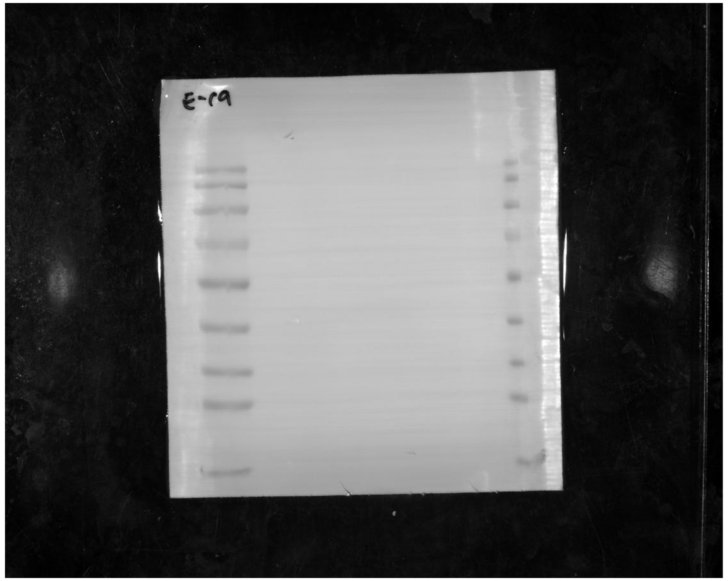

**A549E-Cadherin**

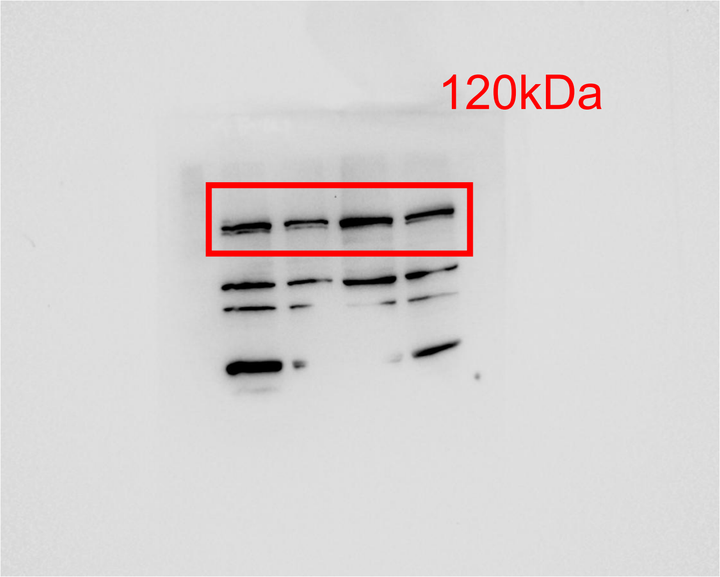


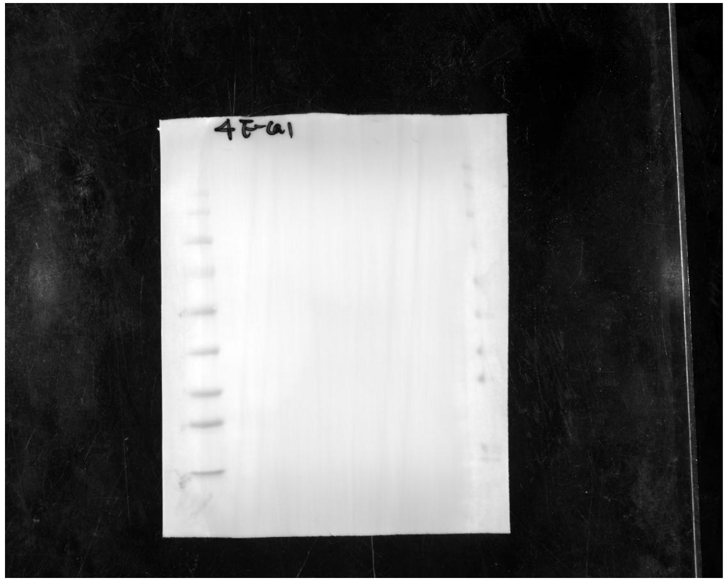

**H1299N-Cadherin**

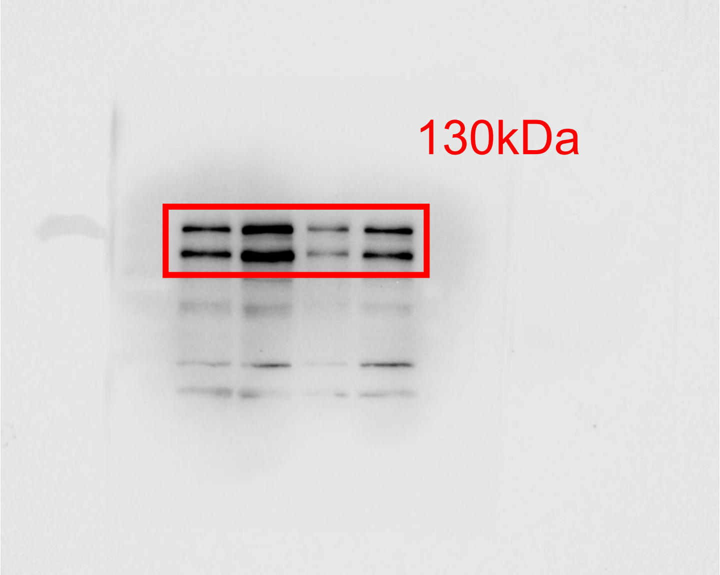


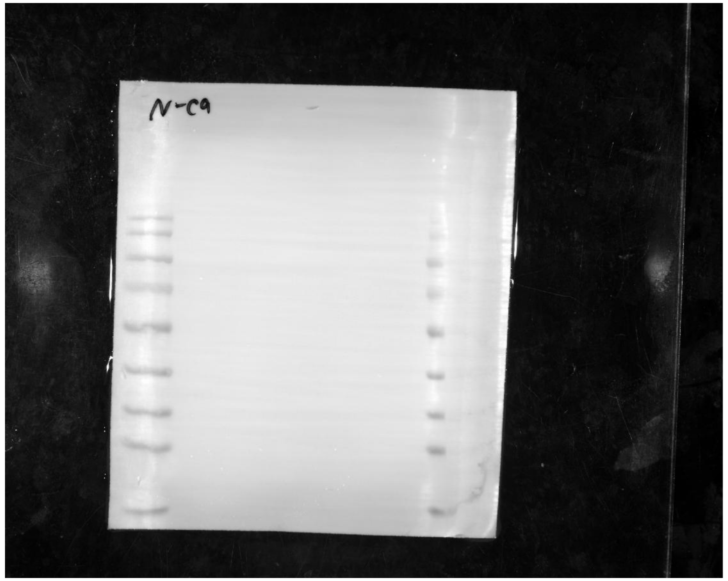


**A549N-Cadherin**

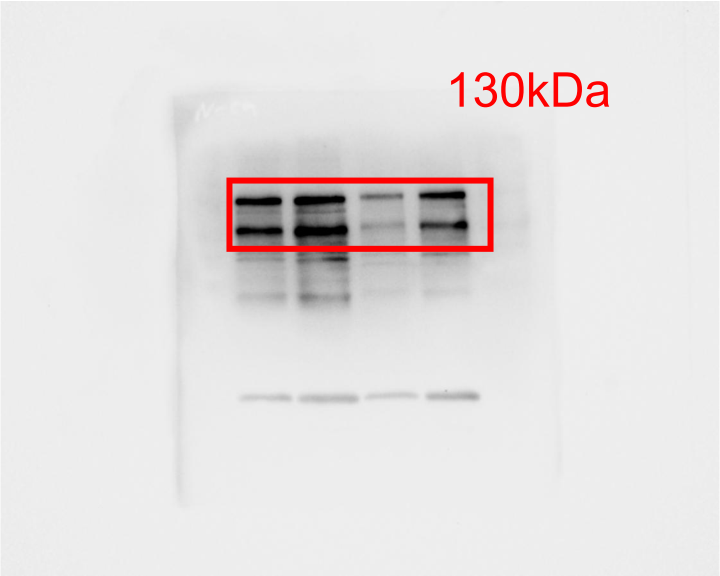


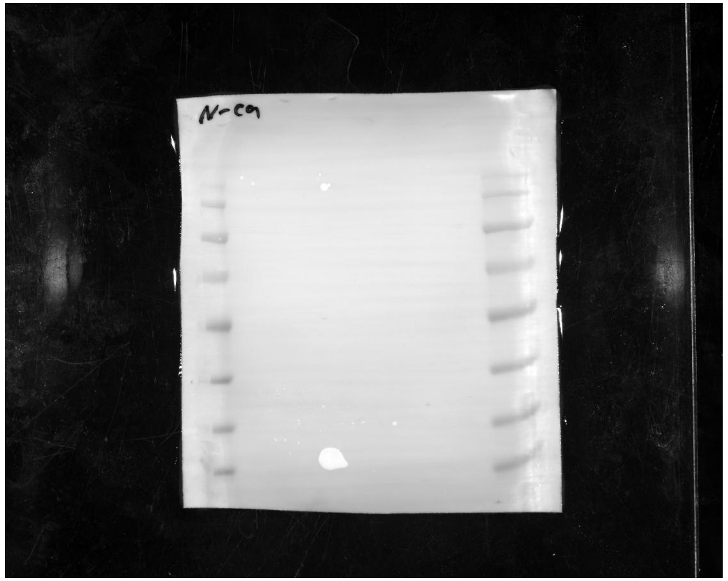

**H1299MMP9**

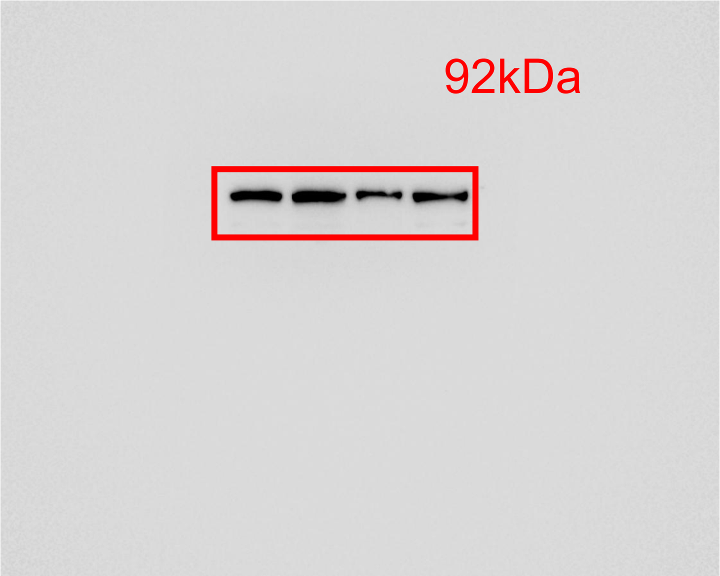


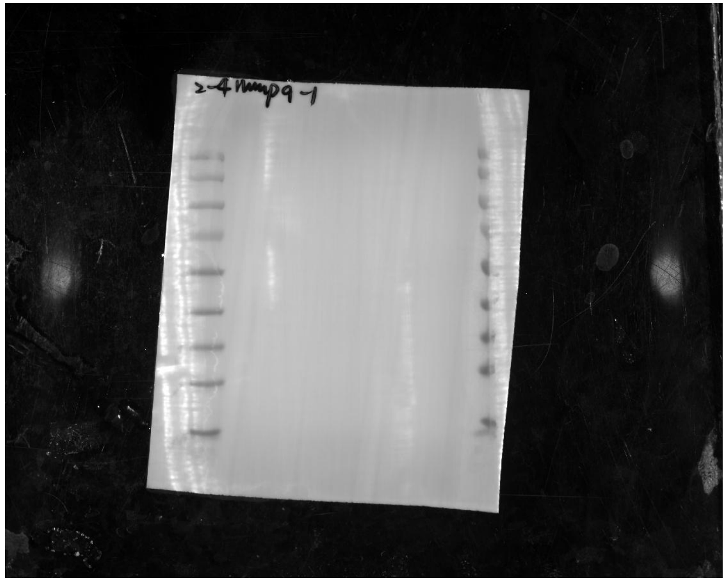

**A549MMP9**

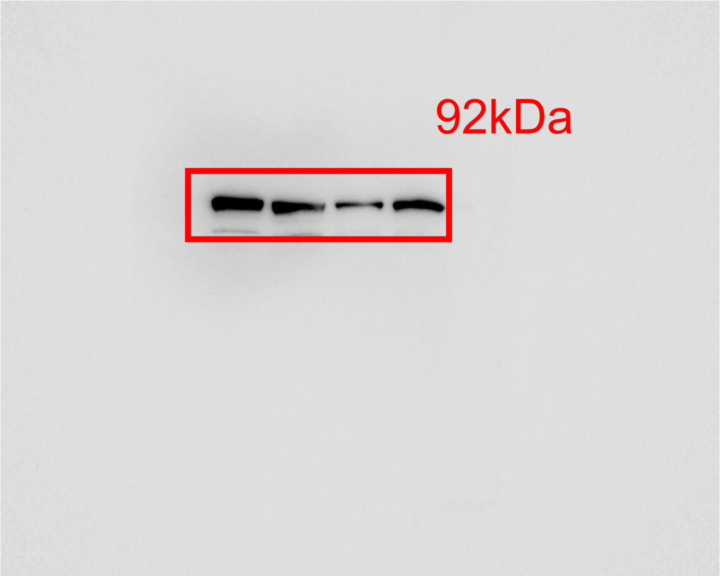


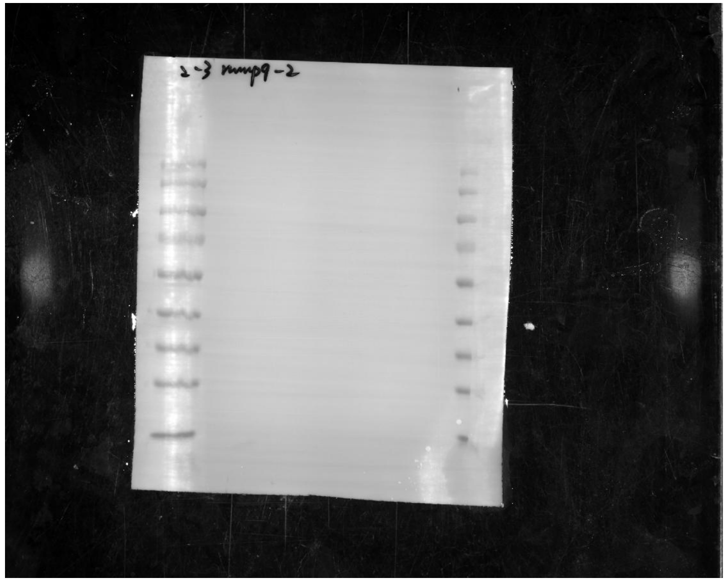

H1299GAPDH

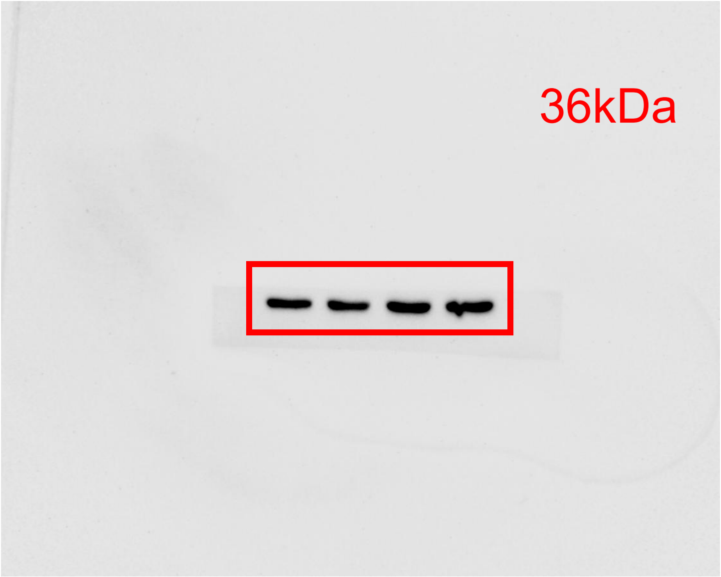


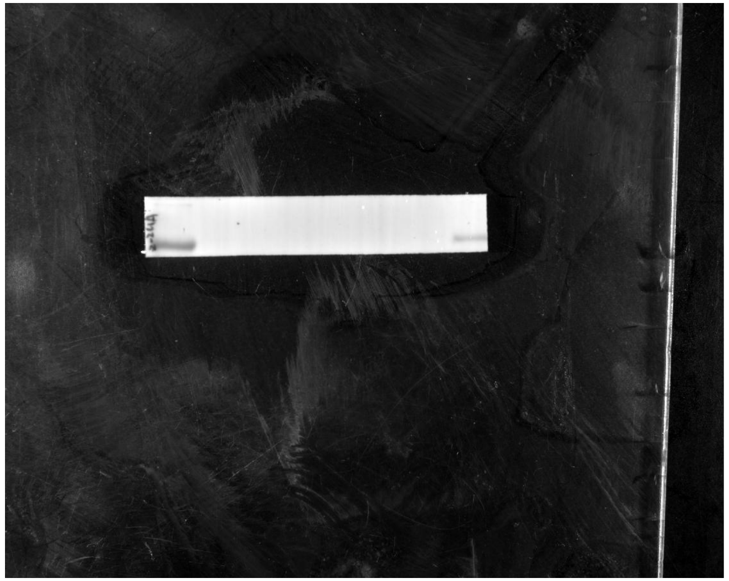

A549GAPDH

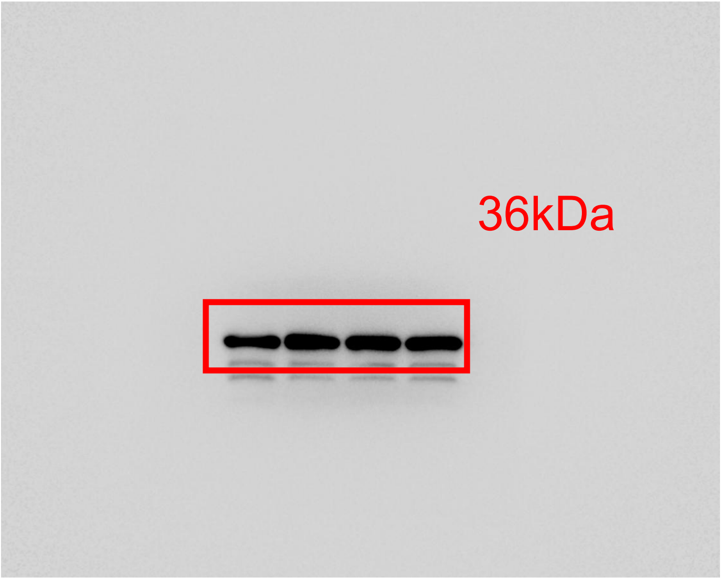


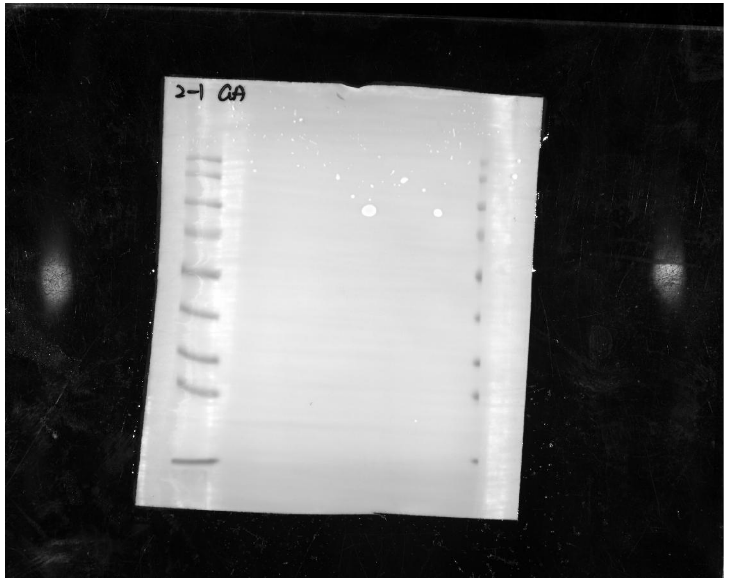

# S-5F

H1299 Zo-1

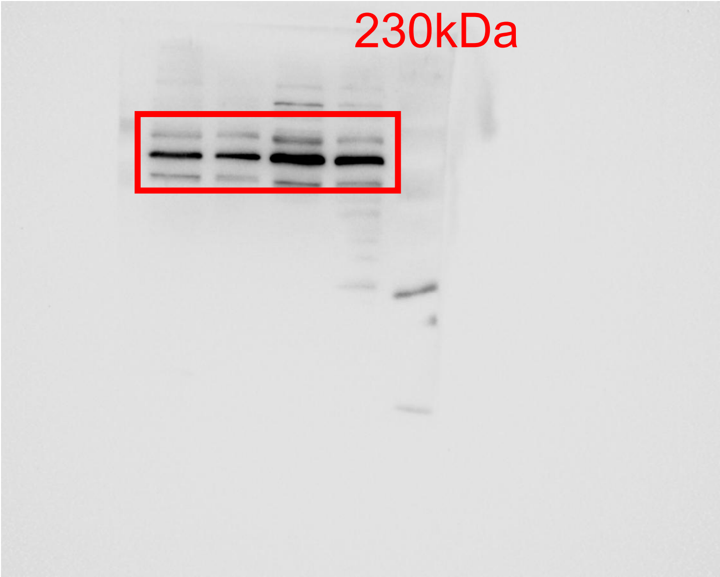


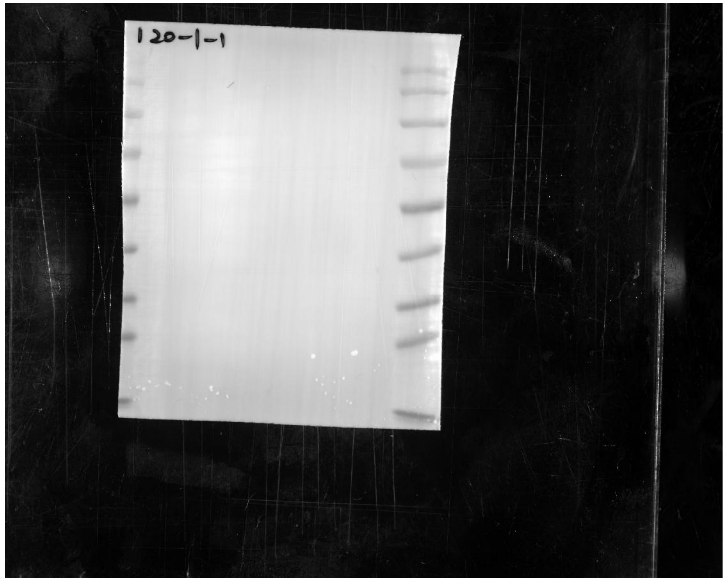


A549 Zo-1

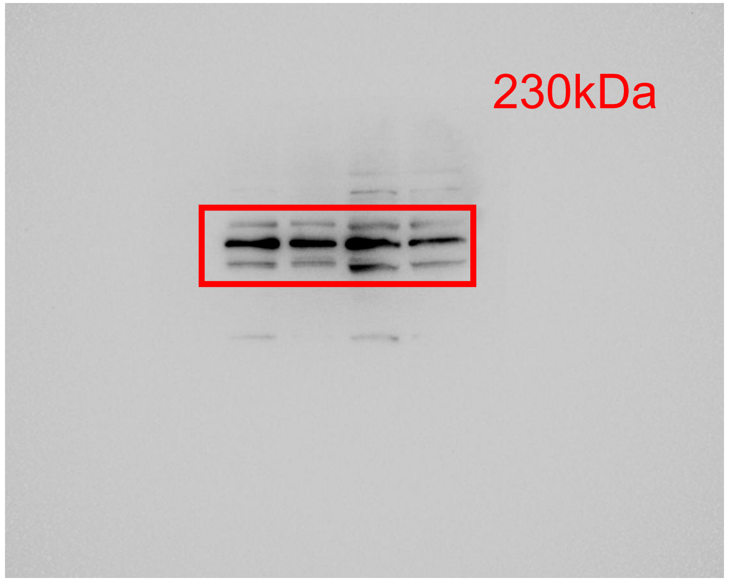


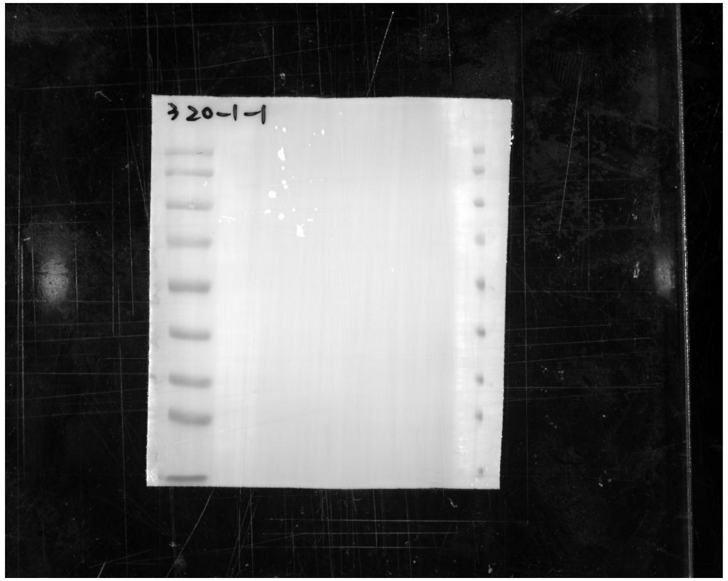

H1299 vimentin

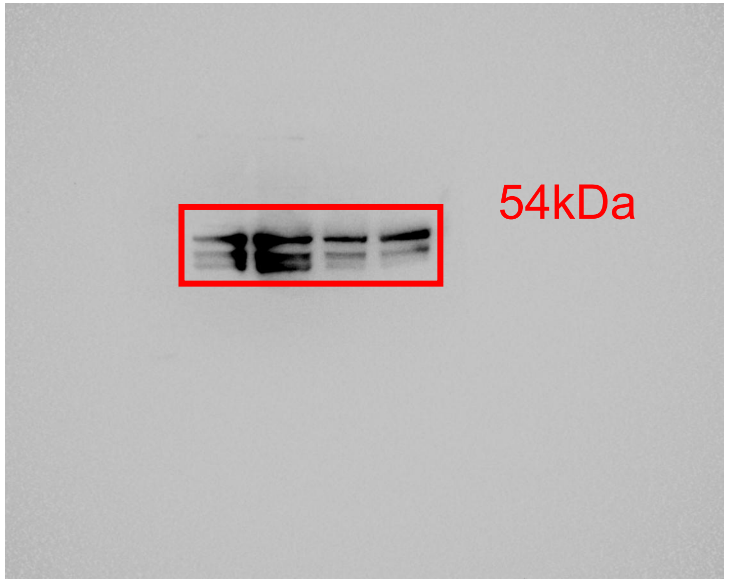


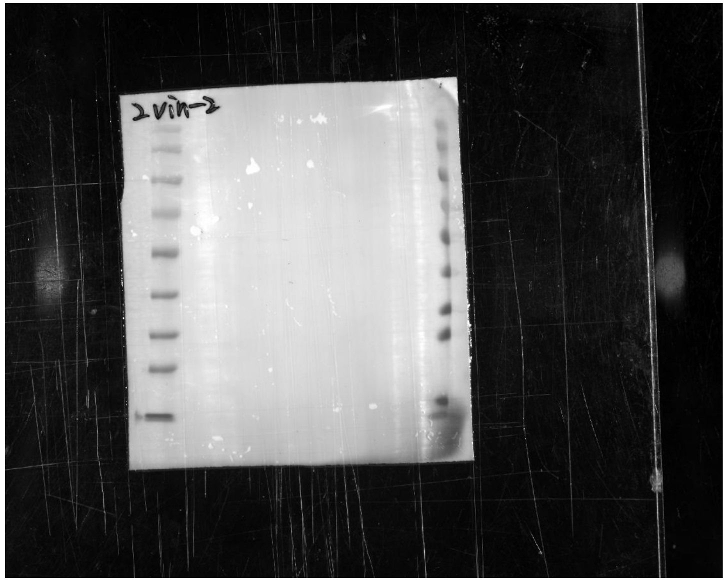


A549 vimentin

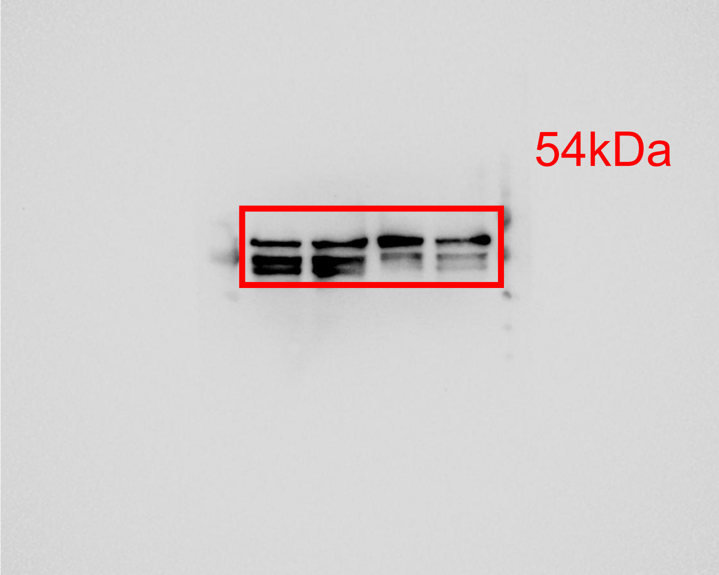


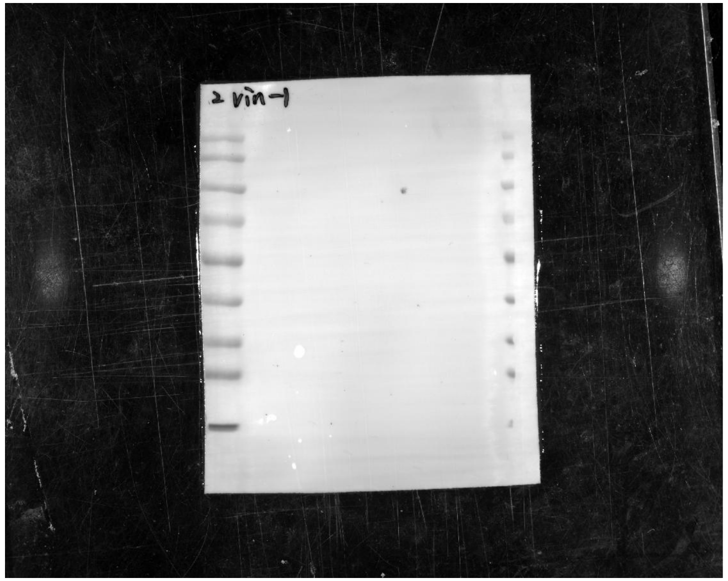

H1299 mmp2

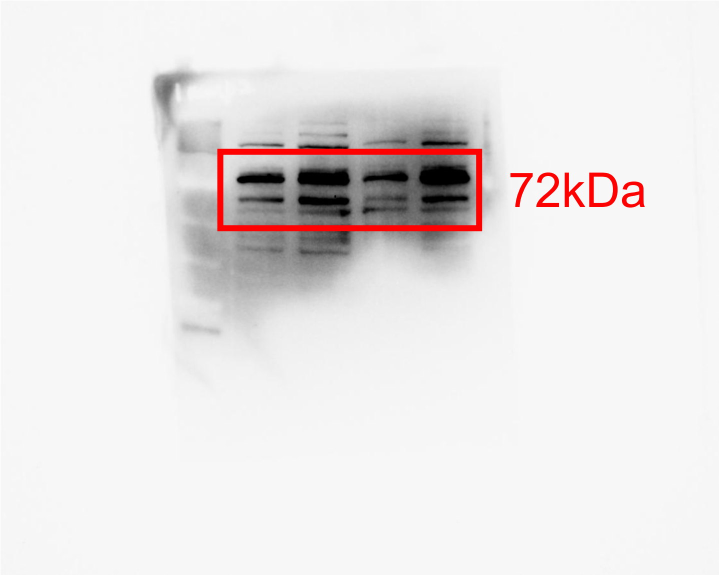


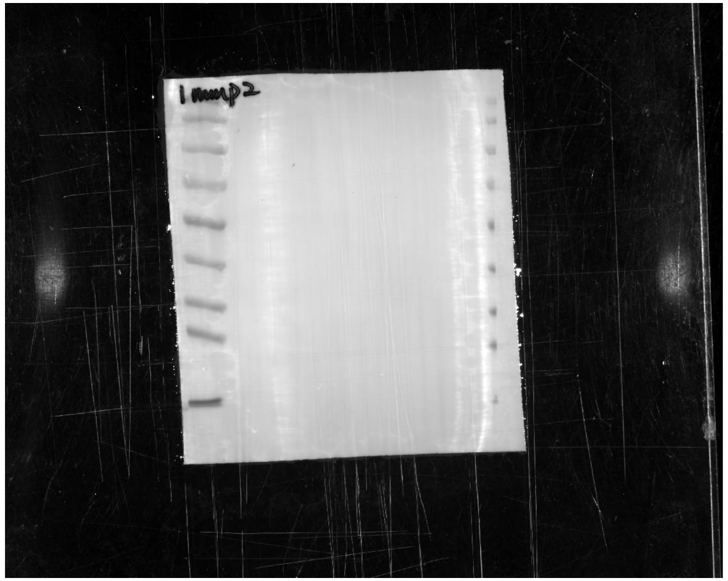

A549 mmp2

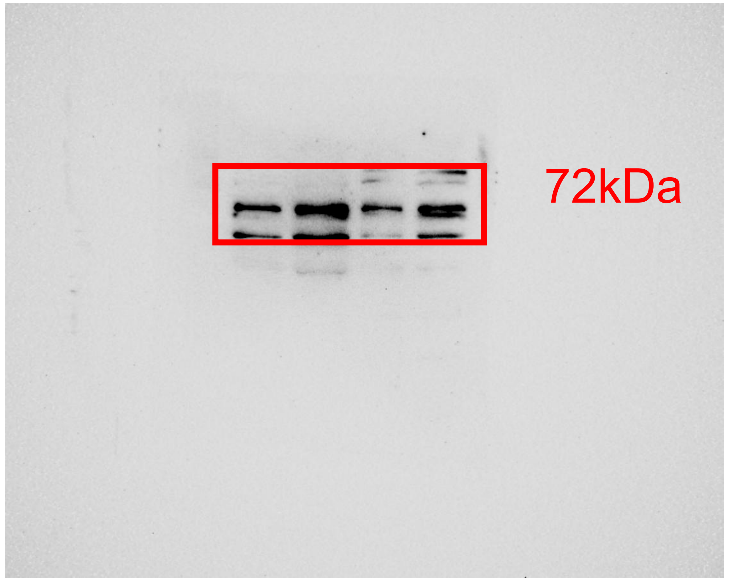


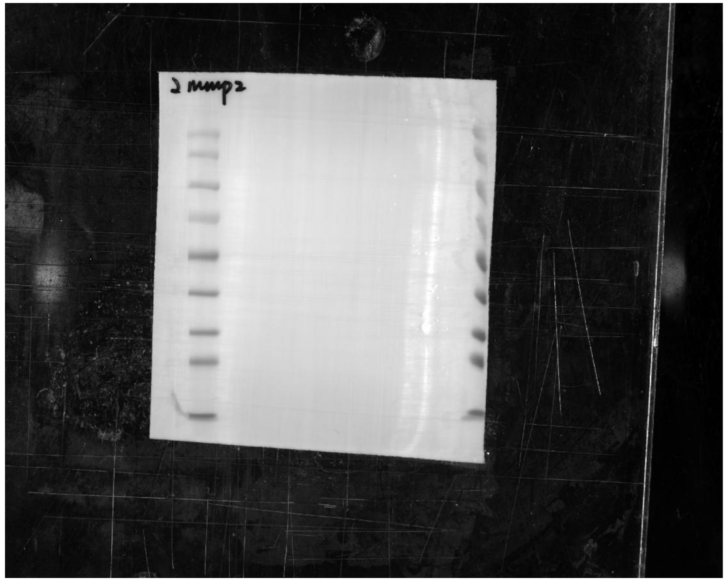

# Figure6B

myc

GAPDH

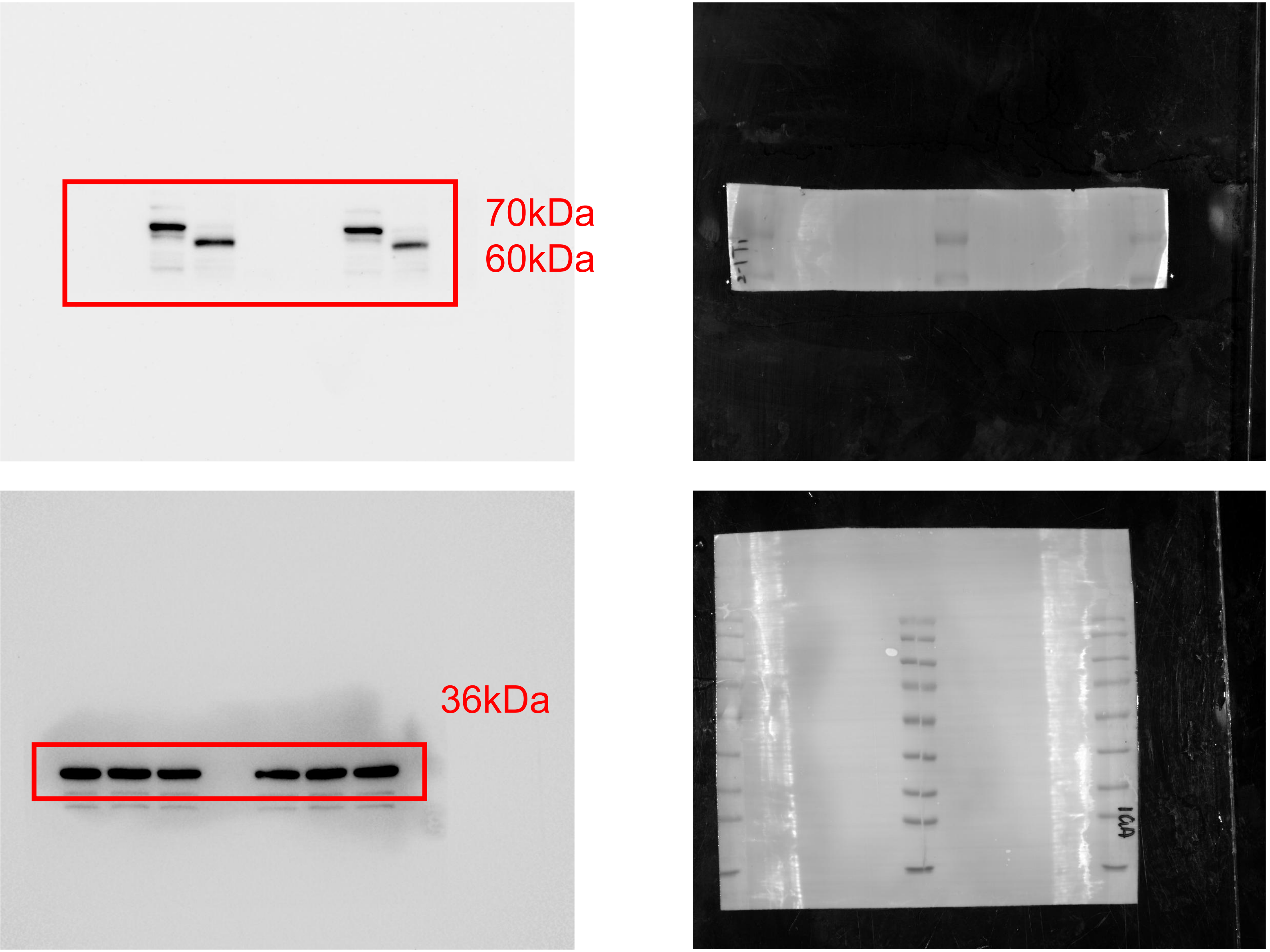

# Figure6C

ACTN4

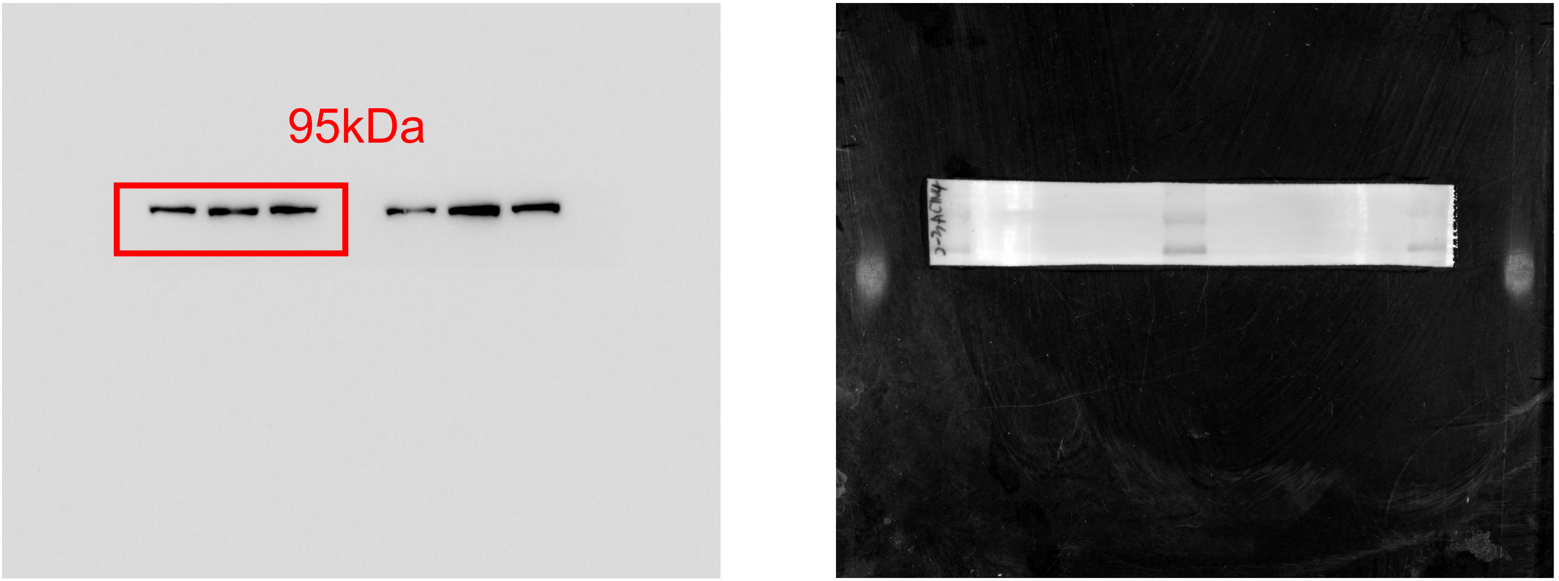


ACTN4

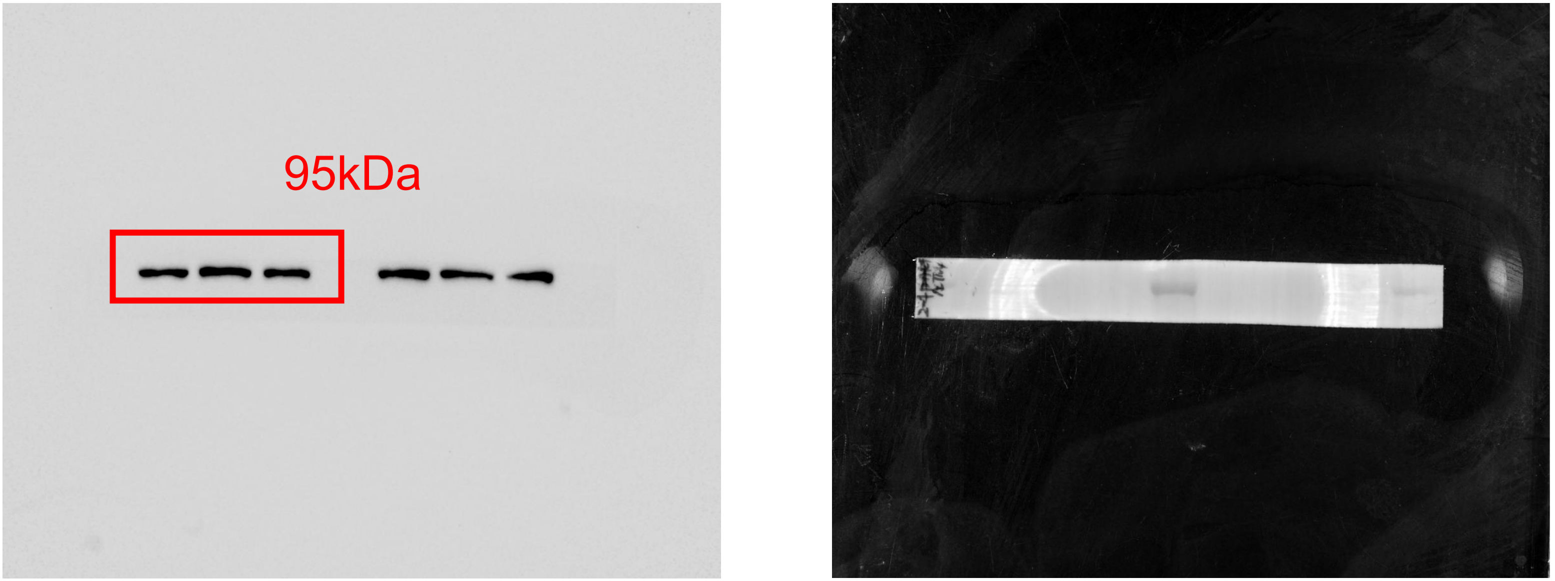

**myc**

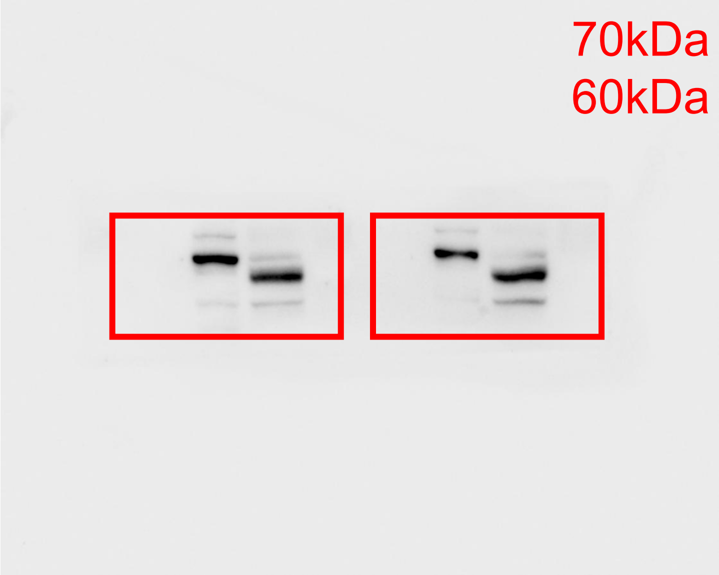


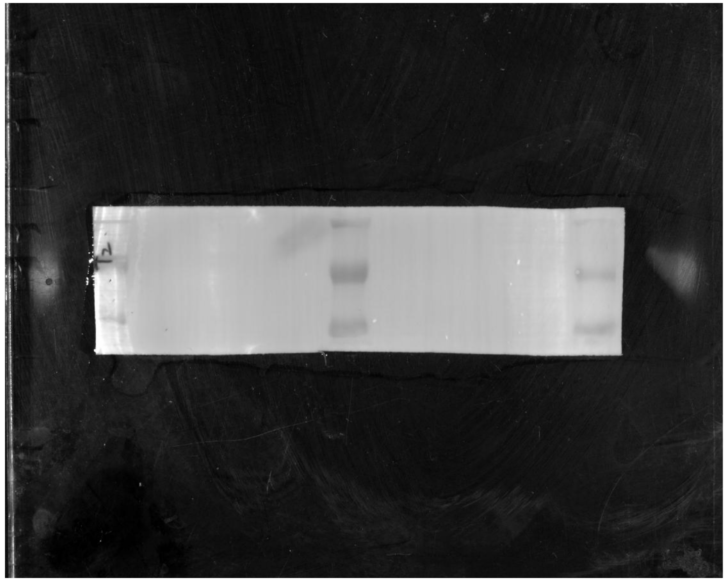

**INPUT: ACTN4**

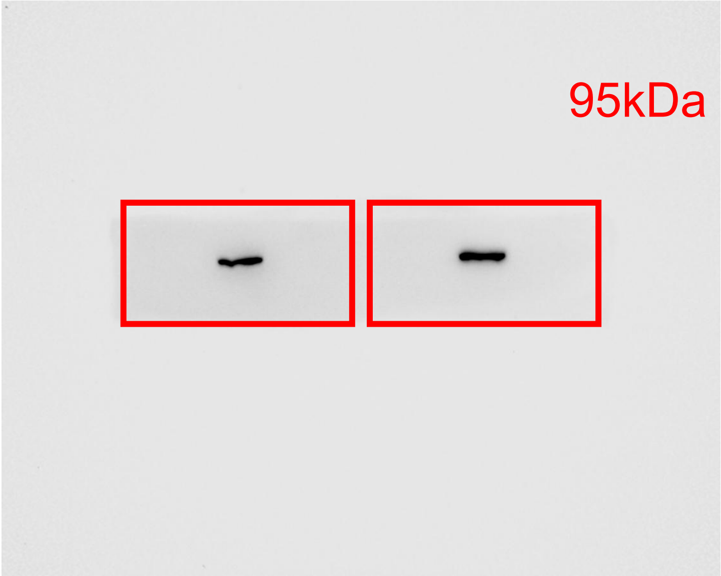


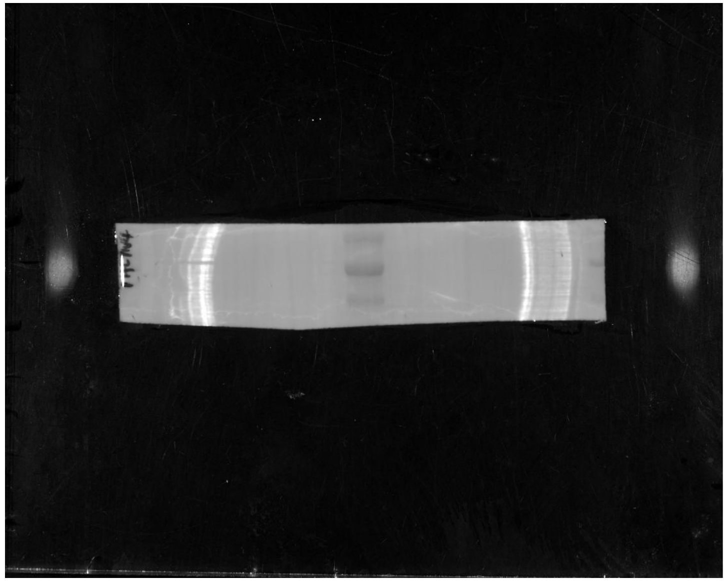

# Figure6D

**ACTN4**

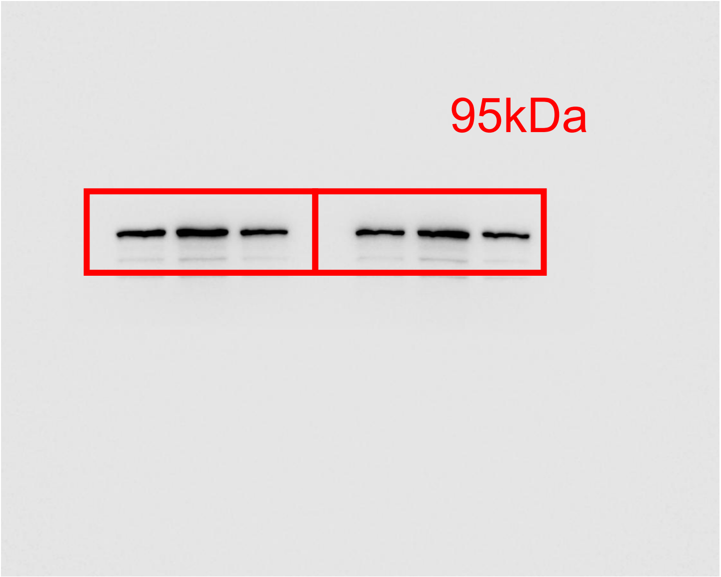


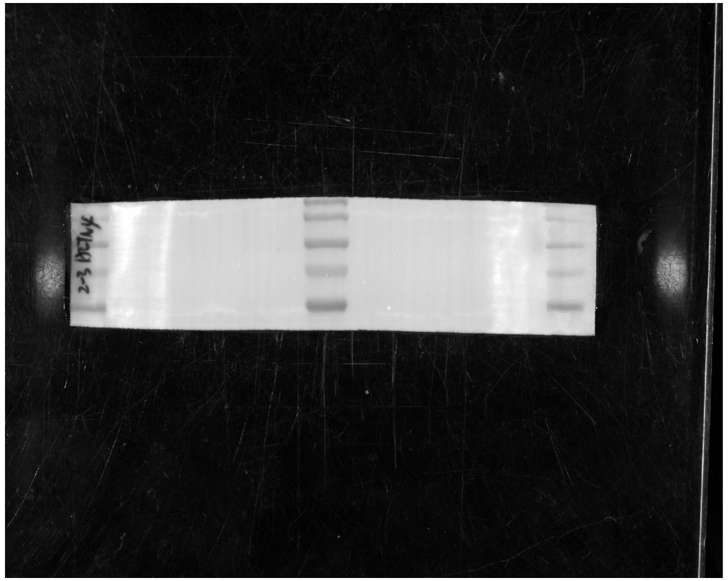

**p-ERK**

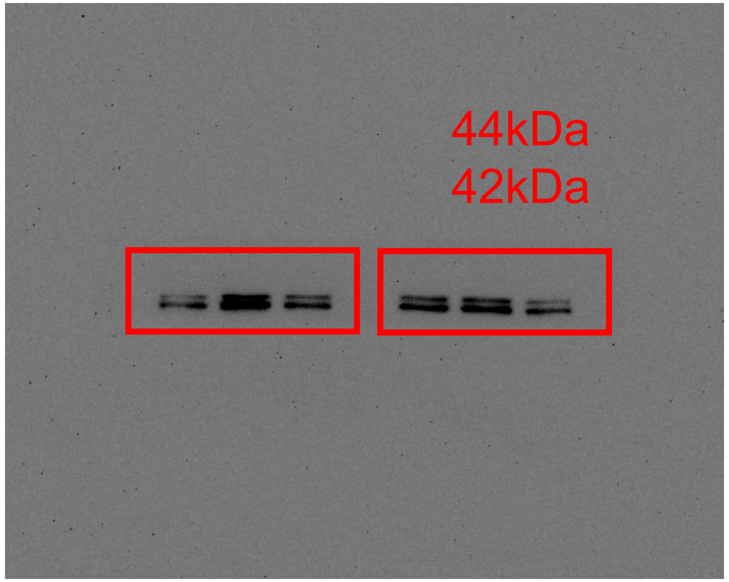


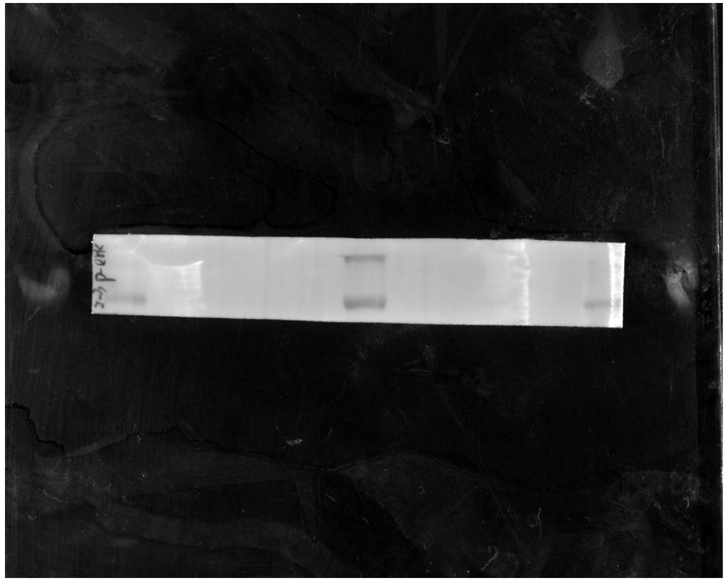

ERK

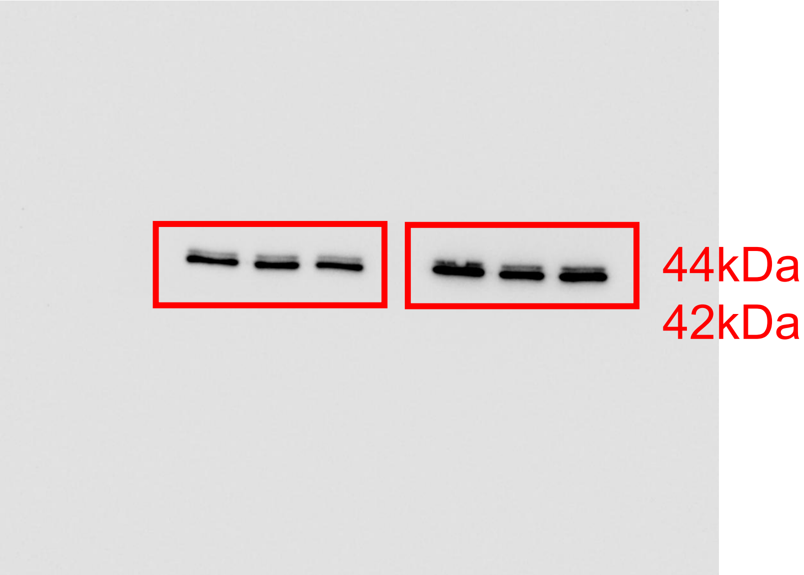


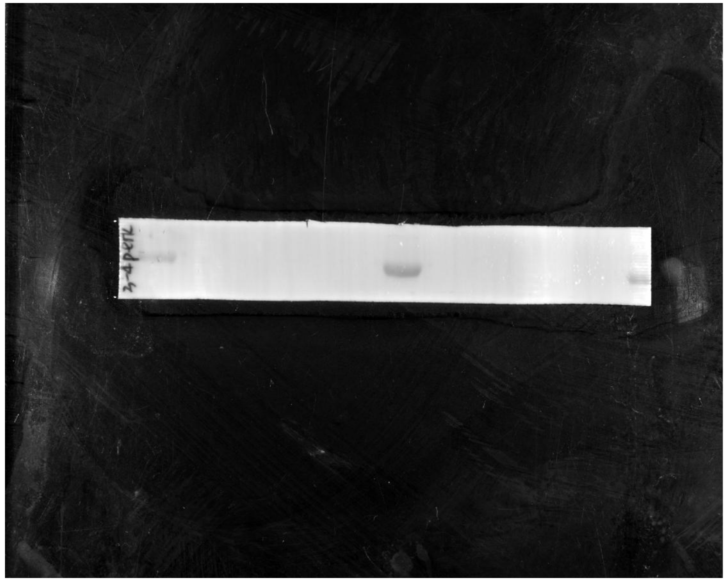

H1299p-jun（Ser73)

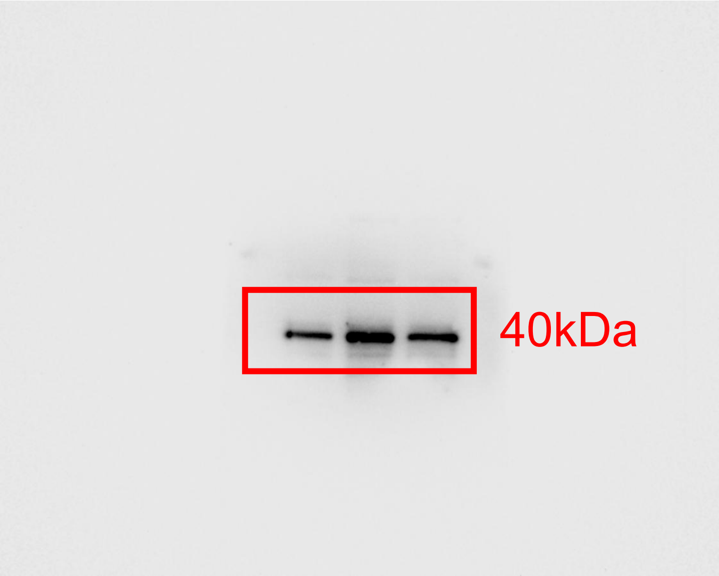


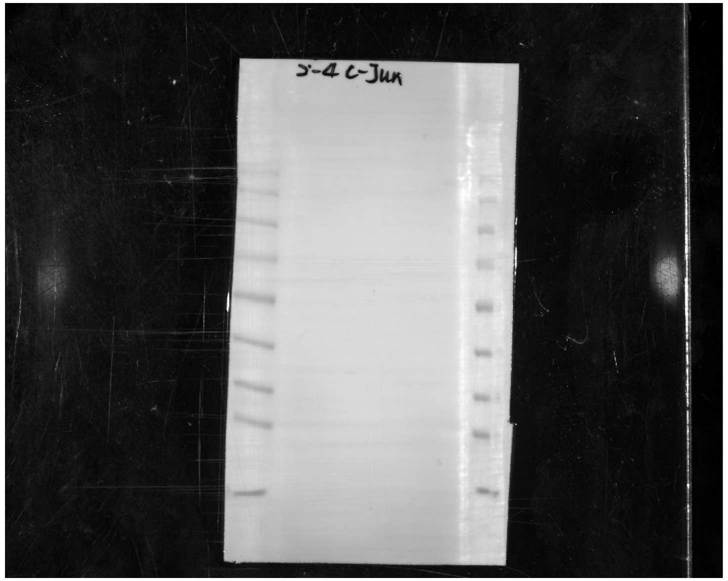

A549p-jun（Ser73）

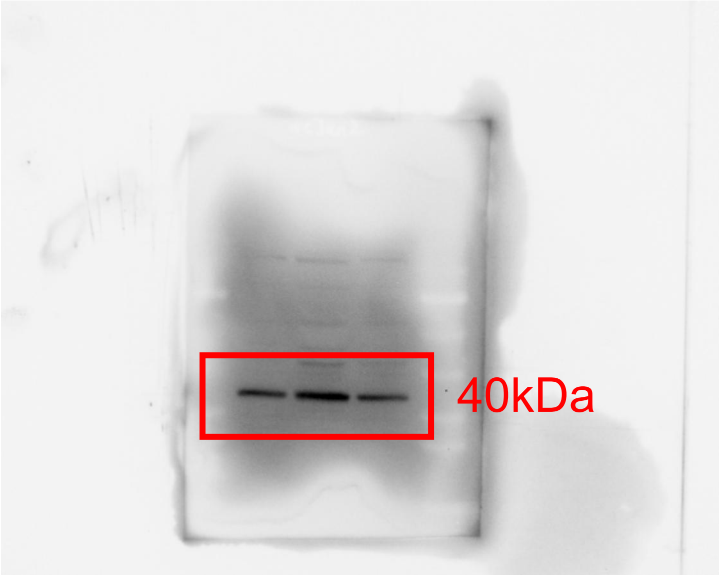


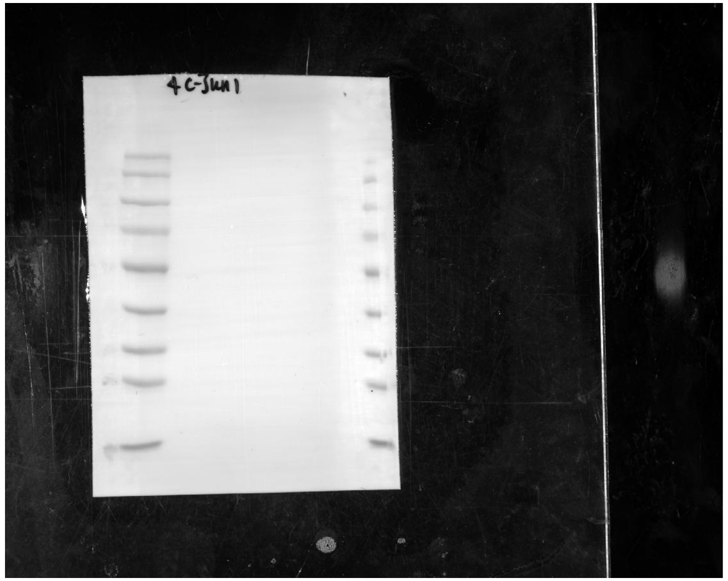

H1299c-myc

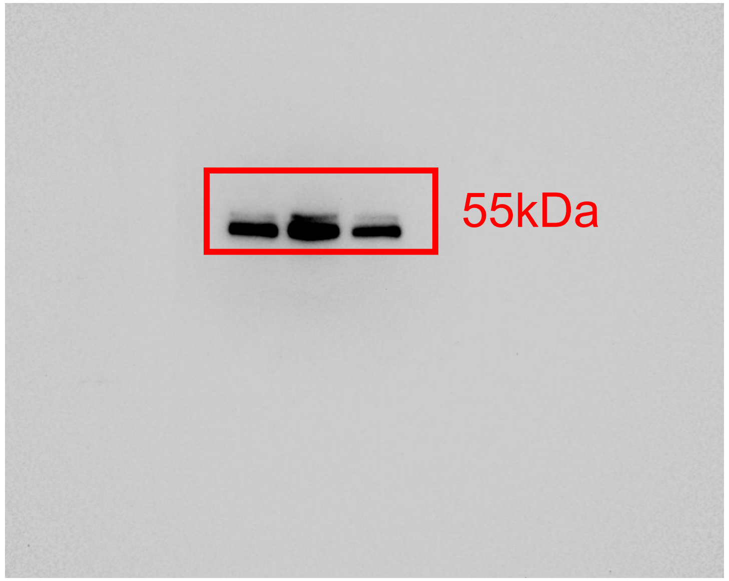


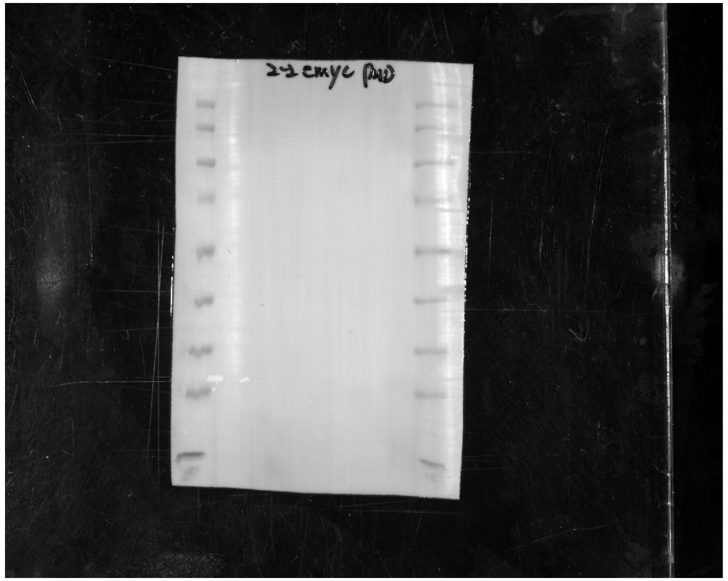

A549c-myc

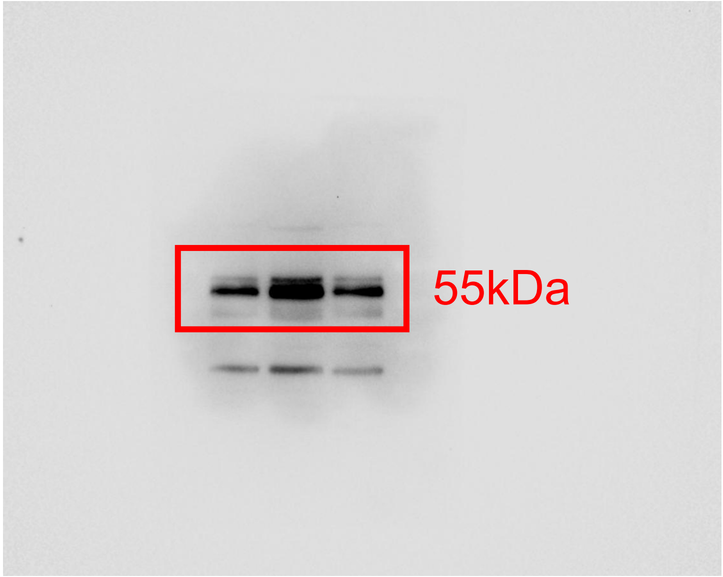


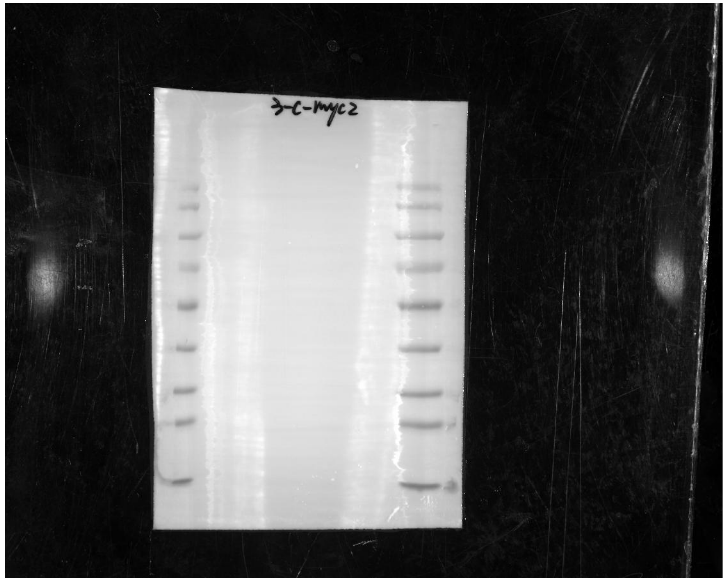

H1299p38

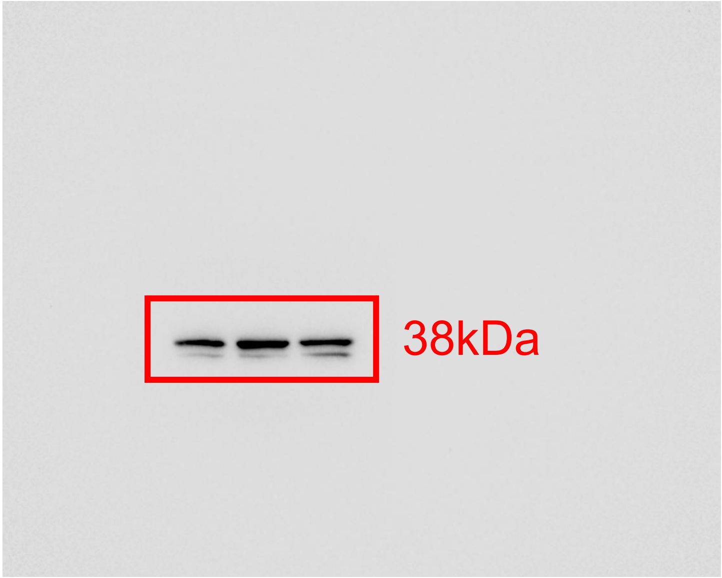


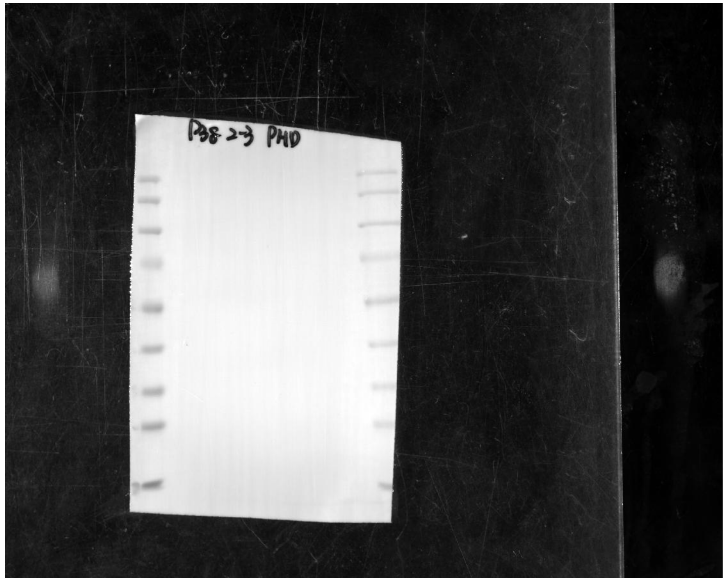

A549p38

GAPDH

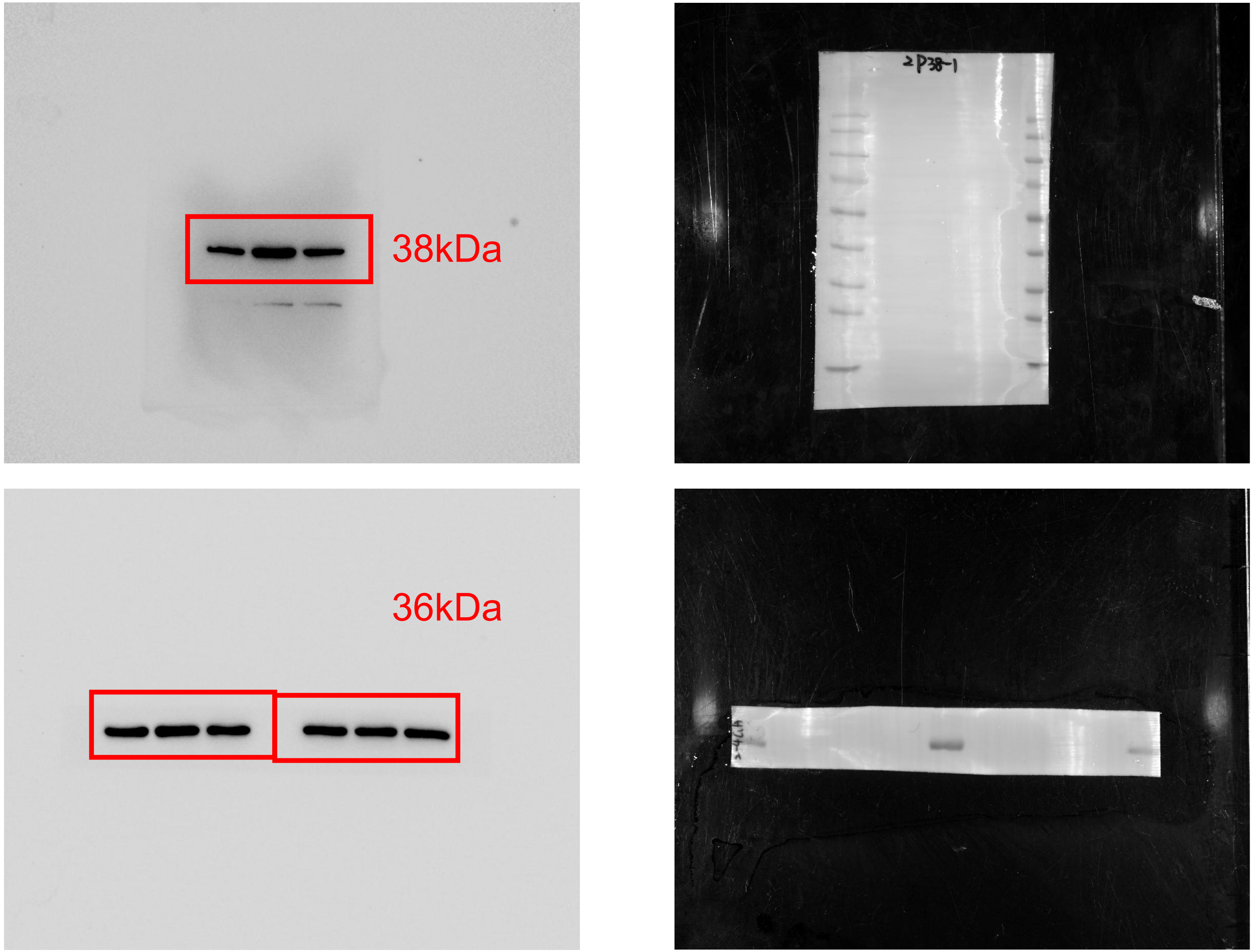

# S-Figure6D

cyclinA2

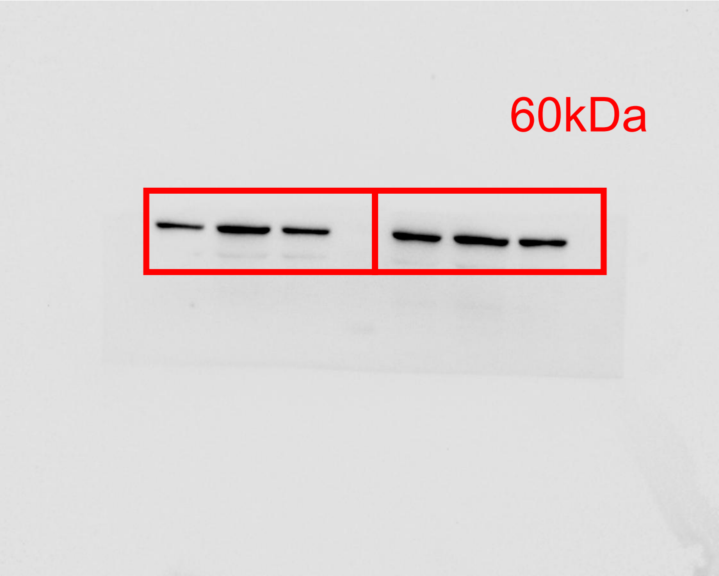


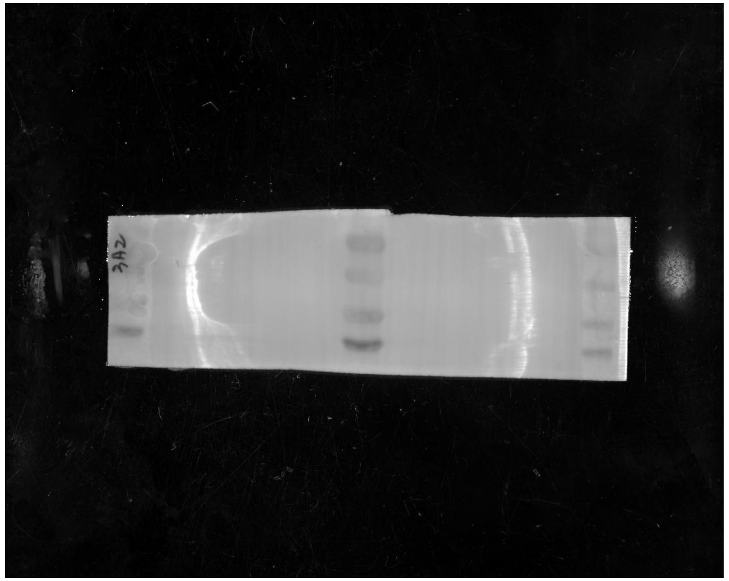

cyclinD1

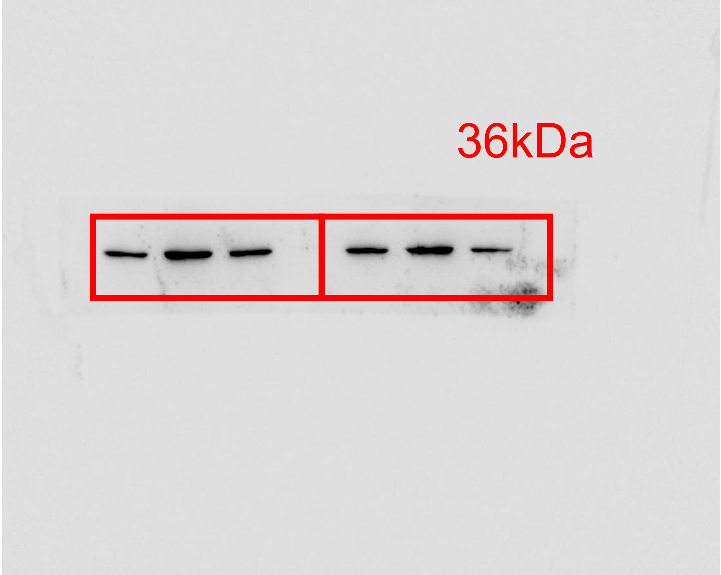


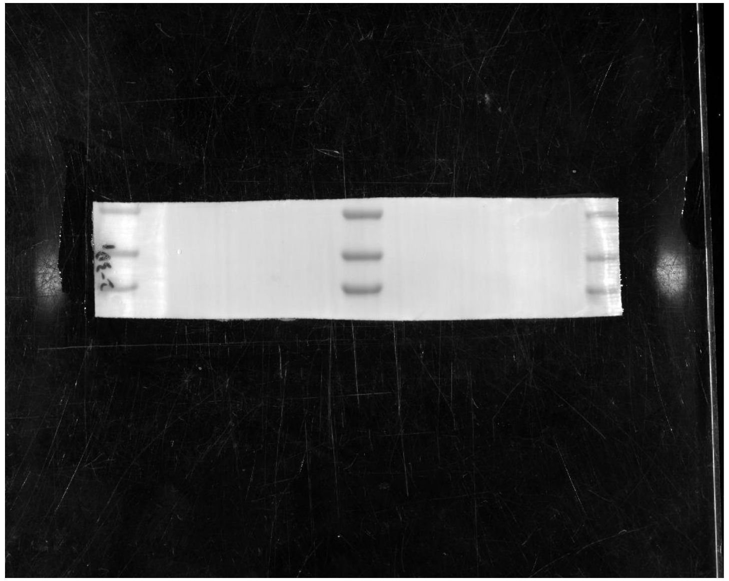

**H1299CDK4**

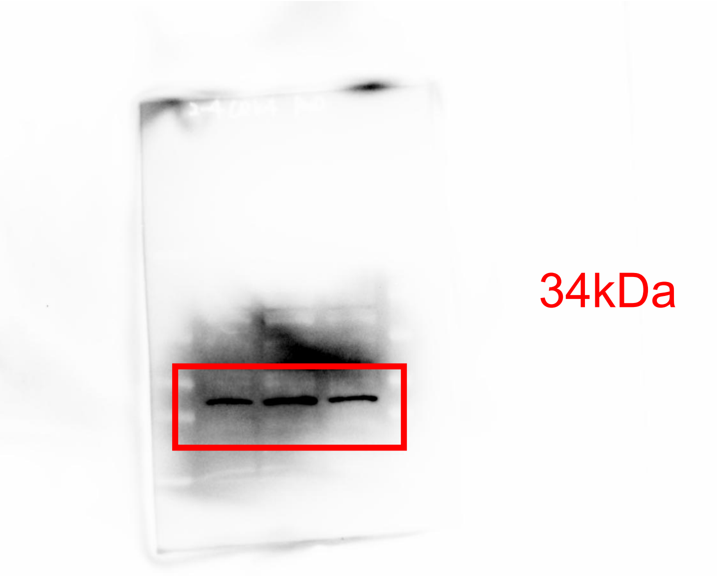


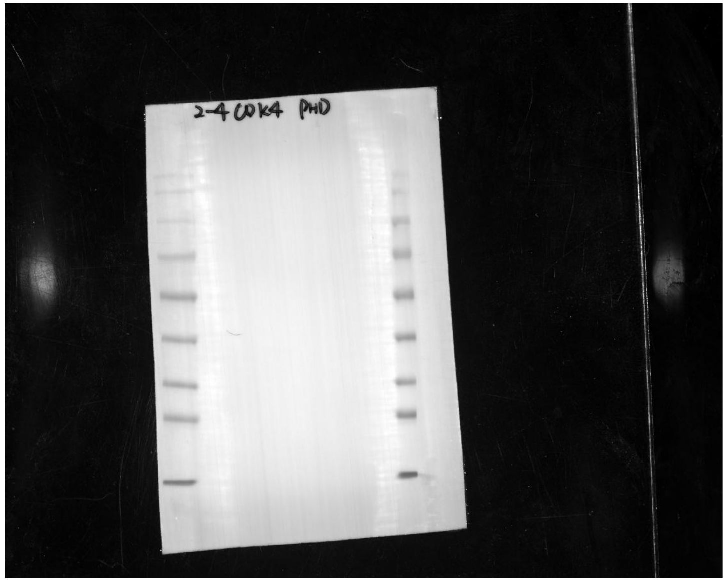

**A549CDK4**

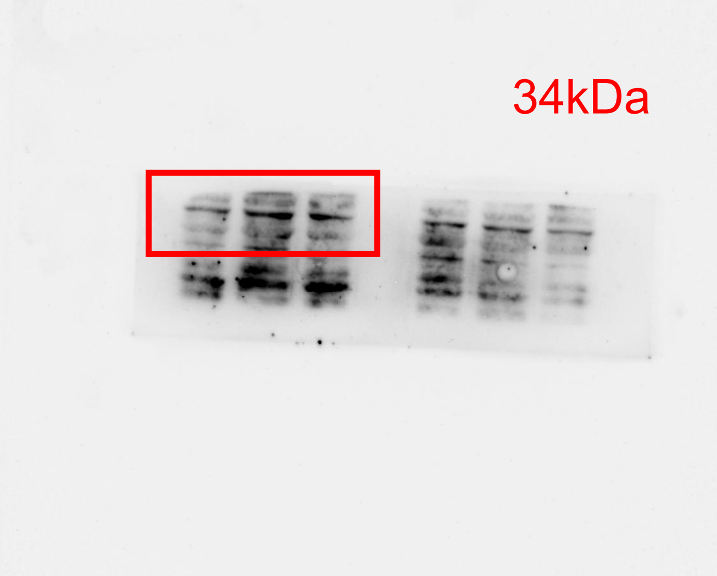


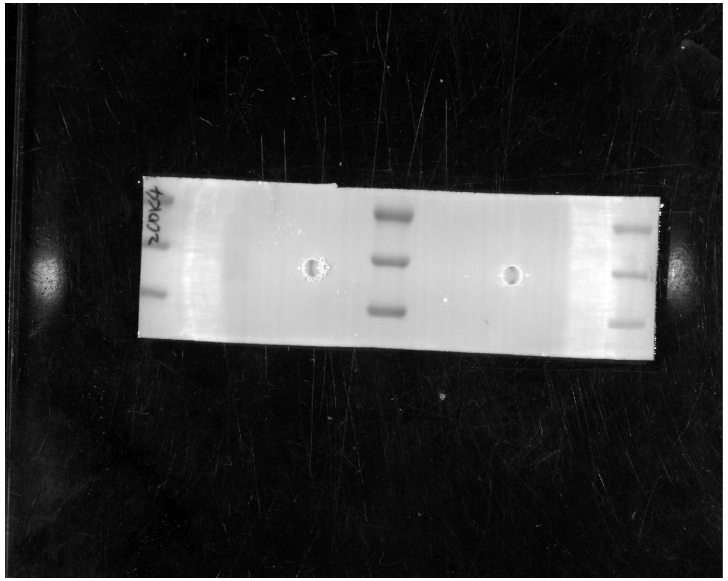

CDK6

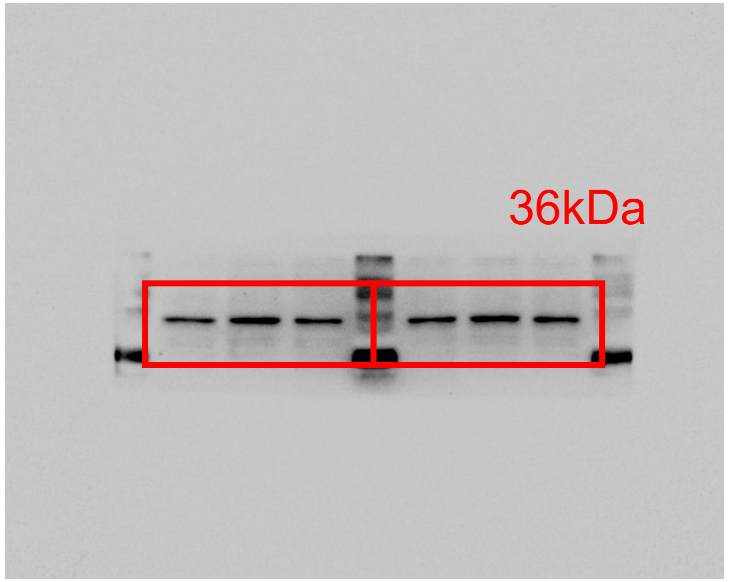


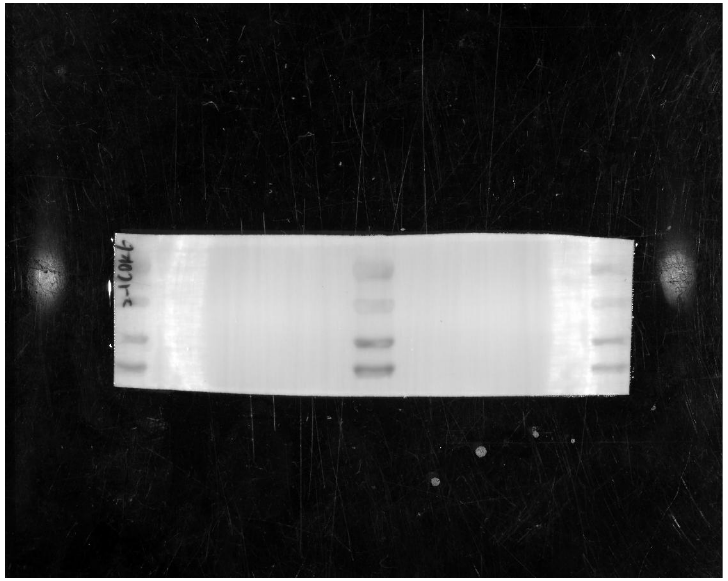

E-Cadherin

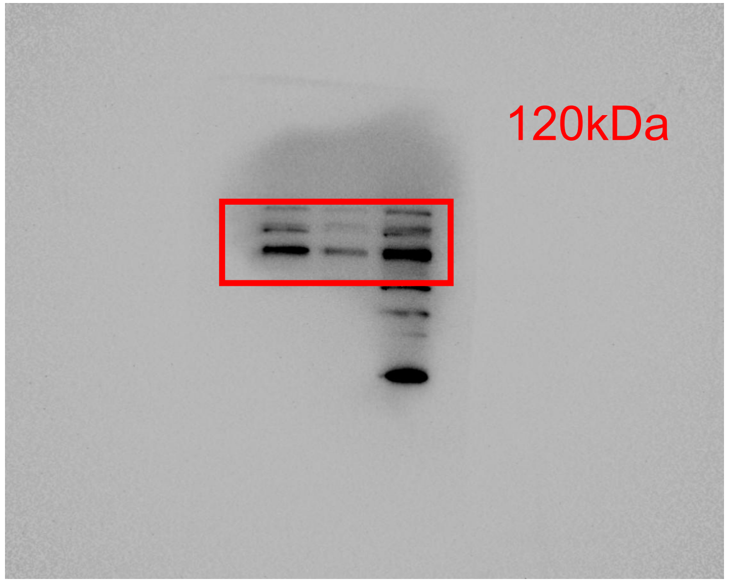


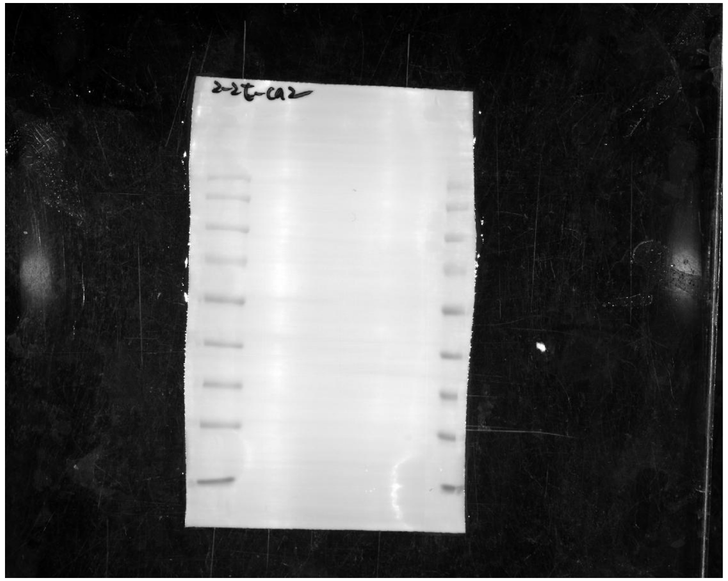

**E-Cadherin**

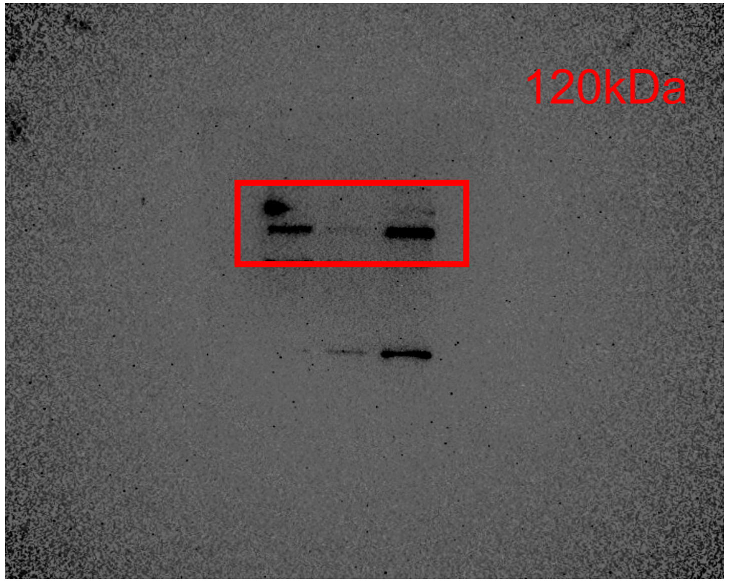


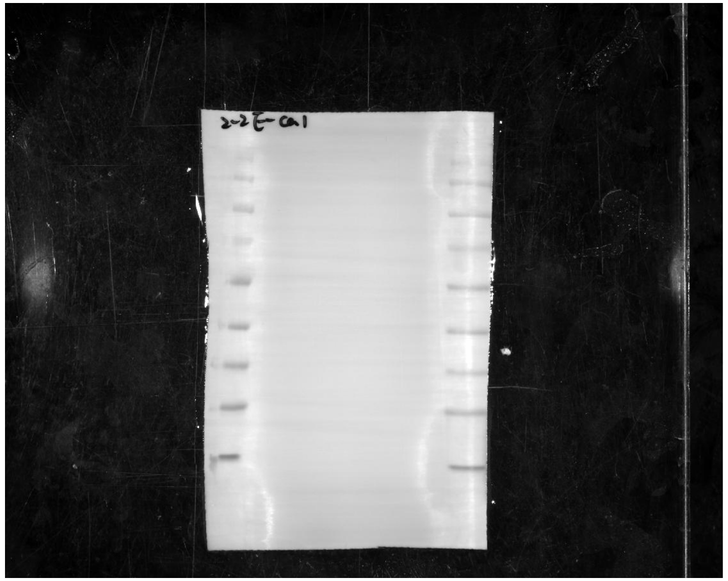

**N-Cadherin**

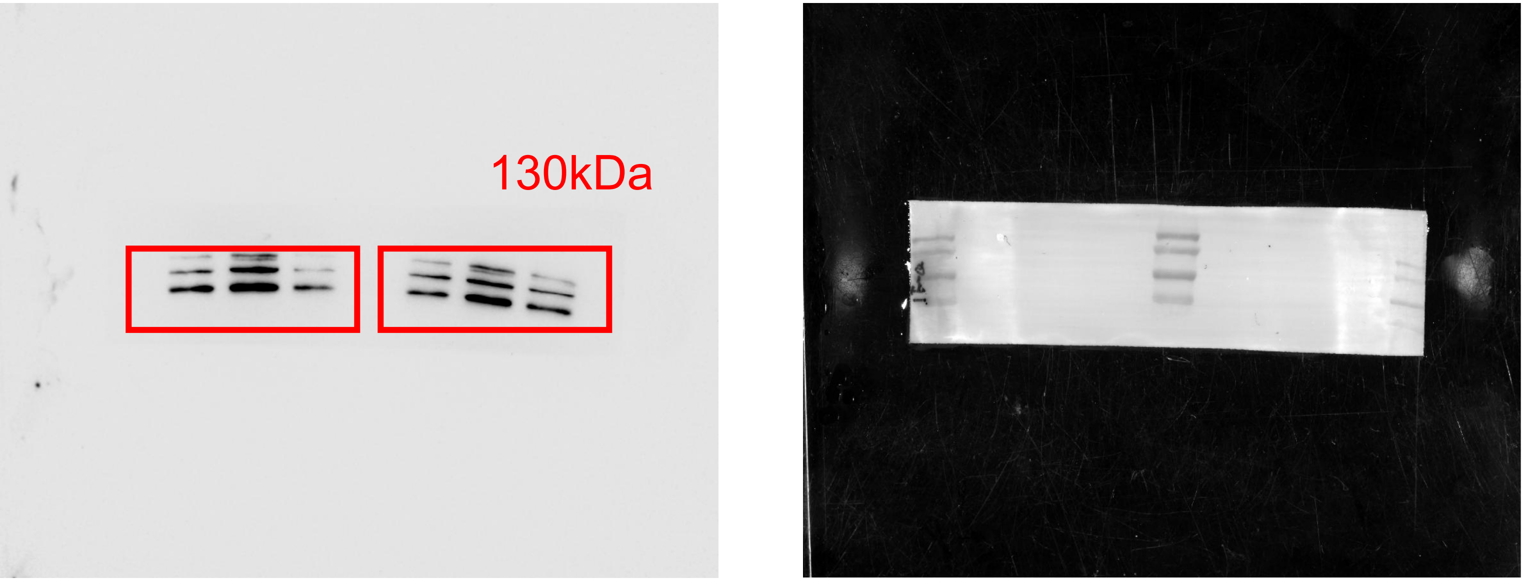


**MMP9**

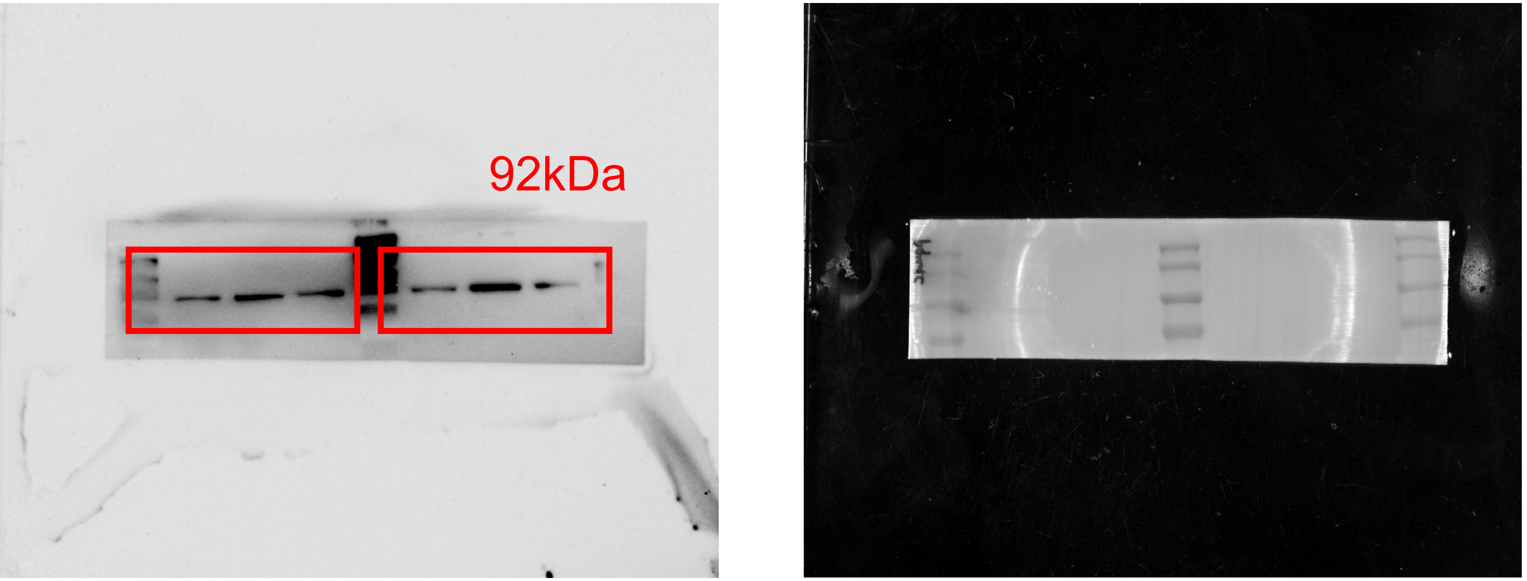

H1299 GAPDH

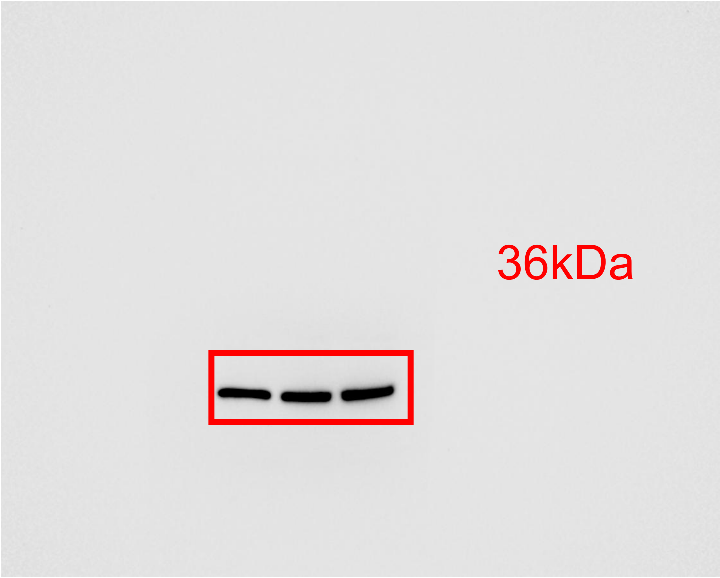


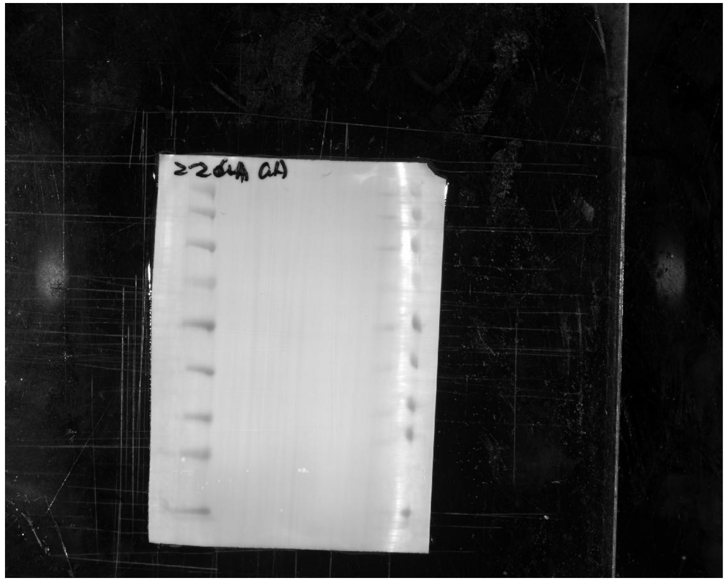

A549 GAPDH

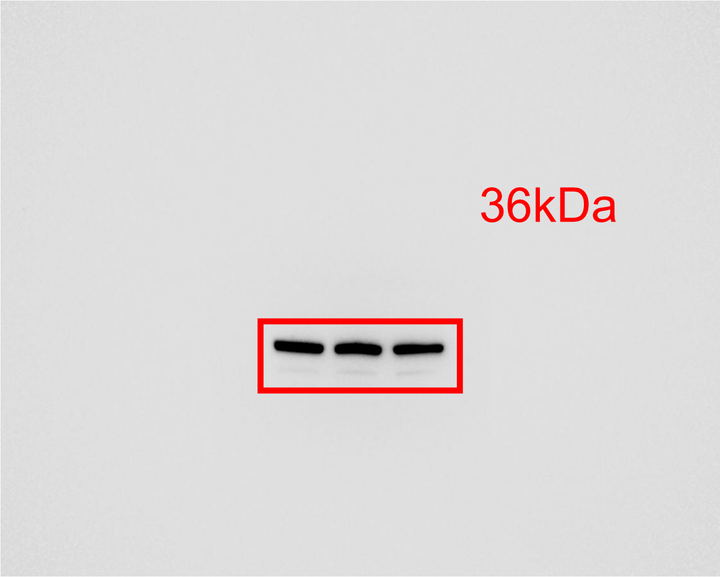


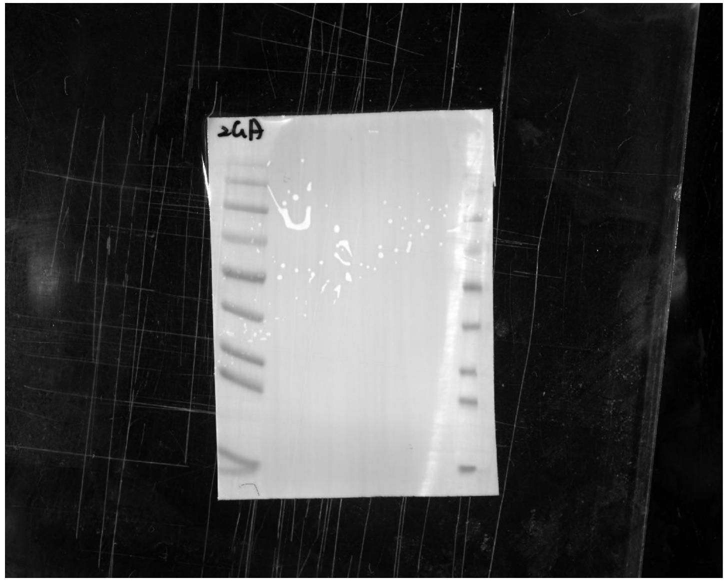

# S-6D

H1299 Zo-1

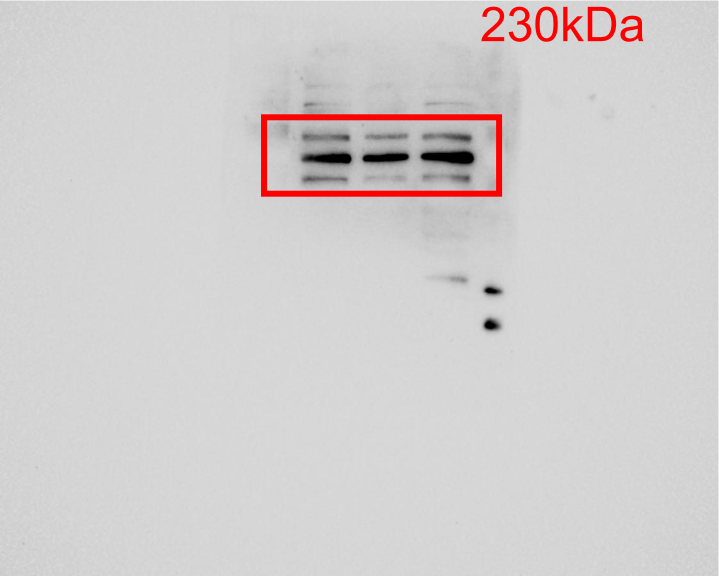


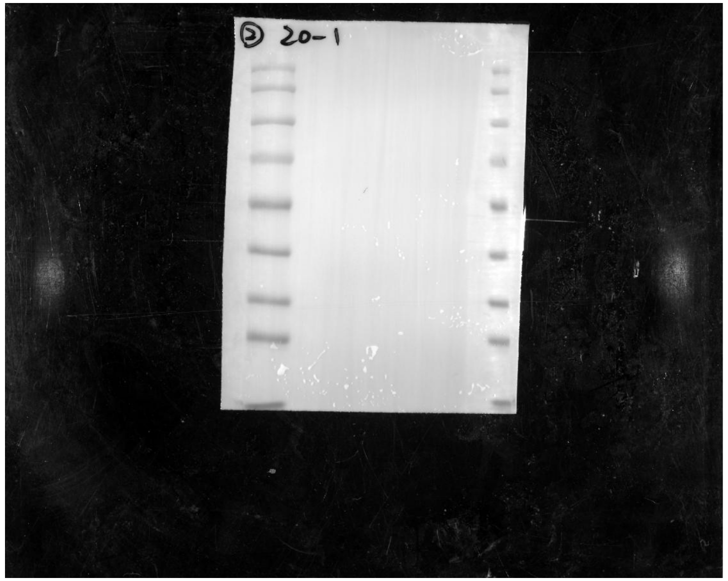

A549 Zo-1

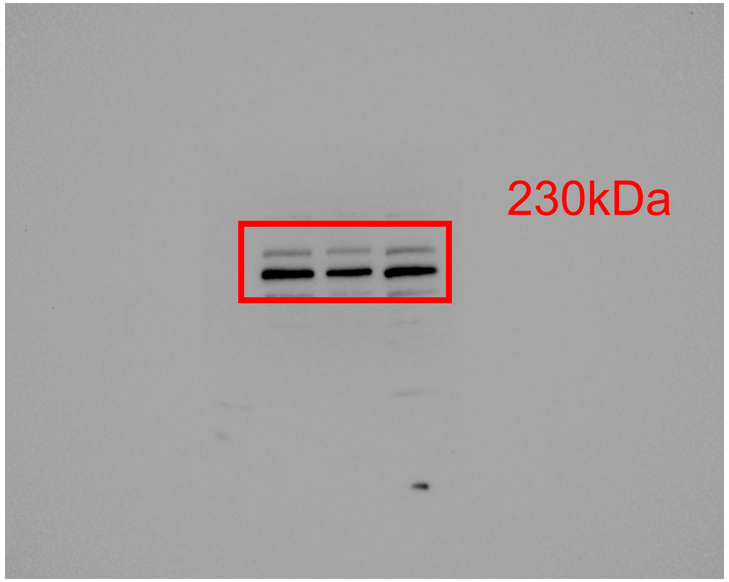


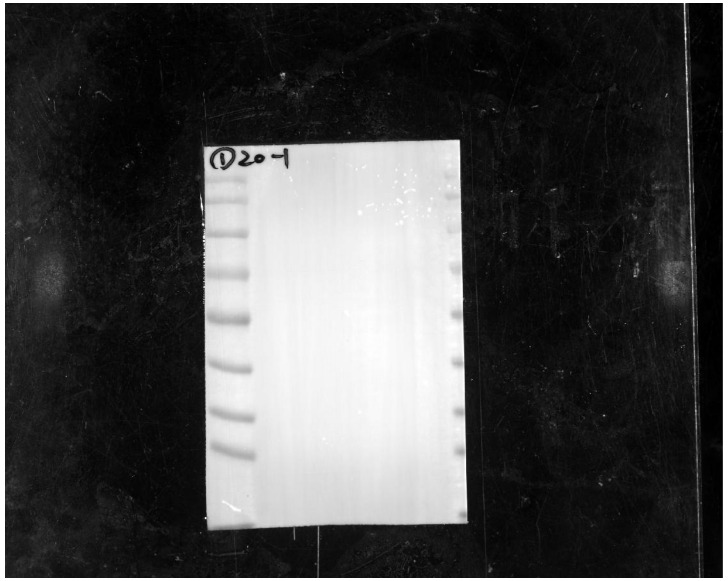

H1299
vimentin

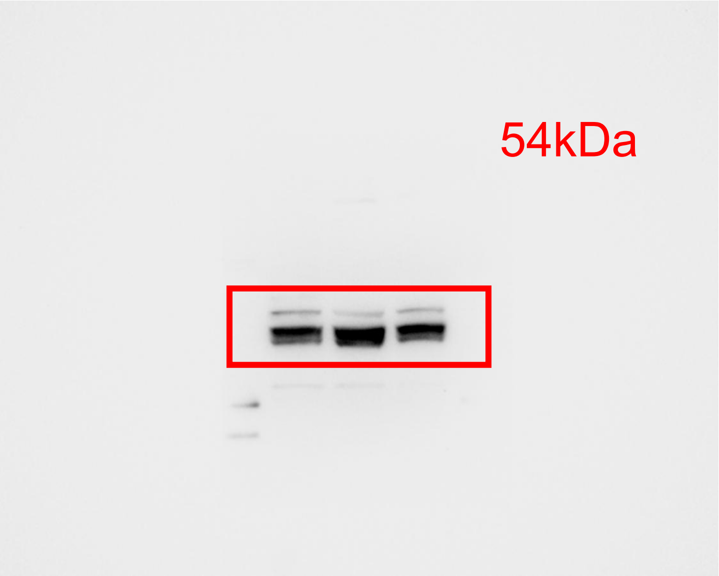


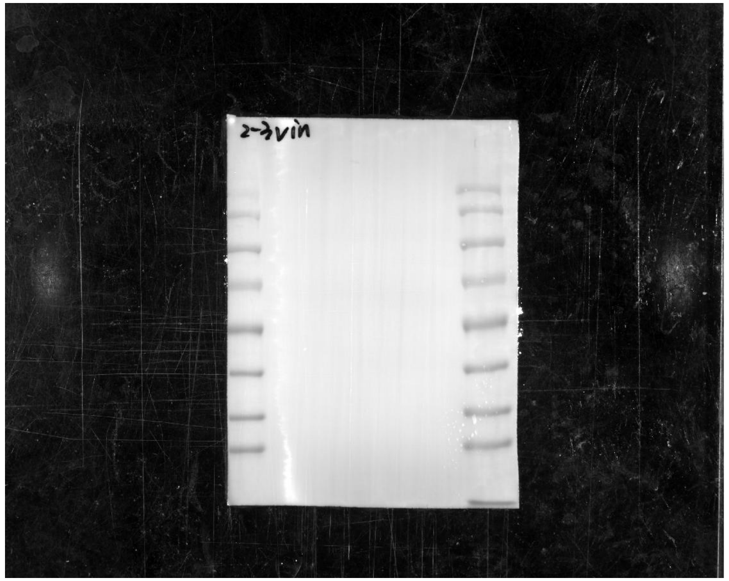


A549 vimentin

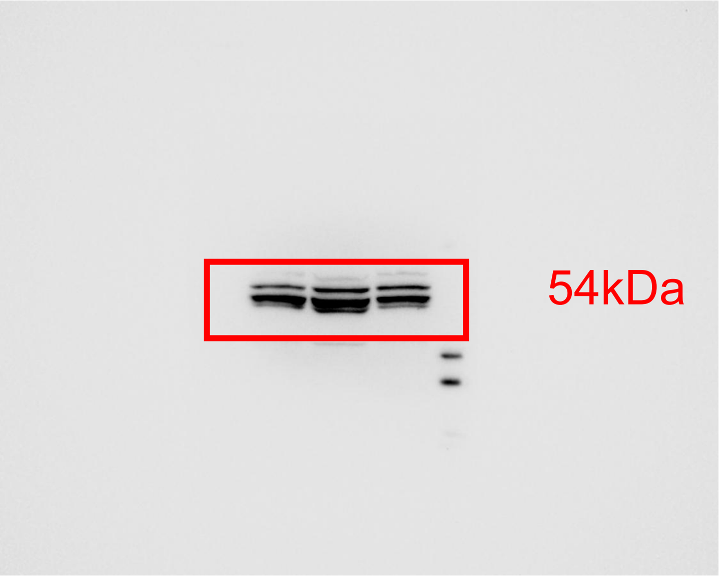


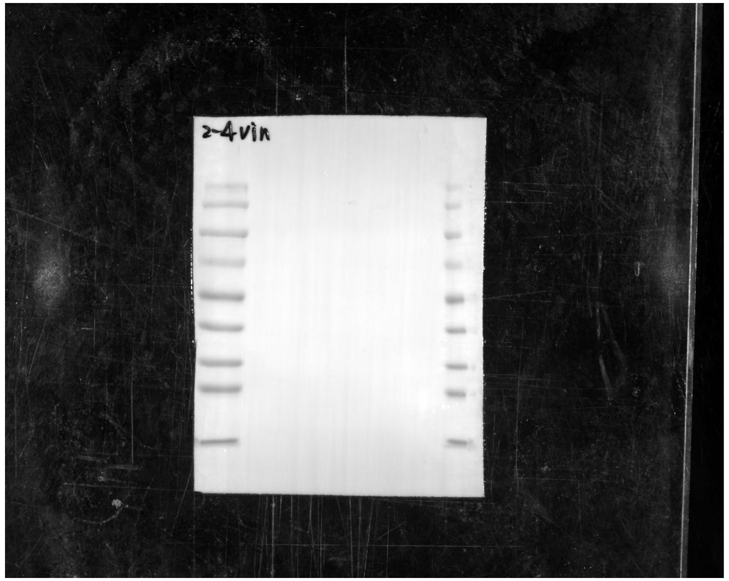

H1299 mmp2

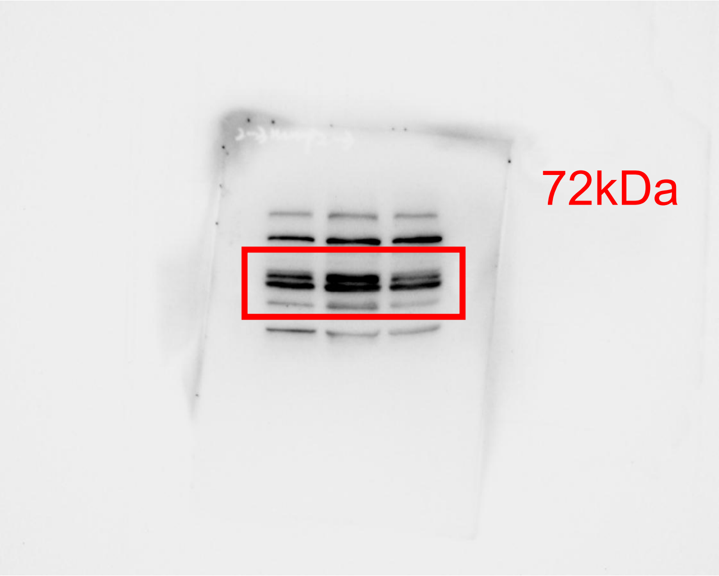


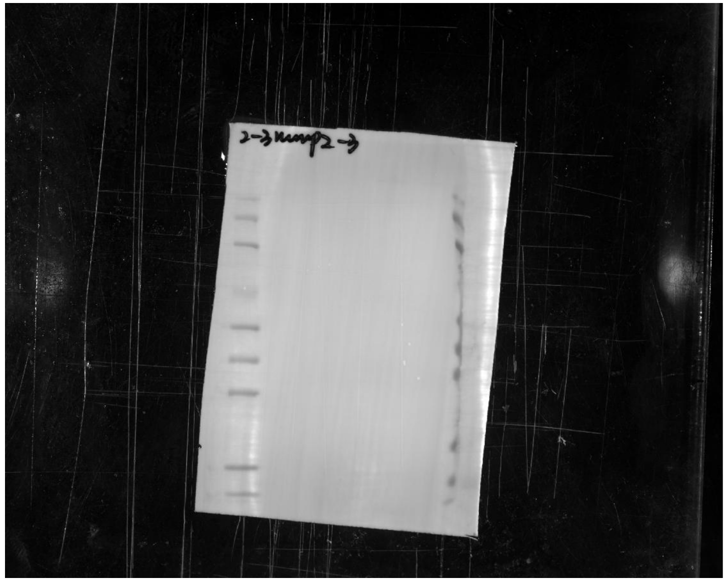

A549 mmp2

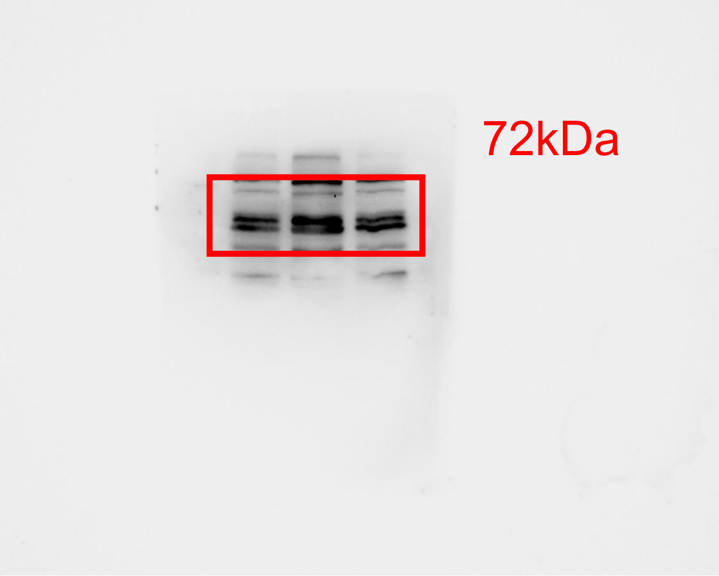


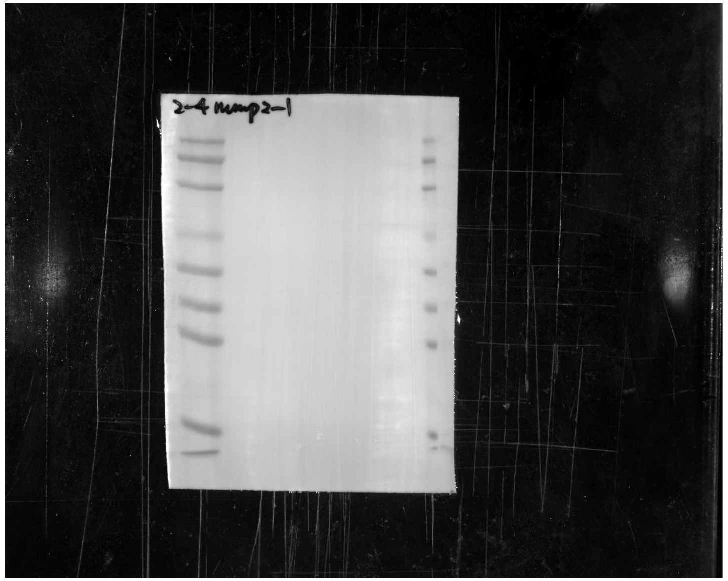

# Figure7A

H1299PHF23

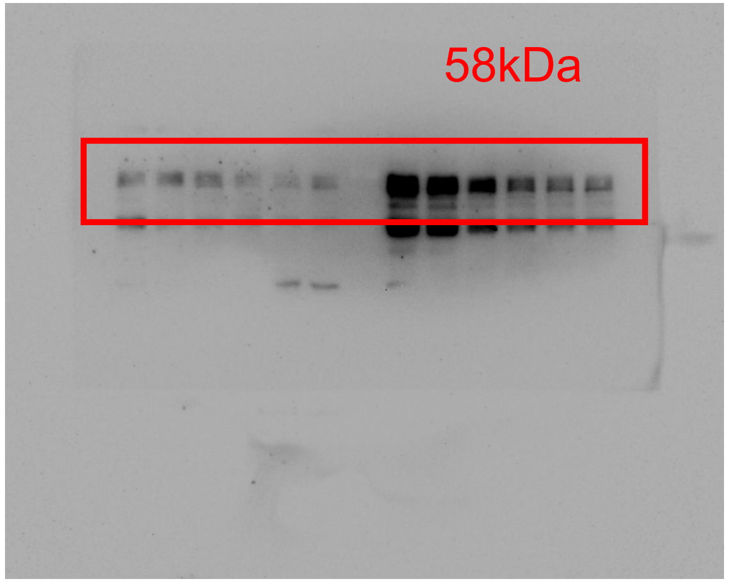


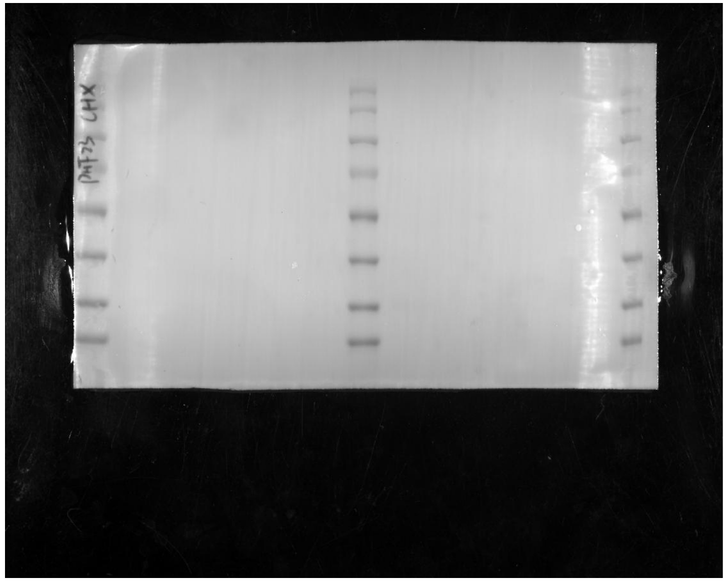

A549PHF23

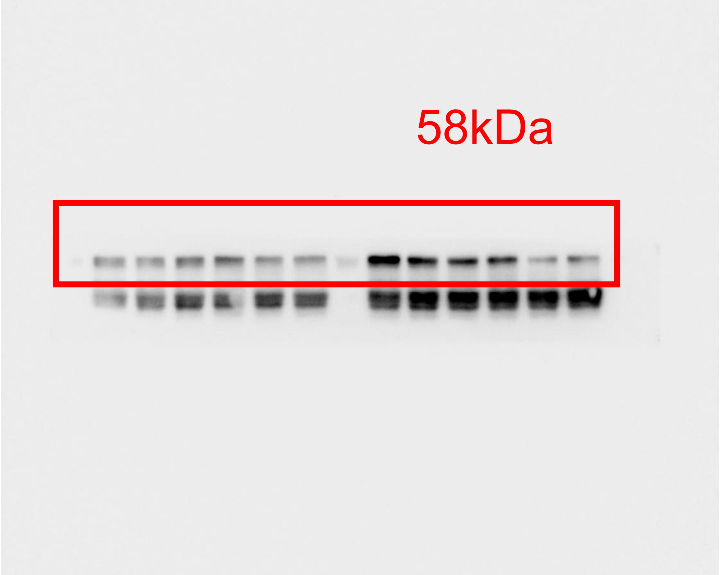


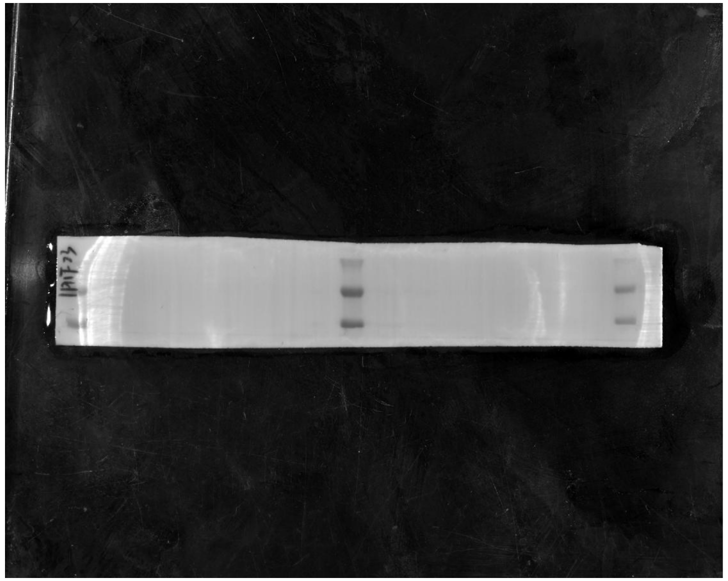

H1299ACTN4

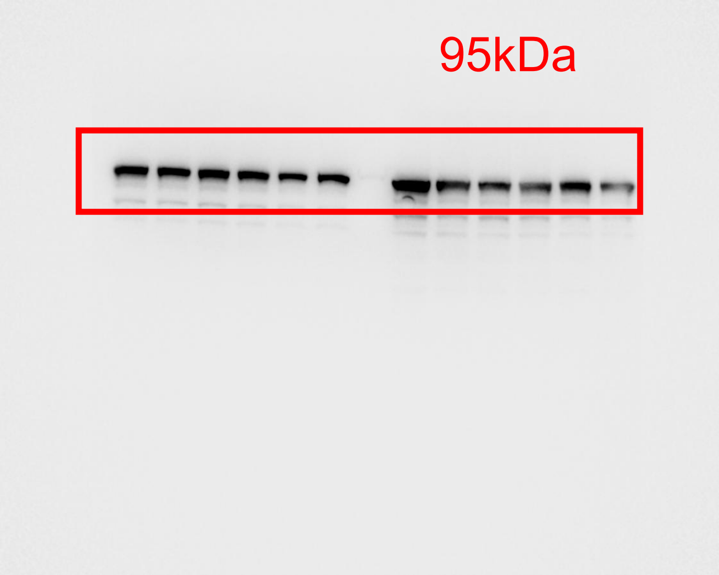


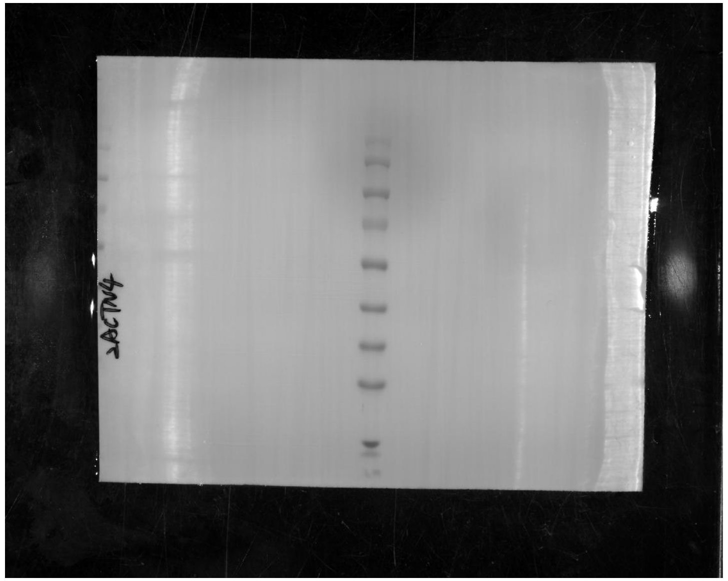

A549ACTN4

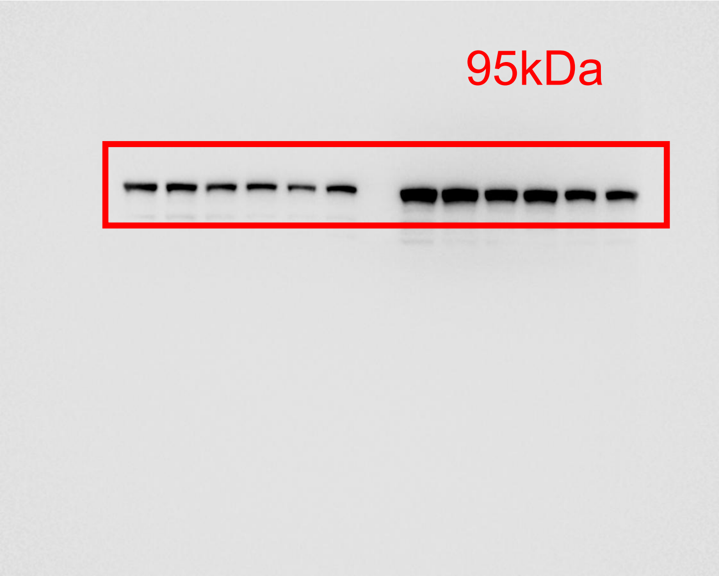


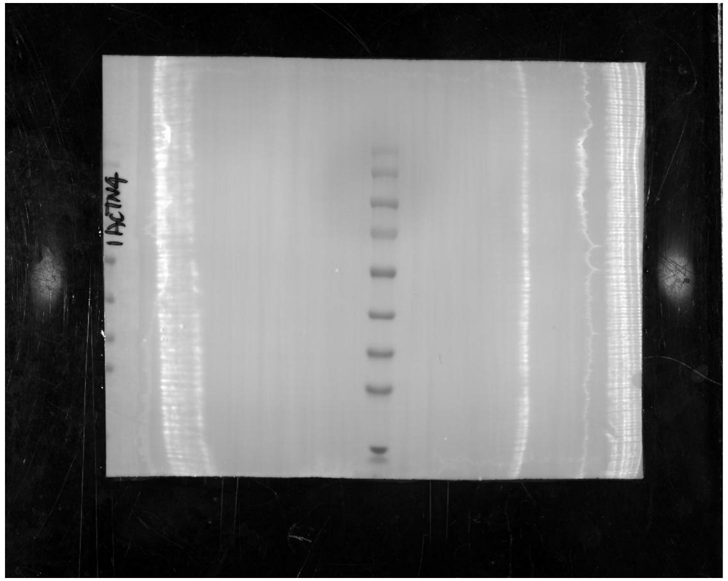

**H1299GAPDH**

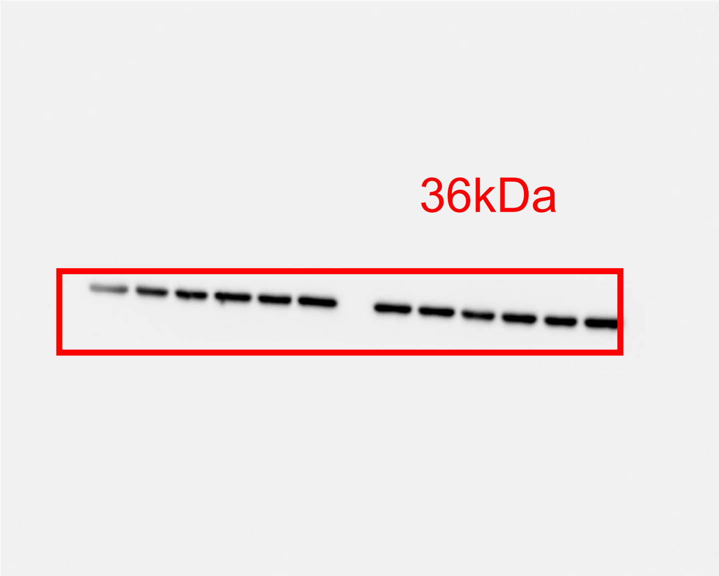


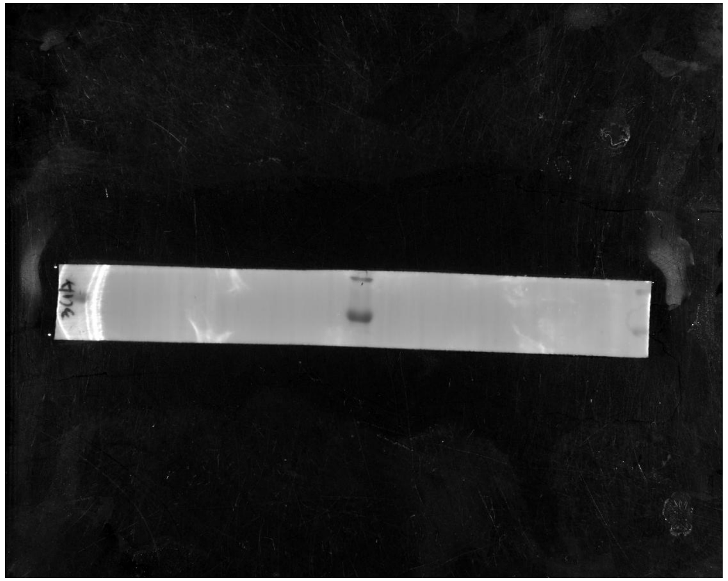

**A549GAPDH**

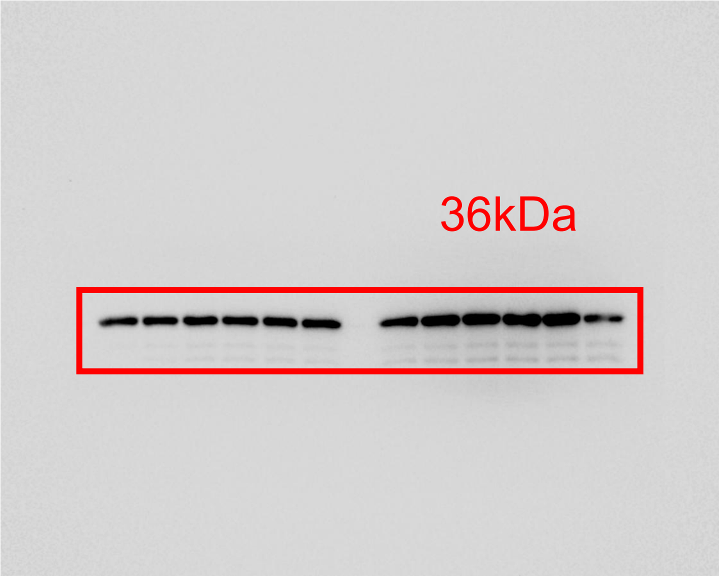


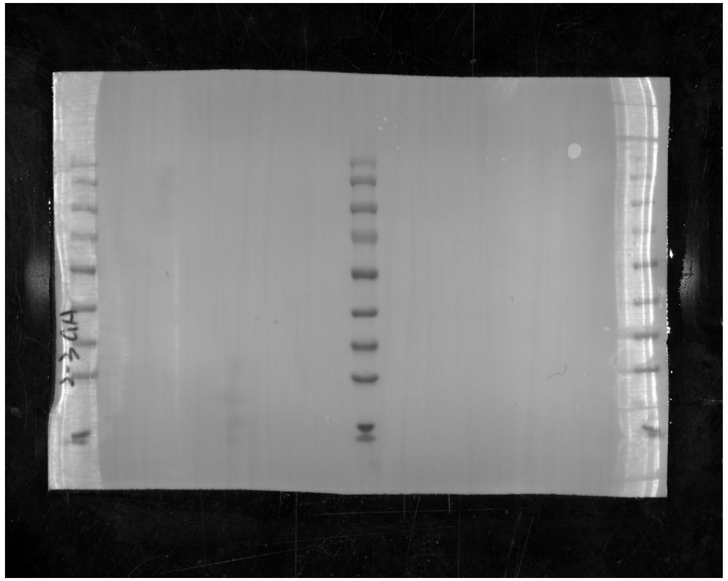

# Figure7B

**H1299PHF23**

**A549PHF23**

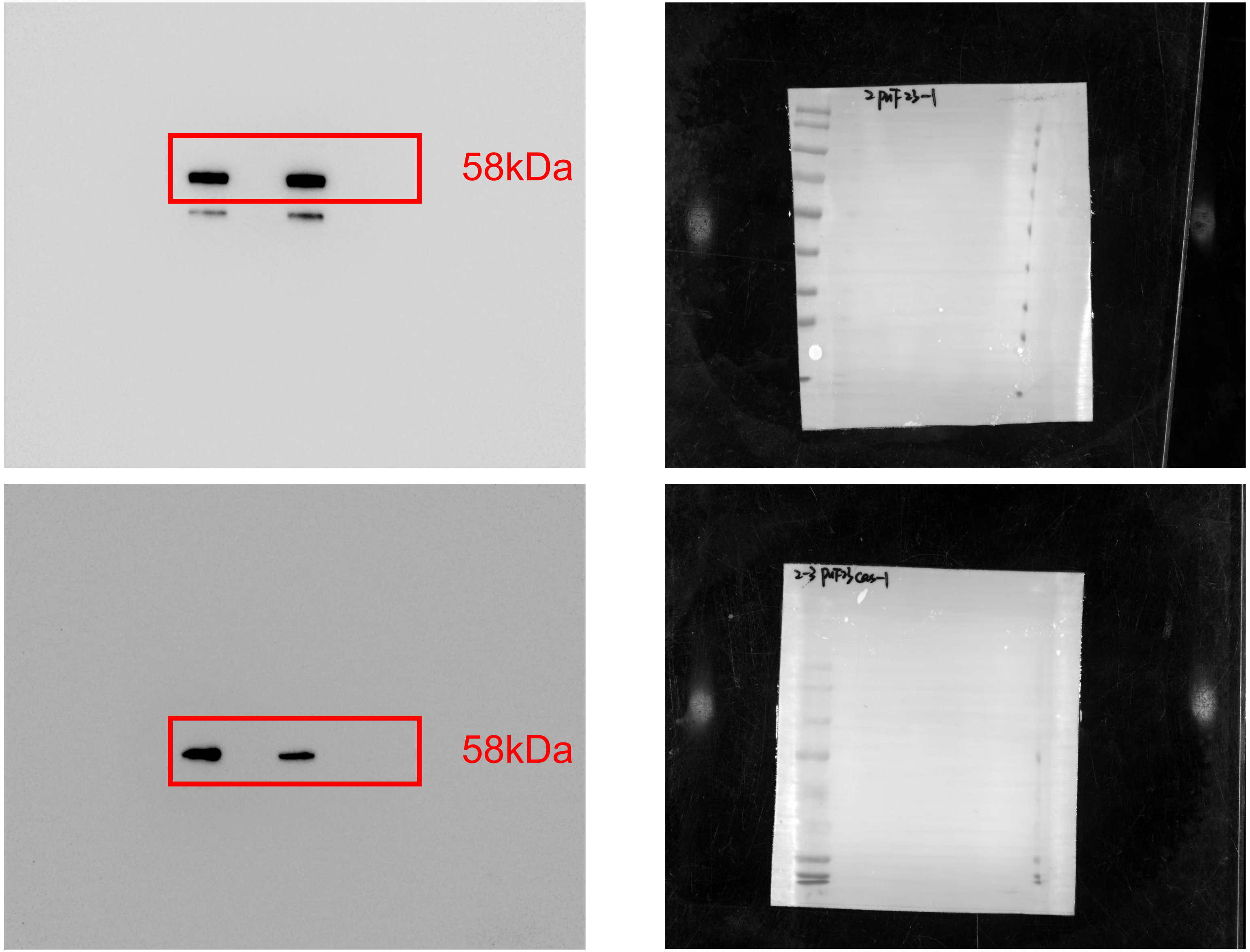

H1299ACTN4

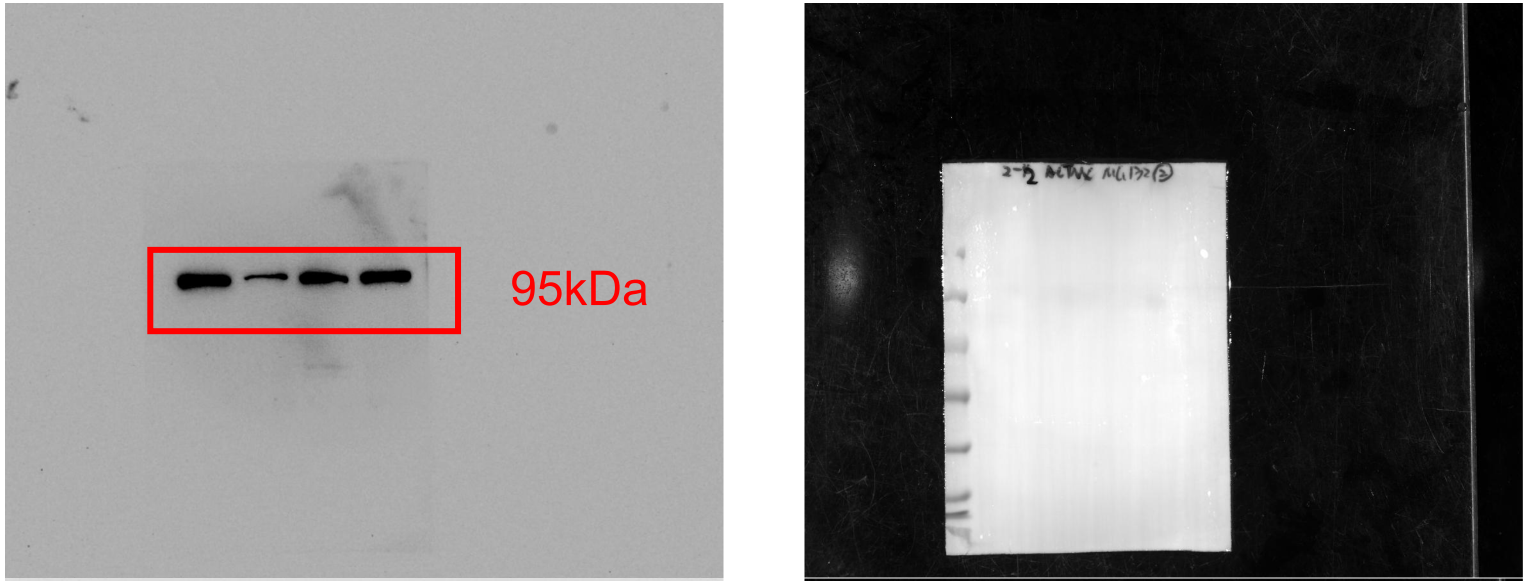


A549ACTN4

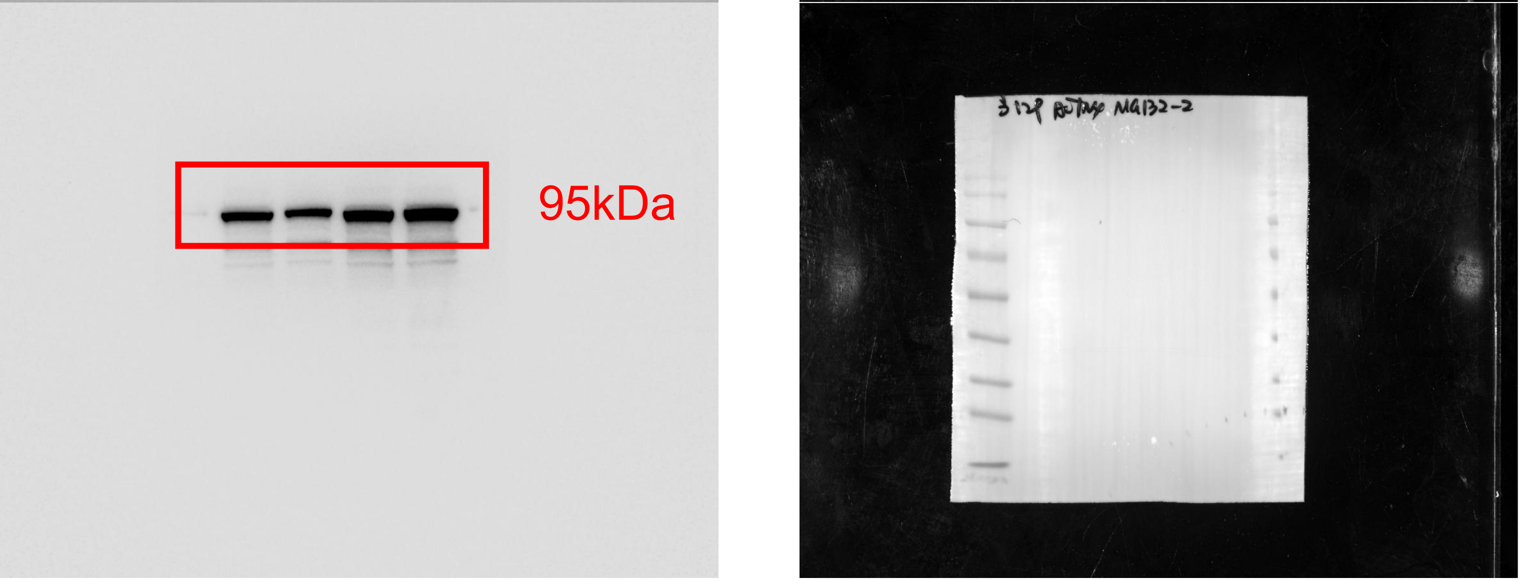

**H1299GAPDH**

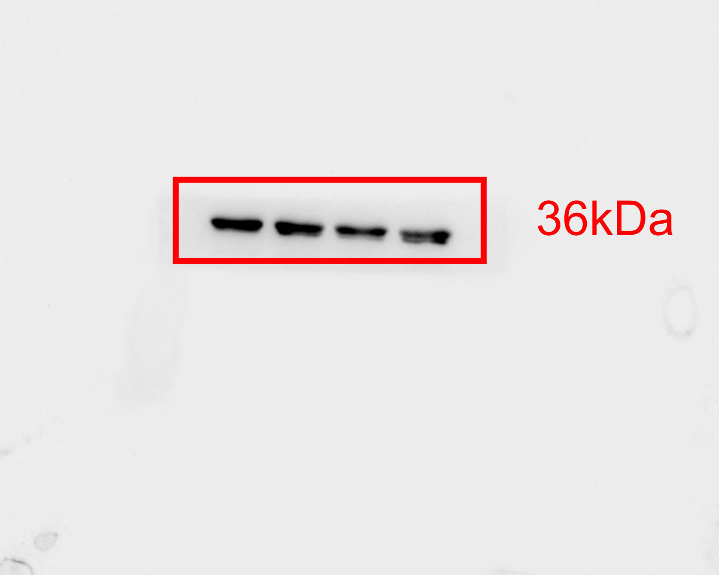


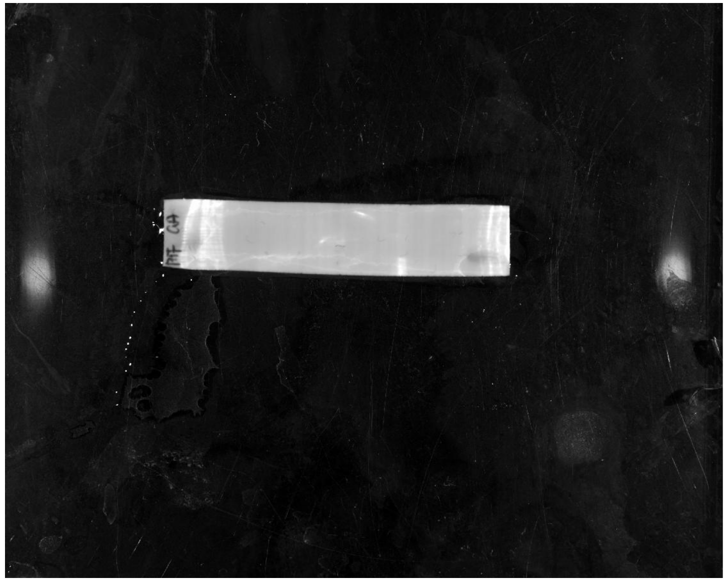

**A549GAPDH**

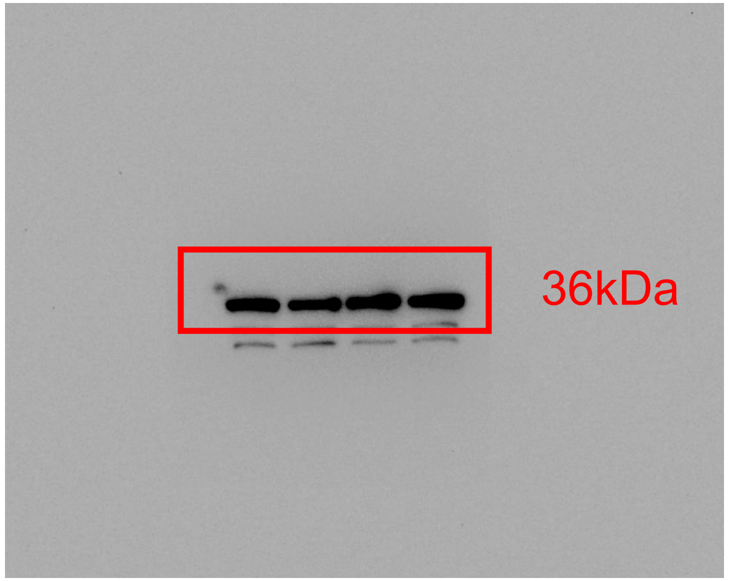


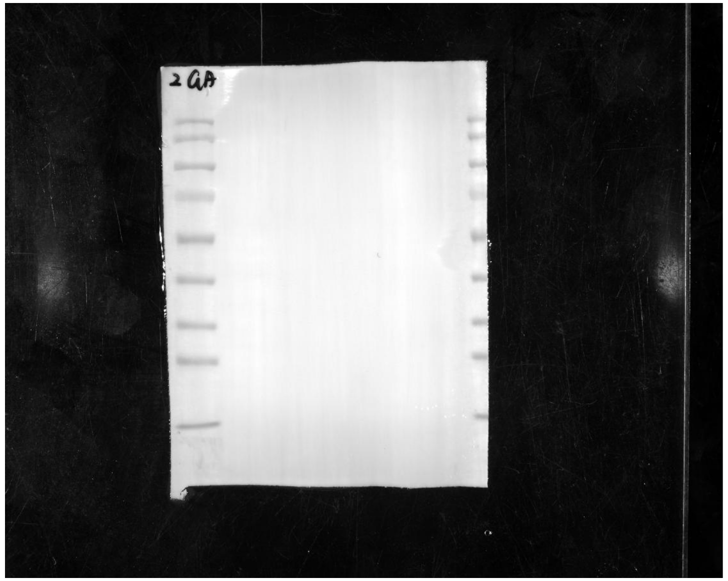

# Figure7C

H1299HA

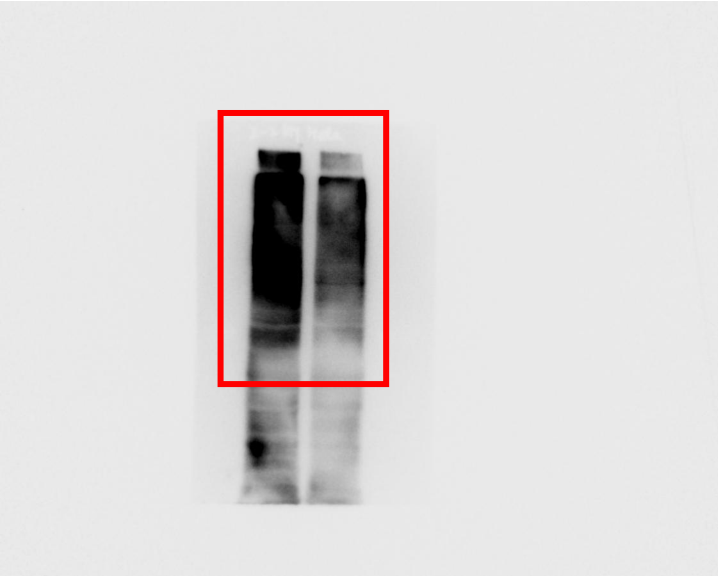

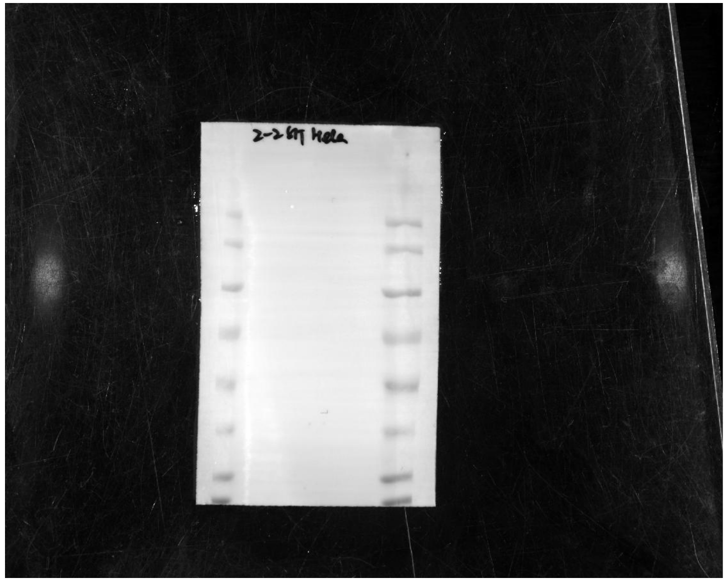

A549HA

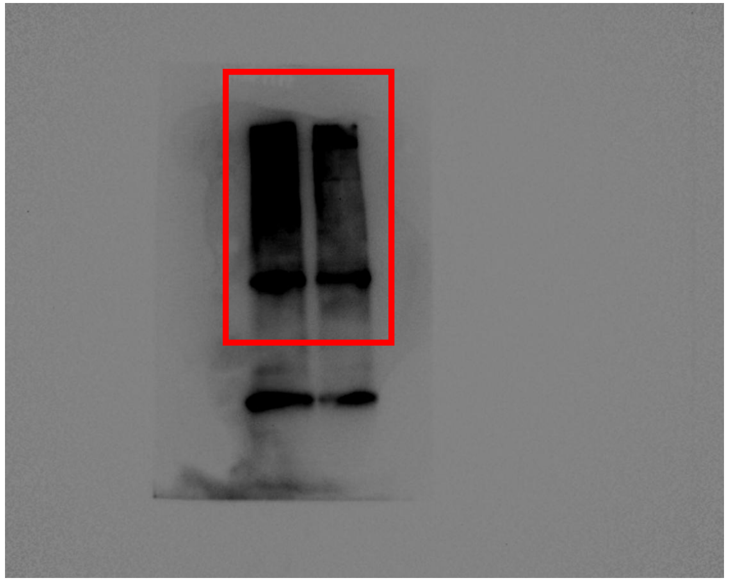

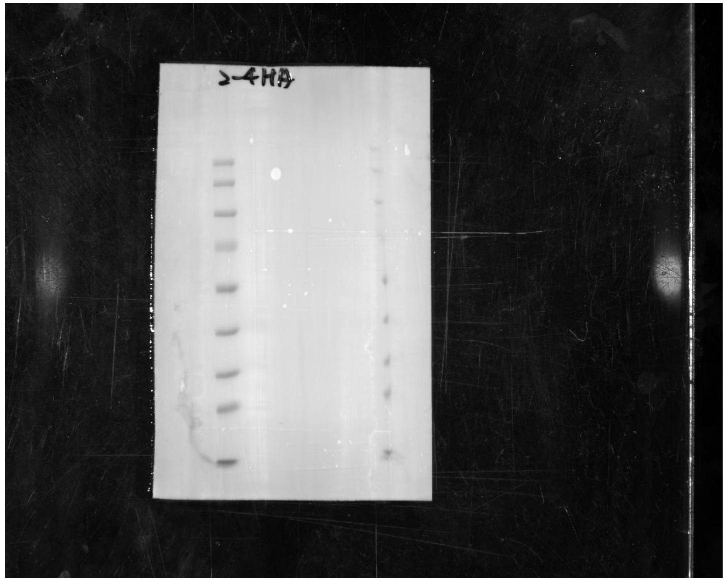

H1299ACTN4

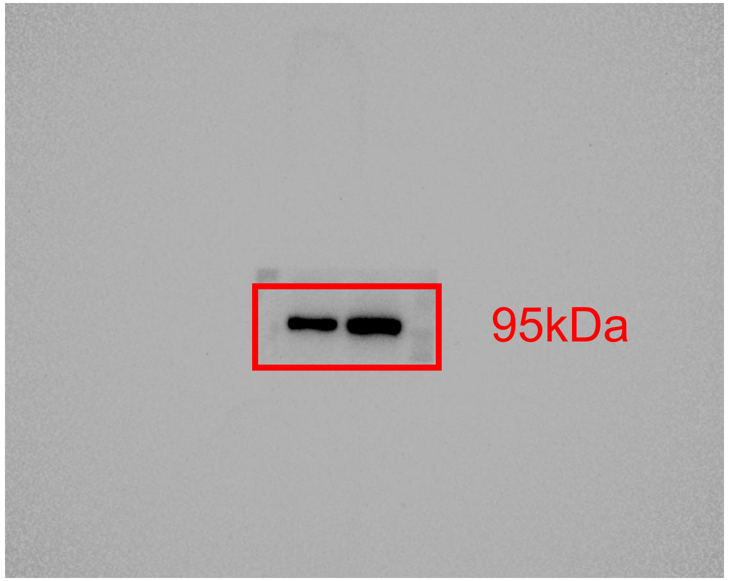


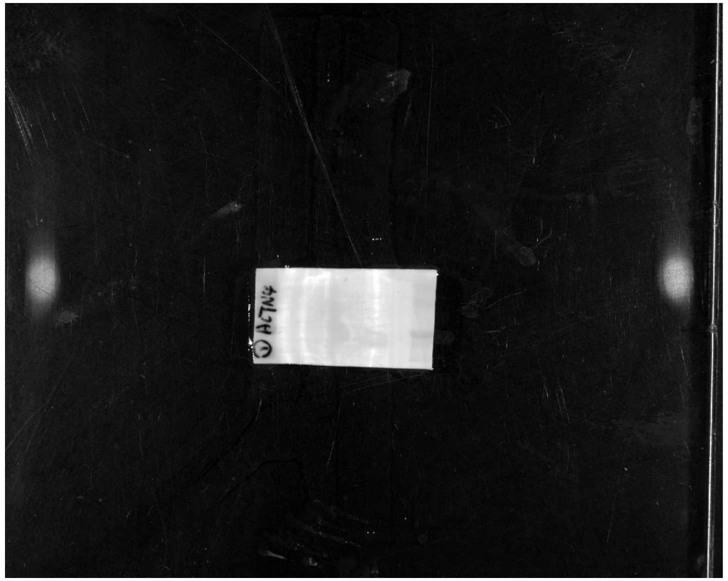

A549ACTN4

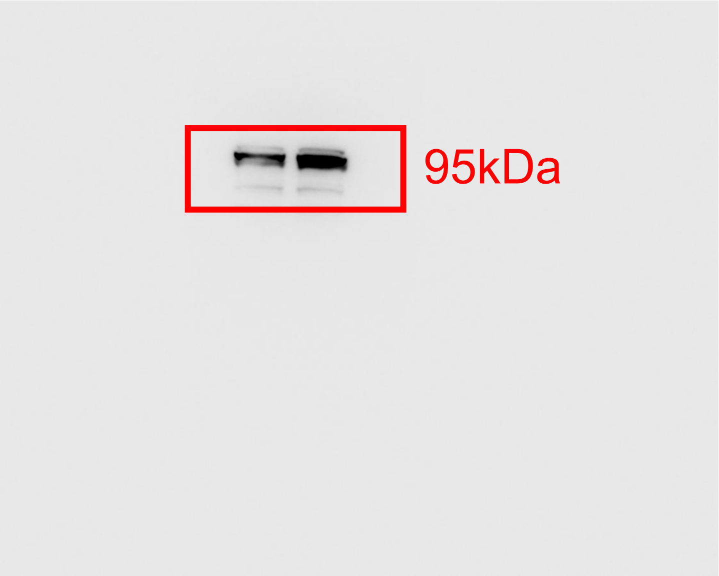


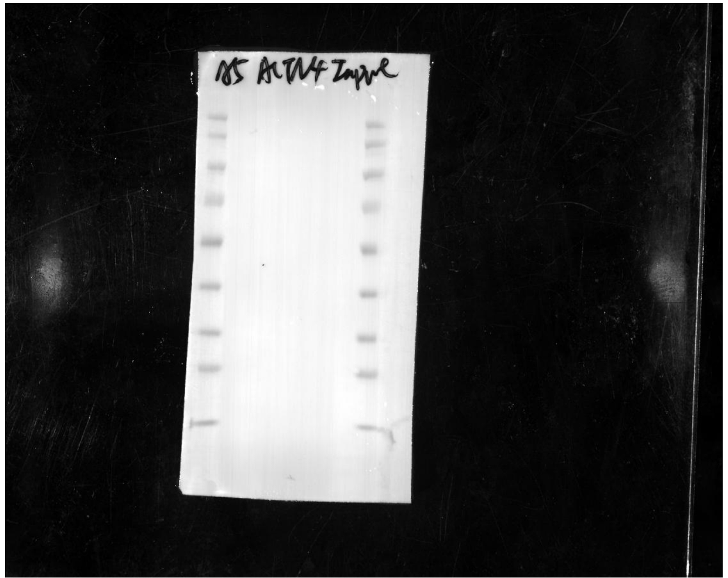

MYC

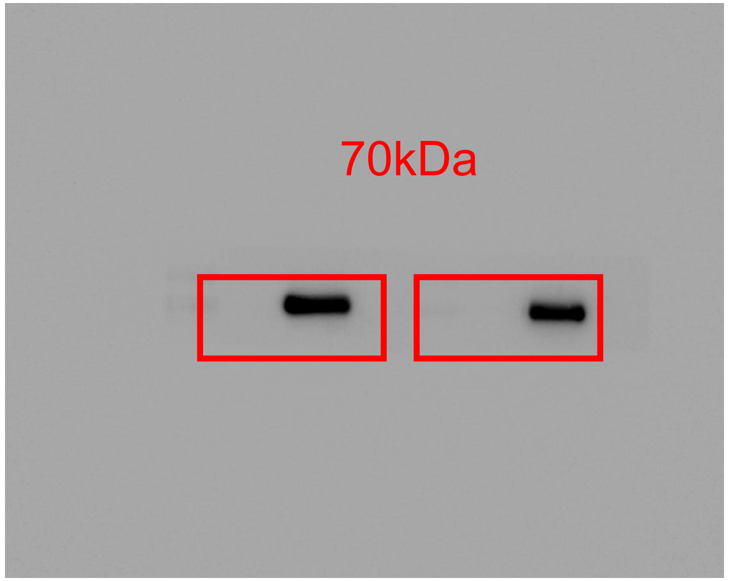


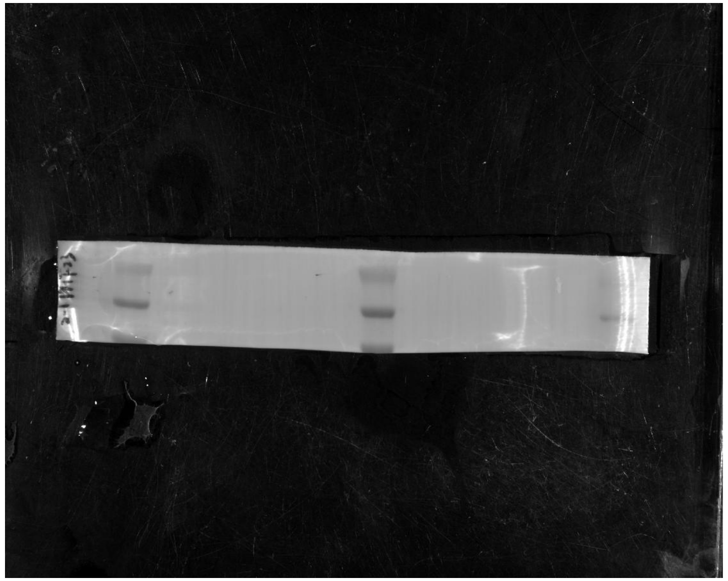

H1299GAPDH

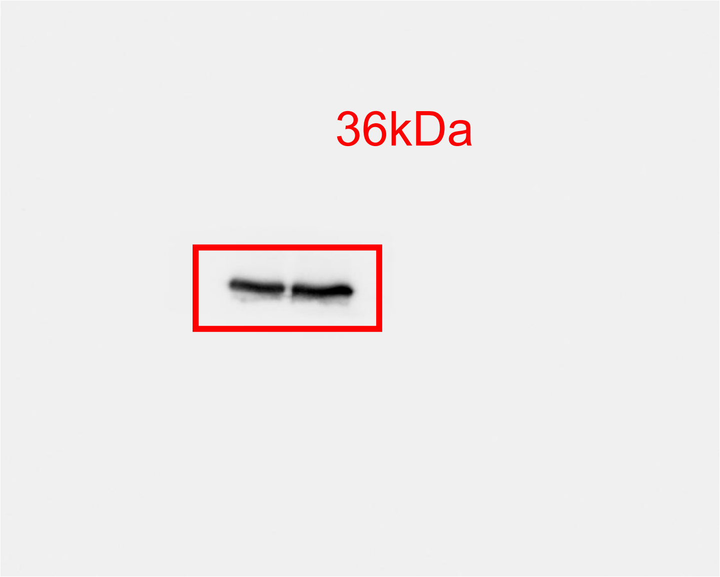


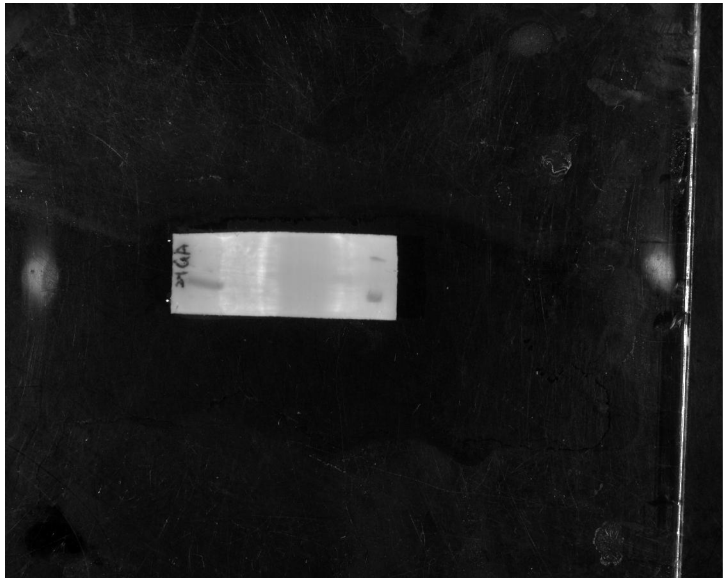

**A549GAPDH**

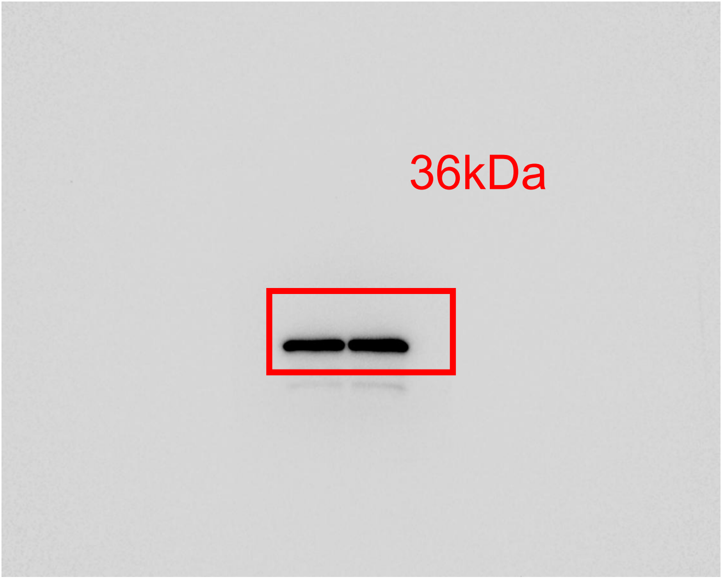


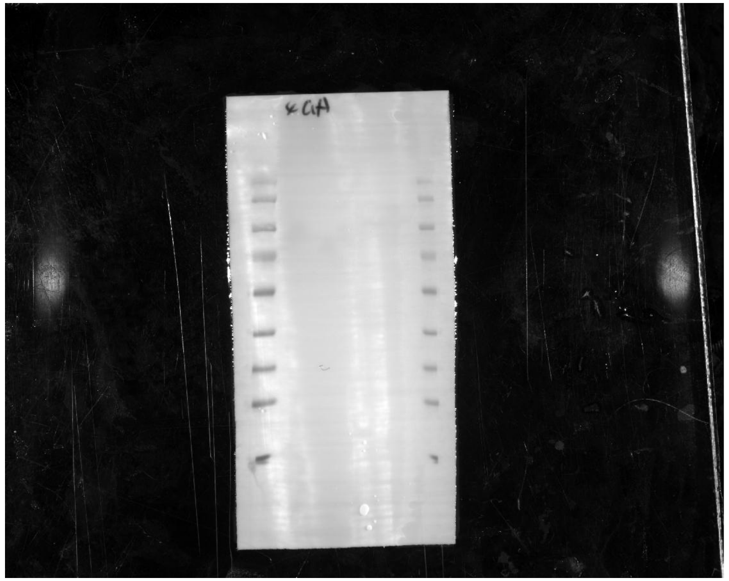

# Figure7D

H1299HA

A549HA

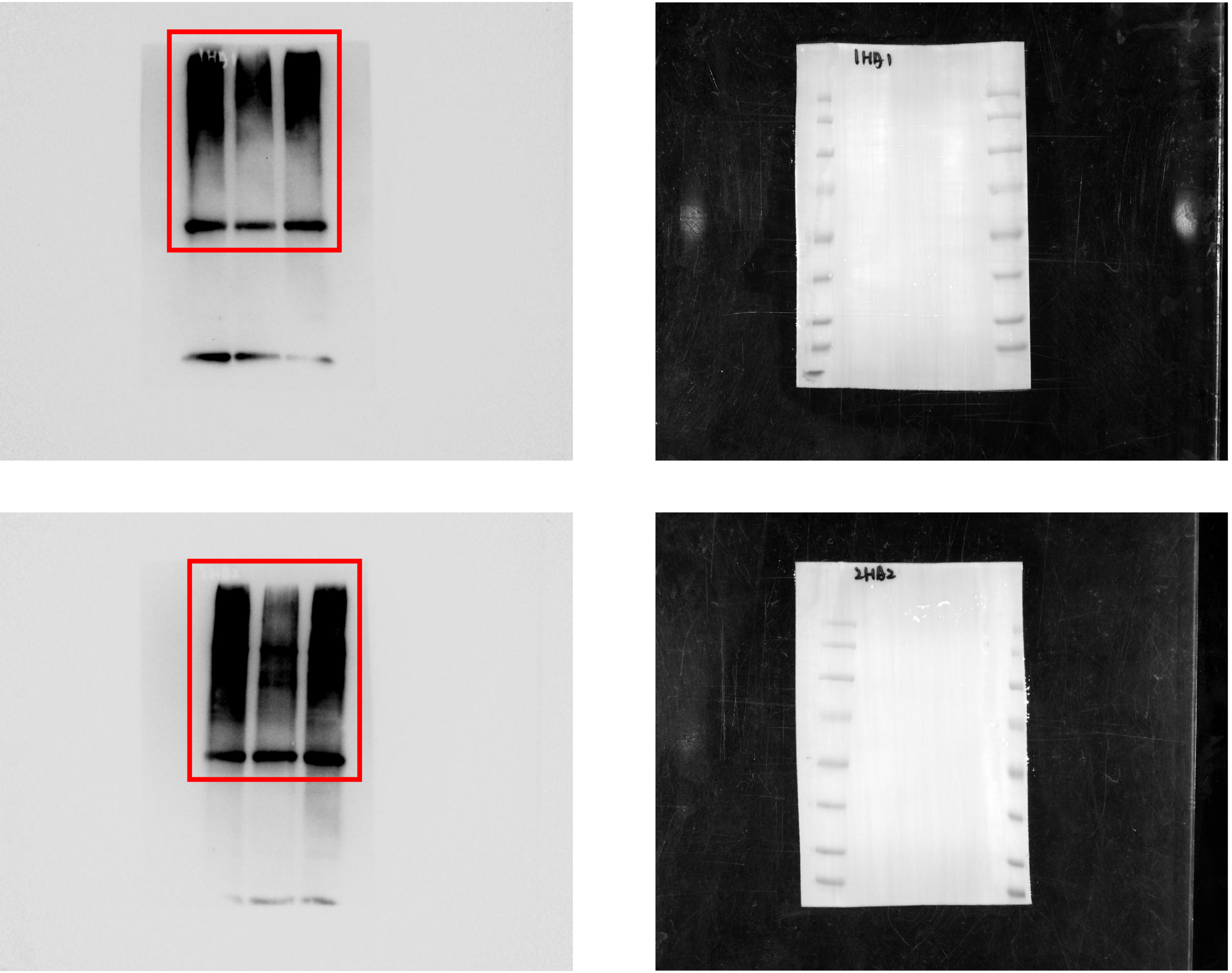

H1299ACTN4

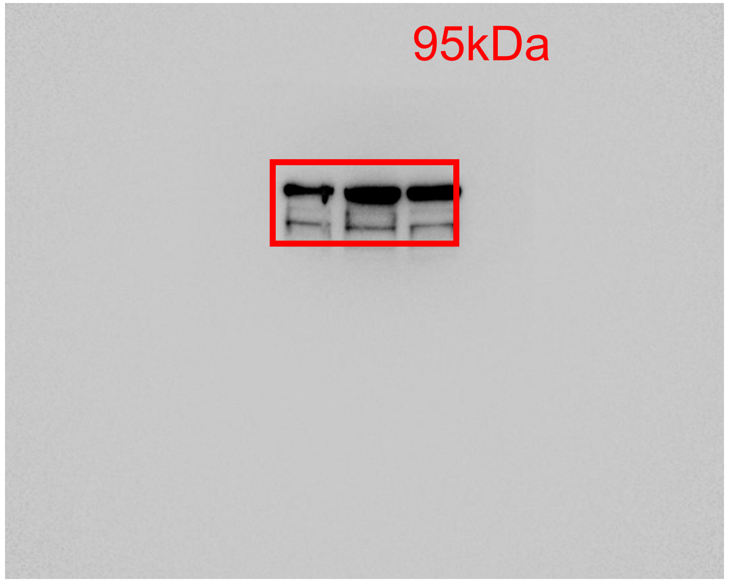


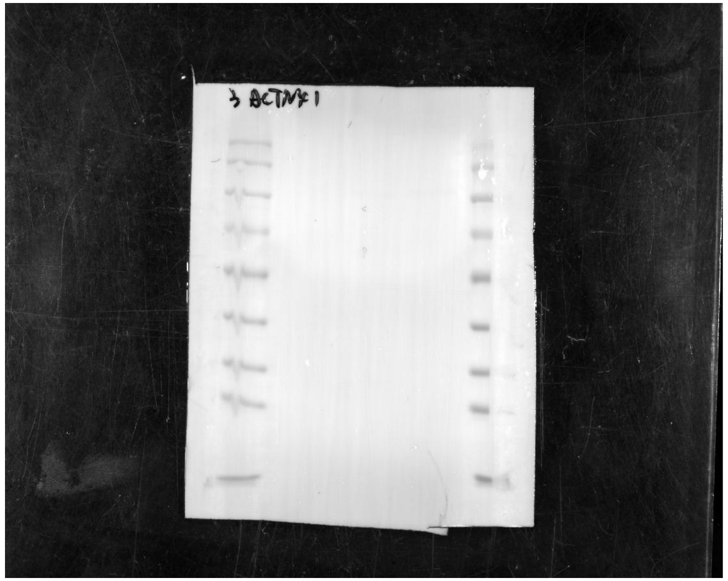

A549ACTN4

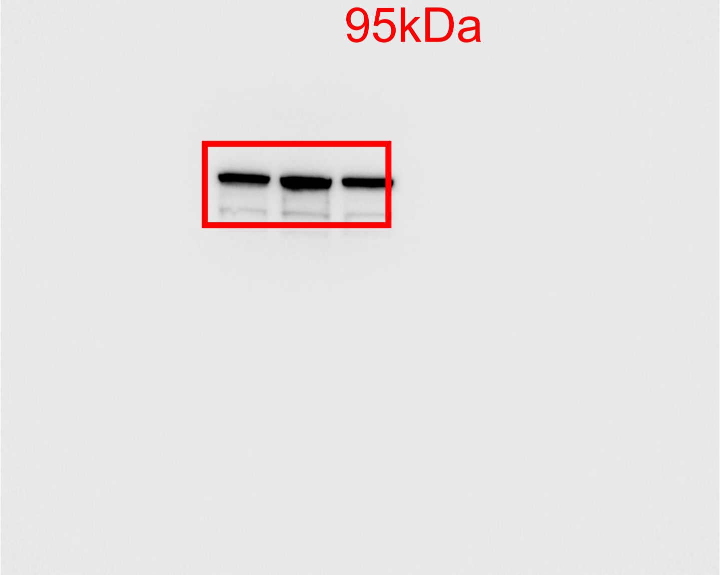


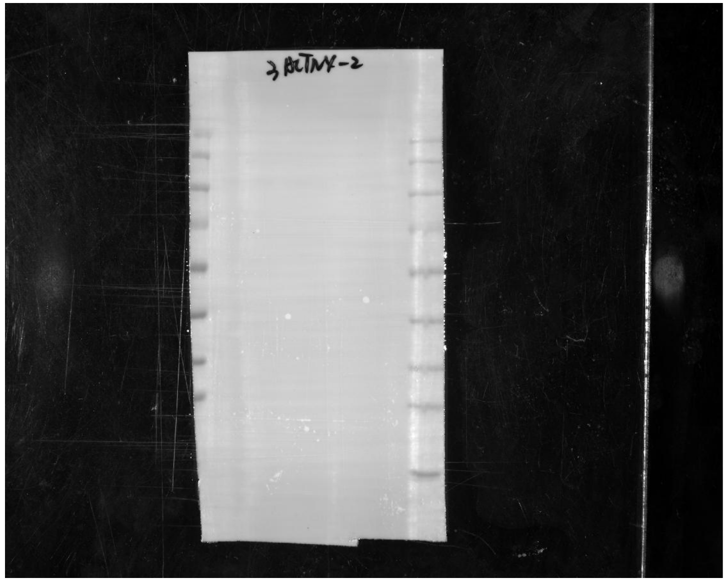

**MYC**

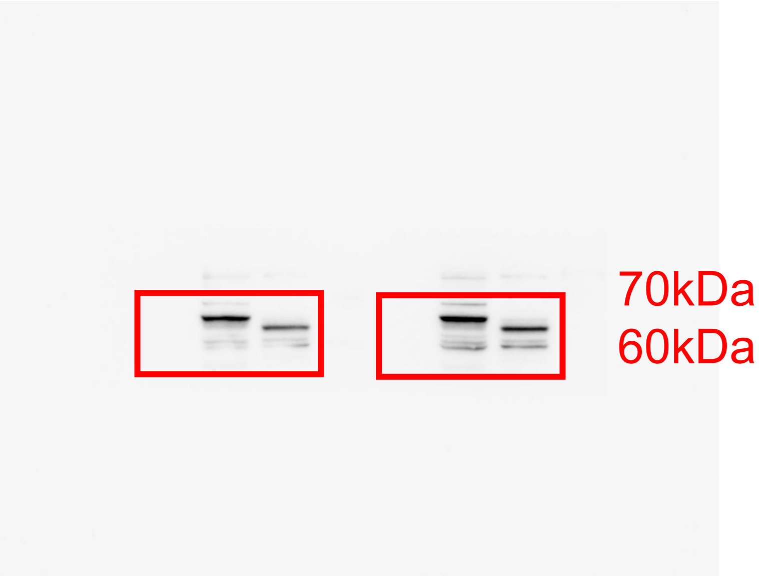


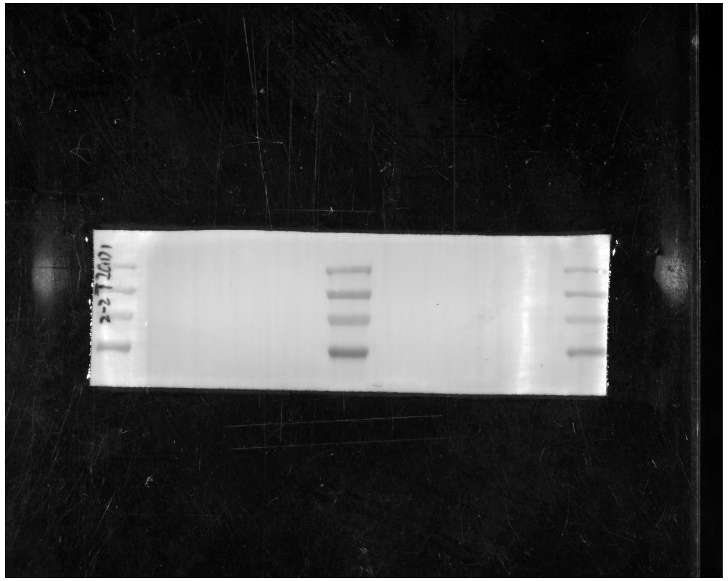

**A549-GAPDH**

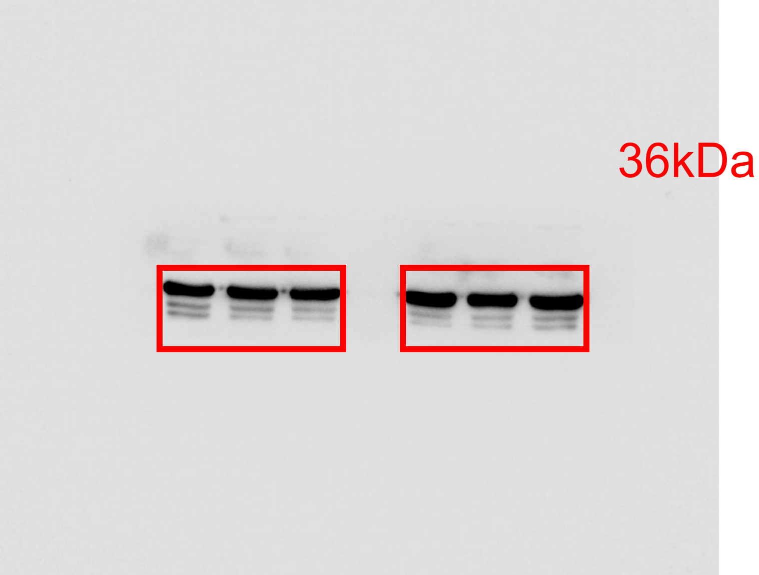


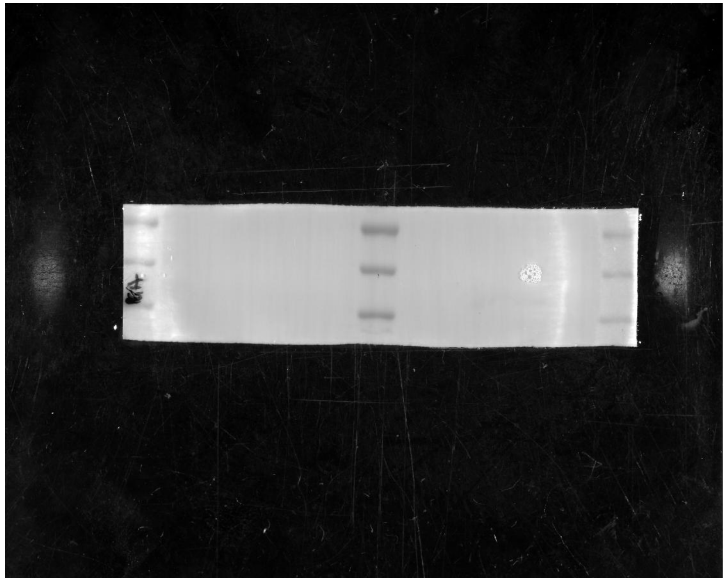

FIG7-E

H1299 PHF23

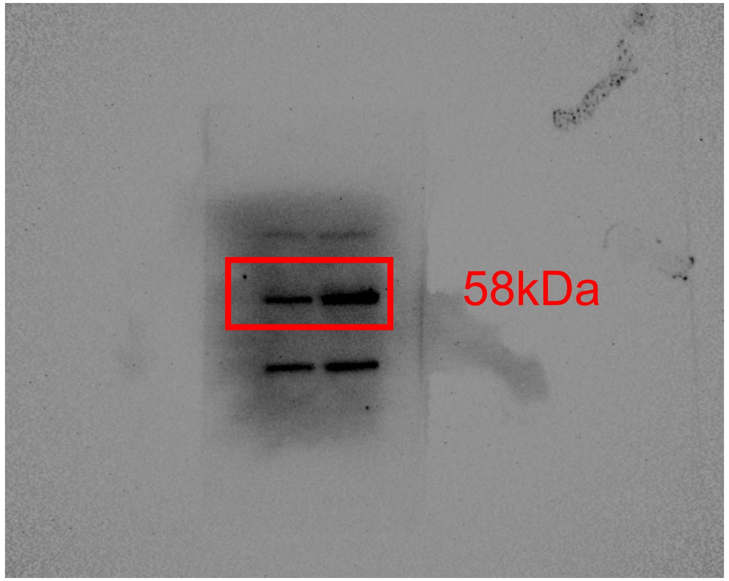


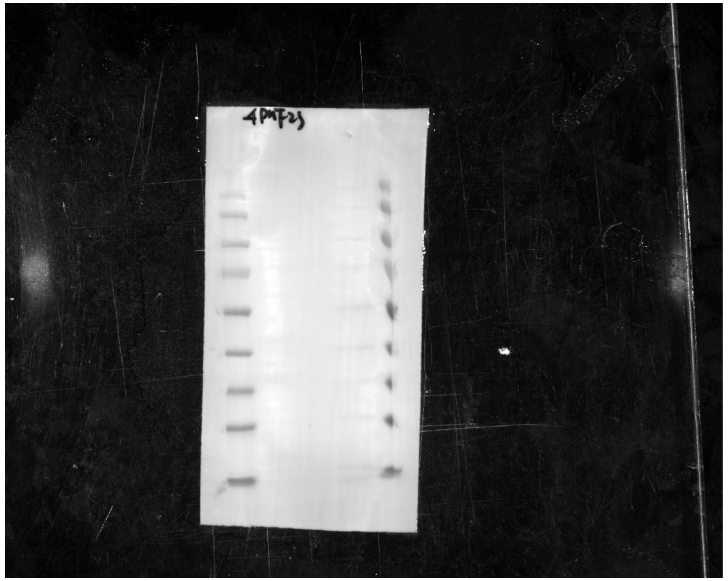

A549 PHF23

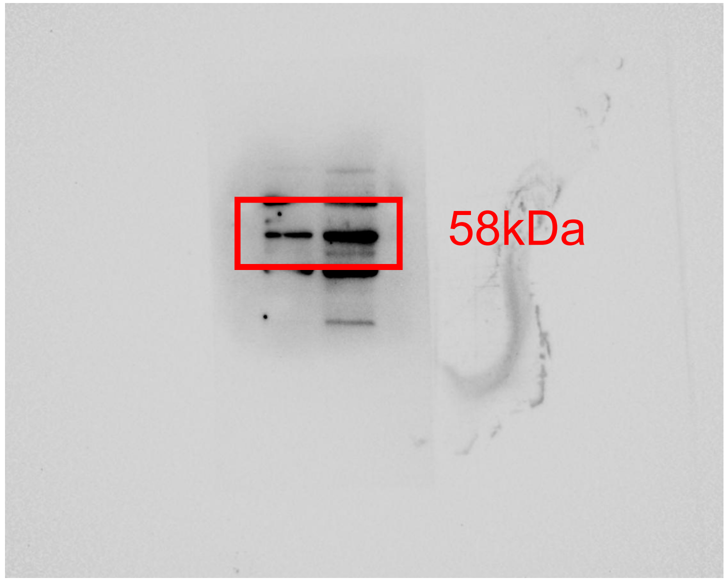


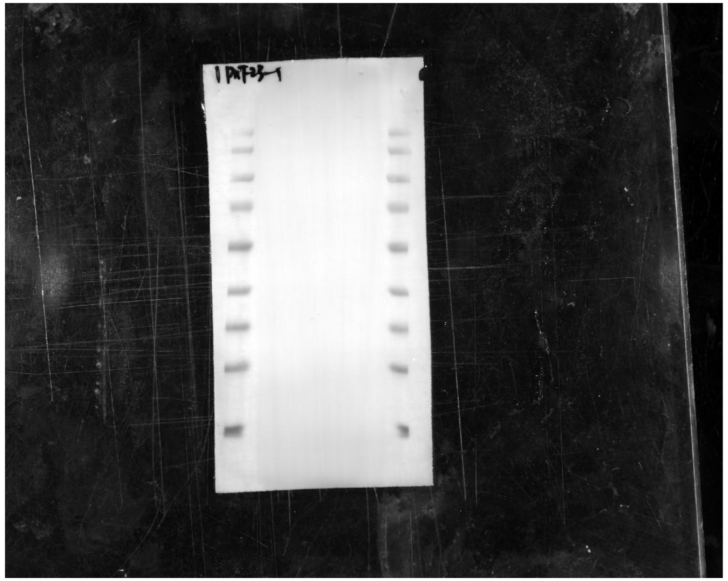

H1299 RNF38

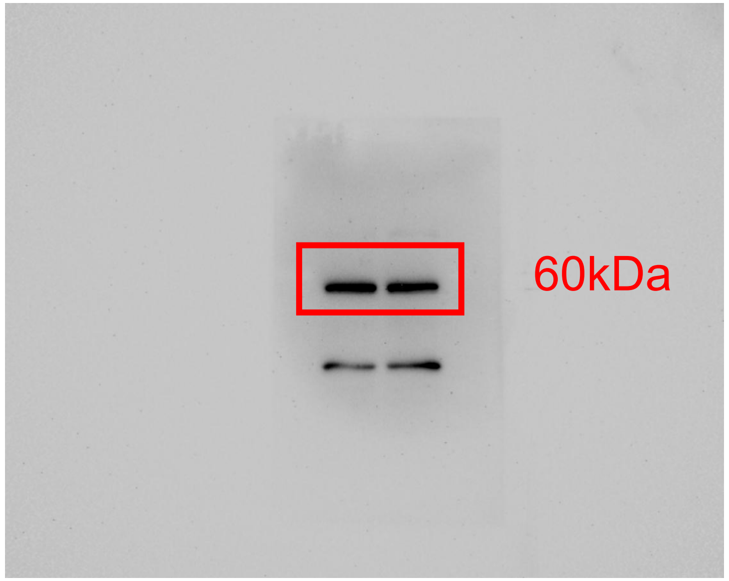

60kDa

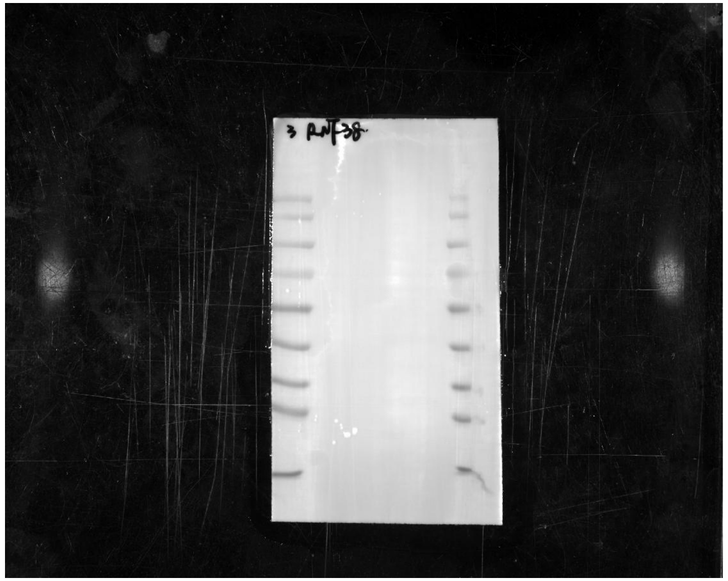

A549 RNF38

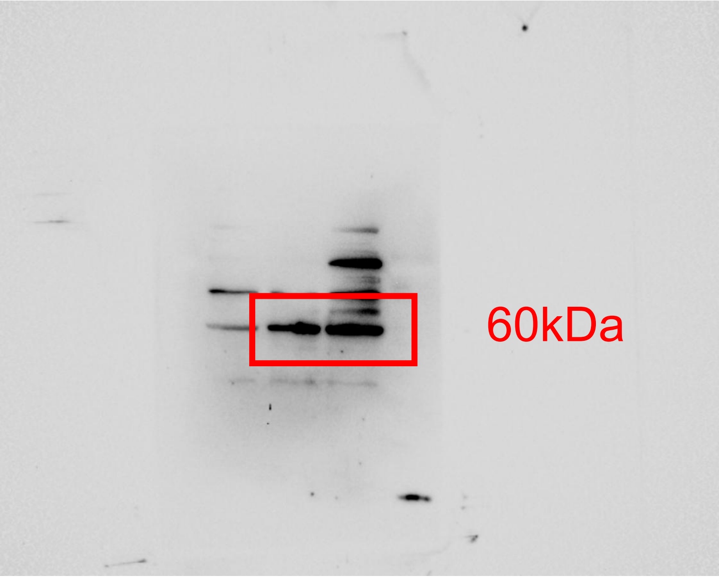

60kDa

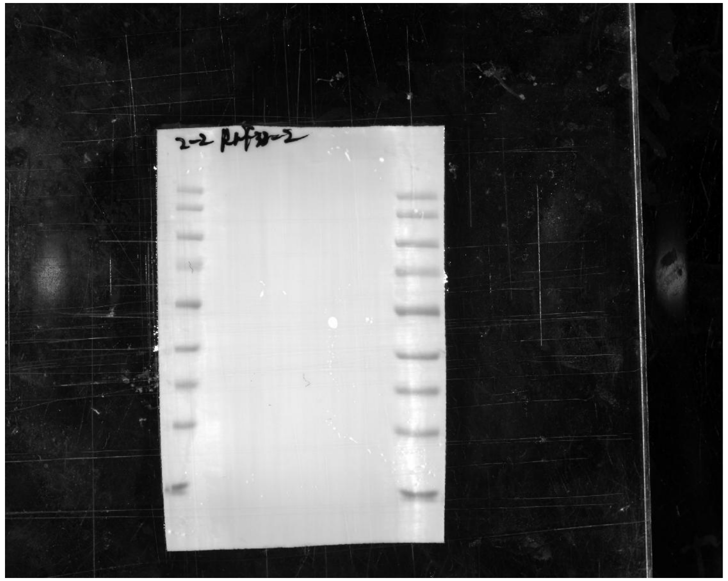

H1299 GAPDH

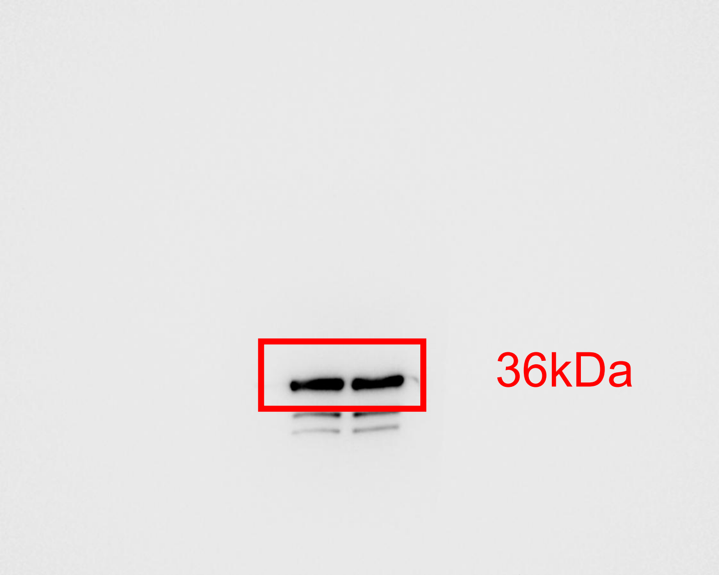


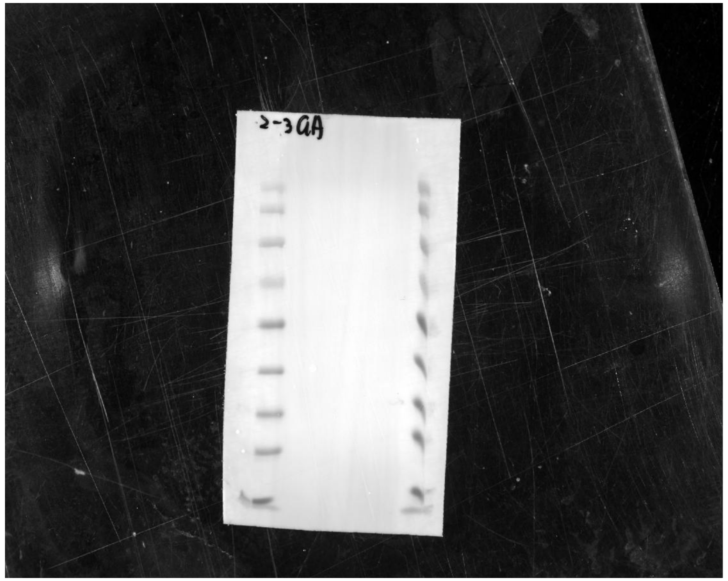

A549 GAPDH

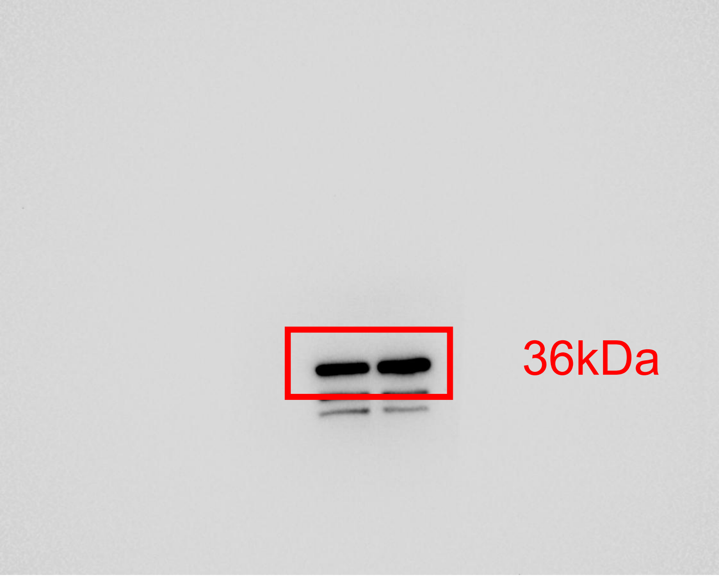


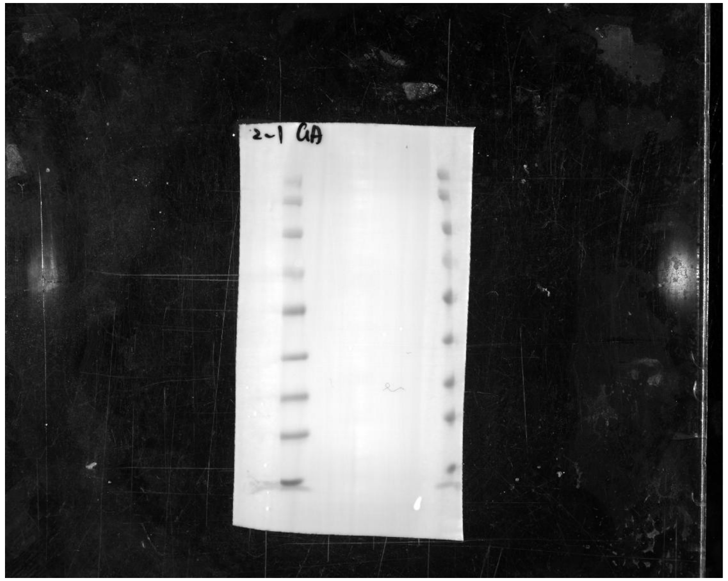

# FIG7-F

H1299 ACTN4

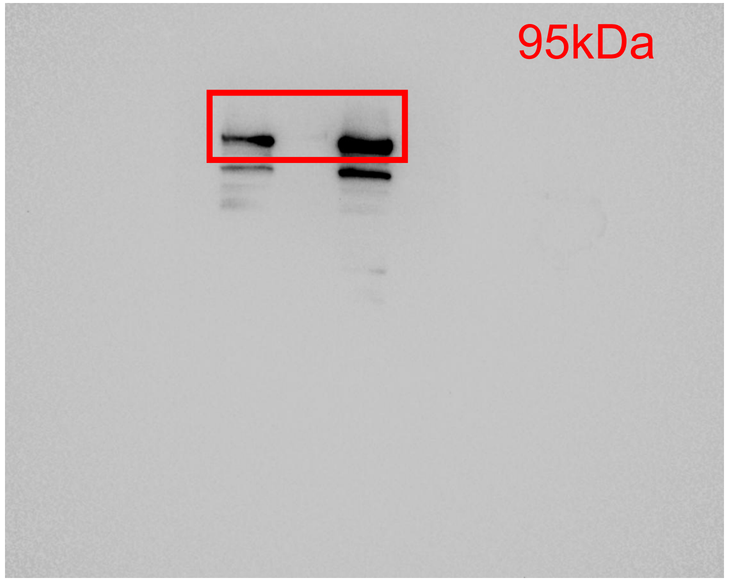


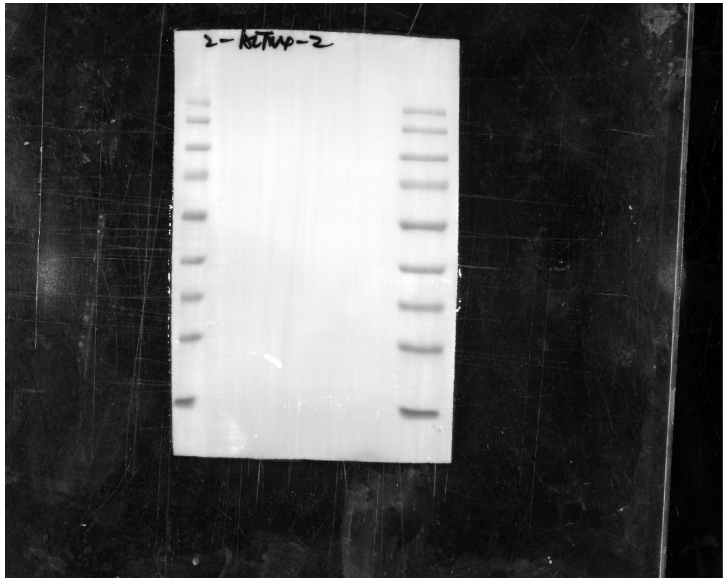

A549 ACTN4

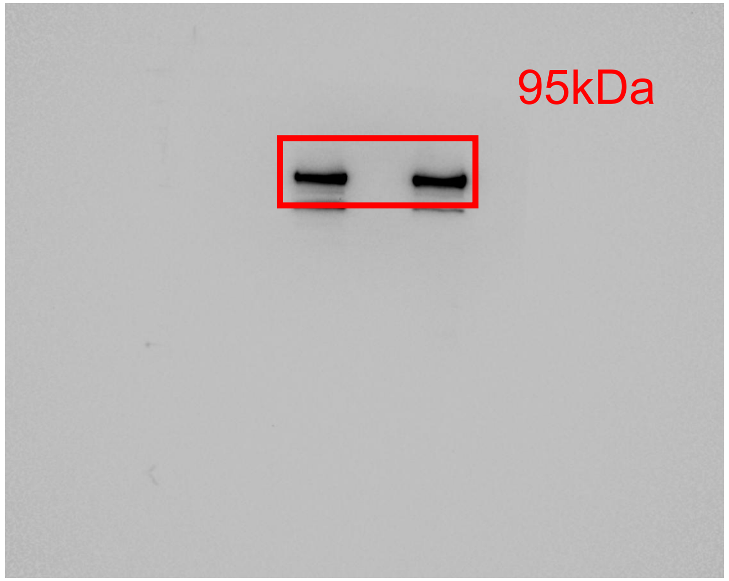


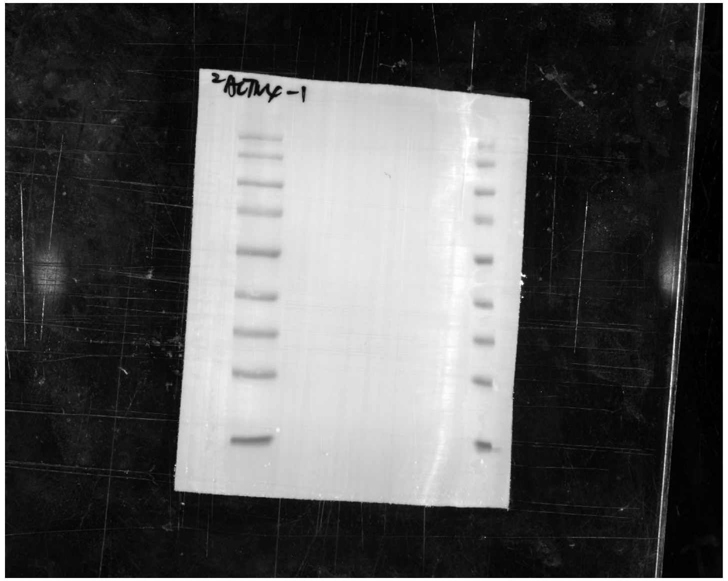

H1299 RNF38

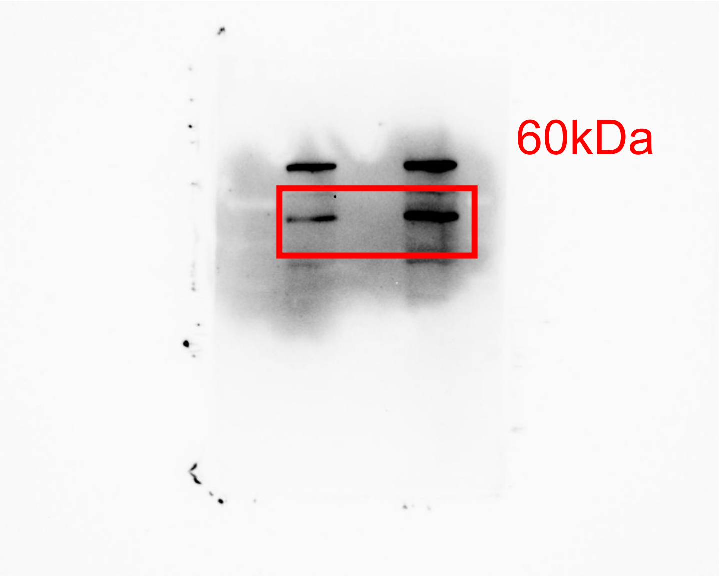


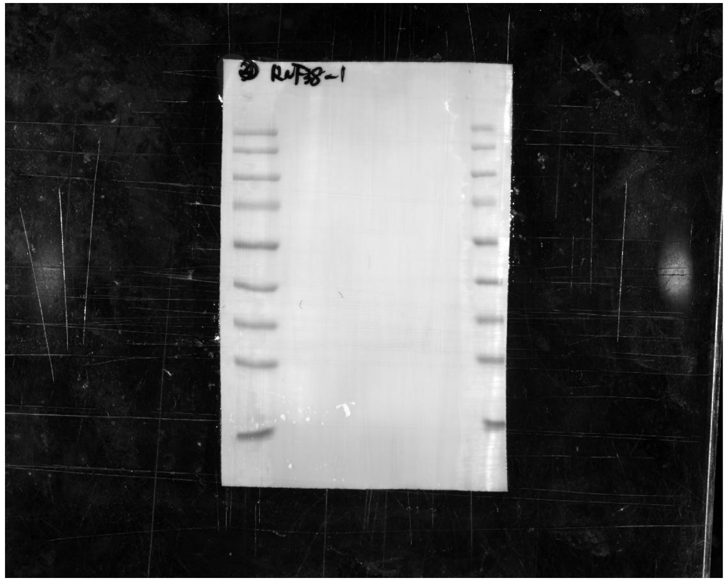

A549 RNF38

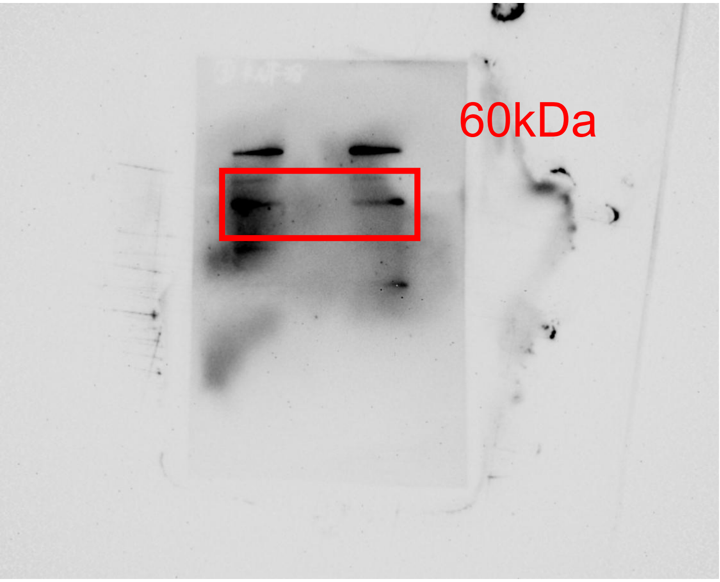


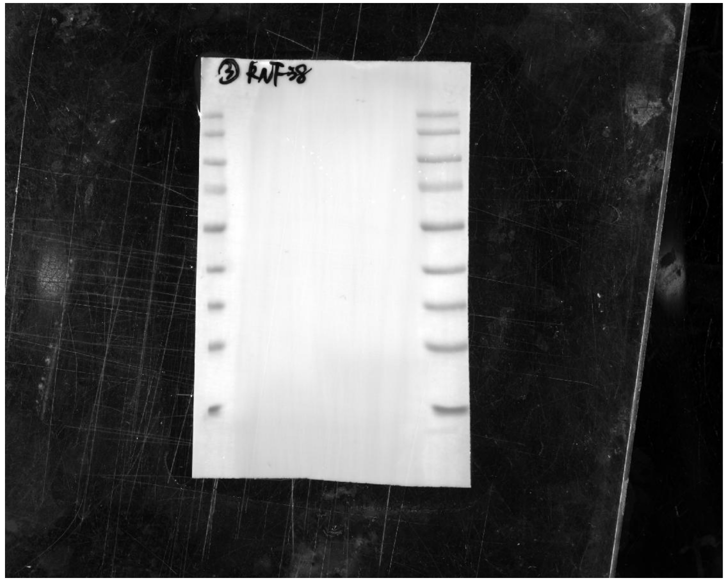

H1299 PHF23

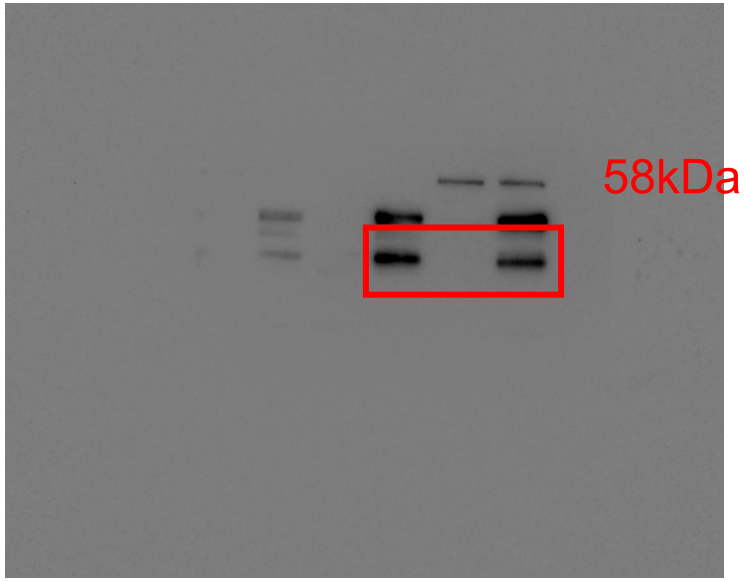


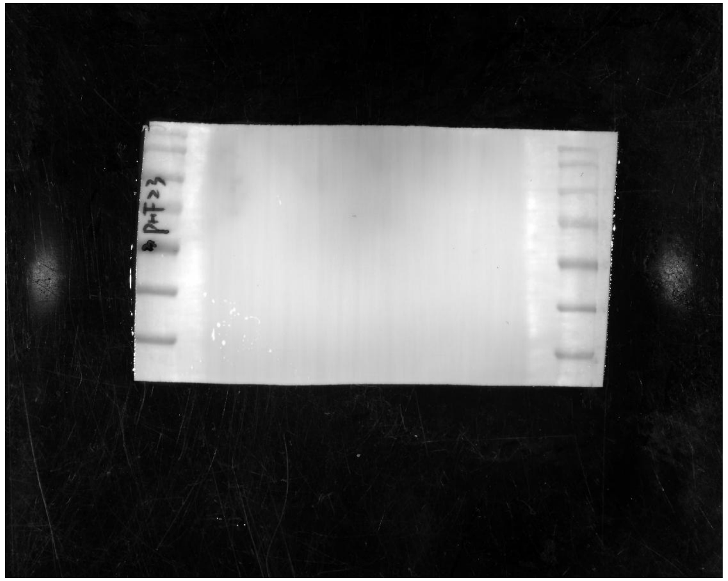

A549 PHF23

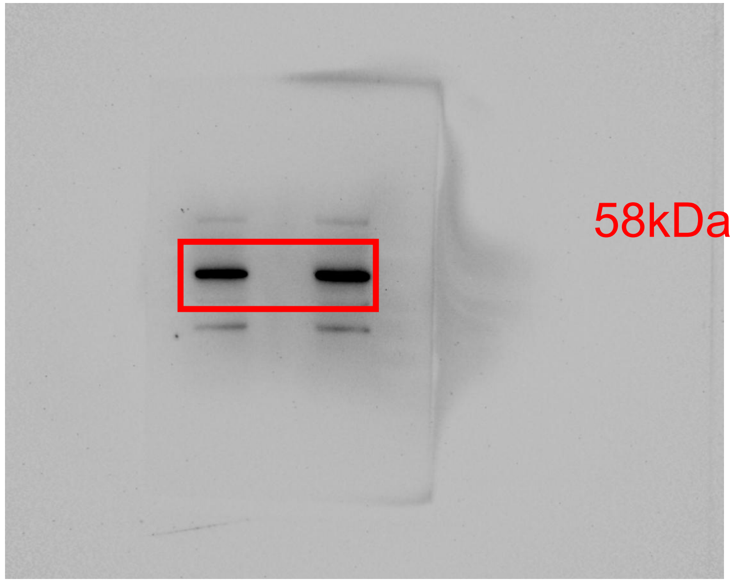


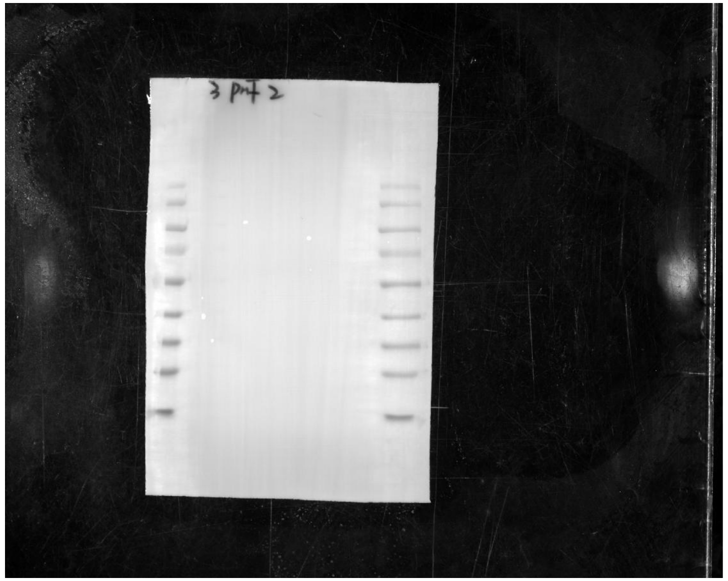

FIG7-G

H1299GFP-ACTN4

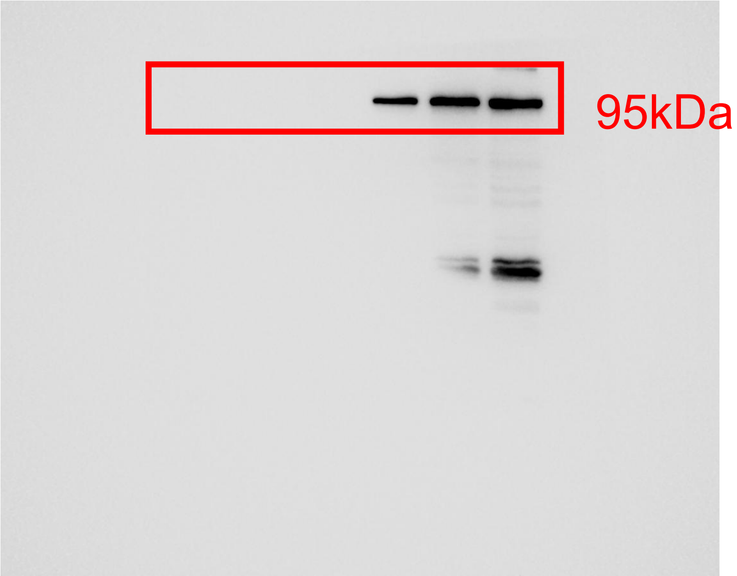


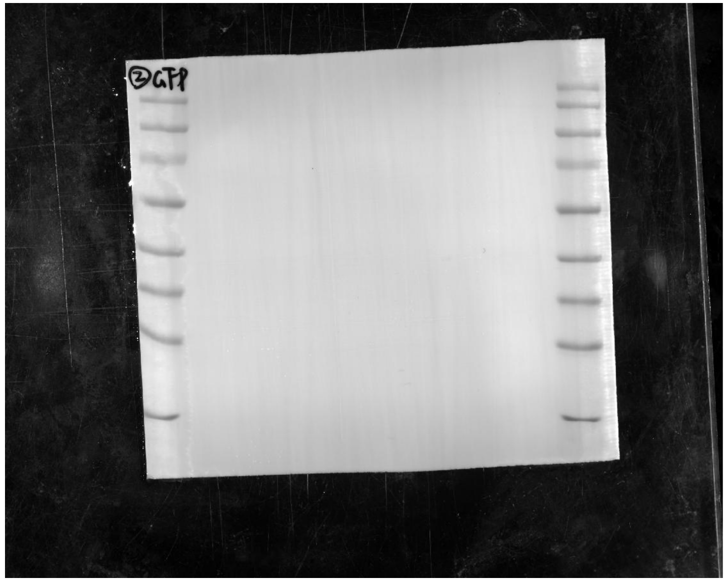


A549GFP-ACTN4

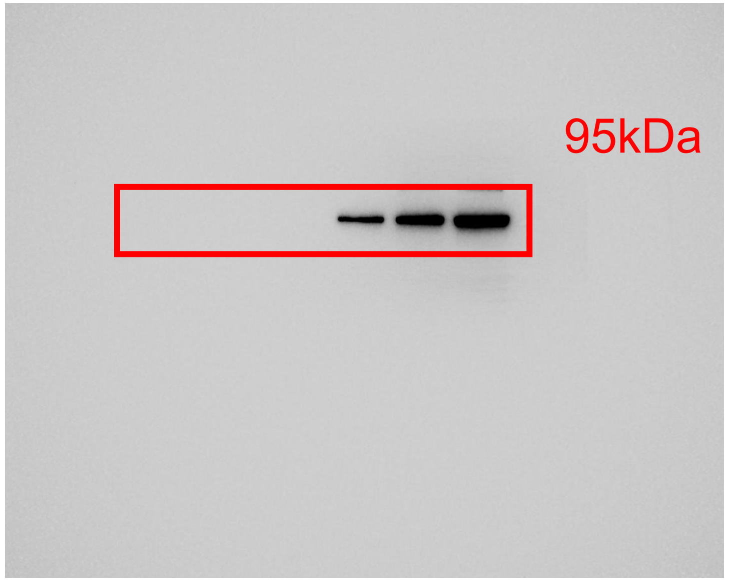


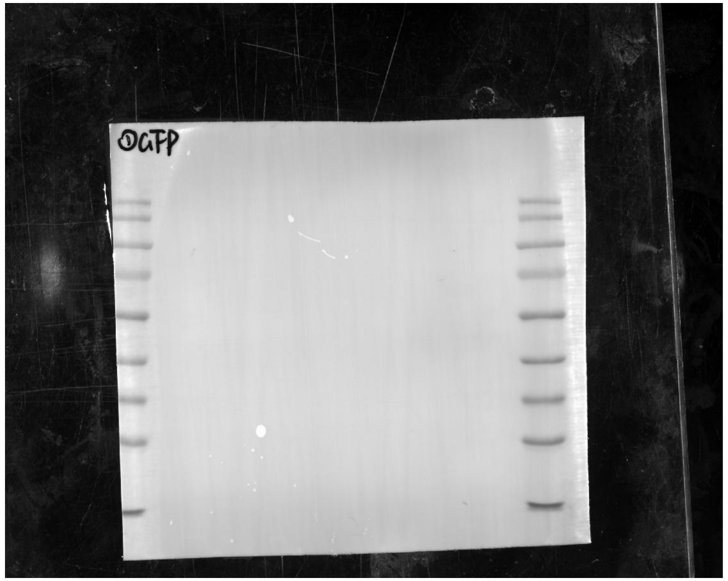

H1299RNF38

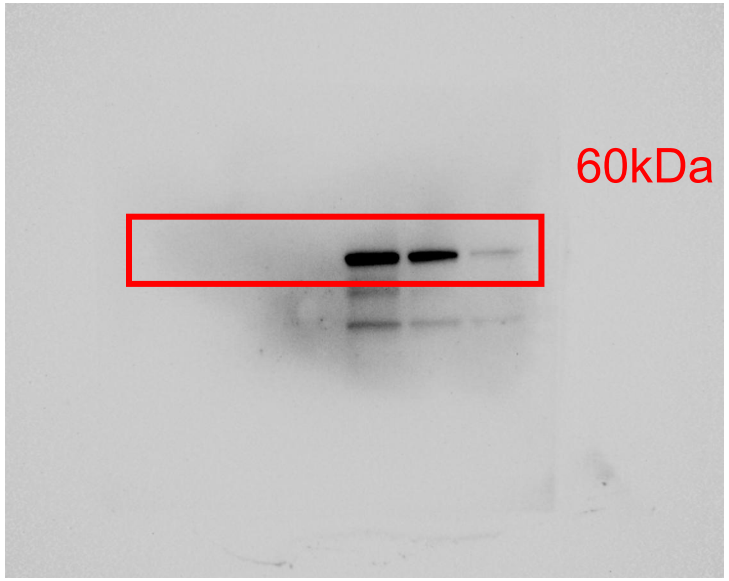


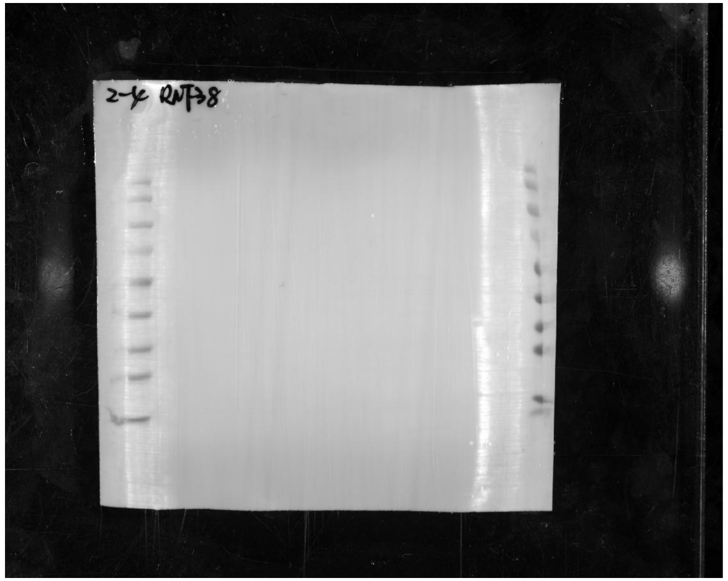

A549RNF38

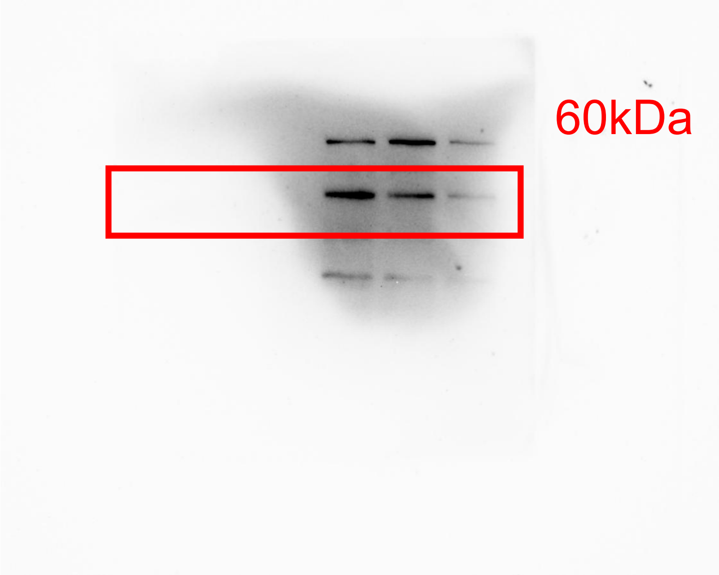


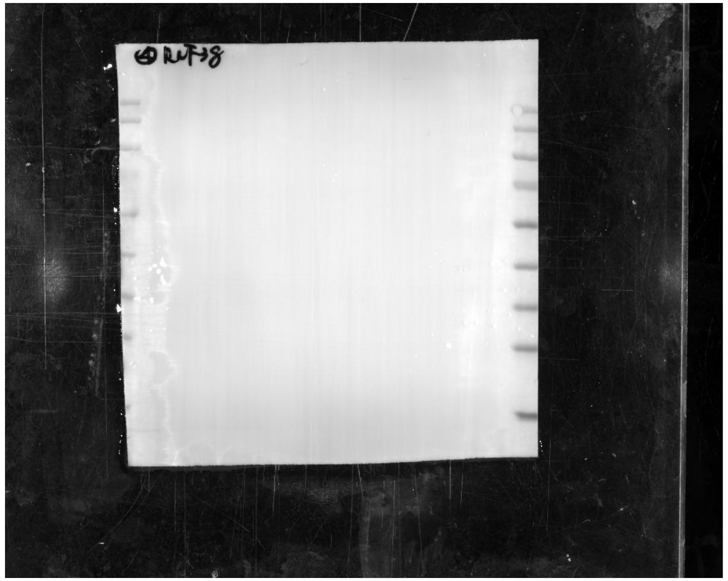

INPUTH1299RNF38

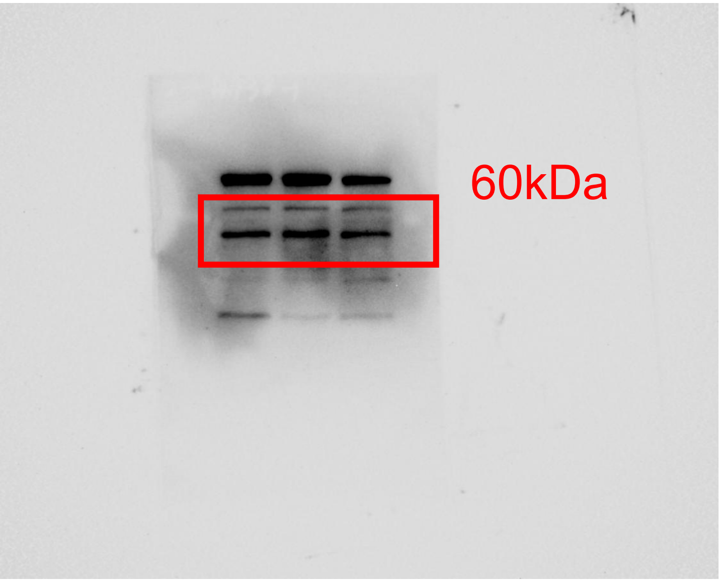


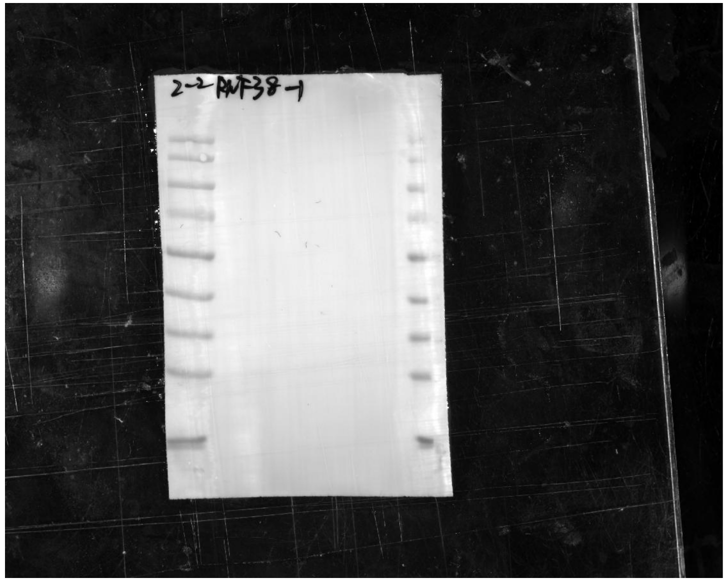

INPUTA549RNF38

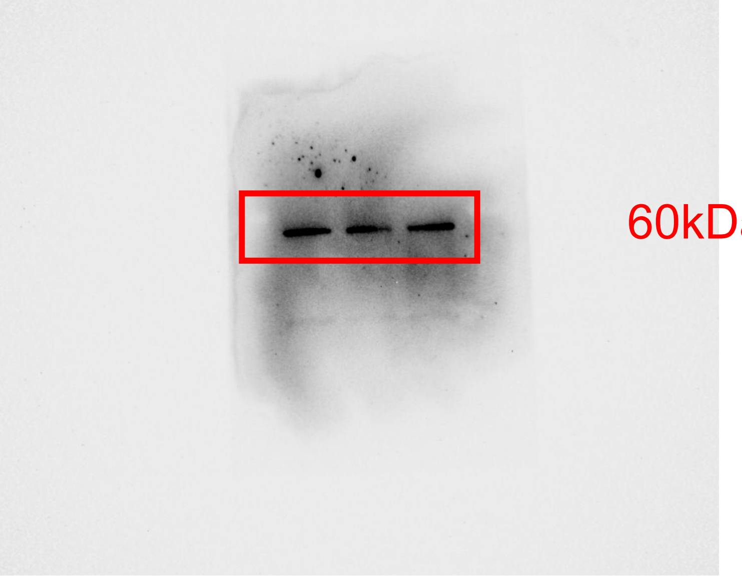


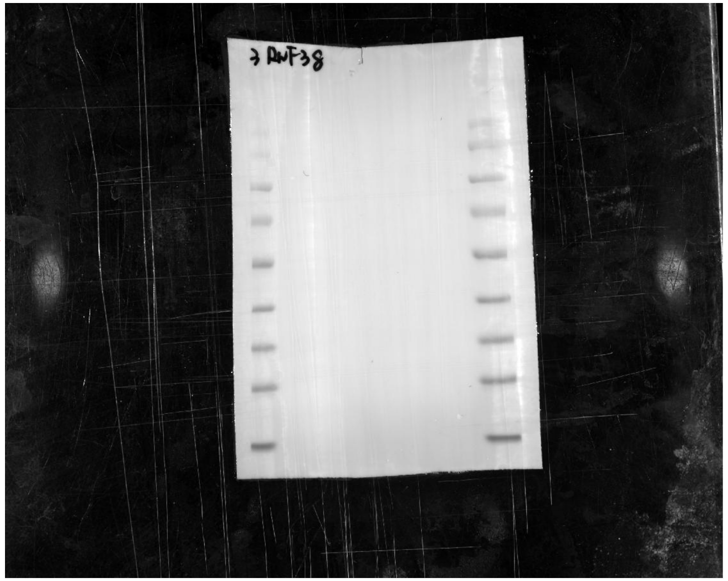

INPUTH1299MYC

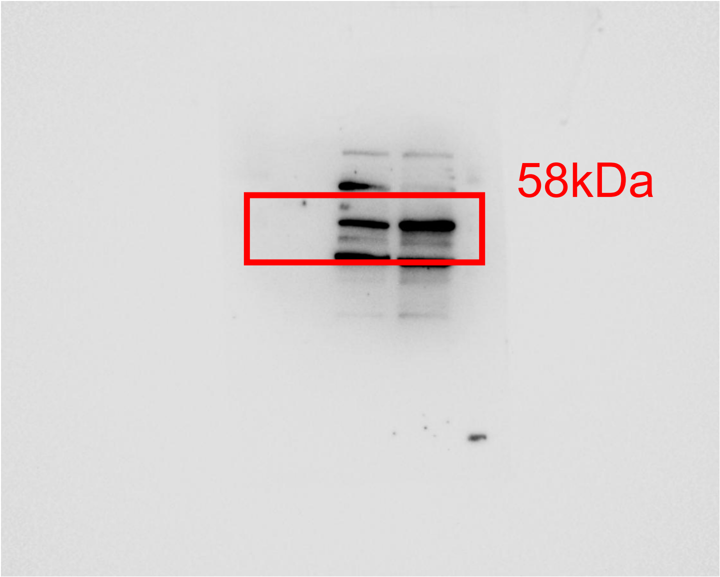


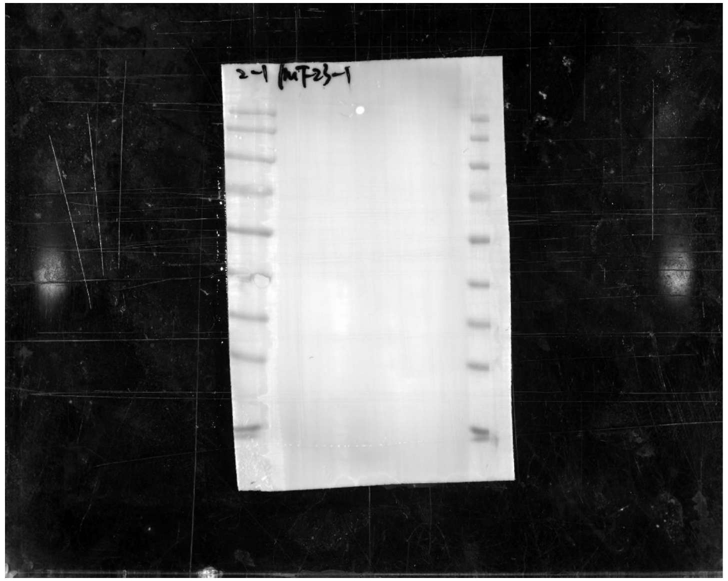

INPUTA549myc

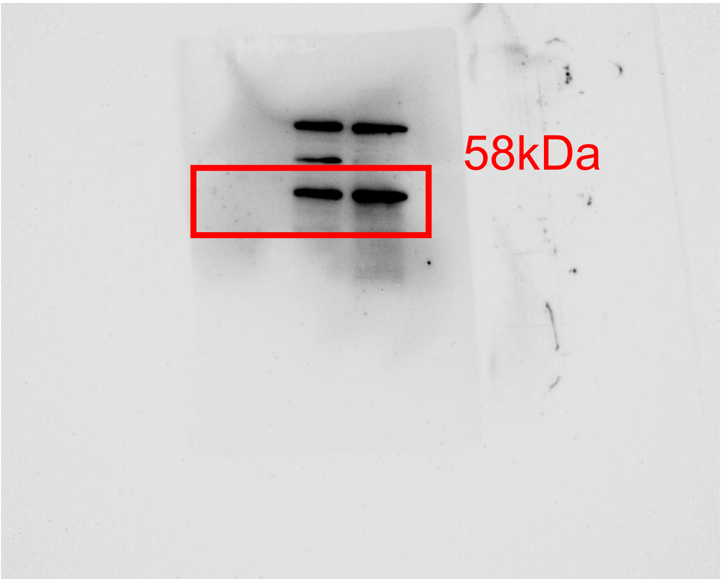


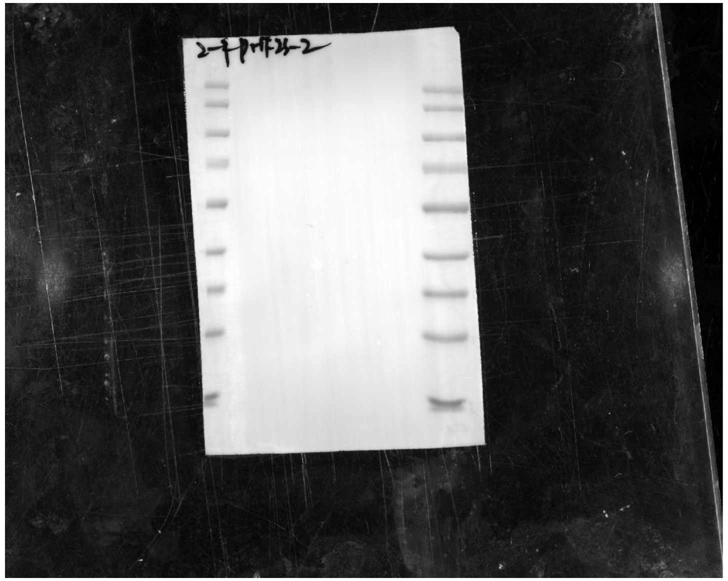

INPUTH1299GFP

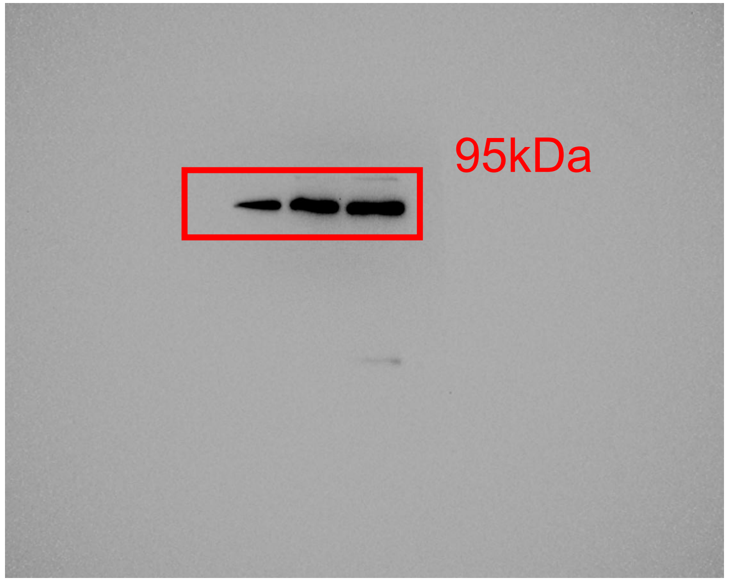


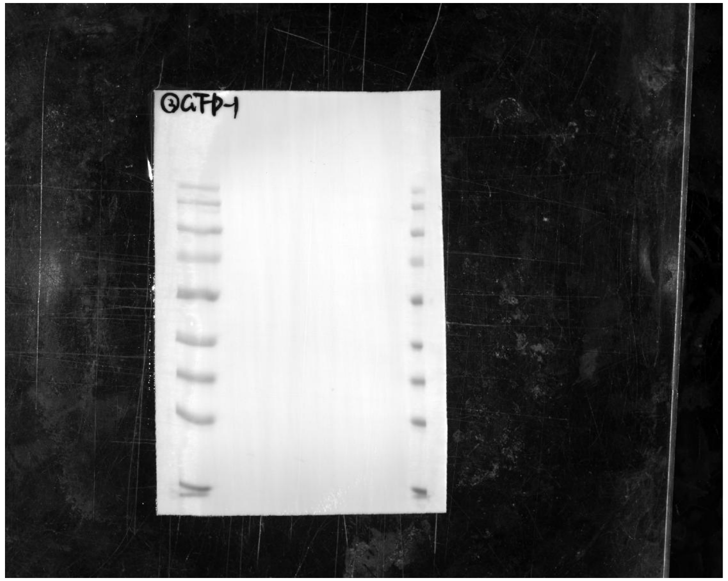

INPUTA549GFP

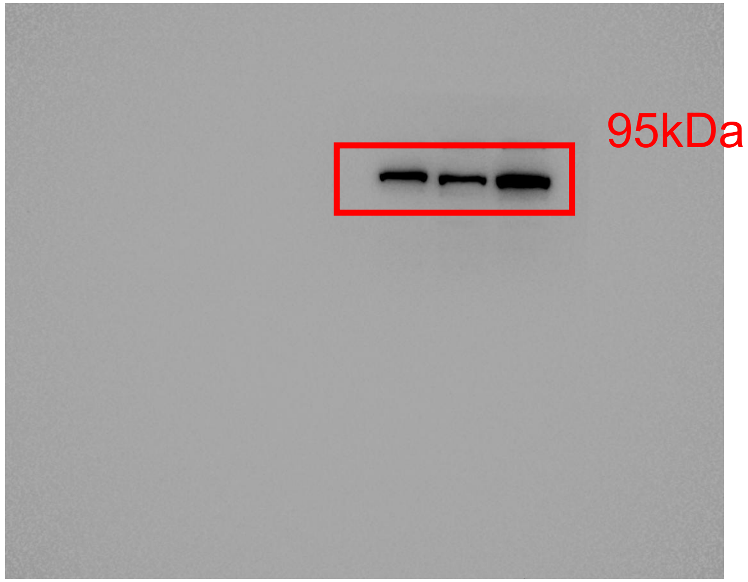


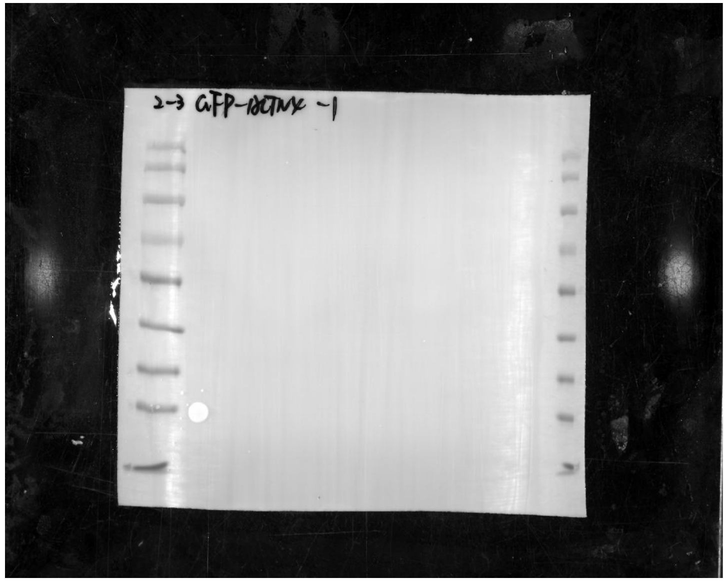

# S-Figure7D

PHF23

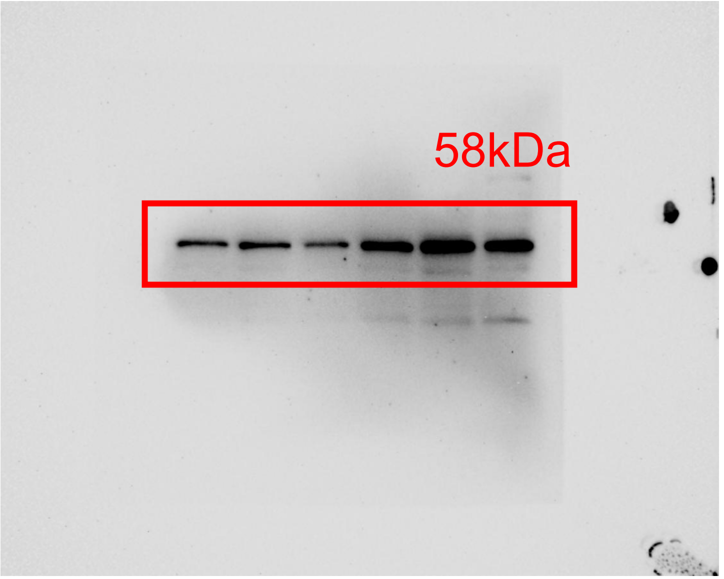


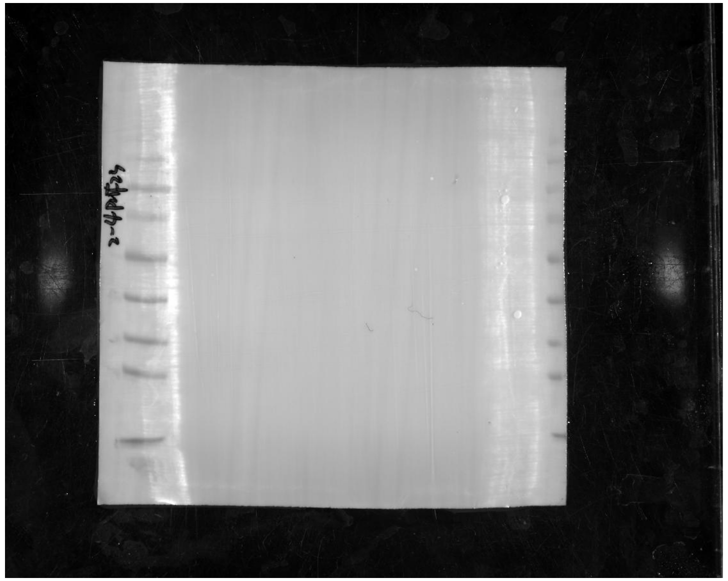

ACTN4

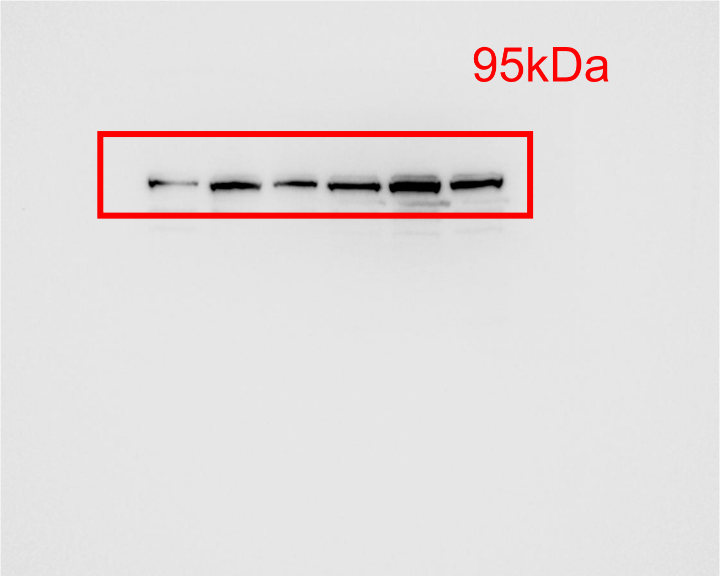


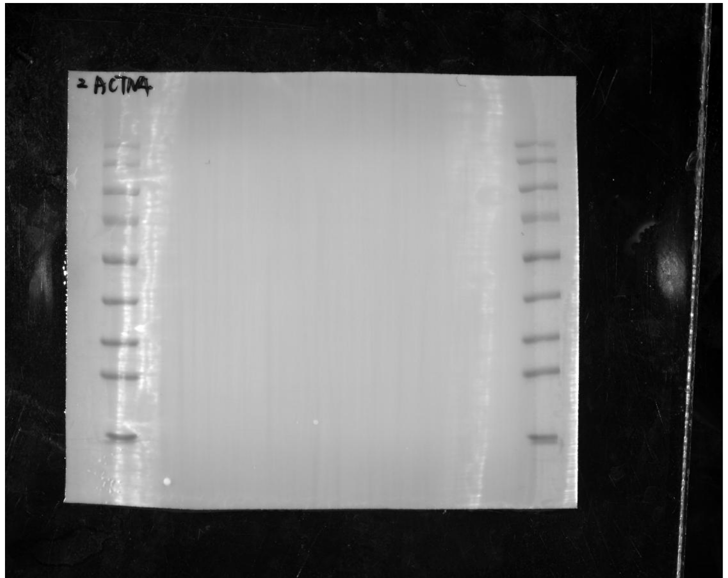

p-ERK

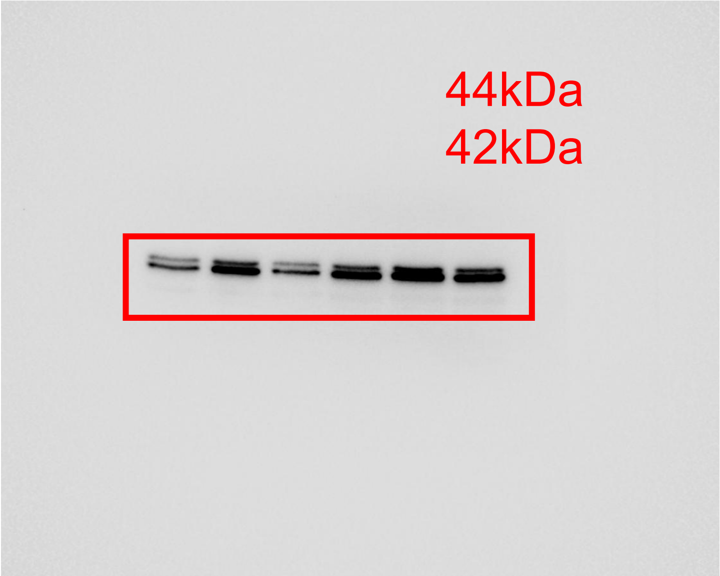


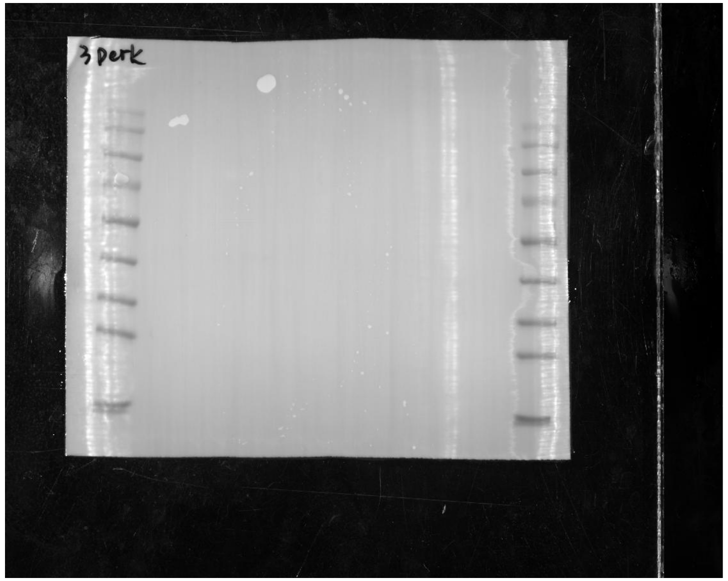

ERK

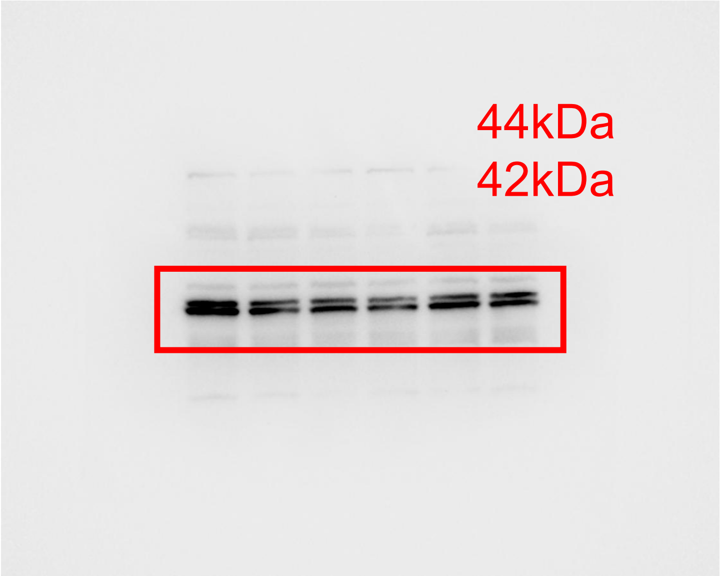


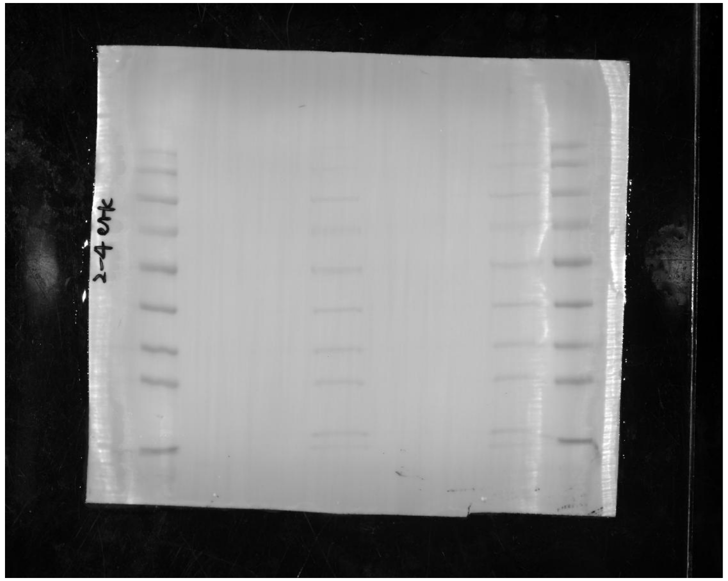

**p-Jun(Ser73)**

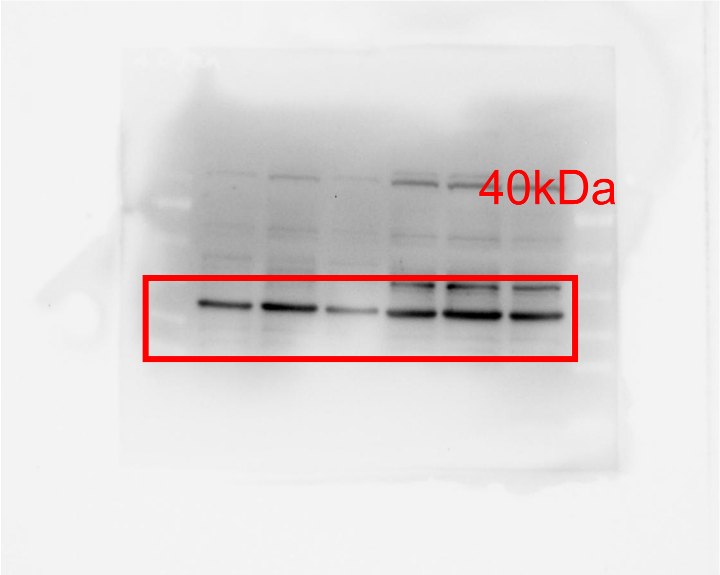


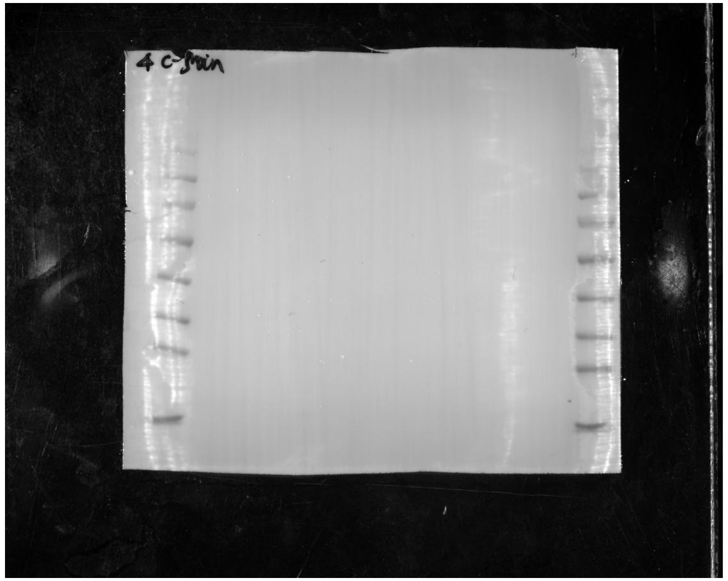

**C-MYC**

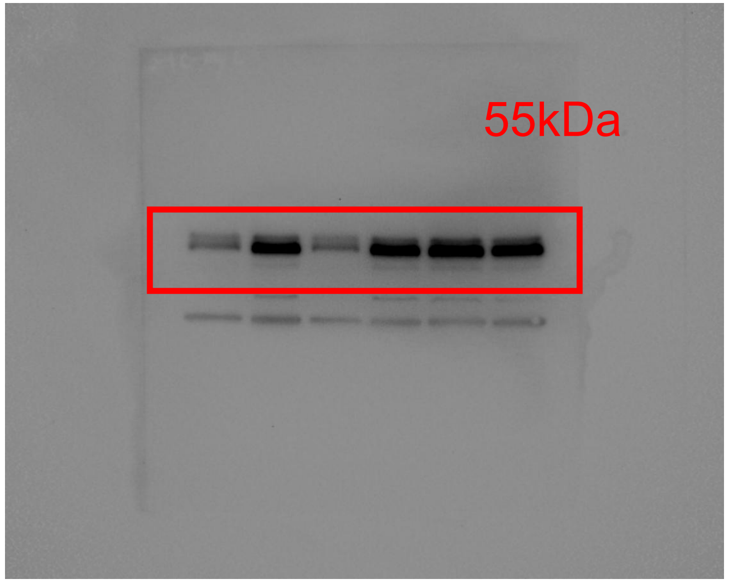


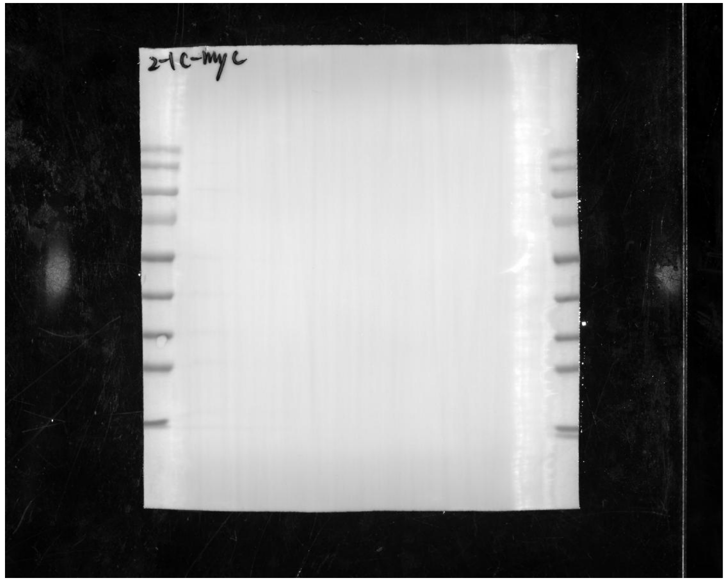

**p38**

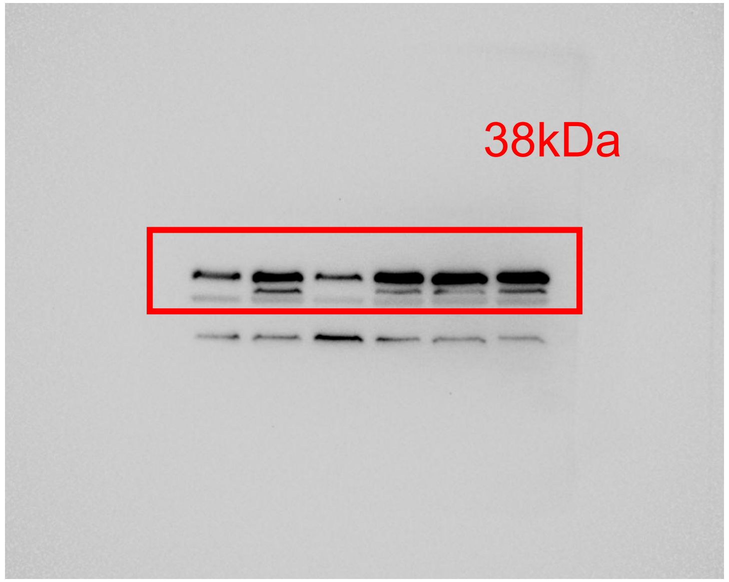


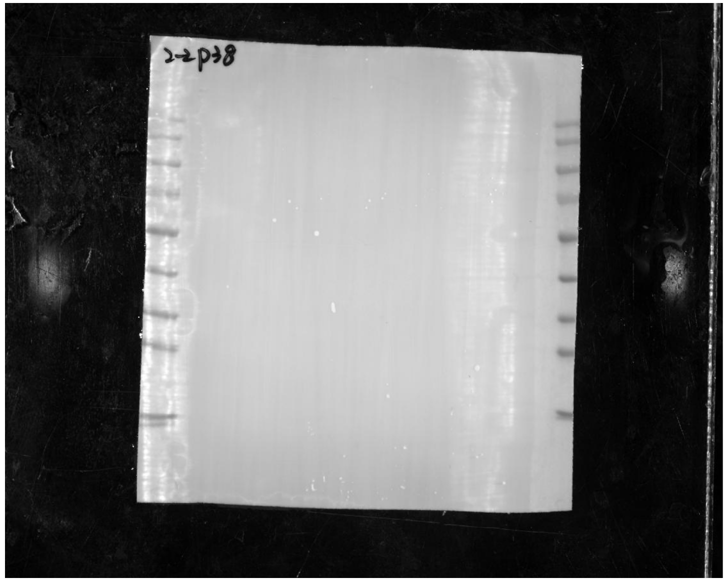

**GAPDH**

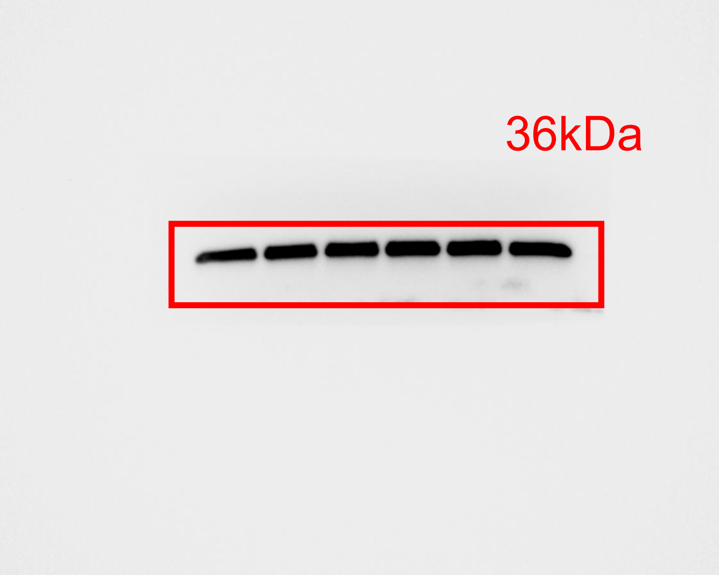


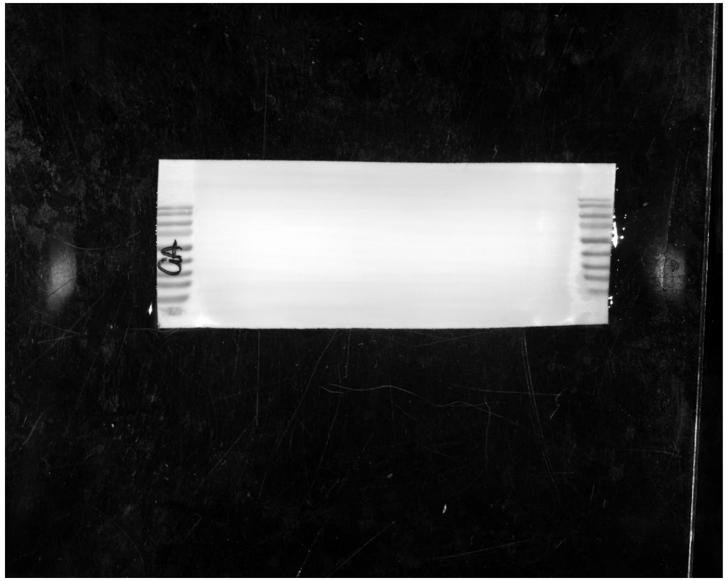

Supplement: Supplementary file 1 — Collated Supplementary Information File [file 41419_2023_6069_MOESM1_ESM.pdf]
